# A neoclerodane orthoester and other new neoclerodane diterpenoids from *Teucrium yemense* chemistry and effect on secretion of insulin

**DOI:** 10.1038/s41598-021-87513-3

**Published:** 2021-04-13

**Authors:** Mohammad Nur-e-Alam, Ifat Parveen, Barrie Wilkinson, Sarfaraz Ahmed, Rahman M. Hafizur, Ahmed Bari, Timothy J. Woodman, Michael D. Threadgill, Adnan J. Al-Rehaily

**Affiliations:** 1 Department of Pharmacognosy, College of Pharmacy, King Saud University, P.O. Box. 2457, Riyadh, 11451 Kingdom of Saudi Arabia; 2 Institute of Biological, Environmental and Rural Sciences (IBERS), Aberystwyth University, Aberystwyth, SY23 3DA UK; 3 John Innes Centre, Norwich Research Park, Norwich, NR4 7UH UK; 4 Dr. Panjwani Center for Molecular Medicine and Drug Research, International Center for Chemical and Biological Sciences, University of Karachi, Karachi, 75270 Pakistan; 5 Department of Pharmaceutical Chemistry, College of Pharmacy, King Saud University, P.O. Box. 2457, Riyadh, 11451 Kingdom of Saudi Arabia; 6 Drug and Target Discovery, Department of Pharmacy and Pharmacology, University of Bath, Claverton Down, Bath, BA2 7AY UK

**Keywords:** Chemical biology, Drug discovery, Plant sciences, Medical research, Chemistry

## Abstract

*Teucrium yemense*, a medicinal plant commonly grown in Saudi Arabia and Yemen, is traditionally used to treat infections, kidney diseases, rheumatism, and diabetes. Extraction of the dried aerial parts of the plant with methanol, followed by further extraction with butanol and chromatography, gave twenty novel neoclerodanes. Their structures, relative configurations and some conformations were determined by MS and 1-D and 2-D NMR techniques. Most were fairly conventional but one contained an unusual stable orthoester, one had its (C-16)–(C-13)–(C-14)–(C-15) (tetrahydro)furan unit present as a succinic anhydride and one had a rearranged carbon skeleton resulting from ring-contraction to give a central octahydroindene bicyclic core, rather than the usual decalin. Mechanisms are proposed for the biosynthetic formation of the orthoester and for the ring-contraction. Four novel neoclerodanes increased the glucose-triggered release of insulin from isolated murine pancreatic islets by more than the standard drug tolbutamide, showing that they are potential leads for the development of new anti-diabetic drugs.

## Introduction

*Teucrium* is a genus of the *Lamiaceae* family. Plants in this large genus are perennial herbs, shrubs and subshrubs but present many different appearances^1^. They are widespread in the Middle East, Southeast Asia, Central and South America and countries surrounding the Mediterranean Sea^2^. Saudi Arabia hosts six species of *Teucrium* and is thought to be one of the original centres in which these plants developed^3^. Various *Teucrium* species have been used traditionally for millennia as diuretic, diaphoretic, antiseptic and antipyretic agents^4^. In Saudi Arabia, they have been used in folk medicine to treat diabetes but several other therapeutic activities have been reported in different countries^4–7^. Plants of this genus have been shown to contain diterpenoids, flavonoids, iridoids, tannins, saponins, alkaloids, sterols, coumarins and glycosides^4,8–10^. One species, *T. yemense* Deflers, is a medicinal plant grown in Saudi Arabia, where it is used traditionally to treat infections, kidney diseases, rheumatism and diabetes^8,11,12^. It is a perennial with a woody base with stems 4–20 cm long; the flowers are pink–pale purple. Moreover, extracts of a related species, *T. polium*, have recently been shown to have activity in animal models of diabetes^13,14^.

We reported previously the isolation and characterisation of six neoclerodanes from an ethyl acetate (EtOAc) extract of *T. yemense*, of which two stimulated the growth of *E. coli* but none had antimicrobial or anthelmintic activity^15^. Nine other neoclerodanes had been identified from this plant by Sattar et al. without evaluation of their biological activity^9^, whereas other neoclerodanes have been isolated from other *Teucrium* species^16,17^. Neoclerodanes have been also characterised from *Scutellaria* species^18,19^ and *Linaria* species^20^, while neoclerodanes from *Salvia* have been identified as inhibitors of HSP90 and as κ-opioid receptor agonists^21–24^. In the present work, we aimed to enlarge our search for bioactive compounds from this species and seek novel structures. We disclose the isolation and structures of twenty new neoclerodanes from the butanol (BuOH) extract of *T. yemense* and report that nine examples enhance the insulin-triggered release of insulin from isolated murine pancreatic islets, indicating potential anti-diabetic activity.

## Materials and methods

### General analytical and chromatographic procedures

See SI. All the methods are carried out in accordance with relevant guidelines and regulations.

### Plant material

*Teucrium yemense* Deflers was collected in February 2014, from Akabat Al-Abna, Baljurashi, Saudi Arabia. The collected material was identified by taxonomist Dr. M. Yusuf, College of Pharmacy, King Saud University (KSU), Riyadh, Saudi Arabia. A voucher specimen (# 15292) has been logged at the herbarium of the College of Pharmacy, KSU.

### Extraction and isolation

The air-dried and coarsely ground powdered aerial parts of *T. yemense* (1.6 kg) were first defatted with hexane and then extracted with MeOH. Evaporation of the solvent from the latter gave a sticky dark mass (388.6 g). This was suspended in water and extracted with EtOAc, then BuOH. Evaporation of the solvent from the BuOH extract gave a residue (43.0 g). Column chromatography (silica gel, mesh size 230–400, hexane → EtOAc/hexane (3:1)) afforded 45 fractions. Fifteen fractions were subjected to radial centrifugal chromatography (Chromatotron) (CH_2_Cl_2_/MeOH 19:1), followed by reverse-phase HPLC (C18 column) to give new compounds **1**–**20**, along with some known compounds. The detailed isolation scheme is shown in the SI as Figure S208.

### Fatimanol F (1)

Colourless gum; IR 3536, 2970, 1761, 1642, 1218 cm^−1^; ^1^H NMR, ^13^C NMR, COSY, HSQC, HMBC, see SI; HRESIMS *m/z* 457 [M + K]^+^, 441.1510 [M + Na]^+^, 419.1690 [M + H]^+^, 401 [M + H—H_2_O]^+^; HRESIMS *m/z* 463.1605 [M + formate]^−^, 453.1316 [M + ^35^Cl]^−^.

### Fatimanone B (2)

Colourless gum; IR 3478, 2937, 1796, 1712, 1325, 1238 cm^−1^; ^1^H NMR, ^13^C NMR, COSY, HSQC, HMBC, see SI; HRESIMS *m/z* 421.1855 [M + Na]^+^.

### Fatimanol G (3)

White amorphous powder; IR 3513, 2401, 1716, 1533, 1202 cm^−1^; ^1^H NMR, ^13^C NMR, COSY, NOESY, HSQC, HMBC, see SI; HRESIMS *m/z* 409 [M + H]^+^, 379.1747 [M + H – H_2_C = O]^+^; HRESIMS *m/z* 407.1690 [M − H]^−^.

### Fatimanol H (4)

Colourless gum; IR 3616, 3014, 1715, 1701, 1575, 1202 cm^−1^; ^1^H NMR, ^13^C NMR, COSY, NOESY, HSQC, HMBC, see SI; HRESIMS *m/z* 361 [M + K]^+^, 383.1113 [M + Na]^+^, 361 [M + H]^+^.

### Fatimanol I (5)

Colourless gum; IR 3618, 2034, 1721, 1763, 1715, 1202 cm^−1^; ^1^H NMR, ^13^C NMR, COSY, HSQC, HMBC, see SI; HRESIMS (+ ve) *m/z* 827.2509 [2 M + Na]^+^, 425.1197 [M + Na]^+^, 403.1378 [M + H]^+^.

### Fatimanol J (6)

Colourless gum; IR 3593, 3132, 1731, 1726, 1704, 1303, 1271 cm^−1^; ^1^H NMR, ^13^C NMR, COSY, HSQC, HMBC, see SI; HRESIMS *m/z* 397.1248 [M + Na]^+^, 375.1429.

### Fatimanol K (7)

Colourless gum; IR 3433, 2971, 1769, 1737, 1221 cm^−1^; ^1^H NMR, ^13^C NMR, COSY, NOESY, HSQC, HMBC, see SI; HRESIMS *m/z* 425.1459 [M − H]^−^.

### Fatimanol L (8)

White amorphous powder; IR 3615, 3062, 1744, 1734, 1695 cm^−1^; ^1^H NMR, ^13^C NMR, COSY, NOESY, HSQC, HMBC, see SI; HRESIMS *m/z* 519.2212 [M + Na]^+^.

### Fatimanol M (9)

Colourless gum; IR 3546, 2965, 1764, 1752, 1708 cm^−1^; ^1^H NMR, ^13^C NMR, COSY, NOESY, HSQC, HMBC, see SI; HRESIMS *m/z* 535.2189 [M + Na]^+^, 513.2350 [M + H]^+^.

### Fatimanol N (10)

Colourless gum; IR: 3631, 3114, 1795, 1790, 1689, 1262, 1132 cm^−1^; ^1^H NMR, ^13^C NMR, COSY, HSQC, HMBC, see SI; HRESIMS *m/z* 427.1596 [M + H]^+^; HRESIMS (− ve) *m/z* 425.1452 [M − H]^−^.

### Fatimanol O (11)

Colourless gum; IR 3414, 3002, 1621, 1417, 1348 cm^−1^; ^1^H NMR, ^13^C NMR, COSY, NOESY, HSQC, HMBC, see SI.

### Fatimanol P (12)

Colourless gum; IR 3584, 3414, 1716, 1419, 1351 cm^−1^; ^1^H NMR, ^13^C NMR, COSY, NOESY, HSQC, HMBC, see SI; HRESIMS *m/z* 443.1706 [M + Na]^+^, 421.1854 [M + H]^+^.

### Fatimanol Q (13)

Colourless gum; ^1^H NMR, ^13^C NMR, 135DEPT, COSY, HSQC, HMBC, see SI; HRESIMS *m/z* 787.3875 [2 M + Na]^+^, 729.3844 [2 M + Na—C_2_H_2_O_2_]^+^, 405.1880 [M + Na]^+^, 347.1850 [M + Na – C_2_H_2_O_2_]^+^, 329.1744 [M + Na—C_2_H_2_O_2_—H_2_O]^+^, 311.1639 [M + Na–C_2_H_2_O_2_—2 × H_2_O]^+^; HRESIMS *m/z* 427.1970 [M + formate]^−^, 417.1689 [M + ^35^Cl]^−^, 381.1920 [M – H]^−^.

### Fatimanol R (14)

Colourless gum; ^1^H NMR, ^13^C NMR, 135DEPT, COSY, HSQC, HMBC, see SI; HRESIMS *m/z* 871.4083 [2 M + Na]^+^, 447.1985 [M + Na]^+^, 425.2167 [M + H]^+^; HRESIMS *m/z* 469.2075 [M + formate]^−^, 459.1795 [M + ^35^Cl]^−^.

### Fatimanol S (15)

Colourless gum; ^1^H NMR, ^13^C NMR, 135DEPT, COSY, NOESY, HSQC, HMBC, see SI; HRESIMS *m/z* 779.3248 [2 M + Na]^+^, 401.1567 [M + Na]^+^, 379.1748 [M + H]^+^, 361.1642 [M + H − H_2_O]^+^, 343.1537 [M + H − 2 × H_2_O]^+^, 325.1341 [M + H—3 × H_2_O]^+^; HRESIMS *m/z* 423.1656 [M + formate]^−^, 377.1605 [M − H]^−^.

### Fatimanol T (16)

Colourless gum; ^1^H NMR, ^13^C NMR, 135DEPT, COSY, NOESY, HSQC, HMBC, see SI; HRESIMS *m/z* 779.3250 [2 M + Na]^+^, 401.1568 [M + Na]^+^, 379.1748 [M + H]^+^, 361.1643 [M + H − H_2_O]^+^, 325.1432 [M + H − 3 × H_2_O]^+^.

### Fatimanol U (17)

Colourless gum; ^1^H NMR, ^13^C NMR, 135DEPT, COSY, HSQC, HMBC, see SI; HRESIMS 983.3881 [2 M + Na]^+^, 503.1886 [M + Na]^+^, 463.1961 [M + H − H_2_O]^+^, 445.1856 [M + H − 2 × H_2_O]^+^, 403.1750 [M + H − H_2_O − AcOH]^+^, 361.1644 [M + H − 2 × AcOH]^+^; HRESIMS *m/z* 525.1974 [M + formate]^−^, 515.1693 [M + ^35^Cl]^−^, 479.1923 [M − H]^−^.

### Fatimanol V (18)

Colourless gum; ^1^H NMR, ^13^C NMR, 135DEPT, COSY, HSQC, HMBC, see SI; HRESIMS *m/z* 803.3255 [2 M + Na]^+^, 413.1568 [M + Na]^+^.

### Fatimanol W (19)

Colourless wax; ^1^H NMR, ^13^C NMR, 135DEPT, HSQC, HMBC, see SI.

### Fatimanol X (20)

Colourless gum; ^1^H NMR, ^13^C NMR, 135DEPT, HSQC, HMBC, see SI.

## Results and discussion

**Figure 1:**
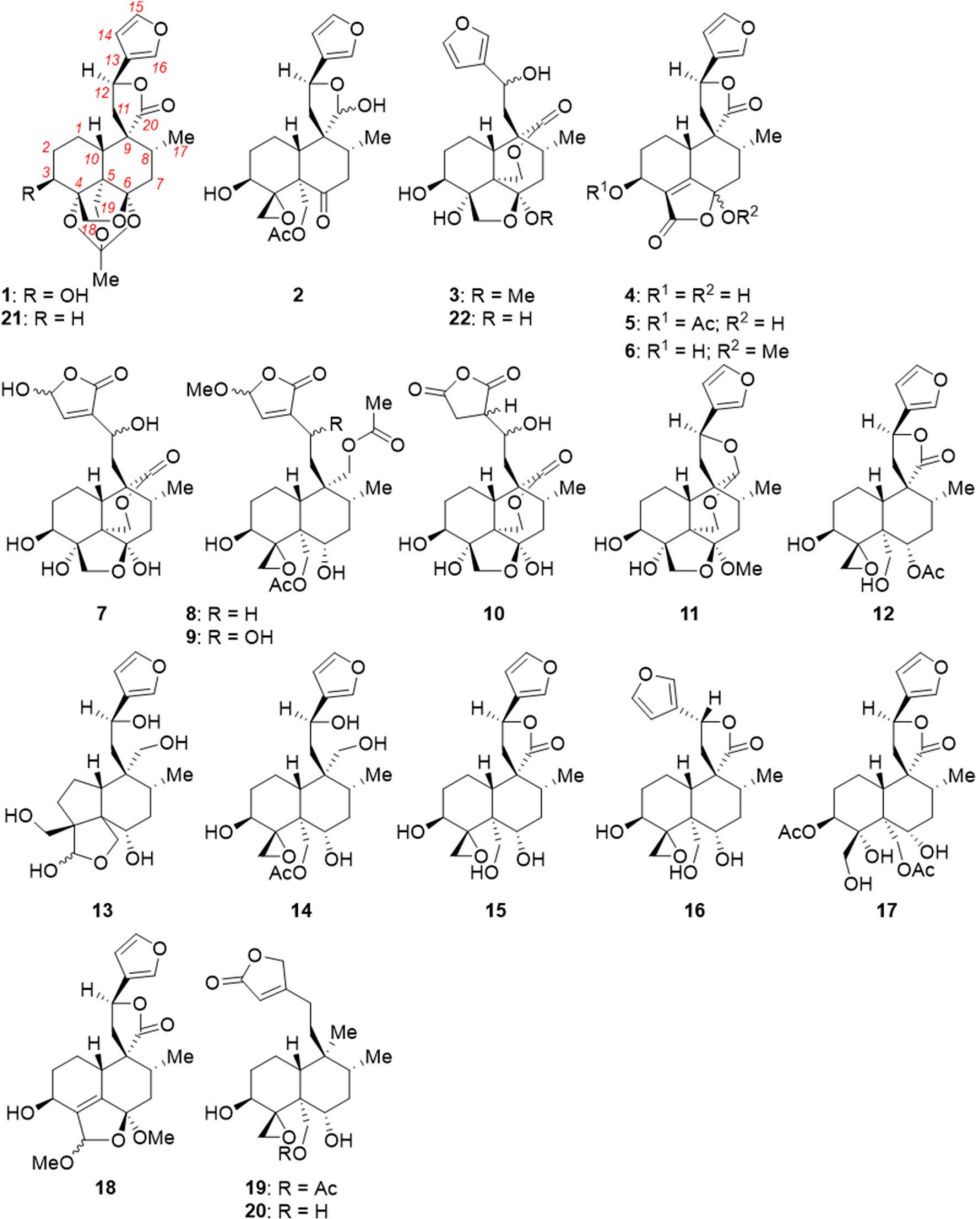
Structures of new neoclerodanes isolated from *Teucrium yemense* (**1**–**20**), of the product **21** of pyrolysis of 19-acetylgnaphalin^27^, and of previously isolated teulepicephin **22**^15^. The numbering of the carbon atoms is shown on **1**.

The dried aerial parts of the plant were defatted and extracted with methanol (MeOH). This solvent was evaporated and the residue was extracted with EtOAc, then extracted with BuOH. The BuOH extract was separated by column chromatography on silica gel. Radial chromatography and HPLC yielded twenty pure compounds (Fig. 1). Their structures were elucidated using 1D and 2D nuclear magnetic resonance (NMR) and high-resolution electrospray ionisation mass spectrometry (HRESIMS) data. Their absolute configurations cannot be confirmed from these data but are assumed on the basis of precedent for related compounds^9,25,26^.

### Compound 1

HRESIMS showed *pseudo*molecular ions at *m/z* 457 [M + K]^+^, *m/z* 441.1509 [M + Na]^+^ (calc 441.1525) and *m/z* 419.1690 [M + H]^+^ (calc 419.1706), for the formula C_22_H_26_O_8_. The ion at *m/z* 401 [M + H — H_2_O]^+^ indicated a hydroxy group. Negative *pseudo*molecular ions were seen at *m/z* 463.1605 [M + formate]^−^ (calc 463.1605) and *m/z* 453.1316 [M + ^35^Cl]^−^ (calc 453.1316). The ^13^C NMR spectrum (Table S1, Supplementary Information (SI)) showed 22 discrete resonances: 2 × CH_3_, 6 × CH_2_, 7 × CH, 7 × C_q_. The core structure was shown to be a decalin and related to the neoclerodane diterpenoids^9,15,16,25^. The infra-red (IR) spectrum showed an OH (3536 cm^−1^) and one γ-lactone carbonyl peak (1761 cm^−1^).

In the upper part of the structure of **1** (Fig. 1), the aromatic furan was characterised by ^1^H NMR signals at δ 6.47 (H-14), δ 7.54 (H-15) and δ 7.61 (H-16) (Table S1, SI). A ^1^H–^1^H correlation spectrum (COSY) cross-peak linked δ 6.47 (H-14) and δ 7.54 (H-15). Heteronuclear single quantum (HSQC) correlation linked the ^1^H signals to ^13^C signals at δ 109.1 (C-14), δ 145.6 (C-15) and δ 141.80 (C-16); the signal for C-13 (δ 126.3) was identified by a strong 3-bond heteronuclear multi-bond (HMBC) correlation to H-15 and weaker 2-bond correlations to H-14 and H-16. These signals match well with those observed previously for the furans in fatimanol B, fatimanol D and fatimanol E.^15^ The *spiro*-lactone was identified through the chemical shift of H-12 (δ 5.46) (*cf*. signals in fatimanol B (δ 5.51) and fatimanol D (δ 5.46)^15^). HMBC tied this proton signal to each furan ^13^C signal. COSY correlation linked H-12 to the doublet signal at δ 2.49 (2 H) and a 2-bond HMBC correlation C-11 at δ 41. Although the two H-11 protons are formally diastereotopic, they are coincident for fatimanol D^15^ and for **1** (δ 2.49). No 3-bond HMBC cross-peaks were seen linking H-12 to the lactone carbonyl (20-C, δ 179.27) or the *spiro*-carbon (9-C, δ 49.06), although examination of the MM2-minimised conformation indicated that the corresponding dihedral angles (H-12)–(C-12)–(O)–(C-9) and (H-12)–(C-12)–(C-11)–(C-9) are very close to 90°, the coupling constant minimum in the Karplus relationship. This model also suggested a rigid *trans-*decalin conformation for the lower part of **1**. This conformational and configurational assignment was supported by H-10 resonating as a broad doublet at δ 1.89 with ^3^*J* = 11.2 Hz for a *trans*-diaxial coupling with H-1_ax_. A nuclear Overhauser effect correlation spectroscopy (NOESY) experiment ((CD_3_)_2_SO solvent) showed a cross-peak between H-18_endo_ (δ 3.69 (δ 3.87 in CD_3_OD)) and H-11 (δ 2.49), thus C-18 (δ 58.3 in CD_3_OD) is axial. H-3 was identified by its chemical shift (δ 4.25) and by HMBC correlations to C-1 (δ 29) and C-4 (δ 87.1). C-3 (δ 71.4) correlated by HSQC to H-3 and by HMBC to both 1-H (δ 1.29 and δ 1.60) and to both 2-H (δ 1.60 and δ 2.20). H-3 had a *trans*-diaxial coupling with H-2_ax_ (^3^*J* = 10.9 Hz) and was thus axial, making 3-OH equatorial and confirming the conformation of ring A as chair. A strong NOESY correlation between 3-H and H-19_exo_ (δ 3.91) showed that CH_2_-19 was close in space to H-3 and also axial. With H-10 and CH_2_-19 both axial, the decalin must be *trans*-fused. CH_3_-17 resonated as expected as a doublet at δ 1.08 (^1^H) and δ 16.1 (^13^C), linked by HSQC . Strong 3-bond HMBC cross-peaks from H_3_-17 to C-9 (δ 49.1) and to C-7 (δ 35.2) and from C-17 to H-7_ax_ (δ 2.27) confirmed this methyl. The ^1^H signal for H-7_ax_ was a dd (^2^*J*_(H-7ax)−(H-7 eq)_ = 14.1 Hz, ^2^*J*_(H-7ax)−(H-8)_ = 12.9 Hz), showing that H-8 is axial and, therefore, CH_3_-17 is equatorial. The NOESY experiment ((CD_3_)_2_SO) showed a cross-peak between H_3_-20 and the H-11 resonance at δ 2.36 (δ 2.49 in CD_3_OD), which is only possible if CH_3_-17 is equatorial.

The orthoacetate unit was more challenging to identify. The CH_3_ protons gave a singlet at δ 1.43, which is inappropriate for an acetate ester, with the ^13^*C*H_3_ signal at δ 23.9. 2-Bond HMBC linked this CH_3_ to the orthoester ^13^C signal at δ 107.7 / 107.8, which is inappropriate for an ester carbonyl. Thus this 2-carbon unit was not a conventional acetate ester, which was consistent with no loss of 60 Da (HOAc) in the MS. The ^1^H NMR spectrum in (CD_3_)_2_SO showed only one OH resonance (HO-3, δ 5.23, with COSY and NOESY correlations with H-3). H-18_endo_ (δ 3.87) and H-18_exo_ both formed HMBC cross-peaks with the ^13^C signal(s) at δ 107.7 / 107.8. Thus one of these signals must have been due to the orthoester carbon (four bonds from H_2_-18) and the other due to acetal carbon C-5 (three bonds from H_2_-18). This confirmed the ring-closure of the (C-6)–(C-5)–(C-4)–(C-18)–(O) tetrahydrofuran. C-4 (δ 87.1), C-18 (δ 58.3) and C-19 (δ 74.6) all carry oxygen as shown by their ^13^C chemical shifts. These data are consistent with the orthoacetate structure for **1** (fatimanol F, Fig. 1). Analogue **21**, which contains the acetal and orthoacetate structures but differs from **1** in lacking HO-3, was reported^27^ as a product of the pyrolysis of 19-acetylgnaphalin.

### Compound 2

HRESIMS showed a *pseudo*molecular ion at *m/z* 421.1855 [M + H]^+^ (calc 421.1862) for the formula C_22_H_28_O_8_. Fragments were seen at *m/z* 379 [M + H − ketene]^+^ and *m/z* 361 [M + H − AcOH]^+^, showing an acetate ester. Twenty-two discrete ^13^C NMR signals were observed (2 × CH_3_, 6 × CH_2_, 8 × CH, 6 × C_q_ (ester and ketone)). IR confirmed these carbonyls, with bands at 1712 cm^−1^ and 1796 cm^−1^, respectively. An OH absorbed at 3478 cm^−1^.

The NMR data (Table S1, SI) showed that **2** to be a neoclerodane. The aromatic furan was shown by ^1^H NMR signals at δ 6.41 (H-14), δ 7.43 (H-15) and δ 7.42 (H-16), with HSQC correlations to C-14 (δ 108.8), C-15 (δ 143.8) and C-16 (δ 139.6), respectively. The C-13 signal (δ 124.8) was identified by HMBC correlations with H-14, H-15, and H-16. C-13, C-14, and C-16 showed HMBC cross-peaks with a double doublet (dd) aliphatic proton signal at δ 5.25 (H-12). HSQC correlated this with C-12 *(δ* 71.2). The corresponding signal for H-12 in the lactone **1** is downfield at δ 5.46, whereas H-12 in the alcohol **3** resonates upfield at δ 4.85. These comparisons suggest that the electron-density at H-12 in **2** is intermediate between that in the lactone **1** and the alcohol **3** and is consistent with the hemiacetal/lactol structure in **2**. A strong 3-bond HMBC cross-peak linked C-12 with the hemiacetal H-20 singlet at δ 5.54. HSQC identified C-20 (δ 99.7). The lactol was completed by identification of both H-11 signals (δ 1.93 dd, δ 2.34 dd) by HBMC correlations with C-20, location of C-12 (δ 44.2) by HSQC correlation with H-12 and characterisation of the quaternary *spiro* carbon C-9 *(δ* 53.6) by HMBC correlations with H-12 and H-20. COSY linked both H-11 and H-12. In the lower part, the OH group was located at C-3 through the chemical shifts of H-3 (δ 4.11) and C-3 *(δ* 66.4). H-3 had been identified by HMBC correlation to C-5 (δ 63.6) and C-3 had been identified by HMBC correlation to H-1 (δ 2.22 and δ 2.64) and H-2 (δ 1.36 and δ 2.22). Observation of two geminally coupled doublets (H-18) at δ 2.81 and δ 3.14 (^2^*J* = 5.3 Hz) revealed the *spiro*-oxirane. This was shown to be at C-4 by HMBC from these H-18 protons to C-5 (δ 63.6) and from H-3 to C-18 (δ 43.7). C-6 was a ketone, as shown by its chemical shift (δ 206.2) and by HMBC cross-peaks to both H-7 signals (δ 2.28 and δ 2.72) and a 4-bond HMBC correlation with CH_3_-17. Examination of a model of **2** suggested that ring B was in a flattened-chair conformation, such that the dihedral angles (C-6)–(C-5)–(C-10)–(H-10) and (C-6)–(C-7)–(C-8)–(H-8) were close to 90°, explaining the lack of these 3-bond HMBC interactions. The acetoxymethyl (AcOCH_2_−) group was defined by CH_3_-2′ (δ_H_ 2.07, δ_C_ 21.1) and the ester carbonyl C-1′ (δ 171.2). HMBC cross-peaks between C-1′ and the geminally coupled doublets for H_2_-19 (δ 4.72, δ 4.83) confirmed the AcOCH_2_-; HMBC cross-peaks from H_2_-19 to C-6, to quaternary C-5 and to the *spiro*-oxirane carbon (C-4) demostrated that this unit was located at C-5. Relevant ^3^* J* coupling constants showed that rings A and B were shown to be in chair conformations. For example, the H-7_ax_ signal (δ 2.72) was a broad triplet with ^3^*J* = ^2^*J* = 14.6 Hz, indicating diaxial and geminal couplings, respectively. H-7_ eq_ (δ 2.28) only showed axial-equatorial and geminal couplings. Thus Me-17 is equatorial and H-8 is axial and the chair conformation is confirmed. The boat conformation would have shown only eq–eq and eq–ax couplings for ^3^*J*_(H-7)–(H-8)_.

The NMR spectra showed, in addition to peaks for this major compound, a full set of peaks for a minor component, with similar chemical shifts and multiplicities. This minor set of peaks integrated for *ca*. 10% of the major compound present. As the sample gave only one pure peak on HPLC, we ascribed these peaks to a minor diastereoisomer in slow equilibrium with the major diastereoisomer. These are likely to be epimers at the lactol hemiacetal C-20. This assignment was supported by the largest differences in chemical shift between the epimers being for H-3 (Δδ 0.1 ppm), H-14 (Δδ 0.05 ppm), and H_2_-19 (Δδ 0.2), as these four protons are close in space to the epimeric C-20. We assign the structure **2** (Fig. 1) to fatimanone B.

### Compound 3

Negative-ion HRESIMS showed ions at *m/z* 407.1690 [M − H]^−^ (calc 407.1706) for the formula C_21_H_28_O_8_. Positive-ion HRESIMS revealed a *pseudo*molecular ion at *m/z* 409 [M + H]^+^ and a fragment at *m/z* 379.1747 [M + H – H_2_C = O]^+^ (calc 379.1757). Twenty-one discrete ^13^C NMR signals were observed: 2 × CH_3_, 6 × CH_2_, 7 × CH, 6 × C_q_, including a carbonyl (δ 172.5). IR showed bands for OH (3513 cm^−1^) and one carbonyl (1716 cm^−1^).

The NMR data (Table S1, SI) indicated that **3** was a neoclerodane. The upper part was an aromatic furan, with ^1^H NMR signals (Table S1, SI) at δ 6.42 (H-14), δ 7.39 (H-16) and δ 7.40 (H-15). The furan ^13^C NMR signals were at δ 108.4 (C-14), δ 130.1 (C-13), δ 138.6 (C-16) and δ 143.9 (C-15), with appropriate HSQC and HMBC connectivities. The chemical shift of H-12 (δ 4.85) showed that it was not part of a lactone or lactol system, confirmed by the lack of a HMBC cross-peak to the signal for carbonyl C-20 (δ 172.5). It did show HMBC cross-peaks to C-13, C-14, and C-16. Further HMBC cross-peaks were seen from H-12 to C-11 (δ 35.8 or δ 40.0) and to C-9 (δ 48.5), linking this upper side-chain to the main decalin. The configuration at C-12 could not be established. The bridging lactone was established by HMBC cross-peaks between carbonyl C-20 and H_2_-19 (δ 4.45, δ 4.68). H_2_-19 had been confirmed by each signal being a doublet with only geminal coupling (^2^*J* = 12.5 Hz) and by HMBC cross-peaks to C-6 (δ 110.0) and to C-4 (δ 84.1). C-19 (δ 66.4) was identified by HSQC to H_2_-19 and by strong HMBC to H-10 (δ 2.24–2.48 m). The signal at δ 72.8 was assigned to C-3 by HMBC cross-peaks to both H-2 (δ 1.4, δ 2.1) and to one of the H-1 signals (δ 2.35). H-3 *(δ* 3.87) was characterised by a HSQC cross-peak to C-3, COSY to both H-2 and HMBC to C-1 (δ 21.9), C-2 (δ 30.3) and C-4 (δ 84.1). H-3 is axial, with large ^3^*J*_ax-ax_ (11.6 Hz) to H-2_ax_. The H-1 signal at δ 1.29 is a quartet (*J* = 13.2 Hz); thus it is H-1_ax_. NOESY correlations from H-3 to one H-19 (δ 4.45) and to H-1_ax_ showed that these are on the same face of the decalin; thus the decalin is *trans*-fused and that ring A is in the chair conformation. The conformation of ring B is less clear, owing to overlap of ^1^H NMR signals but MM2-minimisation suggested that it may be a flattened boat. Acetal carbon C-6 was characterised by its chemical shift (δ 110.0) and by HMBC cross-peaks to both H-19. Further HMBC correlations were seen to the doublets at δ_H_ 3.90 and δ_H_ 4.45, showing that they were due to H_2_-18 and demonstrating the tetrahydrofuran ring. The acetal at C-6 was identified by observation of an OMe (δ_H_ 3.40, δ_C_ 48.9), linked by HMBC to C-6. These data characterise the structure of **3**, fatimanol G (Fig. 1). This structure is identical to that of teulepicephin **22**, with the exception of the acetal (hemiacetal in **14**)^15^; the spectroscopic features are very similar, suggesting a similar conformation.

### Compound 4

HRESIMS showed *pseudo*molecular ion peaks at *m/z* 383.1113 [M + Na]^+^ (calc 383.1107) and *m/z* 384.1148, [M + Na]^+^ (calc for ^12^C_18_^13^CH_20_NaO_7_, 384.1140), appropriate to the formula C_19_H_20_O_7_, with low-intensity ions at *m/z* 361 [M + H]^+^ and *m/z* 399 [M + K]^+^. The ^13^C NMR spectrum contained signals for 19 discrete carbons: 1 × CH_3_, 4 × CH_2_, 7 × CH, 7 × C_q_ (including two C = O). The IR indicated hydroxy groups (3614 cm^−1^) and two carbonyls (1715, 1701 cm^−1^).

The ^1^H NMR spectrum (Table S2, SI) contained two very similar sets of signals, in *ca*. 1:1 ratio, suggesting diastereoisomers which interconverted slowly on the ^1^H NMR timescale. This may indicate a cyclic hemiacetal (*cf*. **2**). Detailed assignment of the signals was challenging, as many overlapped between the two stereoisomers. Taken together, the NMR data showed that **4** had a neoclerodane core. As for **1**–**3**, the upper part was an aromatic furan, with ^1^H NMR signals at δ 6.49 (H-14), δ 7.55 (H-15) and δ 7.61 (H-16). The signals for H-15 and H-16 were distinguished by a NOESY cross-peak from the former to H-14. The ^13^C NMR signals for this ring were at δ 124.7 (C-13), δ 107.9 (C-14), δ 139.6 (C-15) and δ 144.3 (C-16), as identified by HSQC and HMBC. The *spiro*-lactone was initially identified by the chemical shift of H-12 (δ 5.59), corresponding to a benzylic ester. This showed an HSQC cross-peak to C-12 *(δ* 72.3) and HMBC cross-peaks to C-13, C-14, C-16, and C-11 (δ 40.2). HSQC then identified δ 2.73 dd as being due to one H-11 and the two signals at δ 2.56 and δ 2.57 (both dd, each integrating for 0.5 H) as due to the other H-11. The ^13^C signal at δ 176.4 was shown to be the lactone carbonyl C-20 by HMBC to H-11 (δ 2.73), H-8 (δ 2.2), and H-10 (δ 2.85). H-3 (δ 4.41) and C-3 (δ 60.5) had the expected downfield chemical shifts arising from the OH. COSY then identified H-2 at δ 1.66 and δ 2.10, with HSQC showing C-2 (δ 21.6). Further COSY cross-peaks then showed the resonances for H-1 at δ 1.64 and δ 2.3. The lower fused butenolide became evident through HMBC cross-peaks from H-10 to the alkene C_q_ peaks for C-4 (δ 128.5) and C-5 (δ 163.0). These were distinguished by their chemical shifts and by observation of HMBC from C-4 to H-2 (δ 2.01). The carbonyl C-18 was at δ 170.1. The lactone was completed by C-6 (δ 102.4), which shows HMBC correlations with both H_2_-7, H-8 and H-10. Interestingly, there is also a weak 4-bond HMBC cross-peak between H_3_C-17 and C-6. The overlap of many of the ^1^H NMR signals for the two diastereoisomers precluded detailed assignments of the conformations of the decalins. We assign structure **4** (Fig. 1) to this compound, fatimanol H.

### Compound 5

HRESIMS showed *pseudo*molecular ions at *m/z* 425.1197 [M + Na]^+^ (calc 425.1212) and *m/z* 403.1378 [M + H]^+^ (calc 403.1393), for the formula C_21_H_22_O_8_. There was also a peak at *m/z* 827.2509 [2 M + Na]^+^ (calc 827.2527). The ^13^C NMR spectrum contained signals for 21 discrete carbons: 2 × CH_3_, 4 × CH_2_, 7 × CH, 8 × C_q_. The IR showed absorbances for OH (3618 cm^−1^) and three carbonyls (1763, 1715, 1701 cm^−1^). The NMR spectra (Table S2, SI) were very similar to those for **4**, with the exception of additional methyl signals at δ_H_ 2.05 / δ_C_ 20.9, an additional C = O signal at δ_C_ 172.1 and a marked downfield change in the chemical shifts of H-3 (δ 5.59, Δδ 1.18 ppm) and C-3 (δ 64.65, Δδ 4.16 ppm). These indicate that **5** is the 3-O-acetate ester of **4**. The ^1^H NMR spectrum contained only one set of signals, showing that one of the possible hemiacetal diastereoisomers had significantly lower energy than the other but it was not possible to determine which from the spectroscopic data. The methyl protons (H-2′, δ 2.05) of the acetate showed a strong HMBC to the corresponding ester carbonyl (C-1′, δ 170). C-1′ also showed a cross-peak to H-3, confirming the location of the ester. We assign structure **5** (Fig. 1) to fatimanol I.

### Compound 6

Compound 6 was also closely related to **4**. *Pseudo*molecular ions were seen in HRESIMS at *m/z* 397.1248 [M + Na]^+^ (calc 397.1263) and *m/z* 375.1429 [M + H]^+^ (calc 375.1444), consistent with the formula C_20_H_22_O_7_. A ^13^C isotopomer peak was observed at *m/z* 398.1282 (calc 398.1297). The ^13^C NMR spectrum showed discrete signals for 2 × CH_3_, 4 × CH_2_, 7 × CH and 7 × C_q_. The ^1^H and ^13^C NMR spectra (Table S2, SI) (with 2D spectra) were very similar to those for **4**, with the addition of signals for OCH_3_ (δ_H_ 3.19, *δ*_C_ 51.0). These protons showed a strong HMBC to the ^13^C signal for C-6 (δ 150.8), identifying the methoxy group as being part of an acetal at this position. These data show that **6** (fatimanol J, Fig. 1) has the structure shown.

### Compound 7

HRESIMS contained a *pseudo*molecular ion at *m/z* 425.1459, corresponding to [M − H]^−^ (calc 425.1526) for the formula C_20_H_26_O_10_. The ^13^C NMR spectrum showed discrete resonances for 20 carbons: 1 × CH_3_, 6 × CH_2_, 6 × CH, 7 × C_q_. The IR contained absorbances for two carbonyls (1769 cm^−1^, 1737 cm^−1^) and OH (3433 cm^−1^). The NMR data (Table S2, SI) suggested a neoclerodane core. The upper part contained a hydroxyfuranone ring, as in fatimanol A^15^. This cyclic acetal was represented by H-14 (δ 7.17), which showed an HSQC cross-peak to C-14 (δ 146.6); the downfield shifts of these signals were due to the enone system. From H-14, 2-bond HMBC cross-peaks were seen to C-13 (δ 108.1) and to C-15 (δ 99.0) and 3-bond cross-peaks were evident to the carbonyl C-16 (δ 172.2) and (weakly owing to the adverse dihedral angle) to C-12 (δ 64.0). H-14 did not show a COSY cross-peak to the hemiacetal proton H-15 (δ 5.88), owing to the small coupling constant consequent to a dihedral angle *ca*. 90°. HSQC identified H-12 as part of a complex multiplet at δ *ca*. 4.6. A 2-bond HMBC from H-12 then led to C-11 (δ 41.3), from which HSQC showed the two signals for H-11 (δ 1.6, δ 2.25). From the downfield H-11 signal, HMBC identified the signals for C-9 (δ 50.2), C-10 (δ 43.9) and the lactone carbonyl C-20 (δ 174.6). This lactone was confirmed by HMBC cross-peaks from C-20 to H-19 (δ 4.58, δ 4.64) and the latter were linked on to C-4 (δ 85.6), C-5 (δ 49.7) and C-6 (δ 108.2). A 2-bond HMBC correlation from C-4 indicated H-3 (δ 3.83). The latter then gave an HMBC cross-peak to the methylene C-18 (δ 75.6) with H-18 (δ 3.97 d (*J* = 10.4 Hz), δ 4.35 d (*J* = 10.4 Hz)). The downfield H-19 also gave a 3-bond HMBC with C-10, completing the lower lactone ring. H-3 resonated as a dd (*J* = 11.6, 5.9 Hz), the larger of the two coupling constants indicating that this proton is axial. Completing the features of the lower part of the structure, H-18 (δ 4.35) showed a 3-bond HMBC with the hemiacetal carbon C-6. We assign the structure **7** (Fig. 1) to this compound, fatimanol K.

### Compound 8

HRESIMS showed a *pseudo*molecular ion at *m/z* 519.2212 [M + Na]^+^ (calc 519.2206) for the formula C_25_H_36_O_10_. The ^13^C NMR spectrum showed 25 discrete resonances: 4 × CH_3_, 8 × CH_2_, 6 × CH, and 7 × C_q_. Bands for OH (3615 cm^−1^) and three ester / lactone carbonyls (1744, 1734, 1695 cm^−1^) were seen in the IR. Combined interpretation of the NMR spectra (Table S3, SI) showed that **8** was a neoclerodane. The upper ring was a furan-2-one. The acetal proton H-15 resonated as a doublet at δ 5.84, with a small coupling constant (2 Hz) to H-14 (δ 7.02) confirmed by COSY. HSQC then identified C-15 (δ 104.6) and C-14 (δ 144.8). The latter was appropriate for the β-carbon of an enone. HMBC linked H-15 to C-13 (δ 138.9) and the lactone carbonyl C-16 (δ 173.3). H-14 and C-16 also showed a cross-peak in HMBC. The IR band at 1695 cm^−1^ was assigned to this α,β-unsaturated ester. The methoxy protons *(δ* 3.52) showed a HMBC cross-peak to C-15, demonstrating its location. HSQC identified the methoxy carbon signal at δ 57.3. HMBC from C-13 to both H-12 (δ 2.15, δ 2.28) and to both H-11 (δ 1.4. δ 2.25) confirmed the attachment of the methoxyfuranone ring at C-12. C-11 (δ 34.6) and C-12 (δ 19.3) were identified by HSQC. A further HMBC cross-peak from H-11 (δ 1.4) to the ^13^C signal at δ 66.5 (CH_2_) showed that the latter was due to C-20. HSQC confirmed the geminally coupled doublets (δ 3.97 and δ 4.03) (*J* = 12.0 Hz) as due to the two H-20. These were linked by HMBC to the carbonyl ^13^C signal at δ 172.6 and thence to the acetate protons at δ 2.06. Both H-20 signals had 3-bond HMBC correlations with C-10 (δ 47) and C-20 had HMBC correlation with the signal with H-8 (δ 1.7). H-10 (δ 1.7) and C-8 (δ 35.2 or 35.1) were then identified by HSQC. The C-Me group (H_3_C-17) resonated as at high field (δ_H_ 0.97, δ_C_ 16.5). HMBC cross-peaks from H_3_C-20 linked to both H-7 (δ 1.7, δ 1.8), from which C-7 (δ 30.8) was identified by HSQC. A weak 4-bond HMBC cross-peak was observed between H-17 and C-6 (δ 74.3), as in **2** and **4**. The chemical shift of this carbon and of H-6 (δ 3.78) suggested the presence of an oxygen. H-6 had 3-bond HMBC correlations with C-4 (δ 66.9) and with C-19 (δ 63.7). HSQC identified the geminally coupled H-19 protons (δ 4.43, δ 4.65). HMBC from these latter protons confirmed the lower acetate carbonyl *(δ* 172.7), linked on to the methyl proton signal at δ 2.06, co-incident with the other acetate signal. Weak 2-bond HMBC cross-peaks from C-4 identified the oxirane protons H-18 at δ 3.01 and δ 3.21, while a stronger 2-bond cross-peak showed H-3 (δ 4.00), with its C-3 (δ 66.5).

The relative stereochemical configurations were largely determined by use of NOESY. An MM2-minimised structure suggested that the decalin would have both rings in chair conformations. Strong NOESY cross-peaks showed that H-17 and H-20 were *cis* on the lower face. Similarly, H_2_-19 were shown to be on the lower face by strong NOESY cross-peaks to H-20, with these methylenes being diaxial on ring B. Further strong cross-peaks from both H-19 to H-3 confirmed the latter as axial down on ring A and a cross-peak to H-1 (δ 2.1) also suggested that this was on the lower face. On the upper face, the downfield H-18 signal (δ 3.21) gave a cross-peak to H-6 (δ 3.78), which allowed differentiation of the two oxirane proton signals. The relative configuration at C-15 could not be determined. These data show the structure of **8** (Fig. 1), fatimanol L.

### Compound 9

HRESIMS showed *pseudo*molecular ion peaks at *m/z* 535.2189 [M + Na]^+^ (calc 535.2155) and *m/z* 513.2350 [M + H]^+^ (calc 531.2336), corresponding to the formula C_25_H_36_O_11_. The ^13^C NMR spectrum complied, with 25 discrete signals: 4 × CH_3_, 7 × CH_2_, 7 × CH, 7 × C_q_. The IR showed OH (3546 cm^−1^) and three carbonyls (1764, 1752, 1708 cm^−1^). The NMR spectra (Table S3, SI) showed considerable similarity to those for **8**, except in the C-11 / C-12 region and the upper methoxyfuran. The structure of the *trans*-decalin and the lower appendages were identical to those of **8**. HMBC from C-11 (δ 38.0) identified H-12 (δ 4.56); the downfield chemical shift of this peak indicated a hydroxy group. HSQC identified C-12 (δ 63.8). H-12 was also linked by COSY to both H-11 (δ 1.75, δ 1.80). A strong 3-bond HMBC from H-12 showed the signal at δ 144.8 to be due to C-14, with HSQC identifying H-14 (δ 7.13). Appropriate HMBC and HSQC correlations then identified C-13 (δ 144.2), C-15 (δ 104.2), H-15 (δ 5.88) and C-16 (δ 171.7). The methoxy group protons resonated as two singlets (δ 3.54, δ 3.55), each integrating for 1.5 H, suggesting that **9** was a mixture of epimers at C-15. The corresponding methoxy ^13^C signal was linked by HMBC to H-15. NOESY also linked together the upper part of the structure. Strong cross-peaks were seen linking H-15 with the methoxy group and with H-14. Furthermore, H-14 was linked by NOESY with H-12. Addressing the relative configurations of **9** (except for the mixture of epimers at C-15), strong NOESY cross-peaks were seen from H-8 (δ 1.94) to H-6 (δ 3.78), to H-10 (δ 2.22) and to H-12, showing that all of these are on the upper face of the bicycle. NOESY also linked H-6 with one H-18 (δ 3.17), confirming that the latter is on the upper face and differentiating the two H-18 signals. On the lower face, one H-19 (δ 4.42) formed a NOESY cross-peak with H-3 (δ 4.00) and the other H-19 (δ 4.71) was close in space with H-20 (δ 3.95). The ^1^H signals for H-2 were differentiated by a NOESY cross-peak from H-10 to H-2_ax_ (δ 2.10). The configuration at C-12 could not be determined. The structure is thus **9** (Fig. 1), fatimanol M.

### Compound 10

HRESIMS showed a *pseudo*molecular ion at *m/z* 425.1452 [M − H]^−^ (calc 425.1448), corresponding to the formula C_20_H_26_O_10_. Smaller ions were seen at *m/z* 426.1485 [M − H]^−^ (calc 426.1481) and *m/z* 427.1520 [M − H]^−^ (calc 427.1515) for ^13^C_1_ and ^13^C_2_ isotopomers, respectively. In positive-ion mode, the HRESIMS contained the *pseudo*molecular ion at *m/z* 427.1596 [M + H]^+^ (calc 427.1604), in addition to an abundant ion at *m/z* 409 [M + H – H_2_O]^+^ showing an aliphatic alcohol. Twenty discrete ^13^C NMR peaks were evident: 1 × CH_3_, 7 × CH_2_, 5 × CH, 7 × C_q_. Three C_q_ were carbonyls, with two coincident ^13^C NMR signals at δ 174.5 and a singleton at δ 173.1, and IR bands at 1795, 1790, and 1689 cm^−1^. The higher-frequency C = O absorptions suggested that they were likely to be a cyclic anhydride. As with other examples, the NMR data (Table S3, SI) suggested the neoclerodane skeleton for **10**. However, the upper five-membered ring was unusual, in that it was a succinic anhydride. Carbonyl C-15 resonated at δ 174.5 and C-16 appeared at δ 173.1. The latter showed a 3-bond HMBC correlation with one H-14 (δ 2.04). COSY then identified the other H-14 (δ 1.42) and H-13 (δ 3.1), with HSQC then establishing C-14 (δ 33) and C-13 (δ 49.7). COSY linked H-13 to H-12 (δ 4.76) and HSQC identified C-12 (δ 65.8). A 2-bond HMBC from the latter led to assignment of one H-11 (δ 1.89 dd), which was shown by HSQC to be attached to the same carbon (C-11, δ 34.2) as the signal at δ_H_ 2.43 for the other H-11. An additional 2-bond HMBC led to identification of C-9 (δ 36.2). In the lower part, a 3-bond HMBC was observed from the methyl H-17 (δ 0.94) to C-7 (δ 41.2). A 3-bond HMBC from H-8 to the hemiacetal carbon (C-6, *δ* 108.1) confirmed its location. A further HMBC from C-6 to one H-18 (δ 4.34) showed the closure of the lower hemiacetal ring. An MM2-minimised model explained the absence of a HMBC cross-peak from the other H-18 to C-6, in that the dihedral angle is *ca*. 90°. H-18 (δ 4.34) also gave an HMBC with C-3 (δ 73.3), which carries an oxygen. HSQC then identified H-3 (δ 3.83) as a dd with *J* = 9.4, 4.6 Hz. The larger coupling constant implies a *trans*-diaxial coupling; thus H-3 is axial. HMBC correlation from H-3 identified C-2 (δ 32.8). A 2-bond HMBC linked H-3 with C-4. The (C-19)—O―(C-20) ester bridge was also confirmed by HMBC, in that both H-19 (δ 4.59 and δ 4.65) showed cross-peaks with C-5 (δ 85.6), C-6 (δ 50.2) and C-20 (δ 174.5). C-19 was identified by HSQC at δ 68.5, consistent with the ester. Thus the structure of **10** (fatimanol N) is as in Fig. 1.

### Compound 11

**Figure 2:**
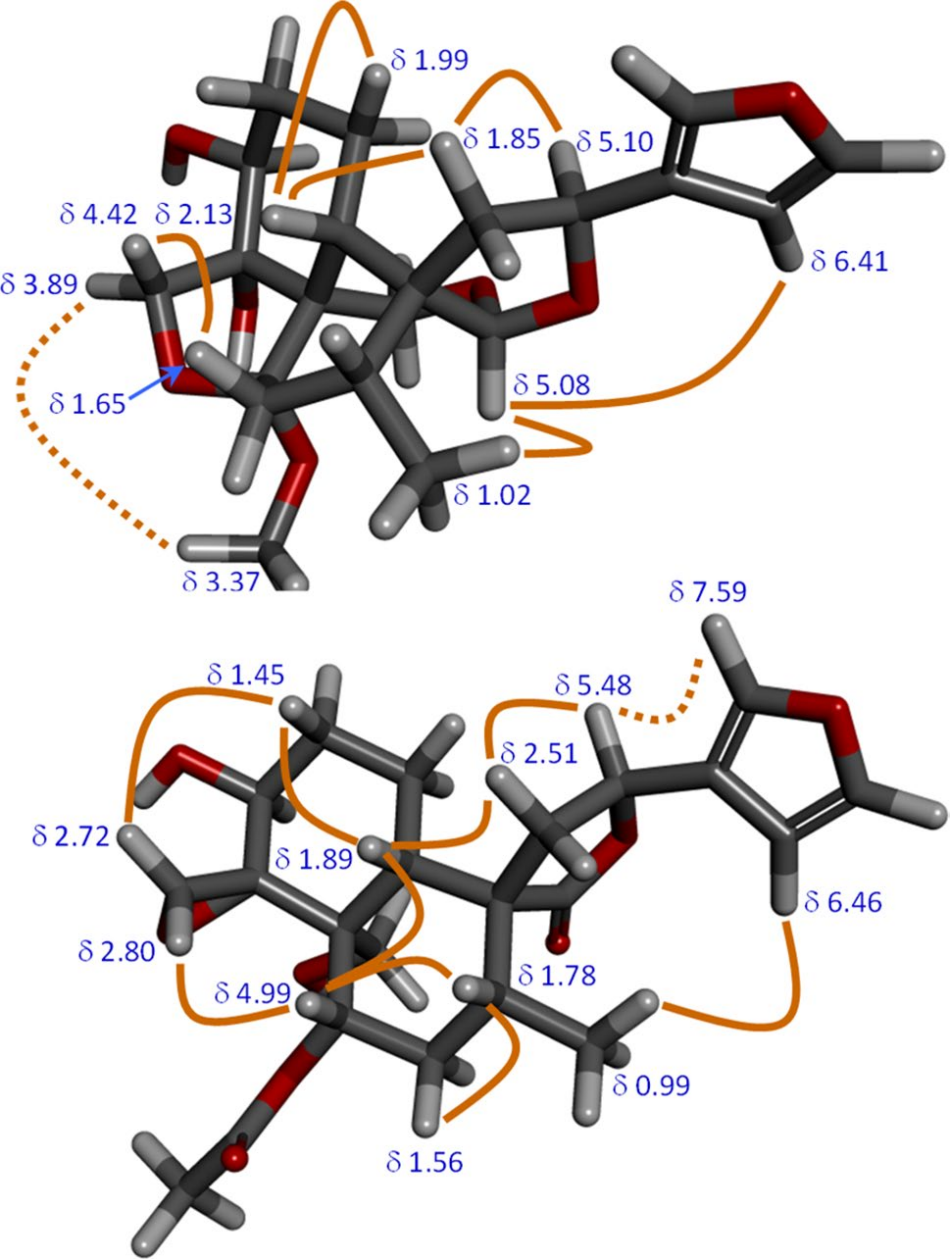
Upper: Key NOESY interactions confirming the relative stereochemical configurations of the rigid polycycle 11. Lower: Key NOESY interactions confirming the relative stereochemical configurations of 12. A NOESY cross-peak was also observed between H 19 (δ 3.90) and H-3. Both conformations were suggested by MM2 minimisation.

Instability under MS conditions precluded obtaining useful mass spectra. The ^13^C NMR showed discrete peaks for 21 carbons: 2 × CH_3_, 6 × CH_2_, 8 × CH, 5 × C_q_. The IR contained a band for OH (3414 cm^−1^) but no carbonyls. The NMR data (Table S3, SI) showed a neoclerodane structure. The 3-substituted aromatic furan was shown by the downfield resonances of the ring-H. H-14 resonated at δ 6.41, while H-15 and H-16 were co-incident at δ 7.43. HMBC correlated H-14 with C-13 (δ 126.7), C-15 (δ 143.6) and C-16 (δ 139.3). Similarly, H-15 correlated with C-16 and H-16 correlated with C-14 (δ 108.6) and with C-15. HMBC also linked C-13, C-13, and C-16 with H-12 (δ 5.10 m) and HSQC identified C-12 at δ 71.6. A COSY cross-peak from H-12 to the multiplet at δ 1.85 identified the latter as one H-11 and HSQC led to C-11 (δ 40.5) and thence to the other H-11 (δ 2.24). C-20 was an acetal carbon, as shown by its chemical shift (δ 101.2), and HSQC identified H-20 as the singlet at δ 5.08. H-20 gave HMBC cross-peaks to C-8 (δ 35.2) and C-9 (δ 44.6) and to C-12, demonstrating the ether linkage. HMBC from C-8 to both H-11 completed this ring. The C-Me group (C-17, H-17) was readily identified as the origin of the most upfield NMR signals (δ_C_ 16.3, δ_H_ 1.02). From here, a 2-bond HMBC identified H-8 (δ 1.85), confirmed by a COSY cross-peak from H-17. Three-bond HMBC interactions also identified both H-7 (δ 1.65 t, δ 2.36 dd). These multiplicities indicated that the former was axial and the latter was equatorial. C-7 (δ 36.1) was located by HSQC. Acetal C-6 (δ 110.9) was confirmed by 3-bond HMBC with both H-19 (δ 3.94, δ 4.15, geminally coupled). Both H-19 also gave HMBC cross-peaks to C-20, confirming the ether bridge between C-19 (δ 58.5) and C-20. The closure of the lower tetrahydrofuran was demonstrated by HMBC from C-6 to both H-18 (δ 3.89, δ 4.42). Moving clockwise around the decalin, HMBC from C-18 (δ 75.1) identified H-3 (δ 3.89), the large ^3^*J* of which indicated a *trans*-diaxial relationship with one H-2 (δ 1.41). Further HMBC, HSQC, and COSY analysis established the assignments of H-1 (δ 1.95 and δ 1.99), C-1 (δ 23.2), H-2 (δ 1.41 and δ 2.15), C-2 (δ 30.3) and C-3 (δ 73.2). Thus **11** has the novel polycyclic structure shown in Fig. 1, with many fused rings and bridges. Fortunately, these fusions and bridges make the structure fairly rigid and it was straightforward to assign the relative stereochemical configurations by use of coupling constants (Karplus relationship) and NOESY. Figure 2 shows the key NOESY interactions used in this assignment. Particularly useful was the NOE interaction between the methoxy protons and H-18_*exo*_ (δ 3.89), which confirms that the methoxy is on the lower face of the *trans*-decalin. The name fatimanol O is assigned to the novel compound **11** (Fig. 1).

### Compound 12

HRESIMS gave *pseudo*molecular ions at *m/z* 443.1706 [M + Na]^+^ (calc 443.1682) and *m/z* 421.1854 [M + H]^+^ (calc 421.1862), corresponding to the formula C_22_H_28_O_8_. The ^13^C NMR spectrum showed 22 discrete signals: 2 × CH_3_, 6 × CH_2_, 8 × CH, 6 × C_q_. The NMR data (Table S4, SI) showed that **12** was a neoclerodane. In the upper furan, C-13 resonated at δ 126.7 and gave HMBC cross-peaks to H-14 (δ 6.46), H-15 (δ 7.53) and H-16 (δ 7.59). The corresponding ^13^C resonated at δ 109.2 (C-14), δ 145.6 (C-15) and δ 141.4 (C-16). C-15 and C-16 were differentiated by HMBC between the latter and H-12 (δ 5.48). HSQC identified C-12 (δ 73.5). Three-bond HMBC from C-13 also identified both H-11 (δ 2.38, δ 2.51), from which C-11 was shown to be at δ 43.6. The downfield H-11 signal also showed HMBC to C-9 (δ 52.0), C-10 (δ 51.8) and carbonyl C-20 (δ 178.9). The methyl group gave the most upfield signals (C-17 δ 16.9, H-17 δ 0.99). Strong HMBC cross-peaks identified C-8 (δ 38.65) and C-7 (δ 33.5), whereas a weak cross-peak suggested the peak at δ 74.7 as C-6. H-8 resonated at δ 1.78 and H_2_-7 at δ 1.56, δ 2.22. The chemical shifts of C-6 and of H-6 (δ 4.99) were consistent with an ester oxygen and HMBC from H-6 to the carbonyl signal at δ 171.6 confirmed that this appendage was an acetoxy group (AcO-) (*Me*CO_2_ δ_C_ 21.3 δ_H_ 2.04). H-6 also gave a strong HMBC to C-5 (δ 48.0) and to C-19 (δ 61.4). The diastereotopic H-19 protons gave doublet signals at δ 3.90 and δ 4.62. C-19 gave a strong 3-bond HMBC to H-10 (δ 1.89). The upfield H-19 gave a 2-bond HMBC with quaternary C-4 (δ 52.8). The relatively upfield chemical shift of C-4 was consistent with the oxirane and C-18 (a methylene) was also identified by its chemical shift at δ 42.5. HSQC then linked it to the doublets for H-18 (δ 2.72, δ 2.80). C-18 gave a moderate HMBC to H-3 (δ 4.28), from which COSY and HSQC identified the signals for C-3 (δ 68.7), C-2 (δ 34.1), H-2 (δ 1.45, δ 2.22), C-1 (δ 23.1) and H-1 (δ 1.78, δ 2.12). Completing ring A, the upfield H-1 gave a strong COSY with H-10. The structure of **12** is less rigid than **11** but it was still possible to assign its relative configurations by NOESY (Fig. 2). These data confirm the structure of **12**, fatimanol P (Fig. 1).

### Compound 13

HRESIMS showed a *pseudo*molecular ion at *m/z* 405.1880 [M + Na]^+^ (calc 405.1889), corresponding to the formula C_20_H_30_O_7_. Other ions were observed at *m/z* 787.3875 [2 M + Na]^+^ (calc 787.3881). Fragment ions were observed at *m/z* 729.3844 [2 M + Na—C_2_H_2_O_2_]^+^ (calc 729.3826), *m/z* 711 [2 M + Na—C_2_H_2_O_2_—H_2_O]^+^, *m/z* 693 [2 M + Na—C_2_H_2_O_2_—2 × H_2_O]^+^, *m/z* 347.1850 [M + Na – C_2_H_2_O_2_]^+^ (calc 347.1834), *m/z* 329.1744 [M + Na—C_2_H_2_O_2_—H_2_O]^+^ (calc 329.1729), and *m/z* 311.1639 [M + Na – C_2_H_2_O_2_—2 × H_2_O]^+^ (calc 311.1623), indicating the presence of at least two hydroxy groups. In the negative-ion HRESIMS, peaks were seen at *m/z* 427.1970 [M + formate]^−^ (calc 427.1968), *m/z* 417.1689 [M + ^35^Cl]^−^ (calc 417.1680), and *m/z* 381.1920 [M – H]^−^ (calc 381.1913). The ^13^C NMR contained signals for 20 carbons: 1 × CH_3_, 7 × CH_2_, 8 × CH, and 4 × C_q_, although two signals were co-incident at δ 143.5.

The NMR data (Table S4, SI) suggested that **13** had a structure broadly similar to those of the neoclerodanes but with important differences. The upper ring was a furan, with ^1^H NMR signals at δ 6.48 (H-14), δ 7.56 (H-15) and δ 7.50 (H-16). The signals for H-15 and H-16 were differentiated through COSY from H-15 to H-14 and by HMBC from H-16 to C-12 (δ 62.1). HSQC then linked these to the ^13^C peaks at δ 109.7 (C-14) and δ 143.5 (C-15, C-16). HMBC from H-14, H-15, and H-16 then identified the signal at δ_C_ 132.4 as being due to C-13. HMBC from C-13, C-14 and C-16 to the ^1^H signal at δ 4.68, along with HSQC from C-12, identified this multiplet signal as H-12. This benzylic proton gave COSY cross-peaks to 12-OH (δ 5.02, d) and both 11-H (δ 1.60, δ 2.01, both dd). HSQC from the latter then gave 11-C (δ 41.7). Linkage of this upper side-chain to the core bicycle was demonstrated by a HMBC correlation from H-12 to C-9 (δ 40.4, C_q_) and by HMBC from both H-11 to C-8 (δ 35.6) and to C-10 (δ 51.5). The 3-bond HMBC cross-peak between H-11 (δ 1.60) and C-8 was weak, as to the dihedral angle (H-11)–(C-11)–(C-9)–(C-8) is close to 90°. H-8 (δ 1.47) also gave a HMBC with C-11. The other side-chain at C-9 was a hydroxymethyl (HOCH_2_-) unit, with C-20 (δ 53.3) giving weak HMBC with both H-11. The geminal protons H-20 (δ 3.21, δ 3.32) gave strong COSY interactions with each other and with HO-20 (δ 4.46). The methyl group was at C-8, as shown by COSY from H-8 to the doublet at δ 0.71 (H-17), from which HSQC identified C-17 (δ 16.3). Moving clockwise around the B-ring, a strong 2-bond HMBC from C-8 identified H_ax_-7 at δ 1.21 (q, *J* = 11 Hz). HSQC revealed C-7 (δ 37.9) and thence H_eq_-7 (δ 1.47). Both H-7 gave strong 2-bond HMBC correlations with C-6 (δ 69.4), from which HSQC showed H-6 at δ 3.36. The COSY cross-peak from H_ax_-7 to H-6 was strong, whereas that from H_eq_-7 to H-6 was much weaker, suggesting that the (H_eq_-7)–(C-7)–(C-6)–(H-6) dihedral angle was *ca*. 90° and thus that H-6 was axial. COSY linked H-6 to HO-6 (δ 4.46). Three-bond HMBC from both H-7 located quaternary C-5 (δ 59.8). Ring B was completed by observation of a 2-bond HMBC from C-5 to H-10 (δ 2.07).

A 2-bond HMBC from H-10 identified C-1 (δ 24.6), from which HSQC showed that the H-1 signals were at δ 1.87 and δ 1.98. A strong 3-bond HMBC linked C-10 with one H-2 signal (δ 1.87), whereas the cross-peak to the other H-2 (δ 1.27) was weaker. A 3-bond HMBC was also seen linking H-1 (δ 1.27) to C-5; the 3-bond path between these two nuclei cannot pass through C-1 and C-10, thus ring A must be five-membered. Quaternary C-3 (δ 57.7) was identified through a 2-bond HMBC with H-2 (δ 1.27) and a 3-bond correlation with H-10, completing the cyclopentane. Three-bond HMBC from both H-2 showed the methylene C-4 (δ 62.5) as being attached at C-3 and the H-4 protons were observed as dd at δ 3.27 and δ 3.66. These H-4 signals were linked by COSY to each other and to HO-4 (δ 4.46). A 2-bond HMBC between H-4 (δ 3.27) and C-3 confirmed the attachment of the HOCH_2_-. 2-bond HMBC from C-3 and a 3-bond cross-peak from H-2 (δ 1.27) revealed the other substituent at C-3 by identifying C-18 (δ 102.0). The hemiacetal was confirmed by COSY from H-18 (δ 4.76) to HO-18 (δ 6.26). HMBC from C-5, C-6 and C-10 to the doublet signals at δ 3.80 and δ 3.87 identified both H-19. C-19 (δ 67.3) was shown to be a CH_2_ by HSQC and 135DEPT. The chemical shifts of C-19 and both H-19 suggested the attachment of an oxygen but this was not an OH (no COSY cross-peak). However, HMBC linked C-19 with H-18 and C-18 with both H-19. The only structure consistent with these connectivities is the cyclic acetal shown. It was not possible to obtain a good NOESY spectrum, so the relative configuration shown in Fig. 1 is speculative, except where suggested by ^3^*J*_H-H_ coupling constants. The NMR spectra contained a second (smaller) set of peaks, which we ascribe to the presence of a minor diastereoisomer in slow equilibrium, probably the epimer at C-18. Thus **13** (fatimanol Q) has the structure in Fig. 1.

### Compound 14

HRESIMS showed a *pseudo*molecular ion *m/z* 447.1985 [M + Na]^+^ (calc 447.1995), consistent with the formula C_22_H_32_O_8_. Other ions were at *m/z* 871.4083 [2 M + Na]^+^ (calc 871.4091) and *m/z* 425.2167 [M + H]^+^ (calc 425.2175) in positive-ion mode and 469.2075 [M + formate]^−^ (calc 469.2074), and 459.1795 [M + ^35^Cl]^−^ (calc 459.1786) in negative-ion mode. The ^13^C NMR spectrum (in (CD_3_)_2_SO) contained discrete signals for 22 carbon atoms: 2 × CH_3_, 7 × CH_2_, 8 × CH, 5 × C_q_.

Analysis of the NMR data ((CD_3_)_2_SO, Table S4, SI) showed that **14** was a conventional neoclerodane. The upper ring was a furan, with ^1^H signals for H-14 (δ 6.42), H-15 (δ 7.56) and H-16 (δ 7.49) and ^13^C signals for C-13 (δ 132.8), C-14 (δ 109.6), C-15 (δ 143.6) and C-16 (δ 138.6) all duly linked by HSQC and HMBC. Strong 3-bond HMBC cross-peaks were evident from H-12 (δ 4.59) to C-14 and C-16. HSQC linked this proton to C-12 (δ 61.7), which also gave HMBC to H-14 and H-16. A 2-bond HMBC from H-12 identified C-11 (δ 39.7), from which both H-11 (δ 1.67, δ 1.86) were identified by HSQC. HO-12 (δ 4.90) was located through a 3-bond HMBC from C-11. A 3-bond HMBC from H-12 identified C-9 (δ 43.3), while a similar correlation from H-11 (δ 1.67) confirmed C-10 (δ 46.8) and thence H-10 (δ 1.96). From here, C-20 (δ 63.0) was located by HMBC to H-10; the H-20 protons were approximately co-incident at δ 3.28, with HMBC cross-peaks to C-9 (weak, 2-bond), C-10, and C-11. Two-bond HMBC linked C-10 to both H-1 (δ 1.75, δ 2.00), from which C-1 (δ 21.5) was identified by HSQC. The upfield H-1 signal gave a weak 2-bond HMBC with C-2 (δ 34.5), whereas the downfield signal correlated strongly with C-3 (δ 65.0). The signal at δ 1.17 was a dq (*J* = 4, 11 Hz), indicating that this was due to H-2_ax_, while H-2_ eq_ resonated as a narrow multiplet at δ 1.91. Thus the coupling to H-3 (δ 3.80) shows that this proton is axial and HO-3 is equatorial . Furthermore, both H-3 and H-10 are axial, showing that ring A is in the chair conformation. A 2-bond HMBC from H-3 identified C-4 (δ 70.0) and a 3-bond correlation revealed C-18 (δ 43.0). H_2_-18 resonated as doublets at δ 2.83 and δ 3.06; the chemical shifts suggested the *spiro*-oxirane ring. Strong HMBC from these protons were observed to C-4 and C-5 (δ 45.47). An AcOCH_2_- group was present at C-5, as demonstrated by HMBC from both H-19 (δ 4.40, δ 4.57) to C-4 and C-5. These protons also correlated with the ester carbonyl Me*C*O_2_-19 (δ 170.8), with the adjacent methyl group (*Me*CO_2_-19) resonating at δ_H_ 2.01/δ_C_ 21.5. Three-bond HMBC cross-peaks were also seen from both H-19 to C-6 (δ 73.3) and HSQC then located the H-6 signal at δ 3.62 (brd, *J* = *ca*. 11 Hz). The H-6 signal was better resolved when the ^1^H NMR spectrum was obtained on a solution in CDCl_3_, which showed it as δ 3.70 (dd, *J* = 10.0, 6.0 Hz). The larger axial-axial coupling shows that H-6 is axial on the *trans*-decalin. Both H-7 (δ 1.39, δ 1.52) were located both by HSQC with C-7 and by HMBC with C-6, whence C-7 (δ 34.7) was revealed by HSQC. H-7 also formed HMBC correlations with the methyl C-17 (δ 17.0), from which H-17 was identified at δ 0.82. Completing ring B, H-17 gave an HMBC cross-peak with C-9. These spectroscopic interpretations were aided, in part, by ^1^H, COSY and HSQC spectra of a solution in CDCl_3_, which were better resolved, although paucity of sample precluded identification of the quaternary carbons (Table S4). The coupling constants confirmed of the *trans*-decalin structure and the relative configurations of most of the substituents, although the relative configuration at C-12 could not be established. We assign the structure shown in Fig. 1 to 14, fatimanol R.

### Compound 15

The HRESIMS contained a *pseudo*molecular ion at *m/z* 379.1748 [M + H]^+^ (calc 379.1757), which showed the formula C_20_H_26_O_7_. Other ions were observed at *m/z* 779.3248 [2 M + Na]^+^ (calc 779.3255), *m/z* 401.1567 [M + Na]^+^ (calc 401.1576), *m/z* 361.1642 [M + H—H_2_O]^+^ (calc 361.1651), 343.1537 [M + H—2 × H_2_O]^+^ (calc 343.1546), and *m/z* 325.1341 [M + H—3 × H_2_O]^+^ (calc 325.1440). The latter three ions indicated the presence of three hydroxy groups. Negative ions were present at *m/z* 423.1656 [M + formate]^−^ (calc 423.1655), and *m/z* 377.1605 [M − H]^−^ (calc 377.1600). The ^13^C NMR spectrum (in (CD_3_)_2_SO) contained individual signals for 20 carbon atoms: 1 × CH_3_, 6 × CH_2_, 8 × CH, 5 × C_q_. The NMR data (Table S5, SI) for **15** indicated a conventional neoclerodane. The upper furan showed ^1^H signals for H-14 (δ 6.49), H-15 (δ 7.74) and H-16 (δ 7.78). HSQC linked these to C-14 (δ 109.1), C-15 (δ 145.0) and C-16 (δ 141.1), respectively. The C-13 ^13^C NMR signal was identified at δ 125.6 by HMBC. Moving south, 3-bond HMBC cross-peaks from C-14 and C-16 identified H-12 as a triplet at δ 5.43. The HMBC from C-16 to H-12 and the NOESY interaction between H-16 and H-12 distinguished H-16 from H15. The chemical shift of H-12 suggested that it carried a lactone oxygen, confirmed by HMBC from H-12 to C-20 at δ 177.0. HSQC linked H-12 to C-12 (δ 71.4). A 2-bond HMBC linked H-12 to C-11 (δ 41.2), with HSQC from this signal H_2_-11 (δ 2.25, δ 2.45). Both H-11 gave HMBC correlations to the lactone carbonyl C-20, further confirming the ring. Both H-11 also gave HMBC cross-peaks to the quaternary carbon (δ 51.2), which was shown to be C-9 where the *spiro*-lactone joins the decalin. Working clockwise around the lower decalin, C-19 gave a strong HMBC to the methyl H-17 protons *(δ* 0.91), from which C-17 (δ 16.9) was identified. A 2-bond HMBC from H-17 located C-8 (δ 37.3) and a 3-bond correlation located the methylene C-7 (δ 34.9), HSQC then showed H-8 (δ 1.62) and both H-7 (δ 1.48 (H-7_ eq_), δ 1.96 (H-7_ax_)). C-7 showed a 2-bond HMBC with H-6 (δ 3.67); this chemical shift indicates an attached alcohol. C-6 (δ 72.5) was shown by HMBC with H-7_ eq_ and HSQC with H-6. The signal for 6-OH was broad but gave HMBC correlation with C-6. The signal at δ 46.4 was due to C-5. HMBC interactions then tied C-5 to the attached CH_2_OH group (H-19 δ 3.69, δ 4.36; HO-19 δ 4.07) and HSQC identified C-19 (δ 59.3). Both H-19 gave strong 3-bond HMBC cross-peaks to quaternary C-4 (δ 68.8), which was shown to be part of a *spiro*-oxirane. In the oxirane, H-18_left_ resonated as a doublet at δ 2.69 and H-18_right_ as a doublet at δ 2.89. These diastereotopic protons were distinguished by NOESY correlations from H-18_left_ to HO-3 (δ 4.66) and one H-2 (δ 1.30) and from H-18_right_ to H-19 (δ 3.69), H-6 and HO-6. Three-bond HMBC from both H-18 identified C-3 (δ 64.3), from which H-3 *(δ* 4.19) was shown by HSQC. HMBC from C-3 identified its HO-3 as a doublet at δ 4.66. The ring was completed by appropriate HMBC and HSQC cross-peaks identifying C-2 (δ 34.1), H-2 (δ 1.30, δ 2.05), C-1 (δ 22.0) and H-1 (δ 1.59, δ 1.96). Finally, there was a strong HMBC linking H-2 (δ 1.30) with C-10 (δ 51.2), closing the ring. H-10 resonated at δ 1.67.

The relative configurations were largely demonstrated by NOESY, with some consideration of ^1^H *J* values. Firstly, a strong NOESY interaction was seen between H-16 and H-17 and a weaker one between H-14 and H-17. This demonstrates that the furan and the methyl group are on the same face of the lactone and that the configuration at C-12 is *S*. The signal for H-7 (δ 1.48) is a broad doublet, thus this proton is equatorial up as the only large coupling constant would be ^2^*J*_gem_ to H-7_ax_ (δ 1.96). The methyl (C-17, H-17) is equatorial down, as for the vast majority of neoclerodanes. H-7_ax_ makes a strong NOESY interaction with H-19 (δ 4.36), consistent with C-19 being axial down. HO-6 is located on the lower face of the decalin, as H-6 experiences a strong NOESY correlation with H-7_ eq_ on the upper face. Running the spectrum in DMSO also allowed a NOESY interaction between HO-6 and H-19 (δ 4.36), confirming the orientation of HO-6. The configuration at C-4 of the *spiro*-oxirane was determined. H-1 (δ 1.59) is axial and down, as it has three large coupling constants (*trans*-diaxial to H-10 and H-2 (δ 1.30) and geminal to H-1_ eq_ (δ 1.96)). Therefore, H-2 (δ 1.30) is axial up. H-18_left_ (δ 2.69) makes strong NOESY contacts with H-2_ax_ and with HO-4 (upper face), which shows that the CH_2_ of the oxirane is on the upper face, as in **2**, **8**, **9**, and **12**. Finally, H-10 is on the upper face, as it shows a *trans*-diaxial coupling to H-1_ax_. Thus the bicycle is a *trans*-decalin. These spectroscopic assignments were aided partly by ^1^H, COSY, and HSQC spectra obtained of a solution in CDCl_3_, which were better resolved, although shortage of sample precluded identification of the quaternary carbons (Table S5). Minor differences in chemical shift were seen for H-2_ eq_, H-3, H-14, H-15, H-18, and H-19, probably reflecting minor changes in hydrogen-bonding and consequent minor changes in conformation. The COSY spectrum confirmed the H–H connectivities within the molecule. We assign the structure shown in Fig. 1 to 15, fatimanol S.

### Compound 16

The HRESIMS contained a *pseudo*molecular ion at *m/z* 379.1748 [M + H]^+^ (calc 379.1757), consistent with the formula C_20_H_26_O_7_. Ions were also seen at *m/z* 779.3250 [2 M + Na]^+^ (calc 779.3255), *m/z* 401.1568 [M + Na]^+^ (calc 401.1576), 361.1643 [M + H − H_2_O]^+^ (calc 361.1651), and *m/z* 325.1432 [M + H − 3 × H_2_O]^+^ (calc 325.1440), indicating three OH groups. The ^13^C NMR spectrum (in (CD_3_)_2_SO) contained signals for 20 carbon atoms: 1 × CH_3_, 6 × CH_2_, 8 × CH, and 5 × C_q_. Therefore, **16** is a closely structurally related isomer of **15**. Analysis of the COSY, HSQC, HMBC and some NOESY connectivities showed an identical network to **15**, strongly suggesting that they were stereoisomers. The NMR signals (in (CD_3_)_2_SO, Table S5, SI) for the lower part of the structure were very similar: position-2 (δ_H_ 1.20, δ_H_ 2.00, δ_C_ 34.1), position-3 (δ_H_ 4.16, δ_C_ 64.5), position-4 (δ_C_ 68.8), position-5 (δ_C_ 46.2) and position-6 (δ_H_ 3.65, δ_C_ 72.5). Slightly more significant differences in chemical shift were observed for the upper and right parts of the decalin, at position-1 (δ_H_ 1.43, δ_H_ 1.68, δ_C_ 21.5), position-7 (δ_H_ 1.42, δ_H_ 1.74, δ_C_ 35.6), position-8 (δ_H_ 1.74, δ_C_ 40.0), and position-10 (δ_H_ 1.50, δ_C_ 49.2). The signal for C-9 (δ 51.5) was identical in **16** and **15**. In the upper *spiro*-lactone, the differences were again observed for position-11 (δ_H_ 2.33, δ_H_ 2.41, δ_C_ 42.6) but less significant for position-12 (δ_H_ 5.42, δ_C_ 71.3) and the lactone carbonyl C-20 (δ 176.8). Small differences were also seen for the furan: position-13 (δ_C_ 125.8), position-14 (δ_H_ 6.51, δ_C_ 109.3), position-15 (δ_H_ 7.71, δ_C_ 145.0) and position-16 (*δ*_H_ 7.77, *δ*_C_ 140.7). The larger differences were in the upper part of the decalin and in the lactone, which suggested that the stereochemical difference between **16** and **15** was at C-9 or at C-12. A detailed study of NOESY data in that area was undertaken. Firstly, the equatorial methyl H-17 gave a strong NOESY cross-peak to H-12, thus these were on the same face of the γ-lactone. Secondly, H-12 gave a strong NOESY correlation with one H-11 (δ 2.41) but only weakly with the other H-11 (δ 2.33). Since the furan protons H-14 and H-16 both formed strong NOESY cross-peaks with H-11 (δ 2.33), the furan and this upfield H-11 must be on the same face of the γ-lactone and this face must be opposite to that carrying H-12. These data are consistent with **16** having the opposite configuration at C-12 from **15**. Other NOESY interactions, COSY cross-peaks and ^1^H-^1^H coupling constants in the decalin were consistent with *trans*-configuration. The spectroscopic assignments were aided, in part, by ^1^H, COSY, and HSQC spectra of a solution in CDCl_3_, which were better resolved, although shortage of sample precluded identification of the quaternary carbons and NOESY data could not be obtained. We assign the structure shown in Fig. 1 to 16, fatimanol T.

### Compound 17

The HRESIMS contained a *pseudo*molecular ion at *m/z* 503.1886 [M + Na]^+^ (calc 503.1893), confirming the formula C_24_H_32_O_10_. Other positive ions at *m/z* 983.3881 [2 M + Na]^+^ (calc 983.3889), *m/z* 481 [M + H]^+^, *m/z* 463.1961 [M + H − H_2_O]^+^ (calc 463.1968), *m/z* 445.1856 [M + H − 2 × H_2_O]^+^ (calc 445.1862), *m/z* 421 [M + H − AcOH]^+^, *m/z* 403.1750 [M + H − H_2_O − AcOH]^+^ (calc 403.1757), and *m/z* 361.1644 [M + H − 2 × AcOH]^+^ (calc 361.1651) confirmed the formula and indicated at least two hydroxy groups and at least two acetate esters. Negative-mode ions were present at *m/z* 525.1974 [M + formate]^−^ (calc 525.1972), *m/z* 515.1693 [M + ^35^Cl]^−^ (calc 515.1684), and *m/z* 479.1923 [M − H]^−^ (calc 479.1917). The ^13^C NMR spectrum (in (CD_3_)_2_SO) contained discrete signals for 24 carbon atoms: 3 × CH_3_, 6 × CH_2_, 8 × CH, 7 × C_q_. Compound **17** was a conventional neoclerodane. As for **15**, the upper part of the structure was a furan linked to a γ-lactone. H-14, H-15, and H-16 resonated at δ 6.51, δ 7.71 and δ 7.79, respectively, with the carbon signals at δ 109.1 (C-14), δ 144.9 (C-15) and δ 141.2 (C-16) (Table S6, SI). C-13 (δ 125.7) was identified through HMBC interactions with H-15 and H-16. HMBC from H-16 also revealed C-12 (δ 74.1), from which H-12 (δ 5.45) was shown by HSQC. COSY then linked H-12 to both H-11 (δ 2.32 and δ 2.45) and thence by HSQC to C-11 (δ 43.1). The chemical shift of H-12 confirmed the lactone and 3-bond HMBC from both H-12 identified the lactone carbonyl C-20 at δ 177.1. The lactone was tied to the decalin through HMBC from the upfield H-11 to C-8 (δ 37.7). H-8 resonated at δ 1.70 and this signal correlated in HMBC with the methyl group (H-17 δ 0.96, C-17 δ 16.8). Strong HMBC cross-peaks linked C-17 to both H-7 (δ 1.54, δ 1.87) and thence by HSQC to C-7 (δ 36.1). HMBC from H-8 and both H-7 to the signal at δ 72.8 identified the latter as C-6. H-6 (δ 4.20) gave HMBC correlations to C-5 (δ 48.2) and C-4 (δ 76.9). The downfield chemical shift of C-4 indicated an oxygen but contraindicated a *spiro*-oxirane. H_2_-19 of the pendant methylene resonated as a pair of geminally coupled doublets at δ 4.77 and δ 4.86, which were linked by HMBC to C-5 and C-4. The chemical shifts of H_2_-19 suggested an AcO- group and this was confirmed by HMBC to 19-Me*C*O_2_, which linked onwards to the acetate methyl (δ_H_ 2.00, δ_C_ 169.9). H-18 / C-18 is a pendant HOCH_2_- group, with 2-bond HMBC from both H-18 (δ 3.73 and δ 4.04) to C-4 and 3-bond cross-peaks to C-5. Both H_2_-18 resonated as dd, with ^2^*J* = 10 Hz and smaller couplings to HO-18 (δ 5.06). This spin-set was confirmed by COSY. A 3-bond HMBC identified C-3 (δ 71.4). H-3 (δ 5.45) had a HMBC cross-peak with a carbonyl at *δ* 170.4, demonstrating that the oxygen at C-3 was acetylated. The methylenes at position-2 and position-1 were also identified by application of HMBC, (C-2 δ 36, H-2 δ 1.80, δ 2.45; C-1 δ 22.6, H-1 δ 1.95, δ 2.0). The closure of the decalin system was confirmed by a weak HMBC from C-2 to H-10 (δ 1.80) and a strong peak from C-4 to H-10. The above spectroscopic assignments were aided by ^1^H, COSY, HSQC, and HMBC spectra of a solution in CDCl_3_, which were better resolved, although shortage of sample precluded identification some quaternary carbons. The relative configurations corresponded to those of most of the neoclerodanes, particularly **15**, as demonstrated largely by ^1^H NMR coupling constants. The structure shown in Fig. 1 was assigned to **17**, fatimanol U.

### Compound 18

The HRESIMS contained ions at *m/z* 803.3255 [2 M + Na]^+^ (calc 803.3255) and *m/z* 413.1568 [M + Na]^+^ (calc 413.1576) confirming the formula C_21_H_26_O_7_. An ion was also seen at *m/z* 371 [M + H]^+^. The ^13^C NMR spectrum (in (CD_3_)_2_SO) contained discrete signals for twenty-one carbon atoms: 3 × CH_3_, 4 × CH_2_, 8 × CH, 6 × C_q_. Combined analysis of the NMR data (Table S6, SI) allowed assignment of all the signals and confirmed that the overall structure was similar to a conventional neoclerodane. However, C-6 was quaternary but not a carbonyl (*cf*. **2**). Two methoxy groups were also evident. The upper part of the structure was a furan, with the usual chemical shifts (position-13: δ_C_ 125.4; position-14: δ_H_ 6.52, δ_C_ 109.2; position-15: δ_H_ 7.72, δ_C_ 145.0; position-16: δ_H_ 7.82, δ_C_ 141.2). HMBC cross-peaks from C-13 linked this to H-12 (δ 5.48) and to one H-11 (δ 2.42). The other H-11 (δ 2.57) was identified by COSY cross-peaks to H-12 and to its geminal partner H-11. HSQC then located C-12 (δ 71.6) and C-11 (δ 40). The carbonyl (C-20) of the γ-lactone resonated at δ 176.6 and showed an HMBC cross-peak to the downfield H-11. It also gave an HMBC with H-10 (δ 2.45) and both H-11 gave HMBC correlations with C-9 *(δ* 53.0), confirming the attachment of the *spiro*-lactone. In the lower bicycle, H-10 also correlated in HMBC with C-8 (δ 35.8), from which H-8 (δ 2.01) and the methyl doublet H-17 (δ 0.92) were identified. COSY from H-8 led to one H-7 (δ 1.87) and thence to its geminal partner H-7 (δ 2.27). HSQC identified C-7 (δ 39.6). Two-bond HMBC linked both H-7 to C-6 (δ 109.16). This chemical shift implied that C-6 was an acetal or hemiacetal carbon, confirmed by a 3-bond HMBC with the upfield methoxy group MeO-6 (δ 2.97). Both H-7 also showed HMBC correlations to C-5 (δ 137.4), an alkene carbon. Thus C-19 is missing from the usual neoclerodane structure. H-10 is present, thus the other alkene carbon is C-4 (δ 139.5). HMBC from C-4 located HO-3 (δ 5.06), from which H-3 was shown (COSY) to be at δ 4.06 and thence C-3 was identified at δ 51.5 by HSQC. COSY joined H-3 to both H-2 (δ 1.39, δ 2.01) and HMBC from HO-4 identified C-2 (δ 33.0). CH_2_-1 resonated at δ_H_ 1.25, δ_H_ 2.27, and δ_C_ 23.7. In the bottom dihydrofuran, HMBC from C-3 and C-5 identified H-18 (δ 6.02) and HSQC confirmed C-18 (δ 107.0), where the chemical shifts were consistent with an acetal. One arm of this acetal was the oxygen linking through to C-6 and the other was a methoxy (MeO-18) (δ_H_ 3.15, δ_C_ 51.5), as shown by HMBC to H-18 and C-18. Turning to the configuration of **18**, the ^1^H chemical shift of MeO-6 was unusually low *(δ* 2.97). Examination of a molecular model showed that this methyl group, if on the lower face, would be held in the anisotropic shielding zone of the C-20 carbonyl; thus it is likely to be located on the lower face. The configuration at the other acetal C-18 could not be determined, although it was clear that only one diastereoisomer was present. A ^1^H NMR spectrum of **18** was also obtained in CDCl_3_, which was consistent with the structure determined above. The novel structure shown in Fig. 1 was assigned to **18**, fatimanol V.

### Compound 19

Instability under MS conditions precluded obtaining useful spectra. The ^13^C NMR spectrum (in (CD_3_)_2_SO) (Table S7, SI) contained twenty-two discrete ^13^C signals: 3 × CH_3_, 8 × CH_2_, 5 × CH, 6 × C_q_. The combined spectra showed it to be a modified neoclerodane. The upper ring was a furanone, with the H-16 protons resonating accidentally equivalently as a singlet at δ 4.86, with C-16 at δ 73.53. These chemical shifts suggested a γ-lactone. Three-bond HMBC cross-peaks from H-16 identified lactone carbonyl C-15 (δ 173.4) and C-14 (δ 114.0), with H-15 (δ 5.96) identified by HSQC. A 2-bond HMBC identified C-13 (δ 174.4). Initially, it was unexpected that the resonance for C-13 (an alkene) would be downfield of that for C-15 (a carbonyl) but comparison with the chemical shifts^29^ for 4-ethyl-5*H*-furan-2-one gave precedent. Moreover, the assignment was shown to be correct by HMBC from H-12 (δ 2.25) to C-13. HSQC then identified the other H-12 (δ 2.15) and C-12 (δ 21.9). HMBC from both H-12 led to C-11 (δ 34.38), confirmed by interactions of C-12 with both H-11 (δ 1.50, δ 1.98). C-9 resonated at δ 38.0 and was linked by HMBC to the two upfield methyl signals, δ 0.78 for H-17 and δ 0.65 for H-20. A 3-bond HMBC from H-20 led to C-10 (δ 46.3) and thence H-10 (δ 1.38). C-10 formed a HMBC cross-peak to H-8 (δ 1.24), from which C-8 (δ 4.1) was identified by HSQC. Confirmation was supplied by HMBC from C-8 to C-17 and C-20. A weak 4-bond HMBC from H-17 to C-6 (δ 72.7) identified the latter. H-6 resonated at δ 3.63. HMBC from C-6 to δ_H_ 1.43 led to identification of one H-7, from which the other H-7 (δ 1.50) and C-7 (δ 34.41) were correlated by HSQC. C-6 also gave an HMBC with H-10, closing the lower-right carbocycle, while a cross-peak from the upfield H-7 located C-5 (δ 45.2). C-5 was shown to carry C-19 by HMBC to both H-19 (δ 4.34, δ 4.49, geminally coupled doublets). Both H-19 linked on to the carbonyl at δ 170.8, demonstrating that C-19 carries an AcO- group. C-5 also gave HMBC cross-peaks to both H-18 (δ 2.84, δ 3.09). HSQC identified C-18 (δ 43.0) and 2-bond HMBC from both H-18 located the signal for C-4 at δ 69.6. A weaker HMBC interaction between C-4 and HO-3 (δ 7.76) linked on to C-3 (δ 64.5) and thence H-3 (δ 3.82). Finally, the remaining CH_2_s were identified through further HMBC (H-2: δ 1.24, δ 1.55, C-2 δ 34.42; H-1: δ 1.50, δ 1.66, C-1 δ 20.0). The structure shown in Fig. 1 was assigned to **19**, fatimanol W.

### Compound 20

Instability under MS conditions precluded obtaining useful spectra. The ^13^C NMR spectrum (in (CD_3_)_2_SO) (Table S7, SI) contained twenty discrete ^13^C signals: 2 × CH_3_, 8 × CH_2_, 5 × CH, and 5 × C_q_. The combined NMR spectra showed it to be very similar to **19**, with similar HMBC and HSQC connectivities. This compound lacked an acetate ester. Relative to **19**, the signals for H-19 and C-19 have moved markedly upfield (δ_H_ 3.78, δ_H_ 3.98, δ_C_ 60.3). This was consistent with the C-19 substituent being HO-. This was confirmed by observation of the HO-19 signal as a dd at δ 4.12, with HMBC to C-5 and C-19. Changes in chemical shift were also seen for the oxirane H-18 (δ 2.70, δ 2.89) and C-18 (δ 40.5), and for C-5 (δ 46.9), reflecting changes in steric effects in that region and the absence of through-space effects from an ester carbonyl. Thus **20** (fatimanol X) was the desacetyl analogue of **19**, as shown in Fig. 1.

**Figure 3:**
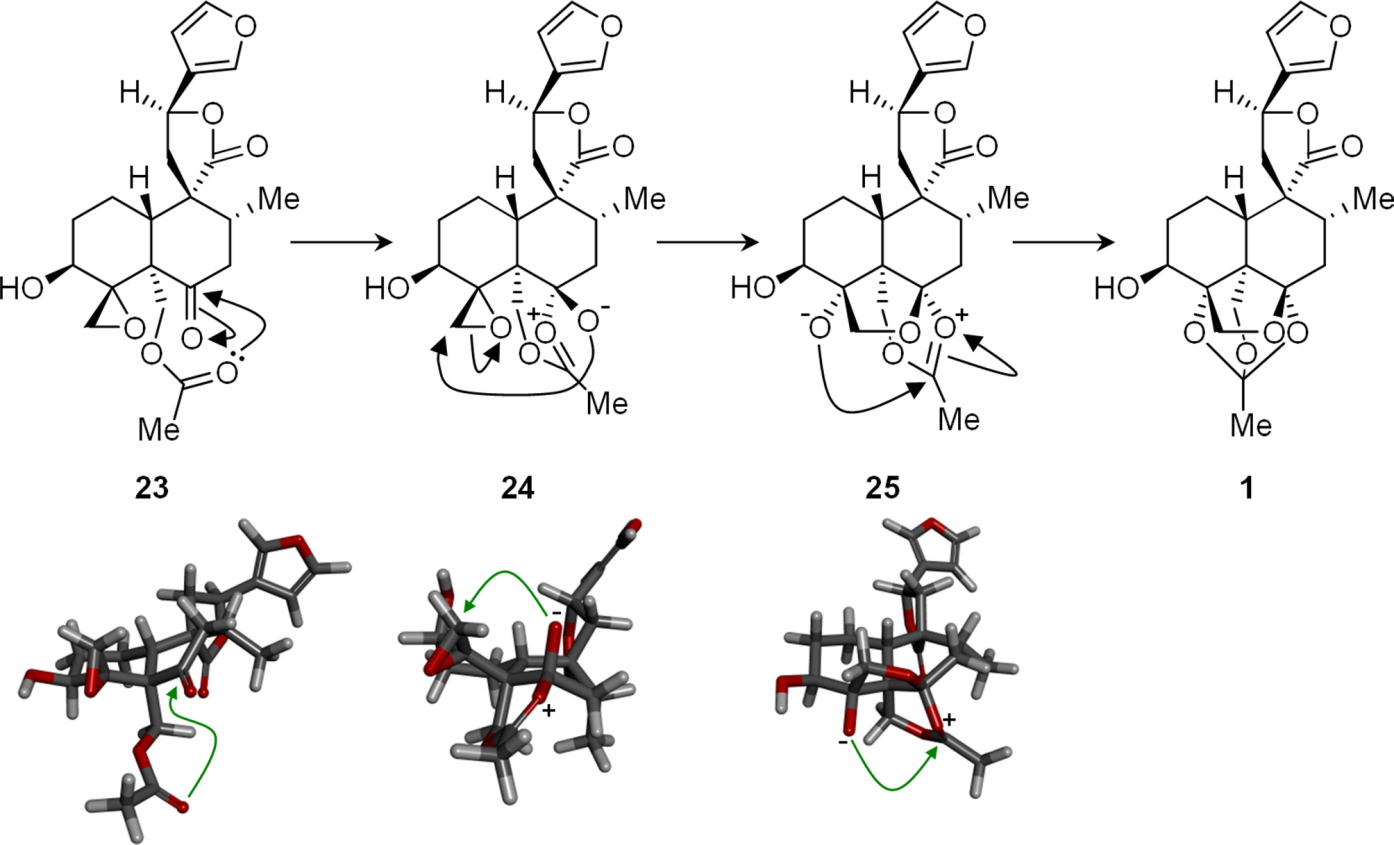
Proposed mechanism of tandem cyclisations forming orthoester 1 from acetate ester 23, noting apposite positions of ketone and oxirane electrophiles.

The occurrence of orthoesters in plant natural products was reviewed by Liao et al*.* in 2008^28^. Orthoacetates have been reported in only a limited number of frameworks, principally the daphnane diterpenoids, phragmalin limonoids, bufadienolide and ergostanoid steroids. Several of these orthoacetates have potent biological activities, including systemic toxicity. In each case, the three oxygen atoms of the orthoacetate unit were situated appositely in space for formation of the orthoester and the rigidity of the framework of the diterpene contributed to the stability of this usually highly acid-labile functionality. We propose the mechanism shown in Fig. 3 for formation of the orthoacetate in **1**. Proposed intermediate **23** is 19-acetylteulepicin, reported by Savona et al.^16^ to be a secondary metabolite in *T. buxifolium* and the formal oxidation product of **2** at the lactol. It is therefore feasible that **23** is the true biosynthetic precursor of **1** in *T. yemense*. The carbonyl oxygen of the acetate attacks the adjacent electrophilic ketone carbonyl from the lower face, generating intermediate **24**. Here the alkoxide anion is held close to the CH_2_ of the electrophilic oxirane and attacks it, opening the strained ring and forming a new bond. Finally, the new alkoxide in **30** is perfectly placed for attack as the electrophilic carbon of the acetate to form the orthoester **1**. Bruno et al.^30^ observed a related cyclisation from pyrolysis of fruticolone (a constituent of *T. fruticans*) at 200 °C and a chemical acid-catalysed epoxyester-orthoester rearrangement has been reported^31^ in a synthesis of petuniasterone D.

**Figure 4:**
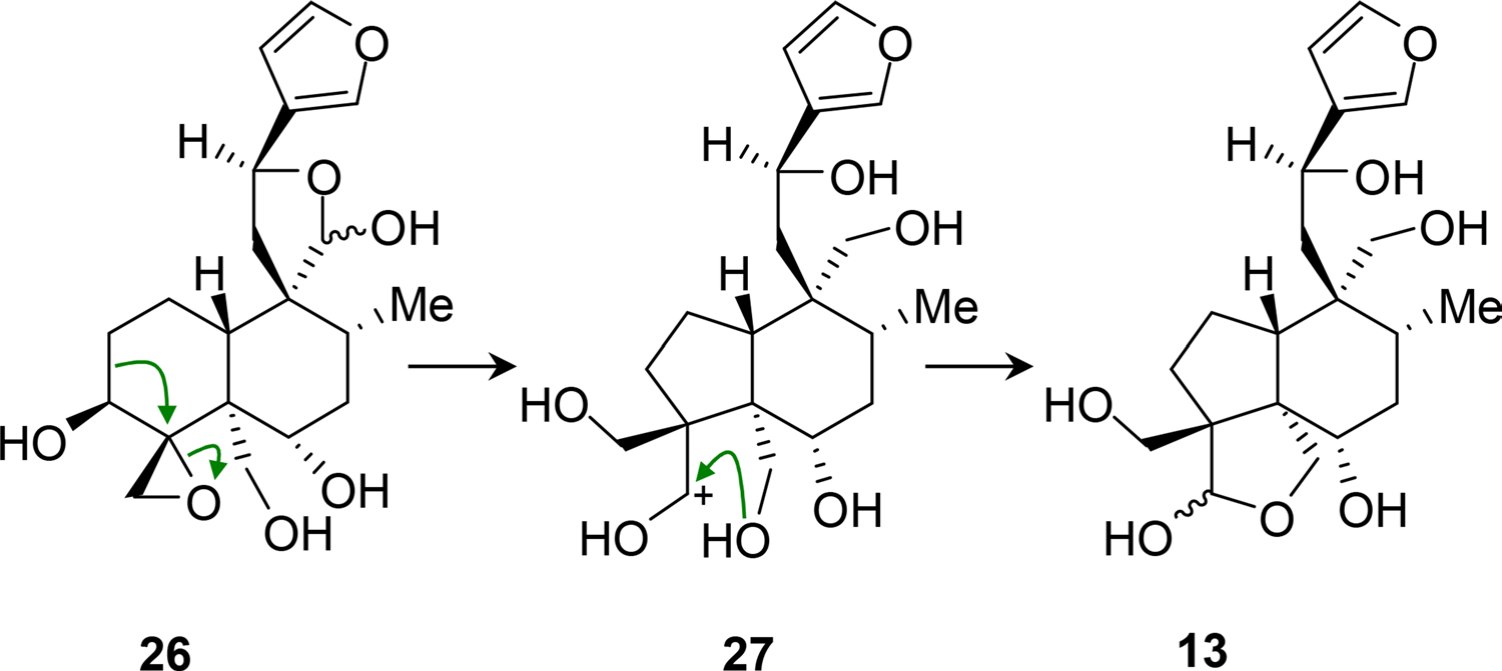
Proposed mechanism of rearrangement/ring-contraction of 26 to form 13.

Neoclerodanes with the A-ring contracted to a cyclopentane, as in **13**, have been reported previously as plant natural products but with more complex substitution patterns. Examples with a cyclobutene fused to the cyclopentane were reported in the 1980s.^32,33^ Ring-contracted neoclerodanes were later identified^34–37^ from *Pteronia*, *Conyza*, and *Microglossa* species. In these neoclerodanes, there is extensive transannular bridging. Fatimanol Q **13** is the simplest ring-contracted neoclerodane identified to date. Bohlmann’s group suggested that their ring-contracted compounds arose from rearrangements involving migration of C-2 to bond with C-4. Mechanisms proposed include protonation of a hydroxy group at C-4 to initiate the rearrangement,^34^ trapping the aldehyde formed from C-3 with a hydroxy group at C-10^34,35^ and formation of a C-1 = C-10 double bond.^34,36,37^ In each case, the C-10 position is oxidised. However, in the present case, C-10 is not oxidised with either an oxygen function or an alkene. We propose the mechanism in Fig. 4 for the biosynthetic ring-contraction. In this mechanism, the rearrangement is triggered by protonation and ring-opening of the strained oxirane. The leaving group oxygen is on the lower face of the ring and thus almost antiperiplanar to the C-3–C-2 bond. The oxirane-opening and the migration of C-2 are probably concerted, given this conformation. C-2 will approach C-4 from the opposite side to the leaving group, resulting in stereochemical inversion at C-4. This places C-18 on the upper face and the newly generated carbocation (C-3) on the lower face, where it can readily form a hemiacetal with HO-19. This hemiacetal closes the second 5-membered ring such that the two 5-membered rings are *cis*-fused, an energetically favoured arrangement.

Acid anhydrides are relatively uncommon in natural products, owing to their potential for electrophilic reactivity and hydrolysis. However, we have firmly identified **10** as having a succinic anhydride as the upper ring; this is the first such neoclerodane to be reported. Only one natural product containing a simple (non-fused) succinic anhydride, tubogenic anhydride A, has previously been isolated, from *Aspergillus tubingensis*^38^.

Compounds **2,3,7–9,11,12,14–17,19,20** contain the conventional neoclerodane carbon framework with various differences in oxidation level, acetylation, bridging rings and ring-opening at the C-4/C-18 oxirane, whereas **4–6,18** lack C-19. The C-12–C-20 lactol in **2** has precedent in gnaphalin (isolated from *T. gnaphalodes*)^39^, although the latter lacks HO-3. The C-19–C-20 bridging lactone of **3,7,10** is present in teulepicephin^15^ and many other neoclerodanes. Compound **11** contains a related bridging acetal, giving a rigid polycyclic structure. This polycycle is also present in teucrin P_1_ (also from *T. gnaphalodes*)^40^, teupyrenone (from *T. pyrenaicum*)^41^, and teupolin III^42^, although the latter do not have the additional lower fused tetrahydrofuran to stiffen the structure further. The upper hydroxyfuranone hemiacetal in **7** is also present in the neoclerodane salvidivin (from *Salvia divinorum*)^22^, whereas the methoxyfuranone acetal moiety in **8,9** has precedent in the labdane 15-methoxyvelutine C (from *Marrubium thessalum*)^43^. Most neoclerodanes have the 12-*S* configuration, so the identification of **16** as a 12-*R* neoclerodane is noteworthy. Gács-Baitz et al.^42^ used NOE NMR spectroscopy to determine configuration at C-12 in neoclerodanes featuring the upper aromatic furan and the *spiro*-lactone but did not have an exact epimeric pair for their study; **15** and **16** are exact epimers which facilitated their stereochemical identification. The lower hydroxyfuranone hemiacetal of **4,5** and the corresponding methoxyfuranone acetal feature of **6** have scant precedent, in teucvisin C^44^ and cracroson B^45^, respectively, while the dimethoxydihydrofuran diacetal of **18** is completely novel in the series. The ajugamarins and related neoclerodanes have the upper furanone unit of **19** and **20** but all known ajugamarins are oxygenated at C-12^46^.

**Figure 5:**
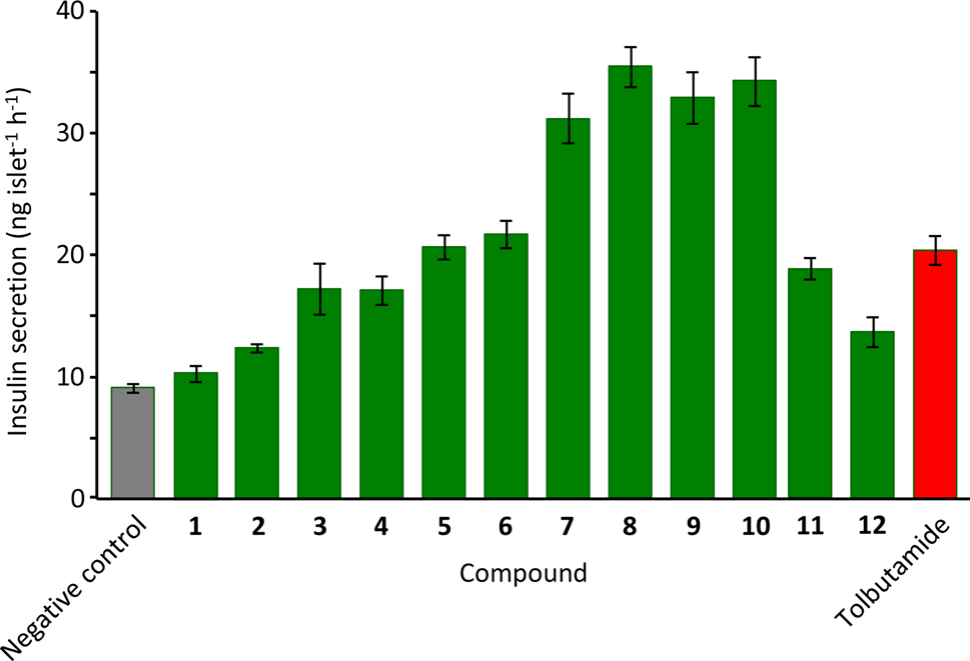
Effects of neoclerodanes 1–12 (from *T. yemense*) on the glucose-triggered secretion of insulin from murine islets. Islets were incubated for 1 h at 37 °C in KRB buffer containing glucose (16.7 mM) in the absence (negative control) or presence of test compounds and the secreted insulin was measured. Test compounds and positive control tolbutamide were used at the single concentration 200 µM. Values are mean ± SD from three independent experiments.

Compounds **1**–**12** were evaluated for their ability to enhance the glucose-triggered secretion of insulin by freshly isolated murine pancreatic islets, using our previous assay^47^. In negative-control islets, insulin secretion was 9.1 ± 0.3 ng islet^−1^ h^−1^, triggered by glucose (16.7 mM) (Fig. 5). This release was increased 2.2-fold by the standard drug tolbutamide (20.2 ± 1.3 ng islet^−1^ h^−1^). The tested compounds showed a range of activities. Compounds **1**,**2**,**12** showed little or no effect on the secretion of insulin. Compounds **3**–**6** and **11** increased the glucose-triggered release of insulin by approximately the same extent as the positive control tolbutamide. Encouragingly, **7**–**10** showed strong enhancement of insulin secretion, by 3–4 × , although not as potently as the coumarins cluteolin D and clueolin J (from *Clutia lanceolata*)^48^.

## Conclusions

We report the isolation and identification of twenty new neoclerodanes from the traditional medicinal plant *T. yemense*. Compound **1** contains an orthoacetate, which is previously unreported in naturally-occurring neoclerodanes. As shown (Fig. 3), the acetate, oxirane and ketone groups in proposed precursor **23** are appositely located to facilitate formation of the orthoester; precursor **23** is 19-acetylteulepicin, previously identified in *T. buxifolium*. The upper (tetrahydro)furan unit in **10** is a succinic anhydride, a reactive moiety not often found in plants but presumably stable in the arid climate in which *T. yemense* grows in nature. Compound **13** results from a relatively unusual ring-contracting skeletal rearrangement during biosynthesis. Interestingly, **7**–**10** were found to enhance the glucose-triggered release of insulin from isolated murine pancreatic islets to a greater extent than the standard anti-diabetic drug tolbutamide; these compounds represent new leads for the development of treatments for this widespread disease.

## Supplementary Information

Supplementary Information.
