# Supplementary material for: A neoclerodane orthoester and other new neoclerodane diterpenoids from Teucrium yemense chemistry and effect on secretion of insulin
Source: Sci Rep. 2021 Apr 13;11:8074. doi: 10.1038/s41598-021-87513-3 (PMC8044157; doi:10.1038/s41598-021-87513-3)
Supplement: Supplementary file 1 — Supplementary Information. [file 41598_2021_87513_MOESM1_ESM.pdf]

A neoclerodane orthoester and other new neoclerodane diterpenoids from *Teucrium yemense* chemistry and effect on secretion of insulin

Mohammad Nur-e-Alam,[1],* Ifat Parveen,[2] Barrie Wilkinson,[3] Sarfaraz Ahmed,[1] Rahman M. Hafizur, [4] Ahmed Bari,[5] Timothy J. Woodman,[6] Michael D. Threadgill[2,6] & Adnan J. Al-Rehaily[1]

[1] Department of Pharmacognosy, College of Pharmacy, King Saud University, P.O. Box. 2457, Riyadh 11451, Kingdom of Saudi Arabia

[2] Institute of Biological, Environmental & Rural Sciences (IBERS), Aberystwyth University, Aberystwyth SY23 3DA, United Kingdom

[3] John Innes Centre, Norwich Research Park, Norwich, NR4 7UH, United Kingdom

[4] Dr. Panjwani Center for Molecular Medicine and Drug Research, International Center for Chemical and Biological Sciences, University of Karachi, Karachi-75270, Pakistan

[5]Department of Pharmaceutical Chemistry, College of Pharmacy, King Saud University, P.O. Box. 2457, Riyadh 11451, Kingdom of Saudi Arabia

Drug & Target Discovery, Department of Pharmacy and Pharmacology, University of Bath, Claverton Down, Bath, BA2 7AY, United Kingdom

Contents **Page**

**Table S1**	^{1}H and ^{13}C NMR data for **1-3**.	9
**Table S2.**	^{1}H and ^{13}C NMR data for **4-7**.	10
**Table S3.**	^{1}H and ^{13}C NMR data for **8-11**.	11
**Table S4.**	^{1}H and ^{13}C NMR data for **12-14**.	12
**Table S5.**	^{1}H and ^{13}C NMR data for **15,16**.	13
**Table S6.**	^{1}H and ^{13}C NMR data for **17,18**.	14
**Table S7.**	^{1}H and ^{13}C NMR data for **19,20**.	15
**Figure S1.**	^{1}H NMR spectrum of **1** in CD_3OD.	16
**Figure S2.**	Expansion of part of ^{1}H NMR spectrum (δ 1.0 – δ 2.7) of **1** in CD_3OD.	17
**Figure S3.**	Expansion of part of ^{1}H NMR spectrum (δ 3.7 – δ 5.6) of **1** in CD_3OD.	18
**Figure S4.**	^{1}H NMR spectrum of **1** in $(CD_3)_2SO$.	19
**Figure S5.**	^{13}C NMR spectrum of **1** in CD_3OD.	20
**Figure S6.**	COSY NMR spectrum of **1** in CD_3OD.	21
**Figure S7.**	HSQC NMR spectrum of **1** in CD_3OD.	22
**Figure S8.**	HMBC NMR spectrum of **1** in CD_3OD.	23
**Figure S9.**	Positive-ion HRESIMS of **1**.	24
**Figure S10.**	^{1}H NMR spectrum of **2** in CD_3OD.	25

Figure S11. Expansion of part of ^{1}H NMR spectrum (δ 0.9 – δ 3.3) of **2** in CD_3OD. 26
Figure S12. Expansion of part of ^{1}H NMR spectrum (δ 3.9 – δ 5.6) of **2** in CD_3OD. 27
Figure S13. ^{13}C NMR spectrum of **2** in CD_3OD. 28
Figure S14. COSY NMR spectrum of **2** in CD_3OD. 29
Figure S15. HSQC NMR spectrum of **2** in CD_3OD. 30
Figure S16. HMBC NMR spectrum of **2** in CD_3OD. 31
Figure S17. Positive-ion HRESIMS of **2**. 32
Figure S18. ^{1}H NMR spectrum of **3** in CD_3OD. 33
Figure S19. Expansion of part of ^{1}H NMR spectrum (δ 0.6 – δ 2.6) of **3** in CD_3OD. 34
Figure S20. Expansion of part of ^{1}H NMR spectrum (δ 3.75 – δ 4.95) of **3** in CD_3OD. 35
Figure S21. ^{13}C NMR spectrum of **3** in CD_3OD. 36
Figure S22. COSY NMR spectrum of **3** in CD_3OD. 37
Figure S23. NOESY NMR spectrum of **3** in CD_3OD. 38
Figure S24. HSQC NMR spectrum of **3** in CD_3OD. 39
Figure S25. HMBC NMR spectrum of **3** in CD_3OD. 40
Figure S26. Positive-ion HRESIMS of **3**. 41
Figure S27. Negative-ion HRESIMS of **3**. 42
Figure S28. ^{1}H NMR spectrum of **4** in CD_3OD. 43
Figure S29. Expansion of part of ^{1}H NMR spectrum (δ 0.5 – δ 3.0) of **4** in CD_3OD. 44
Figure S30. Expansion of part of ^{1}H NMR spectrum (δ 4.3 – δ 7.7) of **4** in CD_3OD. 45
Figure S31. ^{13}C NMR spectrum of **4** in CD_3OD / $CDCl_3$. 46
Figure S32. COSY NMR spectrum of **4** in CD_3OD. 47
Figure S33. NOESY NMR spectrum of **4** in CD_3OD. 48
Figure S34. HSQC NMR spectrum of **4** in CD_3OD. 49
Figure S35. HMBC NMR spectrum of **4** in CD_3OD. 50
Figure S36. Positive-ion HRESIMS of **4**. 51
Figure S37. ^{1}H NMR spectrum of **5** in CD_3OD. 52
Figure S38. Expansion of part of ^{1}H NMR spectrum (δ 0.9 – δ 3.0) of **5** in CD_3OD. 53
Figure S39. Expansion of part of ^{1}H NMR spectrum (δ 5.3 – δ 7.9) of **5** in CD_3OD. 54
Figure S40. ^{13}C NMR spectrum of **5** in CD_3OD. 55
Figure S41. COSY NMR spectrum of **5** in CD_3OD. 56
Figure S42. HSQC NMR spectrum of **5** in CD_3OD. 57
Figure S43. HMBC NMR spectrum of **5** in CD_3OD. 58

**Figure S44.**	Positive-ion HRESIMS of **5**.	59
**Figure S45.**	^{1}H NMR spectrum of **6** in CD_3OD.	60
**Figure S46.**	Expansion of part of ^{1}H NMR spectrum (δ 0.7 – δ 3.4) of **6** in CD_3OD.	61
**Figure S47.**	Expansion of part of ^{1}H NMR spectrum (δ 4.3 – δ 7.7) of **6** in CD_3OD.	62
**Figure S48.**	^{13}C NMR spectrum of **6** in CD_3OD.	63
**Figure S49.**	COSY NMR spectrum of **6** in CD_3OD.	64
**Figure S50.**	HSQC NMR spectrum of **6** in CD_3OD.	65
**Figure S51.**	HMBC NMR spectrum of **6** in CD_3OD.	66
**Figure S52.**	Positive-ion HRESIMS of **6**.	67
**Figure S53.**	^{1}H NMR spectrum of **7** in CD_3OD.	68
**Figure S54.**	Expansion of part of ^{1}H NMR spectrum (δ 0.7 – δ 2.8) of **7** in CD_3OD.	69
**Figure S55.**	Expansion of part of ^{1}H NMR spectrum (δ 3.7 – δ 7.4) of **7** in CD_3OD.	70
**Figure S56.**	^{13}C NMR spectrum of **7** in CD_3OD.	71
**Figure S57.**	COSY NMR spectrum of **7** in CD_3OD.	72
**Figure S58.**.	NOESY NMR spectrum of **7** in CD_3OD.	73
**Figure S59.**	HSQC NMR spectrum of **7** in CD_3OD.	74
**Figure S60.**	HMBC NMR spectrum of **7** in CD_3OD.	75
**Figure S61.**	Negative-ion HRESIMS of **7**.	76
**Figure S62.**	^{1}H NMR spectrum of **8** in CD_3OD.	77
**Figure S63.**	Expansion of part of ^{1}H NMR spectrum (δ 0.4 – δ 2.6) of **8** in CD_3OD.	78
**Figure S64.**	Expansion of part of ^{1}H NMR spectrum (δ 2.9 – δ 4.7) of **8** in CD_3OD.	79
**Figure S65.**	^{13}C NMR spectrum of **8** in CD_3OD.	80
**Figure S66.**	COSY NMR spectrum of **8** in CD_3OD.	81
**Figure S67.**	NOESY NMR spectrum of **8** in CD_3OD.	82
**Figure S68.**	HSQC NMR spectrum of **8** in CD_3OD.	83
**Figure S69.**.	HMBC NMR spectrum of **8** in CD_3OD.	84
**Figure S70.**	Positive-ion HRESIMS of **8**.	85
**Figure S71.**	^{1}H NMR spectrum of **9** in CD_3OD.	86
**Figure S72.**	Expansion of part of ^{1}H NMR spectrum (δ 0.4 – δ 2.6) of **9** in CD_3OD.	87
**Figure S73.**	Expansion of part of ^{1}H NMR spectrum (δ 2.9 – δ 4.8) of **9** in CD_3OD.	88
**Figure S74.**	^{13}C NMR spectrum of **9** in CD_3OD.	89
**Figure S75.**	COSY NMR spectrum of **9** in CD_3OD.	90
**Figure S76.**	NOESY NMR spectrum of **9** in CD_3OD.	91

Figure S77. HSQC NMR spectrum of **9** in CD_3OD. 92
Figure S78. HMBC NMR spectrum of **9** in CD_3OD. 93
Figure S79. Positive-ion HRESIMS of **9**. 94
Figure S80. ^1H NMR spectrum of **10** in CD_3OD. 95
Figure S81. Expansion of part of ^1H NMR spectrum (δ 0.6 – δ 2.7) of **10** in CD_3OD. 96
Figure S82. Expansion of part of ^1H NMR spectrum (δ 3.5 – δ 4.7) of **10** in CD_3OD. 97
Figure S83. ^{13}C NMR spectrum of **10** in CD_3OD. 98
Figure S84. COSY NMR spectrum of **10** in CD_3OD. 99
Figure S85. HSQC NMR spectrum of **10** in CD_3OD. 100
Figure S86. HMBC NMR spectrum of **10** in CD_3OD. 101
Figure S87. Positive-ion and negative-ion HRESIMS of **10**. 102
Figure S88. ^1H NMR spectrum of **11** in CD_3OD. 103
Figure S89. Expansion of part of ^1H NMR spectrum (δ 0.2 – δ 2.6) of **11** in CD_3OD. 104
Figure S90. Expansion of part of ^1H NMR spectrum (δ 3.4 – δ 7.5) of **11** in CD_3OD. 105
Figure S91. ^{13}C NMR spectrum of **11** in CD_3OD. 106
Figure S92. COSY NMR spectrum of **11** in CD_3OD. 107
Figure S93. NOESY NMR spectrum of **11** in CD_3OD. 108
Figure S94. HSQC NMR spectrum of **11** in CD_3OD. 109
Figure S95. HMBC NMR spectrum of **11** in CD_3OD. 110
Figure S96. ^1H NMR spectrum of **12** in CD_3OD. 111
Figure S97. Expansion of part of ^1H NMR spectrum (δ 0.6 – δ 2.9) of **12** in CD_3OD. 112
Figure S98. Expansion of part of ^1H NMR spectrum (δ 3.6 – δ 7.8) of **12** in CD_3OD. 113
Figure S99. ^{13}C NMR spectrum of **12** in CD_3OD. 114
Figure S100. COSY NMR spectrum of **12** in CD_3OD. 115
Figure S101. NOESY NMR spectrum of **12** in CD_3OD. 116
Figure S102. HSQC NMR spectrum of **12** in CD_3OD. 117
Figure S103. HMBC NMR spectrum of **12** in CD_3OD. 118
Figure S104. Positive-ion HRESIMS of **12**. 119
Figure S105. ^1H NMR spectrum of **13** in $(CD_3)_2SO$. 120
Figure S106. Expansion of part of ^1H NMR spectrum (δ 0.9 – δ 5.1) of **13** in $(CD_3)_2SO$. 121
Figure S107. ^{13}C NMR spectrum of **13** in $(CD_3)_2SO$. 122
Figure S108. 135DEPT NMR spectrum of **13** in $(CD_3)_2SO$. 123
Figure S109. COSY NMR spectrum of **13** in $(CD_3)_2SO$. 124

Figure S110. HSQC NMR spectrum of **13** in $(CD_3)_2SO$. 125
Figure S111. HMBC NMR spectrum of **13** in $(CD_3)_2SO$. 126
Figure S112. Positive-ion HRESIMS of **13**. 127
Figure S113. Negative-ion HRESIMS of **13**. 128
Figure S114. ^1H NMR spectrum of **14** in $CDCl_3$. 129
Figure S115. Expansion of part of ^1H NMR spectrum (δ 0.7 – δ 2.5) of **14** in $CDCl_3$. 130
Figure S116. Expansion of part of ^1H NMR spectrum (δ 2.8 – δ 5.2) of **14** in $CDCl_3$. 131
Figure S117. Expansion of part of ^1H NMR spectrum (δ 6.2 – δ 7.7) of **14** in $CDCl_3$. 132
Figure S118. ^{13}C NMR spectrum of **14** in $(CD_3)_2SO$. 133
Figure S119. 135DEPT NMR spectrum of **14** in $(CD_3)_2SO$. 134
Figure S120. COSY ^1H NMR spectrum of **14** in $CDCl_3$. 135
Figure S121. HSQC NMR spectrum of **14** in $(CD_3)_2SO$. 136
Figure S122. HSQC ^1H NMR spectrum of **14** in $CDCl_3$. 137
Figure S123. Expansion of part of HSQC NMR spectrum (δ 0.7 – δ 2.5) of **14** in $CDCl_3$. 138
Figure S124. Expansion of part of HSQC NMR spectrum (δ 3.0 – δ 4.9) of **14** in $CDCl_3$. 139
Figure S125. HMBC NMR spectrum of **14** in $(CD_3)_2SO$. 140
Figure S126.. Positive-ion HRESIMS of **14**. 141
Figure S127. Negative-ion HRESIMS of **14**. 142
Figure S128. ^1H NMR spectrum of **15** in $(CD_3)_2SO$. 143
Figure S129. Expansion of part of ^1H NMR spectrum (δ 0.8 – δ 3.2) of **15** in $(CD_3)_2SO$. 144
Figure S130. Expansion of part of ^1H NMR spectrum (δ 3.1 – δ 5.5) of **15** in $(CD_3)_2SO$. 145
Figure S131. ^1H NMR spectrum of **15** in $CDCl_3$. 146
Figure S132. Expansion of part of ^1H NMR spectrum (δ 0.8 – δ 3.2) of **15** in $CDCl_3$. 147
Figure S133. Expansion of part of ^1H NMR spectrum (δ 3.3 – δ 5.6) of **15** in $CDCl_3$. 148
Figure S134. Expansion of part of ^1H NMR spectrum (δ 5.8 – δ 7.8) of **15** in $CDCl_3$. 149
Figure S135. ^{13}C NMR spectrum of **15** in $(CD_3)_2SO$. 150
Figure S136. 135DEPT NMR spectrum of **15** in $(CD_3)_2SO$. 151
Figure S137. COSY NMR spectrum of **15** in $CDCl_3$. 152
Figure S138. Expansion of part of COSY NMR spectrum of **15** in $CDCl_3$. 153
Figure S139. NOESY NMR spectrum of **15** in $(CD_3)_2SO$. 154
Figure S140. HSQC NMR spectrum of **15** in $(CD_3)_2SO$. 155
Figure S141. HSQC NMR spectrum of **15** in $CDCl_3$. 156
Figure S142. Expansion of part of HSQC NMR spectrum (δ 0.7 – δ 3.2) of **15** in $CDCl_3$. 157

Figure S143. Expansion of part of HSQC NMR spectrum (δ 2.9 – δ 5.5) of **15** in $CDCl_3$. 158
Figure S144. HMBC NMR spectrum of **15** in $(CD_3)_2SO$. 159
Figure S145. Positive-ion HRESIMS of **15**. 160
Figure S146. Negative-ion HRESIMS of **15**. 161
Figure S147. ^1H NMR spectrum of **16** in $(CD_3)_2SO$. 162
Figure S148. Expansion of part of ^1H NMR spectrum (δ 0.9 – δ 3.2) of **16** in $(CD_3)_2SO$. 163
Figure S149. Expansion of part of ^1H NMR spectrum (δ 3.6 – δ 5.5) of **16** in $(CD_3)_2SO$. 164
Figure S150. ^1H NMR spectrum of **16** in $CDCl_3$. 165
Figure S151. Expansion of part of ^1H NMR spectrum (δ 0.7 – δ 3.5) of **16** in $CDCl_3$. 166
Figure S152. Expansion of part of ^1H NMR spectrum (δ 3.7 – δ 7.9) of **16** in $CDCl_3$. 167
Figure S153. ^{13}C NMR spectrum of **16** in $(CD_3)_2SO$. 168
Figure S154. 135DEPT NMR spectrum of **16** in $(CD_3)_2SO$. 169
Figure S155. COSY NMR spectrum of **16** in $(CD_3)_2SO$. 170
Figure S156. COSY NMR spectrum of **16** in $CDCl_3$. 171
Figure S157. Expansion of part of COSY NMR spectrum (δ 0.9 – δ 5.5) of **16** in $CDCl_3$. 172
Figure S158. NOESY NMR spectrum of **16** in $(CD_3)_2SO$. 173
Figure S159. HSQC NMR spectrum of **16** in $(CD_3)_2SO$. 174
Figure S160. HSQC NMR spectrum of **16** in $CDCl_3$. 175
Figure S161. Expansion of part of HSQC NMR spectrum (δ 1.0 – δ 3.1) of **16** in $CDCl_3$. 176
Figure S162. Expansion of part of HSQC NMR spectrum (δ 2.9 – δ 5.4) of **16** in $CDCl_3$. 177
Figure S163. HMBC NMR spectrum of **16** in $(CD_3)_2SO$. 178
Figure S164. Positive-ion HRESIMS of **16**. 179
Figure S165. ^1H NMR spectrum of **17** in $(CD_3)_2SO$. 180
Figure S166. Expansion of part of ^1H NMR spectrum (δ 0.9 – δ 2.8) of **17** in $(CD_3)_2SO$. 181
Figure S167. Expansion of part of ^1H NMR spectrum (δ 3.6 – δ 5.5) of **17** in $(CD_3)_2SO$. 182
Figure S168. ^1H NMR spectrum of **17** in $CDCl_3$. 183
Figure S169. Expansion of part of ^1H NMR spectrum (δ 0.7 – δ 3.2) of **17** in $CDCl_3$. 184
Figure S170. Expansion of part of ^1H NMR spectrum (δ 2.8 – δ 5.8) of **17** in $CDCl_3$. 185
Figure S171. Expansion of part of ^1H NMR spectrum (δ 6.0 – δ 7.7) of **17** in $CDCl_3$. 186
Figure S172. ^{13}C NMR spectrum of **17** in $(CD_3)_2SO$. 187
Figure S173. 135DEPT NMR spectrum of **17** in $(CD_3)_2SO$. 188
Figure S174. COSY NMR spectrum of **17** in $(CD_3)_2SO$. 189
Figure S175. COSY NMR spectrum of **17** in $CDCl_3$. 190

Figure S176. Expansion of part of COSY NMR spectrum (δ 0.5 – δ 5.5) of **17** in $CDCl_3$. 191
Figure S177. HSQC NMR spectrum of **17** in $(CD_3)_2SO$. 192
Figure S178. HSQC NMR spectrum of **17** in $CDCl_3$. 193
Figure S179. Expansion of part of HSQC NMR spectrum (δ 0.8 – δ 2.7) of **17** in $CDCl_3$. 194
Figure S180. Expansion of part of HSQC NMR spectrum (δ 3.6 – δ 5.6) of **17** in $CDCl_3$. 195
Figure S181. HMBC NMR spectrum of **17** in $(CD_3)_2SO$. 196
Figure S182. Positive-ion HRESIMS of **17**. 197
Figure S183. Negative-ion HRESIMS of **17**. 198
Figure S184. ^1H NMR spectrum of **18** in $(CD_3)_2SO$. 199
Figure S185. Expansion of part of ^1H NMR spectrum (δ 0.9 – δ 2.7) of **18** in $(CD_3)_2SO$. 200
Figure S186. ^1H NMR spectrum of **18** in $CDCl_3$. 201
Figure S187. Expansion of part of ^1H NMR spectrum (δ 0.8 – δ 3.7) of **18** in $CDCl_3$. 202
Figure S188. Expansion of part of ^1H NMR spectrum (δ 3.0 – δ 7.8) of **18** in $CDCl_3$. 203
Figure S189. ^{13}C NMR spectrum of **18** in $(CD_3)_2SO$. 204
Figure S190. 135DEPT NMR spectrum of **18** in $(CD_3)_2SO$. 205
Figure S191. COSY NMR spectrum of **18** in $(CD_3)_2SO$. 206
Figure S192. HSQC NMR spectrum of **18** in $(CD_3)_2SO$. 207
Figure S193. HMBC NMR spectrum of **18** in $(CD_3)_2SO$. 208
Figure S194. Positive-ion HRESIMS of **18**. 209
Figure S195. ^1H NMR spectrum of **19** in $(CD_3)_2SO$. 210
Figure S196. Expansion of part of ^1H NMR spectrum (δ 0.75 – δ 2.3) of **19** in $(CD_3)_2SO$. 211
Figure S197. ^{13}C NMR spectrum of **19** in $(CD_3)_2SO$. 212
Figure S198. 135DEPT NMR spectrum of **19** in $(CD_3)_2SO$. 213
Figure S199. HSQC NMR spectrum of **19** in $(CD_3)_2SO$. 214
Figure S200. HMBC NMR spectrum of **19** in $(CD_3)_2SO$. 215
Figure S201. ^1H NMR spectrum of **20** in $(CD_3)_2SO$. 216
Figure S202. Expansion of part of ^1H NMR spectrum (δ 0.6 – δ 2.3) of **20** in $(CD_3)_2SO$. 217
Figure S203. Expansion of part of ^1H NMR spectrum (δ 2.5 – δ 4.2) of **20** in $(CD_3)_2SO$. 218
Figure S204. ^{13}C NMR spectrum of **20** in $(CD_3)_2SO$. 219
Figure S205. 135DEPT NMR spectrum of **20** in $(CD_3)_2SO$. 220
Figure S206. HSQC NMR spectrum of **20** in $(CD_3)_2SO$. 221
Figure S207. HMBC NMR spectrum of **20** in $(CD_3)_2SO$. 222
Figure S208. Fractionation tree for isolation of compounds **1**-**20**. 223

General analytical and chromatographic procedures. 224
Assay for enhancement of insulin secretion. 225

Table S1. ^{1}H and ^{13}C NMR data for **1-3.**

	**1** (CD_3OD)		**1** ($(CD_3)_2SO$)	**2** (CD_3OD)		**3** (CD_3OD)	
**Position**	**δ_H mult. (*J* Hz)**	**δ_C type**	**δ_H mult. (*J* Hz)**	**δ_H mult. (*J* Hz)**	**δ_C type**	**δ_H mult. (*J* Hz)**	**δ_C type**
1	1.29 m, 1.7 m	22.0 CH_2		2.22 m,<br>2.64 brq (13.6)	22.2 CH_2	1.29 brq (13.2),<br>2.35 brd (13.0)	21.9 CH_2
2	1.7 m, 2.19 m	33.9 CH		1.36 d (6.8), 2.22 m	33.0 CH_2	1.40-1.46 m,<br>2.09-2.16 m	30.3 CH_2
3	4.24 dd (10.9, 6.5)	71.4 CH	4.07 m	4.11 dd (10.7, 4.2)	66.4 CH	3.87 dd (11.5, 5.2)	72.8 CH
4	-	87.1 C_q		-	54.3 C_q	-	84.107 C_q
5	-	43.8 C_q		-	63.6 C_q	-	49.4 C_q
6	-	107.8 C_q		-	206.2 C_q	-	110.0 C_q
7	2.03 m,<br>2.27 dd (14.1, 12.9)	35.2 CH_2		2.28 dd (15.0, 2.7),<br>2.72 brt (14.6)	46.2 CH_2	1.40-1.46 m,<br>2.09-2.16 m	35.8 *or* 36.0 CH_2
8	1.96 m	37.6 CH	1.8 m	1.83 m	42.9 CH	2.21 m	37.2 CH
9	-	51.4 C_q		-	53.6 C_q	-	48.5 C_q
10	1.89 d (11.2)	47.1 CH		2.22 m	54.3 CH	2.24-2.48 m	43.6 CH
11	2.49 m	42.9 CH_2		1.93 dd (12.5, 11.0),<br>2.34 dd (13.2, 6.8)	44.2 CH_2	2.09-2.16 m	33.0 *or* 35.3 CH_2
12	5.46 t (8.6)	73.5 CH		5.25 brdd, (9.8, 7.1)	71.2 CH	4.85 brd (9.7)	63.7 CH
13	-	126.3 C_q		-	124.8 C_q	-	130.1 C_q
14	6.47 brs	109.1 CH	6.52 brs	6.41 m	108.8 CH	6.42 m	108.4 CH
15	7.54 brs	145.6 CH	7.72 brs	7.43 m	143.8	7.40 m	143.9 CH
16	7.61 brs	141.8 CH	7.83 brs	7.42 m	139.6 CH	7.39 m	138.7 CH
17	1.08 d (6.8)	16.1 CH_3	1.00 d (6.7)	1.03 d (6.7)	15.6 CH_3	0.93 d (6.2)	16.6 CH_3
18	3.87 d (10.0),<br>4.39 d (10.0)	58.3 CH_2	4.31 d (9.8),<br>3.69 d (9.8)	2.81 d (5.3),<br>3.14 d (5.3)	43.676 CH_2	3.90 brd (10.9),<br>4.45 m	75.8 CH_2
19	3.93 d (10.2),<br>4.82 d (10.2)	74.6 CH_2	3.91 d (10.0),<br>4.61 d (10.1)	4.72 d (12.7),<br>4.83 d (12.7)	64.1 CH_2	4.45 m,<br>4.68 brd (12.5)	66.4 CH_2
20	-	179.3 C_q		5.54 s	99.7 CH	-	172.5 C_q
*Me*C(OR)$_3$	1.41 s	23.9 CH_3					
Me*C*(OR)$_3$	-	107.7 C_q					
HO-3			5.23 d (5.0)				
MeCO_2-19				2.07 s	21.1 CH_3		
Me*C*O_2-19				-	171.2 C_q		
MeO-6						3.40 s	48.9 CH_3

[a]Only selected peaks shown

Table S2. ^{1}H and ^{13}C NMR data for **4-7.**

Position	**4** (CD_3OD)		**5** (CD_3OD)		**6** (CD_3OD)		**7** (CD_3OD)	
	**δ_H mult. (*J* Hz)**	**δ_C type**	**δ_H mult. (*J* Hz)**	**δ_C type**	**δ_H mult. (*J* Hz)**	**δ_C type**	**δ_H mult. (*J* Hz)**	**δ_C type**
1	1.64 m, 2.3 m	29.9 CH_2	1.51 brq (11.3), 2.30 m	23.2 CH_2	1.65 m, 2.39 m	22.2 CH_2	1.3 m, 2.45 m	22.8 CH_2
2	1.66 m, 2.01 m	21.6 CH_2	1.67 brq (11.3), 2.14 m	29.0 CH_2	1.65 m, 2.01 m	31.3 CH_2	1.45 m, 2.05 m	32.8 CH_2
3	4.41 m	60.5 CH	5.59 m	64.7 CH	4.41 m	61.6 CH	3.83 dd (11.6, 5.9)	73.4 CH
4	-	128.5 C_q	-	133.9 C_q	-	133.0 C_q	-	85.6 C_q
5	-	163.0 C_q	-	162.9 C_q	-	162.4 C_q	-	49.7 C_q
6	-	102.4 C_q	-	103.5 C_q	-	105.8 C_q	-	108.2 C_q
7	2.2 m (0.5 H), 2.3 m (0.5 H), 2.4 m (0.5 H), 2.6 m (0.5 H)	39.9 CH_2	2.30 m 2.91 m	37 CH_2	2.18 m, 2.28 t (9.7)	40.8 CH_2	1.48 m, 2.25 m	36.3 CH_2
8	2.2 m	36.1 CH	2.37 m	41.7 CH	2.18 m	37.0 CH	2.27 m	34.9 CH
9	-	54.0 C_q		56.1 C_q	-	55.2 C_q		50.2 C_q
10	2.85 m	39.9 CH	1.91 m	39.2 CH	2.85 t (8.3)	41.4 CH		43.9 CH
11	2.56 dd (14.2, 8.7, 0.5 H), 2.57 dd (13.6, 8.5, 0.5 H), 2.73 dd (14.3, 8.7, 1.0 H)	40.2 CH_2	2.51 dd (13.9, 9.0), 2.74 dd (14.1, 8.2)	40.4 CH_2	2.56 dd (14.4, 8.5), 2.73 dd (14.3, 8.8)	40.7 CH_2	1.6 m, 2.25 m	41.3 CH_2
12	5.60 t (8.6, 0.5 H), 5.61 t (8.6, 0.5 H)	72.3 CH	5.53 dd (8.6, 7.6)	74.1 CH	5.60 t (8.6)	74.0 CH	4.6 m	64.0 CH
13	-	124.7 C_q	-	126.5 C_q	-	126.5 C_q	-	108.1 C_q
14	6.49 m	107.9 CH	6.52 m	109.2 CH	6.49 m	109.2 CH	7.17 m	146.6 CH
15	7.54 d (1.7, 0.5 H), 7.55 d (1.8, 0.5 H)	139.6 CH	7.56 m	145.7 CH	7.55 m	145.6 CH	5.88 m	99.0 CH
16	7.61 m	144.3 CH	7.65 m	141.8 CH	7.62 m	141.5 CH	-	172.2 C_q
17	1.00 d (6.4, 1.5 H), 1.01 d (6.4, 1.5 H)	16.4 CH_3	1.17 d (6.9)	16.8 CH_3	1.00 d (6.4)	16.9 CH_3	1.00 d (6.4)	16.5 CH_3
18	-	170.1 C_q	-	179 C_q		170.5 C_q	3.97 d (10.4), 4.35 d (10.4)	75.8 CH_2
19							4.58 d (12), 4.64 d (12)	68.2 CH_2
20	-	176.4 C_q	-	178.3 C_q	-	178.6 C_q	-	174.6 C_q
MeCO_2-6			2.05 s	20.9 CH_3				
Me*C*O_2-6				172.0 C_q				
MeO-6					3.19 s	51.0 CH_3		

Table S3. ^{1}H and ^{13}C NMR data for **8-11.**

Position	**8** (CD_3OD)		**9** (CD_3OD)		**10** (CD_3OD)		**11** ($CDCl_3$)	
	δ_H mult. (*J* Hz)	δ_C type	δ_H mult. (*J* Hz)	δ_C type	δ_H mult. (*J* Hz)	δ_C type	δ_H mult. (*J* Hz)	δ_C type
1	1.7 m, 2.1 m	21.9 CH_2	1.79 m, 2.15 m	22.4 CH_2	1.3 m, 2.39 m	22.7 CH_2	1.95 m, 1.99 m	23.2 CH_2
2	2.2 m, 1.4 m	35.1 or 35.2 CH_2	1.35 m, 2.10 m	34.4 CH_2	1.4 m, 2.03 m	32.8 CH_2	1.41 brdq (2.9, 9.3), 2.15 m	30.3 CH_2
3	4.00 m	66.5 CH	4.00 brdd (11.5, 4.0)	66.64 *or* 66.55 CH	3.83 dd (9.4, 4.6)	73.3 CH	3.90 d (7.5)	73.2 CH
4	-	66.9 C_q	-	70.6 C_q	-	85.6 C_q	-	83.9 C_q
5	-	44.5 C_q	-	44 C_q	-	50.2 C_q	-	50.8 C_q
6	3.78 dd (8.1, 7.7)	74.3 CH	3.78 dd (8.1, 7.7)	74.5 CH	-	108.1 C_q	-	110.9 C_q
7	-	30.8 CH_2	1.65 m, 1.75 m	38.1 CH_2	1.45 m, 2.2 m	41.2 CH_2	1.65 t (10.1), 2.36 dd (10.3, 3.9)	36.1 CH_2
8	1.7	35.1 *or* 35.2 CH	1.94 m	35.4 CH	2.28 m	36.3 CH	1.86 m	35.2 CH
9	-	48 C_q	-	43.5 C_q	-	36.2 C_q	-	44.6 C_q
10	1.7 m	47 CH	2.22 brd (14.9)	47.5 CH	2.23 m	43.9 CH	2.13 m	37.8 CH
11	1.4 m, 2.25 m	34.6 CH_2	1.75 m	38.0 CH_2	1.89 dd (12.2, 9.6), 2.43 m	34.2 CH_2	1.85 m, 2.24 brdd (9,3)	40.5 CH_2
12	2.15 m, 2.28 m	19.3 CH_2	4.56 brt (7)	63.8 CH_2	4.76 m	65.8 CH	5.10 m	71.6 CH
13	-	138.9 C_q	-	144.1 C_q	3.1 m	49.7 CH	-	126.7 C_q
14	7.02 d (2.2)	144.8 CH	7.13 brs	144.8 CH	14.2 m, 2.04 m	33 CH_2	6.41 m	108.6 CH
15	5.84 d (1.9)	104.6 CH	5.88 brs	104.6 CH	-	174.5 C_q	7.43 m	143.6 CH
16	-	173.3 C_q	-	171.7 C_q	-	173.1 C_q	7.43 m	139.3 CH
17	0.97 d (6.0)	16.5 CH_3	1.00 (6.7)	16.7 CH_3	0.94 br	16.4 CH_3	1.02 d (6.0)	16.3 CH_3
18	3.21 d (3.8), 3.01 d (3.8)	42 CH_2	2.98 d (4.2), 3.17 d (3.9)	43 CH_2	3.97 d (10.5), 4.34 d (10.5)	75.6 CH_2	3.89 d (7.5), 4.42 d (7.7)	75.1 CH_2
19	4.65 d (12.2), 4.43 d (12.2)	63.7 CH_2	4.12 d (13.5), 4.71 d (13.5)	63.9 CH_2	4.59 d (12.3), 4.65 d (12.3)	68.5 CH_2	3.94 d (9.1), 4.15 d (9.1)	58.5 CH_2
20	3.97 d (12.0), 4.03 d (12.0)	66.5 CH_2	3.89 d (13.5, 0.5 H), 3.90 d (13.5, 0.5 H), 3.94 d (13.5, 0.5 H), 3.95 d (13.5, 0.5 H)	66.64 or 66.55 CH_2	-	174.5 C_q	5.08 s	101.1 CH
MeCO_2-19	2.06 s	20.98 *or* 21.03 CH_3	2.02 s	21.0 CH_3				
Me*C*O_2-19		172.7 C_q	-	172.8 C_q				
MeCO_2-20	2.06 s	20.98 *or* 21.03 CH_3	2.01 s	21.0 CH_3				
Me*C*O_2-20		172.7 C_q	-	172.5 C_q				
MeO-6							3.37 s	48.1 CH_3
MeO-15	3.52 s	57.3 CH_3	3.54 s (1.5 H), 3.55 s (1.5 H)	57.4 CH_3				

Table S4. ^{1}H and ^{13}C NMR data for **12-14.**

	**12** (CD_3OD)		**13** ($(CD_3)_2SO$)		**14** ($(CD_3)_2SO$)		**14** ($CDCl_3$)	
**Position**	**δ_H mult. (*J* Hz)**	**δ_C type**	**δ_H mult. (*J* Hz)**	**δ_C type**	**δ_H mult. (*J* Hz)**	**δ_C type**	**δ_H mult. (*J* Hz)**	**δ_C type**
1	1.78 m, 2.12 m	23.1 CH_2	1.86 m, 1.98 m	24.3 CH_2	1.75 m, 2.05 m	21.5 CH_2	1.92 brd (10.5), 2.15 m	40.1 CH_2
2	1.45 m, 2.22 m	34.1 CH_2	1.27 m, 1.87 m	31.6 CH_2	1.17 dd (11, 4), 1.91 m	43.5 CH_2	1.31 dq (4.5, 12.0), 2.18 m	32.9 CH_2
3	4.28 m	68.6 CH	-	57.7 C_q	3.80 brd (11)	65.0 CH	4.02 dd (12.0, 4.5)	66.0 CH
4	-	52.8 C_q	3.66 dd (11, 4), 3.27 dd (11, 5)	62.5 CH_2	-	70.0 C_q	-	nd
5	-	48.0 C_q	-	59.3 C_q	-	45.5 C_q	-	nd
6	4.99 m	74.7 CH	3.36 dd (11, 4)	69.4 CH	3.62 brd (11)	73.3 CH	3.70 dd (10.0, 6.0)	73.6 CH
7	1.56 m, 2.22 m	33.5 CH_2	1.21 q (11), 1.47 m	37.9 CH_2	1.39 brd (12), 1.52 brq (12)	34.7 CH_2	1.65 m	34.0 CH_2
8	1.78 m	38.7 CH	1.47 m	35.6 CH	1.68 m	34 CH	1.73 m	35.6 CH
9	-	52.0 C_q	-	40.4 C_q	-	43.3 C_q	-	nd
10	1.89 m	51.8 CH	2.07 dd (12, 5)	51.5 CH	1.96 brd (11)	46.8 CH	2.02 dd (13.0, 2.5)	48.3 CH
11	2.38 m, 2.51 m	43.6 CH_2	1.60 dd (16, 4), 2.01 dd 16, 9)	41.7 CH_2	1.67 m, 1.86 m	39.7 CH_2	1.80 dd (16.0, 2.0), 2.17 m	32.9 CH_2
12	5.48 m	73.5 CH	4.68 m	62.1 CH	4.59 m	61.7 CH	4.86 brd (8.5)	63.3 CH
13	-	126.7 C_q	-	132.4 C_q	-	132.8 C_q	-	nd
14	6.46 m	109.17 CH	6.46 m	109.7 CH	6.42 m	109.6 CH	6.41 m	108 CH
15	7.53 m	145.6 CH	7.56 m	143.5 CH	7.56 m	143.6 CH	7.39 m	138 CH
16	7.59 m	141.4 CH	7.50 m	143.5 CH	7.49 m	138.6 CH	7.39 m	138 CH
17	0.99 m	16.9 CH_3	0.71 d (7)	16.3 CH_3	0.82 d (6)	17.0 CH_3	0.93 d (6.5)	17.0 CH_3
18	2.72 br, 2.82 br	42.5 CH_2	4.76 d (7)	102.0 CH	2.83 m, 3.06 m	43.0 CH_2	3.03 d (4.0), 3.13 d (3.5)	43.5 CH_2
19	3.90 d (9.4), 4.62 d (9.3)	61.4 CH_2	3.80 d (9), 3.87 d (9)	67.3 CH	4.40 d (12), 4.57 d (12)	62.4 CH_2	4.48 d (12.0), 4.68 d (12.0)	62.5 CH_2
20	-	178.9 C_q	3.21 dd (11, 5), 3.32 dd (11, 5)	63.3 CH_2	3.28 m	63.0 CH_2	3.59 s (2 H)	64.3 CH_2
MeCO_2-6	2.04 s	21.3 CH_3						
Me*C*O_2-6		171.6 C_q						
HO-3			4.68 br	-		nd		
HO-4	4.46 m	-						
HO-6	4.46 m	-	3.28 m	-		nd		
HO-12	5.02 d (5)	-	4.90 br	-		nd		
HO-18	6.26 d (7)	-						
HO-20	4.46 m	-	4.90 br	-		nd		

Table S5. ^{1}H and ^{13}C NMR data for **15,16.** nd = not observed

Position	**15** ($(CD_3)_2SO$)		**15** ($CDCl_3$)		**16** ($(CD_3)_2SO$)		**16** ($CDCl_3$)	
	**δH mult. (*J* Hz)**	**δC type**	**δH mult. (*J* Hz)**	**δC type**	**δH mult. (*J* Hz)**	**δC type**	**δH mult. (*J* Hz)**	**δC type**
1	1.59 brq (12), 1.96 m	22.0 CH_2	1.83 dq (4.5, 13.0), 1.95 m	22.1 CH_2	1.43 m, 1.68 brd (12)	21.5 CH_2	1.70 m (2 H)	21.5 CH_2
2	1.30 dq (4, 12), 2.05 m	34.1 CH_2	1.40 dq (5.0, 12.5), 2.30 m	32.5 CH_2	1.20 dq (11, 4), 2.00 m	34.1 CH_2	1.30 dd (14.0, 8.0), 2.26 m	32.2 CH_2
3	4.19 m	64.3 CH	4.43 ddd (13.0, 5.0, 2.0)	64.8 CH	4.16 t (10,4)	64.5 CH	4.47 ddd (10.0, 5.0, 3.5)	65.1 CH
4	-	68.8 C_q	-	nd	-	68.8 C_q	-	nd
5	-	46.4 C_q	-	nd		46.2 C_q	-	nd
6	3.67 m	72.5 CH	3.73 dd (12.0, 4.0)	73.9 CH	3.65 m	72.5 CH	3.75 dd (13.5, 3.5)	74.3 CH
7	1.48 brd (12), 1.96 m	34.9 CH_2	1.69 dt (13.0, 4.0), 2.17 q (12.5)	33.4 CH_2	1.43 m, 1.74 m	35.6 CH_2	1.65 dt (13.0, 3.5), 2.08 brq (12.0)	33.6 CH_2
8	1.62 m	37.3 CH	1.58 m	38.3 CH	1.74 m	40.0 CH	1.70 m	41.1 CH
9	-	51.5 C_q	-	nd	-	51.5 C_q	-	nd
10	1.67 brd (12)	51.2 CH	1.60 dd (13.0, 3.0)	52.1 CH	1.50 brd (12)	49.2 CH	1.45 dd (12.0, 4.0)	49.8 CH
11	2.25 dd (10, 7), 2.45 dd (10, 7)	41.2 CH_2	2.34 d (8.5)	nd	2.33 dd (11, 7), 2.41 dd (11, 7)	42.6 CH_2	2.32 dd (14.0, 8.0), 2.42 m	43.8 CH_2
12	5.43 t (7)	71.4 CH	5.34 t (8.5)	71.5 CH	5.42 t (7)	71.3 CH	5.36 t (8.5)	71.3 CH
13	-	125.6 C_q	-	nd	-	125.8 C_q	-	nd
14	6.49 m	109.1 CH	6.36 m	107 CH	6.51 m	109.3 CH	6.35 m	107.5 CH
15	7.74 m	145.0 CH	7.42 m	143 CH	7.71 m	145.0 CH	7.45 m	139 CH
16	7.78 m	141.1 CH	7.42 m	139 CH	7.77 m	140.7 CH	7.43 m	139 m
17	0.91 d (6)	16.9 CH_3	1.01 d (7.0)	16.5 CH_3	1.00 d (6)	17.3 CH_3	1.13 d (7.0)	17.0 CH_3
18	2.69 d (4), 2.89 d (4)	40.6 CH_2	2.99 d (4.0), 3.01 d (4.0)	42.2 CH_2	2.65 d (4), 2.83 d (4)	40.9 CH_2	2.97 d (4.0), 3.00 d (3.5)	42.4 CH_2
19	3.69 m, 4.36 m	59.3 CH_2	3.88 dd (12.5, 10.0), 4.73 (12.5, 3.5)	60.7 CH_2	3.76 dd (11, 8), 4.40 dd (11, 2)	60.1 CH_2	4.00 dd (13.5, 11.0), 4.76 dd (13.5, 4.0)	61.5 CH_2
20	-	177.0 C_q	-	nd	-	176.8 C_q	-	nd
HO-3	4.66 d (4)		2.82 br		4.60 d (4)		2.12 d (5.0)	
HO-6	3.99 br				4.03 brd (7)		nd	
HO-19	4.07 m		2.69 dd (10.0, 3.5)		4.10 brd (8)		2.42 m	

Table S6. ^{1}H and ^{13}C NMR data for **17,18.** nd = not observed

	**17** ($(CD_3)_2SO$)		**17** ($CDCl_3$)		**18** ($(CD_3)_2SO$)		**18** ($CDCl_3$)
**Position**	**δ_H mult. (*J* Hz)**	**δ_C type**	**δ_H mult. (*J* Hz)**	**δ_C type**	**δ_H mult. (*J* Hz)**	**δ_C type**	**δ_H mult. (*J* Hz)**
1	1.95 m, 2.0 m	22.6 CH_2	1.91 m, 2.12 m	22 CH_2	1.25 brq (12), 2.27 m	71.6 CH_2	1.13 m, 2.22 m
2	1.80 m, 2.45 m	36 CH_2	1.50 dd (12.5, 5.0)	28 CH_2	1.39 dq (3, 12)	33.0 CH_2	1.4 m
3	5.45 m	71.4 CH	5.54 dd (12.5, 5.0)	55 CH	4.06 m	51.5 CH	4.44 m
4	-	76.9 C_q	-	nd	-	139.5 C_q	-
5	-	48.2 C_q	-	nd	-	137.4 C_q	-
6	4.20 brd (11)	72.8 CH	4.21 brd (11.0)	54 CH	-	109.16 C_q	-
7	1.54 m, 1.87 brq (11)	36.1 CH_2	1.70 m, 2.20 brq (12.5)	35 CH_2	1.82 dd (13, 3), 2.27 t (13)	39.6 CH_2	1.99 dd (13.5, 4.0), 2.22 m
8	1.70 m	37.7 CH	1.70 m	39 CH	2.01 m	35.8 CH	2.09 m
9	-	51.7 C_q	-	51 C_q	-	53.0 C_q	-
10	1.80 m	42.6 CH	1.87 m	49 CH	2.45 m	40 CH	2.48 m
11	2.32 dd (12, 9), 2.45 m	43.1 CH_2	2.40 d (8.5)	44 CH_2	2.42 dd (12, 8), 2.57 dd (12, 8)	40 CH_2	2.48 m
12	5.45 m	74.0 CH	5.35 t (8.5)	71 CH	5.48 t (8)	71.6 CH	5.38 t (8.5)
13	-	125.7 C_q	-	124 C_q	-	125.4 C_q	-
14	6.51 m	109.1 CH	6.38 m	109 CH	6.52 m	109.20 CH	6.39 m
15	7.71 m	144.9 CH	7.44 m	143 CH	7.72 t (3)	145.0	7.45 m
16	7.79 m	141.2 CH	7.44 m	139 CH	7.82 m	141.2	7.45 m
17	0.96 d (6)	16.8 CH_3	1.06 d (7.5)	17 CH_3	0.92 d (7)	17.2 CH_3	0.99 d (6.5)
18	3.43 dd (10, 3),<br>4.04 dd (10, 5)	61.1 CH_2	4.04 brd (12.0),<br>4.09 brd (12.0)	60.5 CH_2	6.02 s	107.0 CH	6.21 s
19	4.77 d (12), 4.86 d (12)	63.9 (CH_2)	5.06 d (13.0), 5.08 d (13.0)	63 CH_2			
20	-	177.1 C_q	-	nd	-	176.6 C_q	-
MeCO_2-3	2.05 s	21.6 CH_3	2.11 s	21 CH_3			
Me*C*O_2-3	-	170.4 C_q	-	170 C_q			
MeCO_2-19	2.00 s	21.8 CH_3	2.10 s	21 CH_3			
Me*C*O_2-19	-	169.9 C_q	-	170 C_q			
HO-3					5.06 d (7)		nd
HO-4	4.39 br		nd				
HO-6	5.06 m		nd				
HO-18	5.06 m		nd				
MeO-6					2.97 s	49.0 CH_3	3.11 s
MeO-18					3.15 s	51.5 CH_3	3.39 s

Table S7. ^{1}H and ^{13}C NMR data for **19,20.**

Position	**19** ($(CD_3)_2SO$)		**20** ($(CD_3)_2SO$)	
	**δ_H mult. (*J* Hz)**	**δ_C type**	**δ_H mult. (*J* Hz)**	**δ_C type**
1	1.50 m, 1.66 dq (4,11)	20.0 CH_2	1.49 m, 1.65 dq (4,11)	20.0 CH_2
2	1.24 m, 1.55 m	34.42 CH_2	1.20 dq (4,11), 1.51 m	34.1 CH_2
3	3.82 m	64.5 CH	4.10 m	64.7 CH
4	-	69.6 C_q	-	69.4 C_q
5	-	45.2 C_q	-	46.9 C_q
6	3.63 dd, 12, 3)	72.7 CH	3.55 dd (12, 6)	72.8 CH
7	1.43 m, 1.50 m	34.41 CH_2	1.45 m, 1.51 m	34.1 CH_2
8	1.24 m	34.1 CH	1.44 m	34.2 CH
9	-	38.6 C_q	-	38.5 C_q
10	1.38 m	46.3 CH	1.25 dd (11, 3)	46.5 CH
11	1.50 m, 1.98 m	34.38 CH_2	1.45 m, 1.97 m	34.2 CH_2
12	2.15 ddd (11, 9, 4), 2.25 ddd (11, 9, 3)	21.9 CH_2	2.12 ddd (11, 9, 4), 2.25 ddd (11, 9, 3)	22.0 CH_2
13	-	174.4 C_q	-	174.4 C_q
14	5.96 br	114.0 CH	5.95 s	113.9 CH
15	-	173.4 C_q	-	173.5 C_q
16	4.86 s	73.5 CH_2	4.86 s	73.5 CH_2
17	0.78 d (7)	15.8 CH_3	0.77 d (7)	15.9 CH_3
18	2.84 d (4), 3.09 d (4)	43.0 CH_2	2.70 d (4), 2.89 d (4)	40.5 CH_2
19	4.34 d (11), 4.49 d (11)	62.4 CH_2	3.78 dd (11, 9), 3.98 dd (11, 3)	60.3 CH_2
20	0.65 s	17.6 CH_3	0.62 s	17.7 CH_3
MeCO_2-19	2.02 s	21.5 CH_3		
Me*C*O_2-19	-	170.8 C_q		
HO-3	4.76 d (5)	-	4.56 d (4)	-
HO-6	nd	-	3.82 m	-
HO-19	-	-	4.12 dd (9, 3)	-

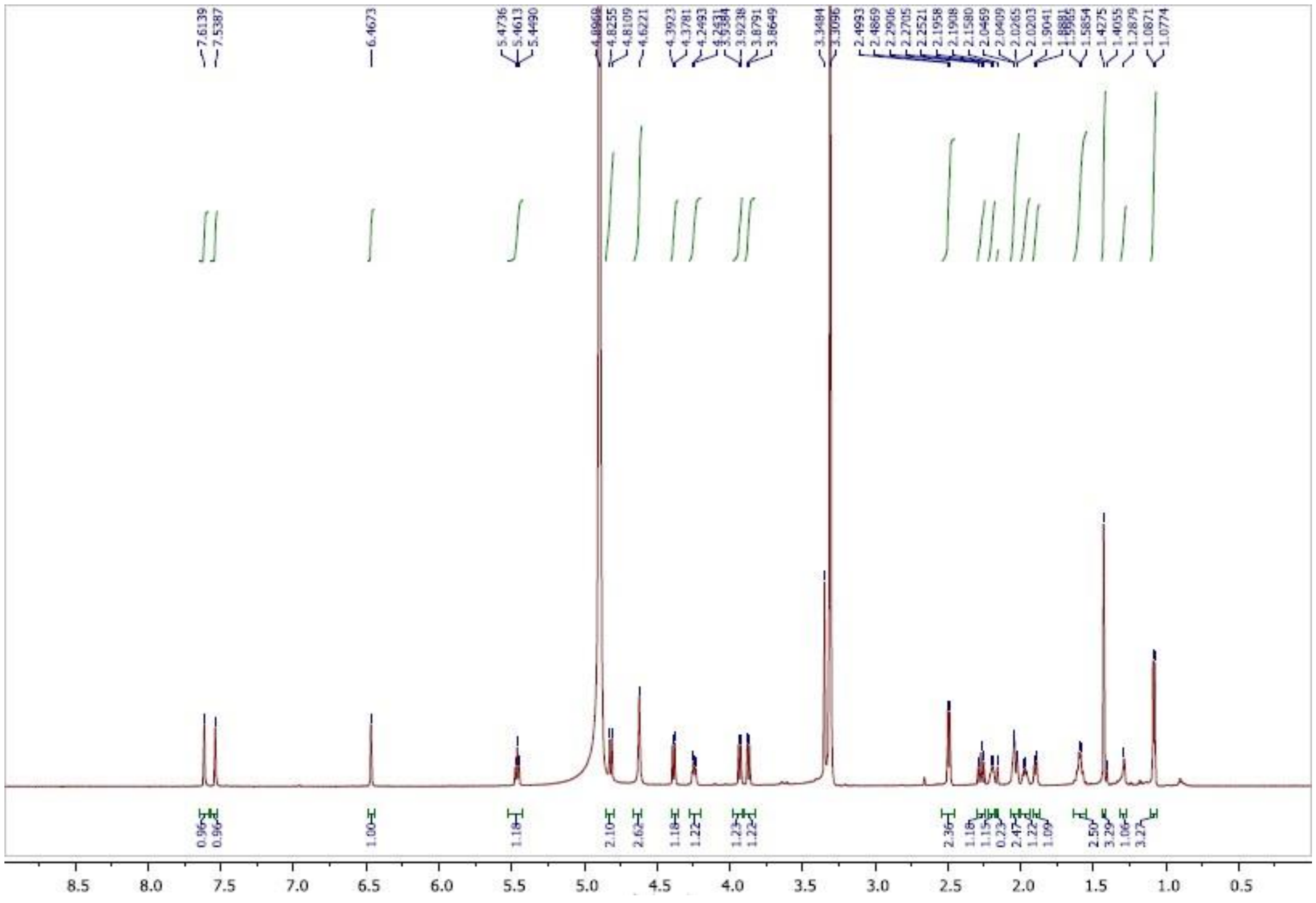

Figure S1. ^{1}H NMR spectrum of **1** in CD_3OD.

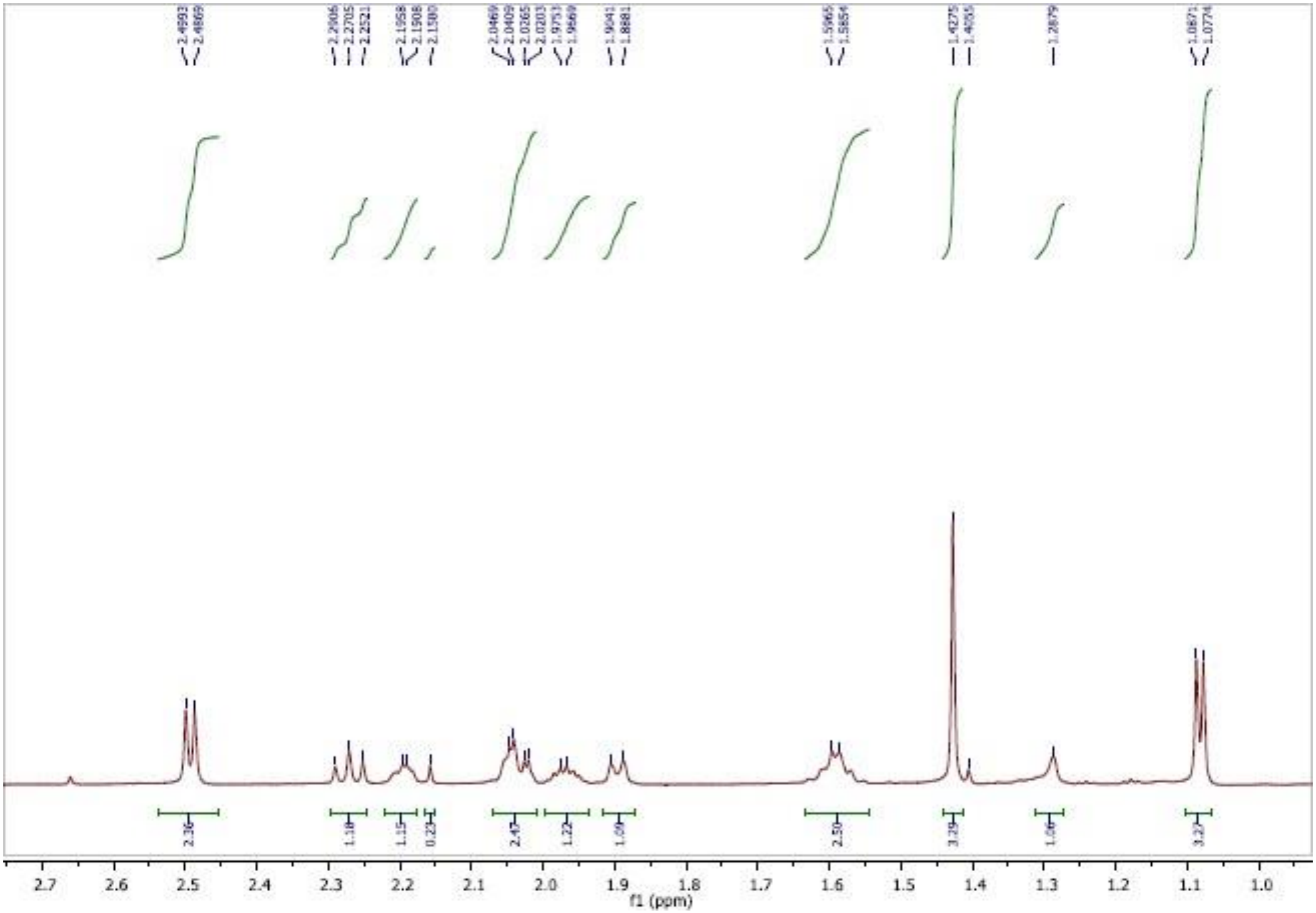

Figure S2. Expansion of part of ^{1}H NMR spectrum (δ 1.0 – δ 2.7) of **1** in CD_3OD.

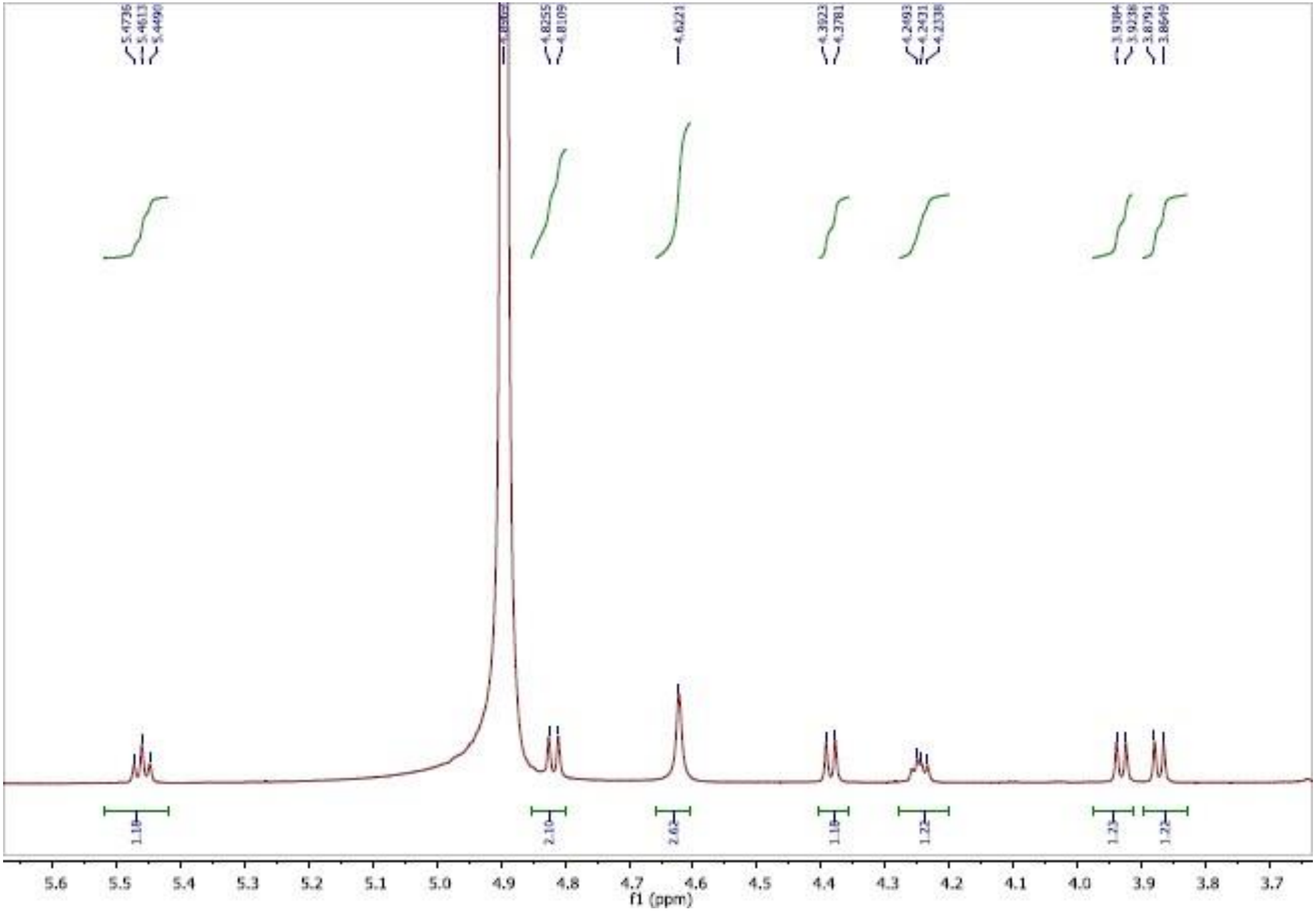

Figure S3. Expansion of part of ^{1}H NMR spectrum (δ 3.7 – δ 5.6) of **1** in CD_3OD.

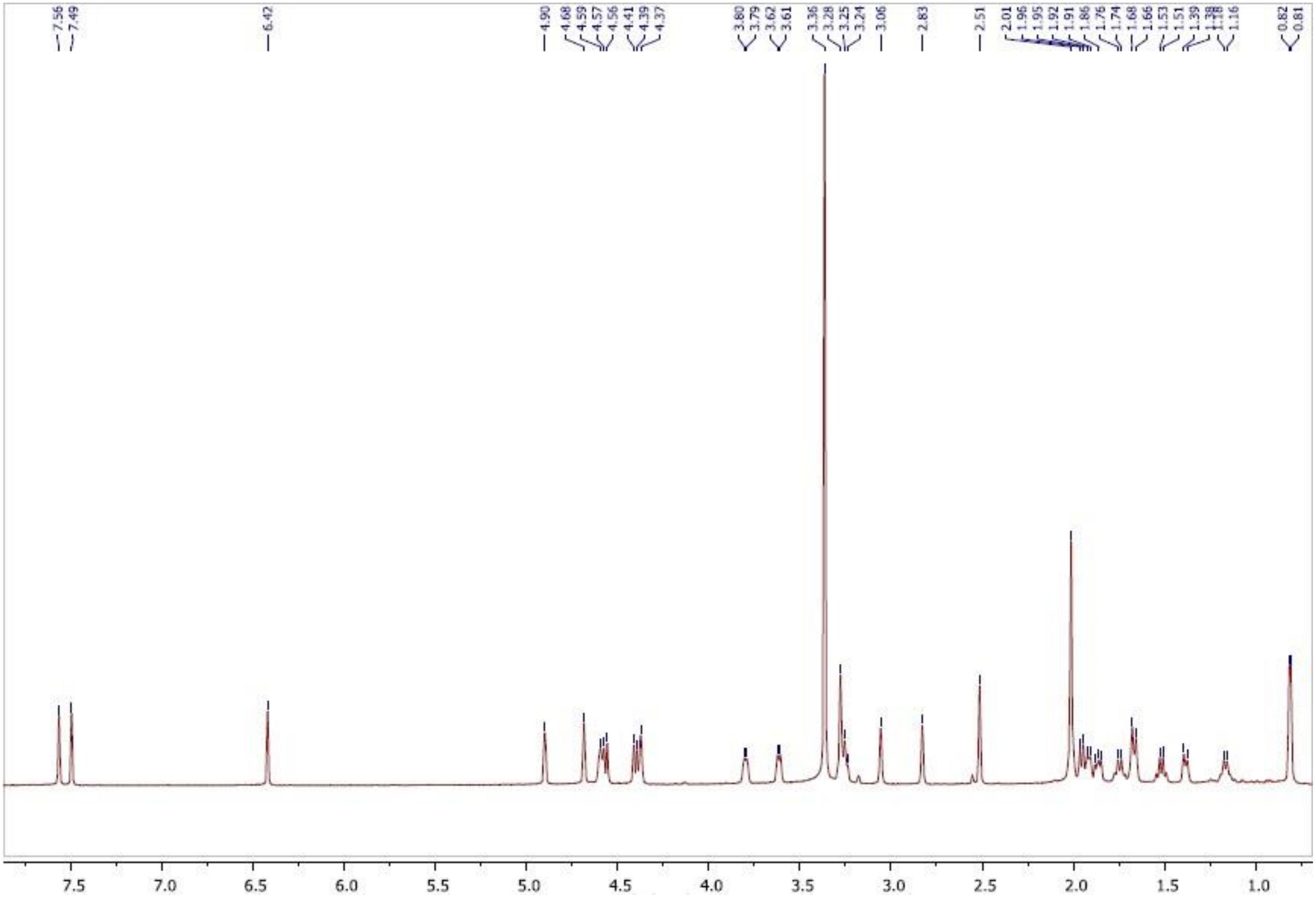

Figure S4. ^{1}H NMR spectrum of **1** in $(CD_3)_2SO$.

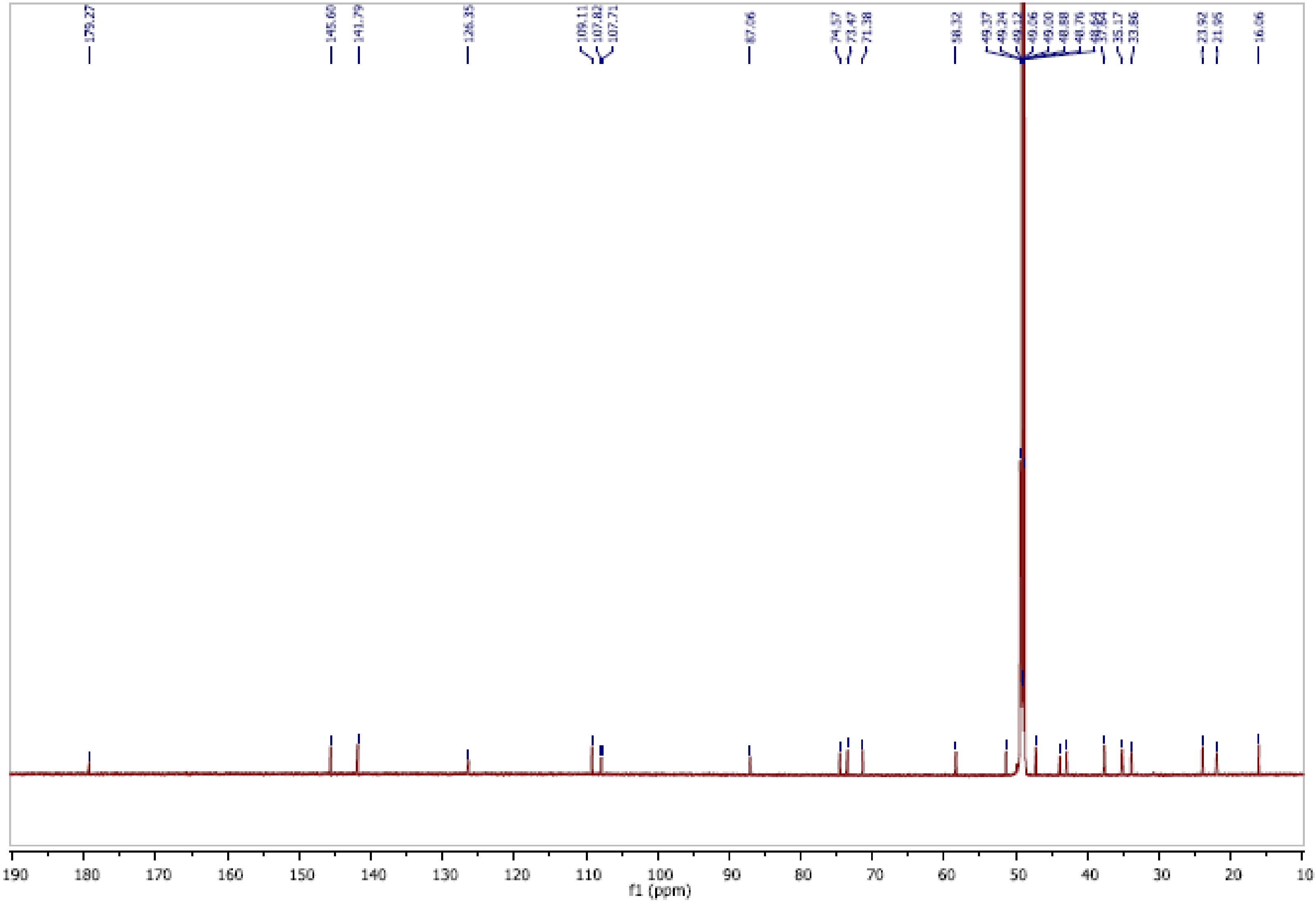

Figure S5. ^{13}C NMR spectrum of **1** in CD_3OD.

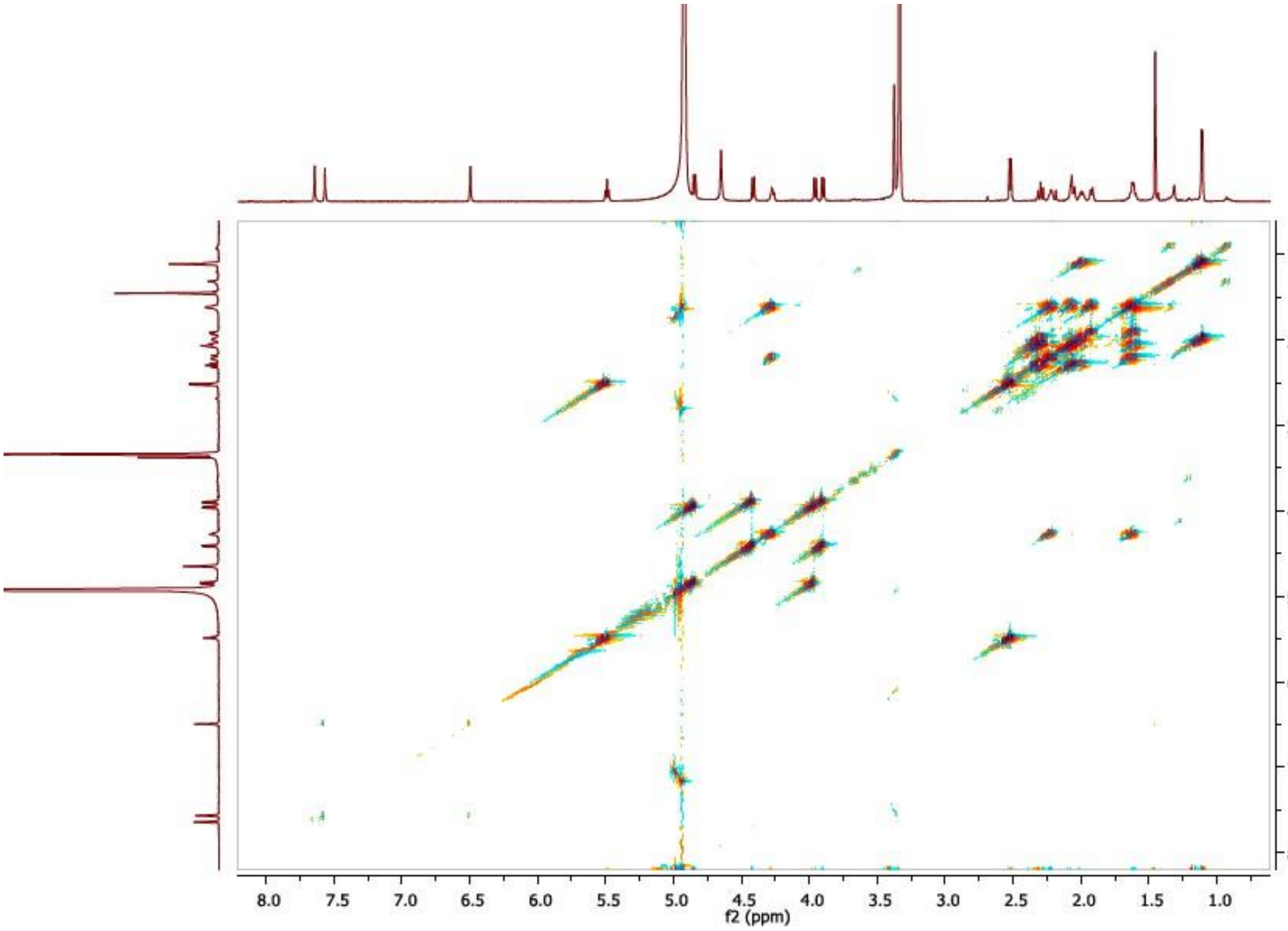

Figure S6. COSY NMR spectrum of **1** in CD_3OD.

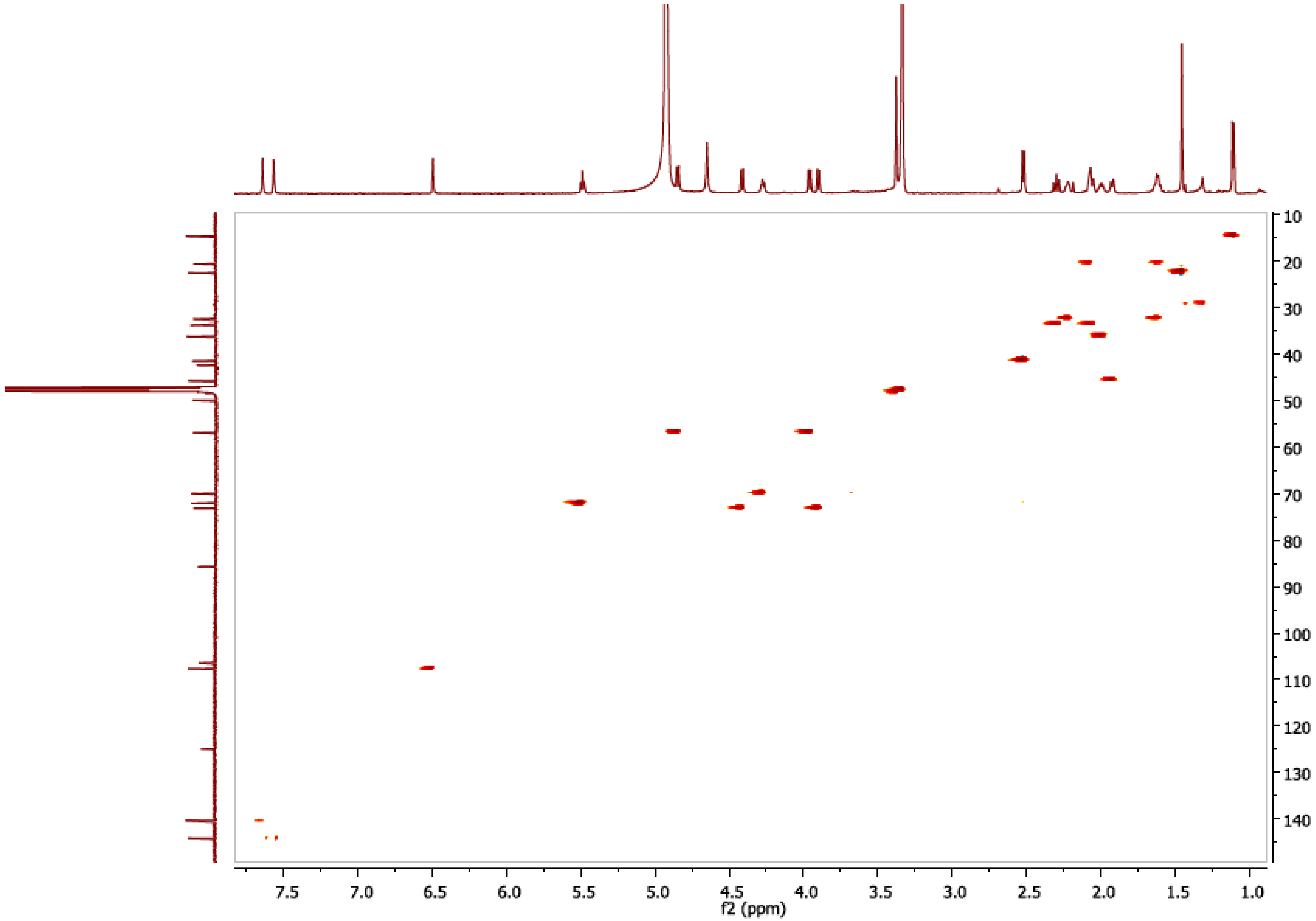

Figure S7. HSQC NMR spectrum of **1** in CD_3OD.

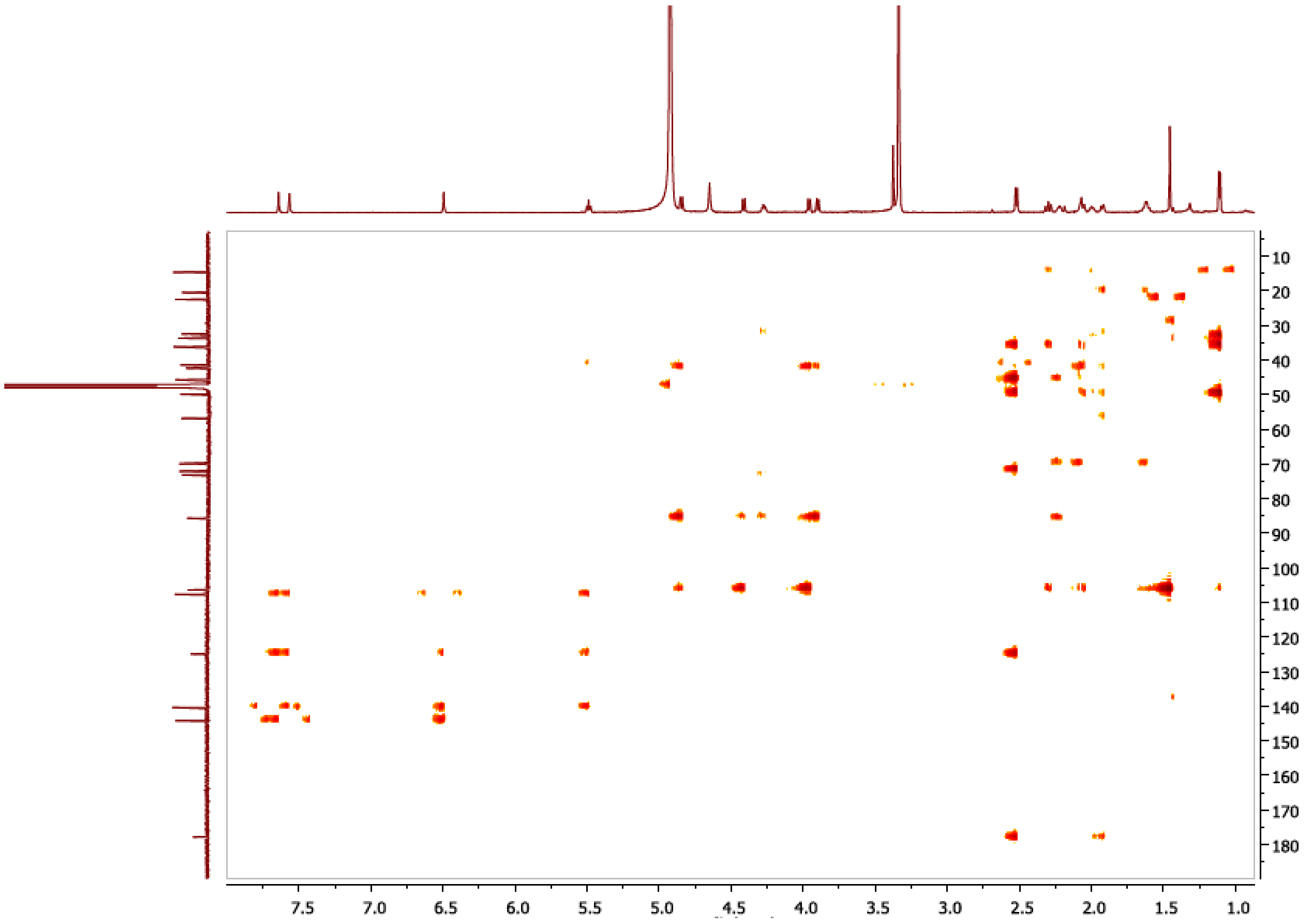

Figure S8. HMBC NMR spectrum of **1** in CD_3OD.

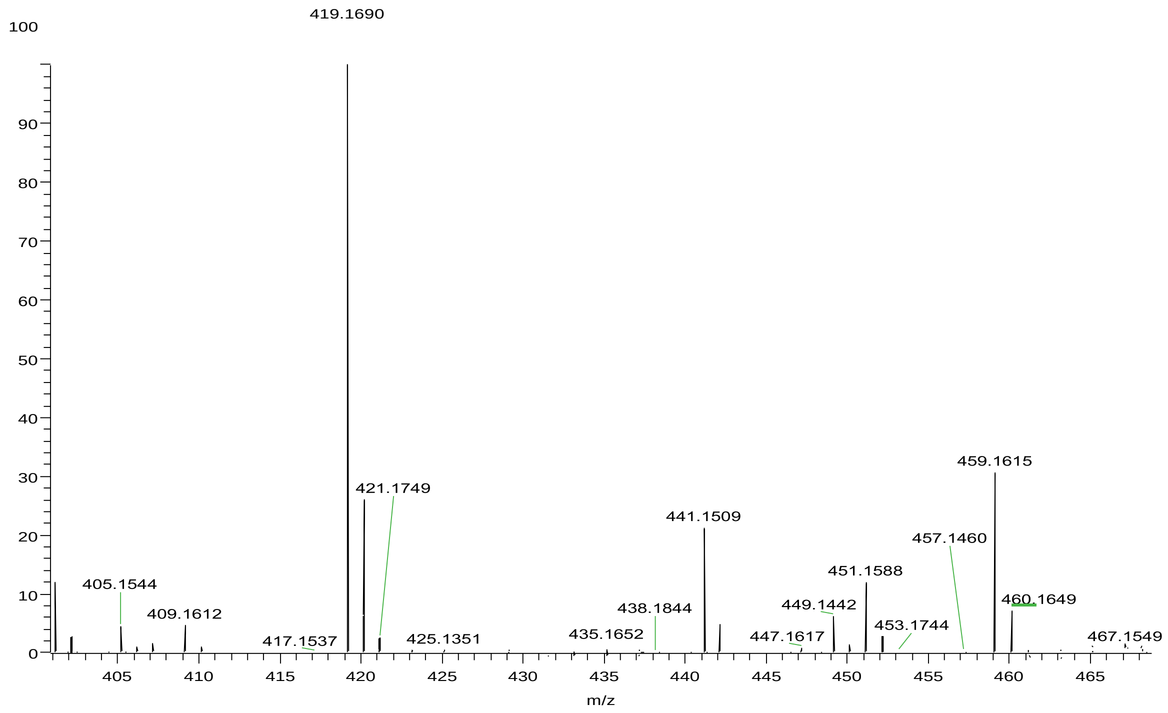

100
419.1690
90
80
70
60
50
40
30
20
10
0
459.1615
421.1749
441.1509
457.1460
451.1588
405.1544
460.1649
449.1442
438.1844
409.1612
453.1744
467.1549
417.1537
425.1351
435.1652
447.1617
405
410
415
420
425
430
435
440
445
450
455
460
465
m/z

Figure S9. Positive-ion HRESIMS of **1**.

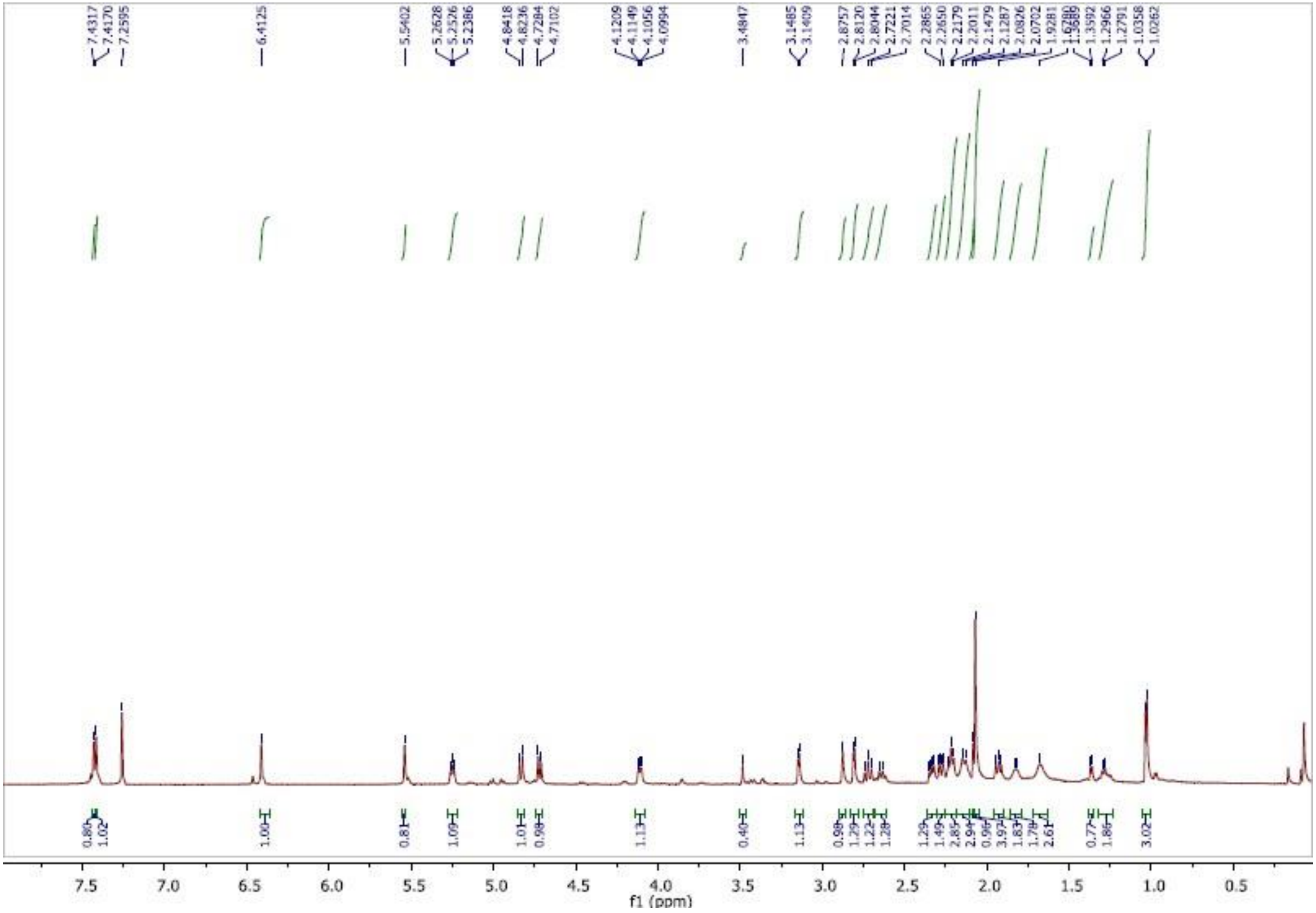

Figure S10. ^{1}H NMR spectrum of **2** in CD_3OD.

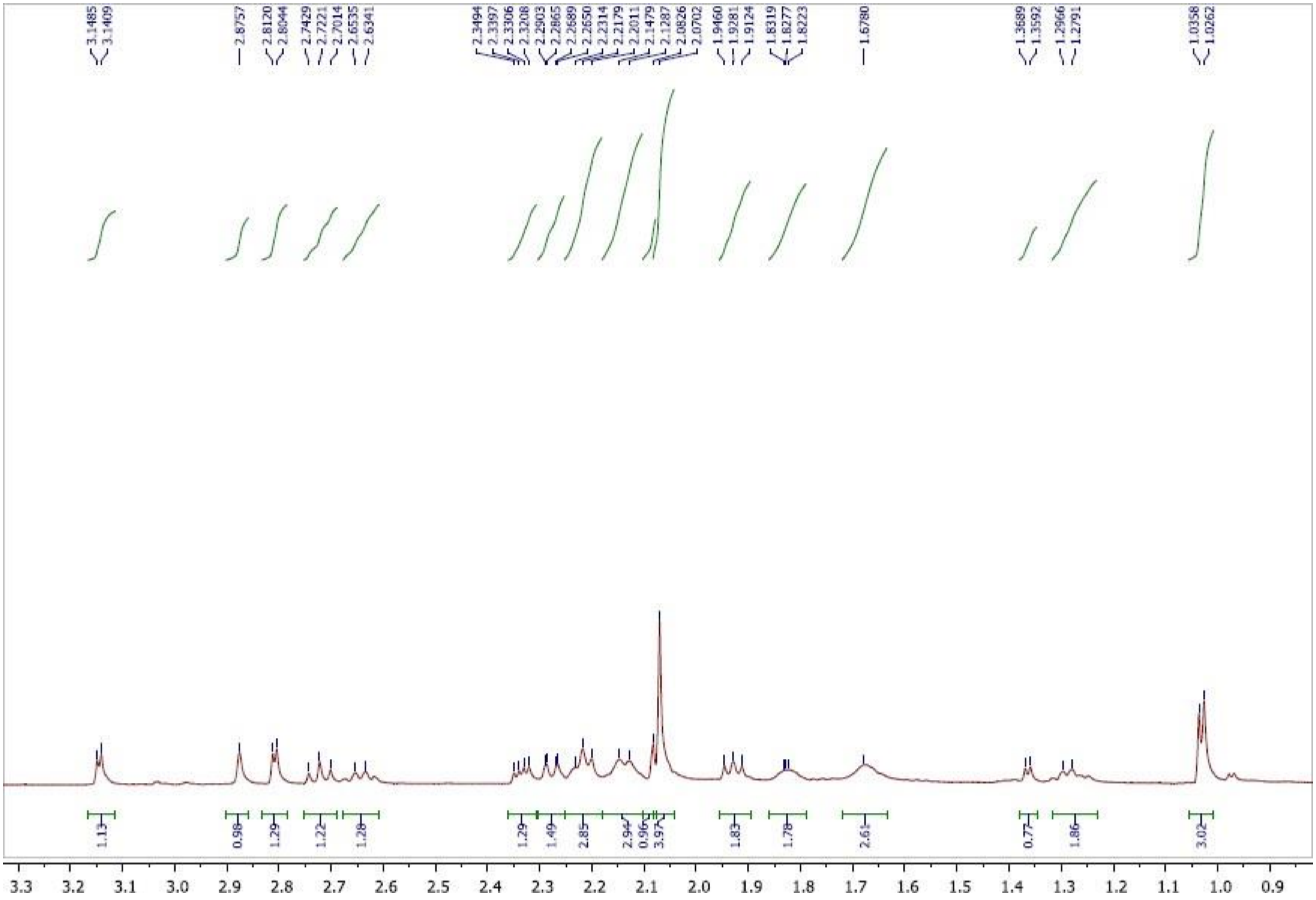

Figure S11. Expansion of part of ^{1}H NMR spectrum (δ 0.9 – δ 3.3) of **2** in CD_3OD.

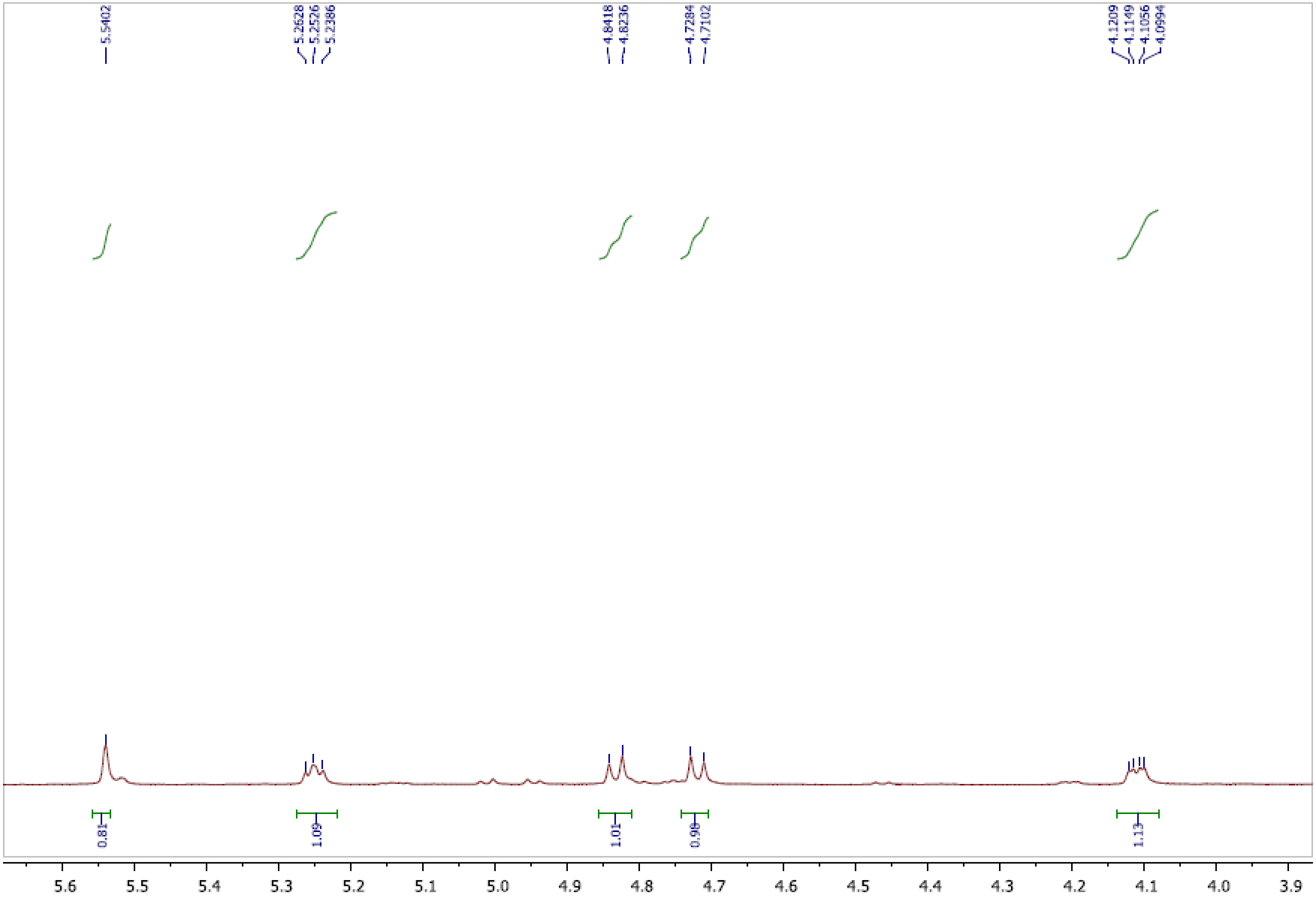

Figure S12. Expansion of part of ^{1}H NMR spectrum (δ 3.9 – δ 5.6) of **2** in CD_3OD.

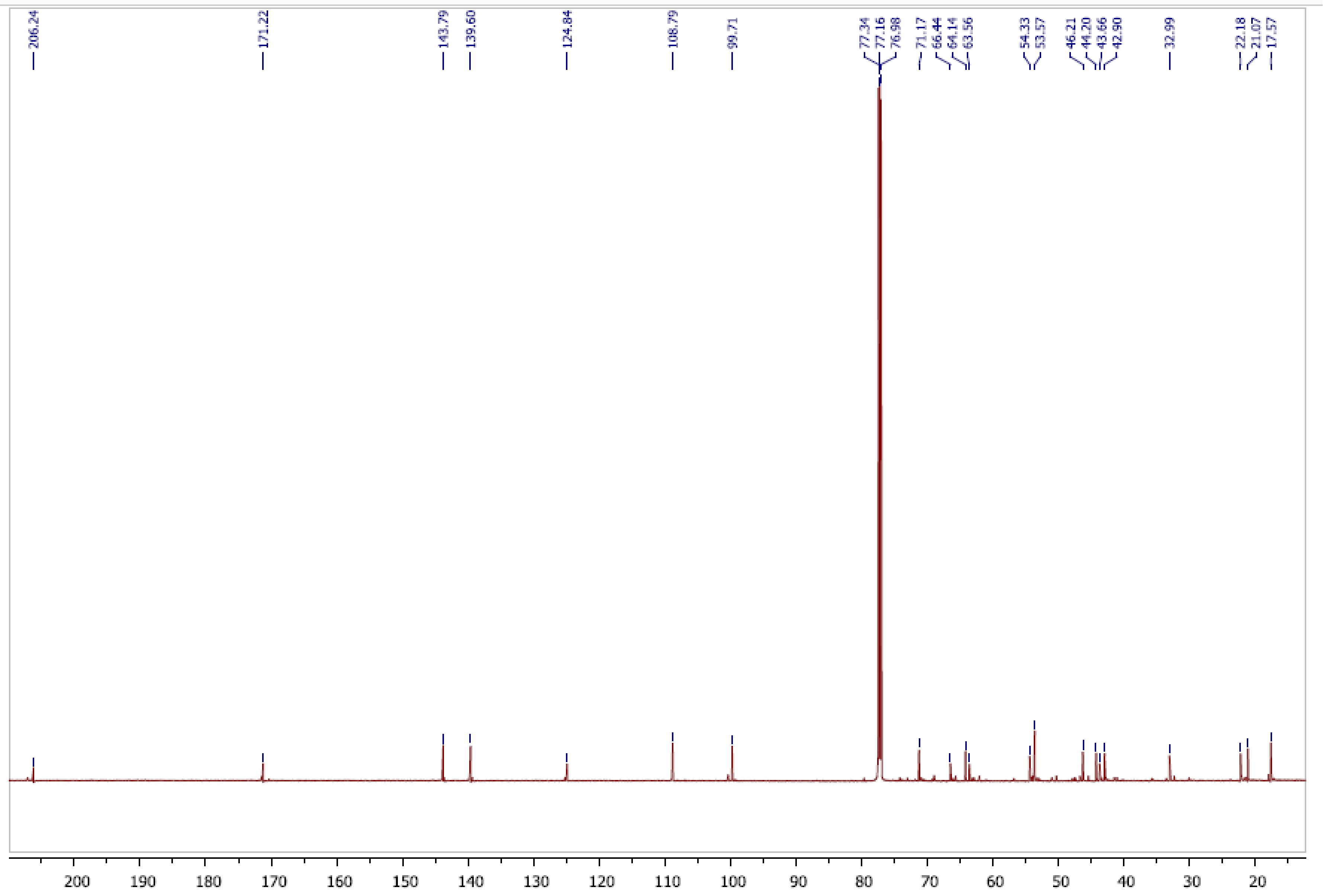

Figure S13. ^{13}C NMR spectrum of **2** in CD_3OD.

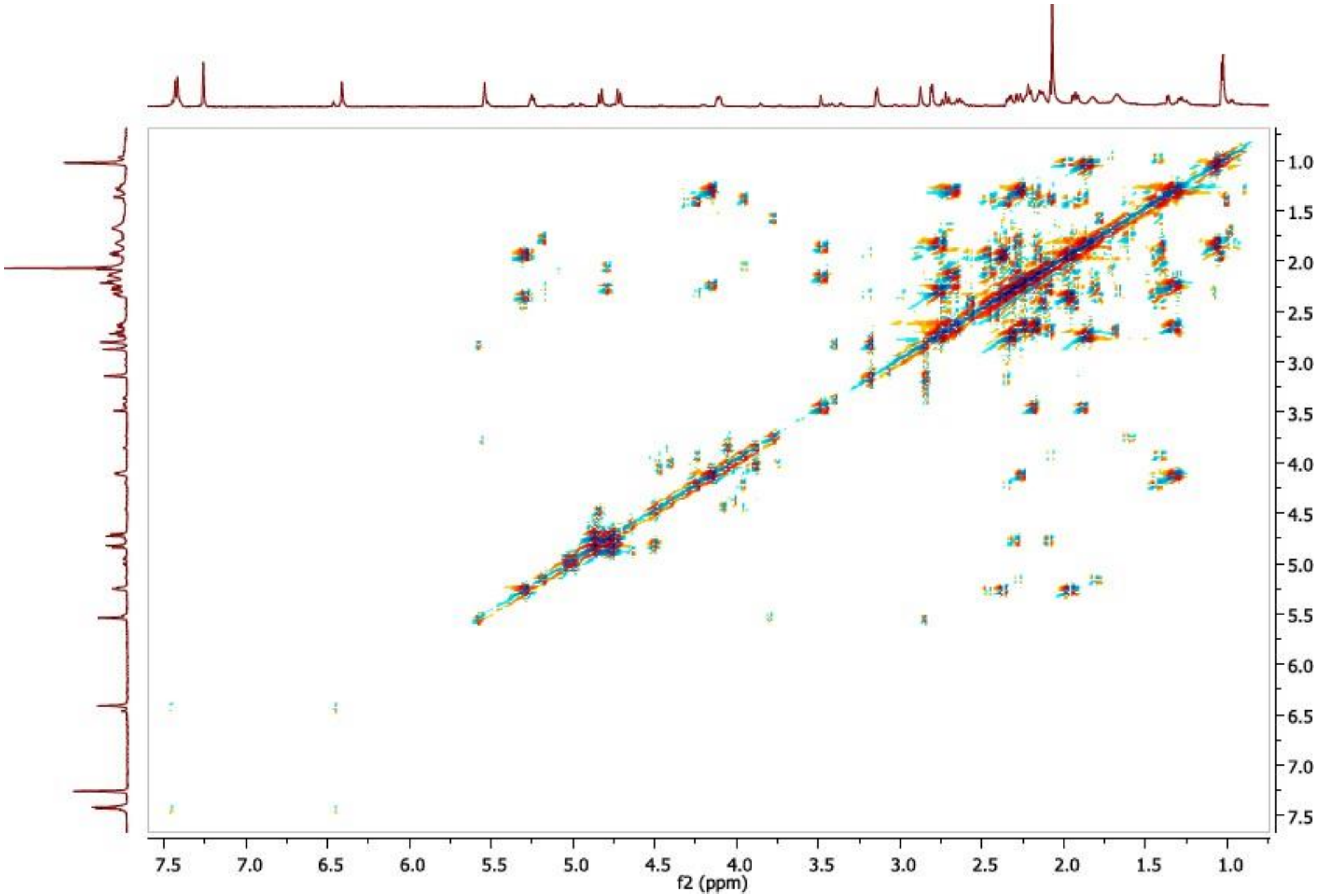

Figure S14. COSY NMR spectrum of **2** in CD_3OD.

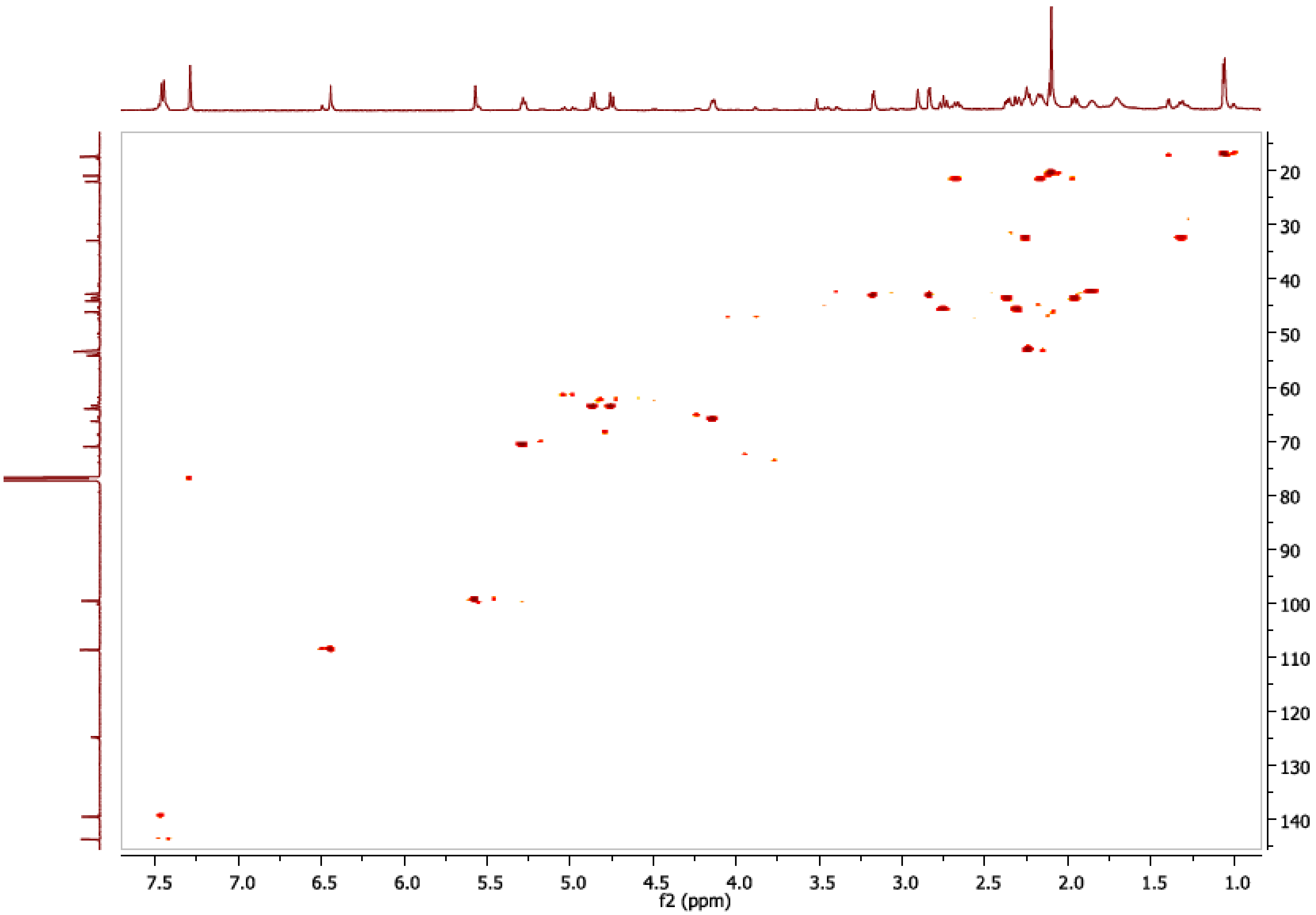

Figure S15. HSQC NMR spectrum of **2** in CD_3OD.

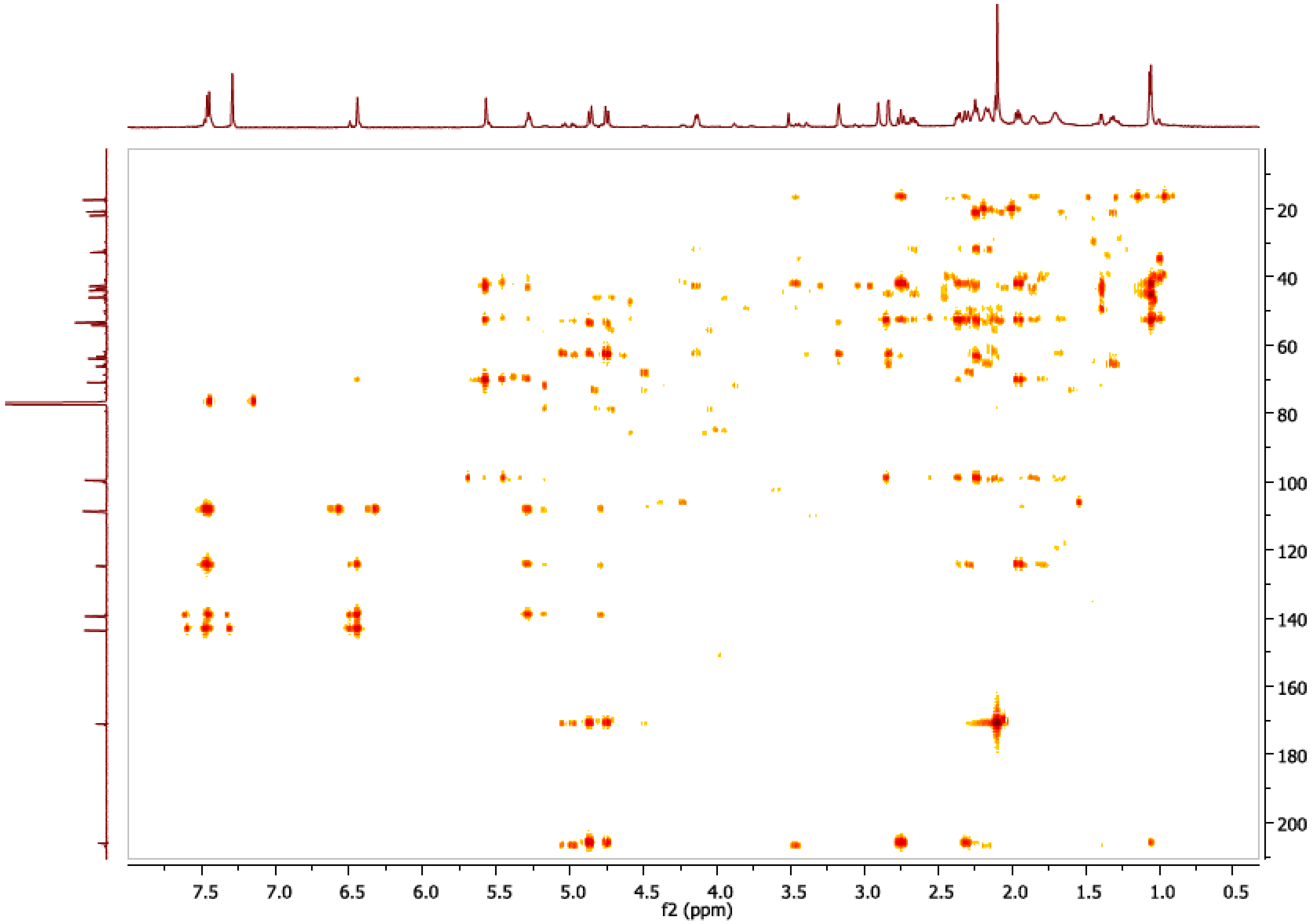

Figure S16. HMBC NMR spectrum of **2** in CD_3OD.

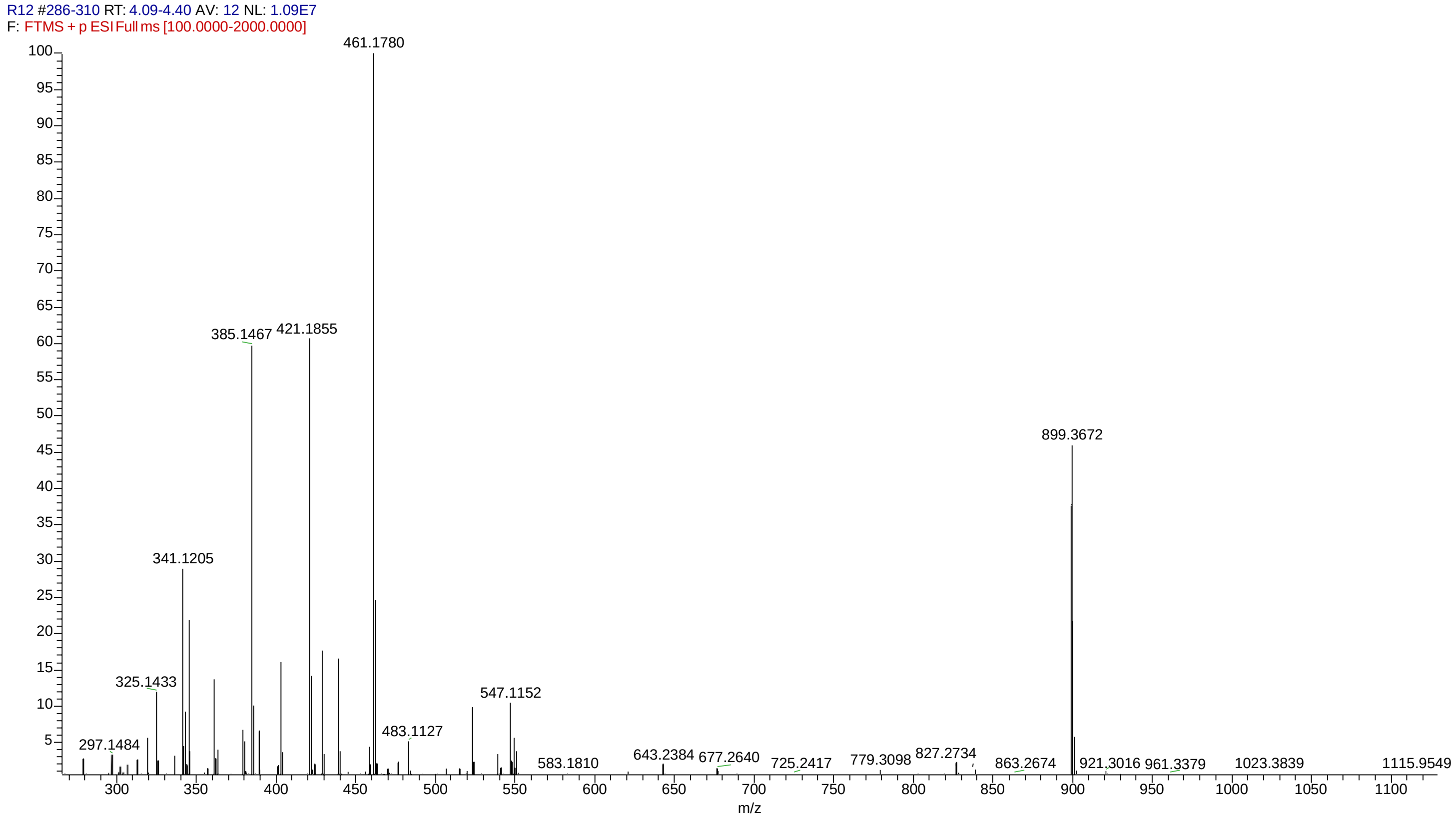

Figure S17. Positive-ion HRESIMS of **2**.

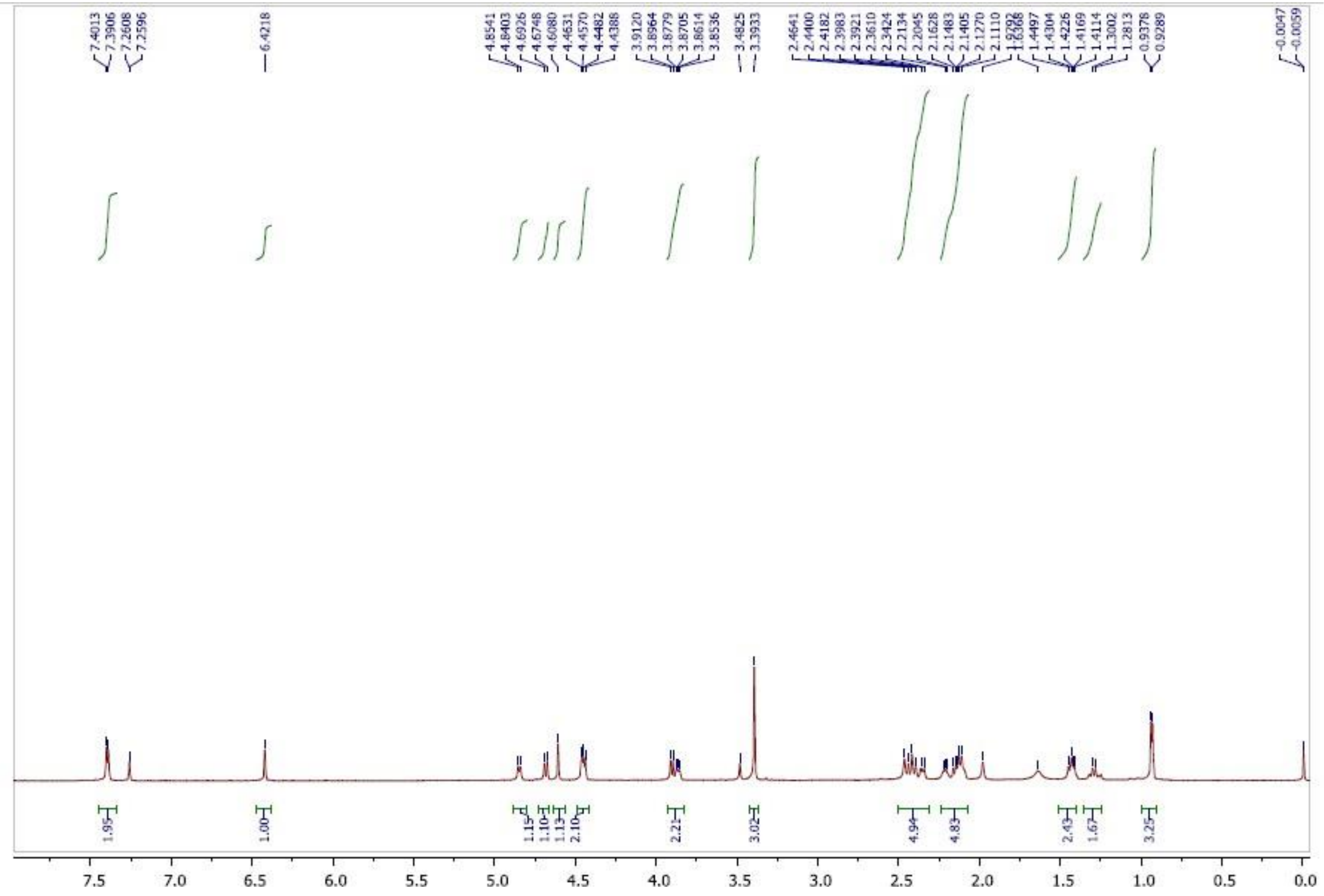

Figure S18. ^{1}H NMR spectrum of **3** in CD_3OD.

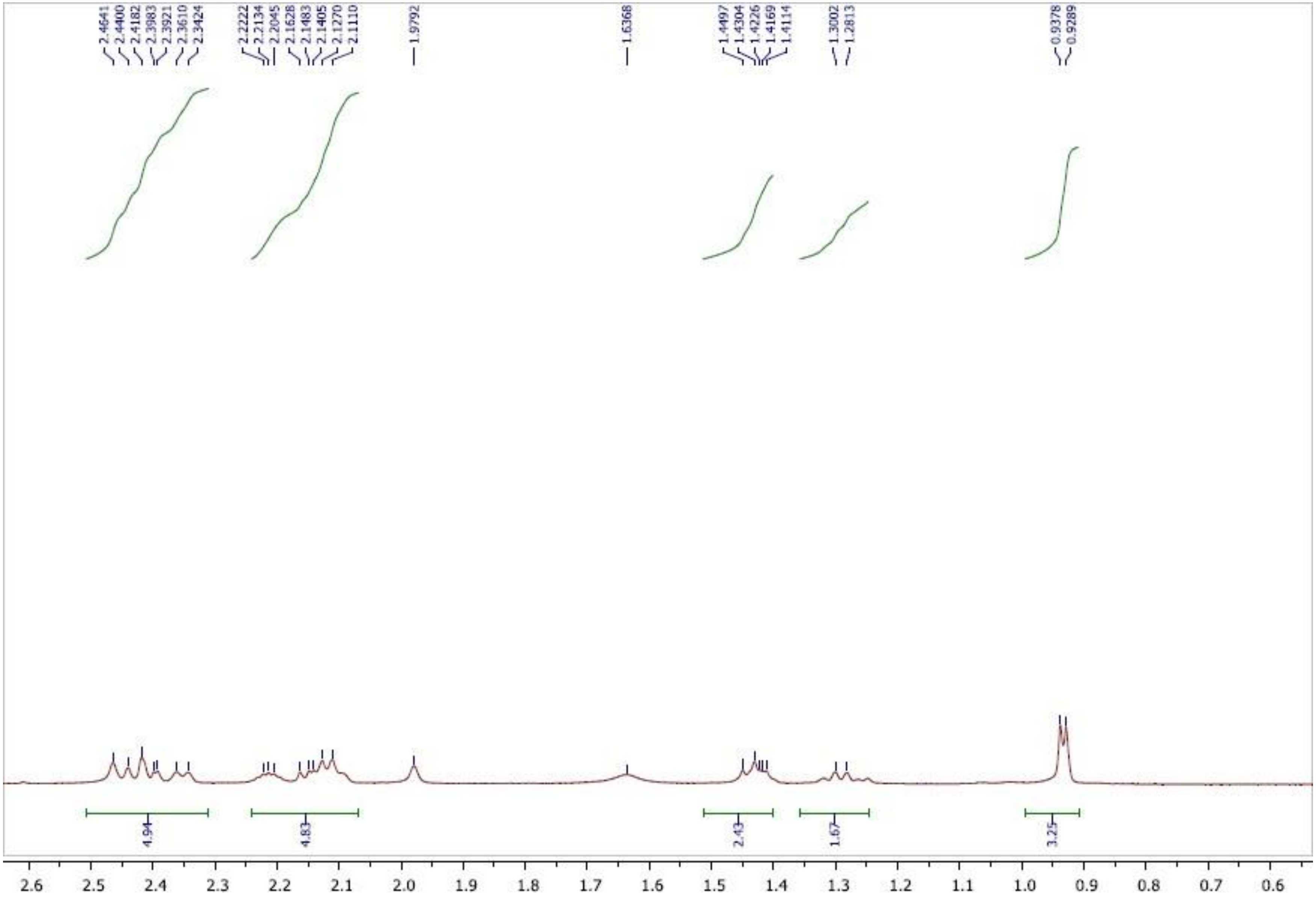

Figure S19. Expansion of part of ^{1}H NMR spectrum (δ 0.6 – δ 2.6) of **3** in CD_3OD.

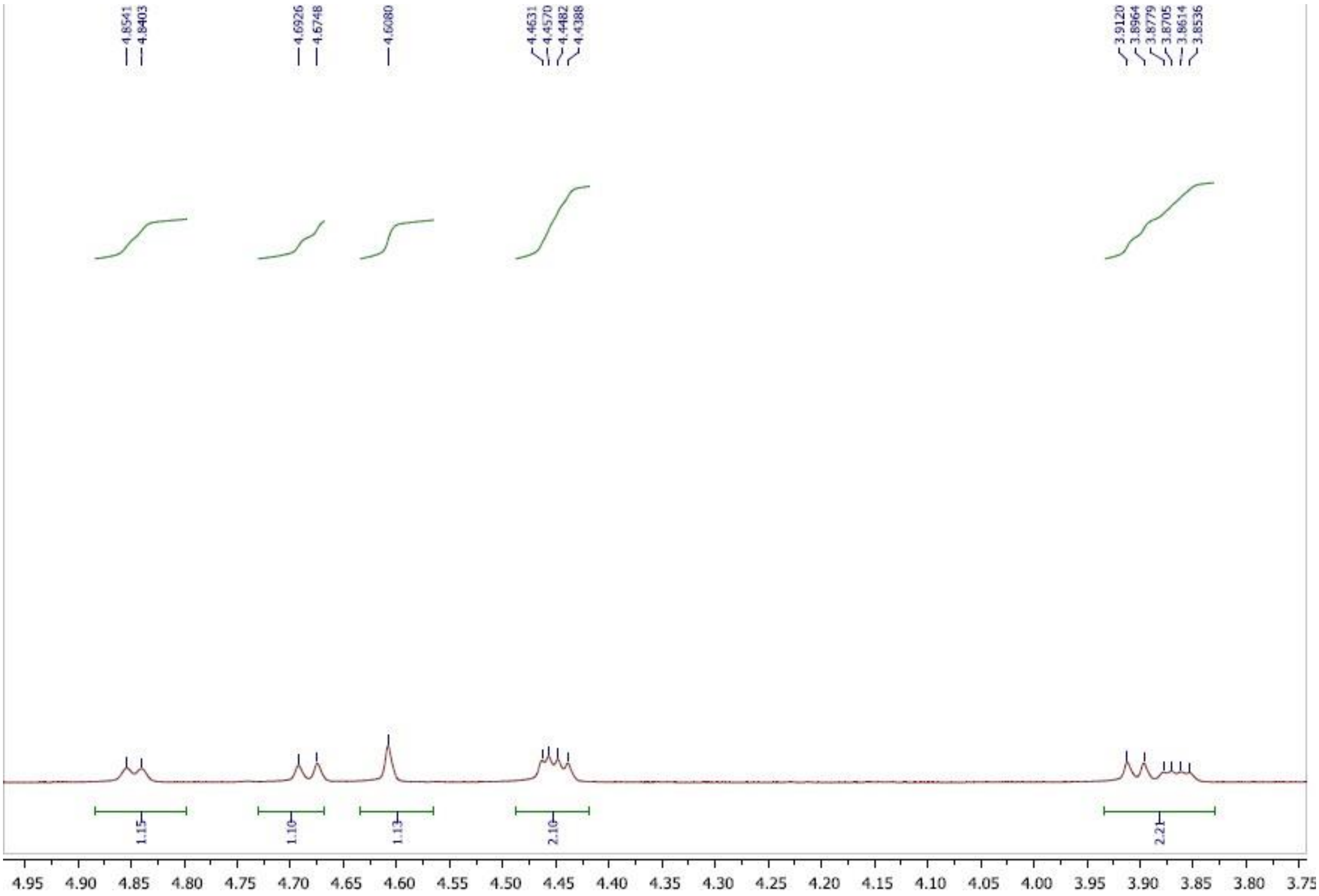

Figure S20. Expansion of part of ^{1}H NMR spectrum (δ 3.75 – δ 4.95) of **3** in CD_3OD.

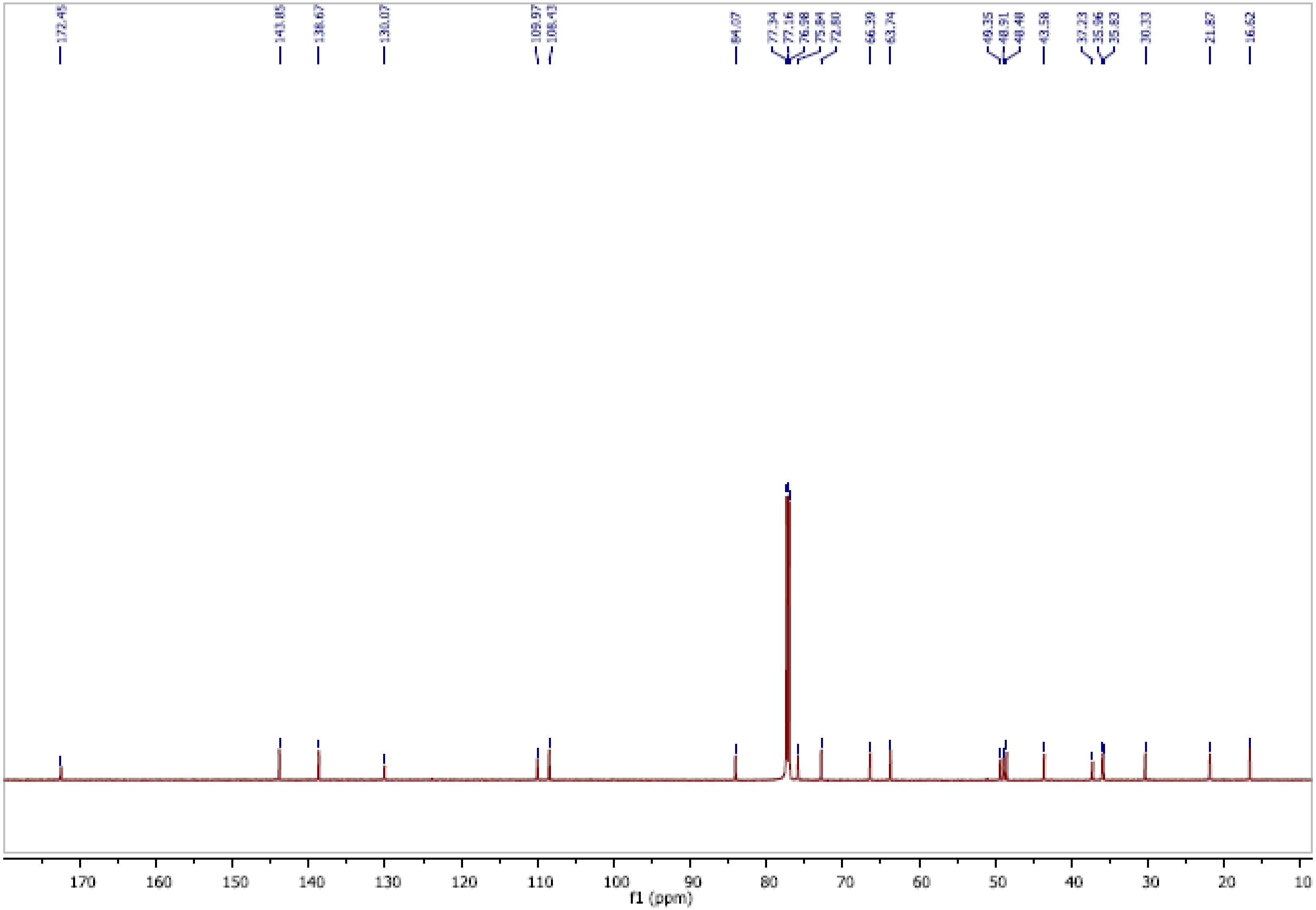

Figure S21. ^{13}C NMR spectrum of **3** in CD_3OD.

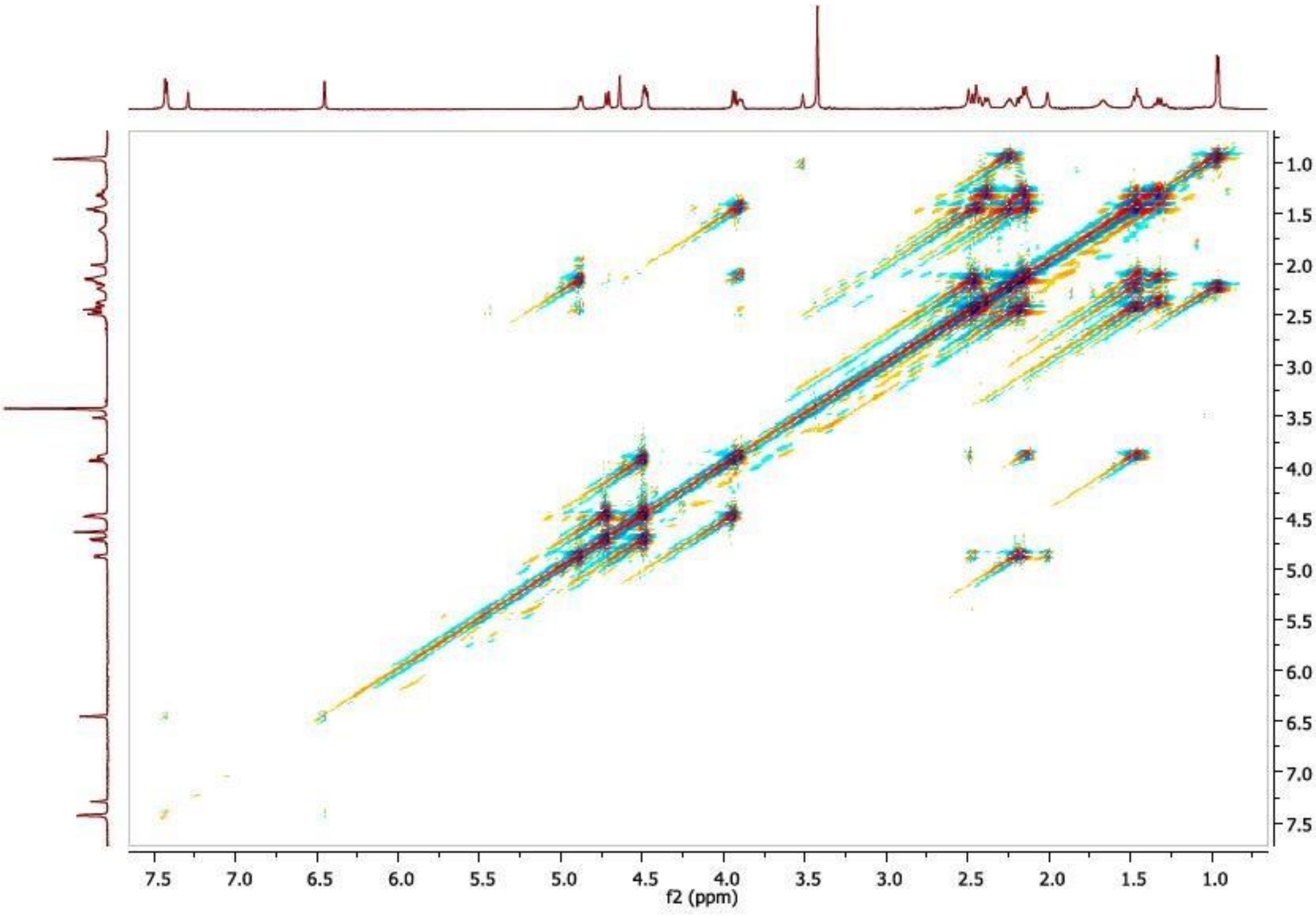

Figure S22. COSY NMR spectrum of **3** in CD_3OD.

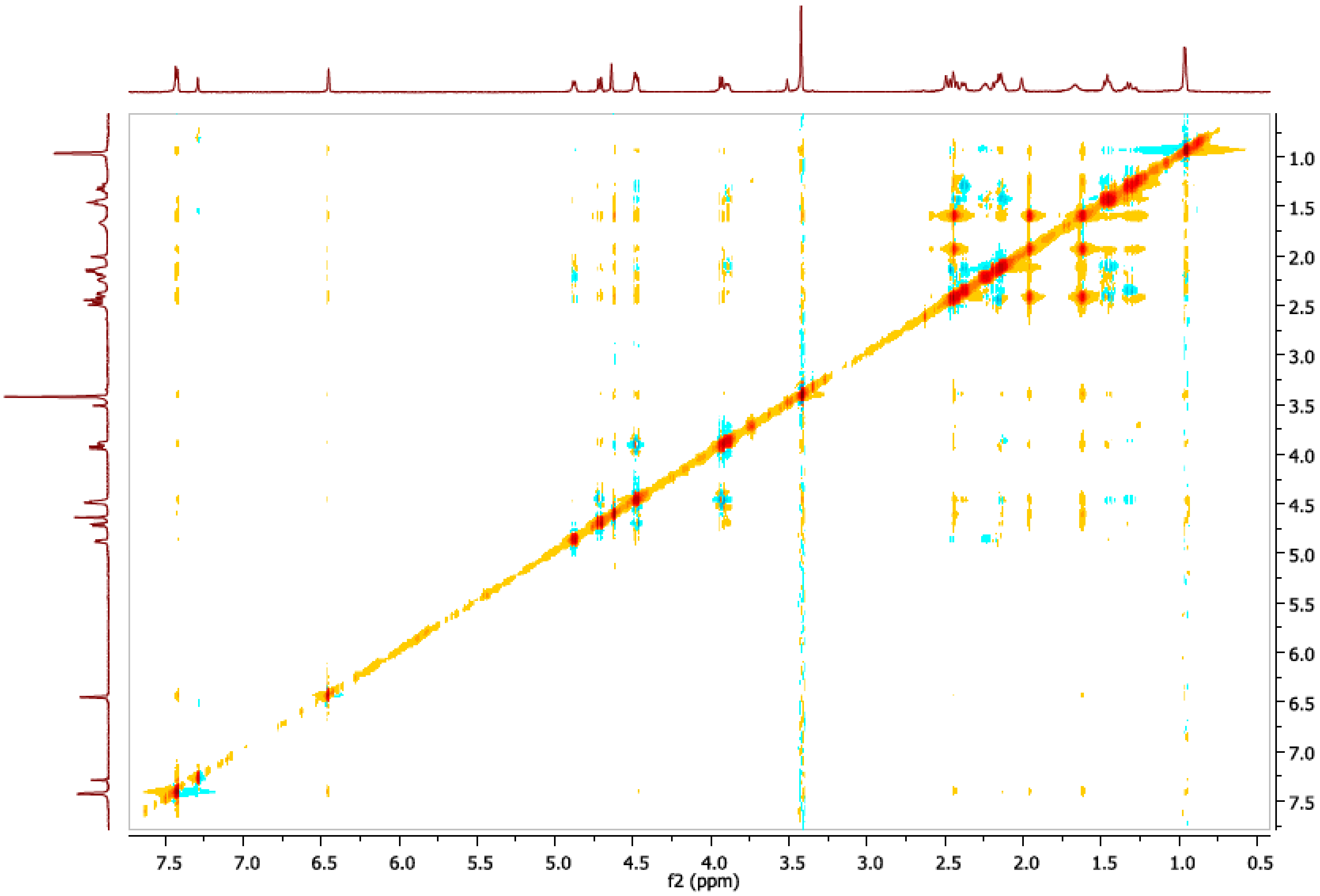

Figure S23. NOESY NMR spectrum of **3** in CD_3OD.

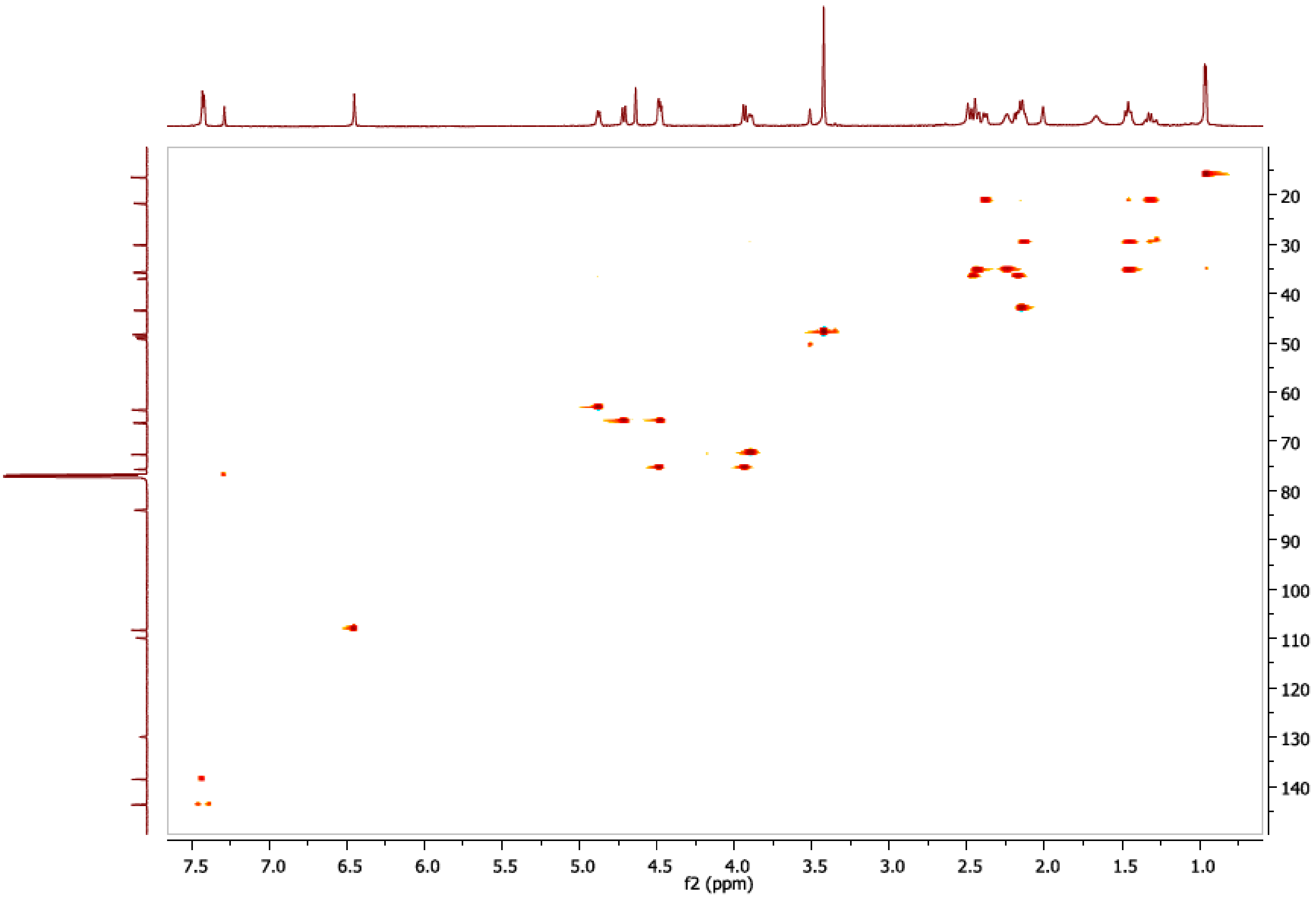

Figure S24. HSQC NMR spectrum of **3** in CD_3OD.

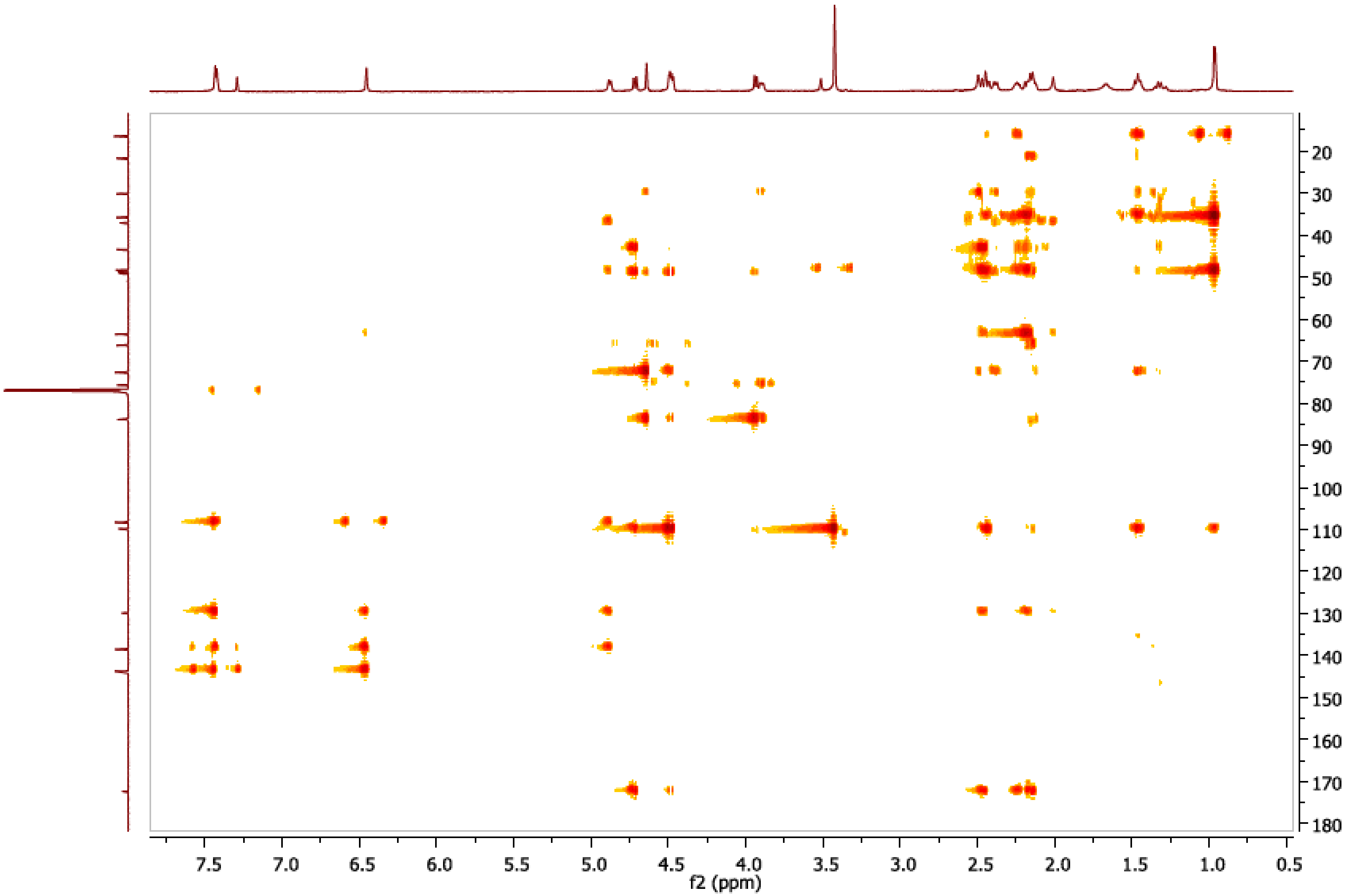

Figure S25. HMBC NMR spectrum of **3** in CD_3OD.

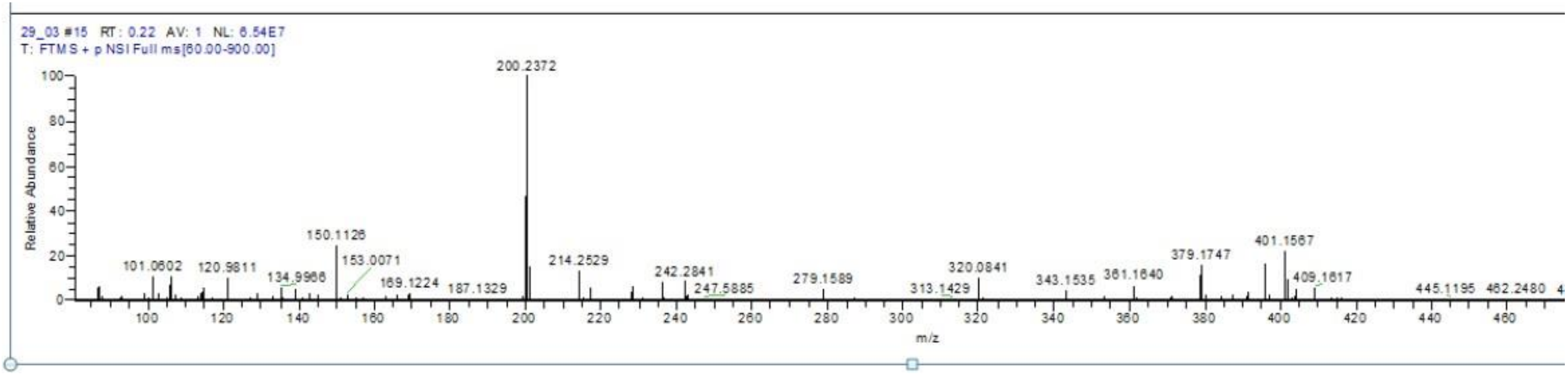

Figure S26. Positive-ion HRESIMS of **3**.

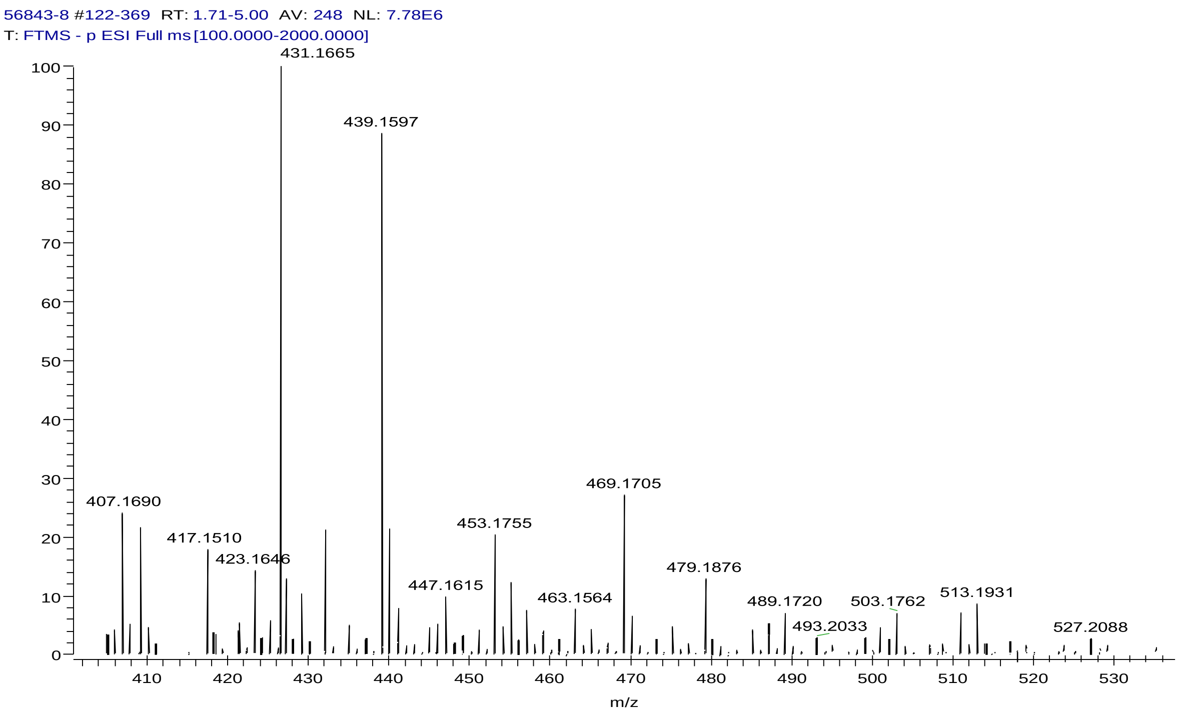

Figure S27. Negative-ion HRESIMS of **3**.

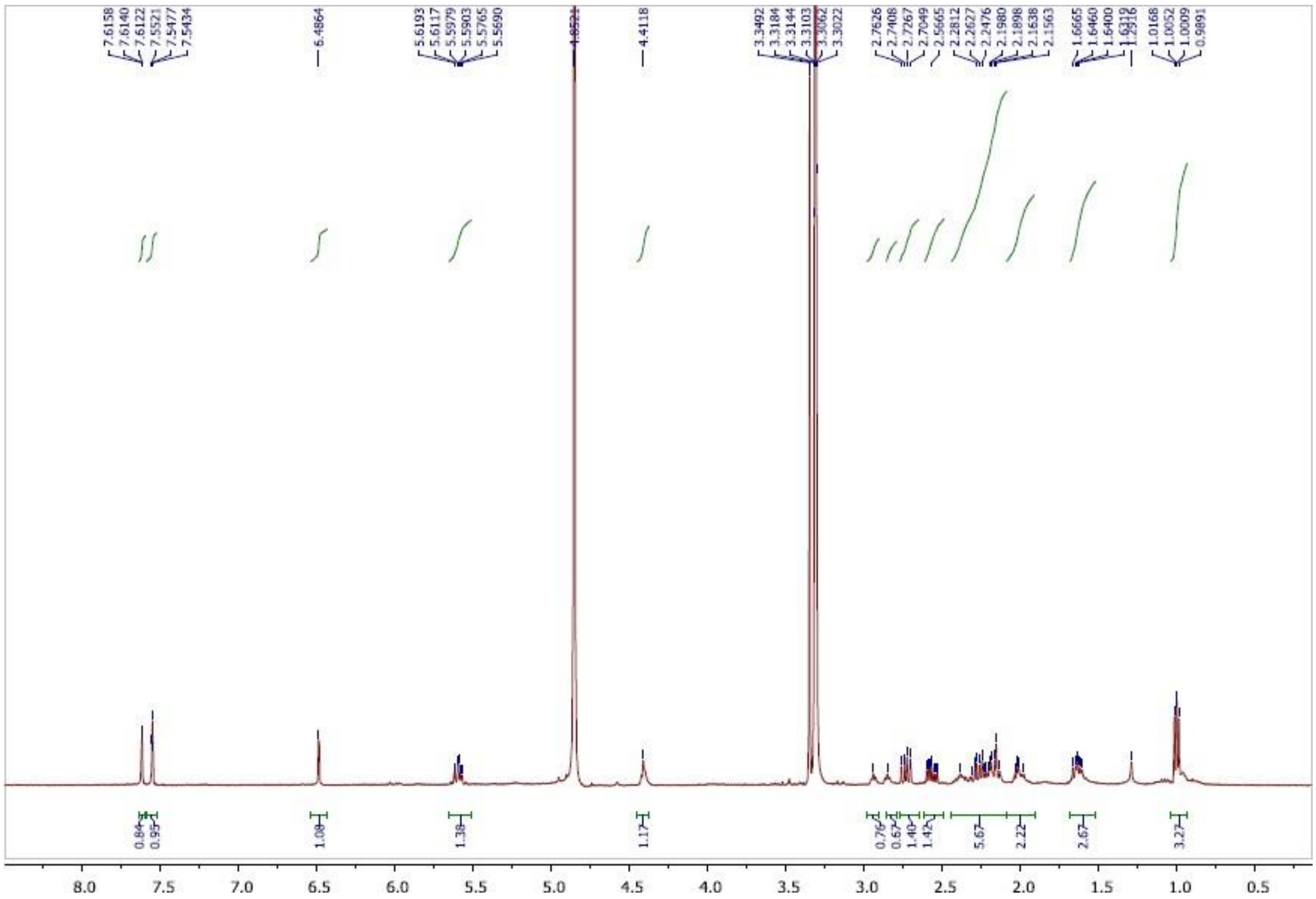

Figure S28. ^{1}H NMR spectrum of **4** in CD_3OD.

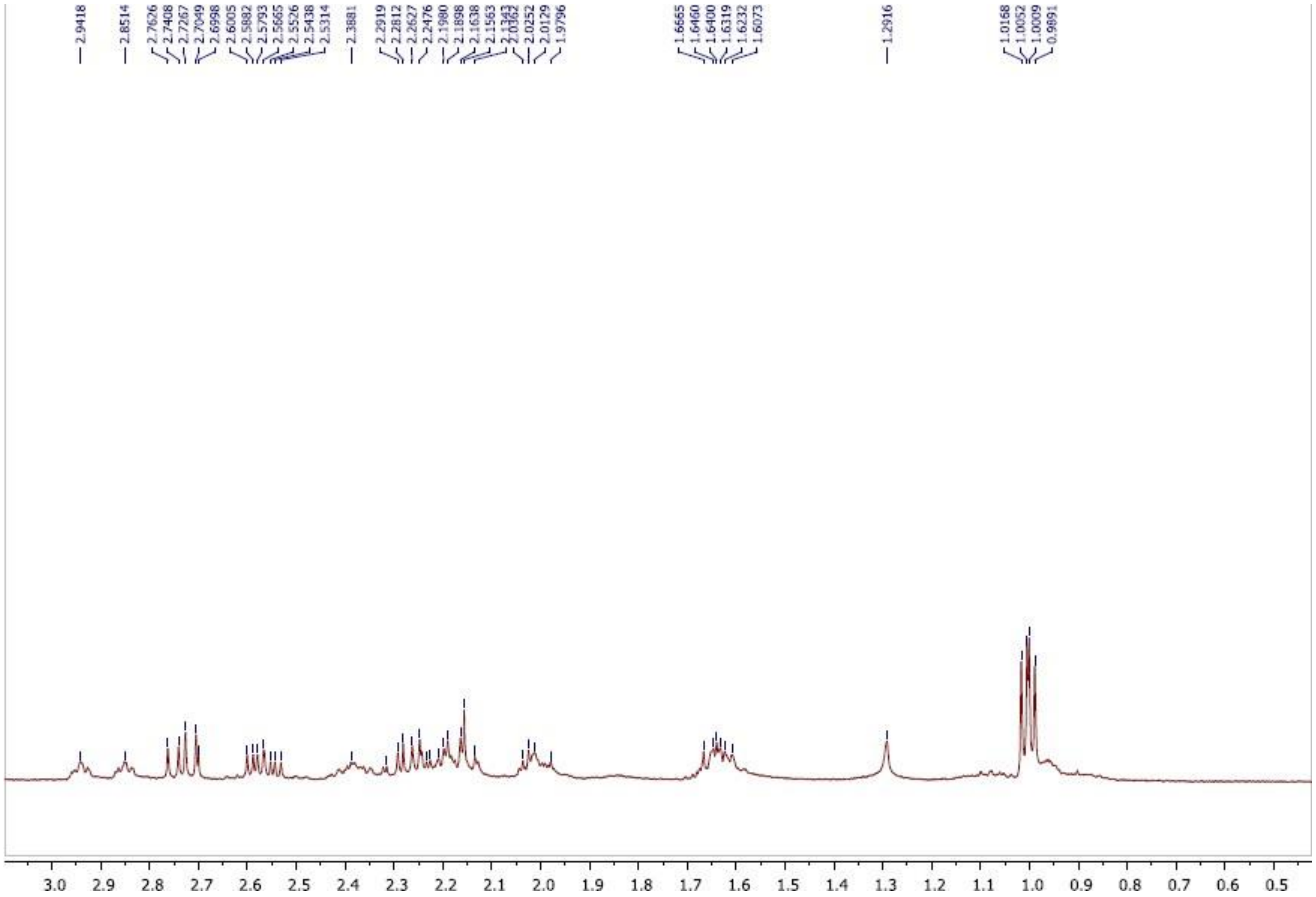

Figure S29. Expansion of part of ^{1}H NMR spectrum (δ 0.5 – δ 3.0) of **4** in CD_3OD.

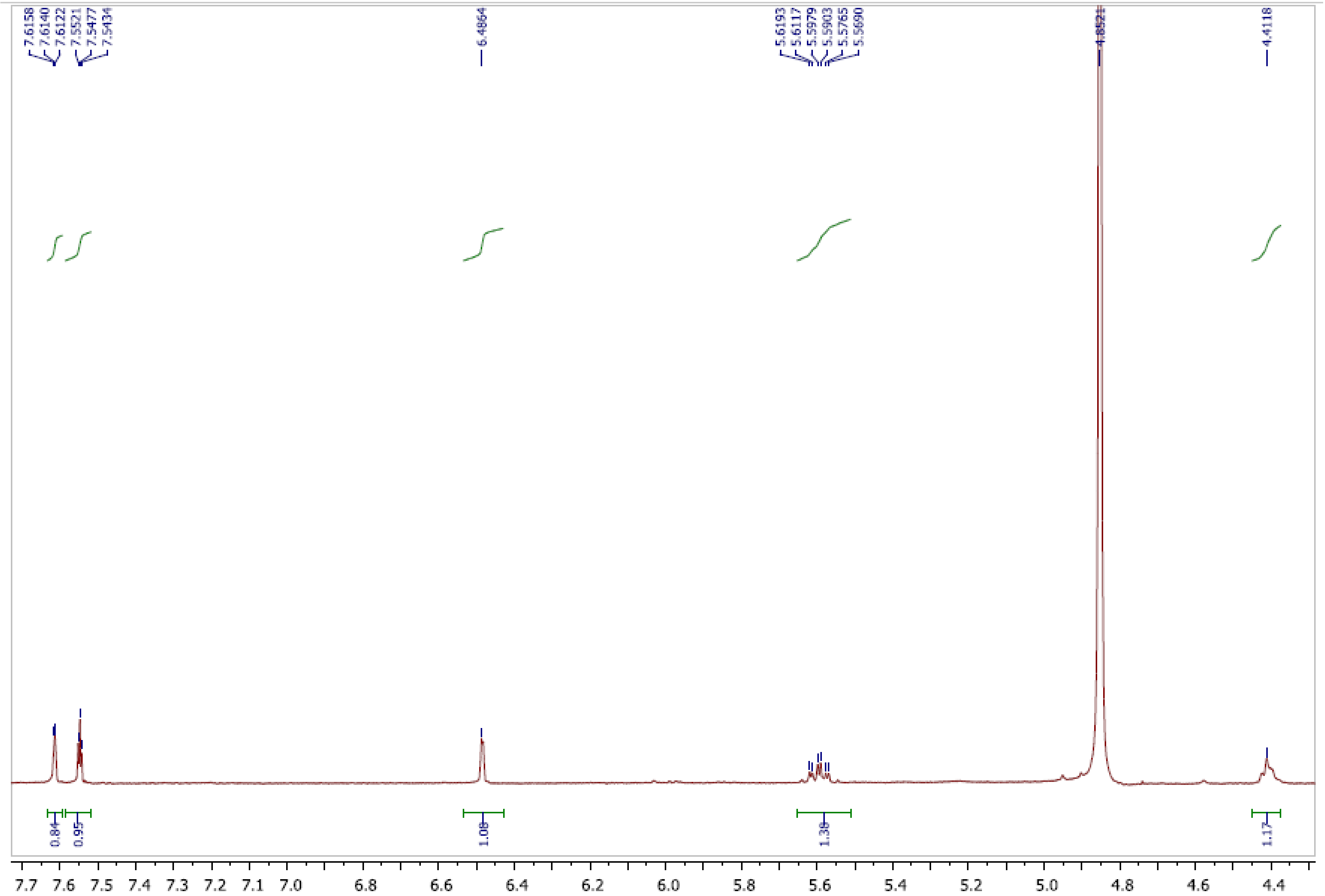

Figure S30. Expansion of part of ^{1}H NMR spectrum (δ 4.3 – δ 7.7) of **4** in CD_3OD.

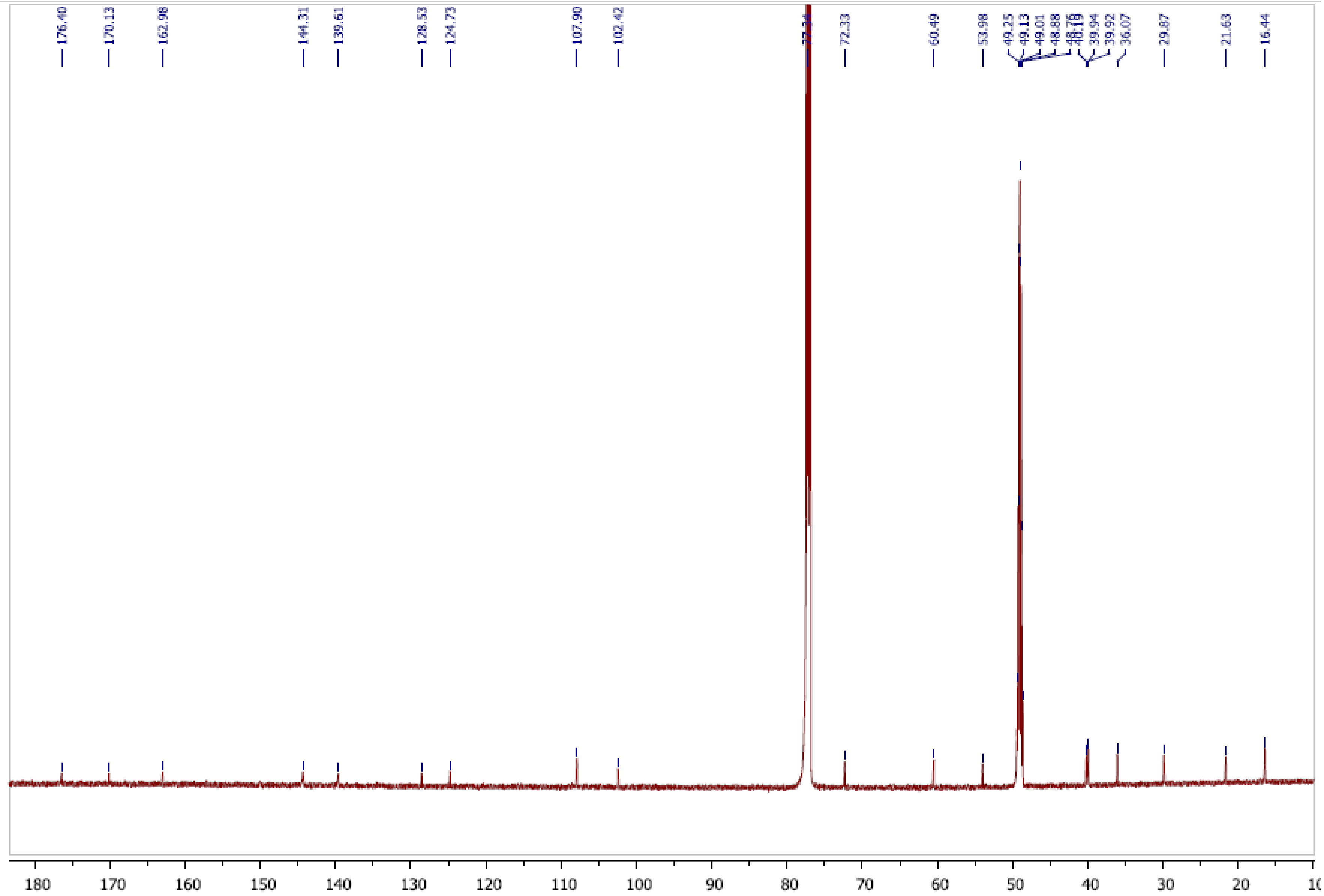

Figure S31. ^{13}C NMR spectrum of **4** in CD_3OD / $CDCl_3$.

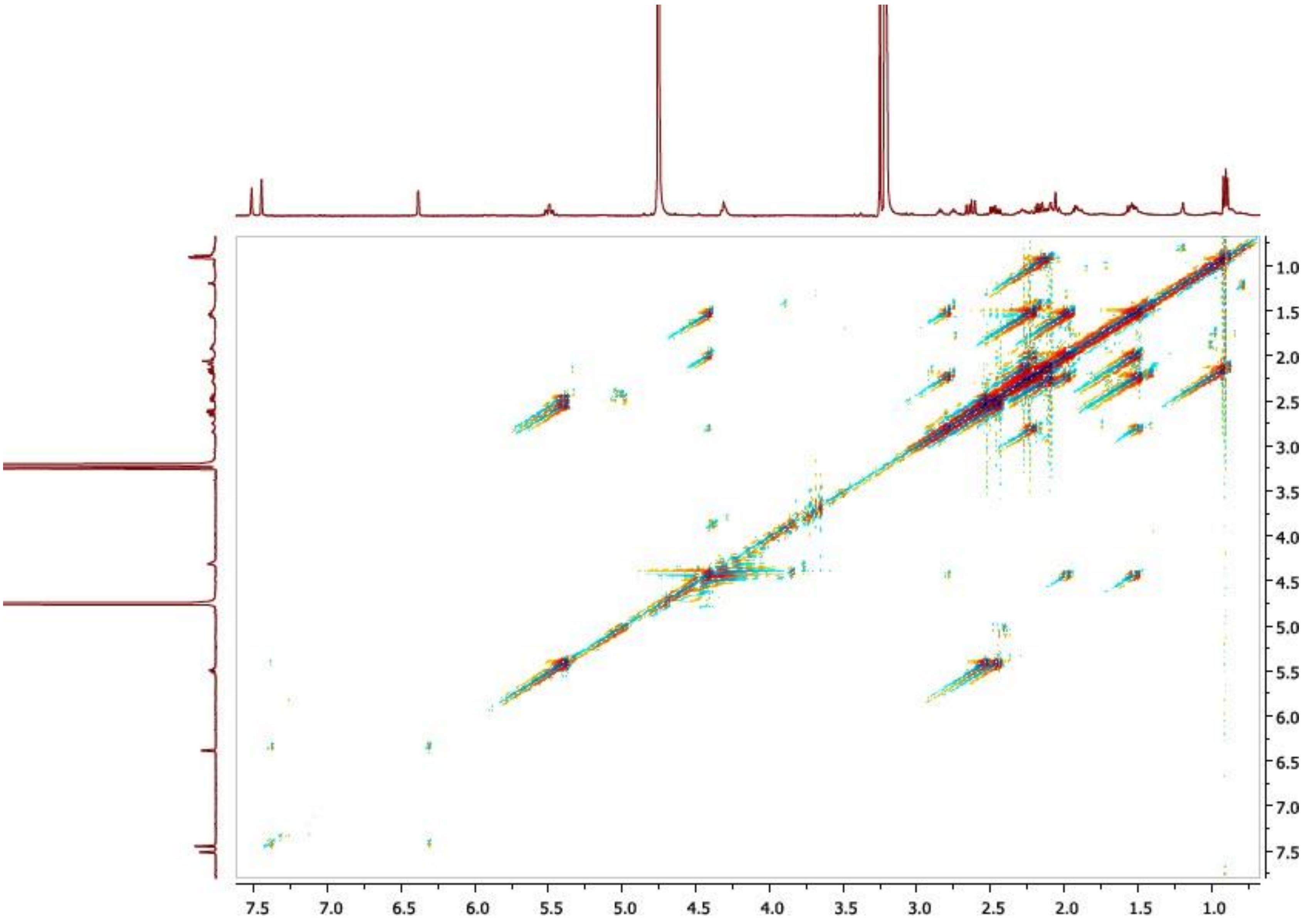

Figure S32. COSY NMR spectrum of **4** in CD_3OD.

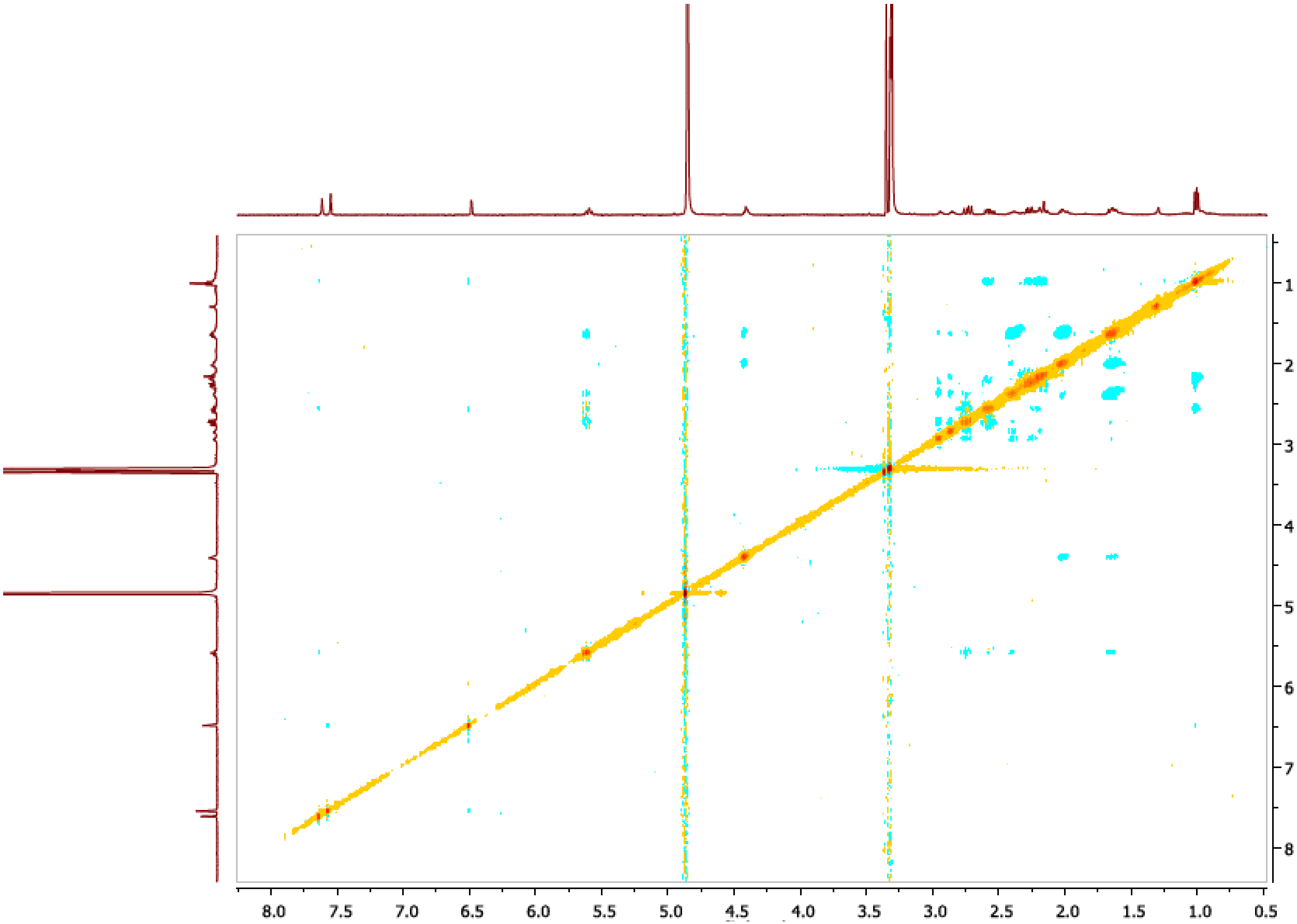

Figure S33. NOESY NMR spectrum of **4** in CD_3OD.

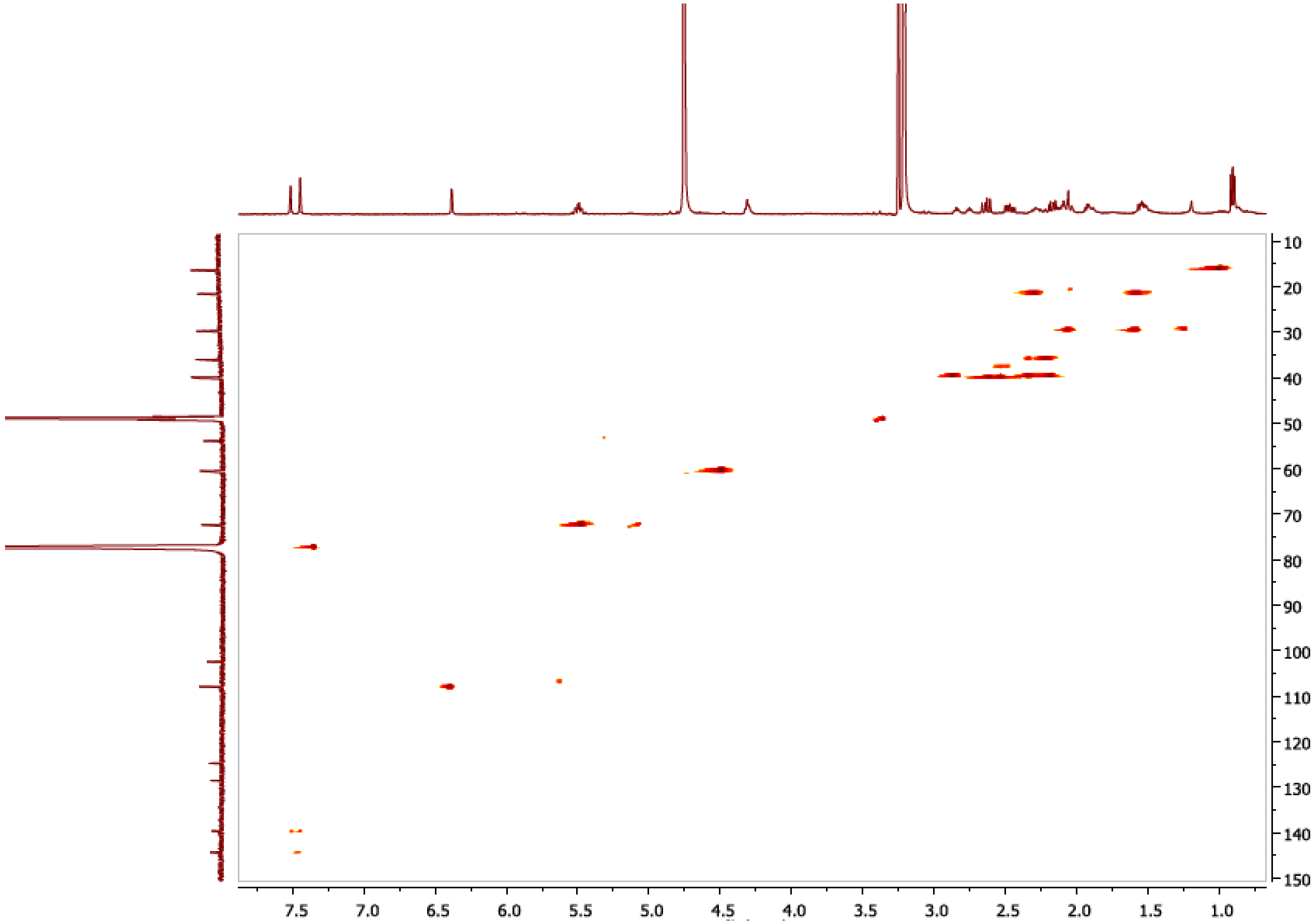

Figure S34. HSQC NMR spectrum of **4** in CD_3OD.

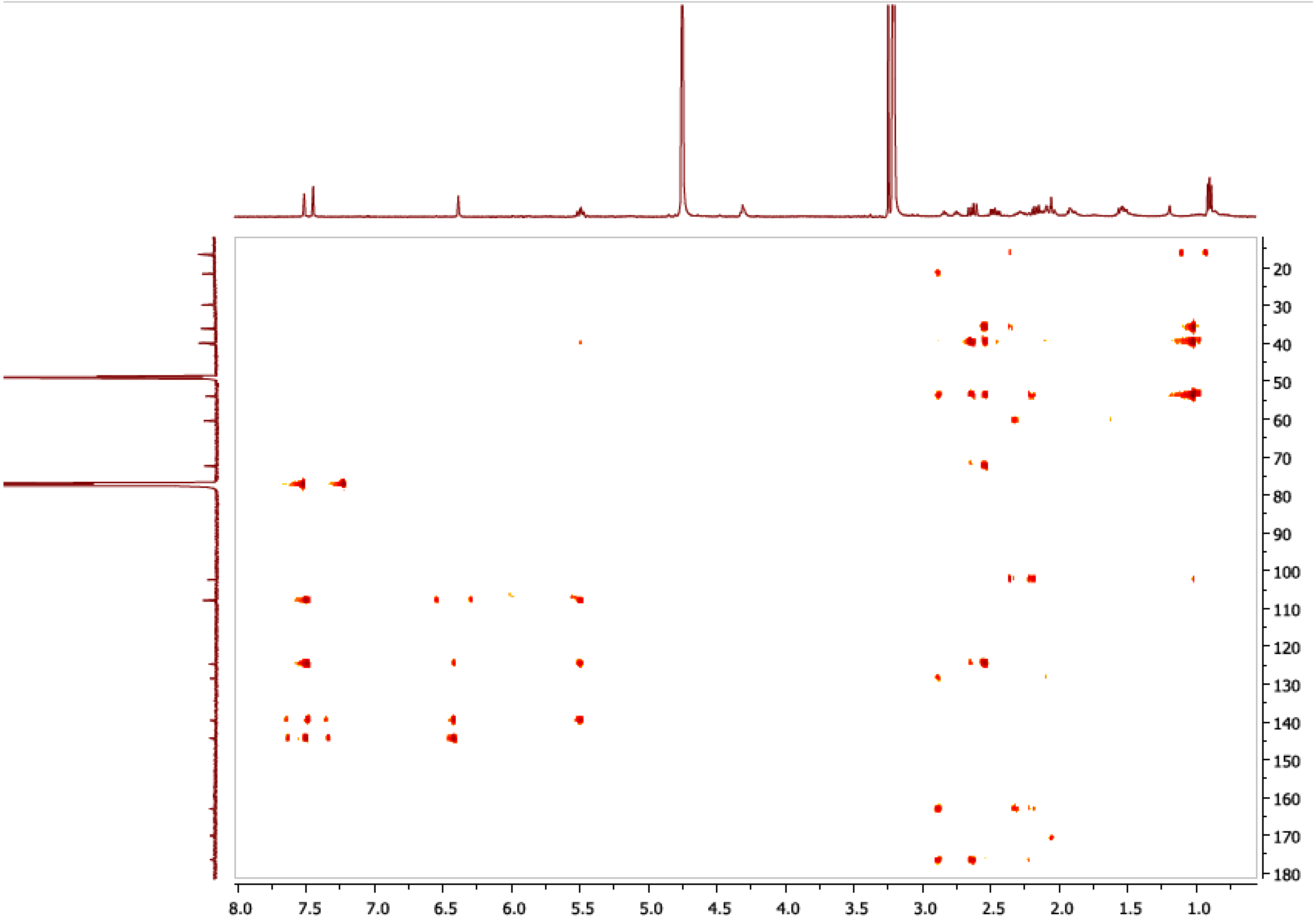

Figure S35. HMBC NMR spectrum of **4** in CD_3OD.

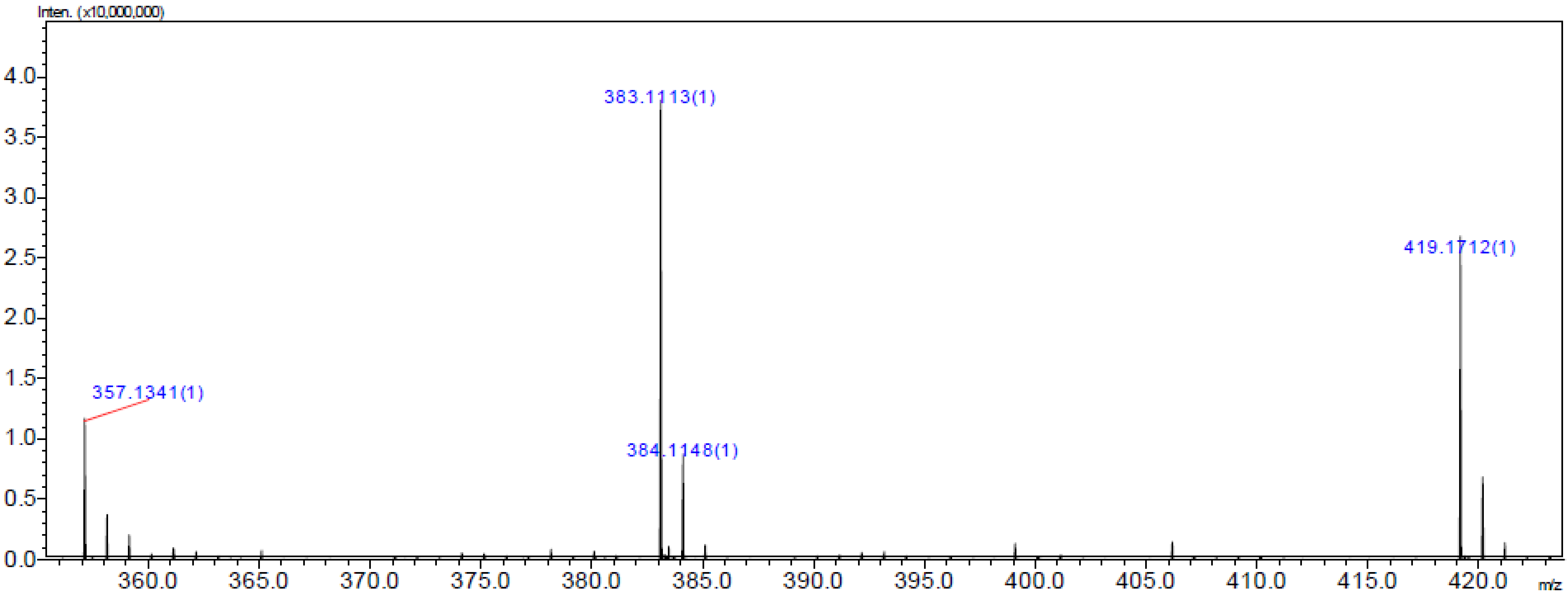

Figure S36. Positive-ion HRESIMS of **4**.

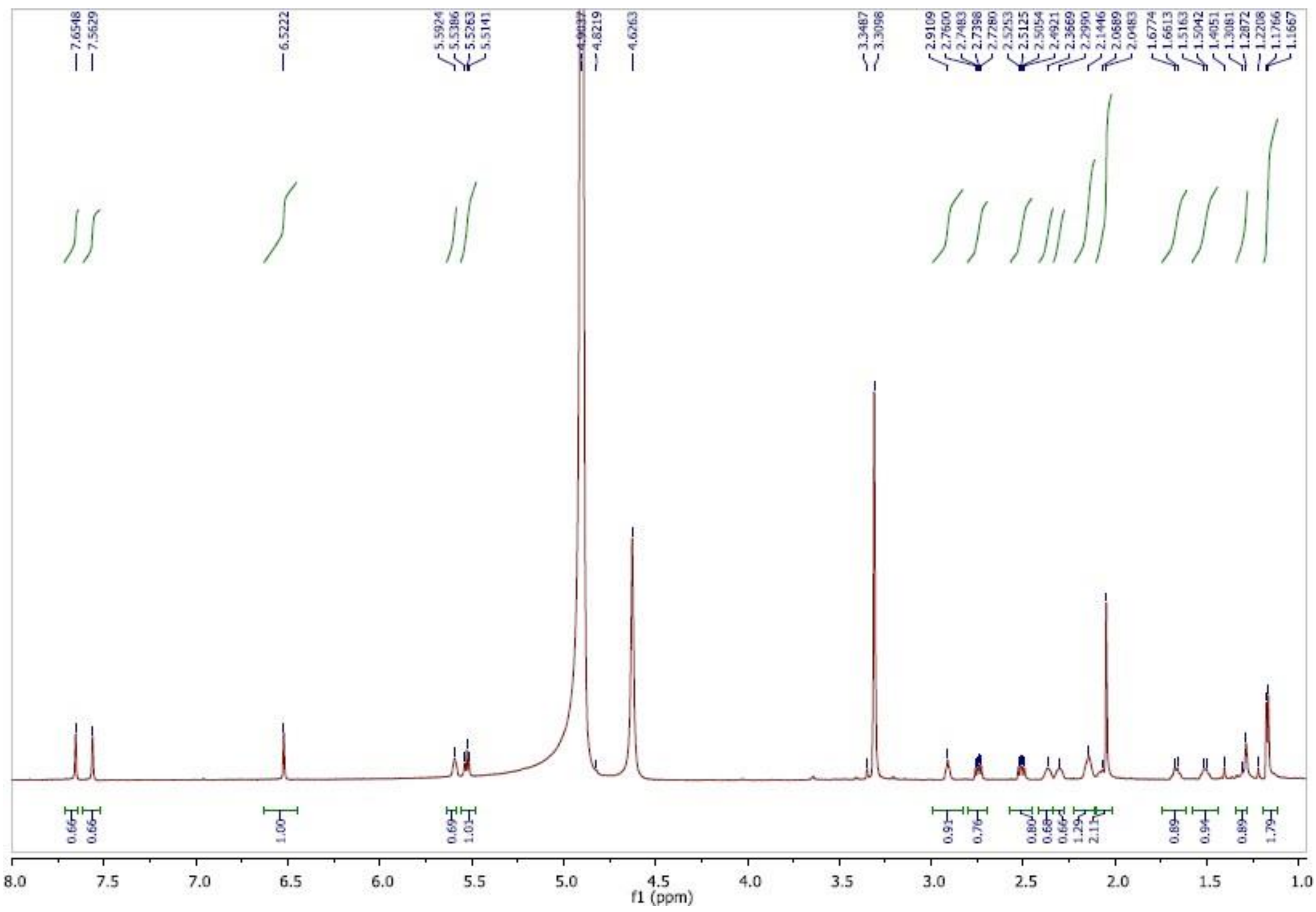

Figure S37. ^{1}H NMR spectrum of **5** in CD_3OD.

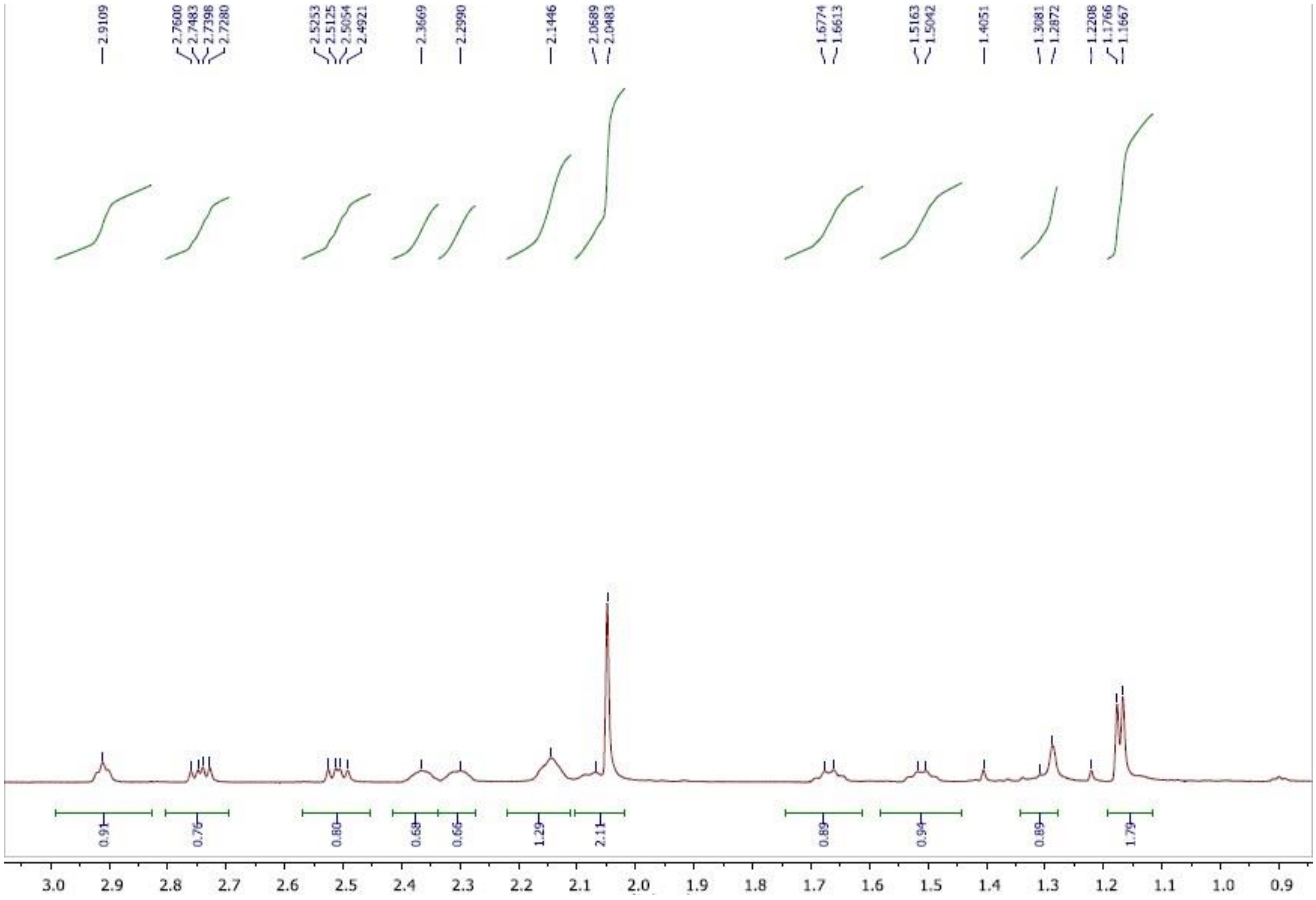

Figure S38. Expansion of part of ^{1}H NMR spectrum (δ 0.9 – δ 3.0) of **5** in CD_3OD.

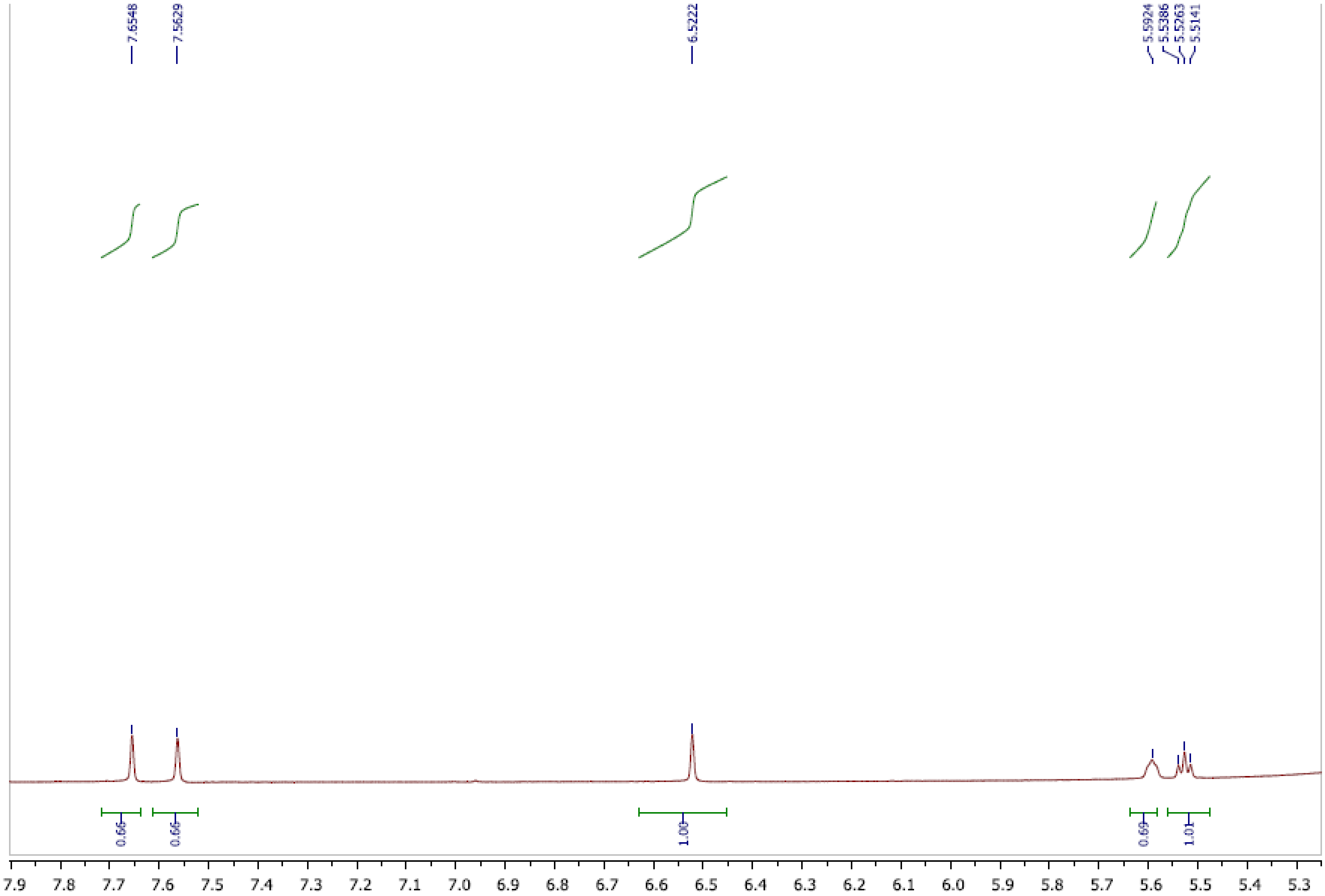

Figure S39. Expansion of part of ^{1}H NMR spectrum (δ 5.3 – δ 7.9) of **5** in CD_3OD.

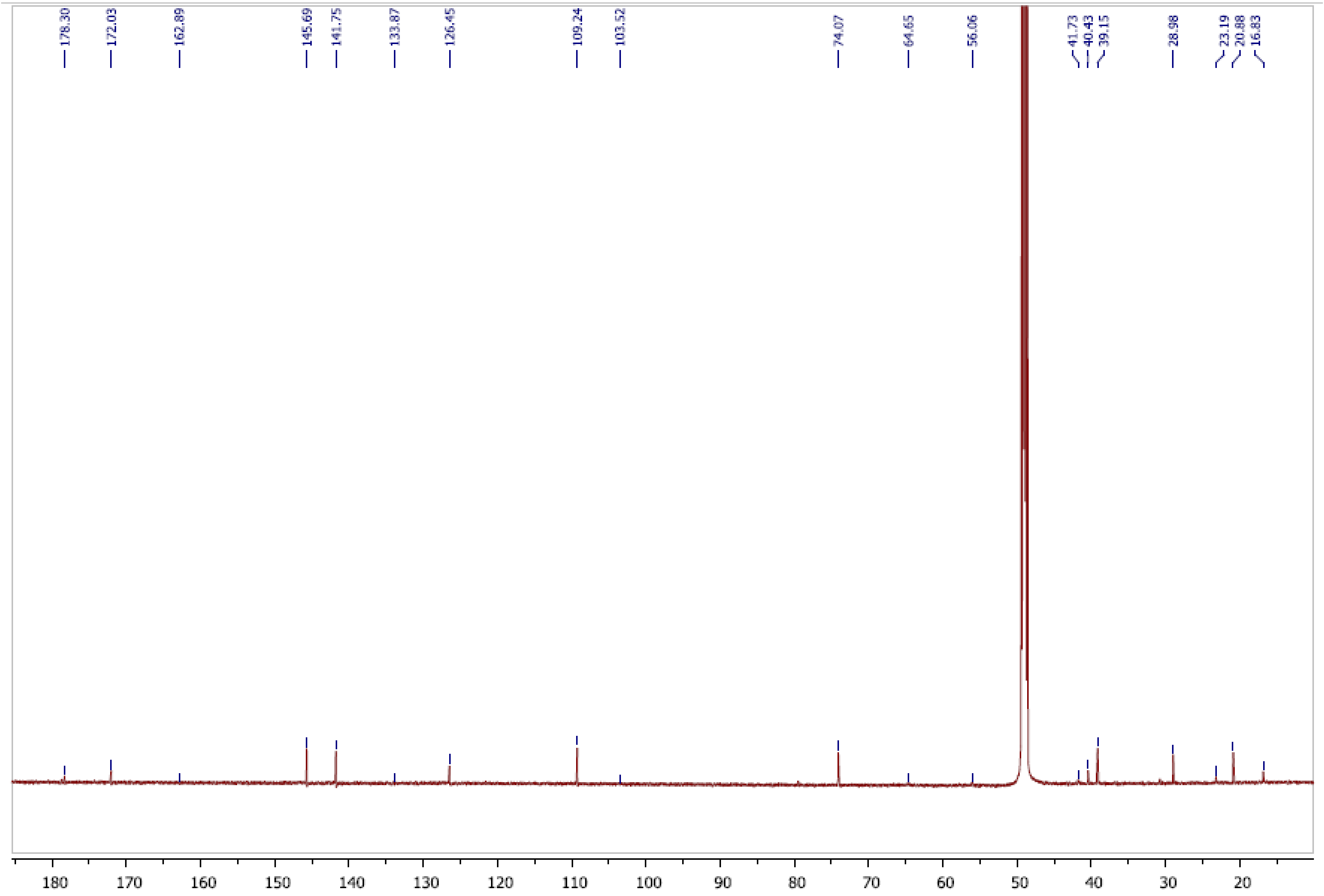

Figure S40. ^{13}C NMR spectrum of **5** in CD_3OD.

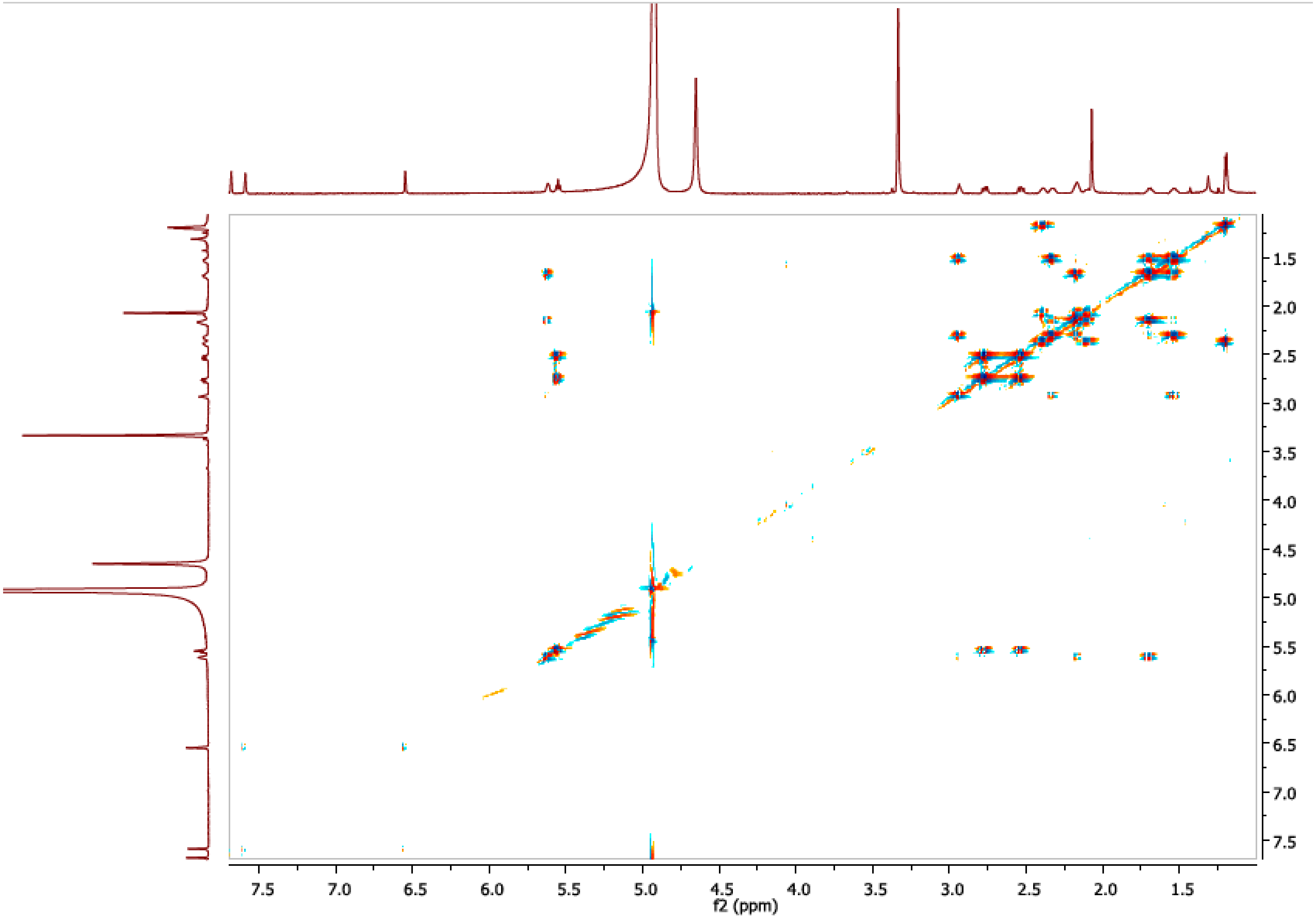

Figure S41. COSY NMR spectrum of **5** in CD_3OD.

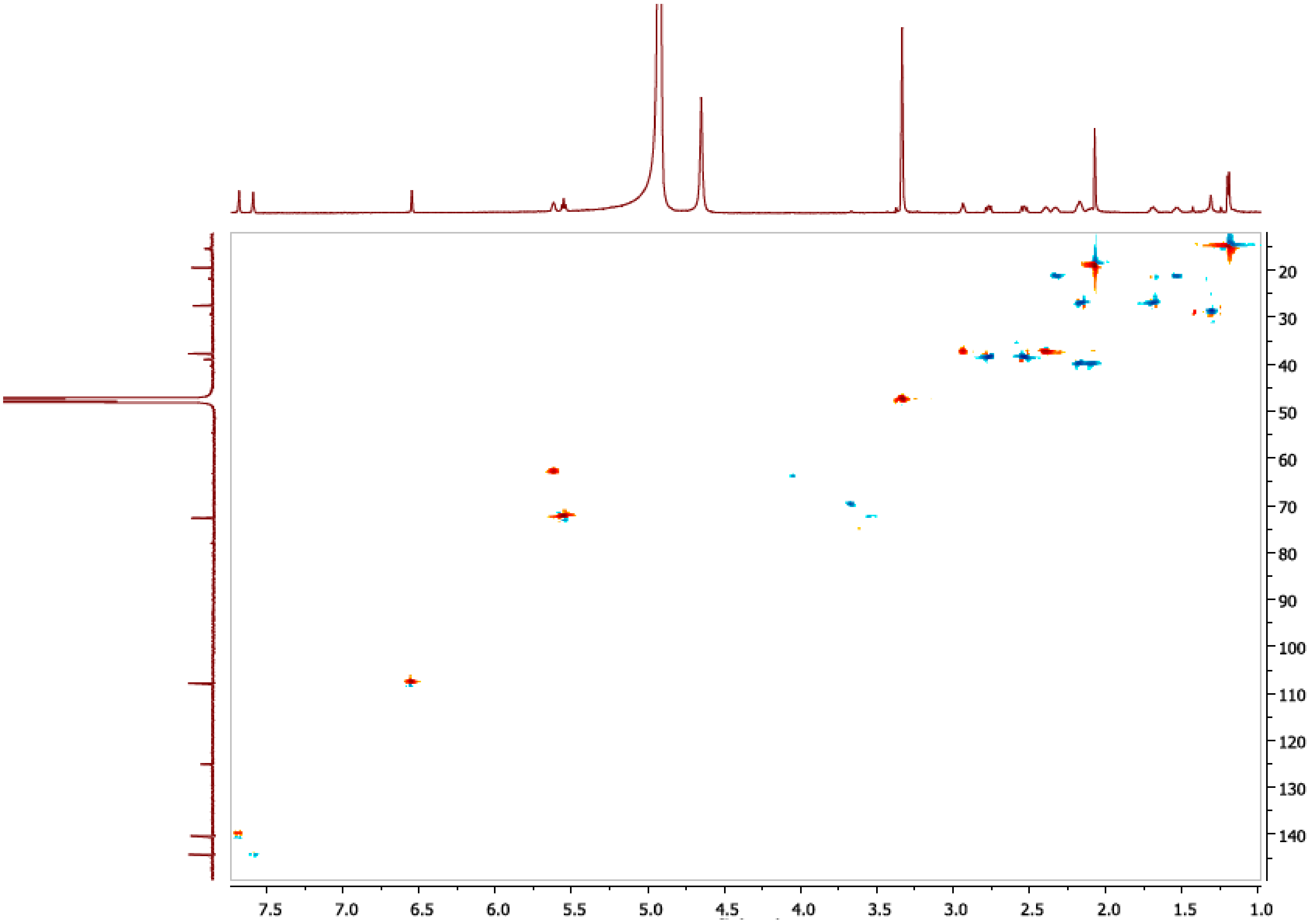

Figure S42. HSQC NMR spectrum of **5** in CD_3OD.

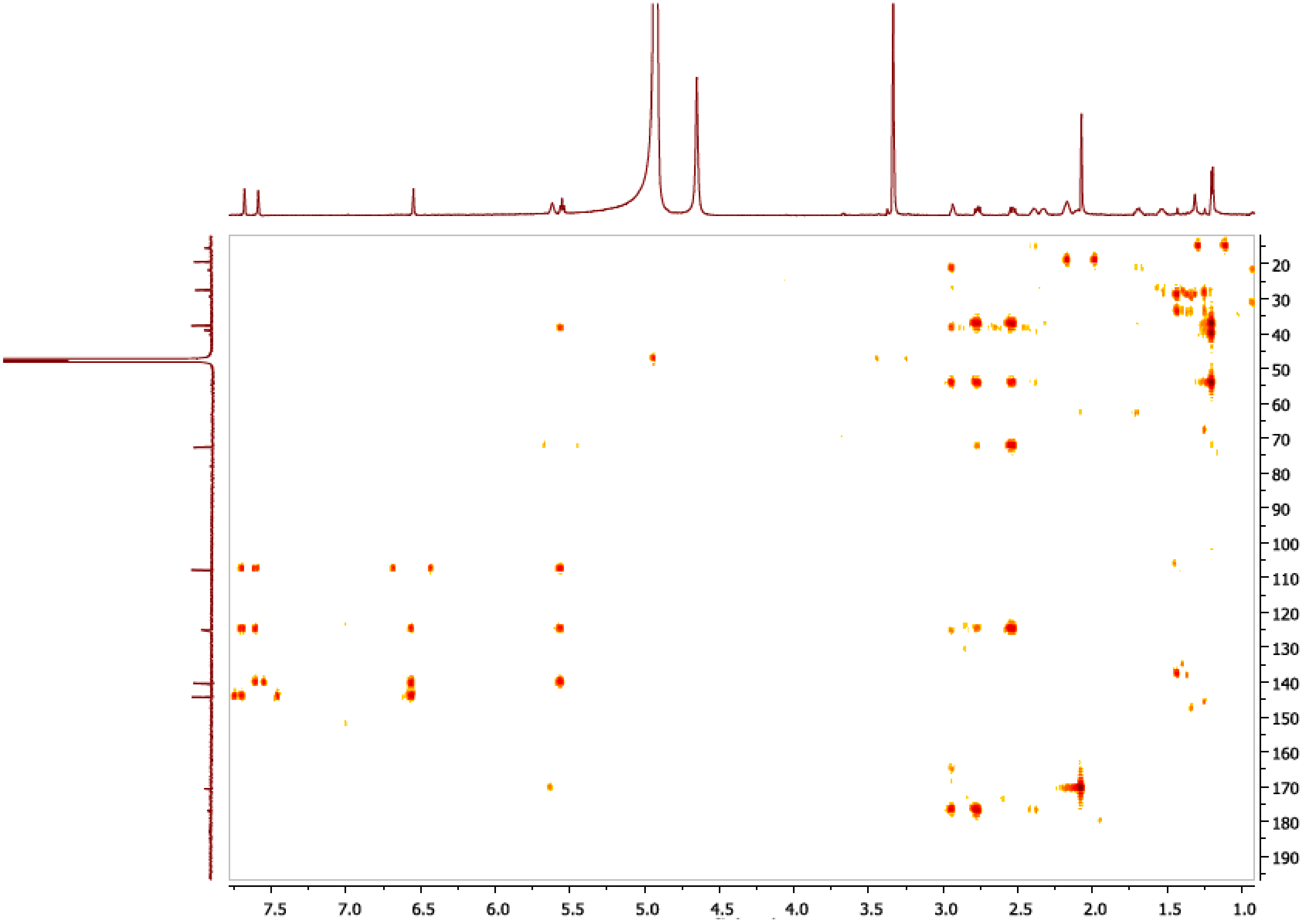

Figure S43. HMBC NMR spectrum of **5** in CD_3OD.

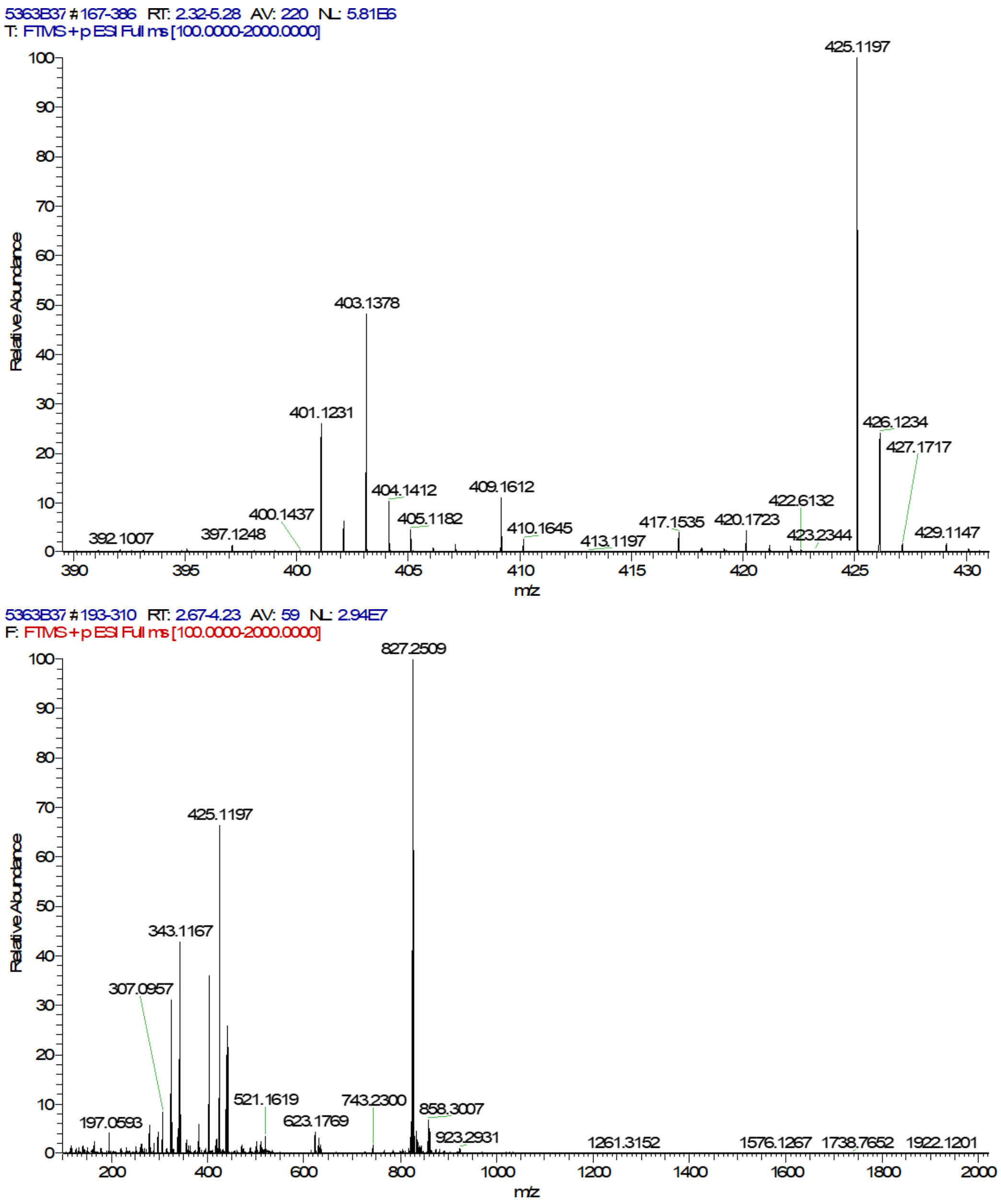

Figure S44. Positive-ion HRESIMS of **5**.

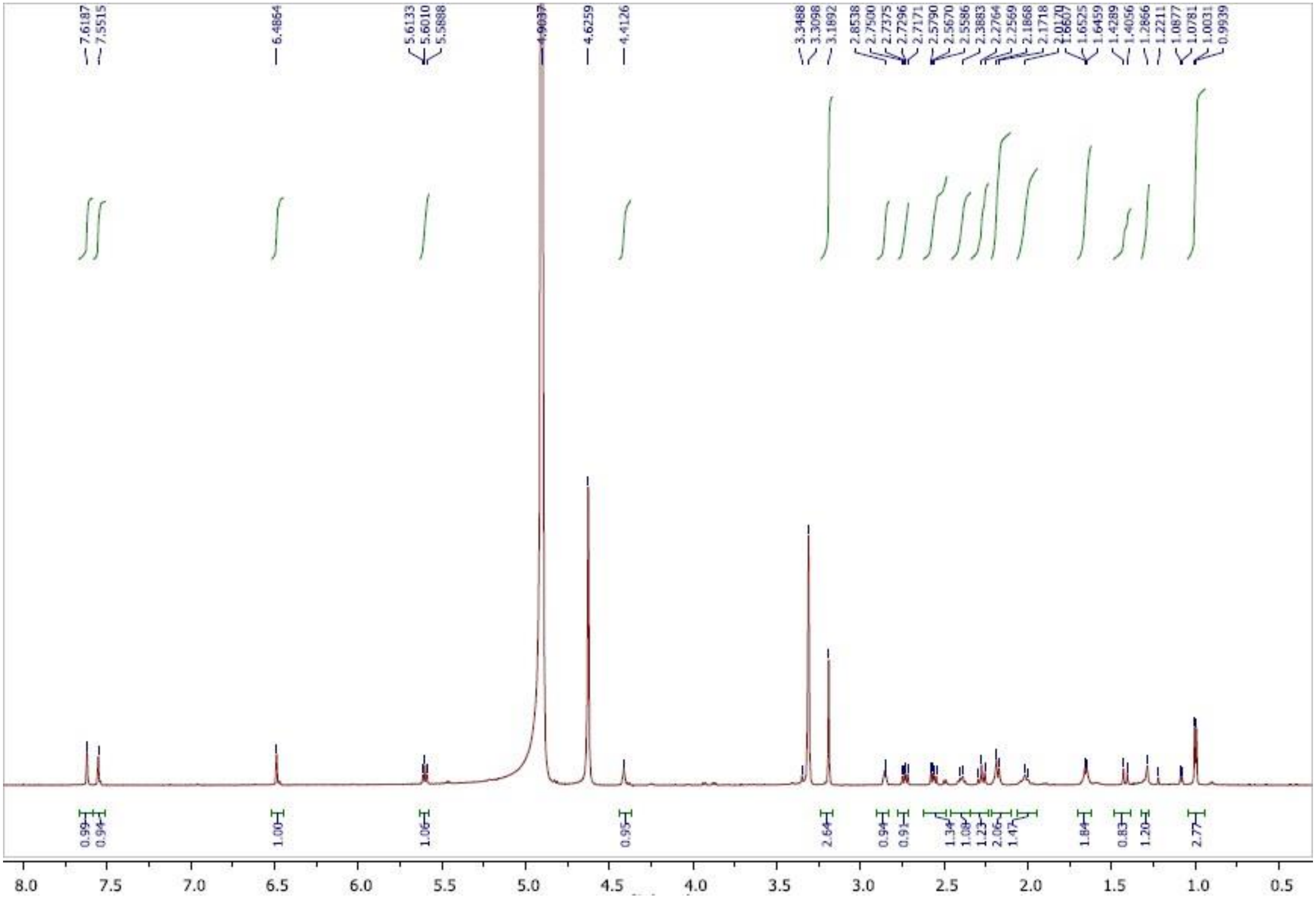

Figure S45. ^{1}H NMR spectrum of **6** in CD_3OD.

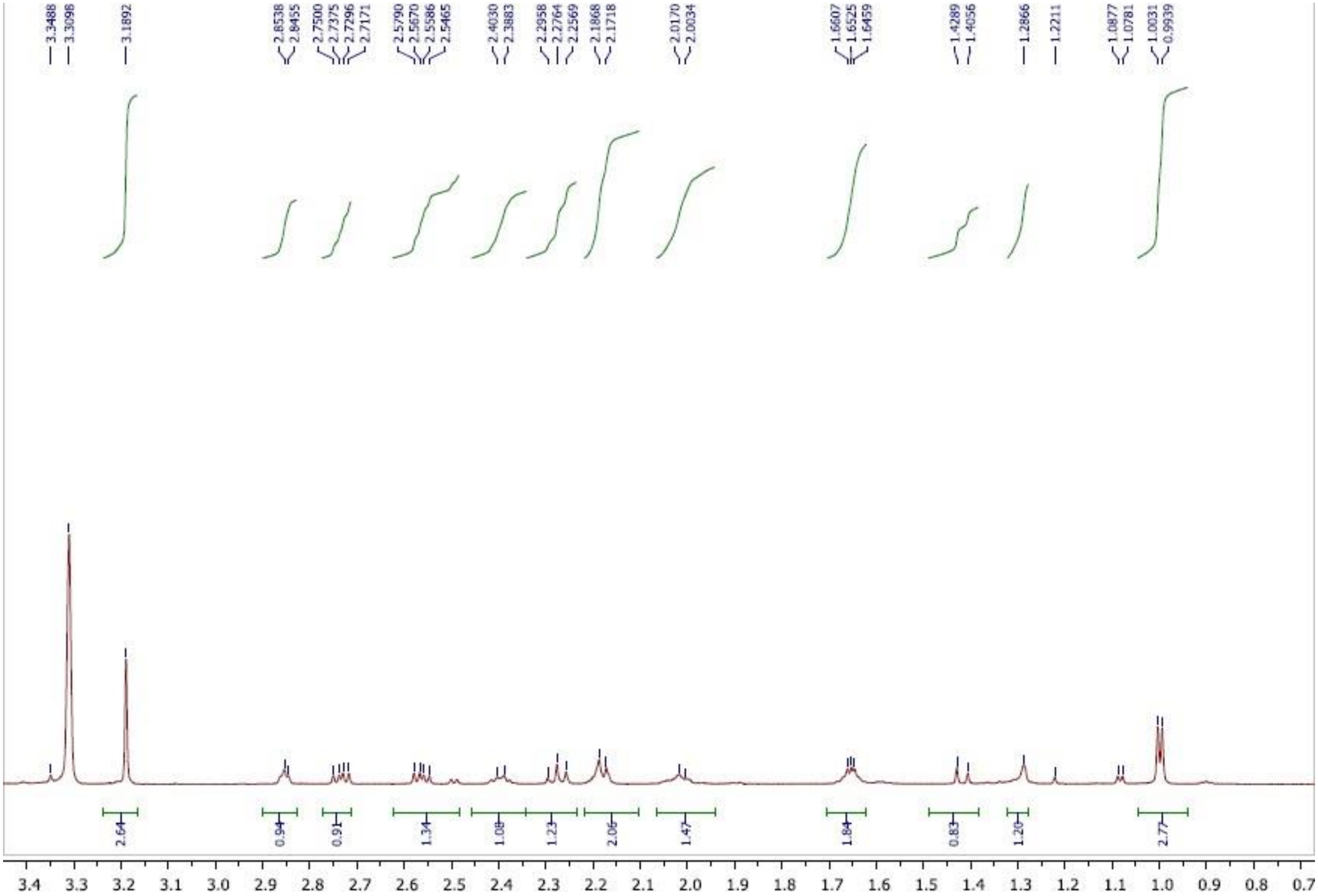

Figure S46. Expansion of part of ^{1}H NMR spectrum (δ 0.7 – δ 3.4) of **6** in CD_3OD.

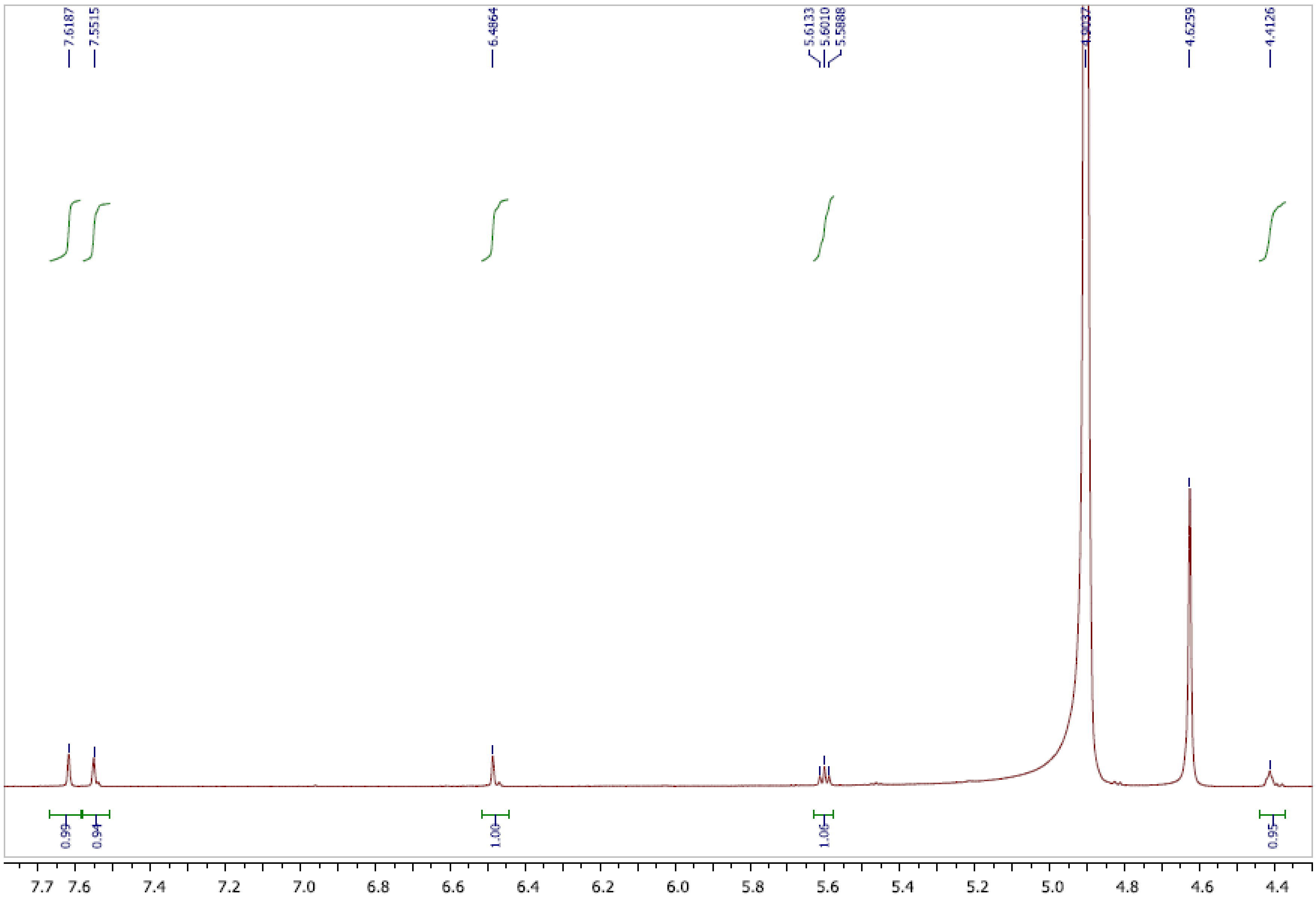

Figure S47. Expansion of part of ^{1}H NMR spectrum (δ 4.3 – δ 7.7) of **6** in CD_3OD.

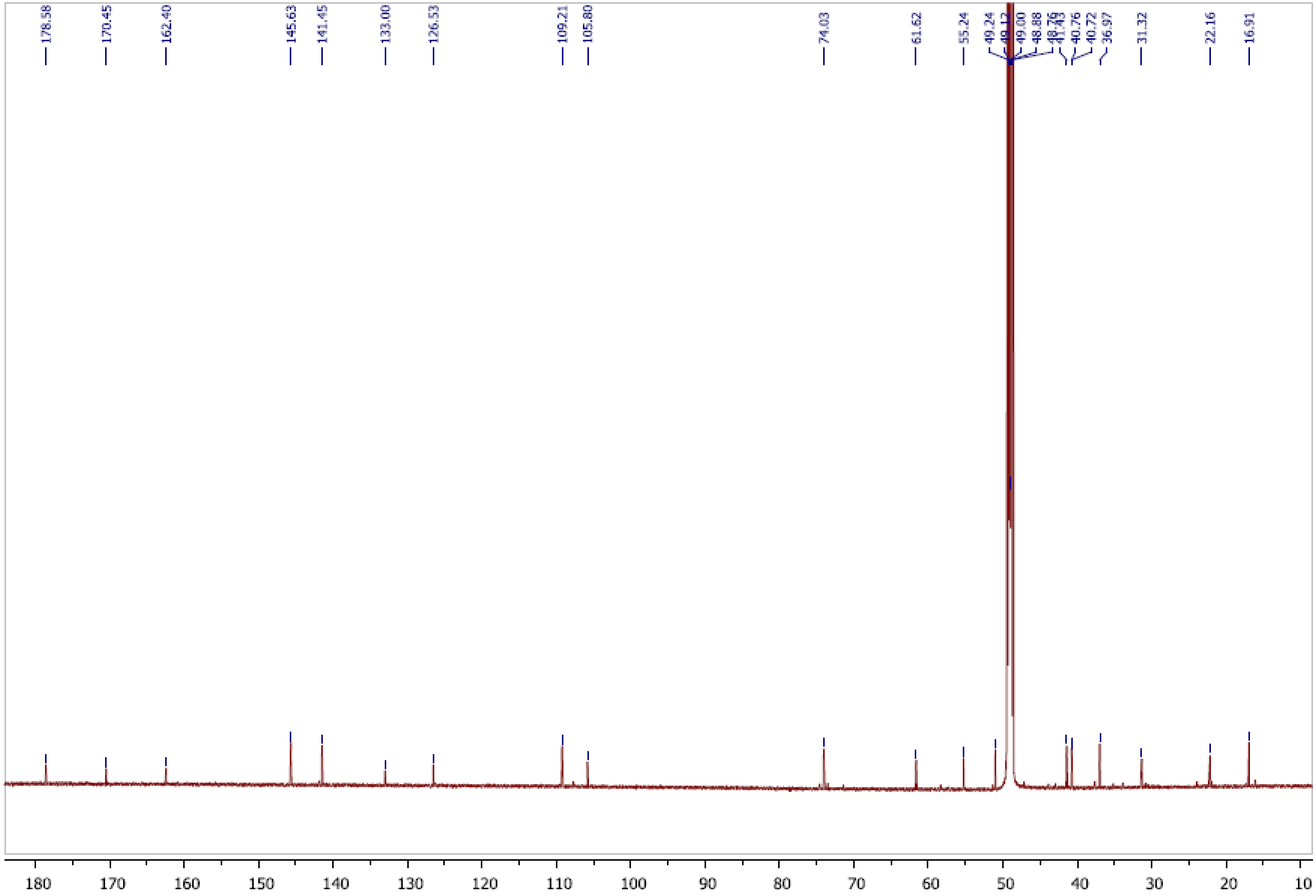

Figure S48. ^{13}C NMR spectrum of **6** in CD_3OD.

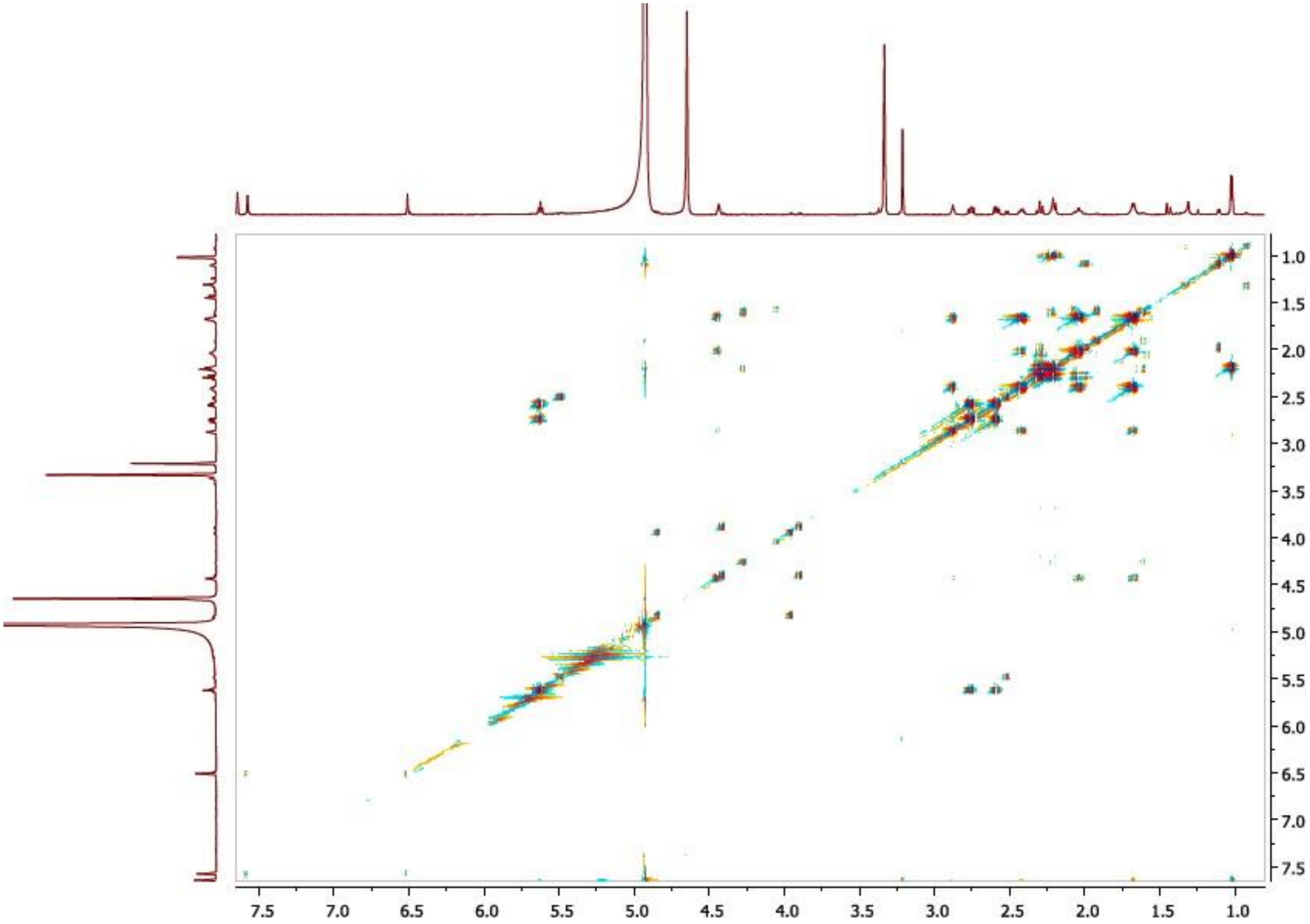

Figure S49. COSY NMR spectrum of **6** in CD_3OD.

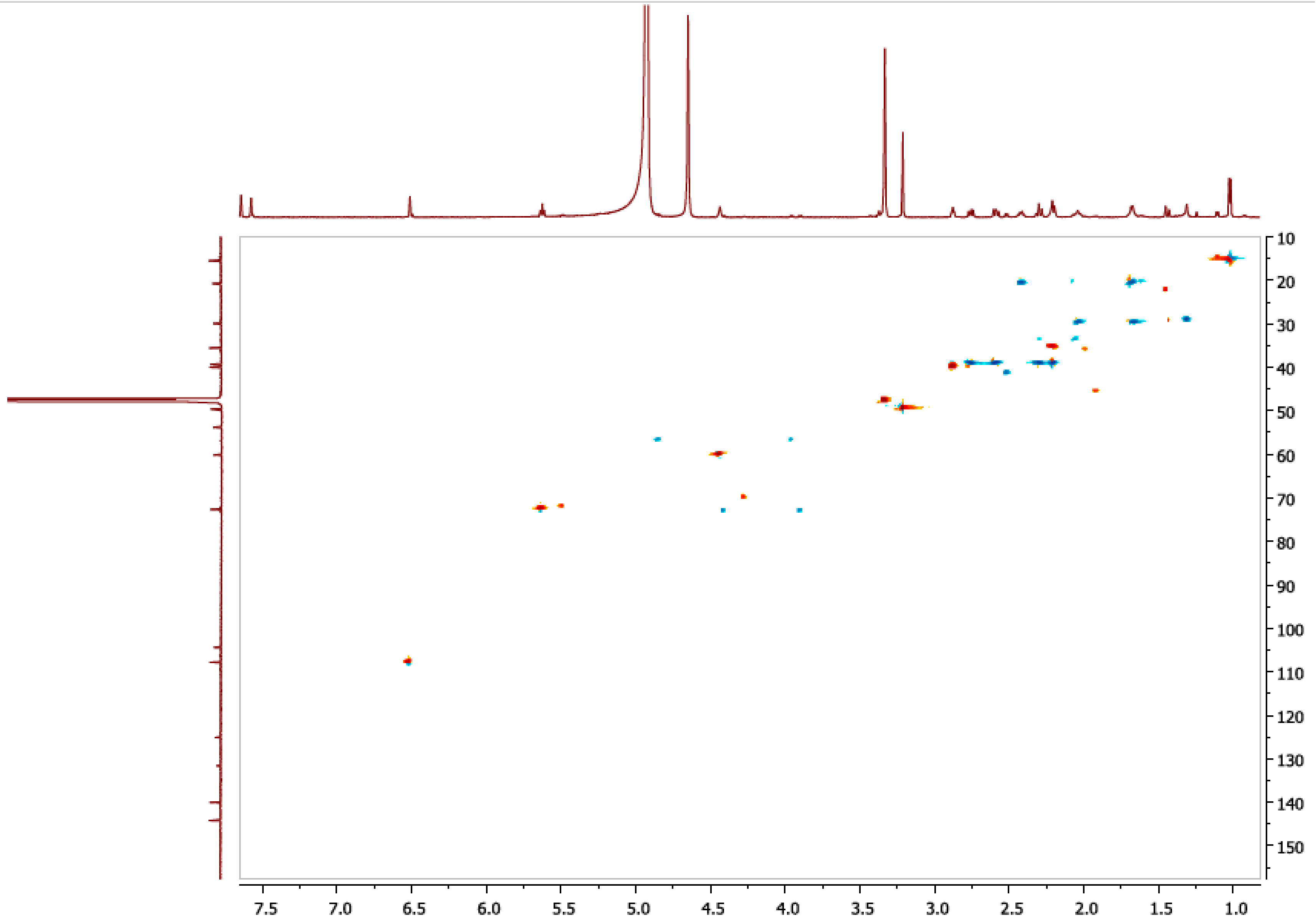

Figure S50. HSQC NMR spectrum of **6** in CD_3OD.

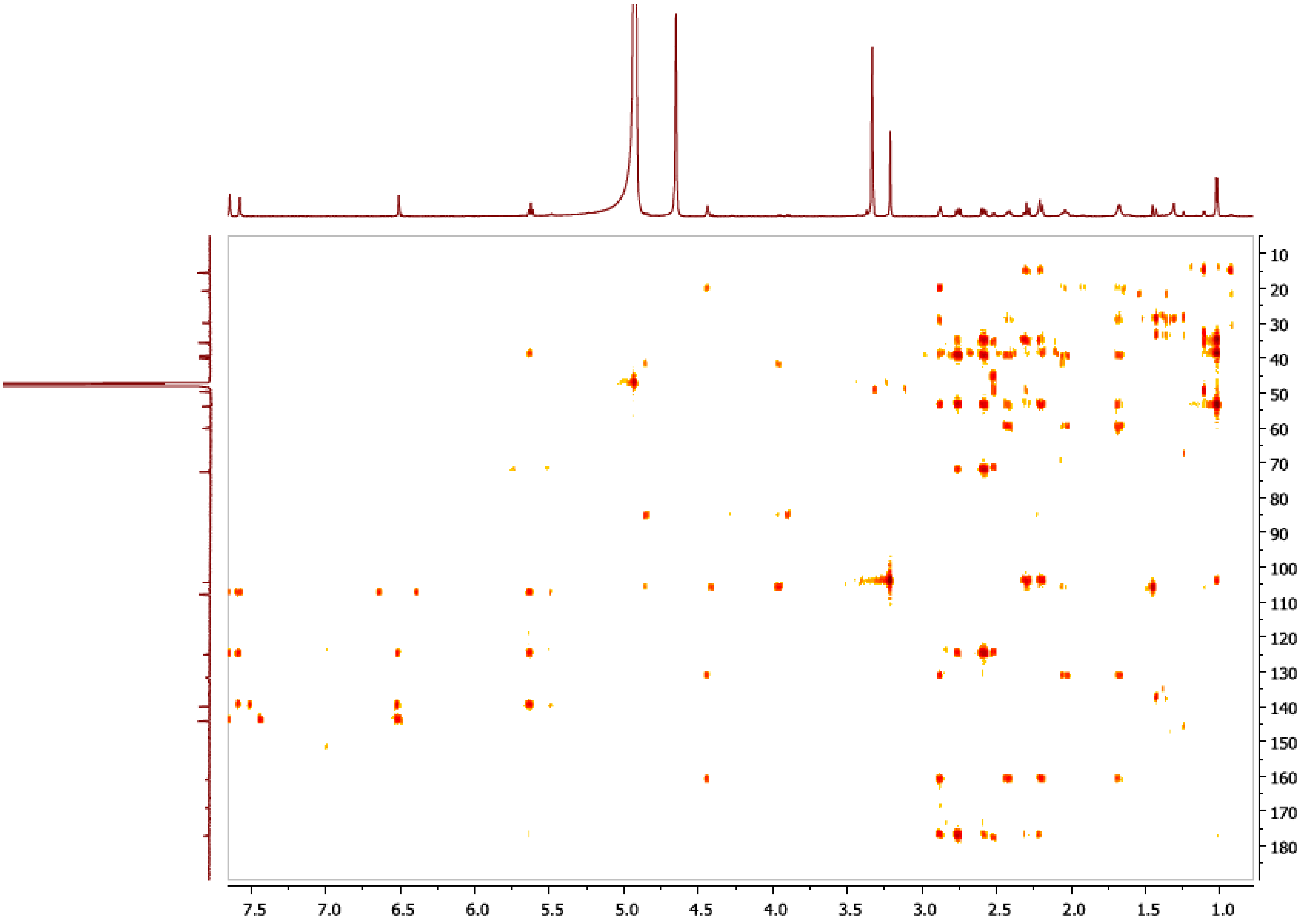

Figure S51. HMBC NMR spectrum of **6** in CD_3OD.

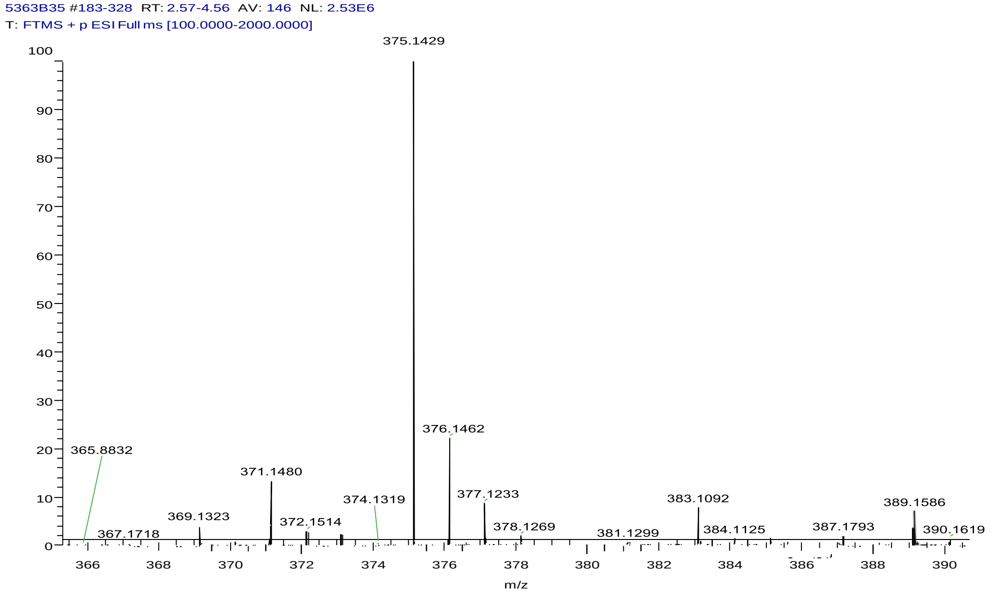
5363B35 #183-328 RT: 2.57-4.56 AV: 146 NL: 2.53E6
T: FTMS + p ESI Full ms [100.0000-2000.0000]
375.1429
376.1462
365.8832
371.1480
374.1319
377.1233
383.1092
389.1586
369.1323
372.1514
378.1269
367.1718
381.1299
384.1125
387.1793
390.1619
m/z

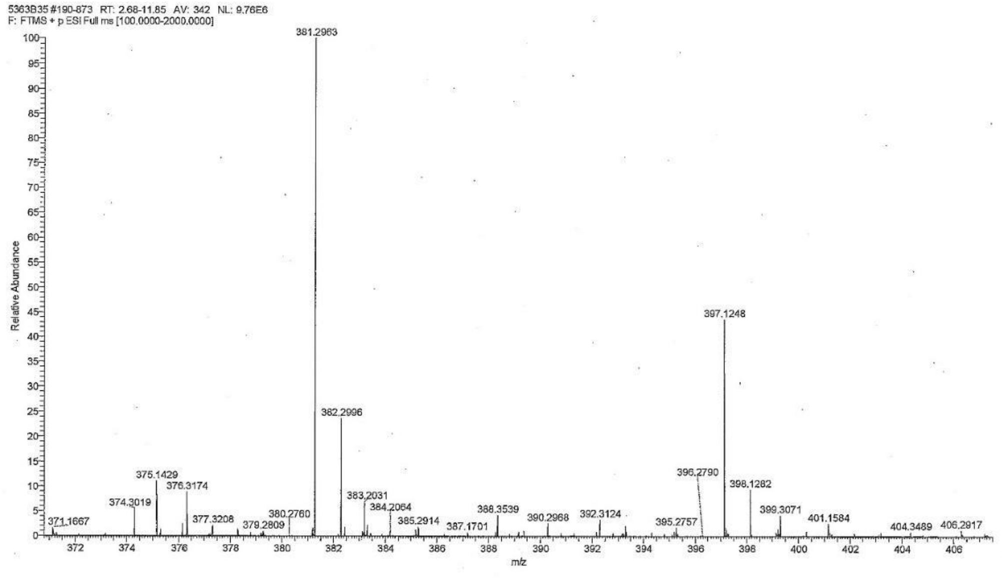
5363B35 #190-873 RT: 2.68-11.85 AV: 342 NL: 9.76E6
F: FTMS + p ESI Full ms [100.0000-2000.0000]
381.2983
397.1248
382.2996
396.2790
398.1282
375.1429
376.3174
374.3019
383.2031
384.2064
388.3539
392.3124
399.3071
401.1584
371.1667
377.3208
379.2809
380.2760
385.2914
387.1701
390.2968
395.2757
404.3489
406.2917
Relative Abundance
m/z

Figure S52. Positive-ion HRESIMS of **6**.

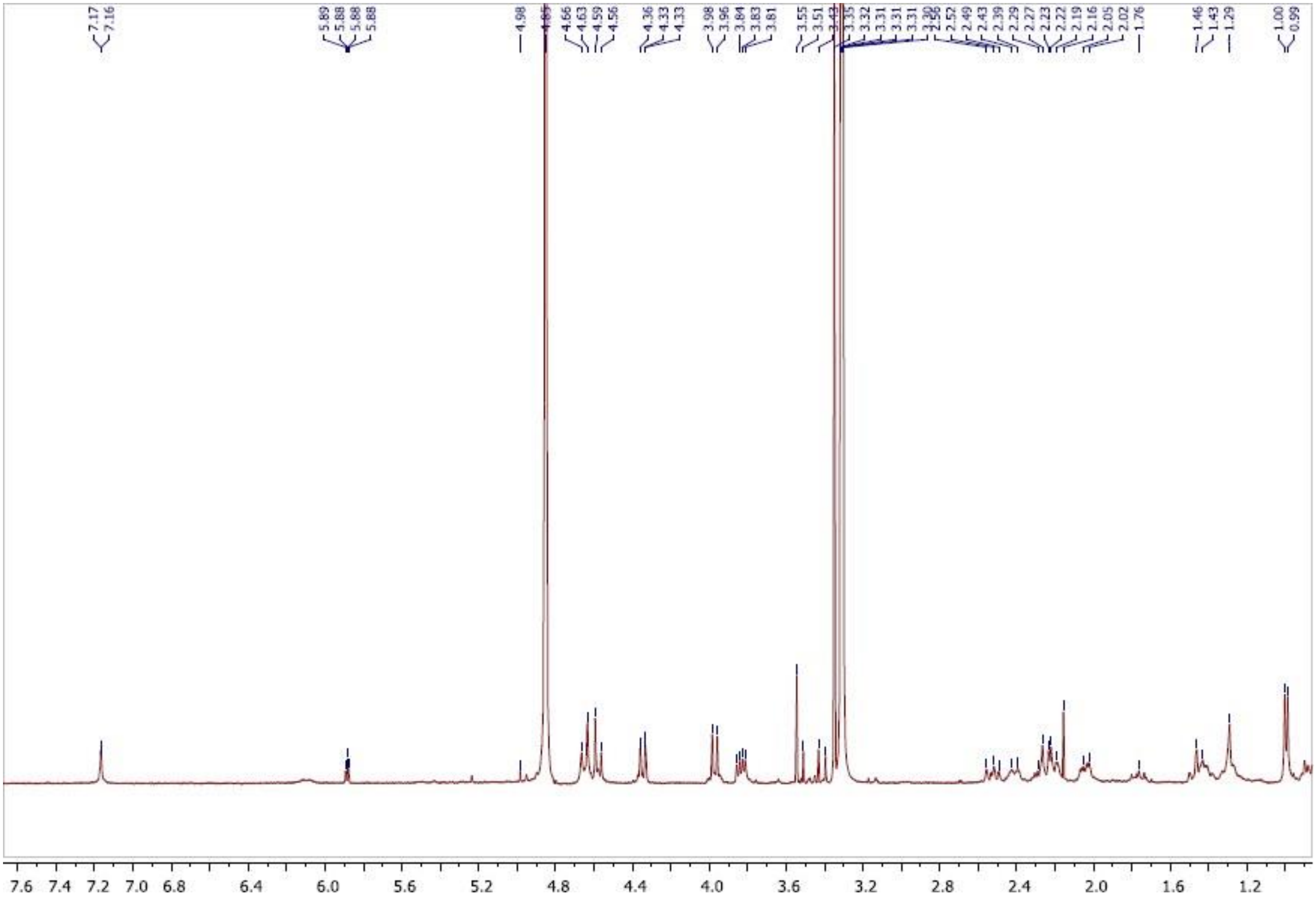

Figure S53. ^{1}H NMR spectrum of **7** in CD_3OD.

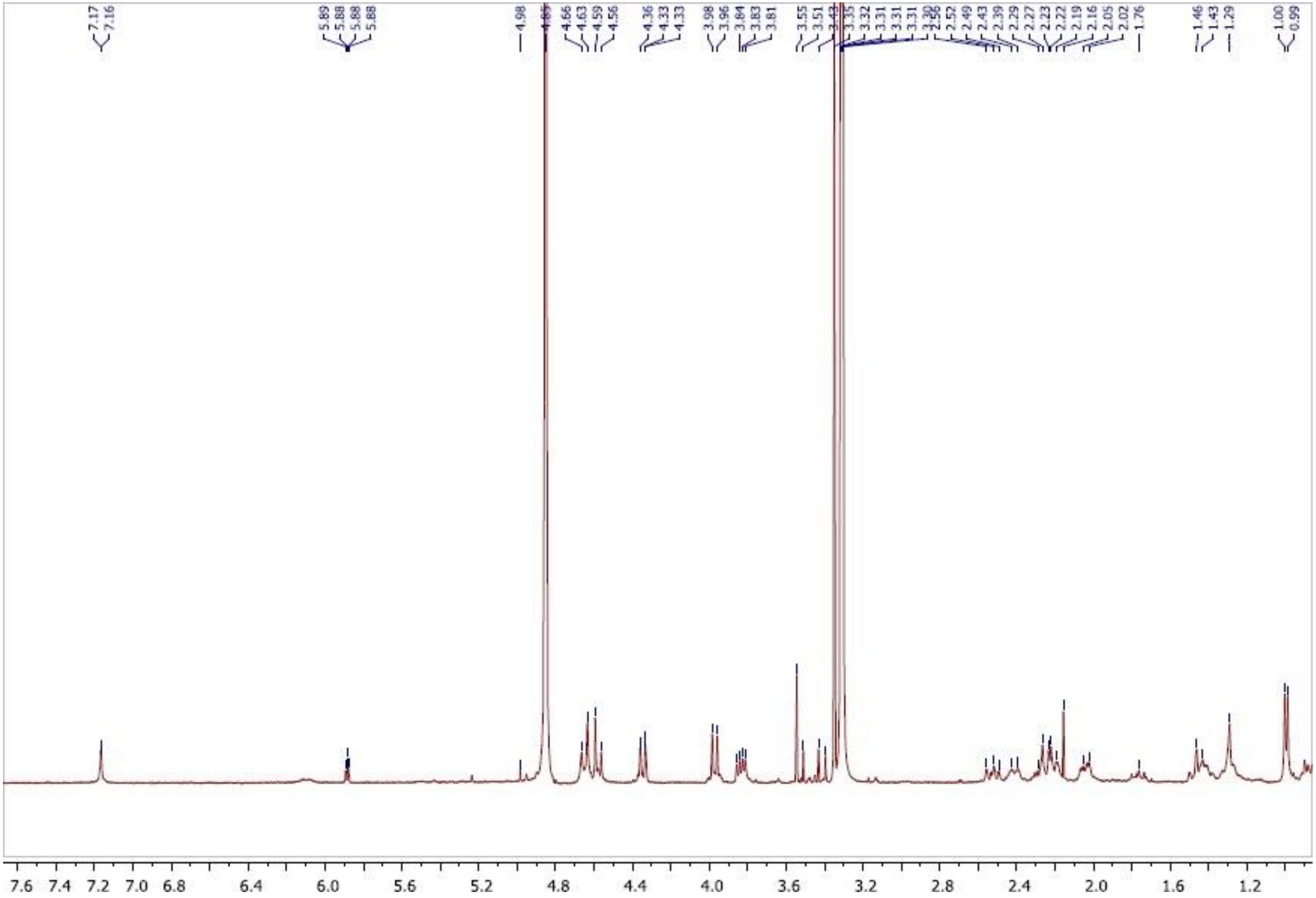

Figure S54. Expansion of part of ^{1}H NMR spectrum (δ 0.7 – δ 2.8) of **7** in CD_3OD.

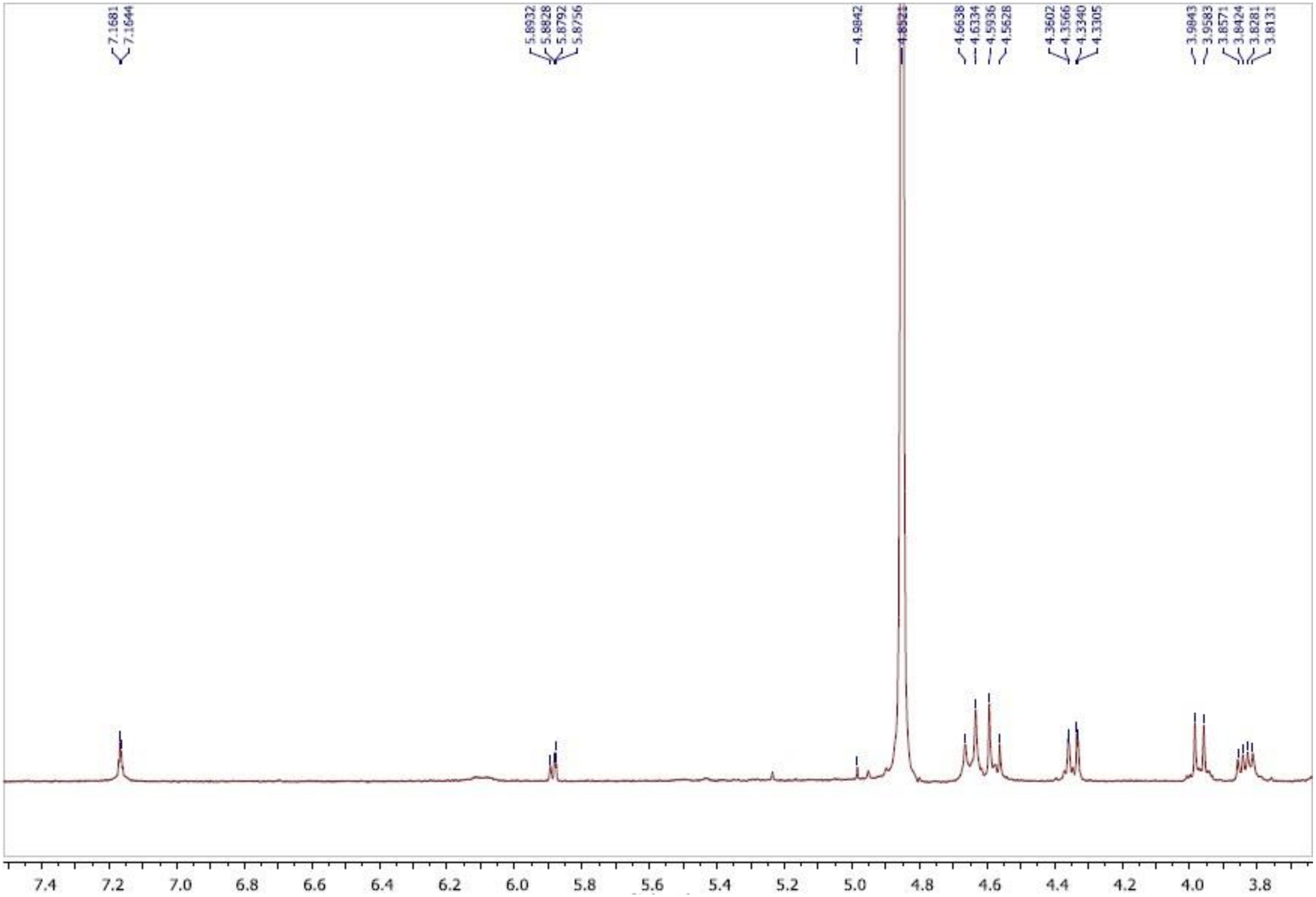

Figure S55. Expansion of part of ^{1}H NMR spectrum (δ 3.7 – δ 7.4) of **7** in CD_3OD.

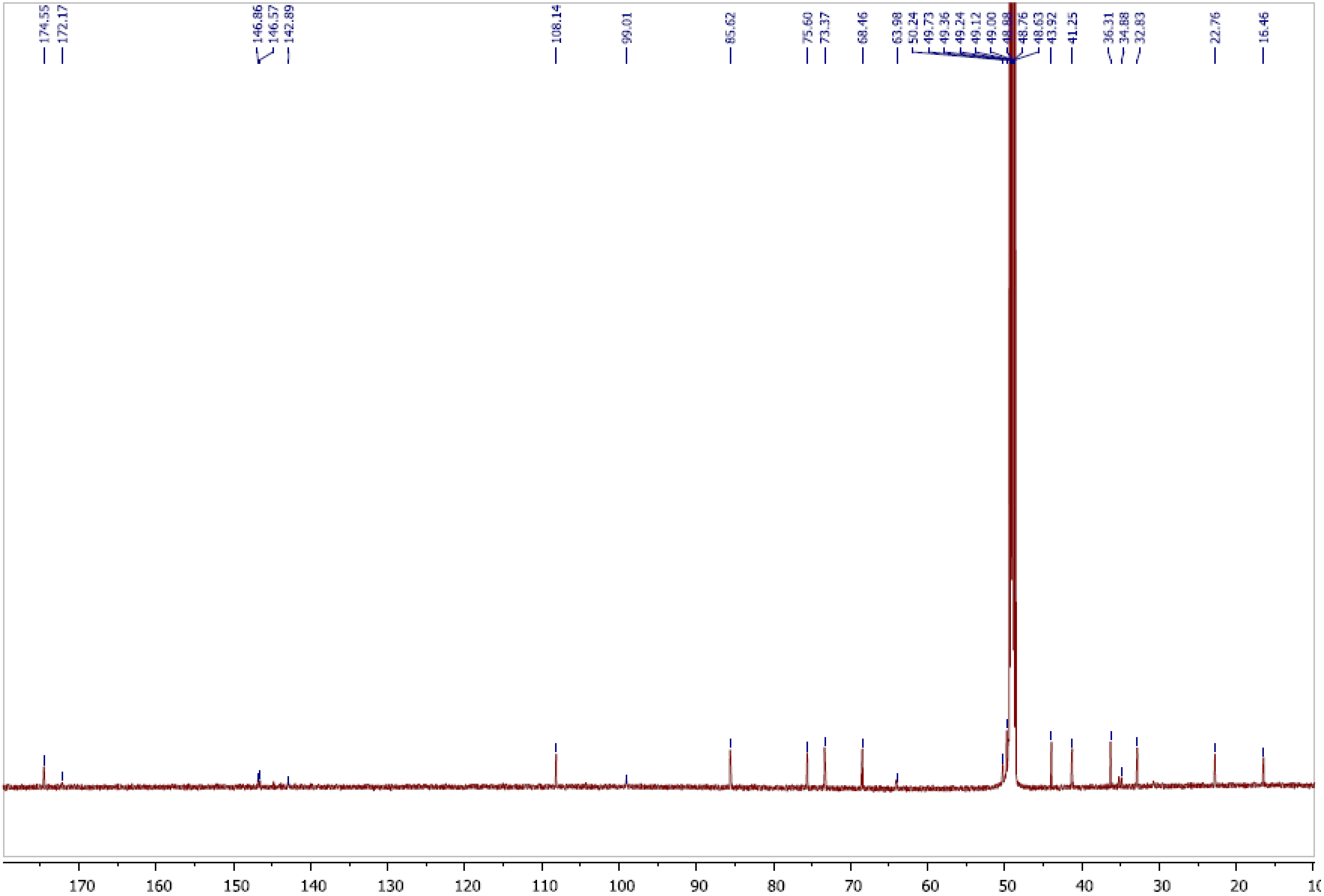

Figure S56. ^{13}C NMR spectrum of **7** in CD_3OD.

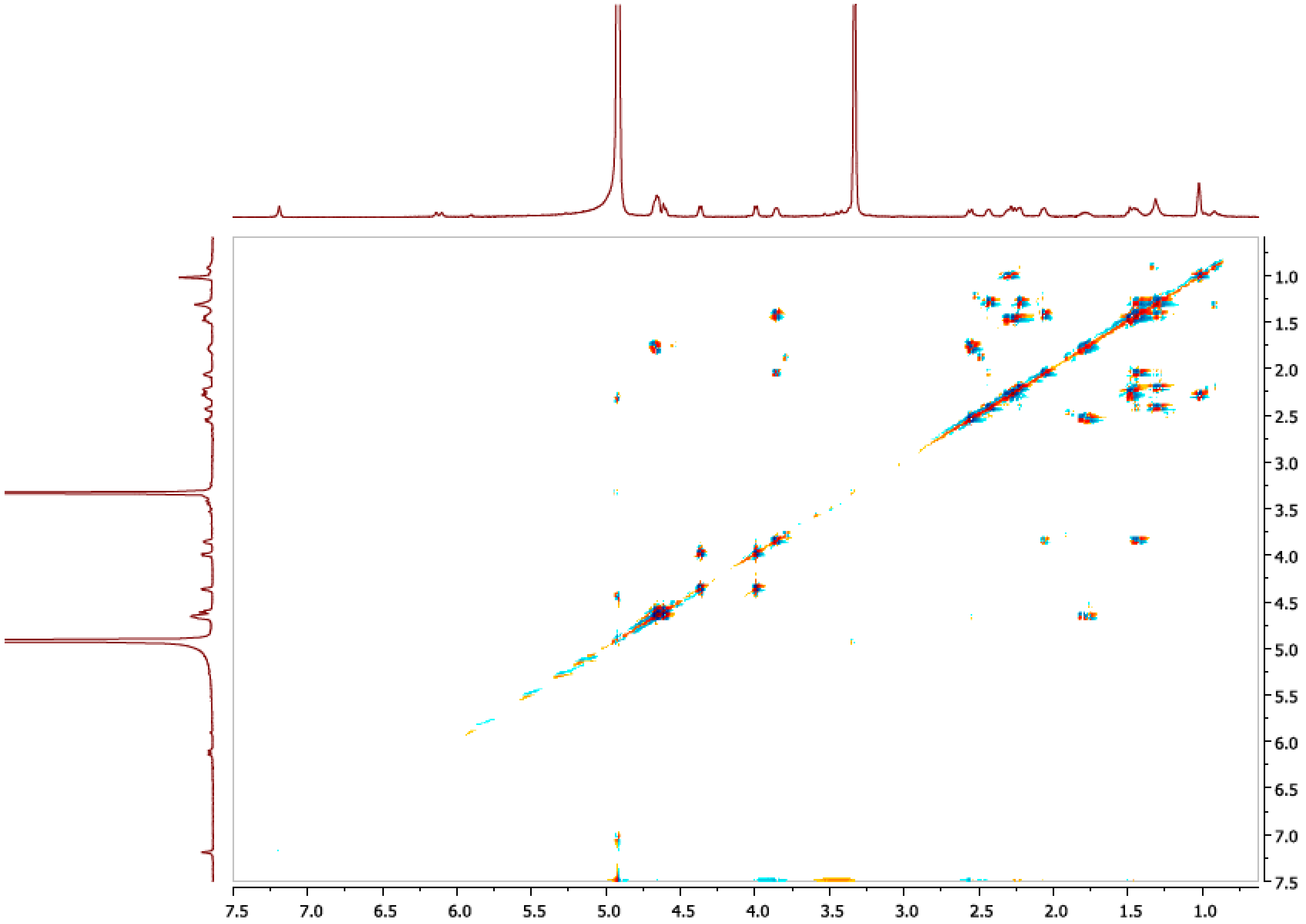

Figure S57. COSY NMR spectrum of **7** in CD_3OD.

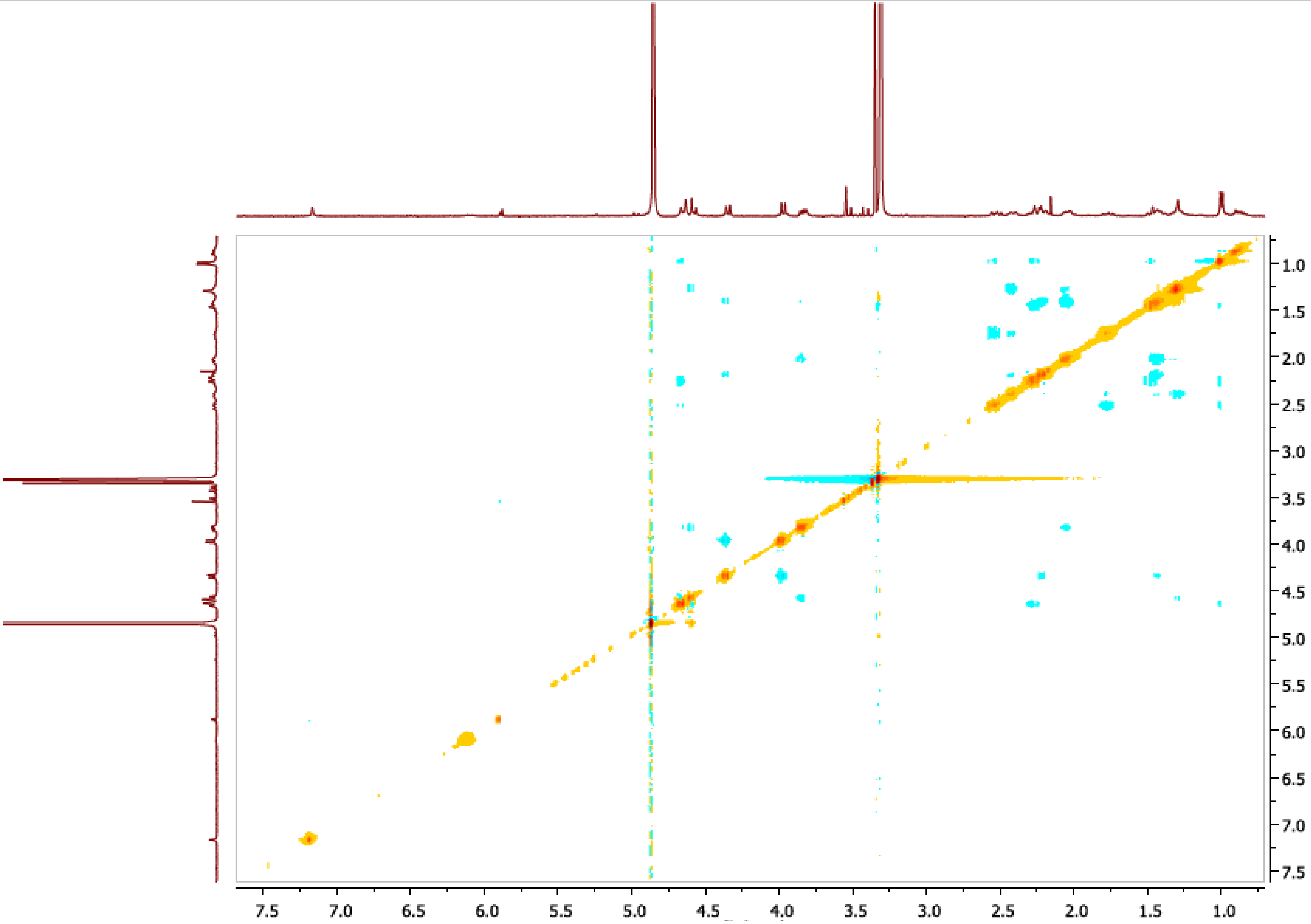

Figure S58. NOESY NMR spectrum of **7** in CD_3OD.

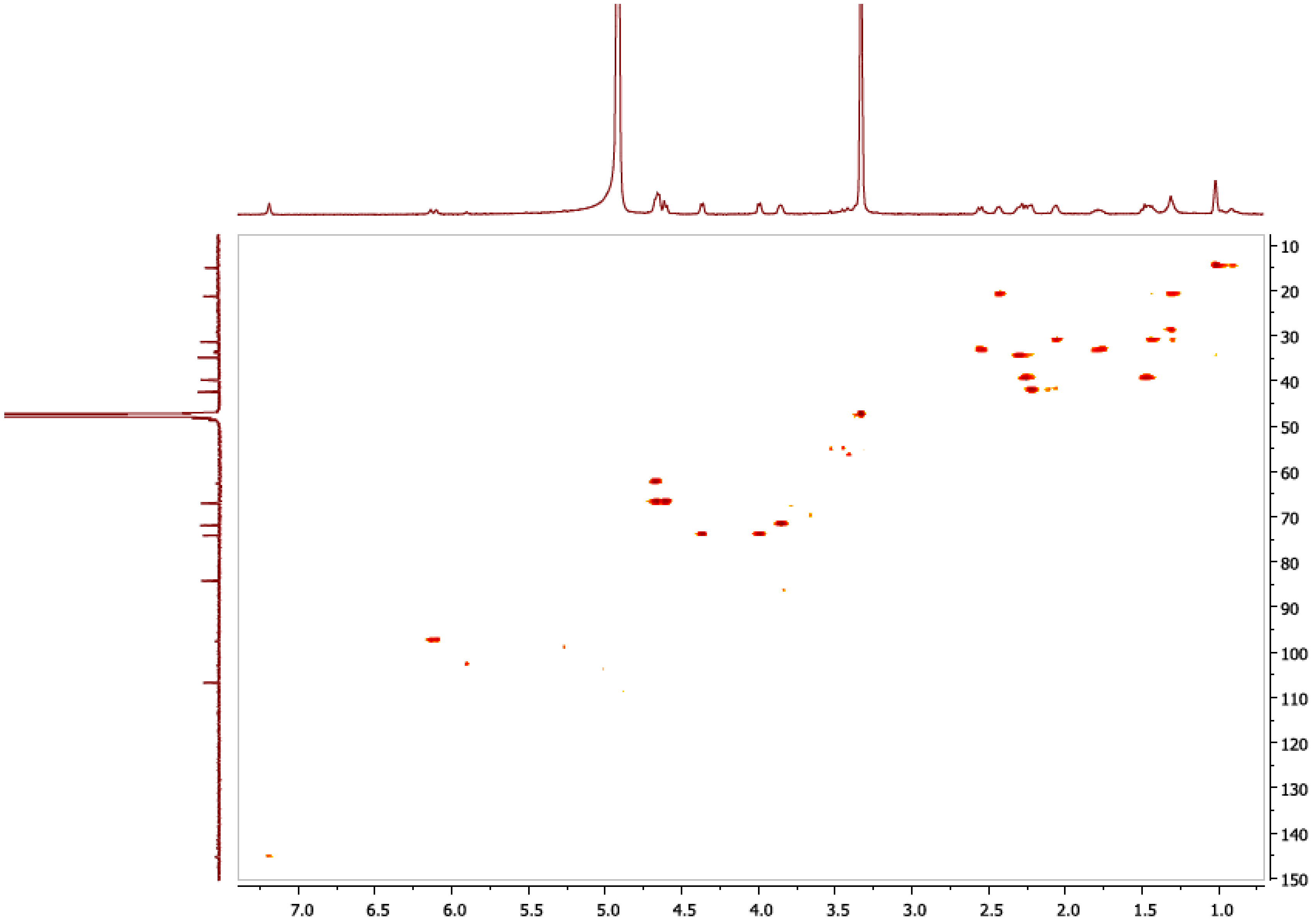

Figure S59. HSQC NMR spectrum of **7** in CD_3OD.

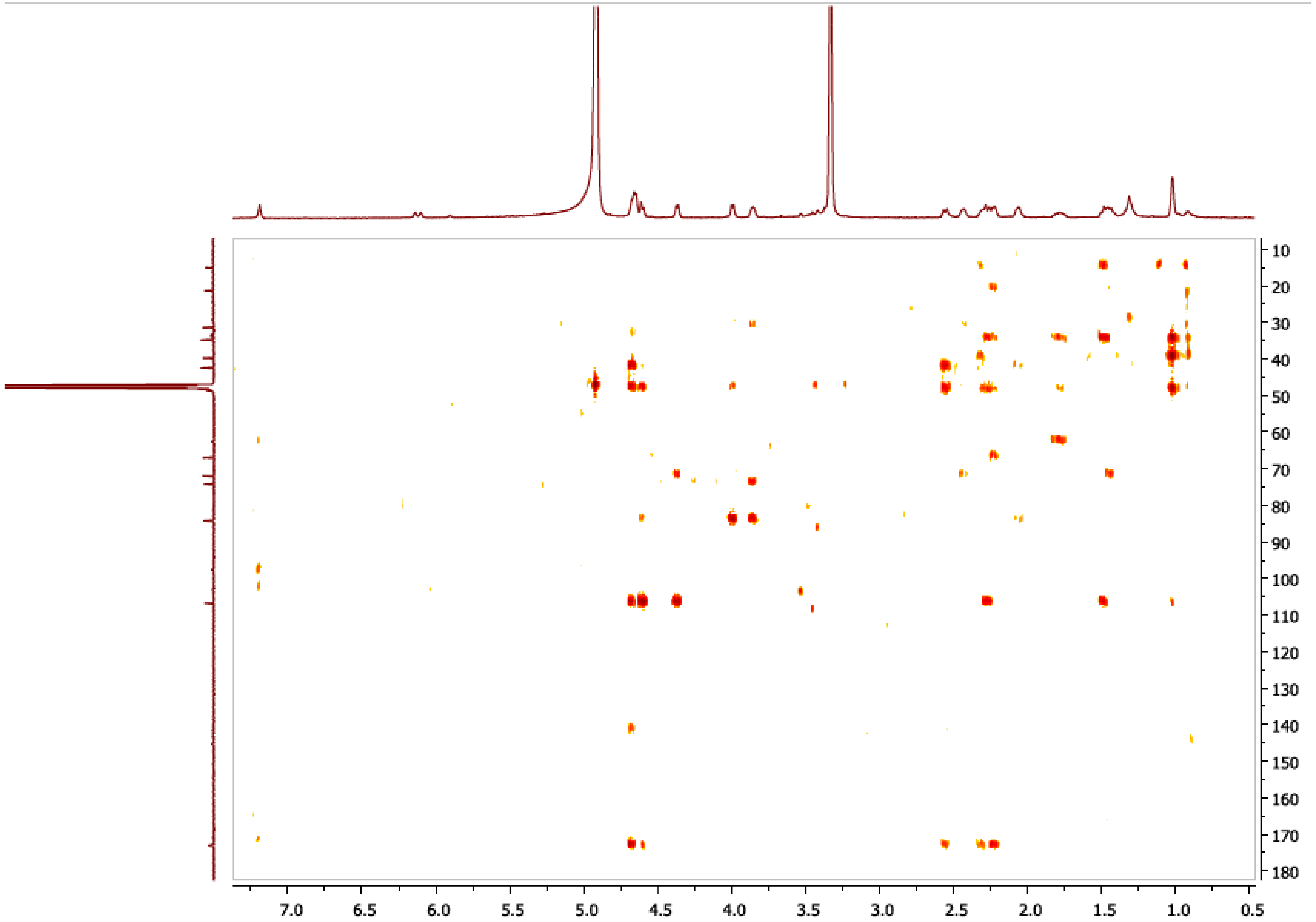

Figure S60. HMBC NMR spectrum of **7** in CD_3OD.

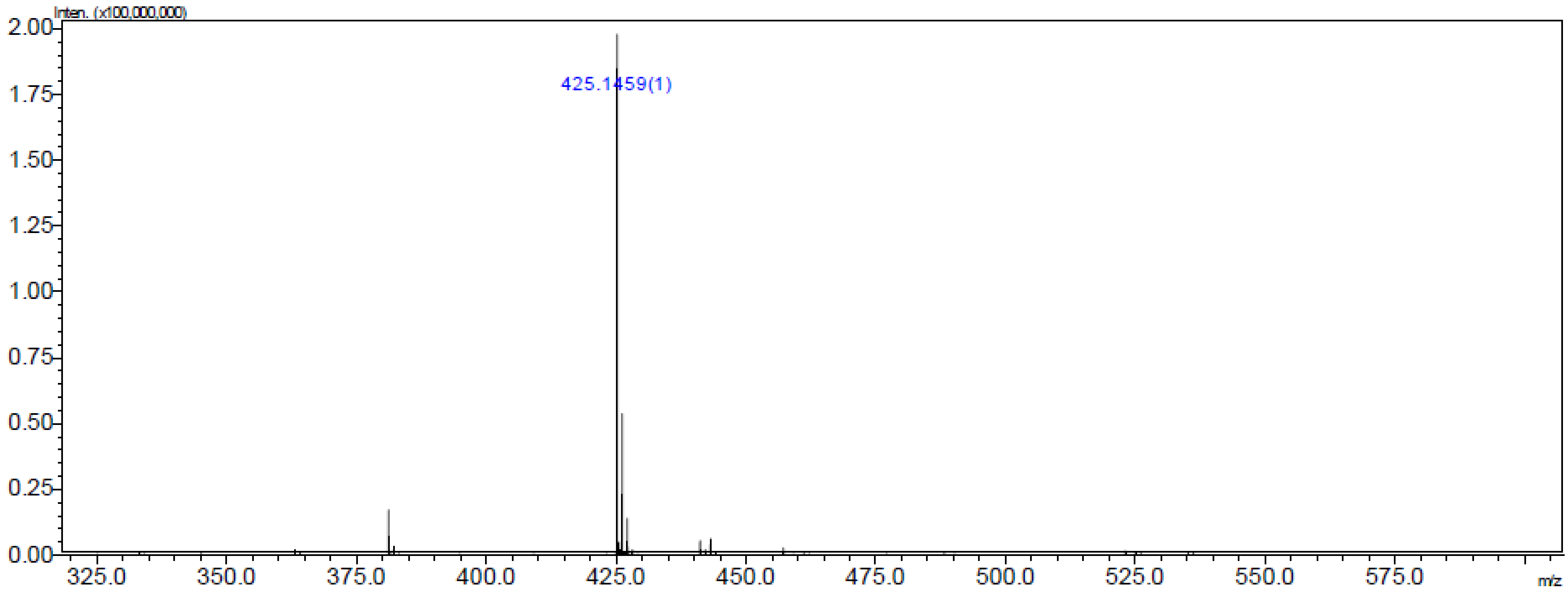

Figure S61. Negative-ion HRESIMS of **7**.

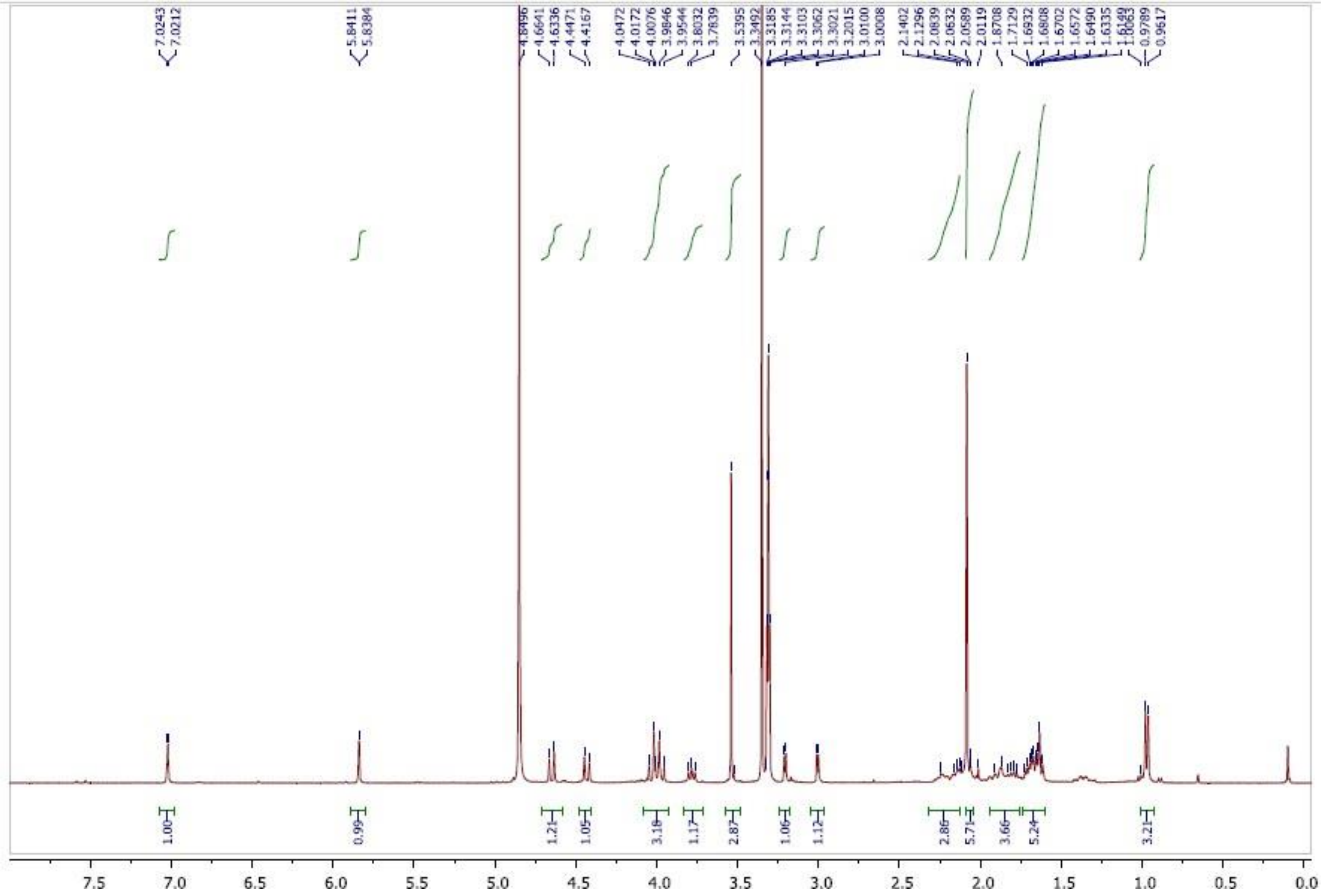

Figure S62. ^{1}H NMR spectrum of **8** in CD_3OD.

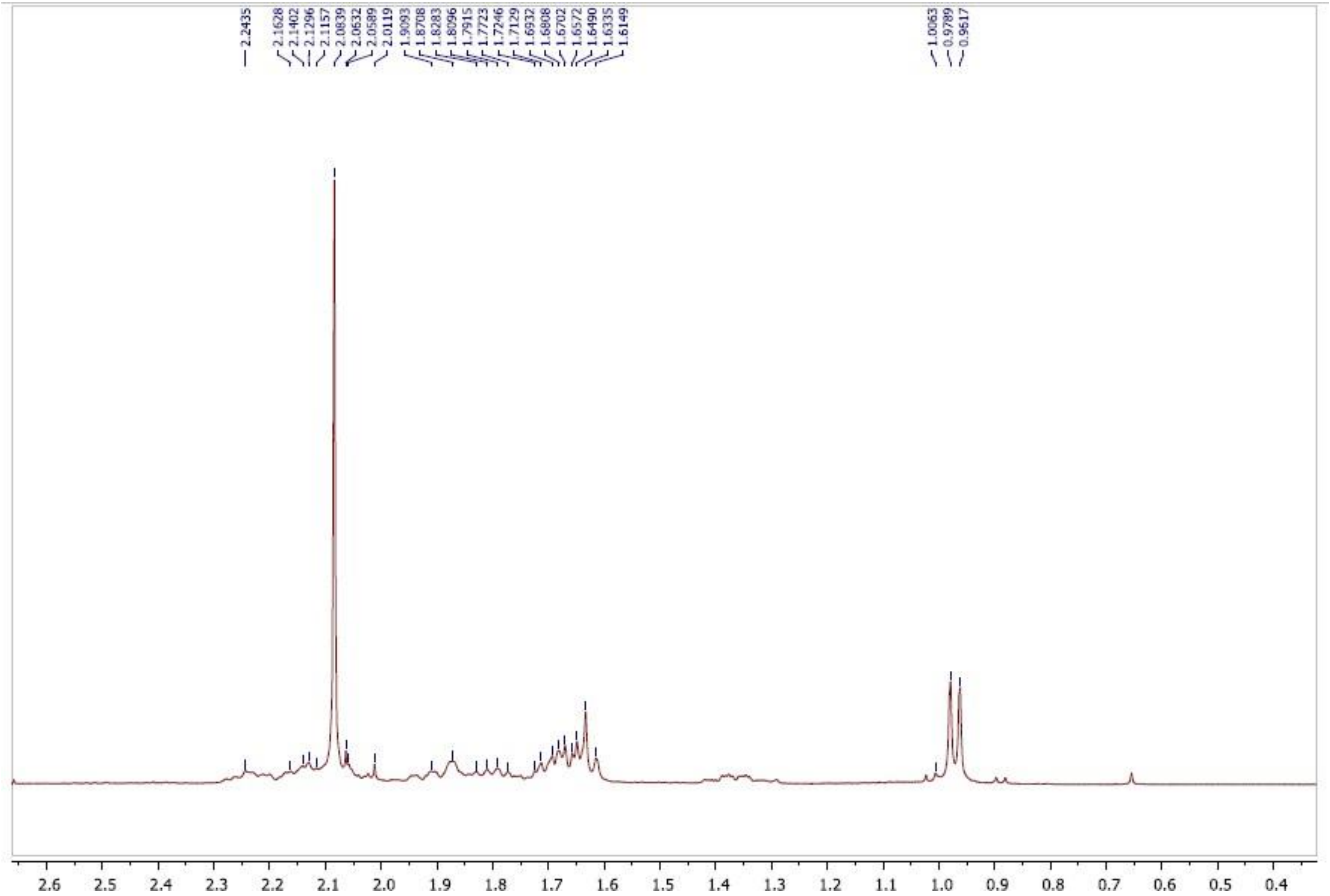

Figure S63. Expansion of part of ^{1}H NMR spectrum (δ 0.4 – δ 2.6) of **8** in CD_3OD.

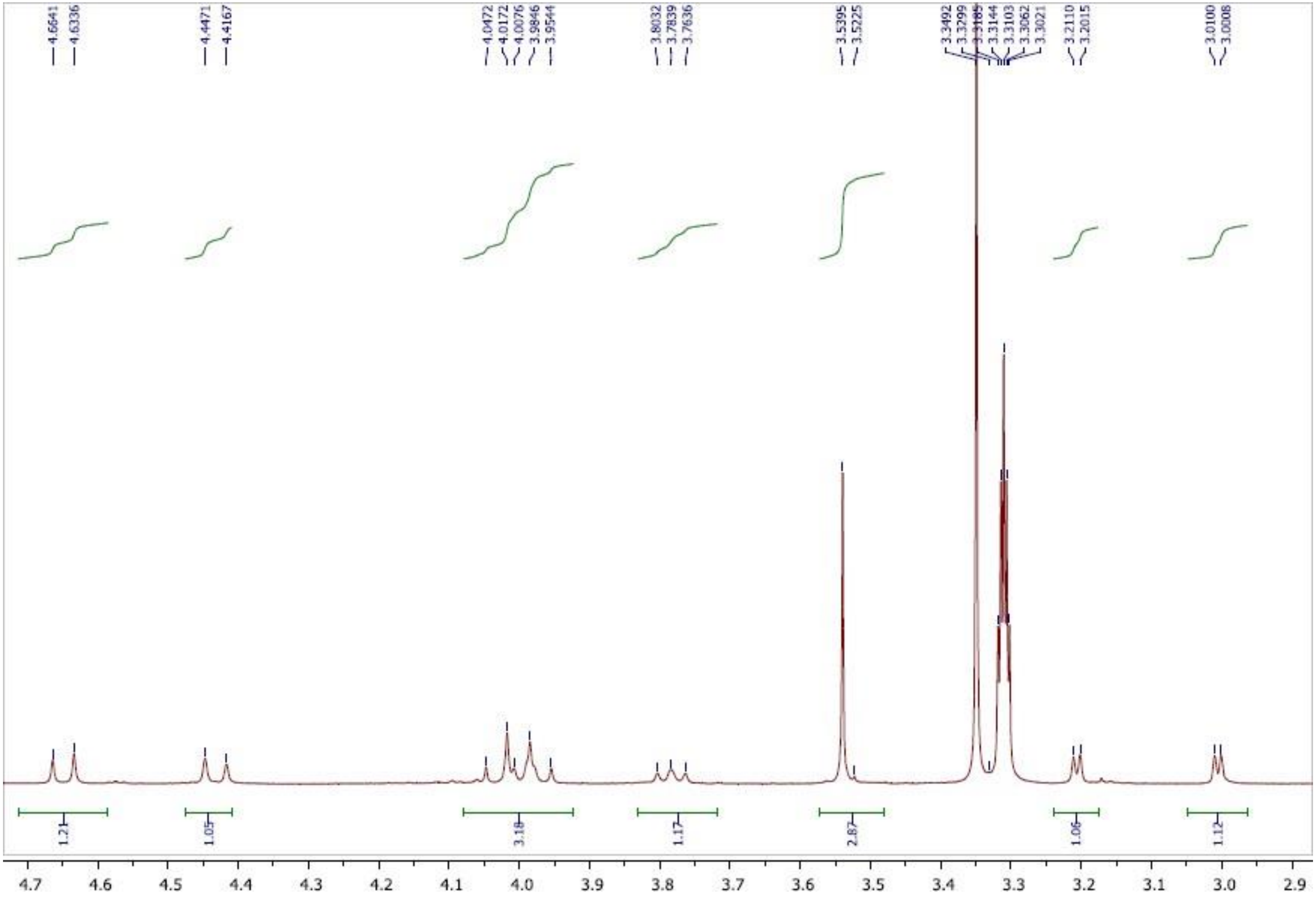

Figure S64. Expansion of part of ^{1}H NMR spectrum (δ 2.9 – δ 4.7) of **8** in CD_3OD.

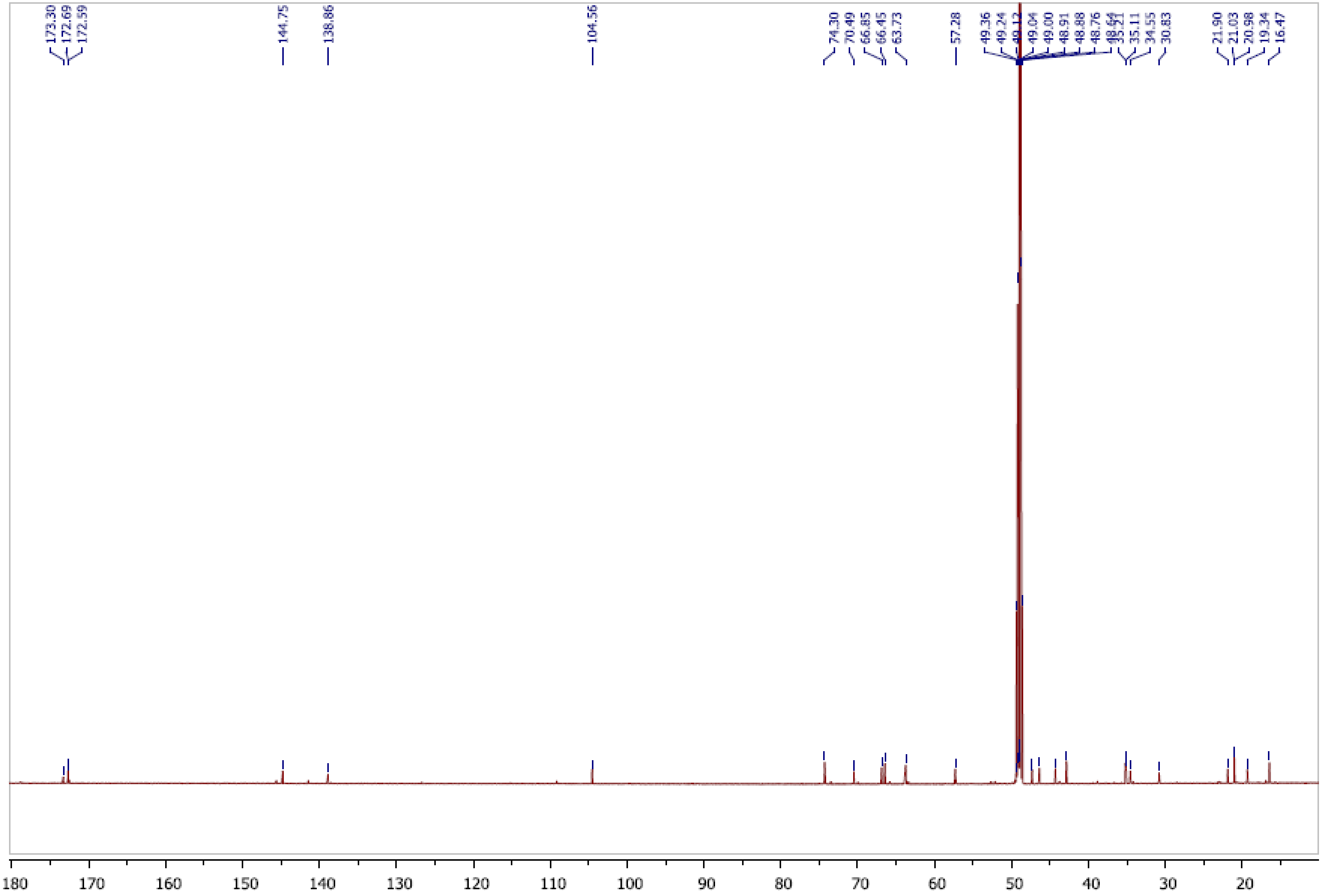

Figure S65. ^{13}C NMR spectrum of **8** in CD_3OD.

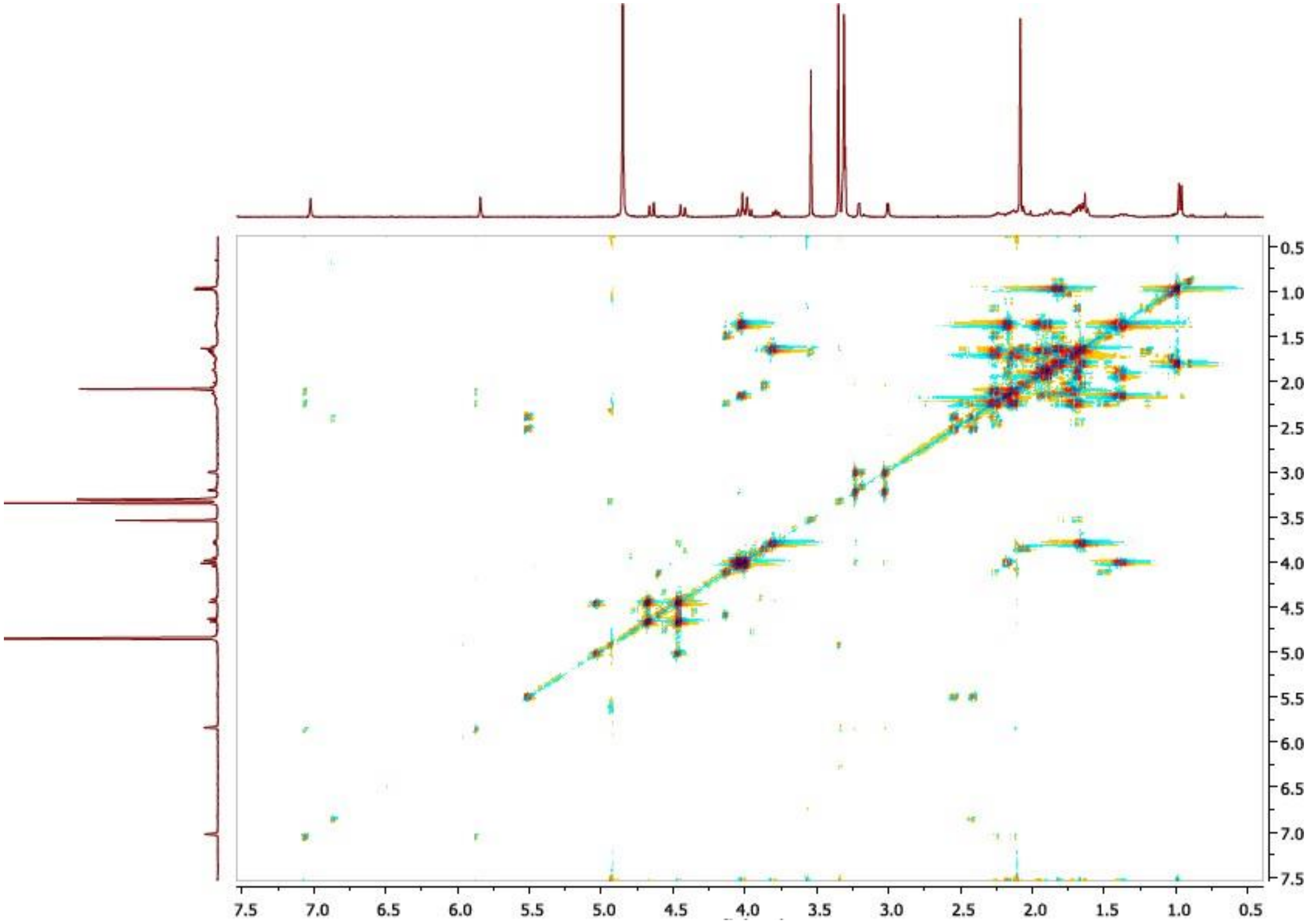

Figure S66. COSY NMR spectrum of **8** in CD_3OD.

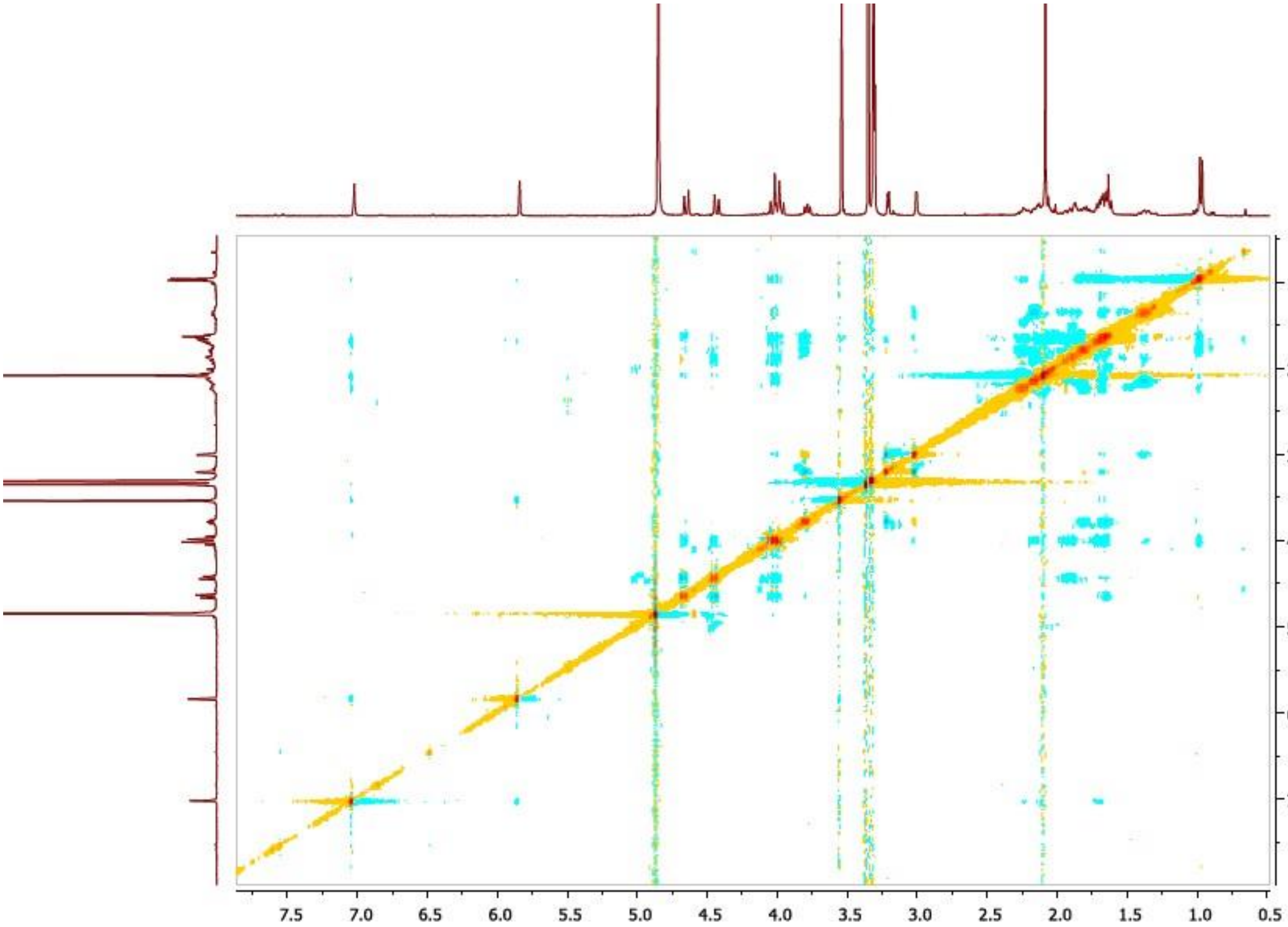

Figure S67. NOESY NMR spectrum of **8** in CD_3OD.

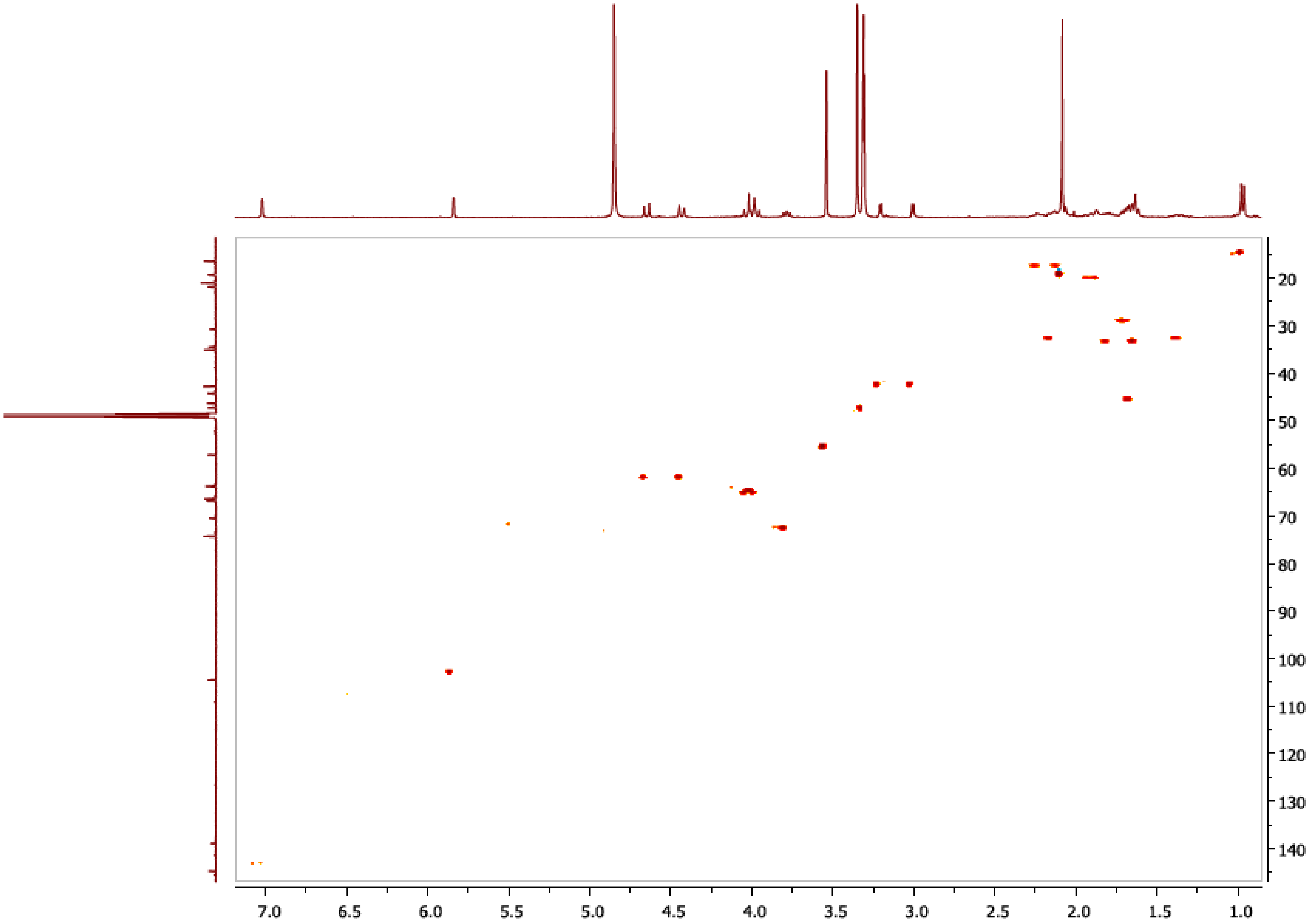

Figure S68. HSQC NMR spectrum of **8** in CD_3OD.

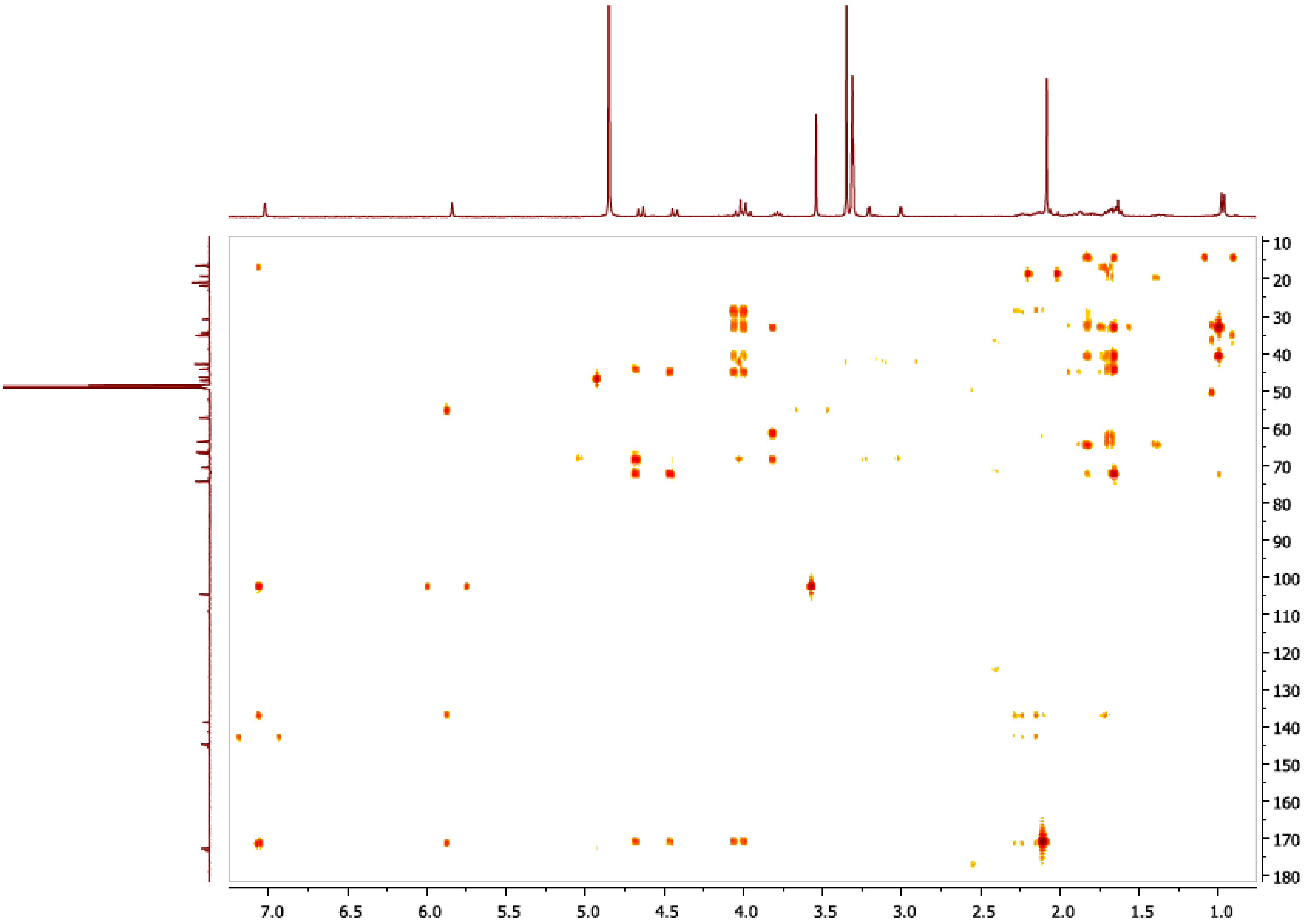

Figure S69. HMBC NMR spectrum of **8** in CD_3OD.

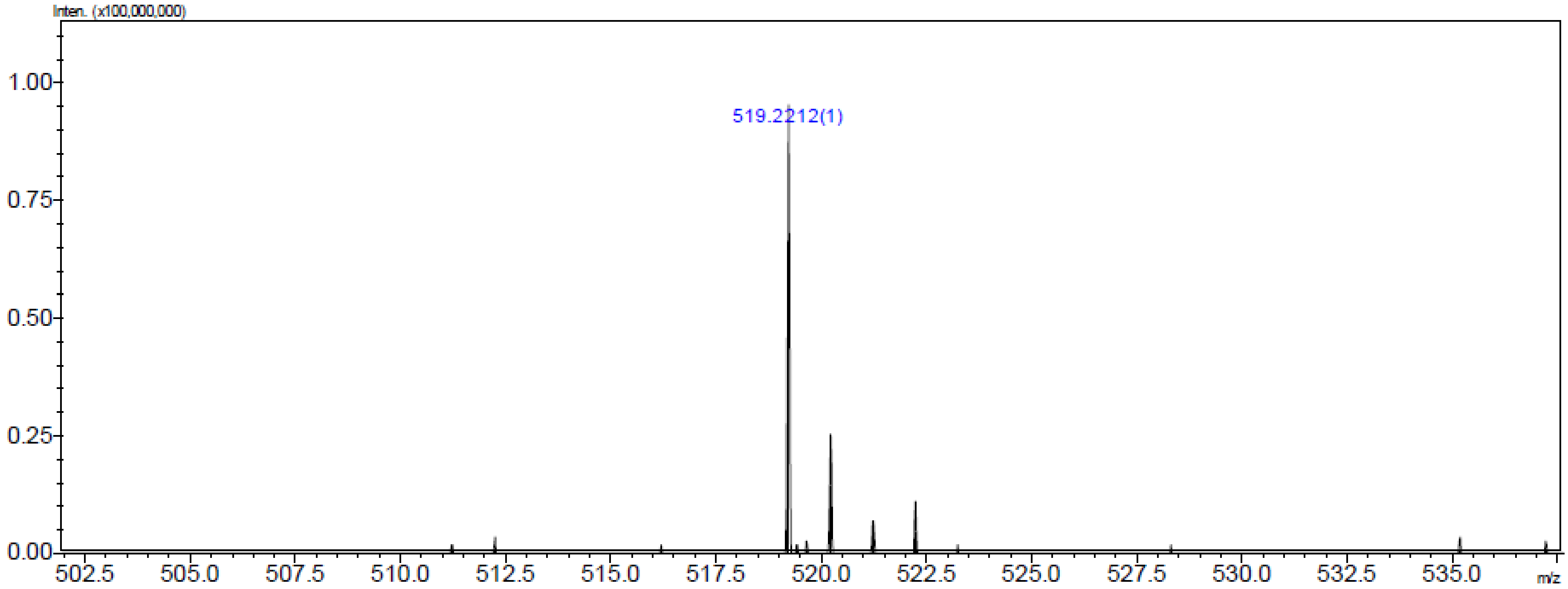

Figure S70. Positive-ion HRESIMS of **8**.

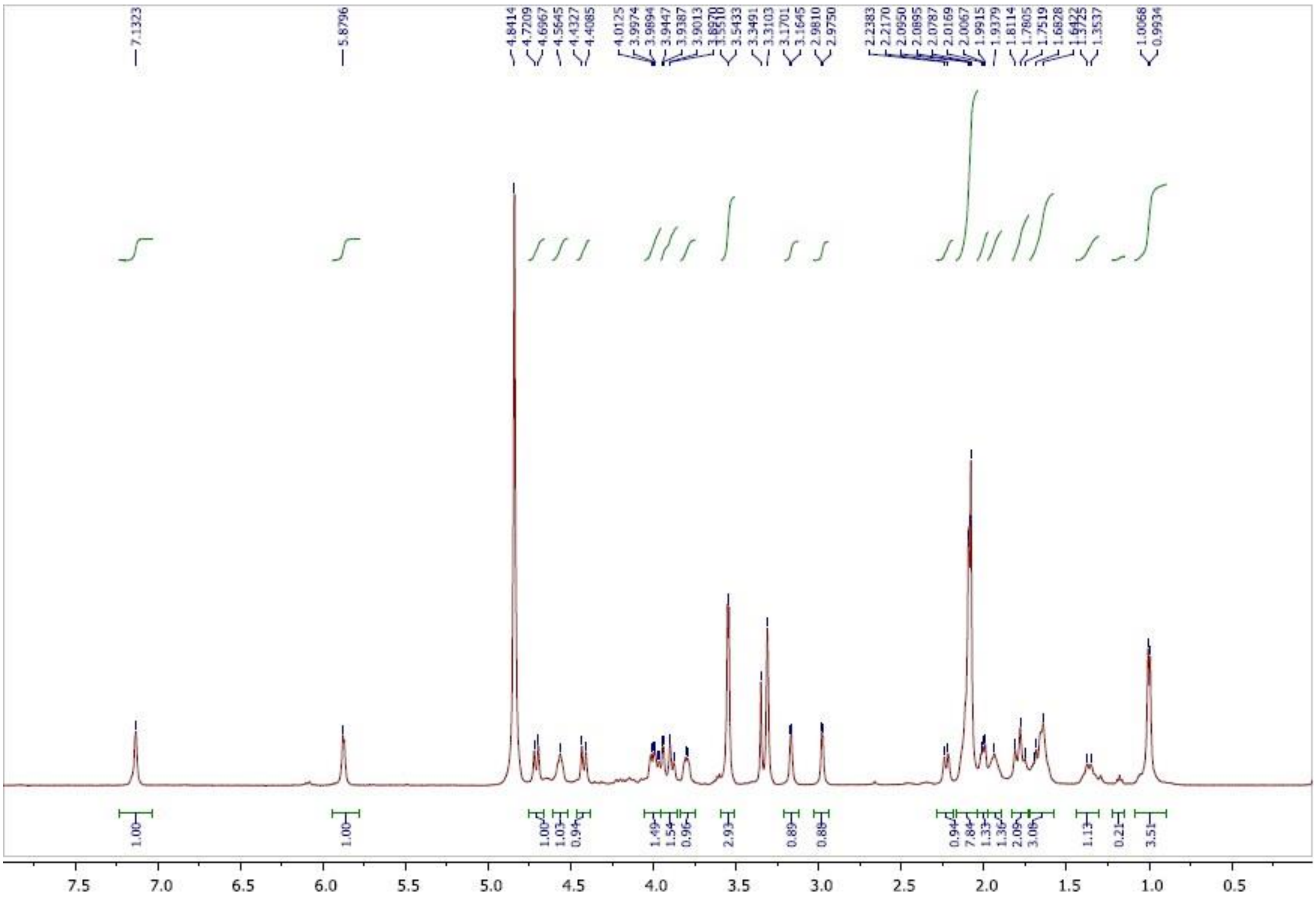

Figure S71. ^{1}H NMR spectrum of **9** in CD_3OD.

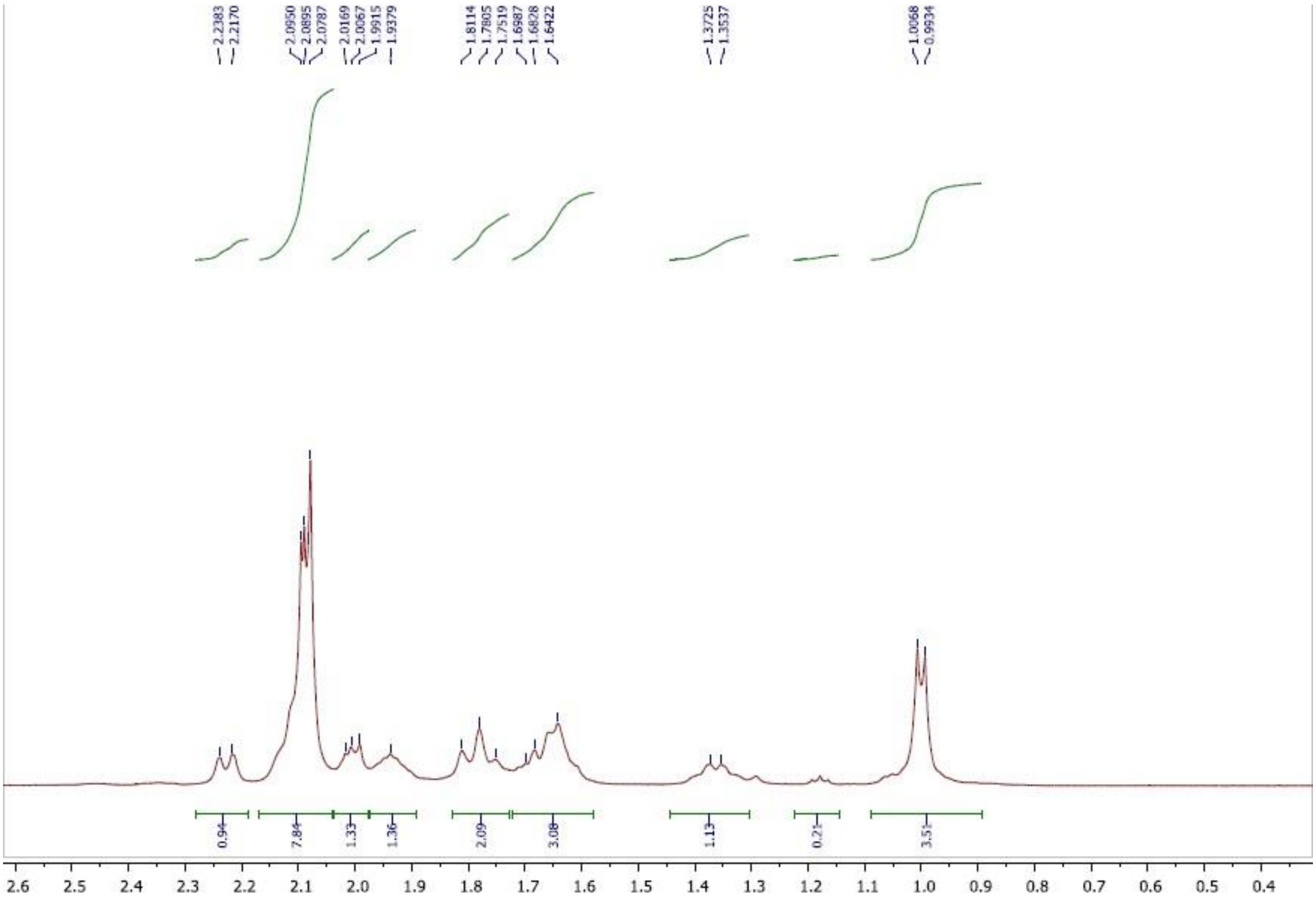

Figure S72. Expansion of part of ^{1}H NMR spectrum (δ 0.4 – δ 2.6) of **9** in CD_3OD.

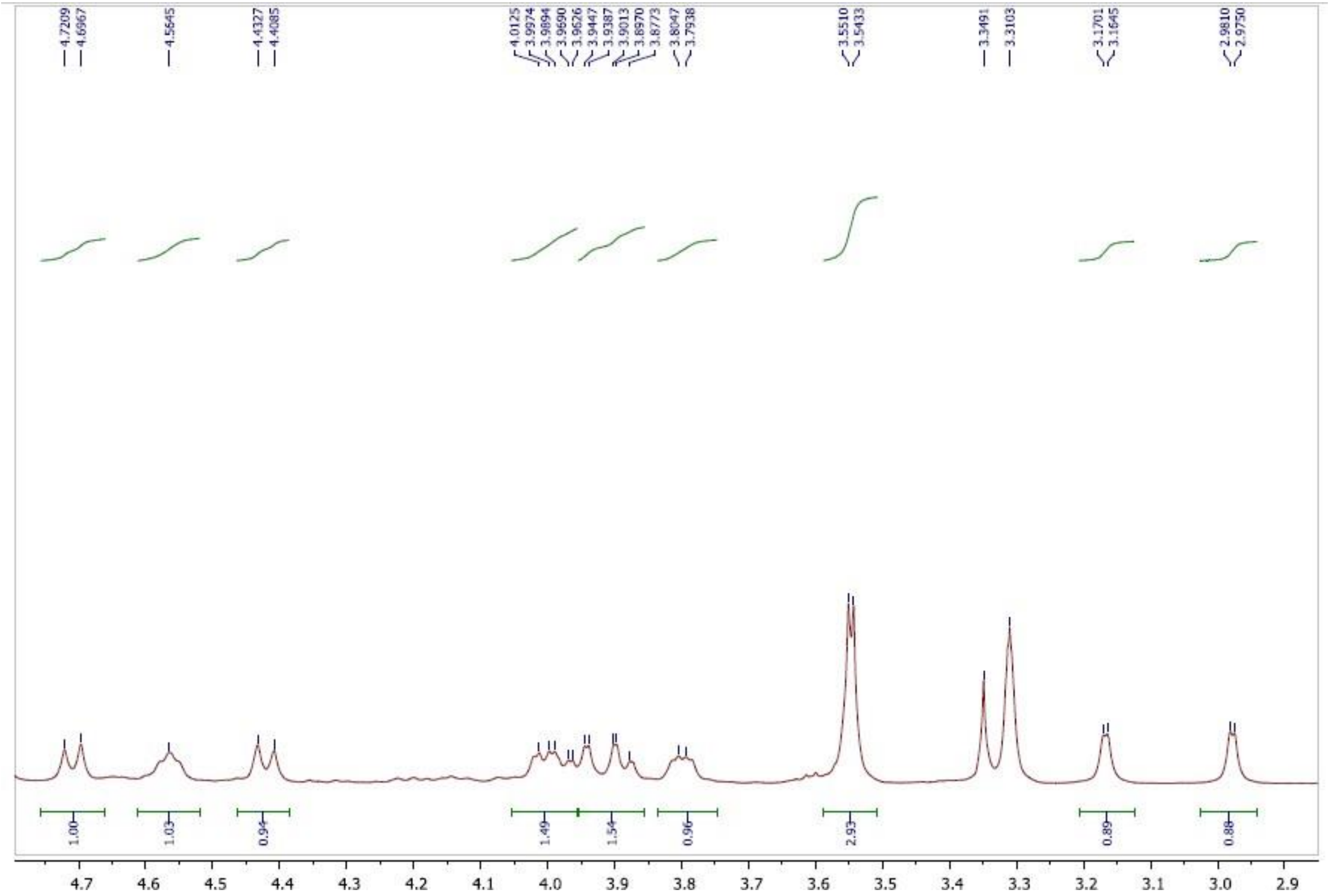

Figure S73. Expansion of part of ^{1}H NMR spectrum (δ 2.9 – δ 4.8) of **9** in CD_3OD.

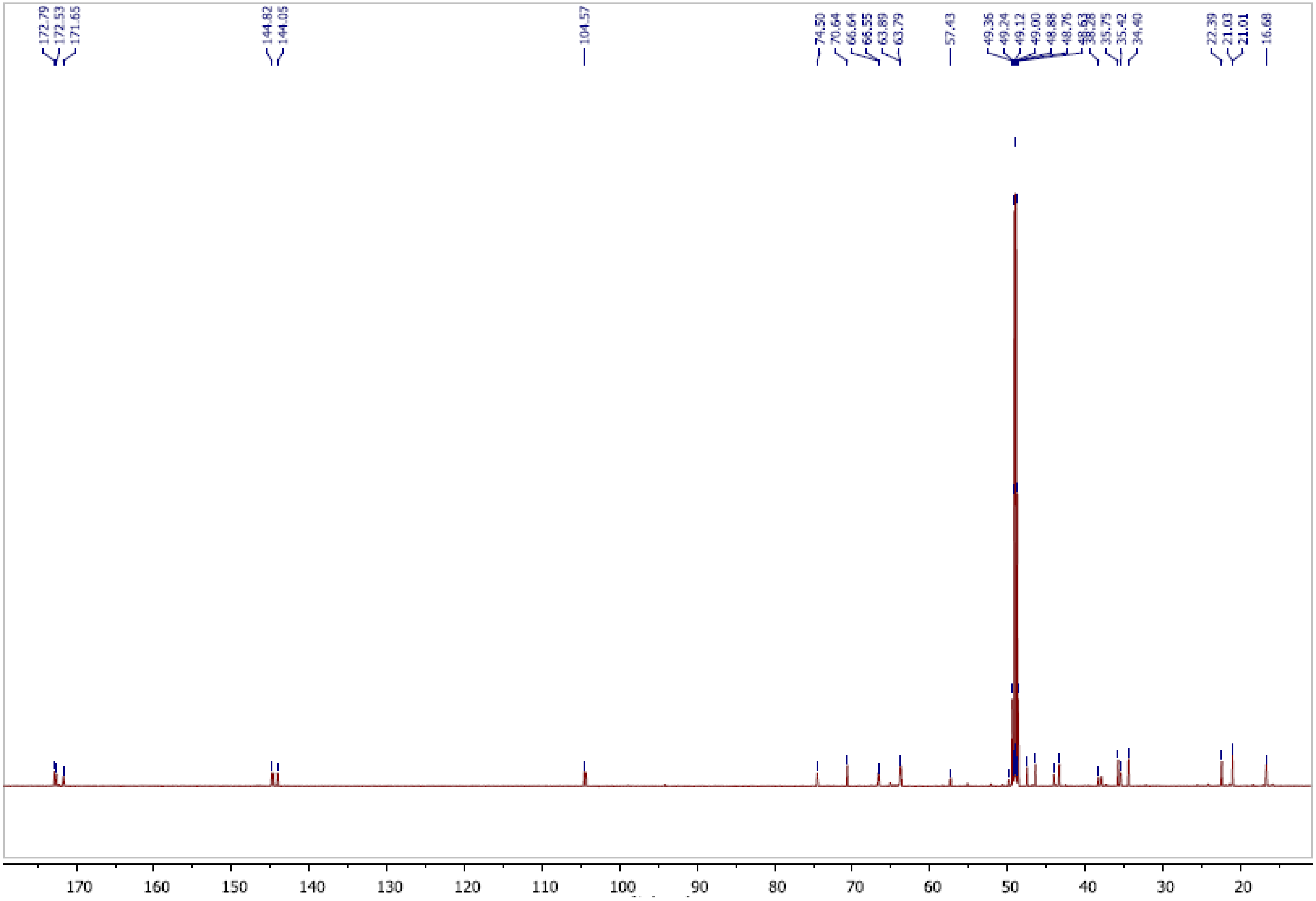

Figure S74. ^{13}C NMR spectrum of **9** in CD_3OD.

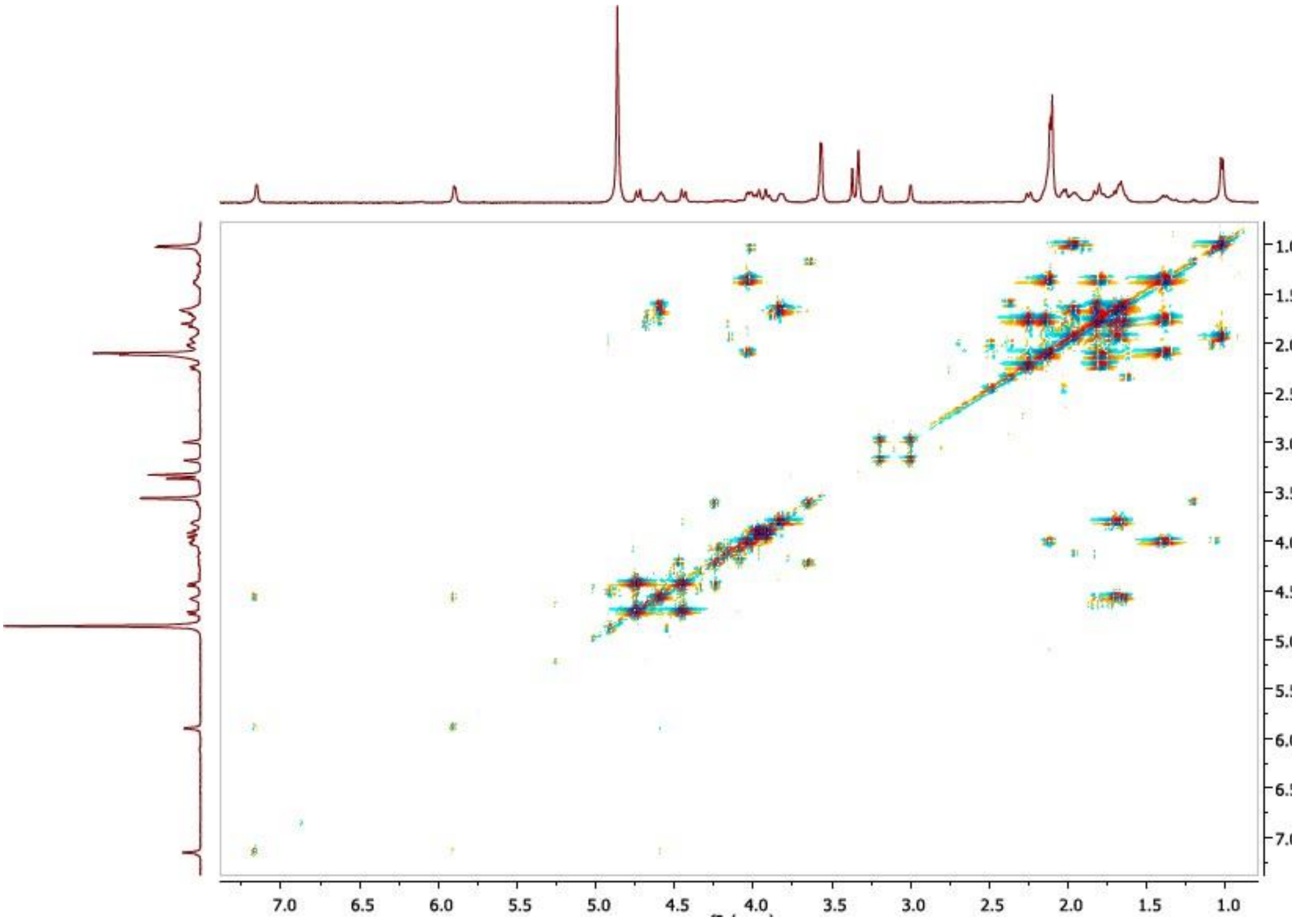

Figure S75. COSY NMR spectrum of **9** in CD_3OD.

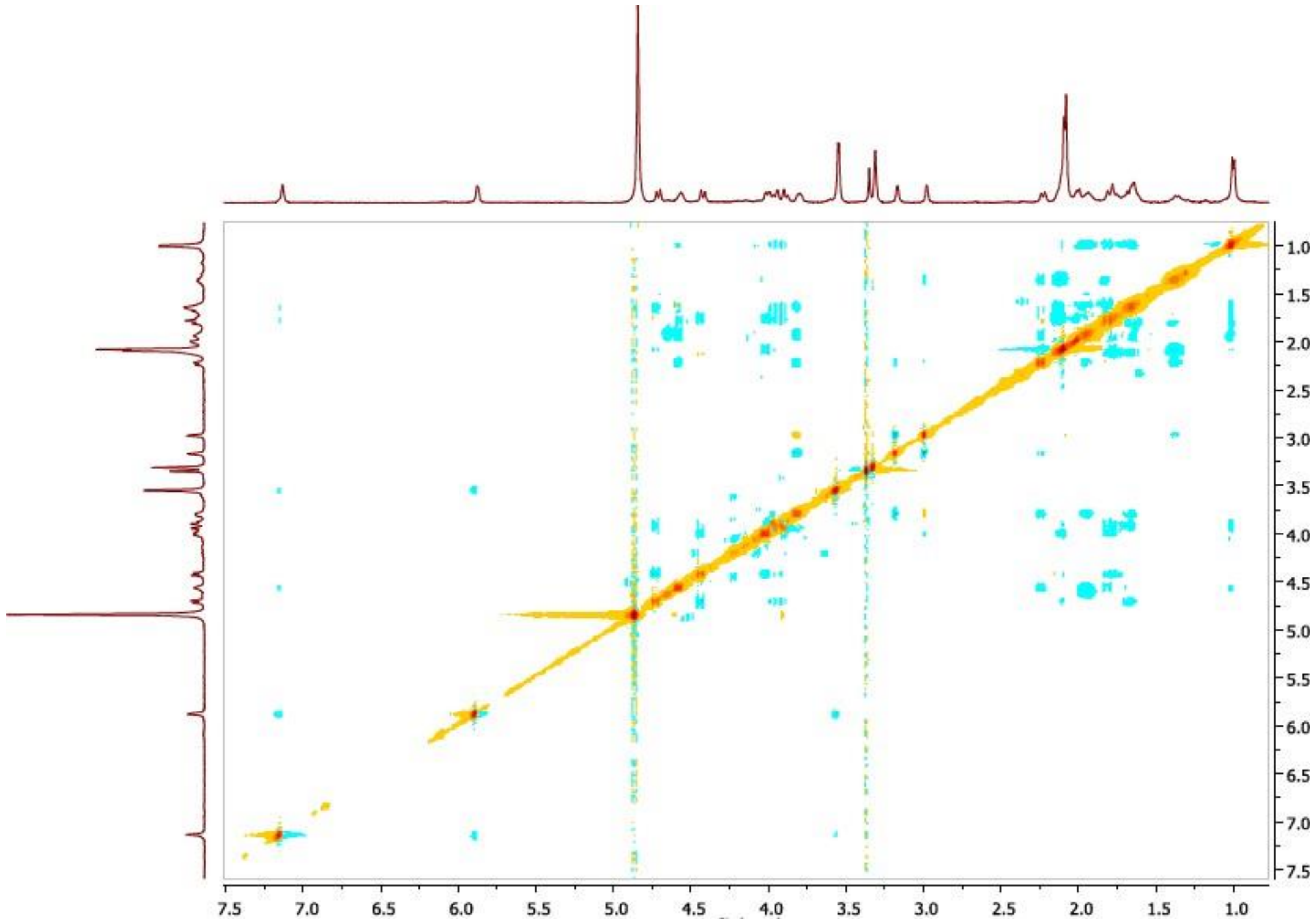

Figure S76. NOESY NMR spectrum of **9** in CD_3OD.

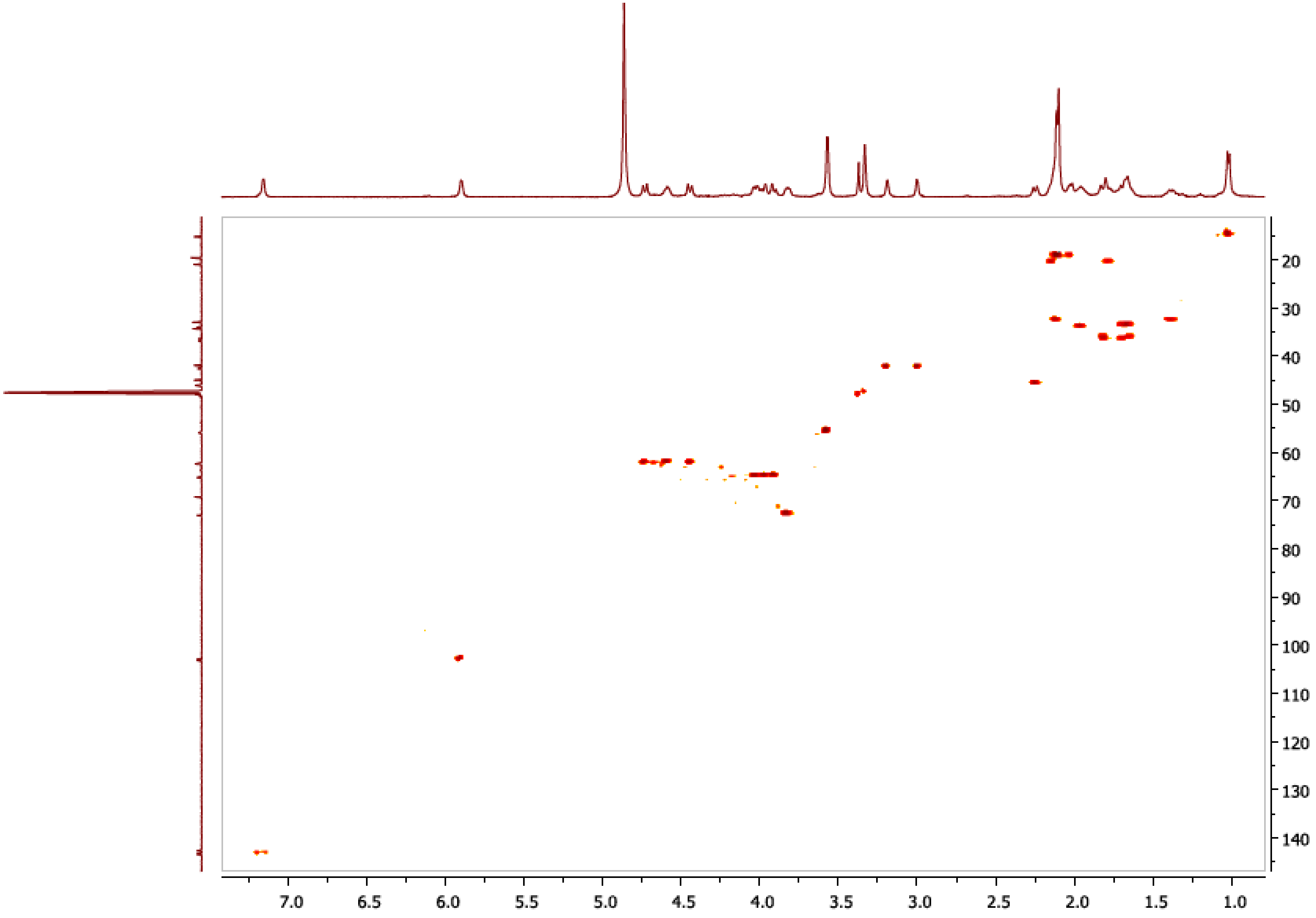

Figure S77. HSQC NMR spectrum of **9** in CD_3OD.

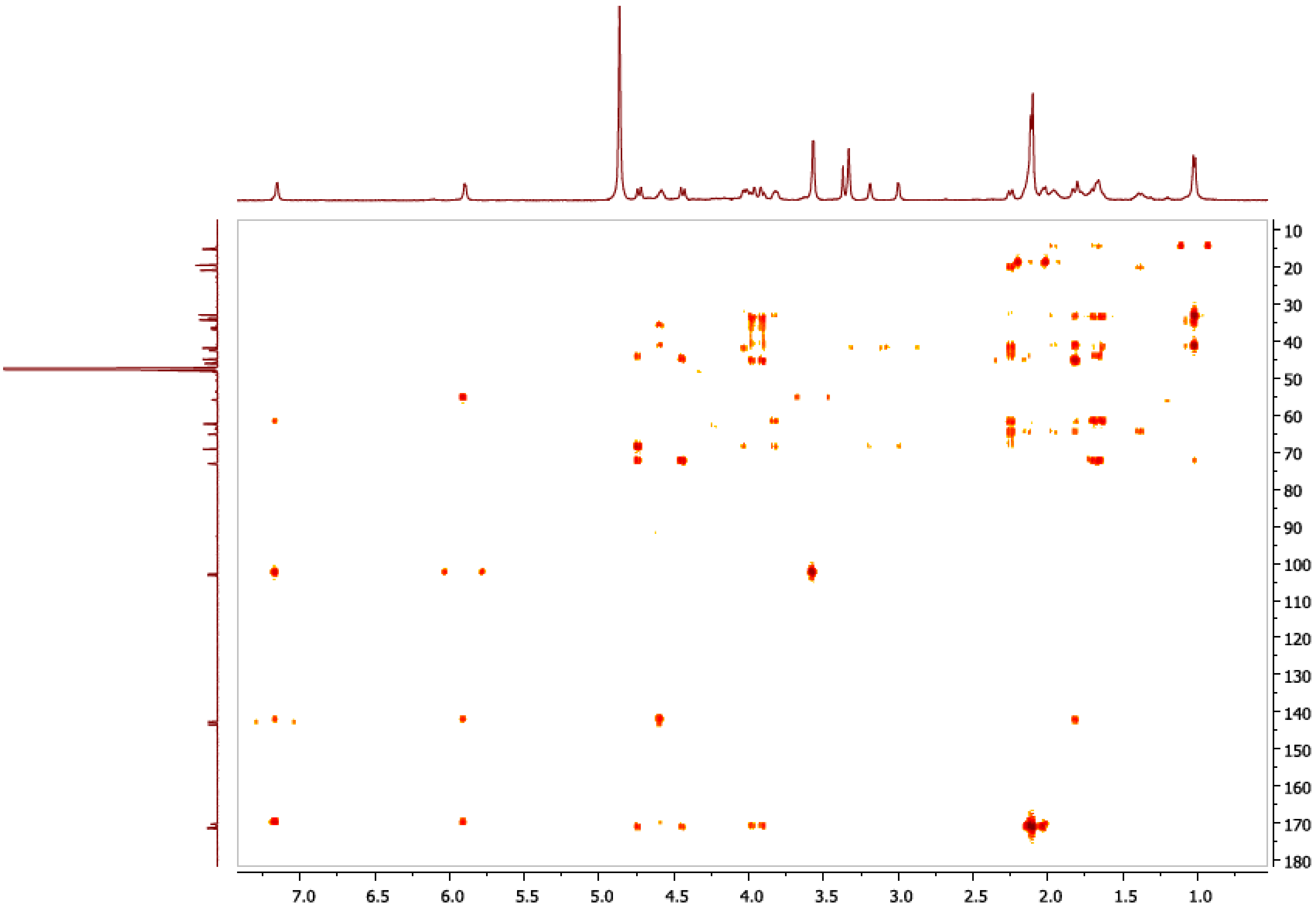

Figure S78. HMBC NMR spectrum of **9** in CD_3OD.

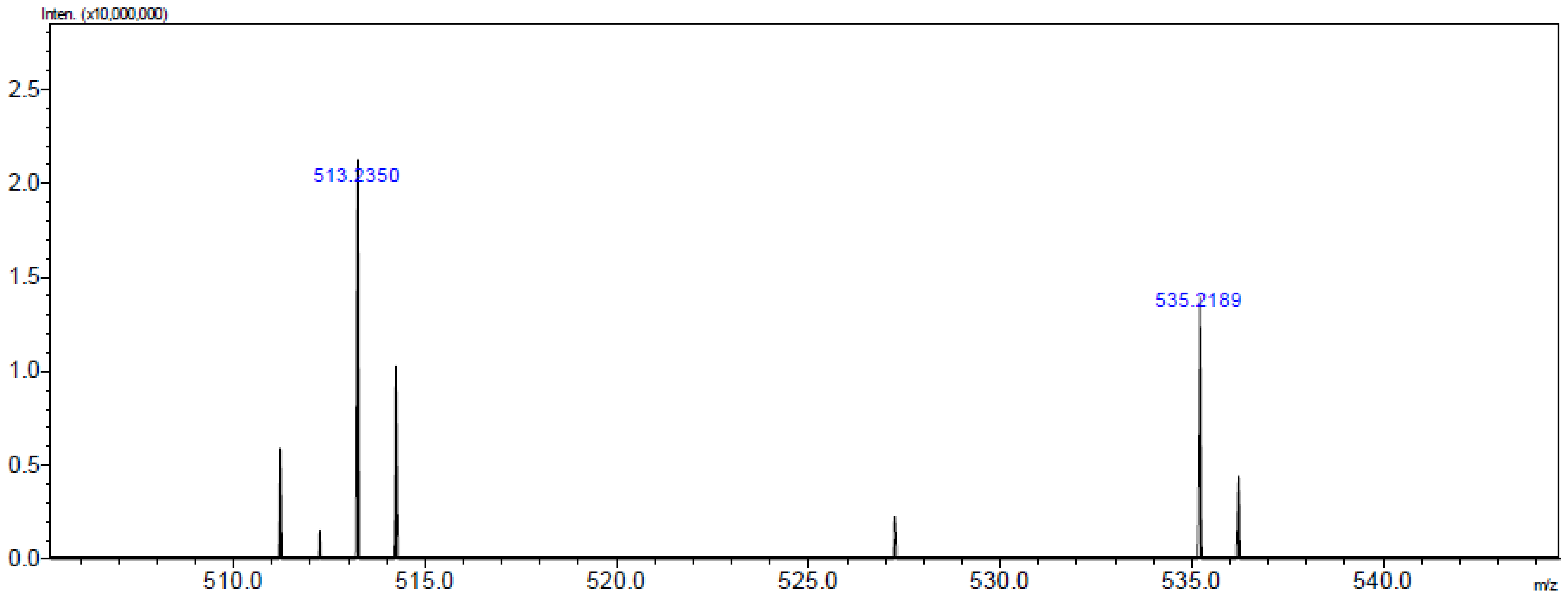

Figure S79. Positive-ion HRESIMS of **9**.

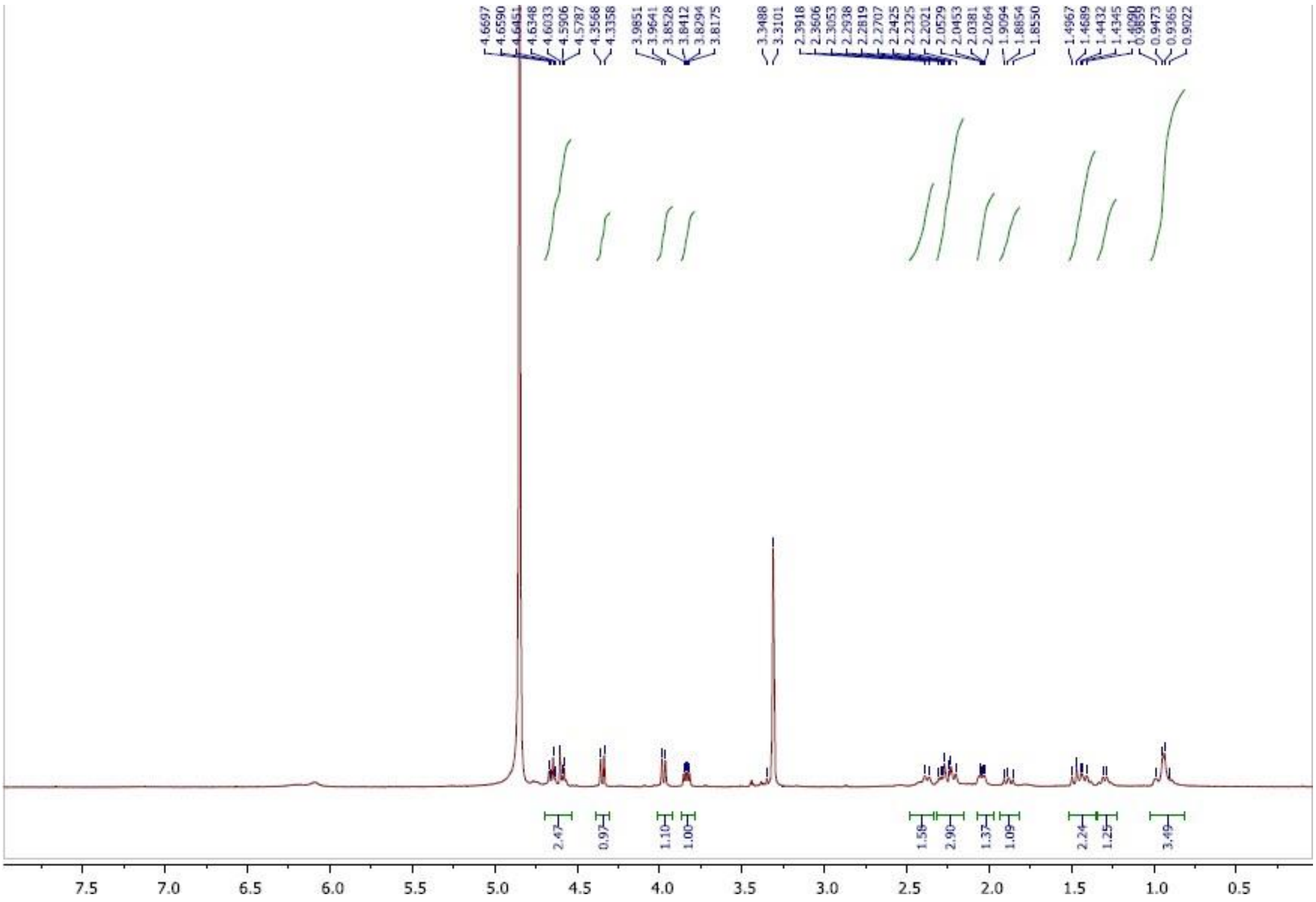

Figure S80. ^{1}H NMR spectrum of **10** in CD_3OD.

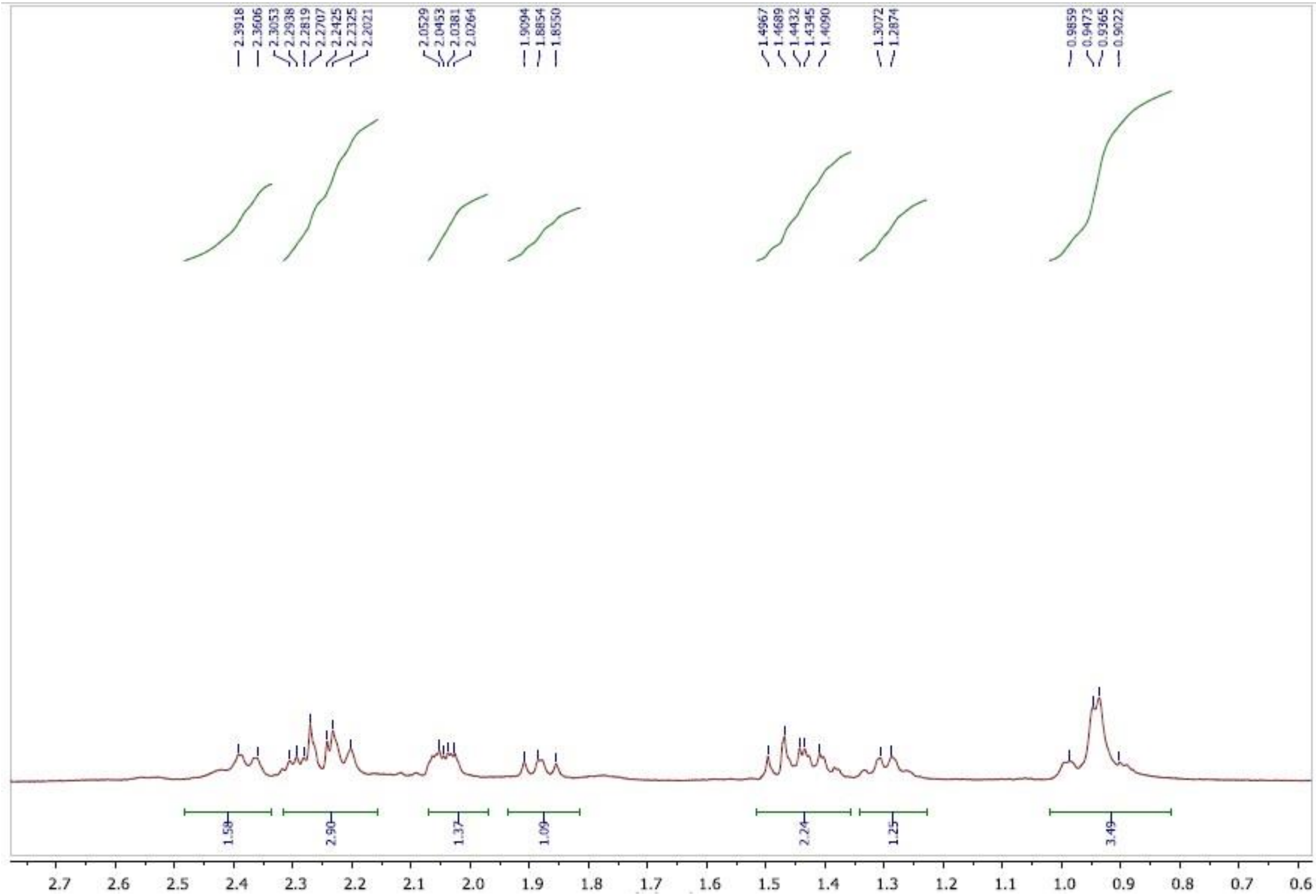

Figure S81. Expansion of part of ^1H NMR spectrum (δ 0.6 – δ 2.7) of **10** in CD_3OD.

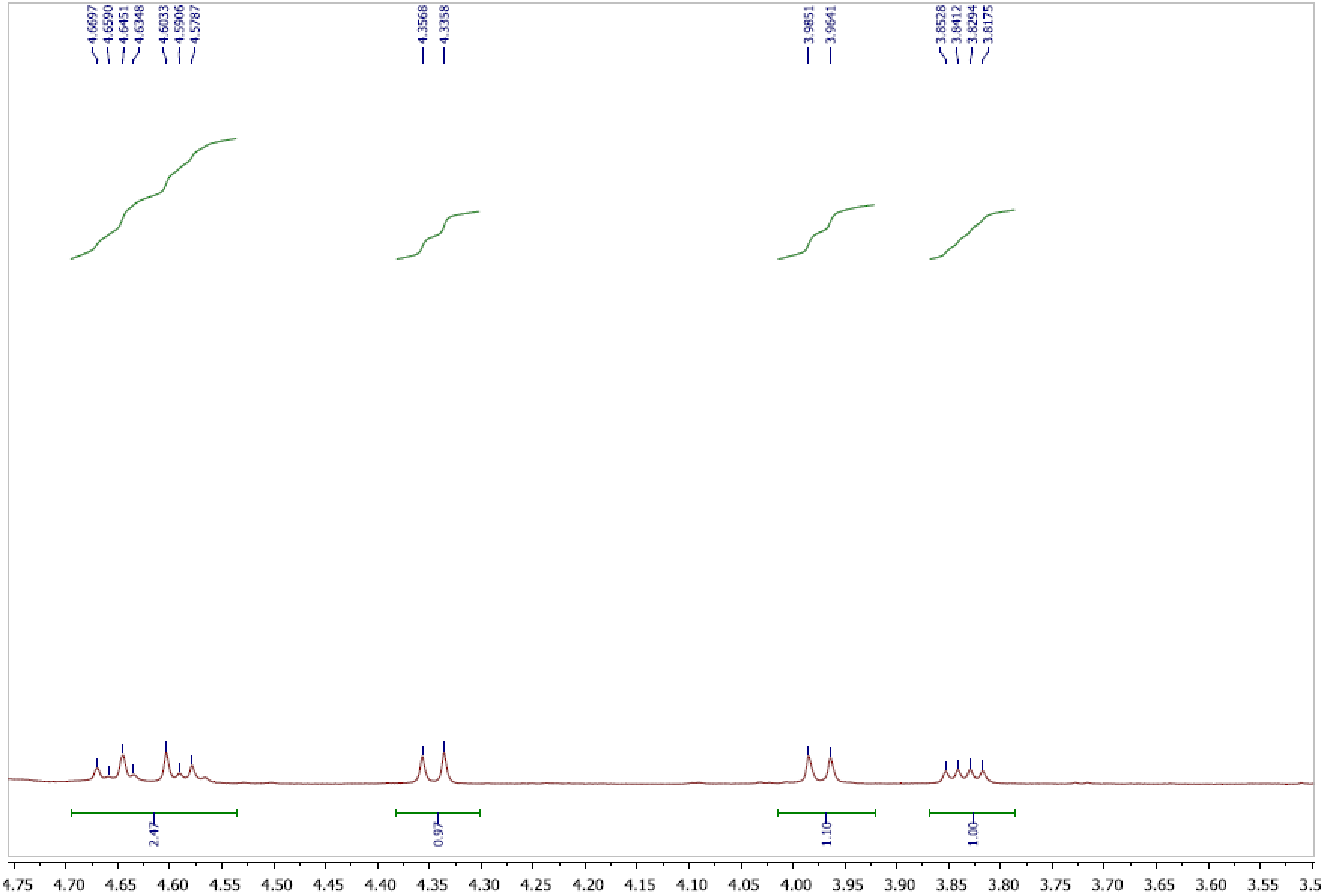

Figure S82. Expansion of part of ^{1}H NMR spectrum (δ 3.5 – δ 4.7) of **10** in CD_3OD.

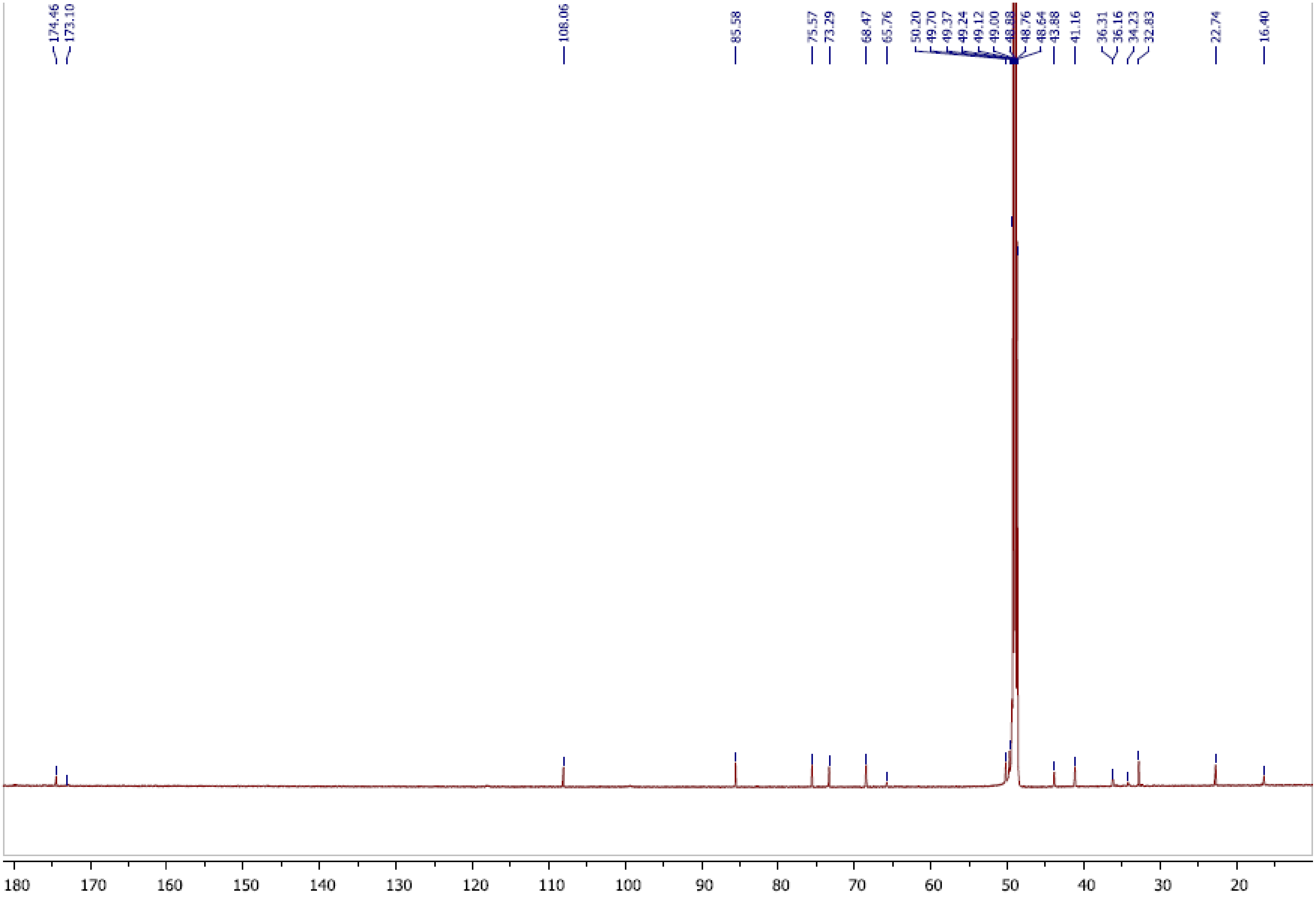

Figure S83. ^{13}C NMR spectrum of **10** in CD_3OD.

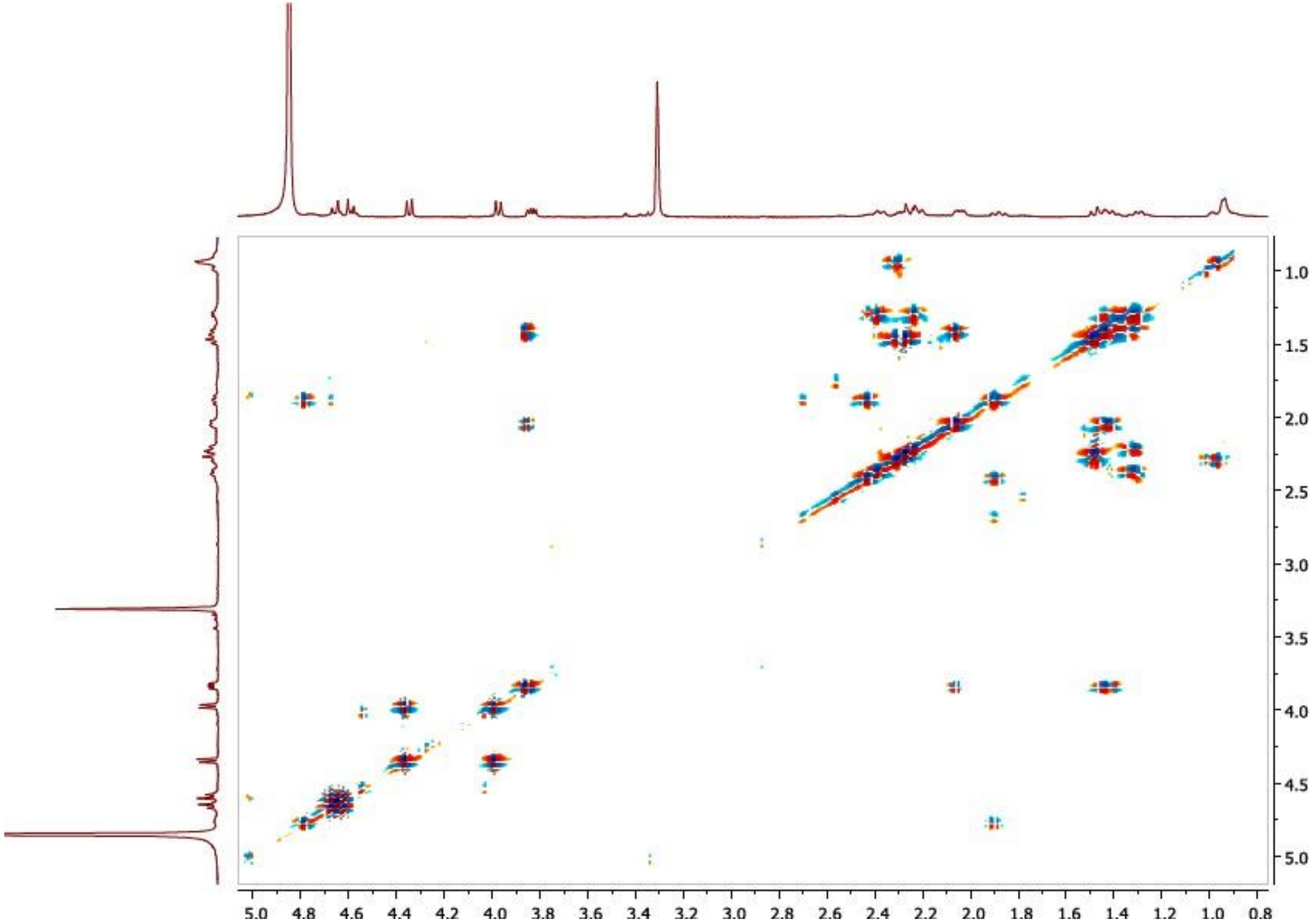

Figure S84. COSY NMR spectrum of **10** in CD_3OD.

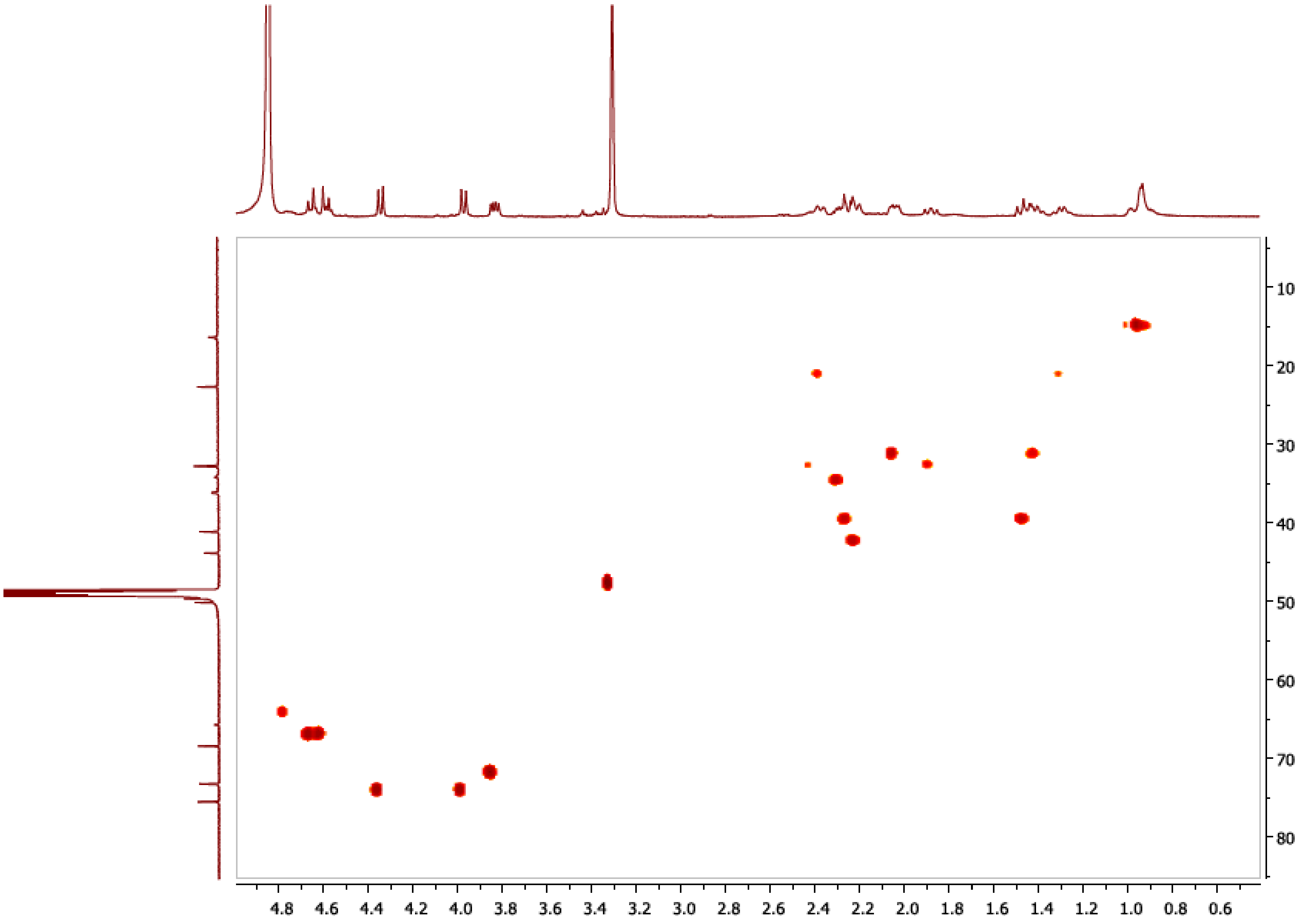

Figure S85. HSQC NMR spectrum of **10** in CD_3OD.

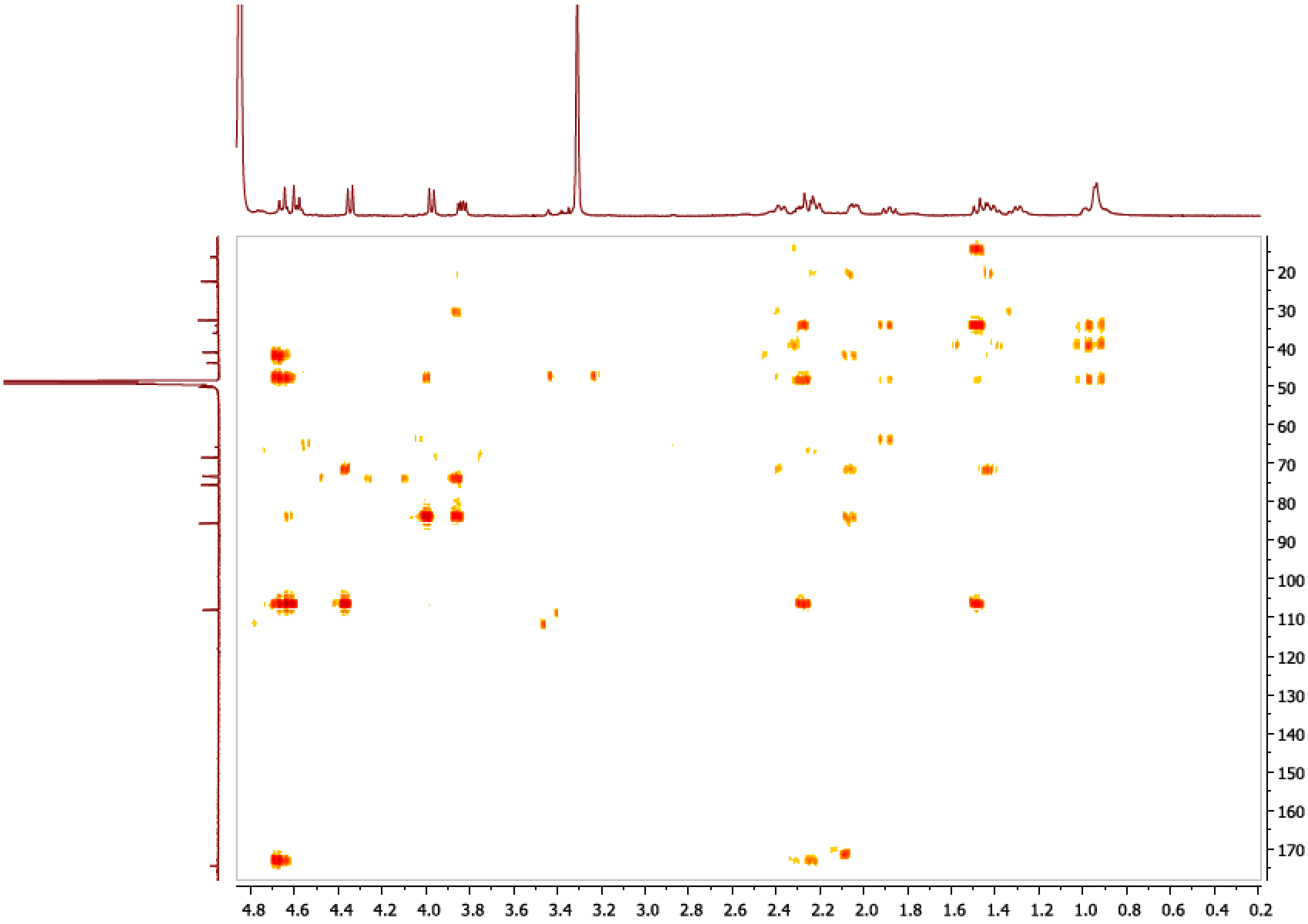

Figure S86. HMBC NMR spectrum of **10** in CD_3OD.

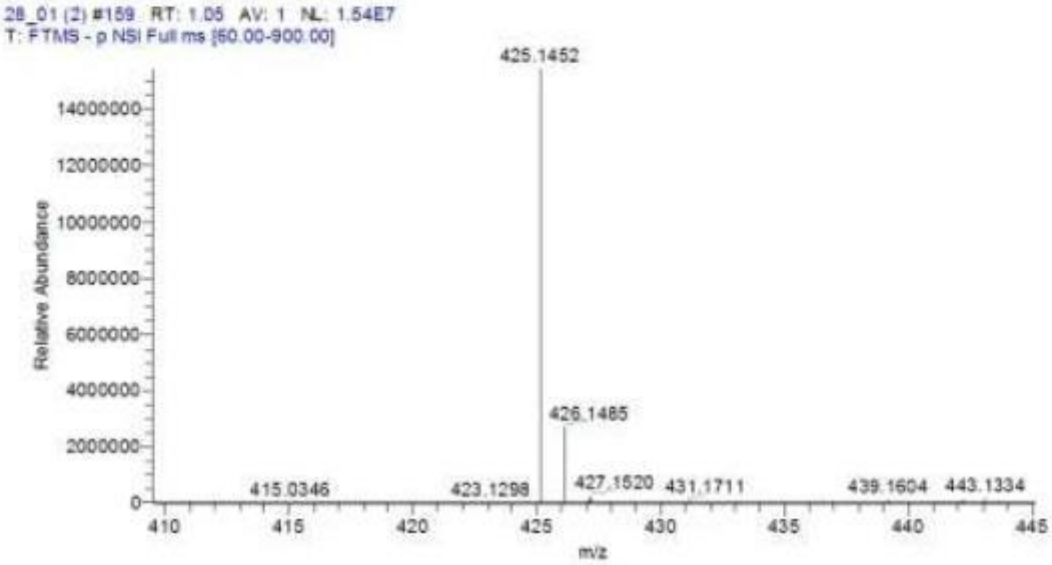

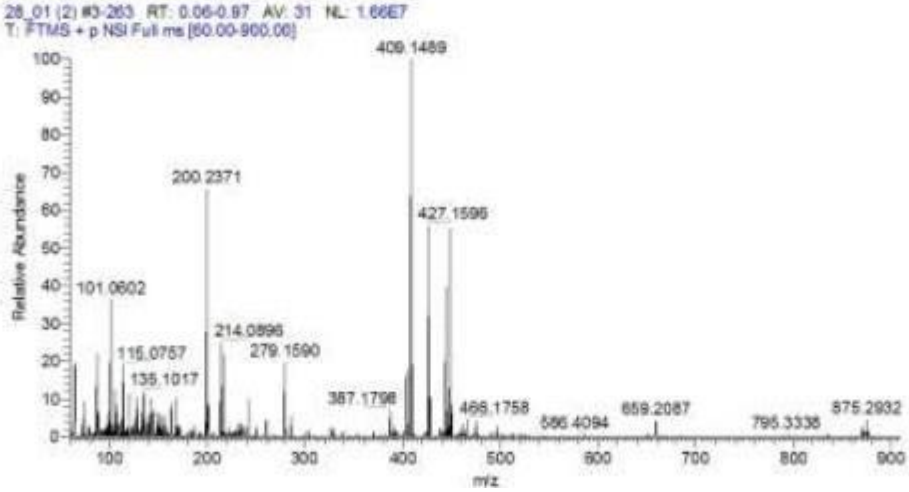

Figure S87. Positive-ion and negative-ion HRESIMS of **10**.

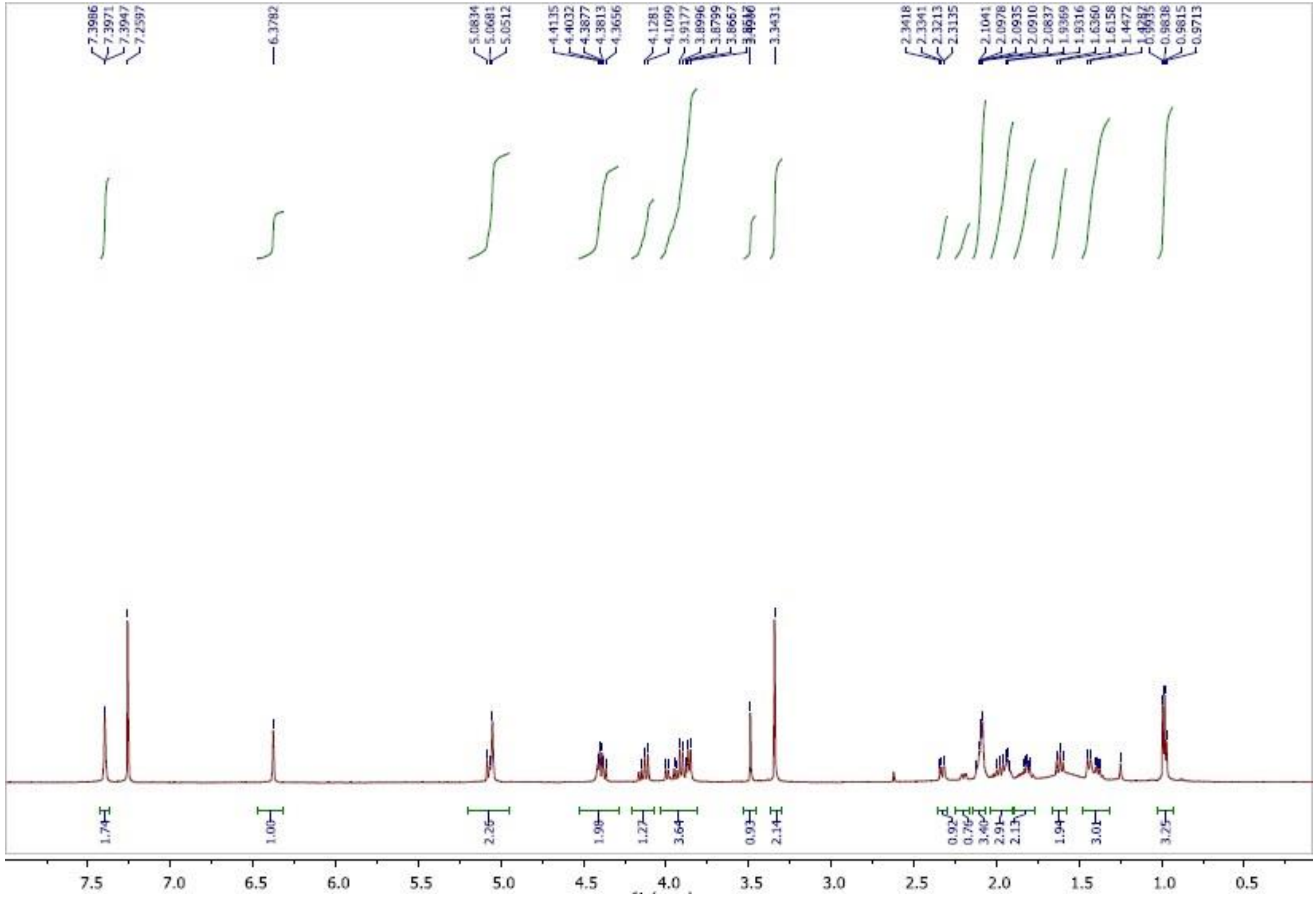

Figure S88. ^{1}H NMR spectrum of **11** in CD_3OD.

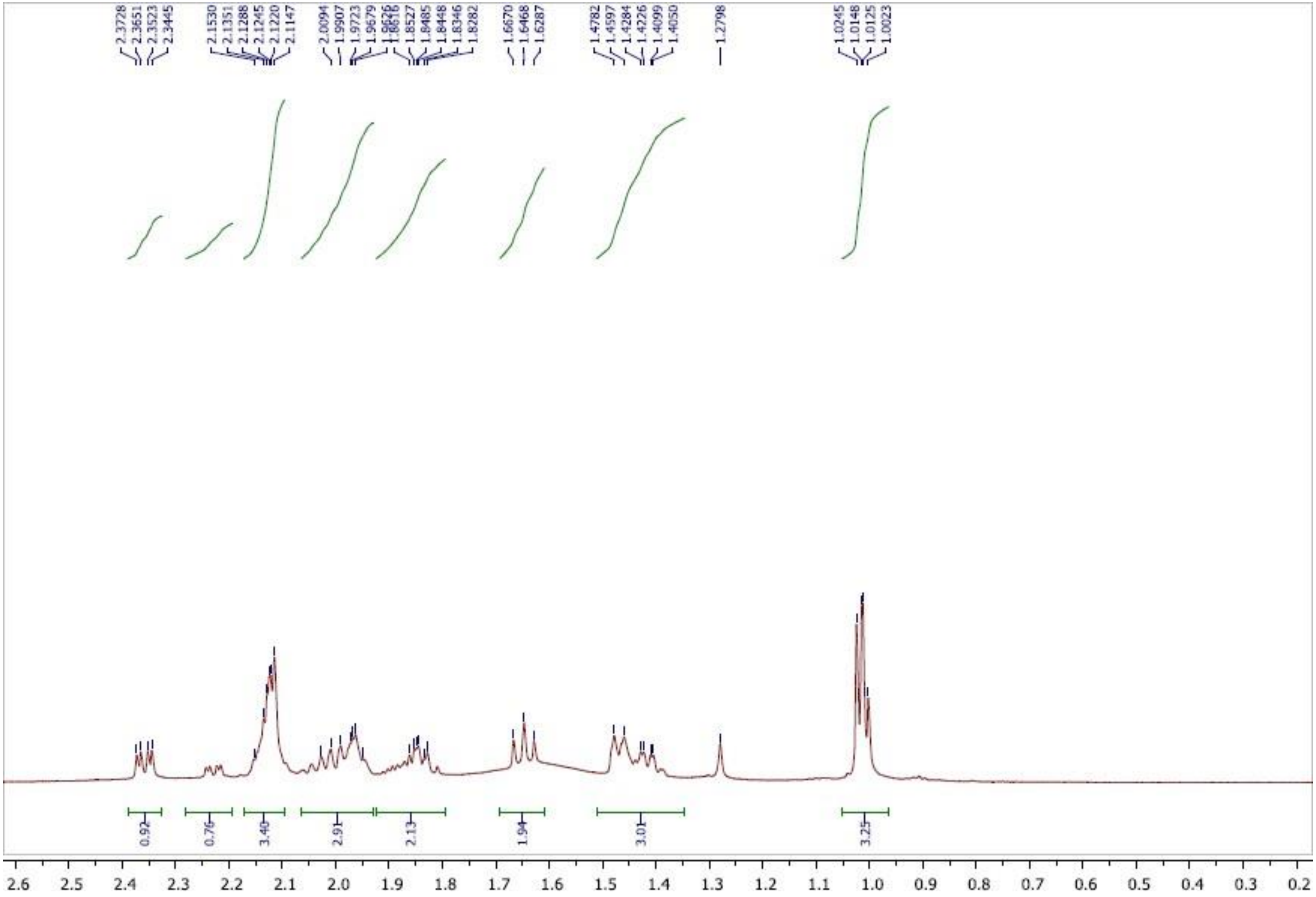

Figure S89. Expansion of part of ¹H NMR spectrum (δ 0.2 – δ 2.6) of **11** in CD_3OD.

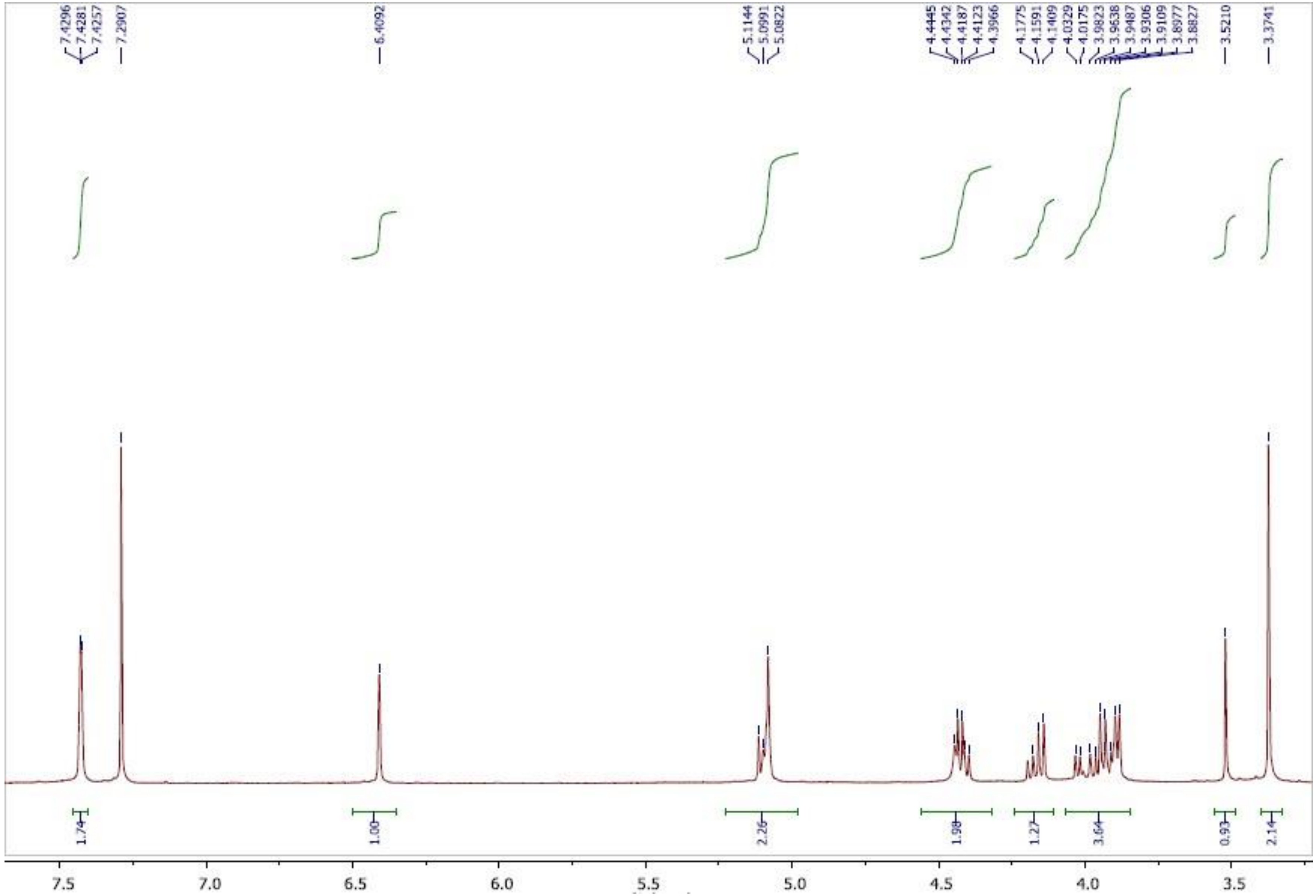

Figure S90. Expansion of part of ^{1}H NMR spectrum (δ 3.4 – δ 7.5) of **11** in CD_3OD.

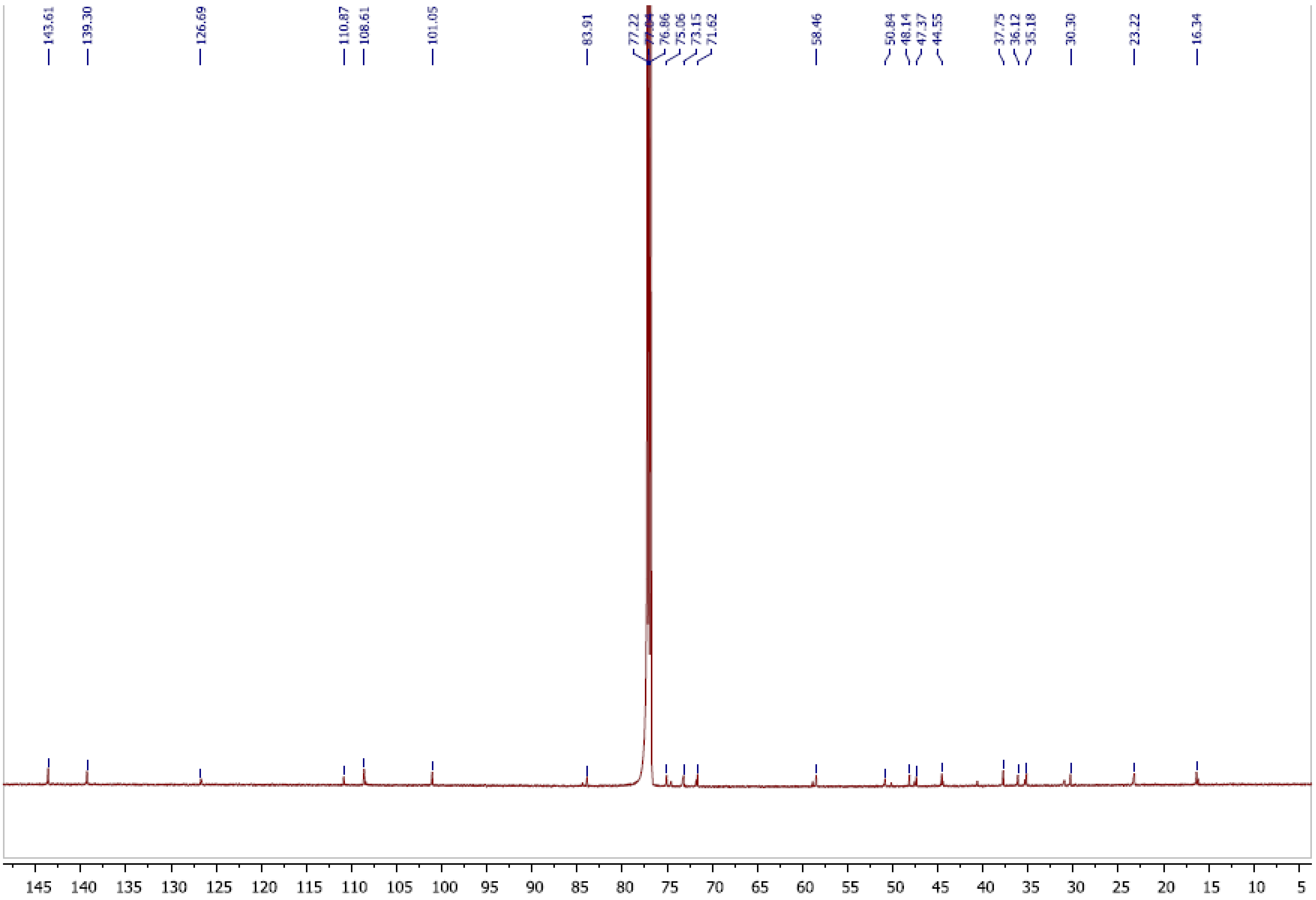

Figure S91. ^{13}C NMR spectrum of **11** in CD_3OD.

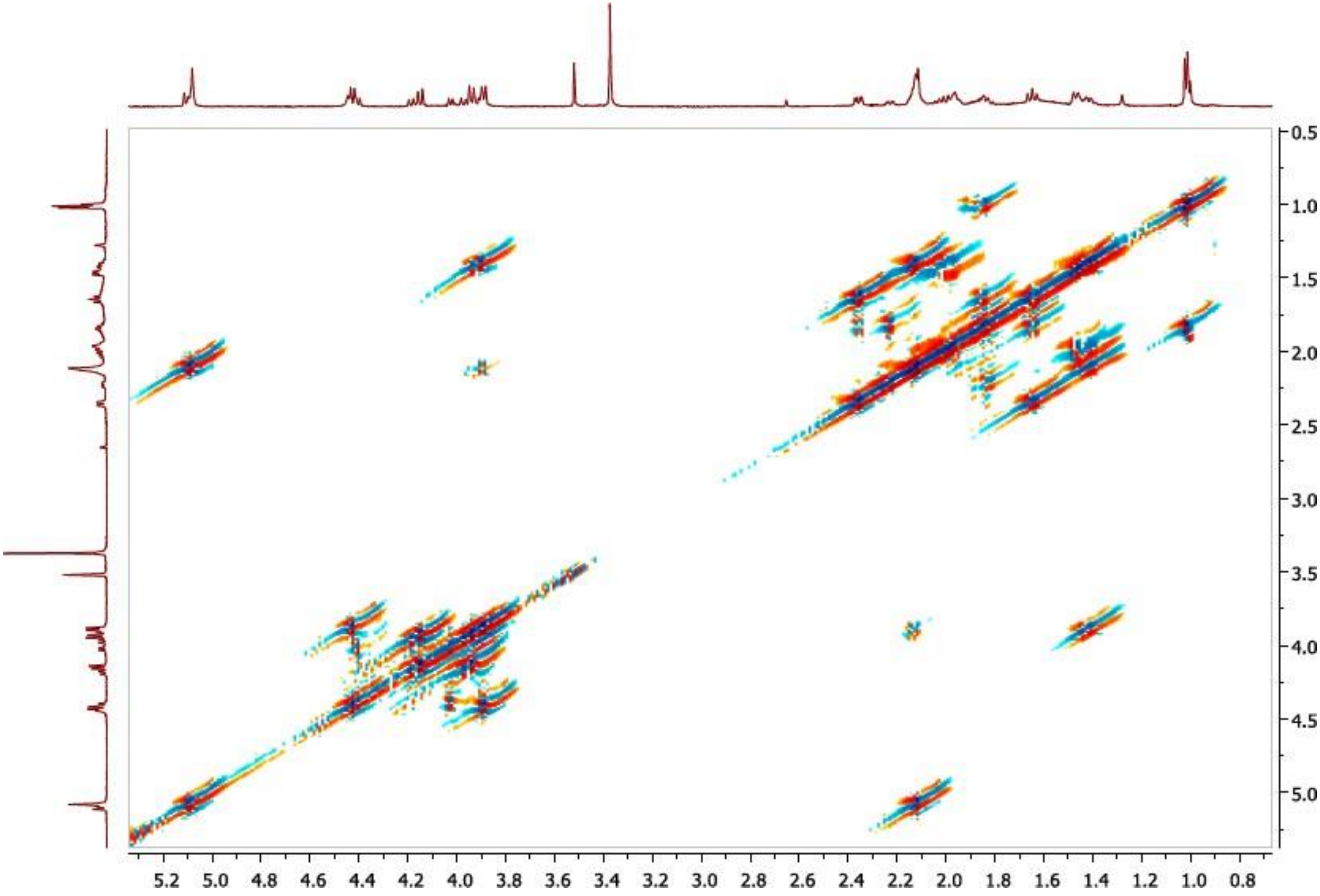

Figure S92. COSY NMR spectrum of **11** in CD_3OD.

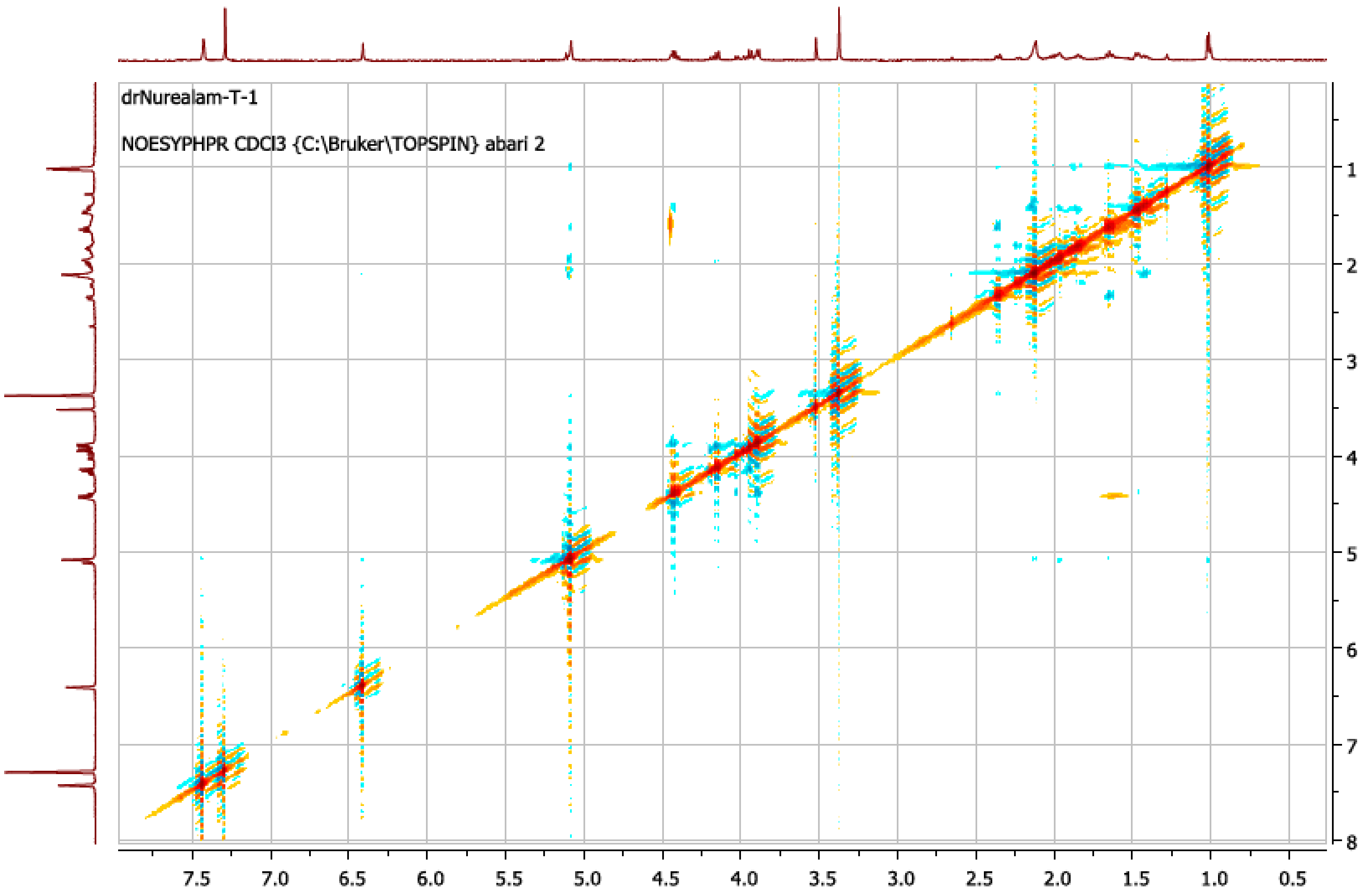

Figure S93. NOESY NMR spectrum of **11** in CD_3OD.

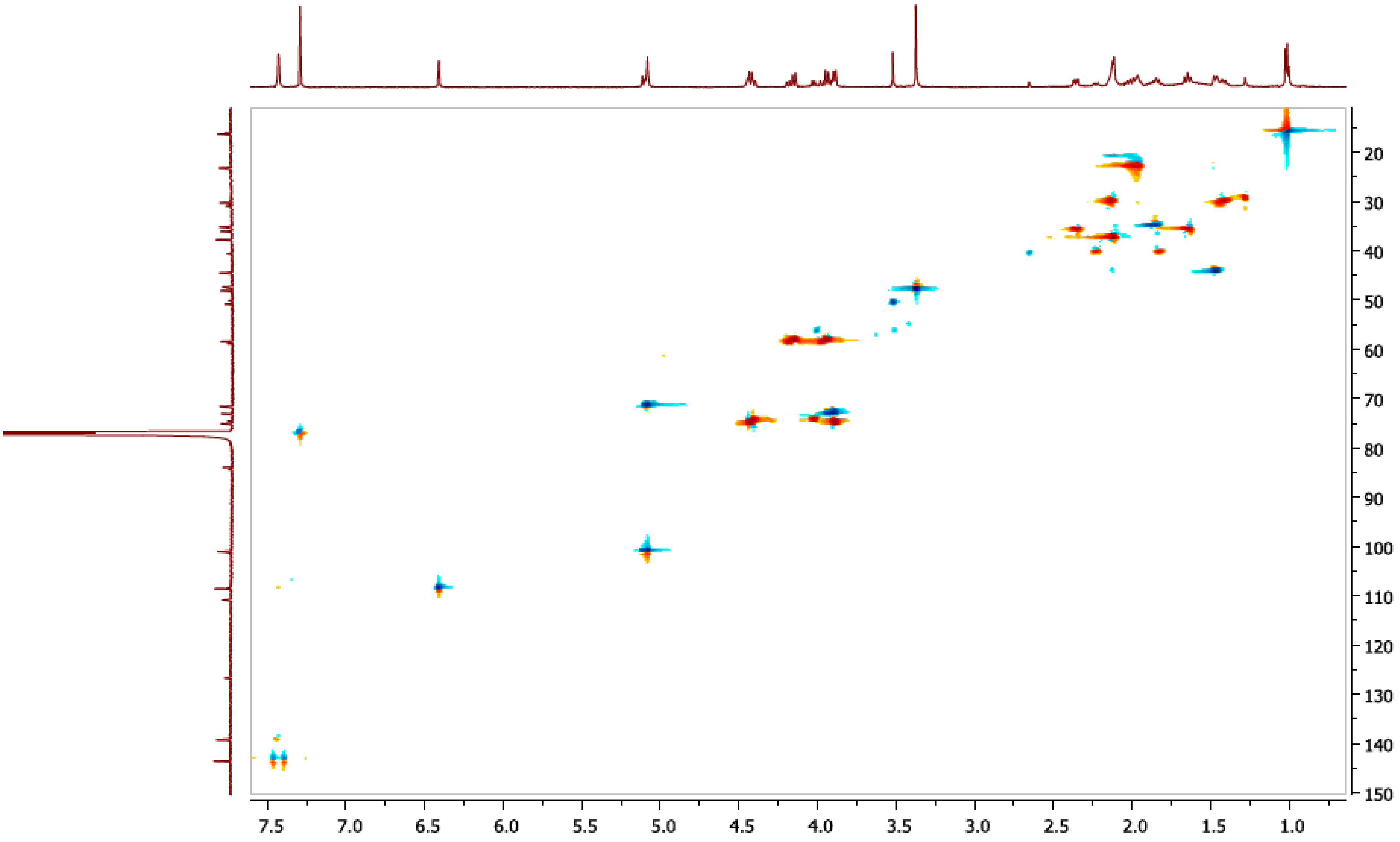

Figure S94. HSQC NMR spectrum of **11** in CD_3OD.

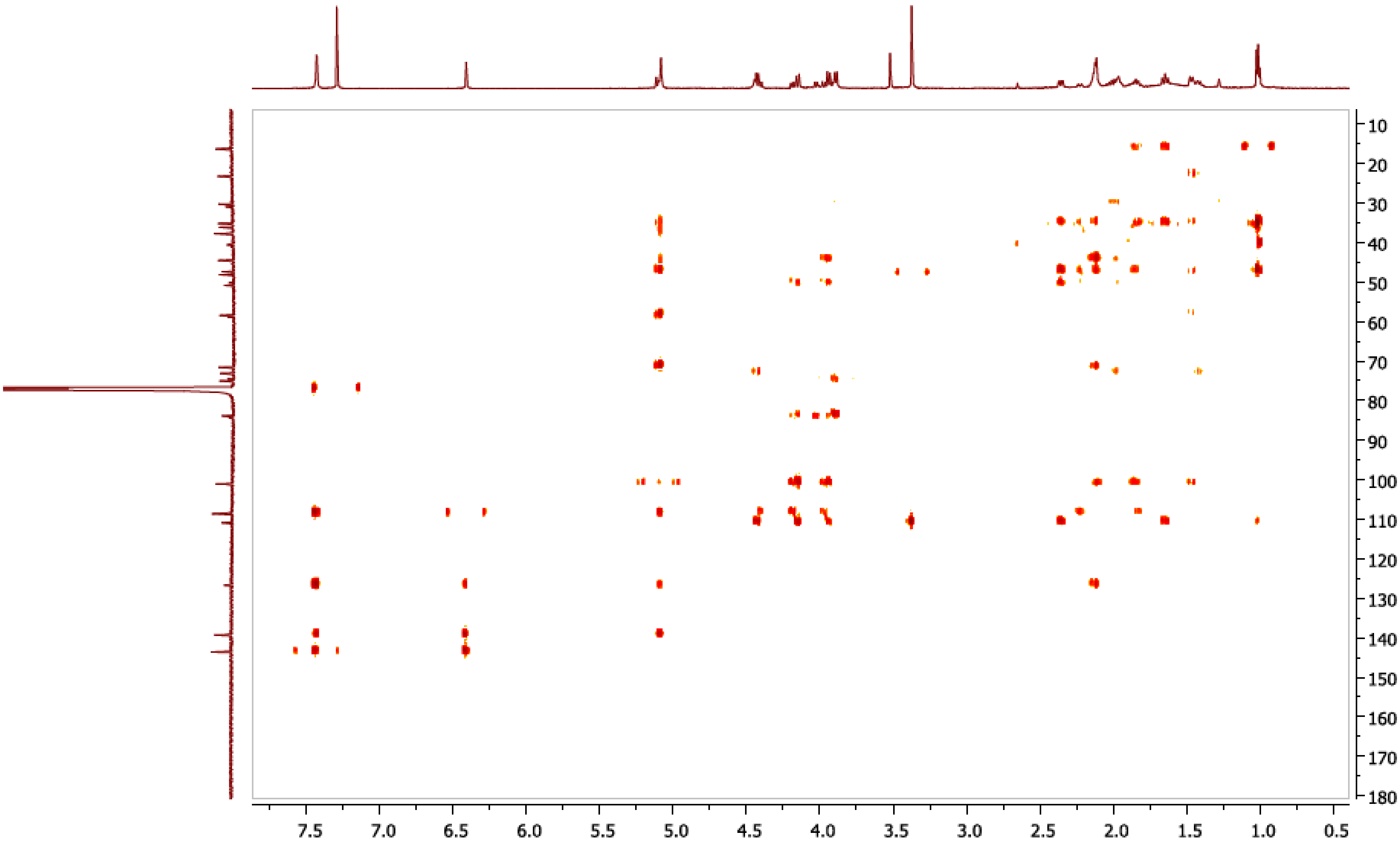

Figure S95. HMBC NMR spectrum of **11** in CD_3OD.

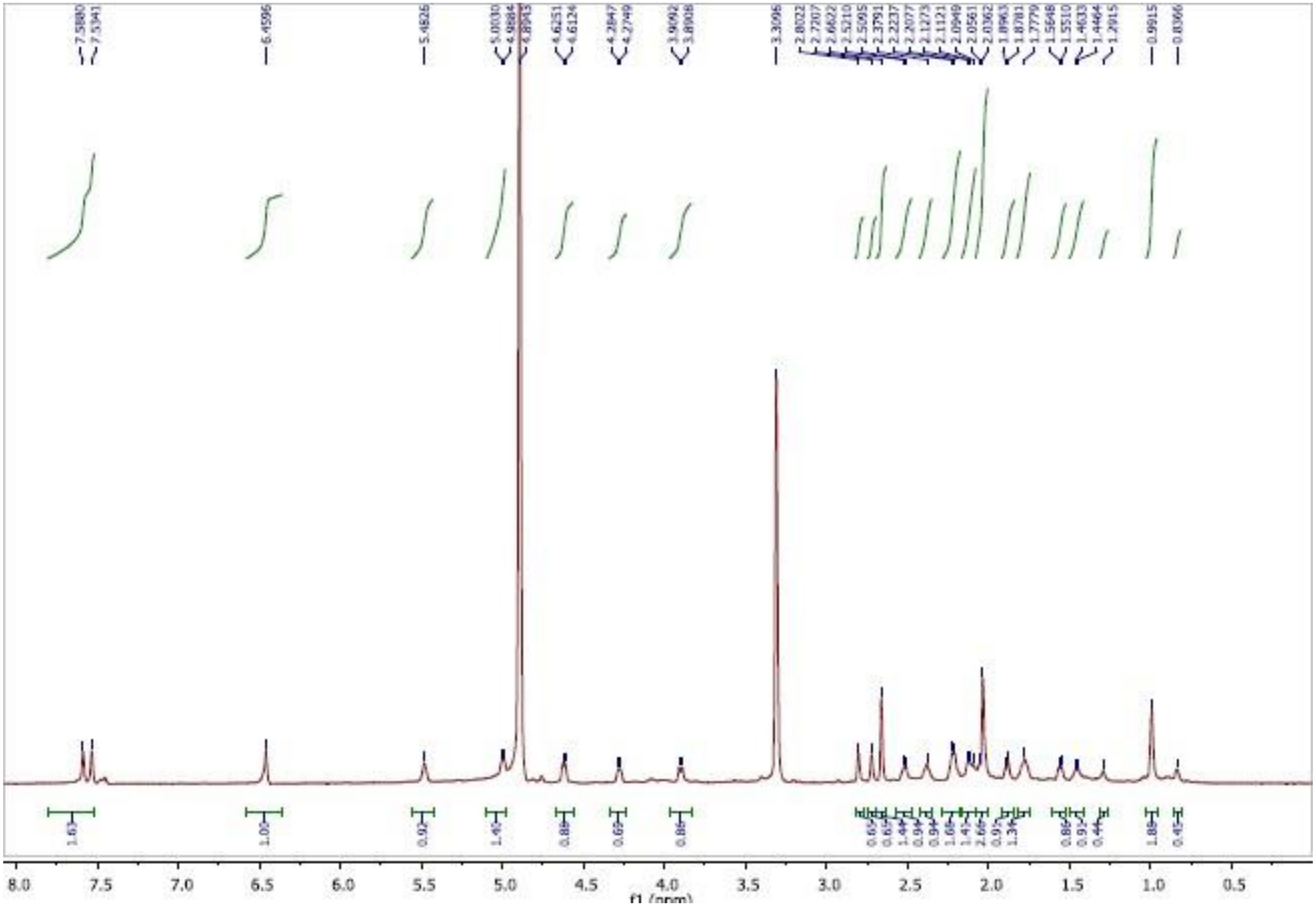

Figure S96. ^{1}H NMR spectrum of **12** in CD_3OD.

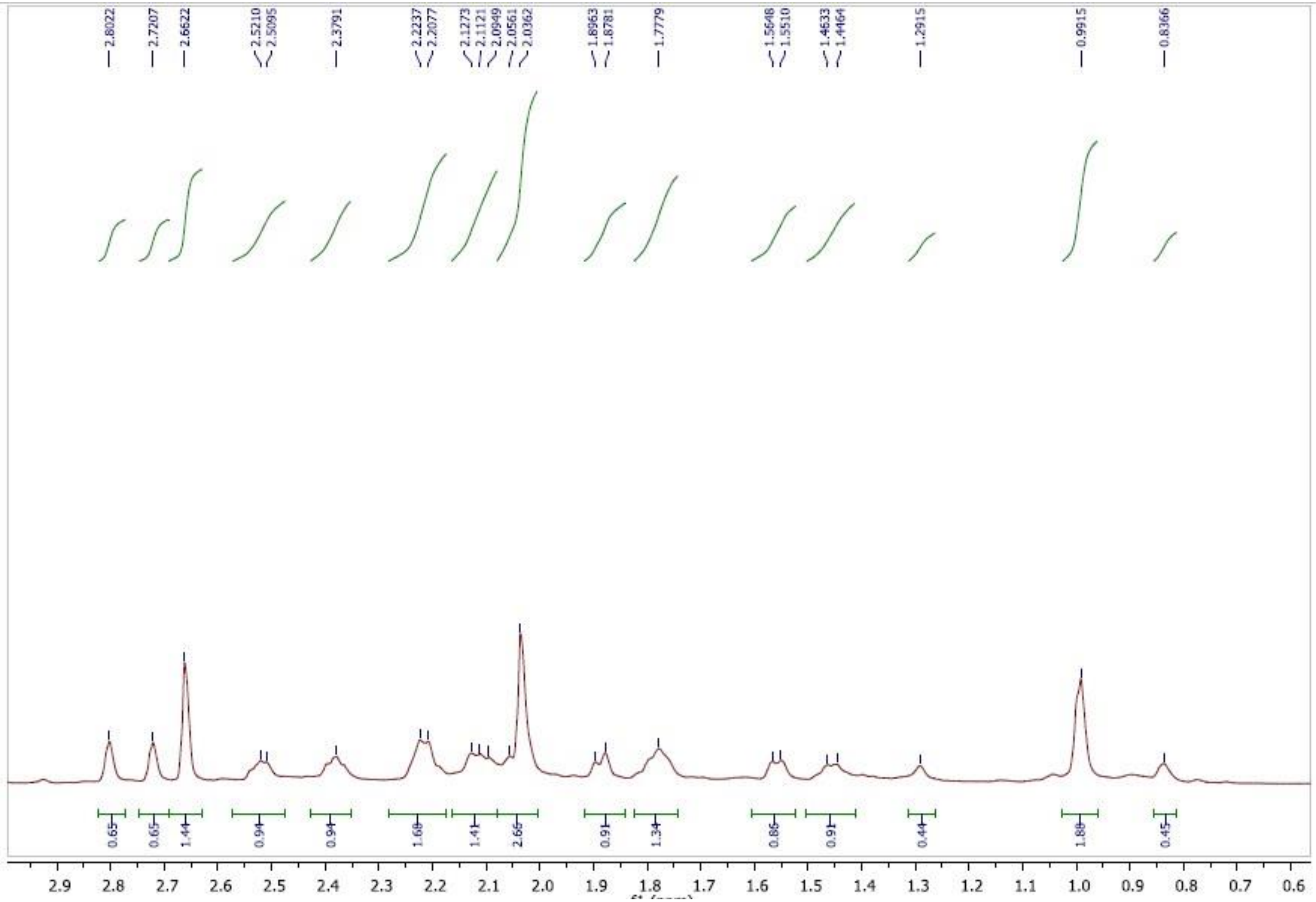

Figure S97. Expansion of part of ^{1}H NMR spectrum (δ 0.6 – δ 2.9) of **12** in CD_3OD.

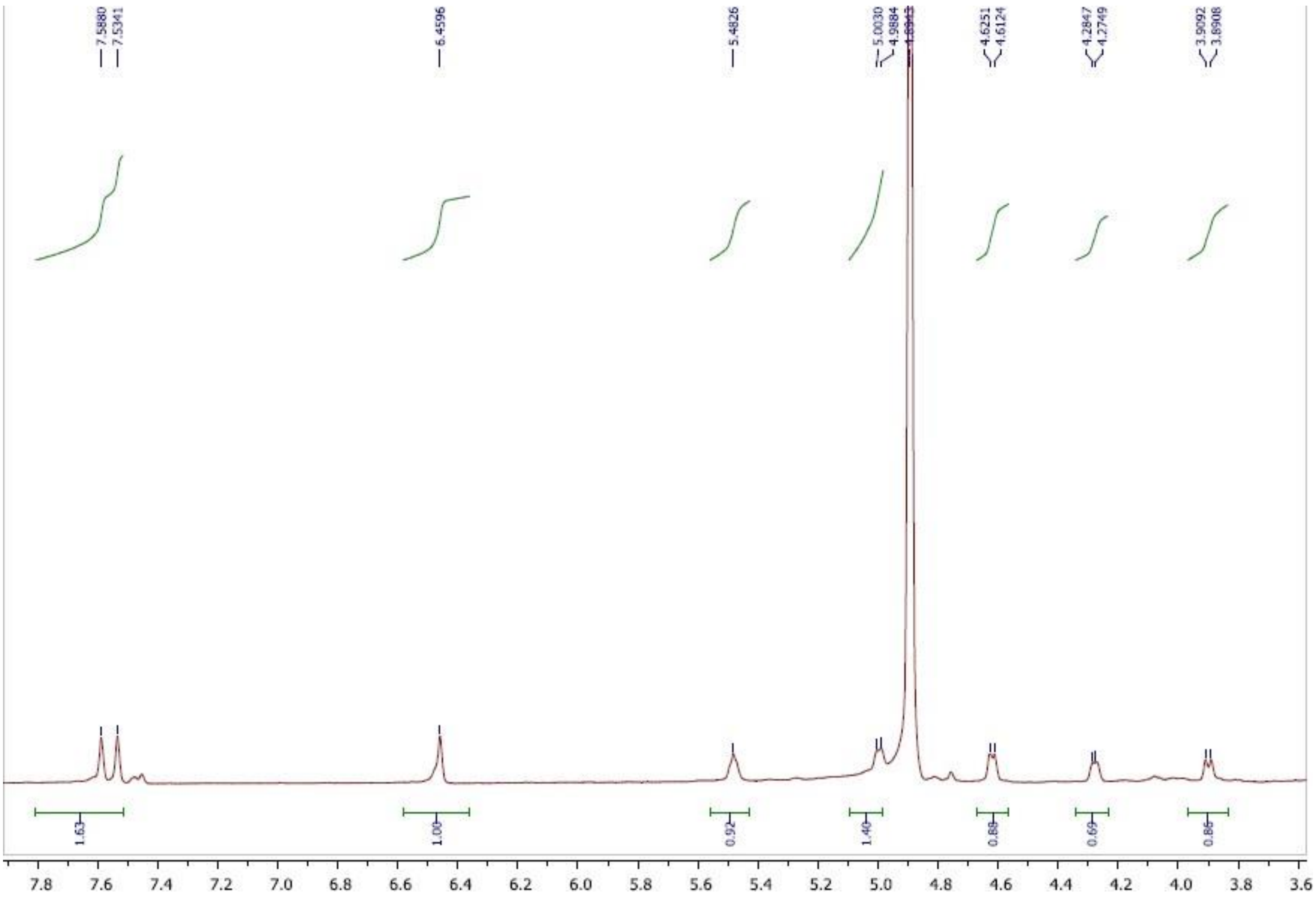

Figure S98. Expansion of part of ^{1}H NMR spectrum (δ 3.6 – δ 7.8) of **12** in CD_3OD.

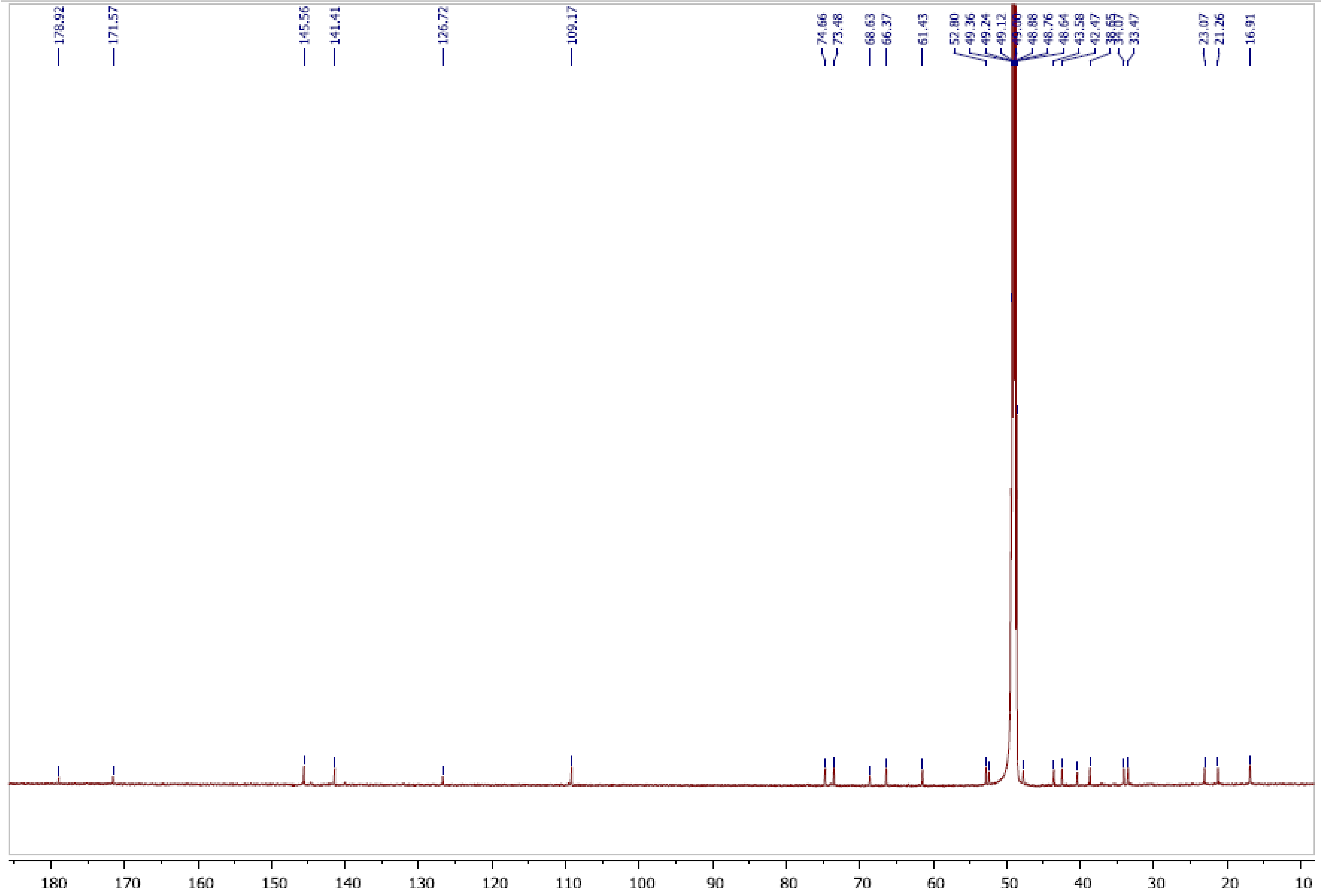

Figure S99. ^{13}C NMR spectrum of **12** in CD_3OD.

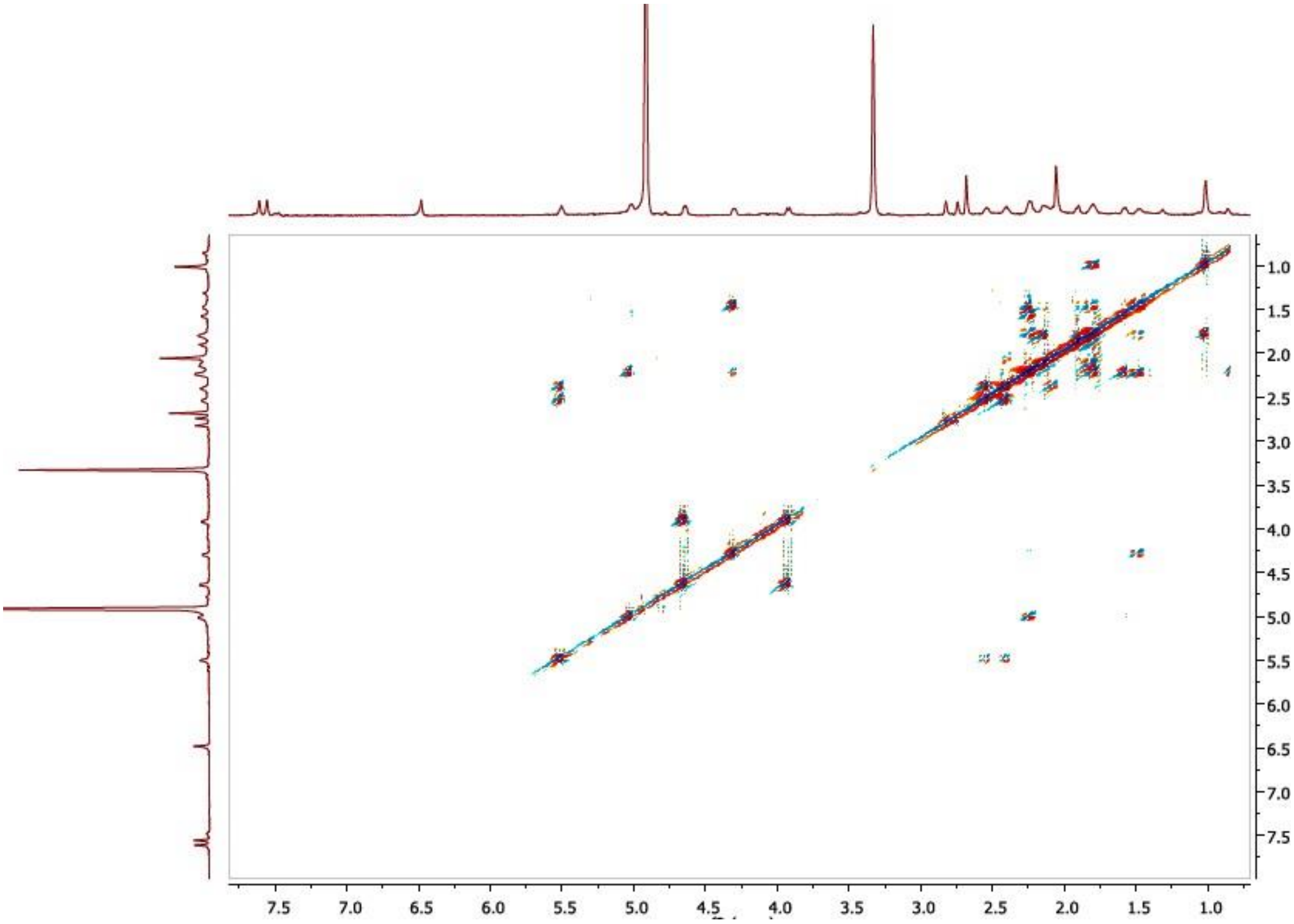

Figure S100. COSY NMR spectrum of **12** in CD_3OD.

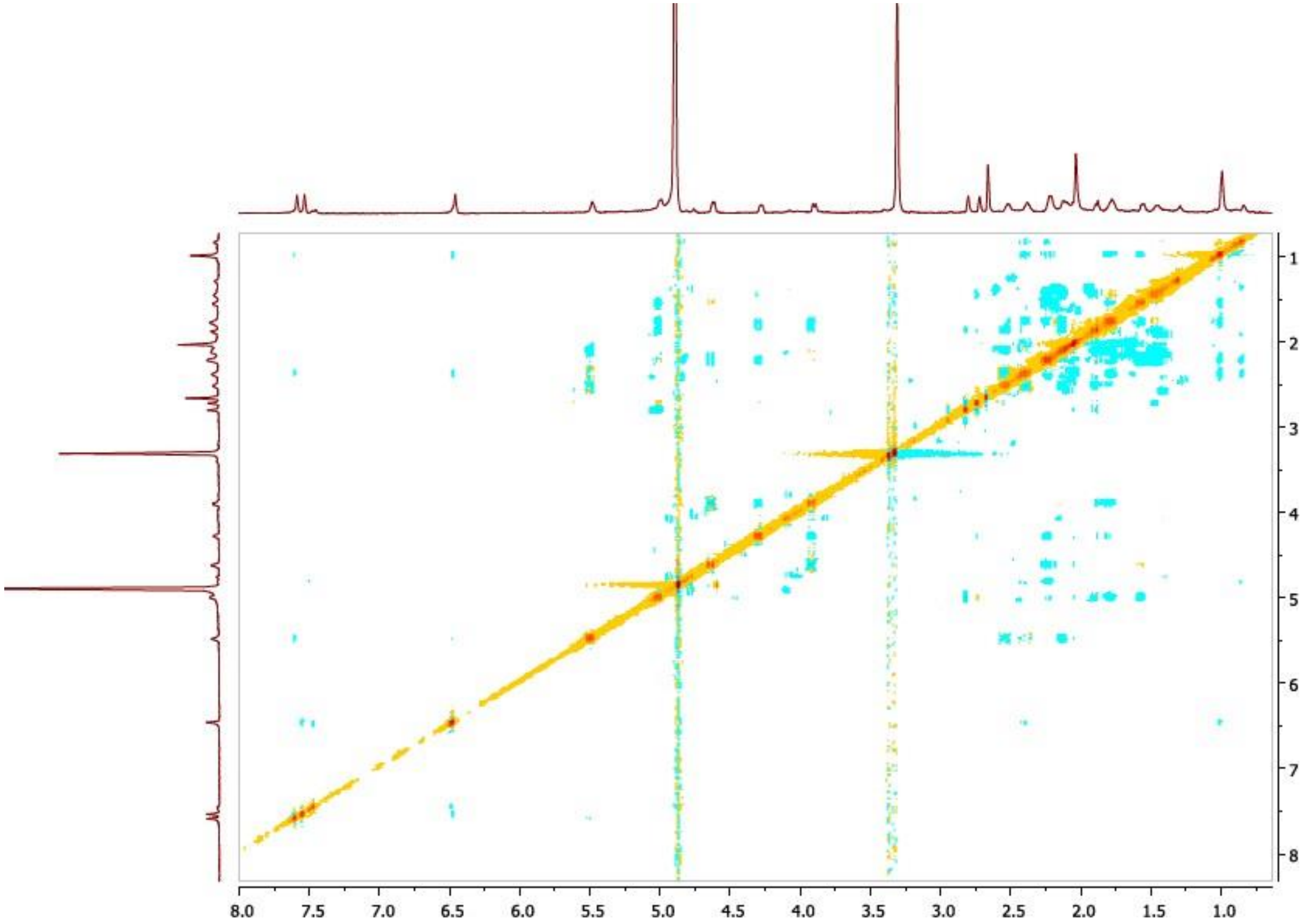

Figure S101. NOESY NMR spectrum of **12** in CD_3OD.

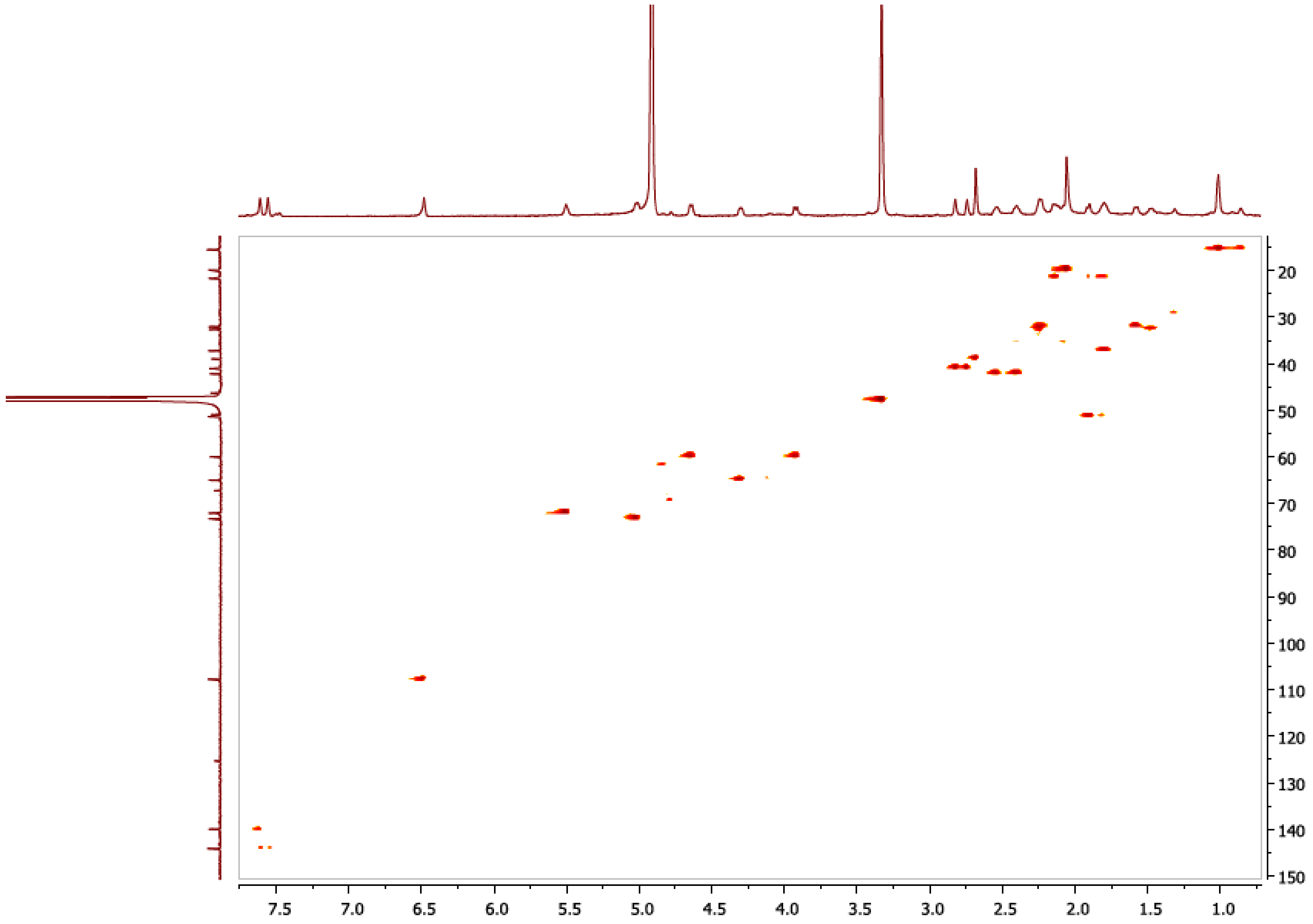

Figure S102. HSQC NMR spectrum of **12** in CD_3OD.

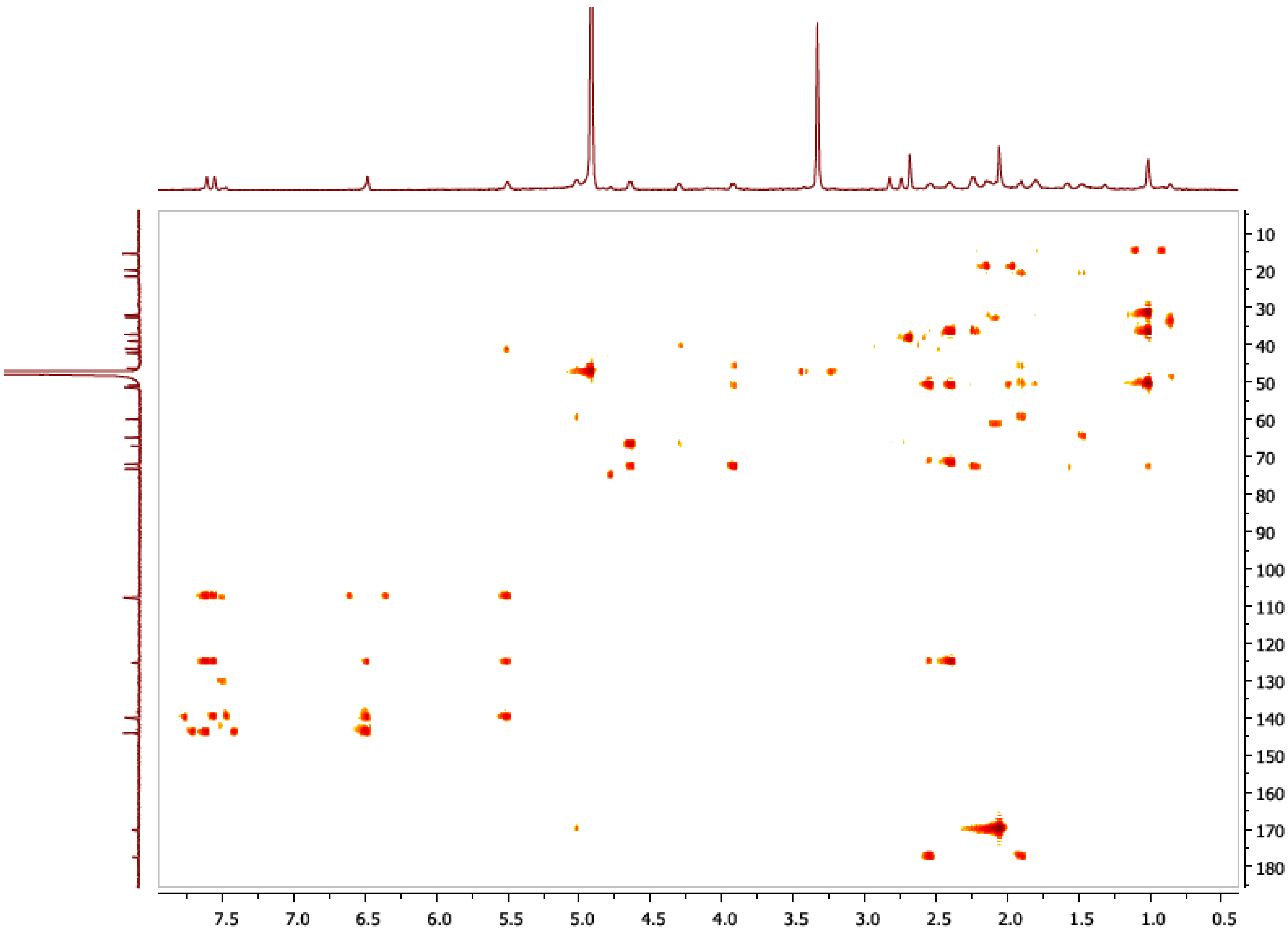

Figure S103. HMBC NMR spectrum of **12** in CD_3OD.

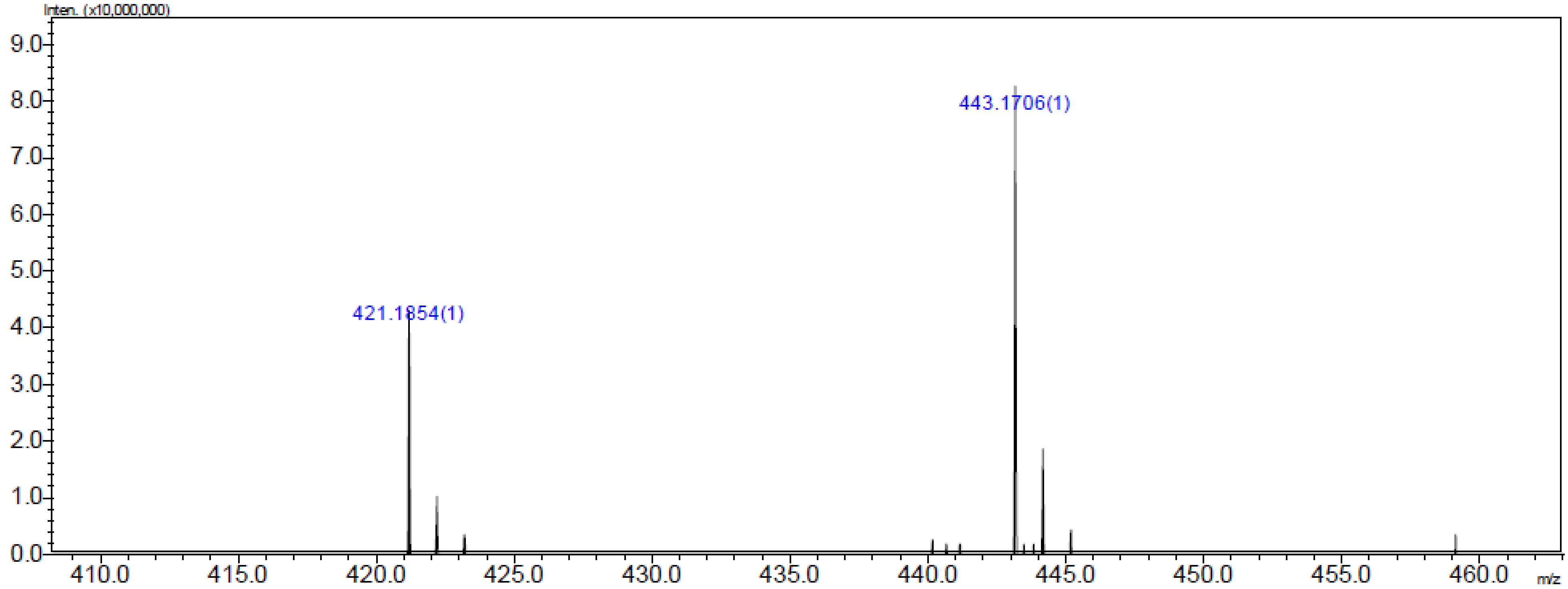

Figure S104. Positive-ion HRESIMS of **12**.

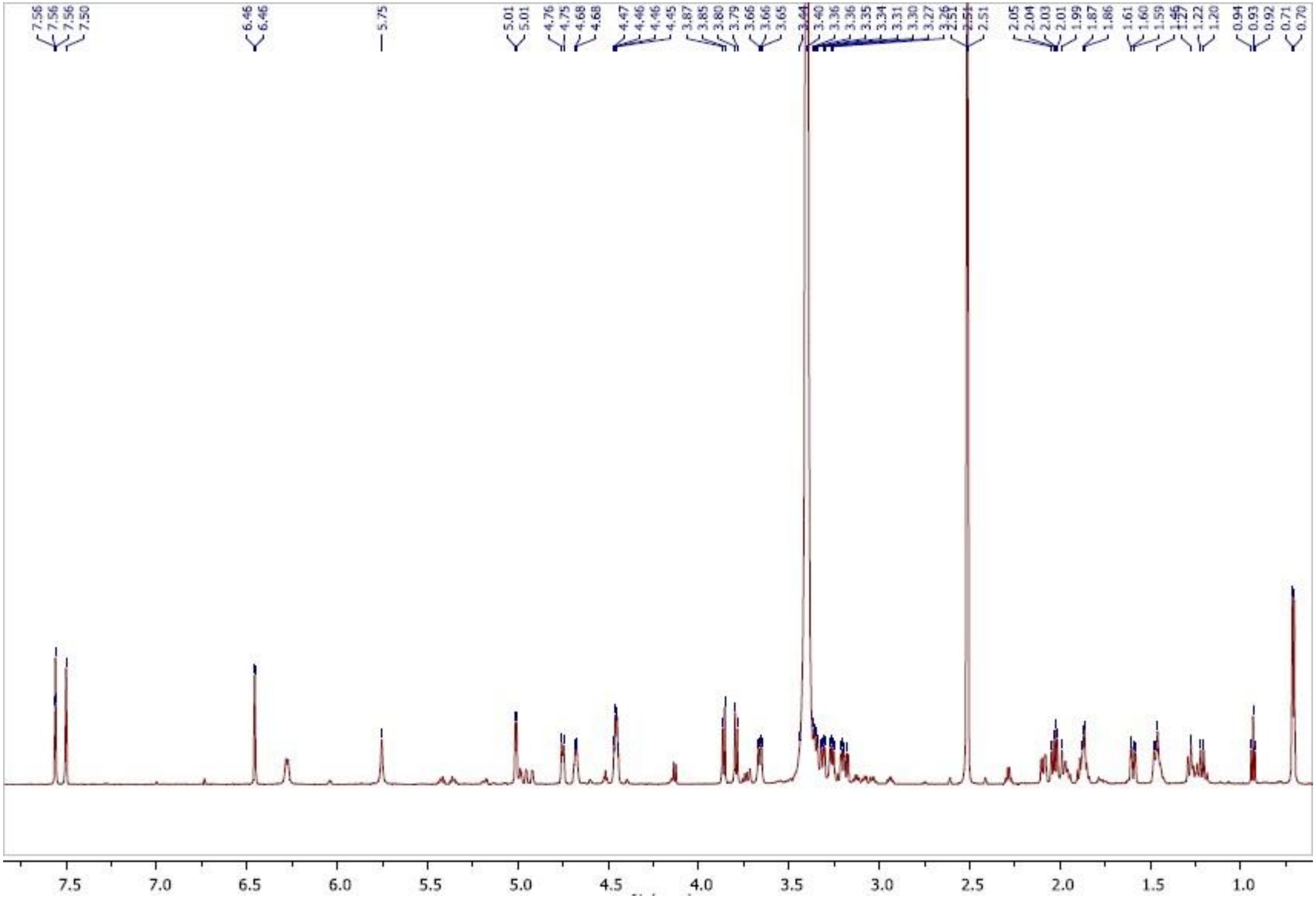

Figure S105. ^1H NMR spectrum of **13** in $(CD_3)_2SO$.

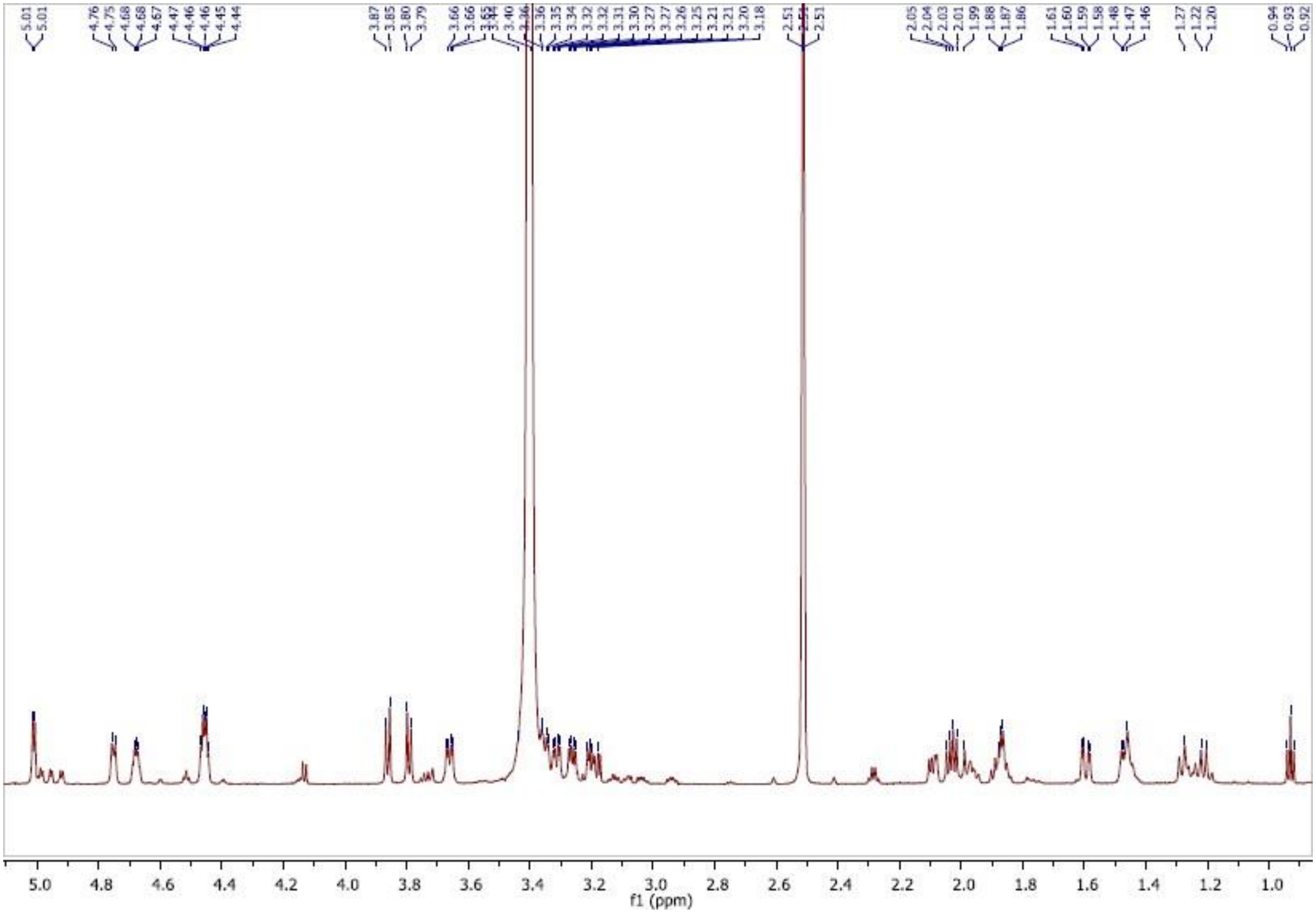

Figure S106. Expansion of part of ^{1}H NMR spectrum (δ 0.9 – δ 5.1) of **13** in $(CD_3)_2SO$.

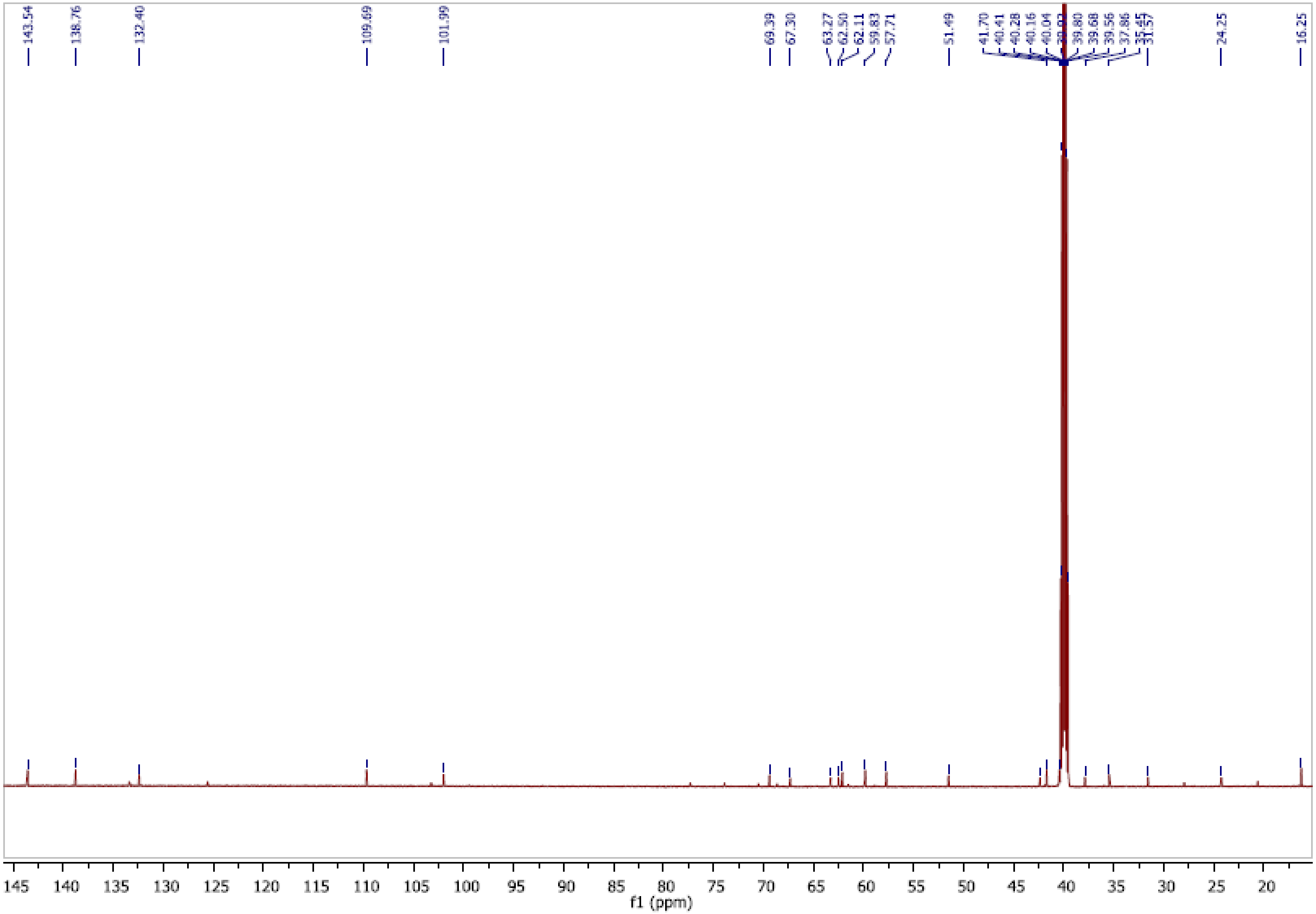

Figure S107. ^{13}C NMR spectrum of **13** in $(CD_3)_2SO$.

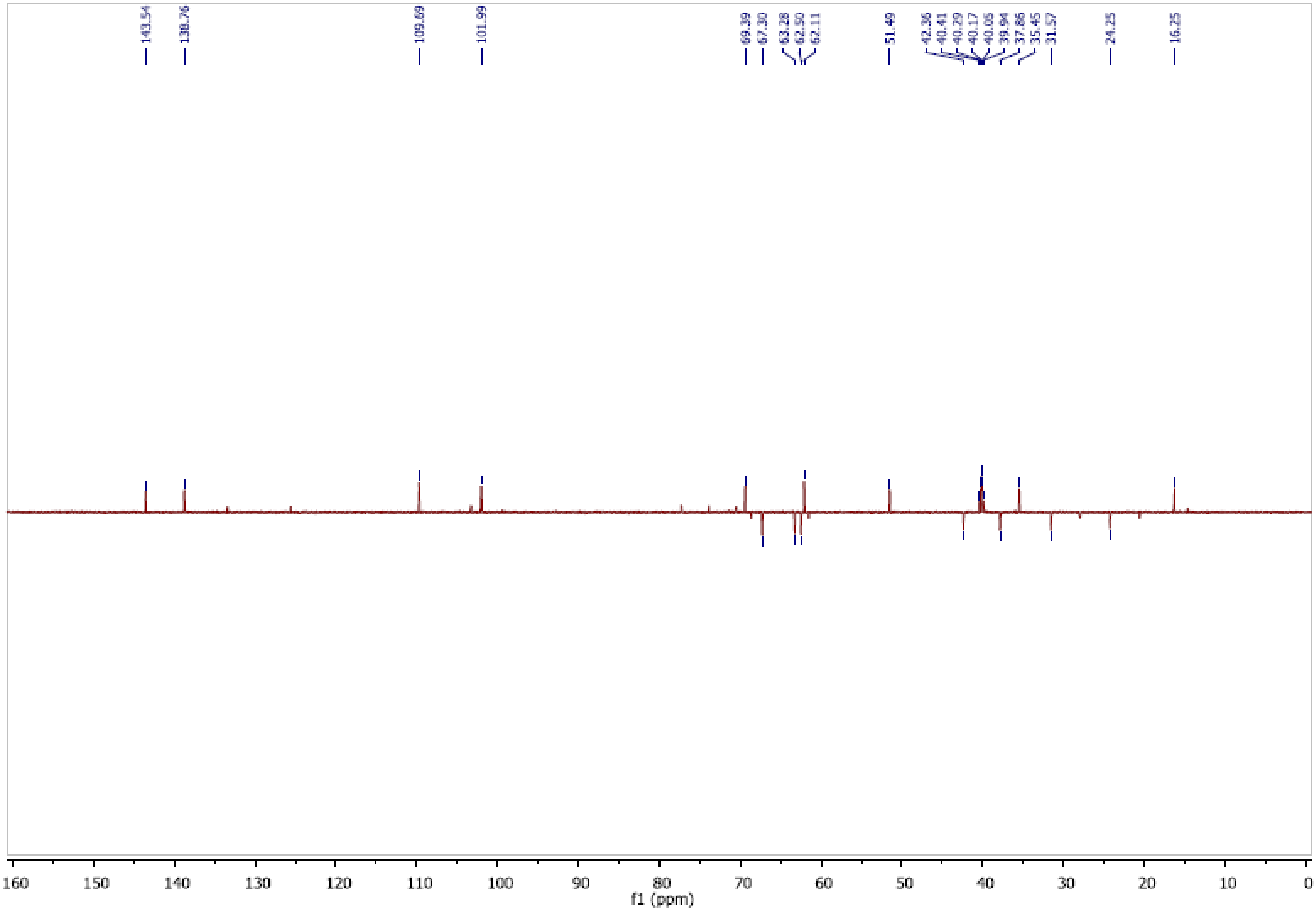

Figure S108. 135DEPT NMR spectrum of **13** in $(CD_3)_2SO$.

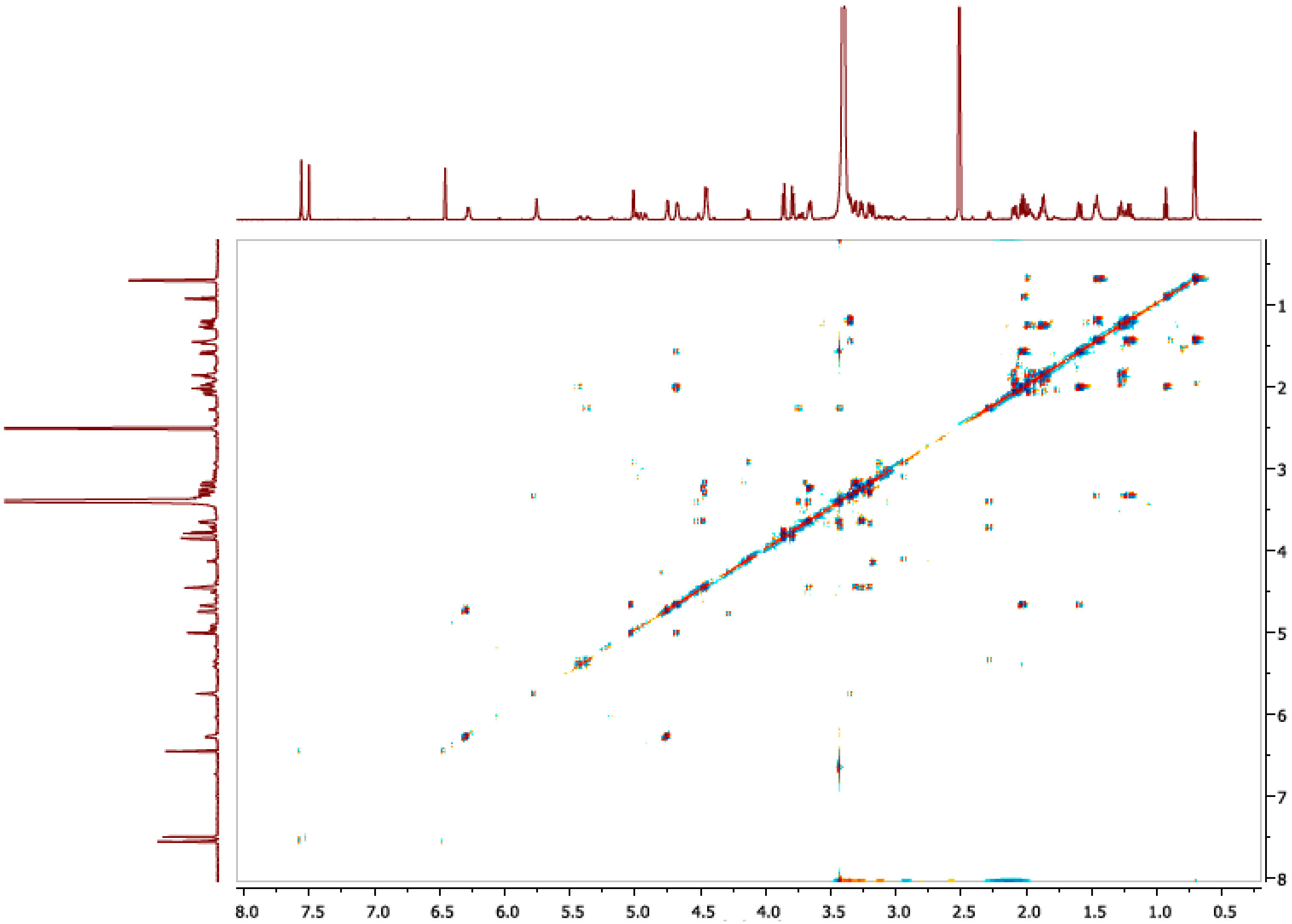

Figure S109. COSY NMR spectrum of **13** in $(CD_3)_2SO$.

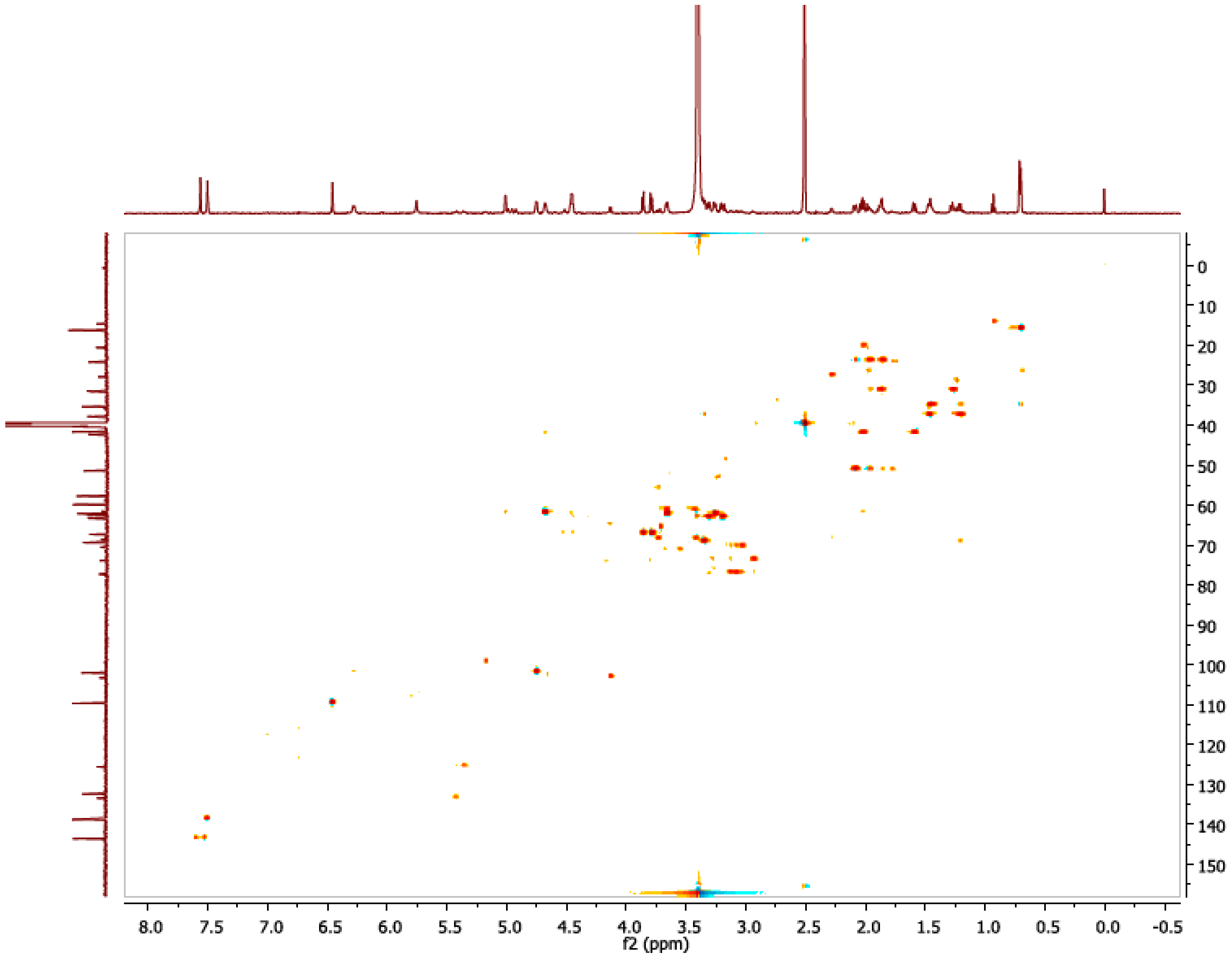

Figure S110. HSQC NMR spectrum of **13** in $(CD_3)_2SO$.

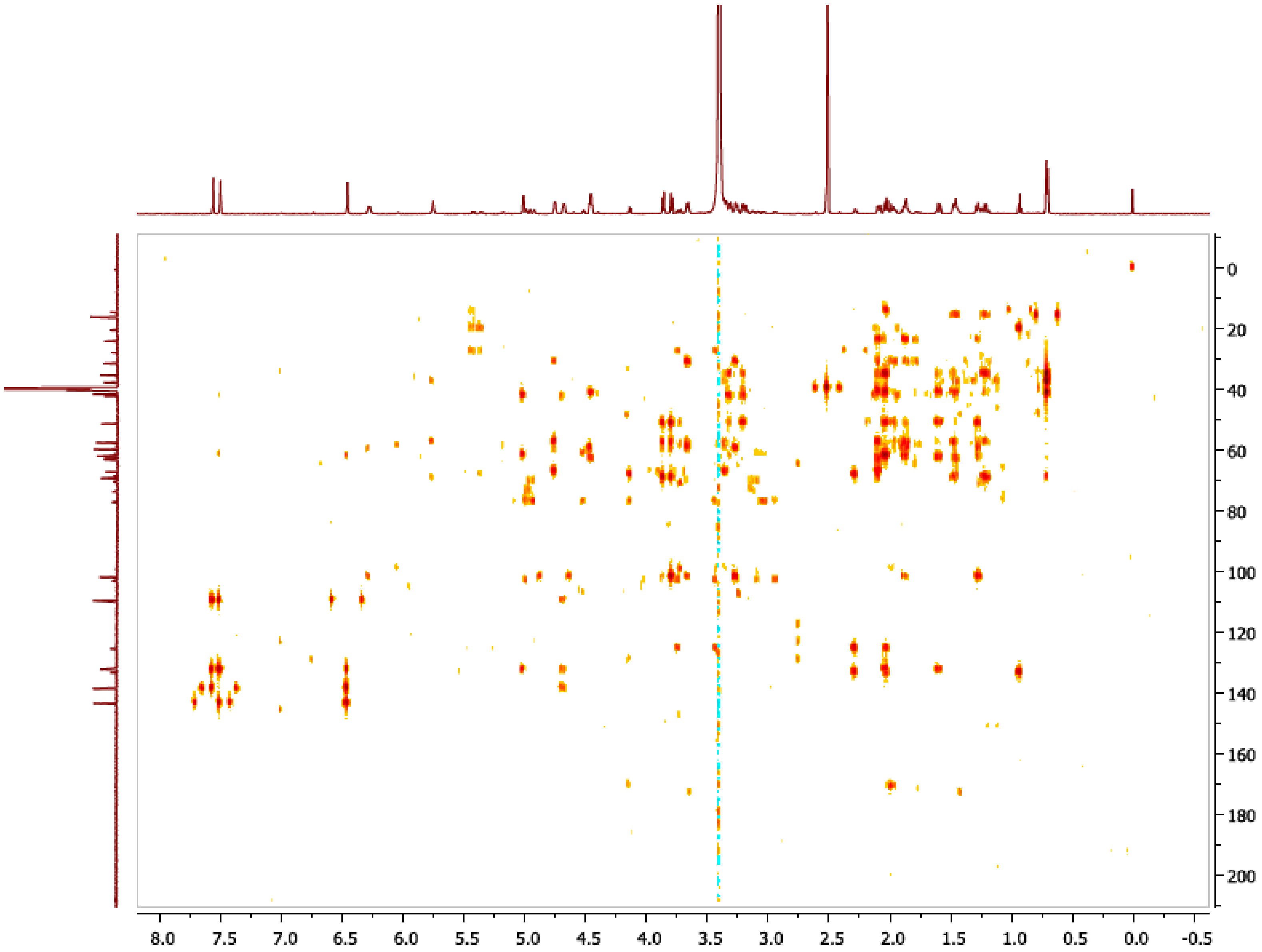

Figure S111. HMBC NMR spectrum of **13** in $(CD_3)_2SO$.

R13 #232-252 RT: 3.34-3.58 AV: 10 NL: 1.43E7
F: FTMS + p ESI Full ms [100.0000-2000.0000]

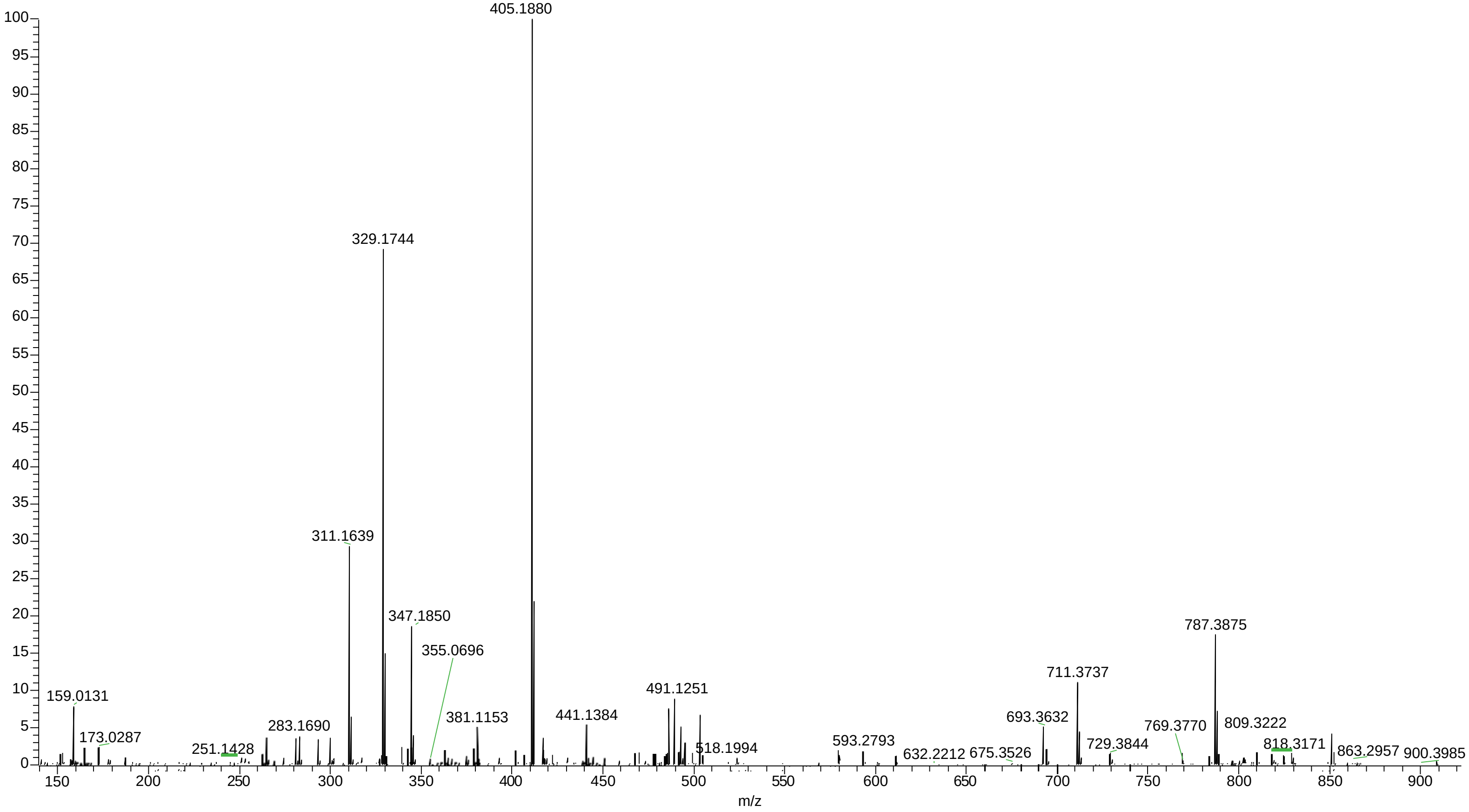

Figure S112. Positive-ion HRESIMS of **13**.

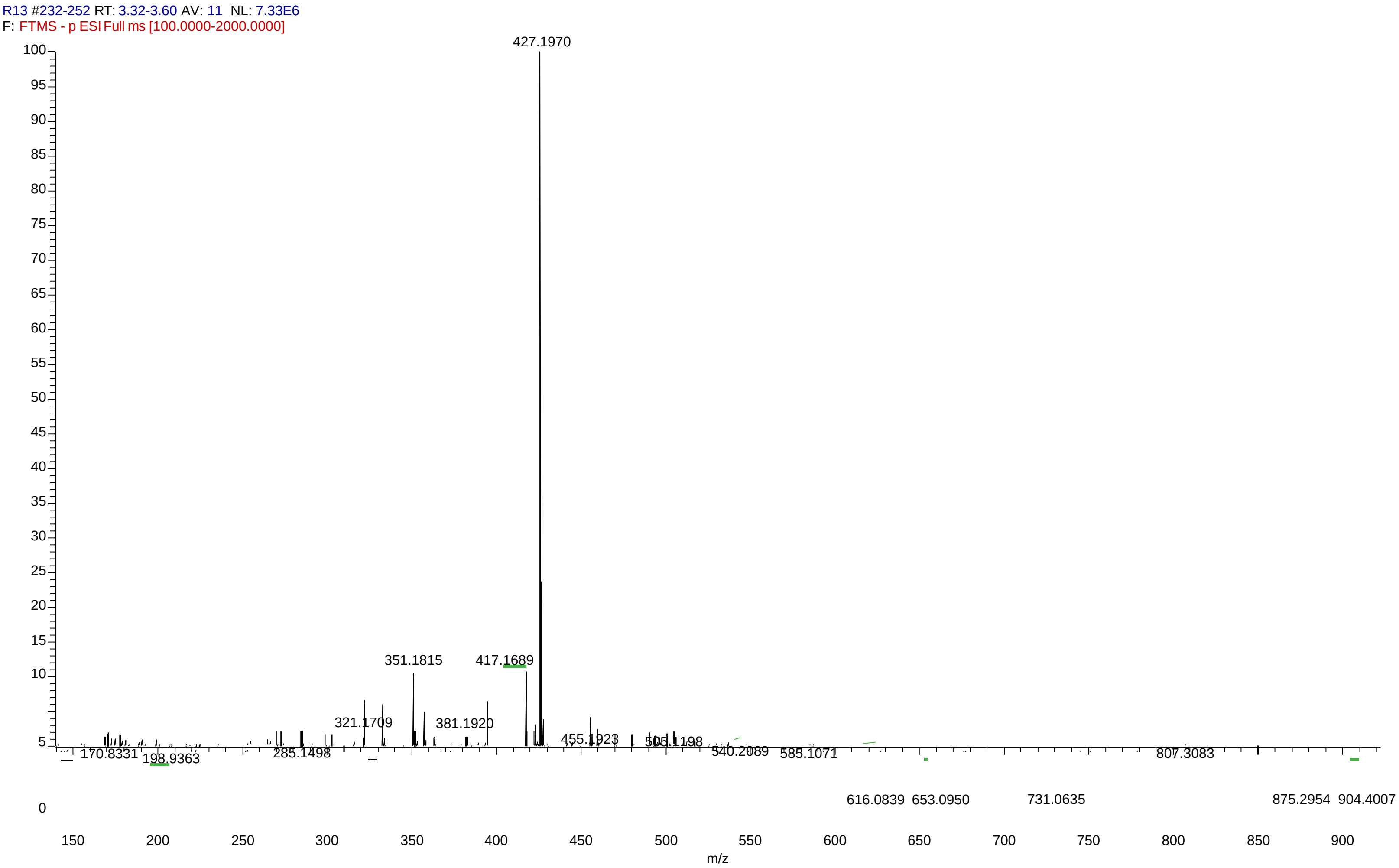
R13 #232-252 RT: 3.32-3.60 AV: 11 NL: 7.33E6
F: FTMS - p ESI Full ms [100.0000-2000.0000]
427.1970
351.1815
417.1689
321.1709
381.1920
455.1923
505.1198
540.2089
585.1071
170.8331
198.9363
285.1498
807.3083
616.0839
653.0950
731.0635
875.2954
904.4007
m/z

Figure S113. Negative-ion HRESIMS of **13**.

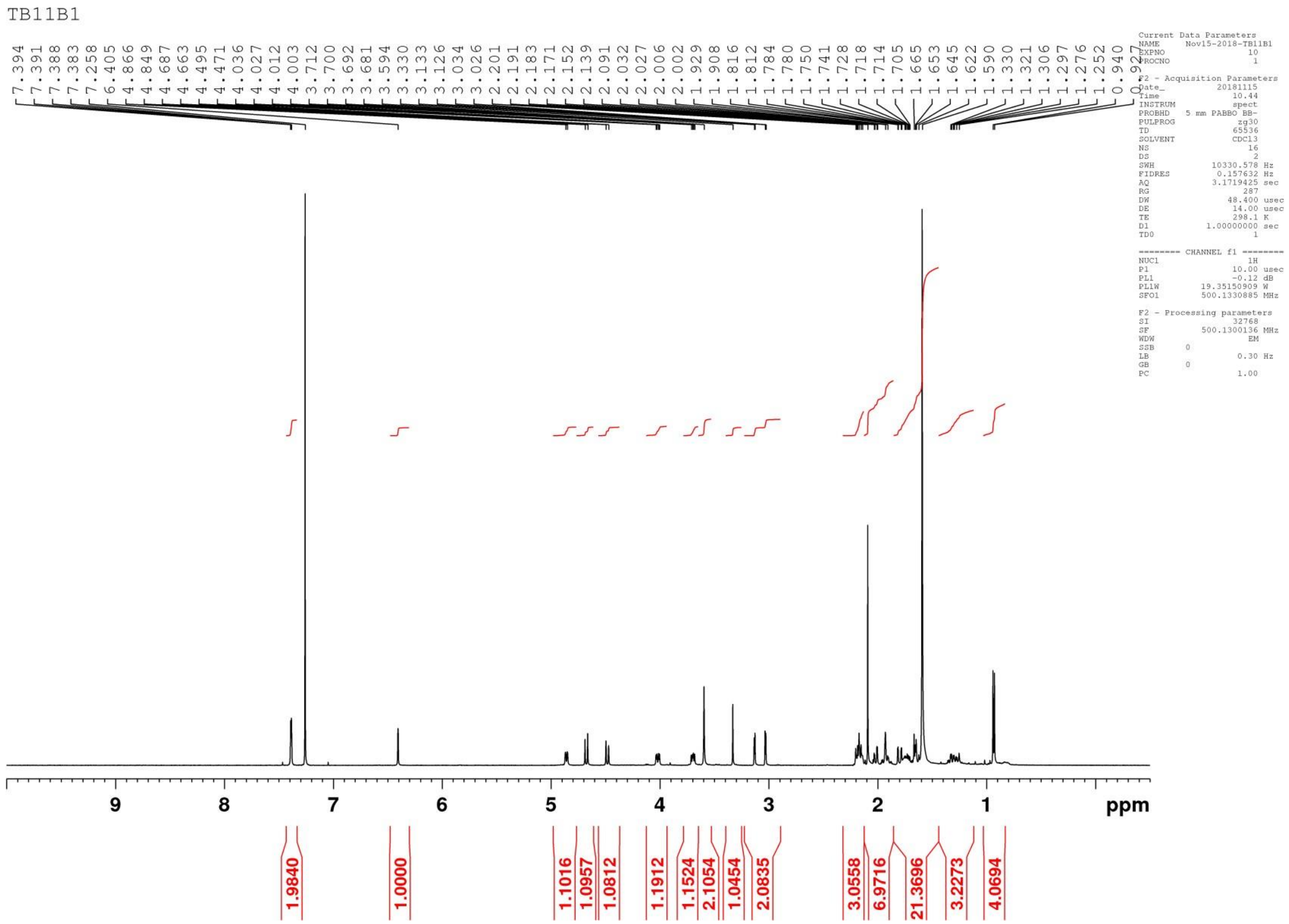

Figure S114. ^{1}H NMR spectrum of **14** in $CDCl_3$.

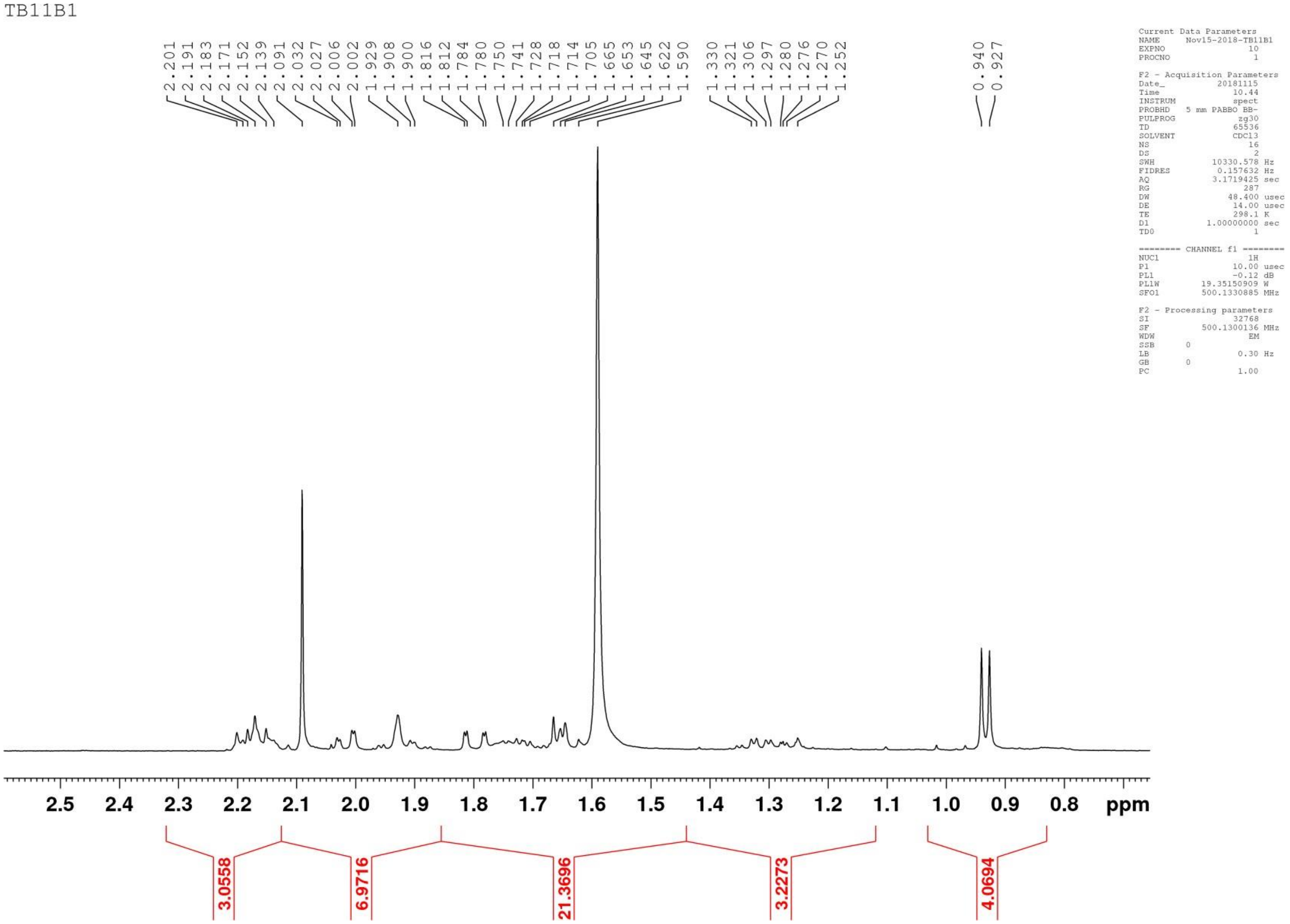

Figure S115. Expansion of part of ^{1}H NMR spectrum (δ 0.7 – δ 2.5) of **14** in $CDCl_3$.

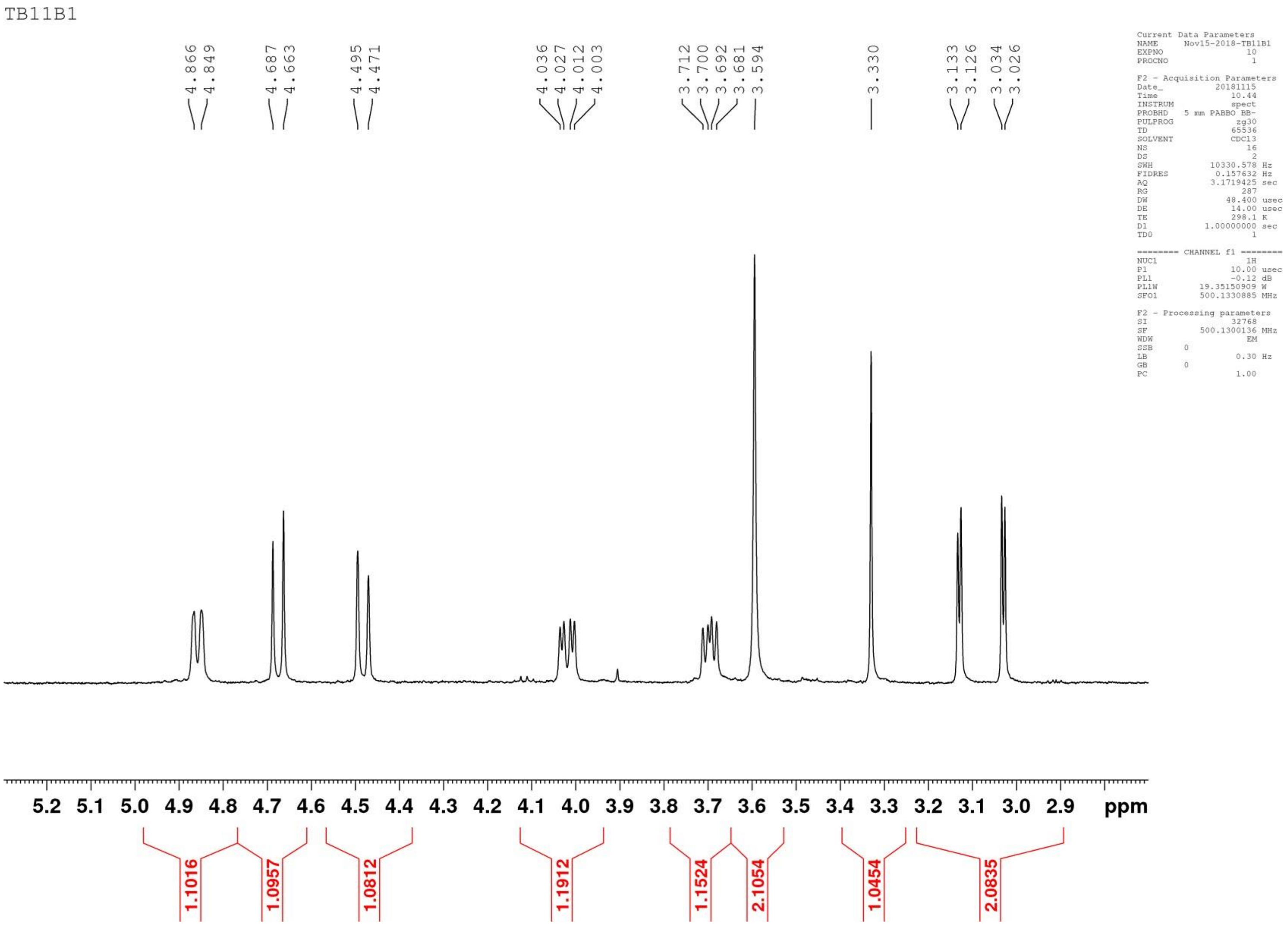

Figure S116. Expansion of part of ^{1}H NMR spectrum (δ 2.8 – δ 5.2) of **14** in $CDCl_3$.

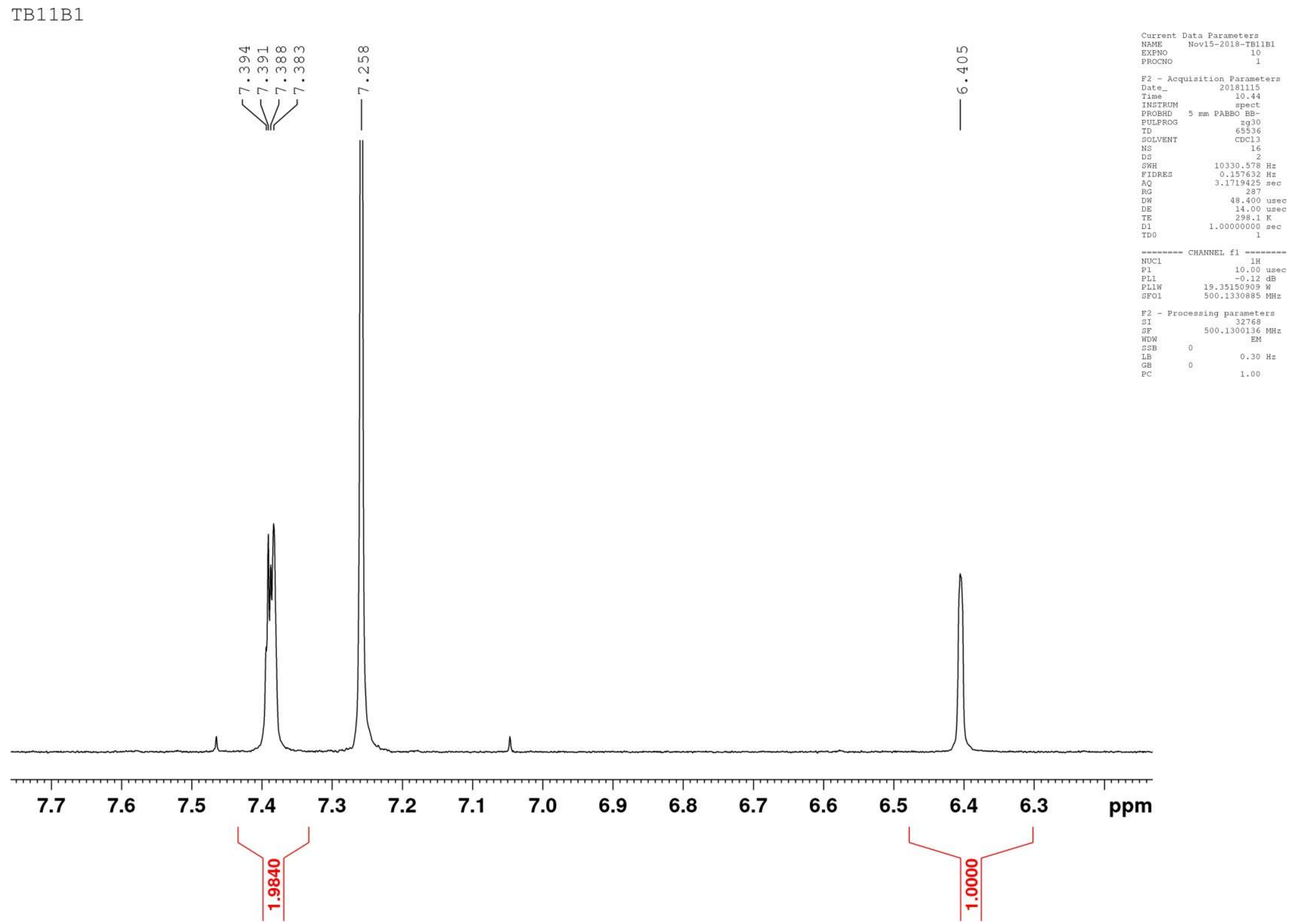

Figure S117. Expansion of part of ^{1}H NMR spectrum (δ 6.2 – δ 7.7) of **14** in $CDCl_3$.

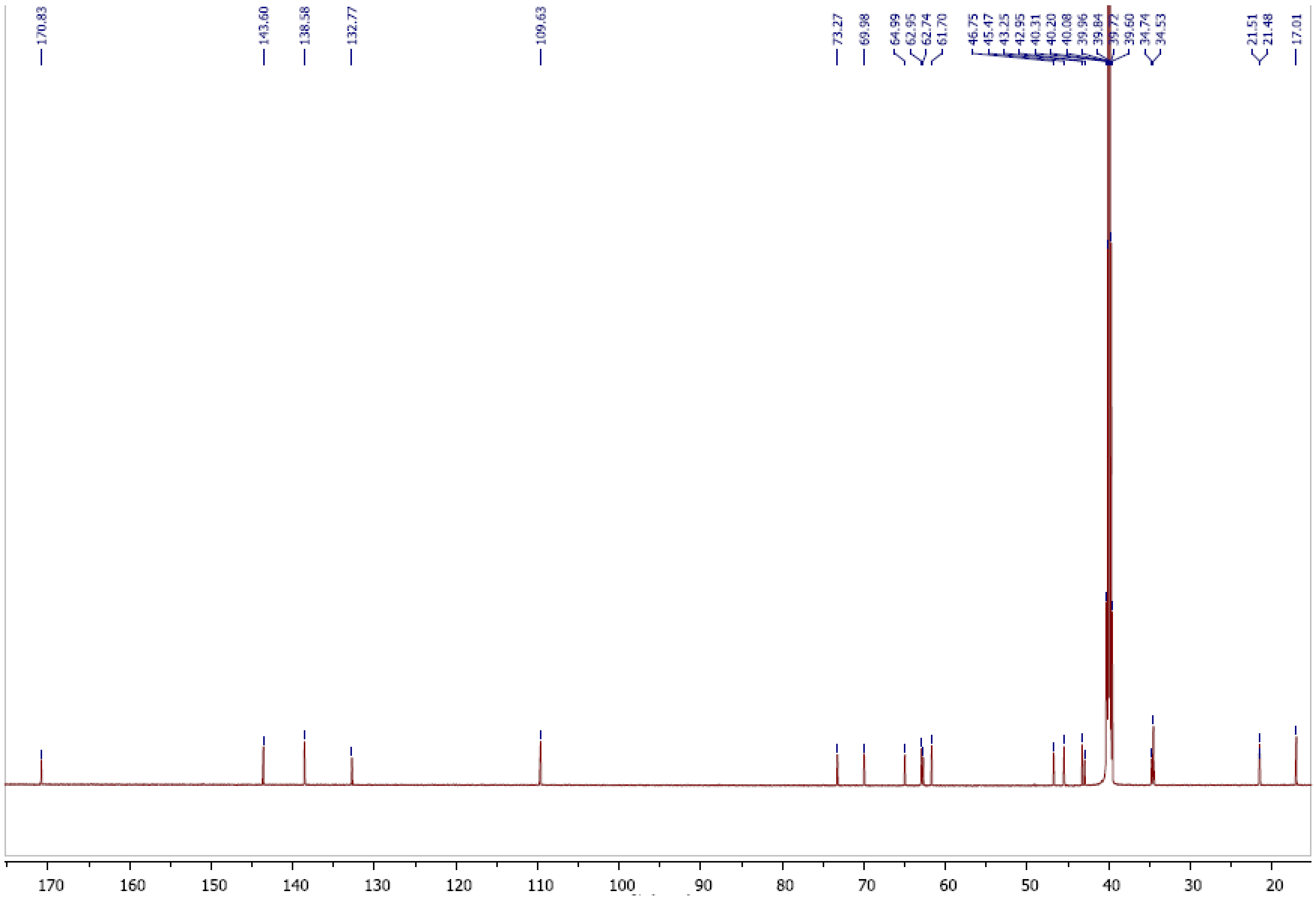

Figure S118. ^{13}C NMR spectrum of **14** in $(CD_3)_2SO$.

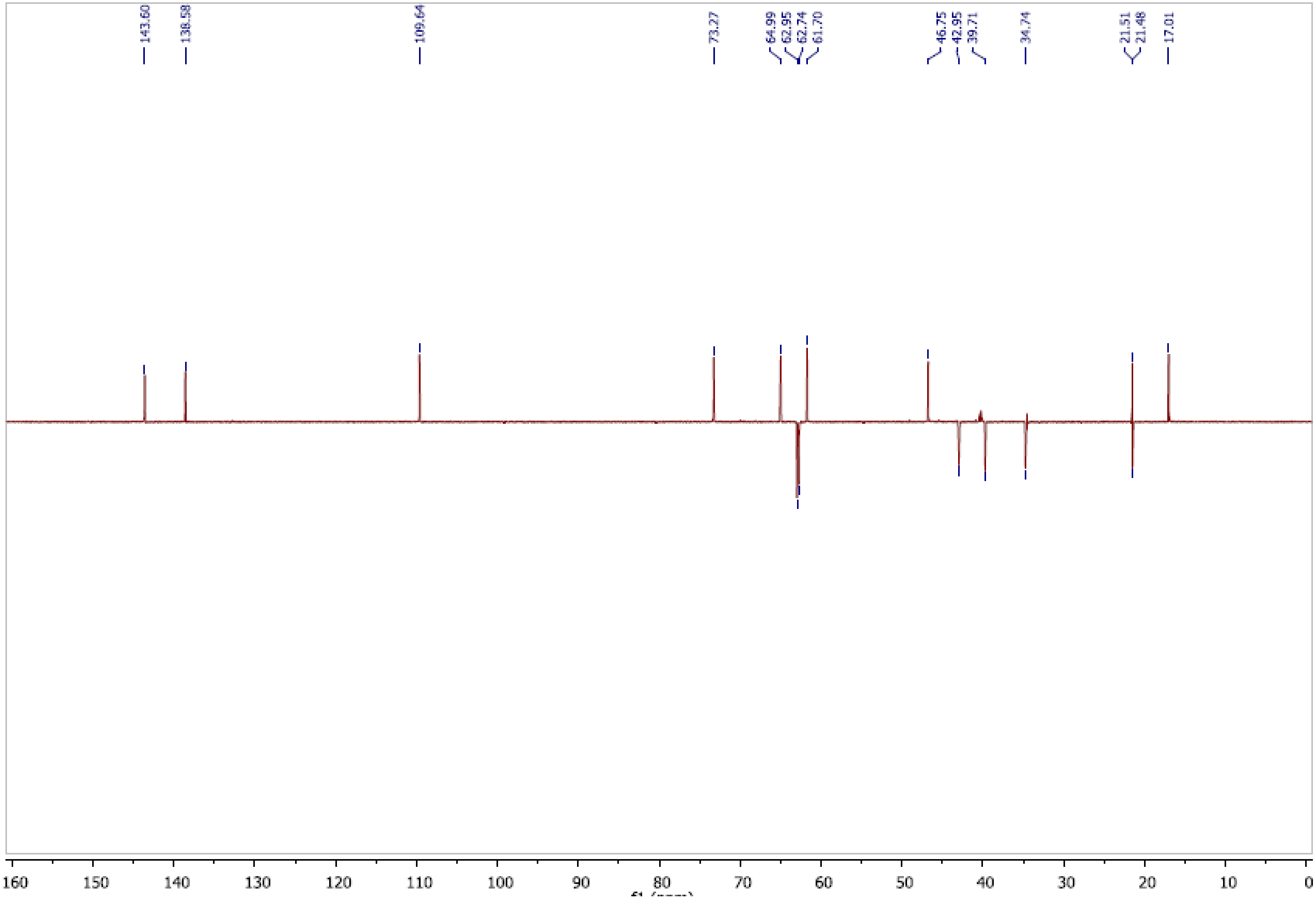

Figure S119. 135DEPT NMR spectrum of **14** in $(CD_3)_2SO$.

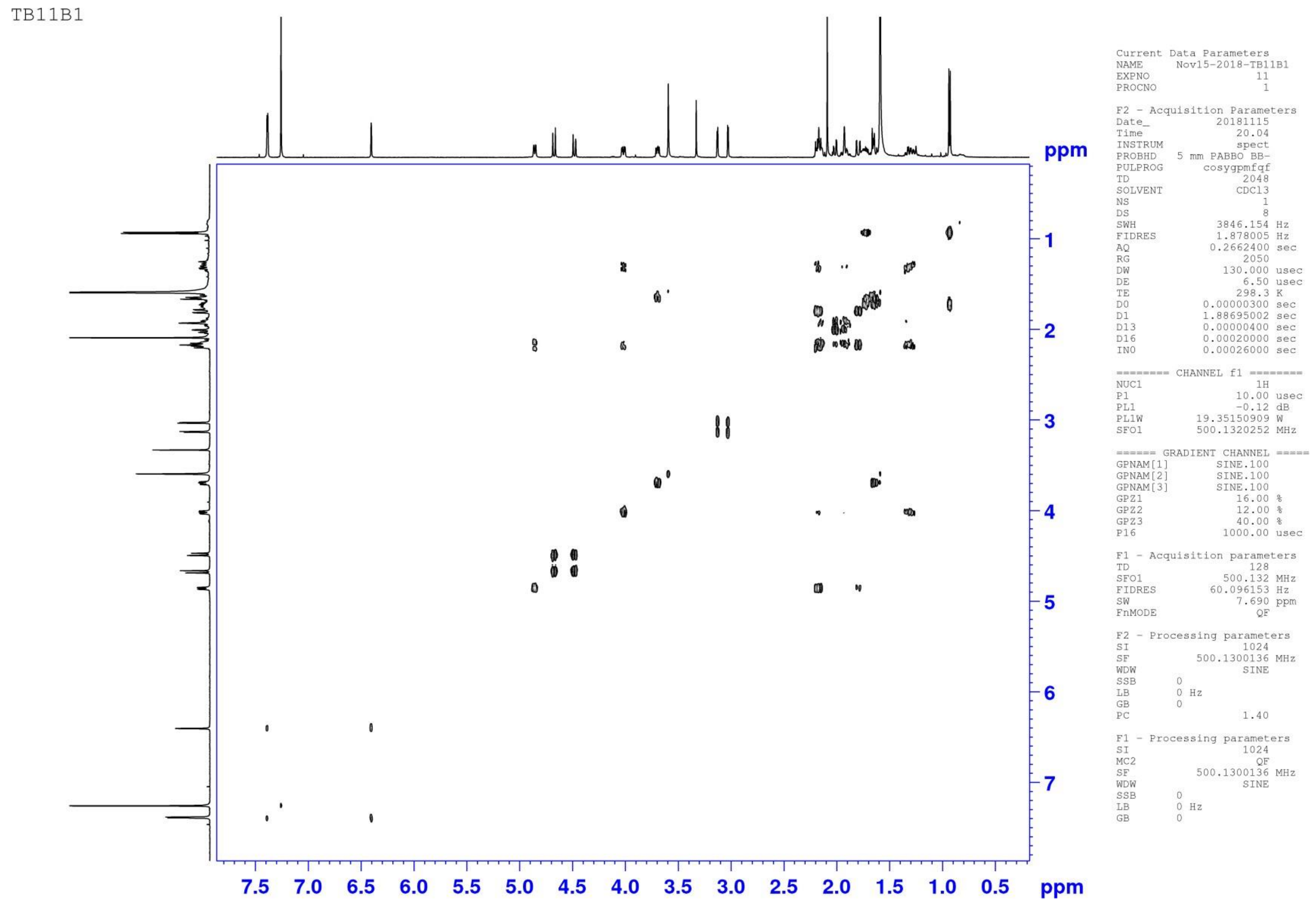

Figure S120. COSY ^{1}H NMR spectrum of **14** in $CDCl_3$.

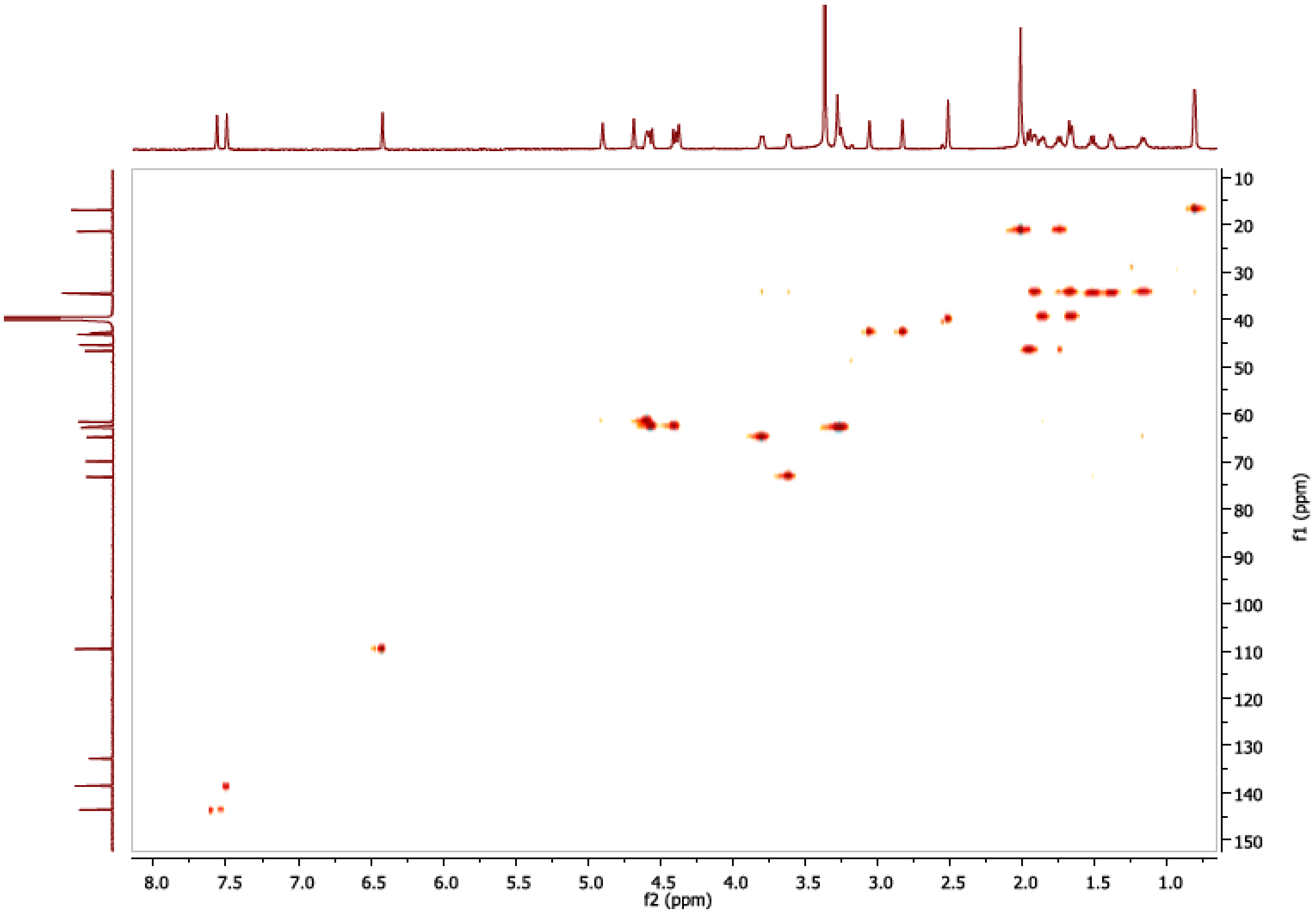

Figure S121. HSQC NMR spectrum of **14** in $(CD_3)_2SO$.

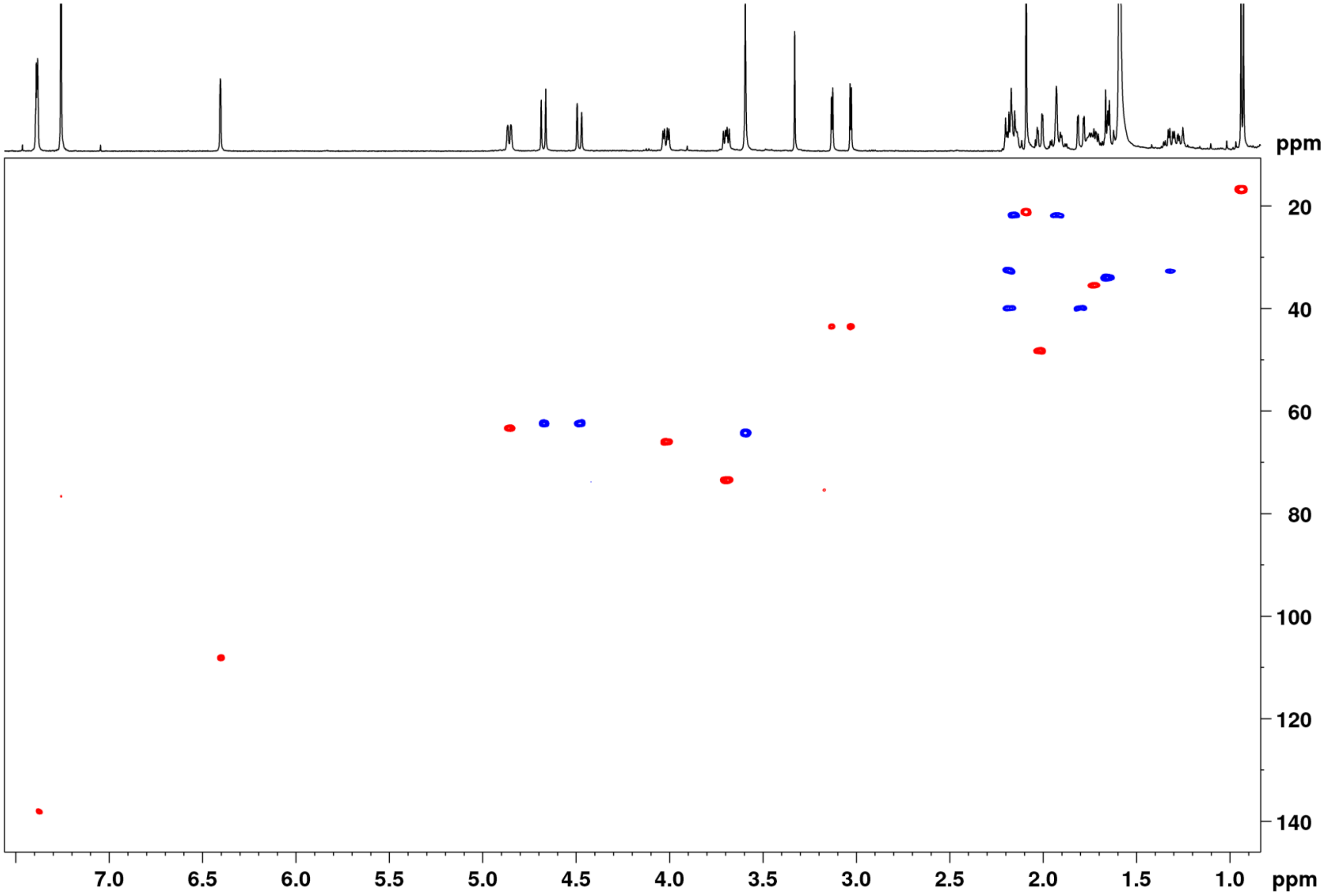

Figure S122. HSQC ^{1}H NMR spectrum of **14** in $CDCl_3$.

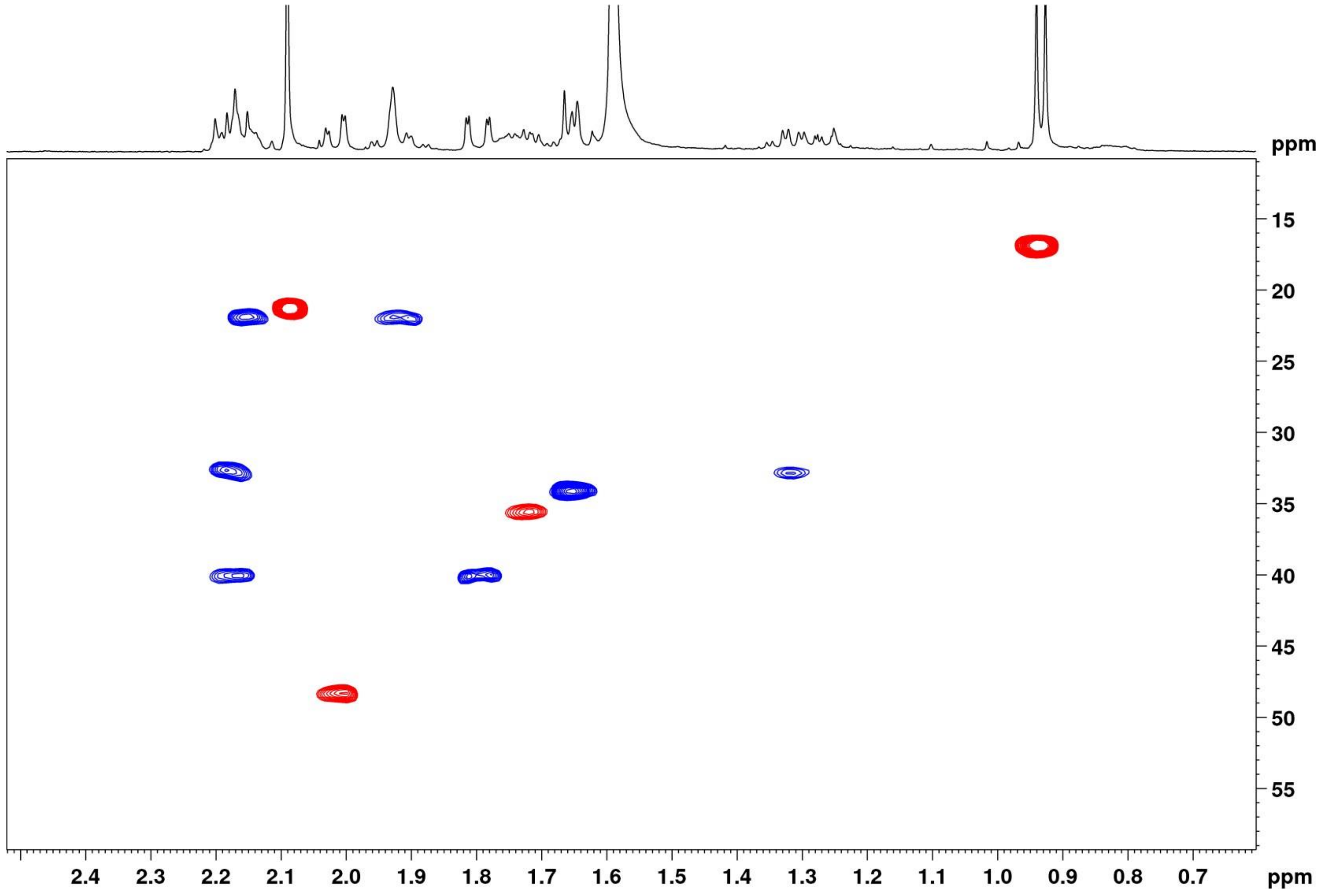

Figure S123. Expansion of part of HSQC NMR spectrum (δ 0.7 – δ 2.5) of **14** in $CDCl_3$.

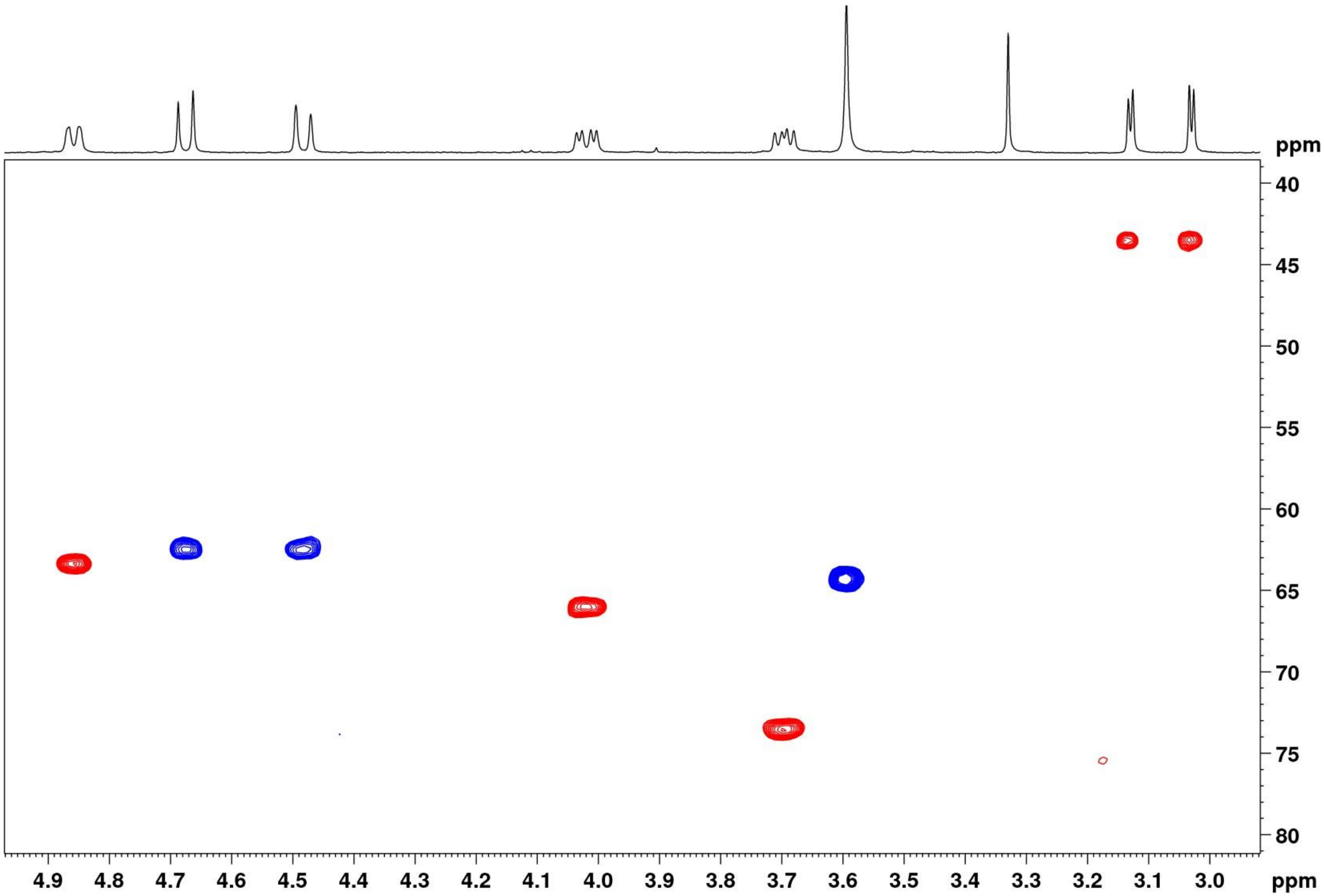

Figure S124. Expansion of part of HSQC NMR spectrum (δ 3.0 – δ 4.9) of **14** in $CDCl_3$.

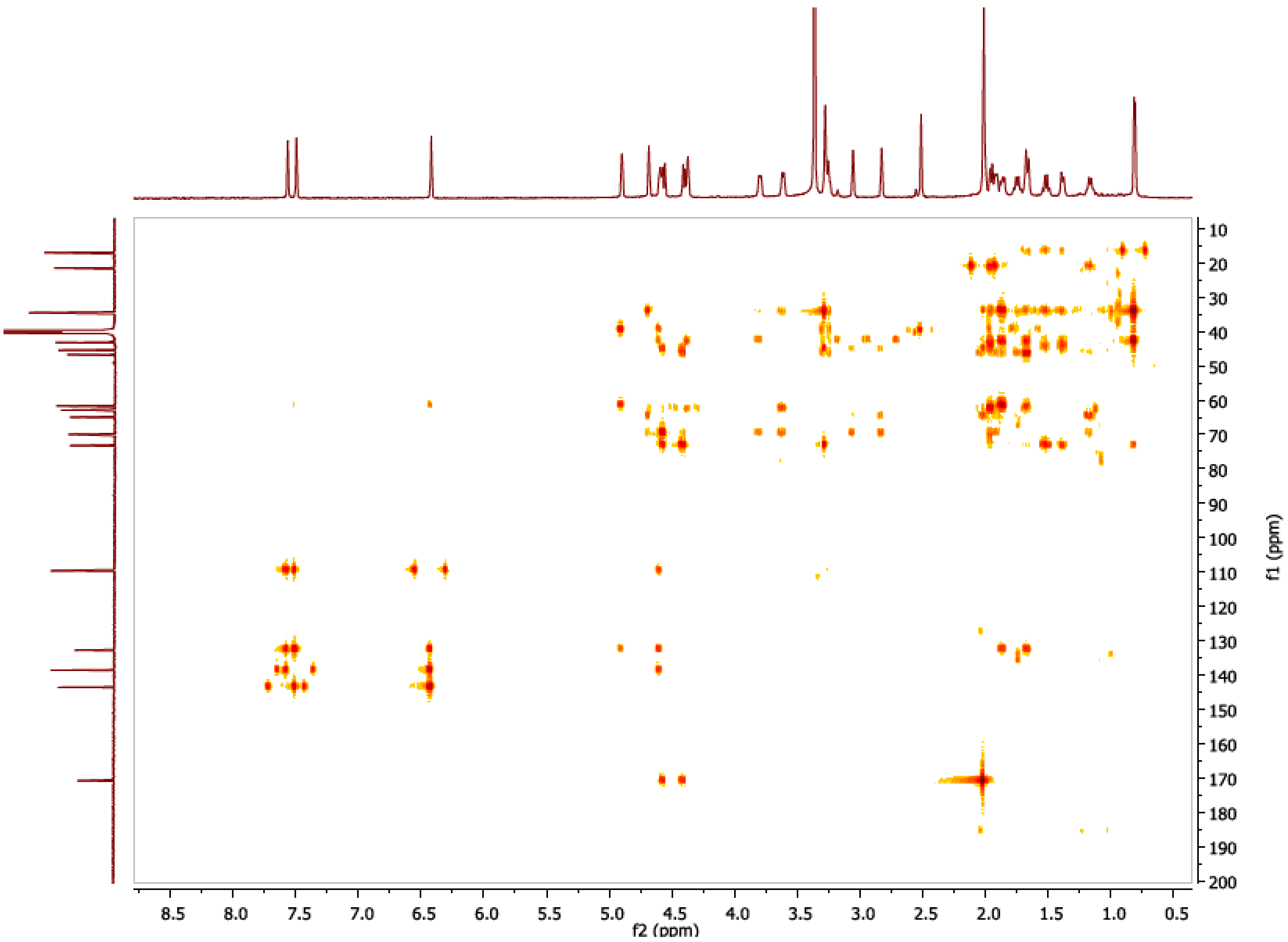

Figure S125. HMBC NMR spectrum of **14** in $(CD_3)_2SO$.

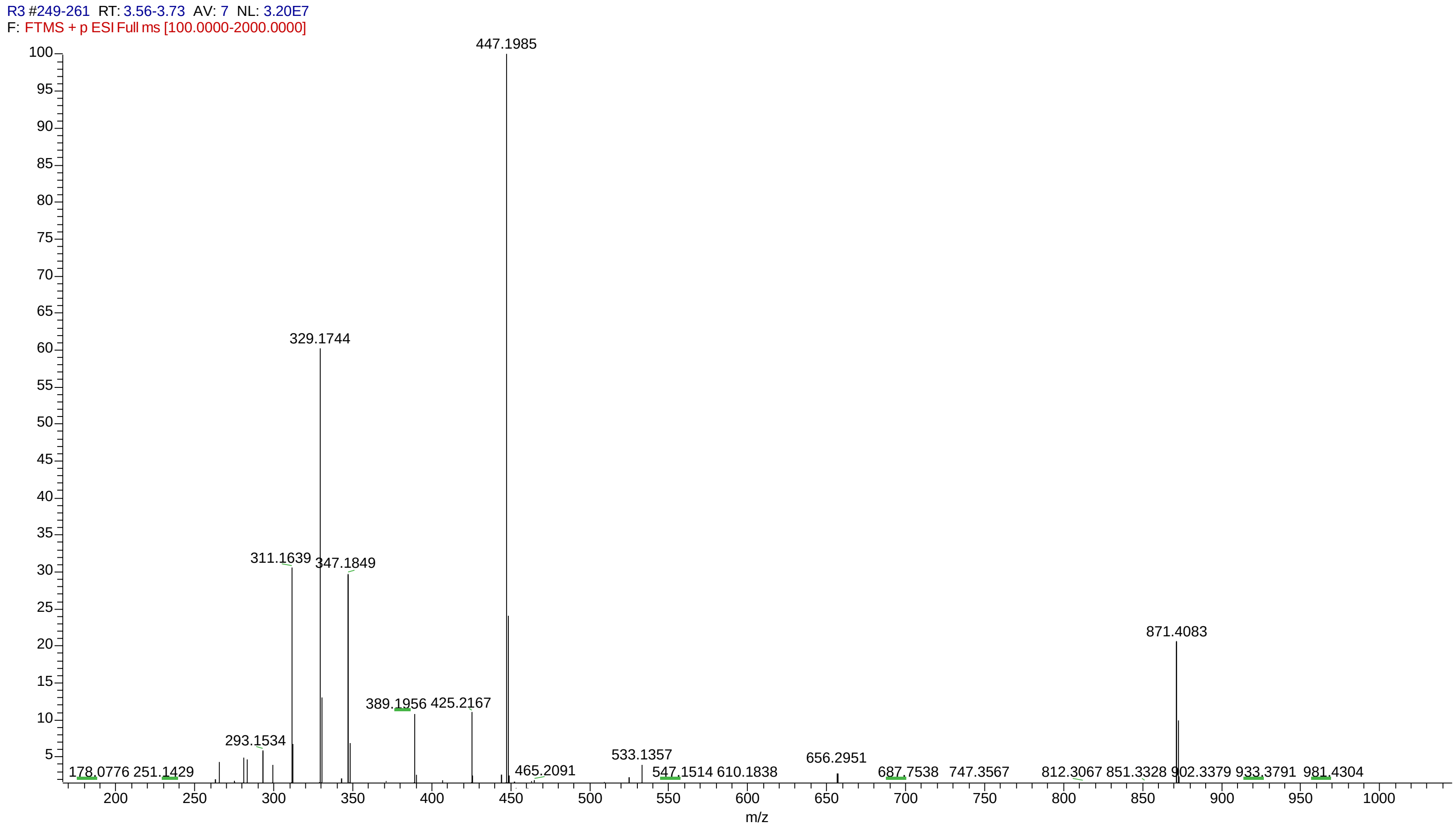

Figure S126. Positive-ion HRESIMS of **14**.

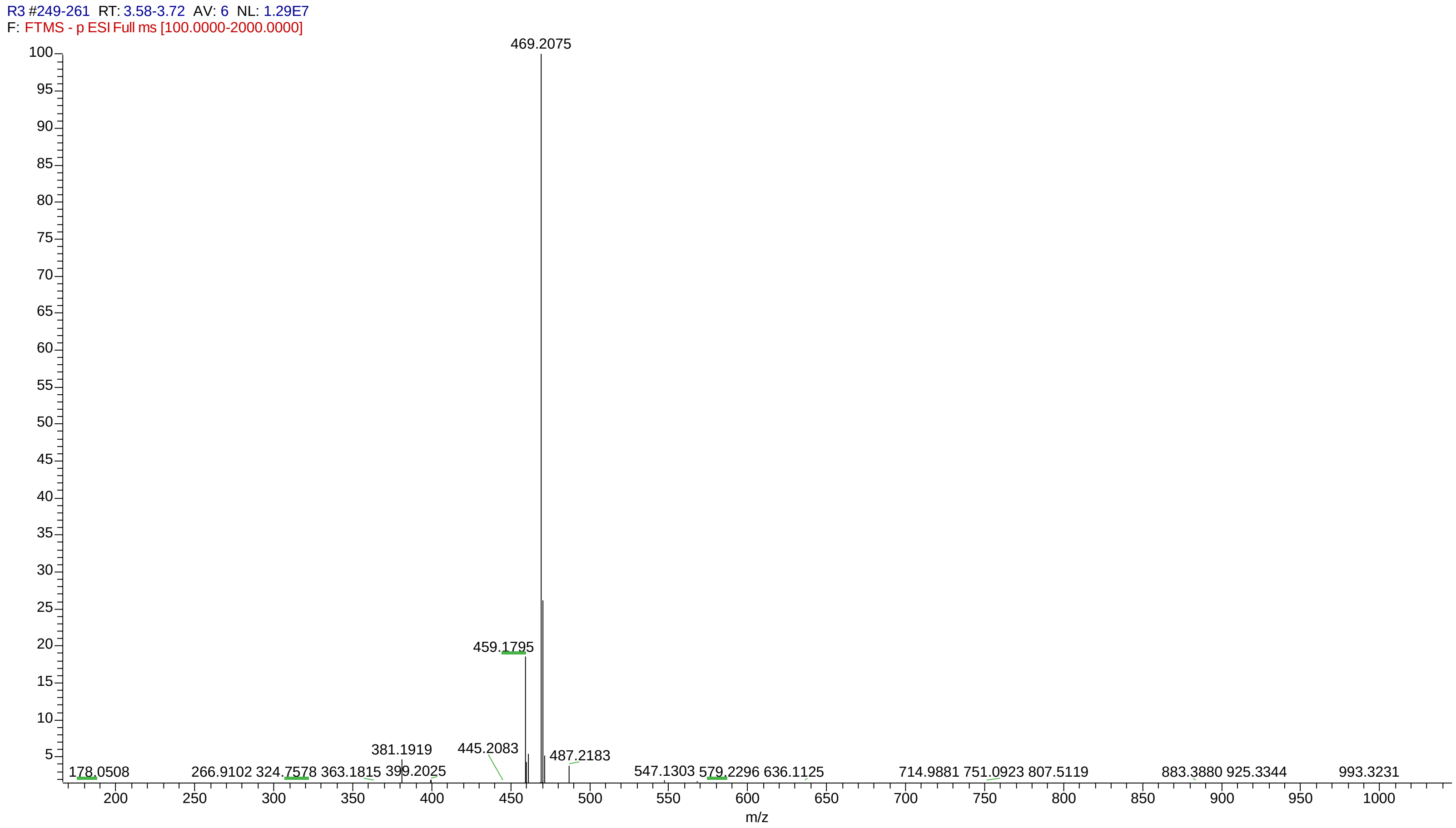

Figure S127. Negative-ion HRESIMS of **14**.

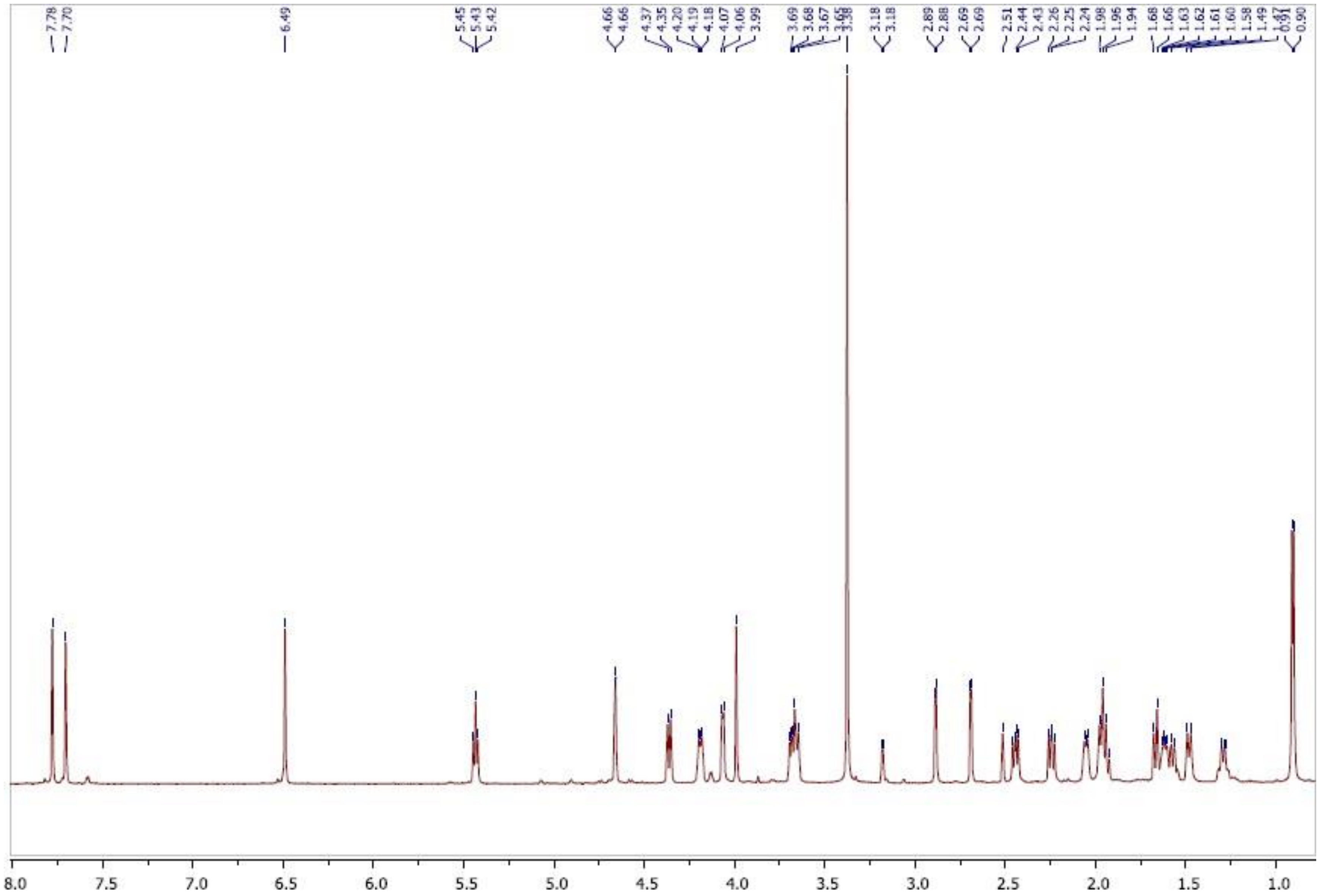

Figure S128. ^{1}H NMR spectrum of **15** in $(CD_3)_2SO$.

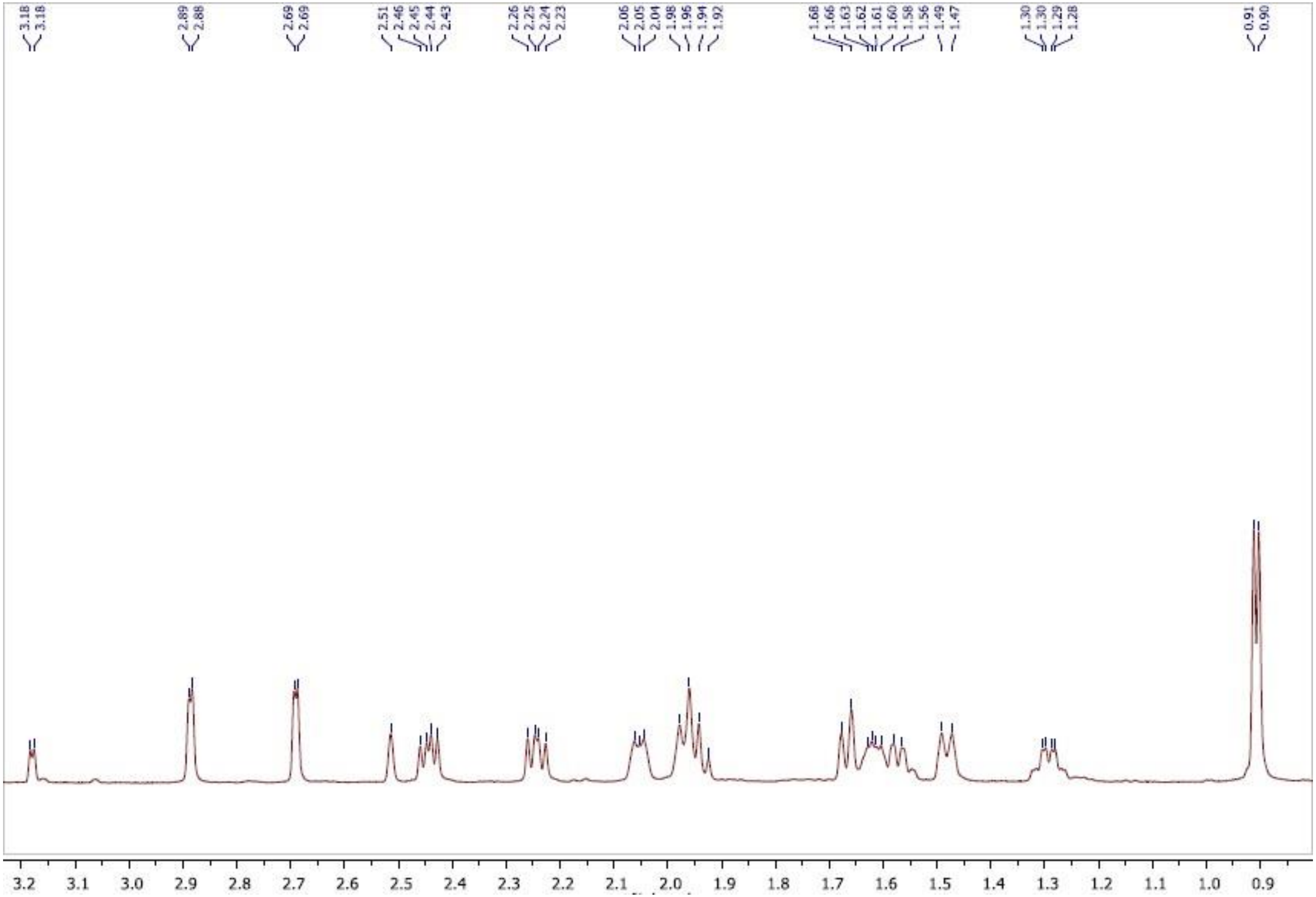

Figure S129. Expansion of part of ^{1}H NMR spectrum (δ 0.8 – δ 3.2) of **15** in $(CD_3)_2SO$.

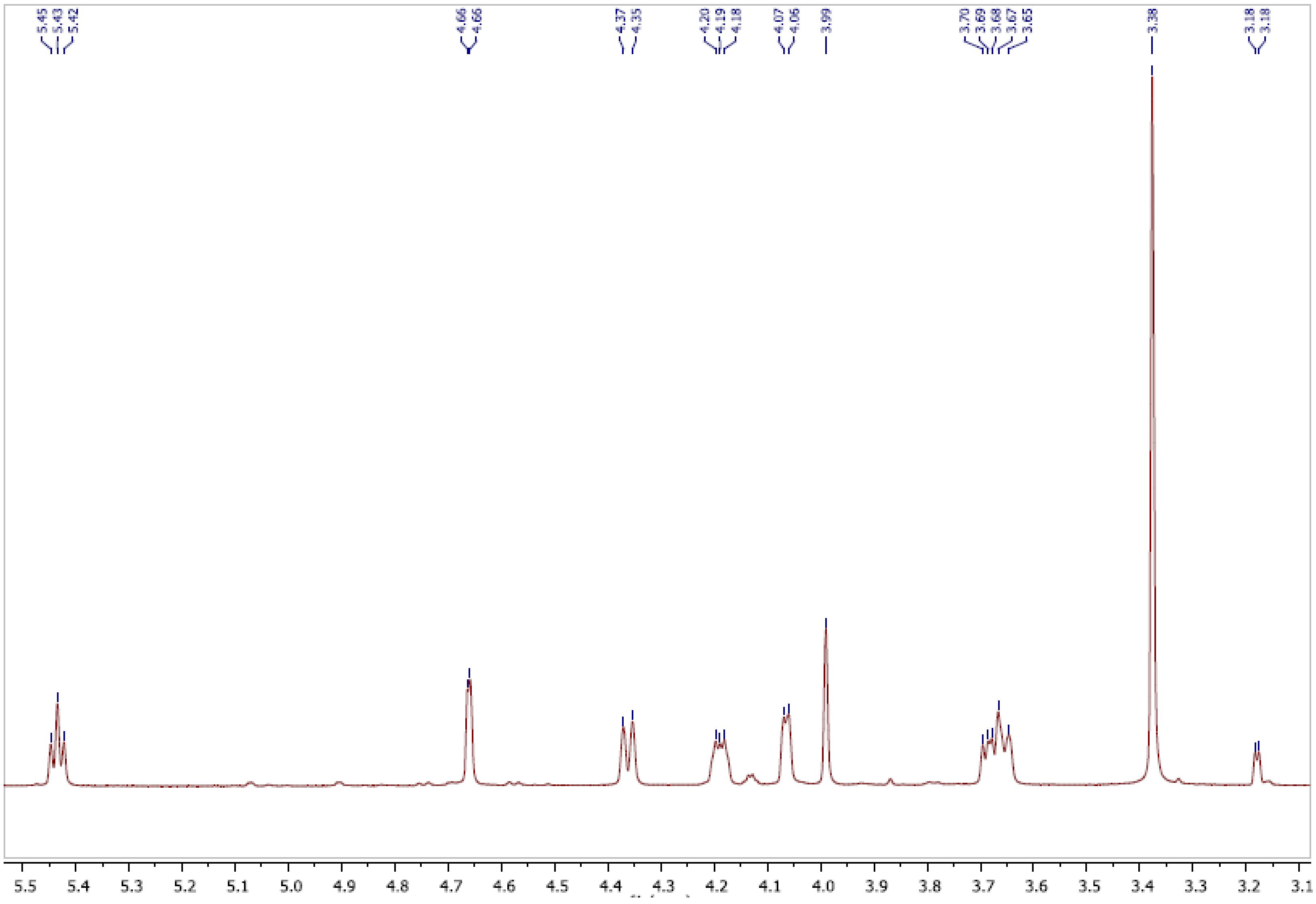

Figure S130. Expansion of part of ^{1}H NMR spectrum (δ 3.1 – δ 5.5) of **15** in $(CD_3)_2SO$.

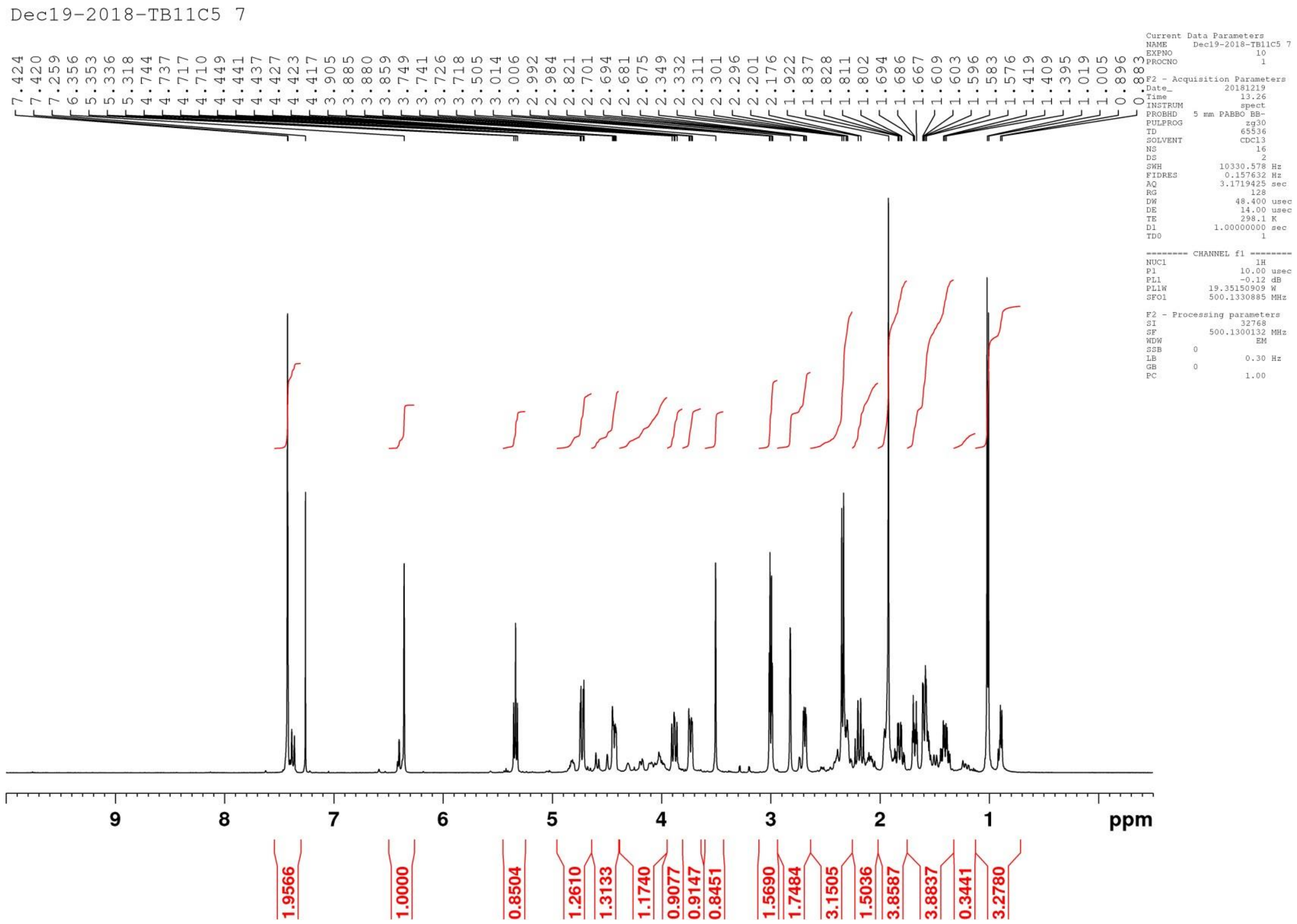

Figure S131. ^{1}H NMR spectrum of **15** in $CDCl_3$.

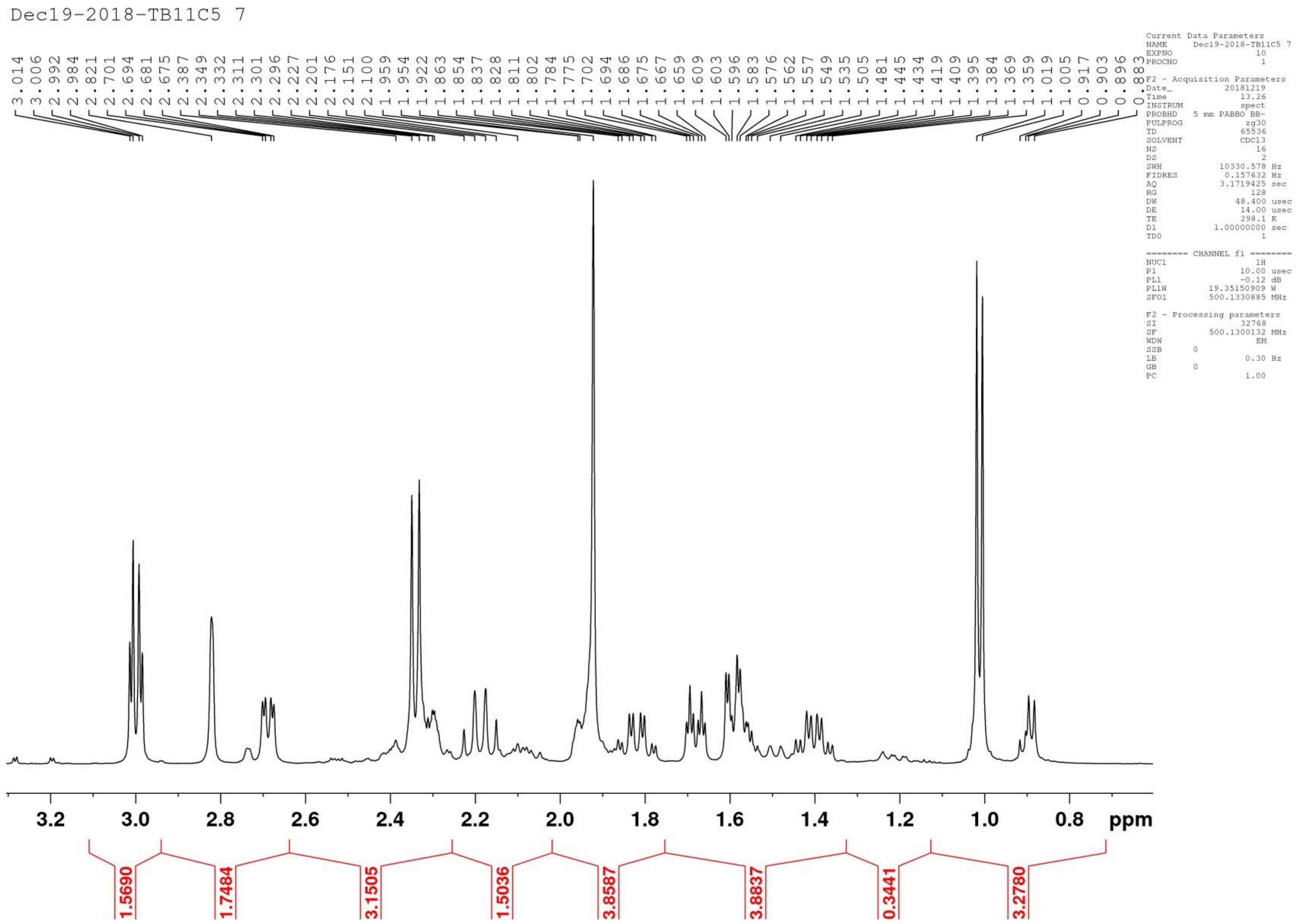

Figure S132. Expansion of part of ^{1}H NMR spectrum (δ 0.8 – δ 3.2) of **15** in $CDCl_3$.

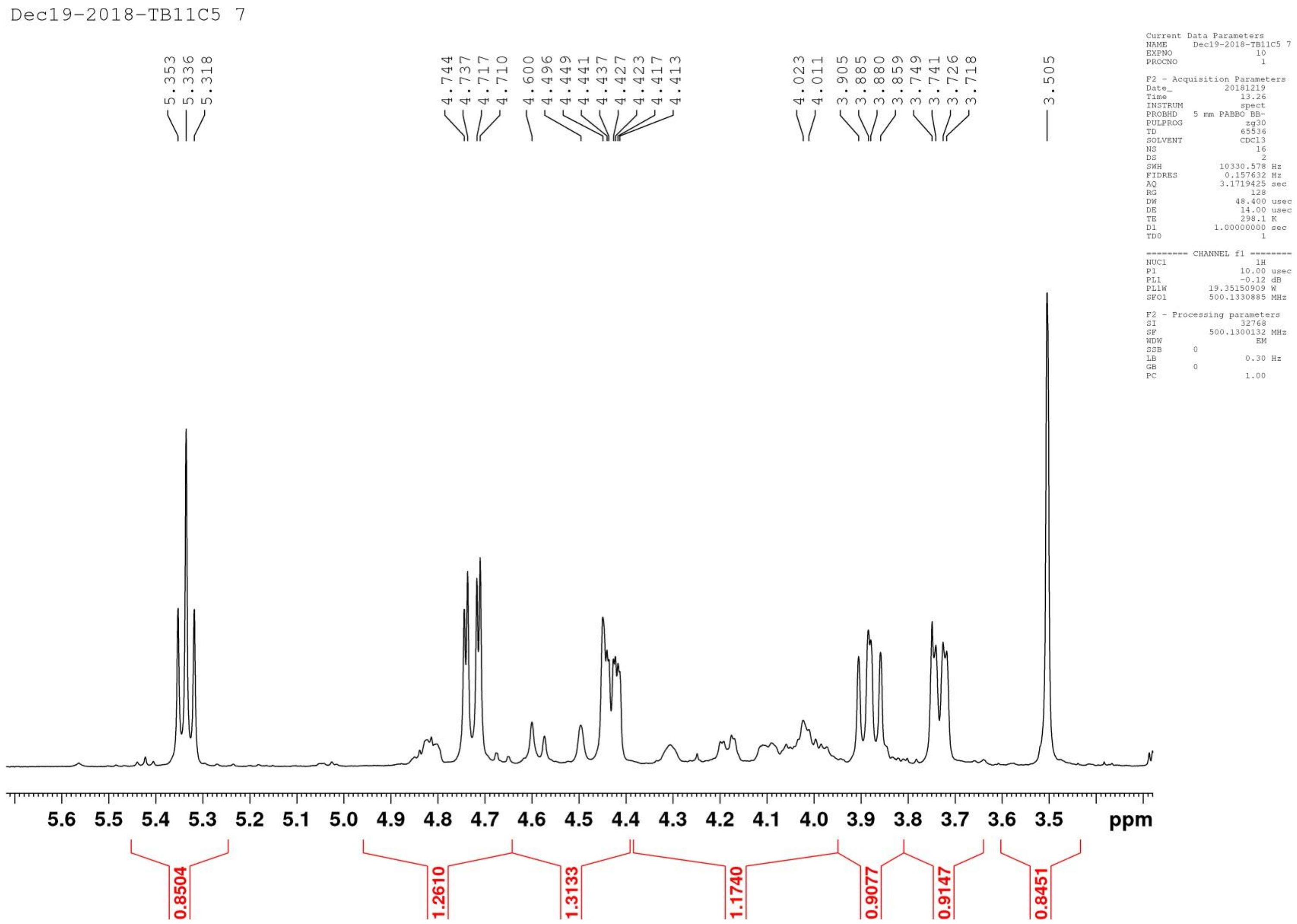

Figure S133. Expansion of part of ^{1}H NMR spectrum (δ 3.3 – δ 5.6) of **15** in $CDCl_3$.

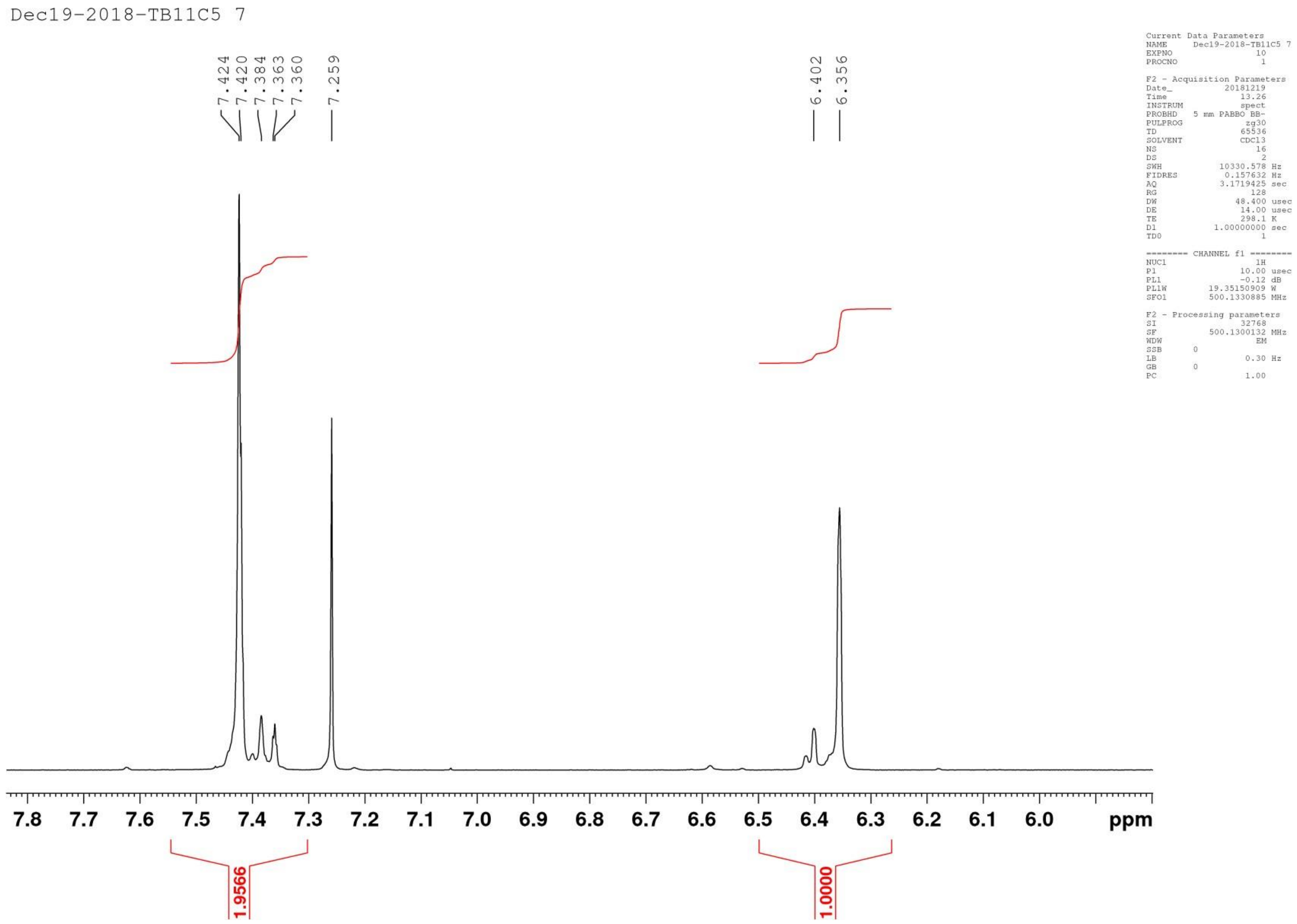

Figure S134. Expansion of part of ^{1}H NMR spectrum (δ 5.8 – δ 7.8) of **15** in $CDCl_3$.

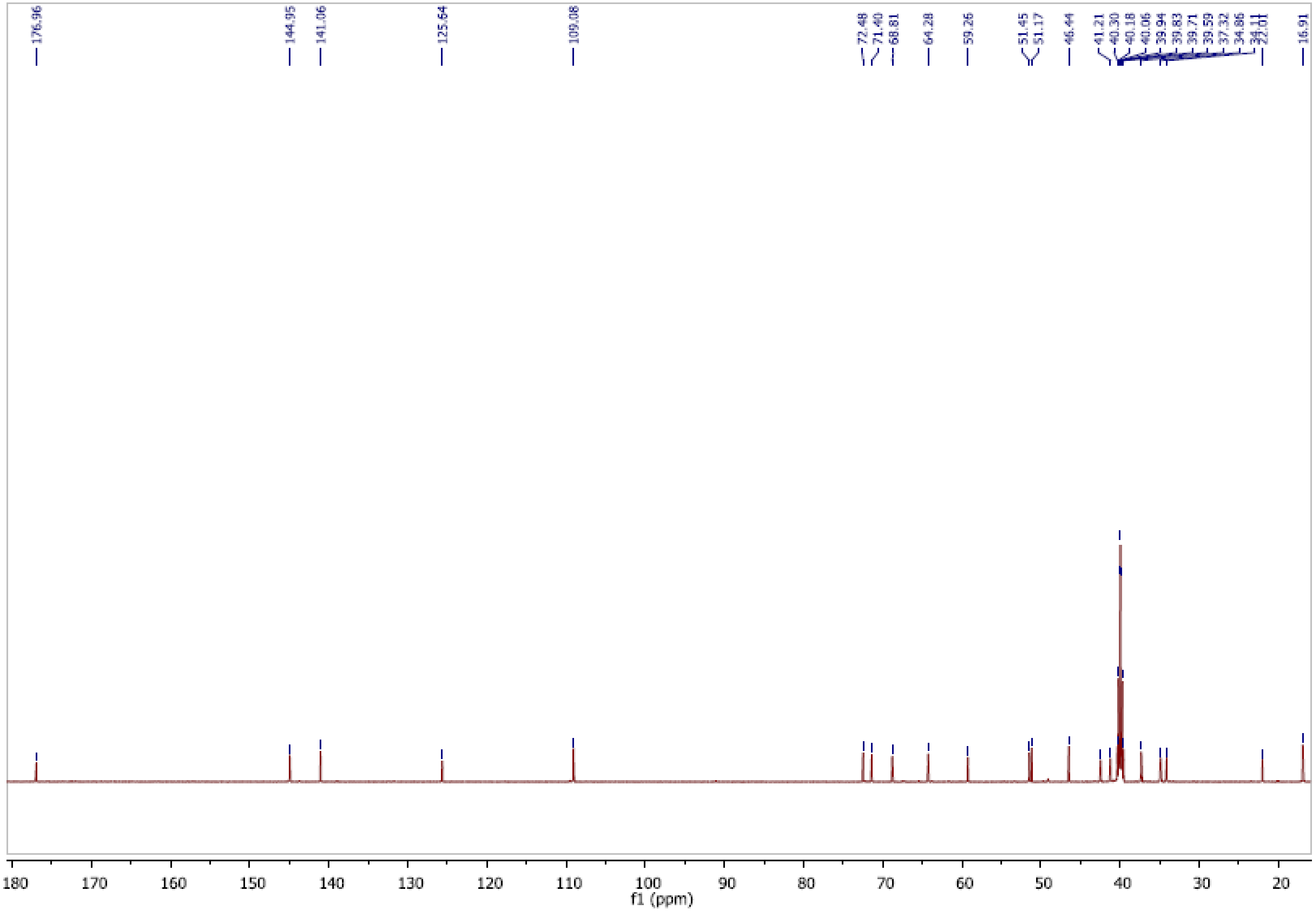

Figure S135. ^{13}C NMR spectrum of **15** in $(CD_3)_2SO$.

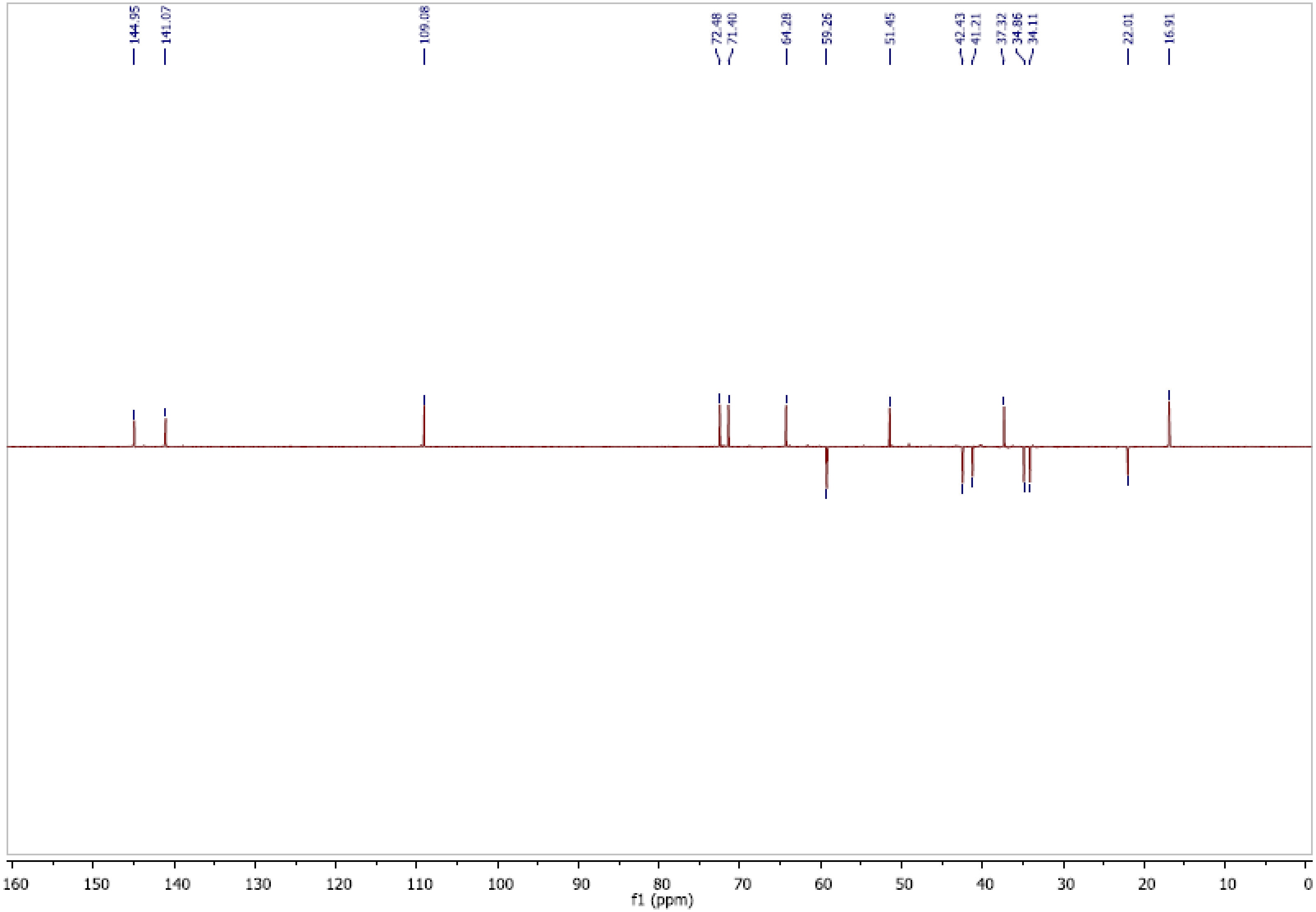

Figure S136. 135DEPT NMR spectrum of **15** in $(CD_3)_2SO$.

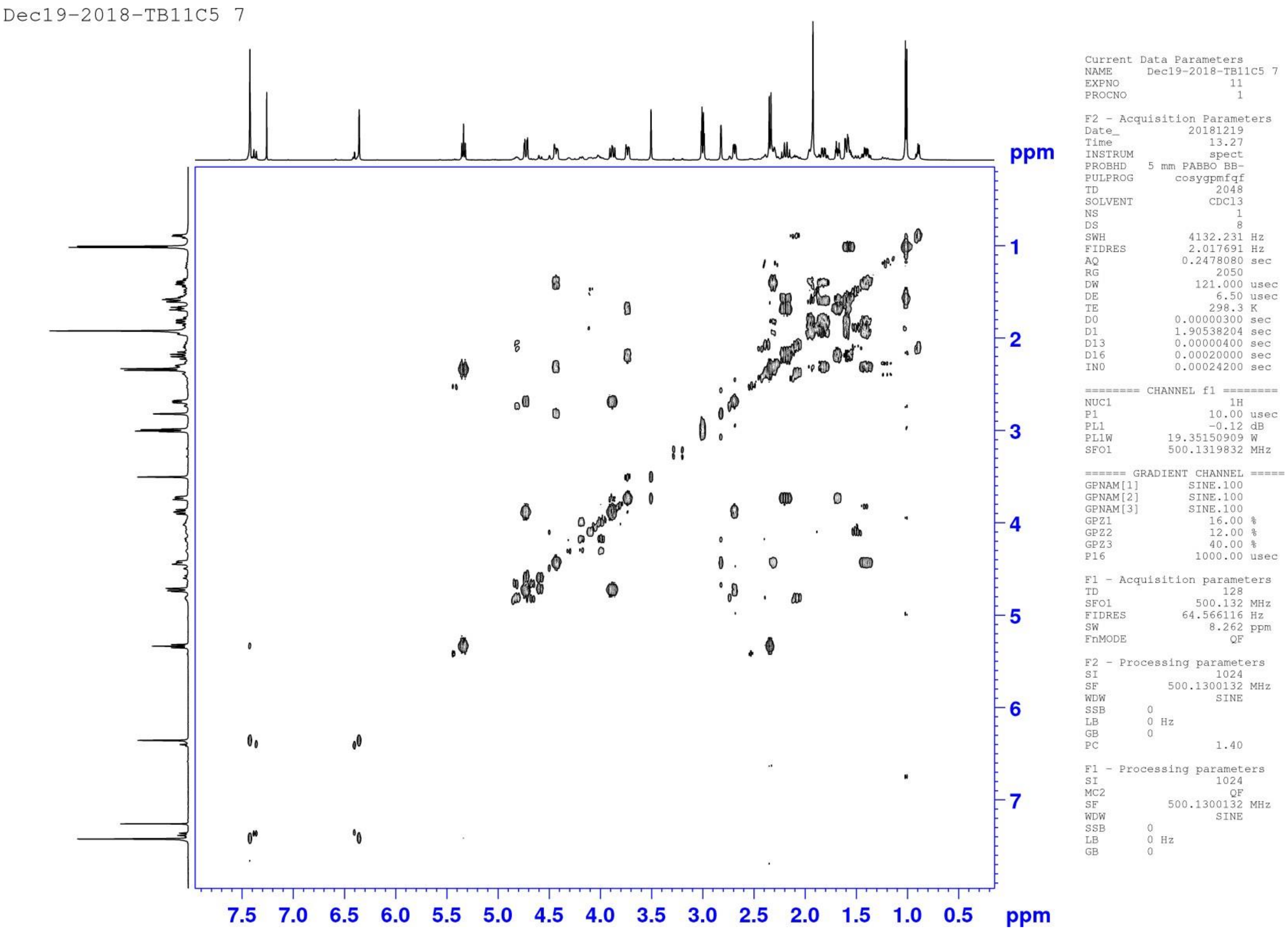

Figure S137. COSY NMR spectrum of **15** in $CDCl_3$.

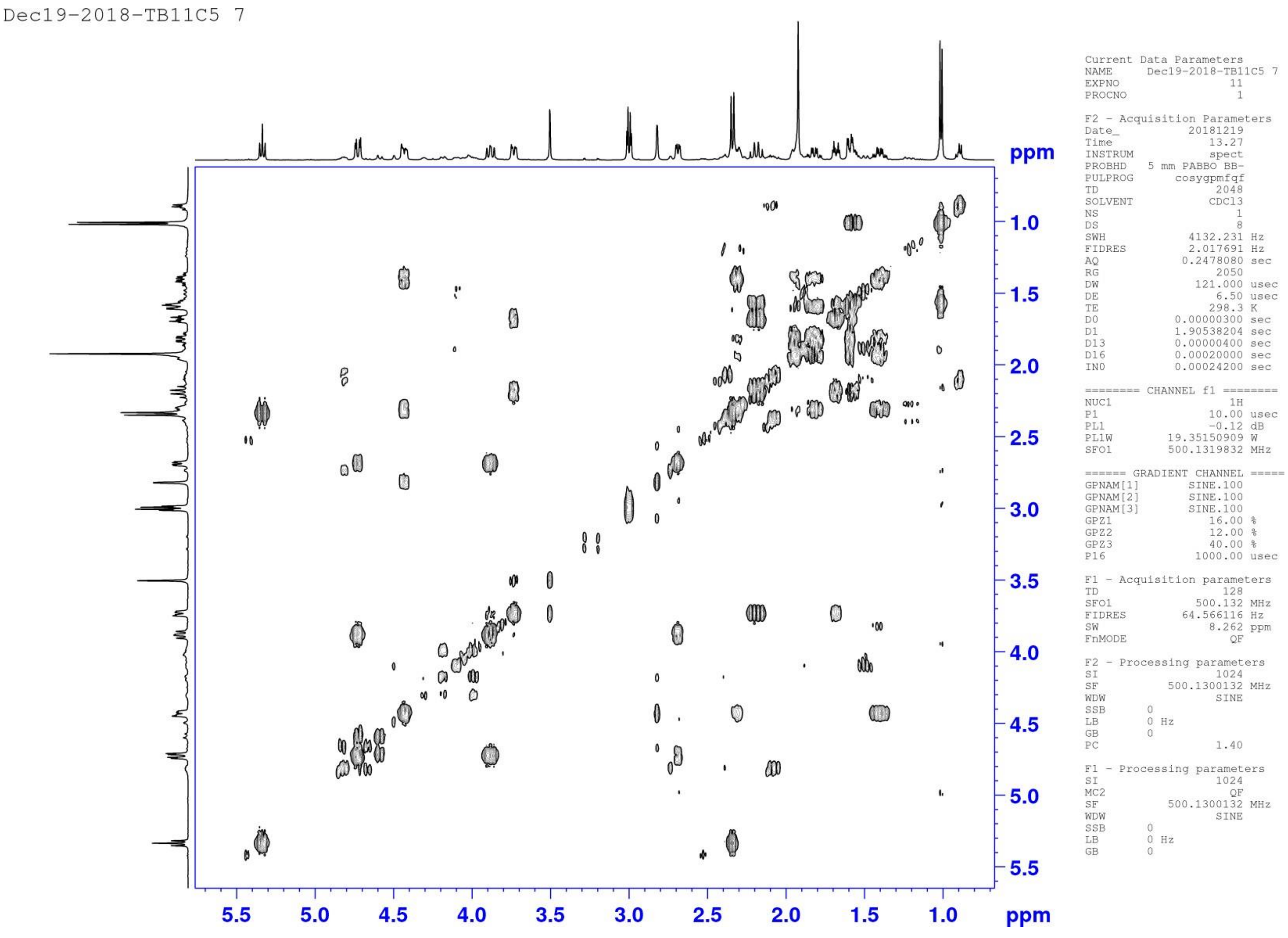

Figure S138. Expansion of part of COSY NMR spectrum of **15** in $CDCl_3$.

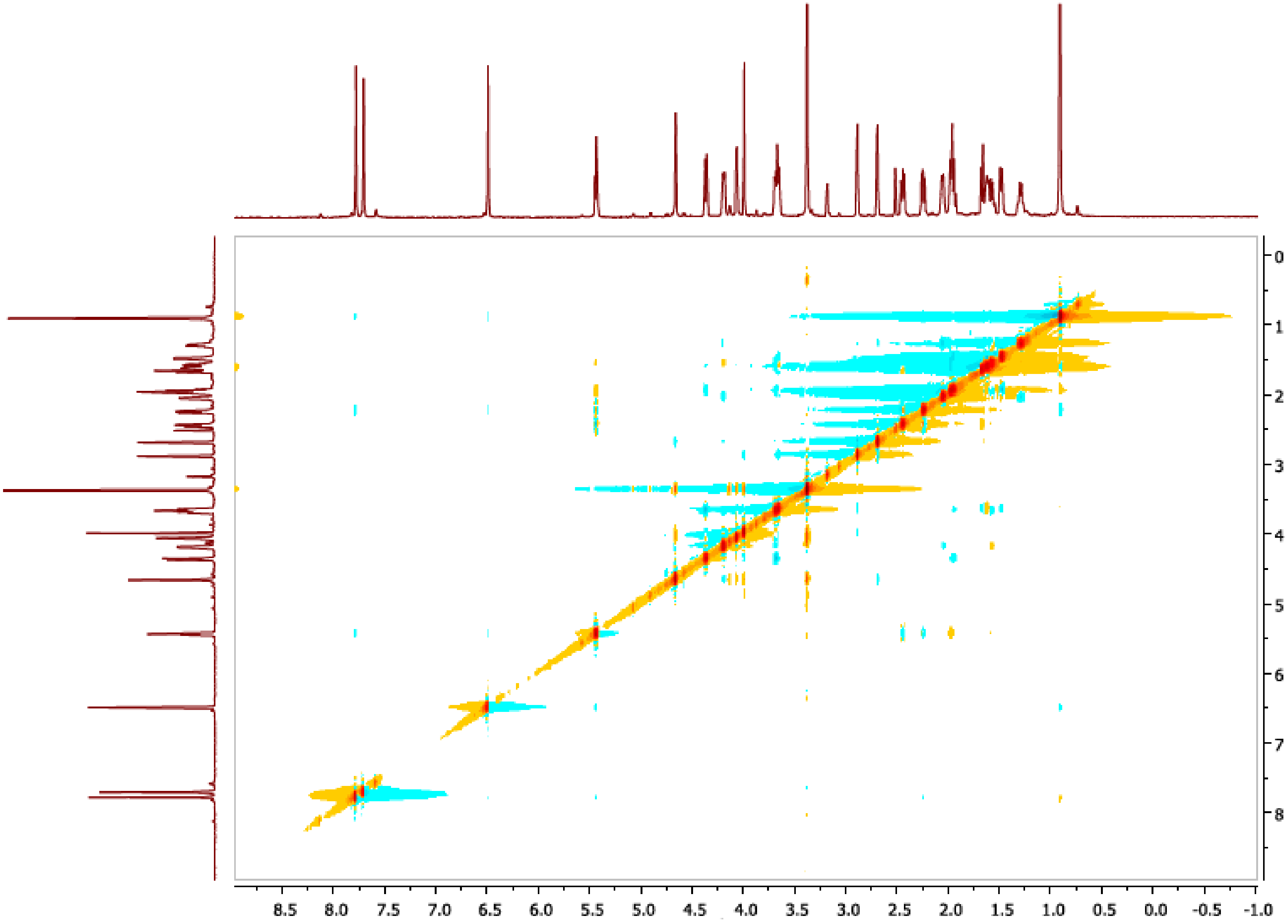

Figure S139. NOESY NMR spectrum of **15** in $(CD_3)_2SO$.

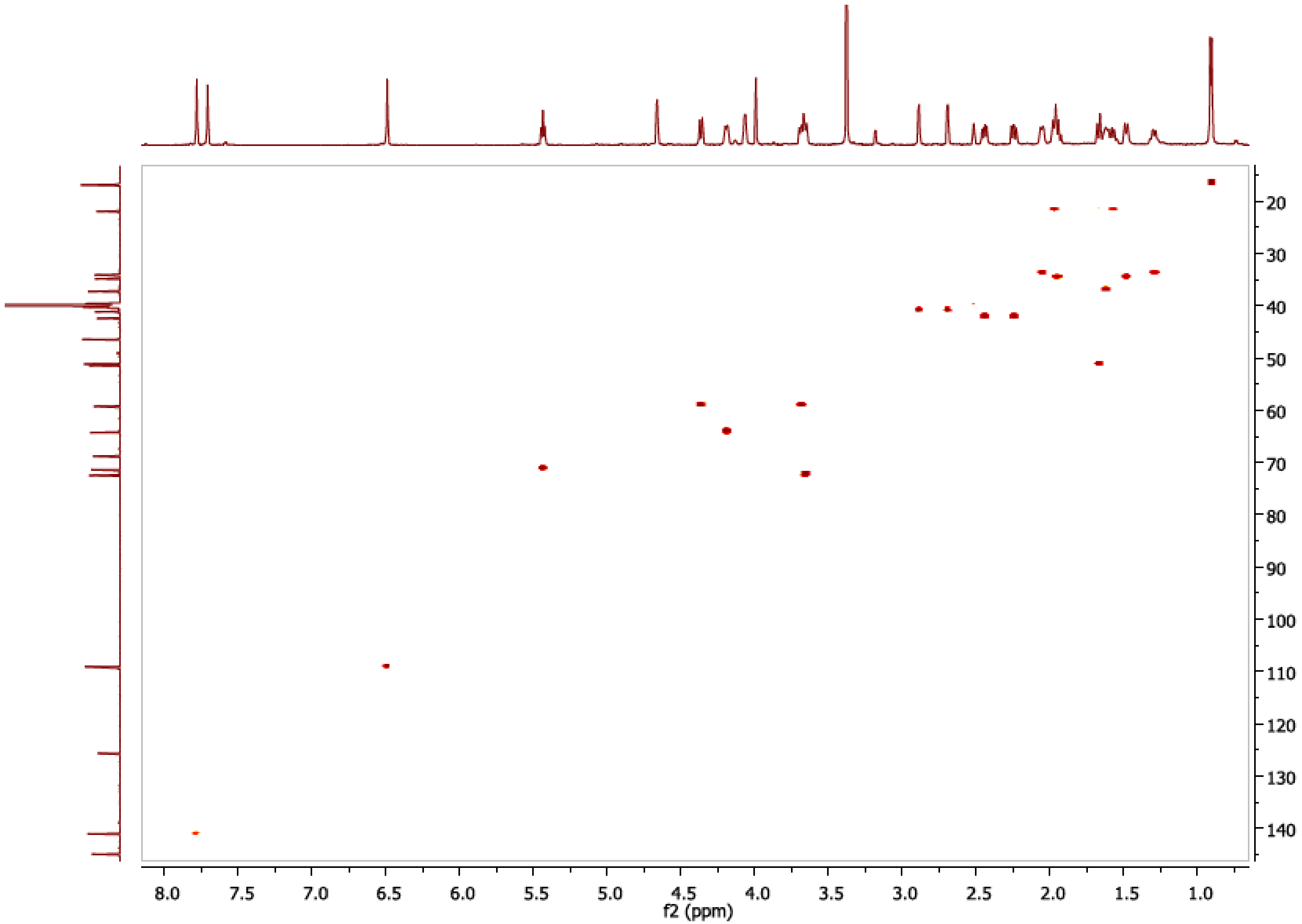

Figure S140. HSQC NMR spectrum of **15** in $(CD_3)_2SO$.

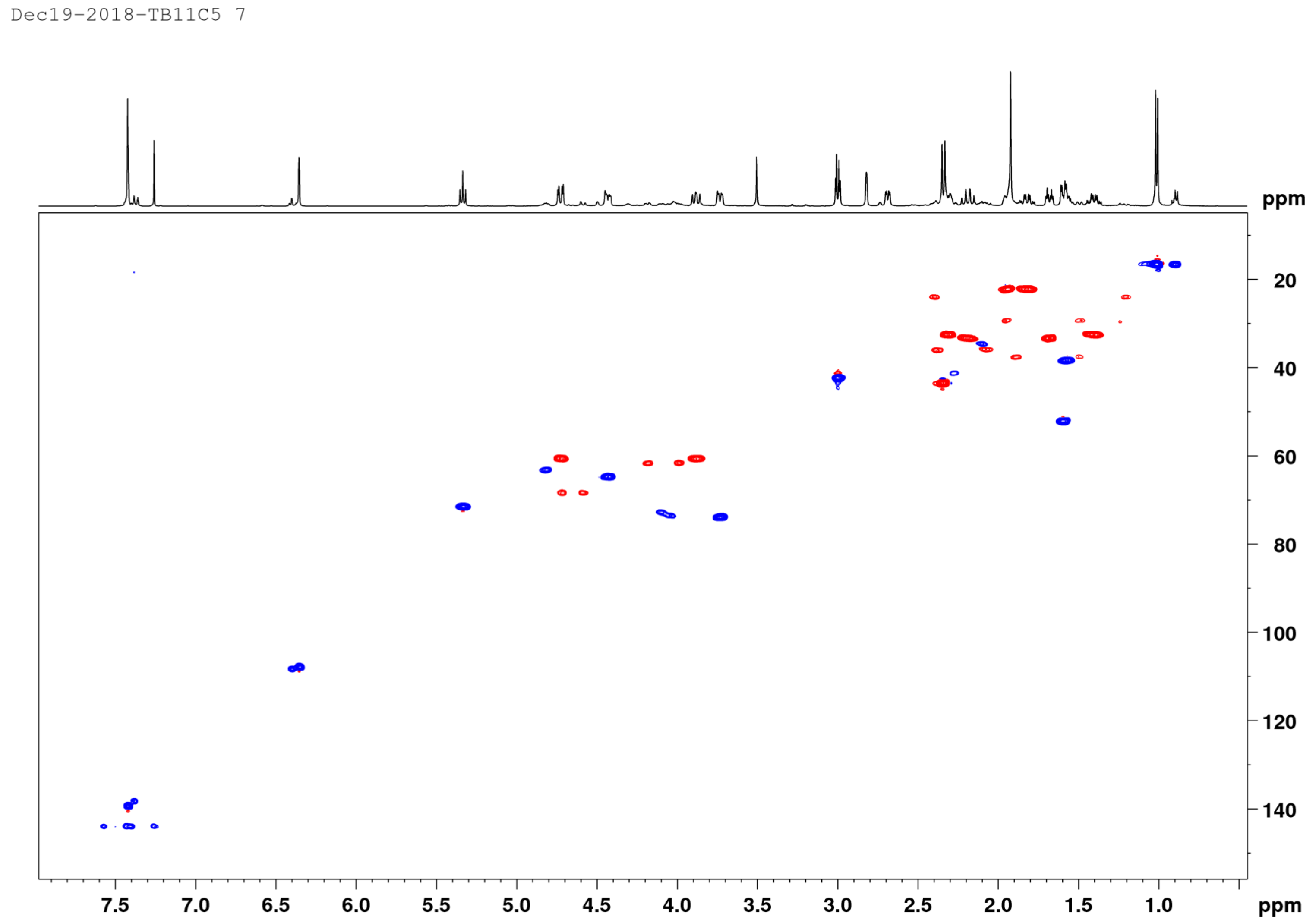

Figure S141. HSQC NMR spectrum of **15** in $CDCl_3$.

Dec19-2018-TB11C5 7

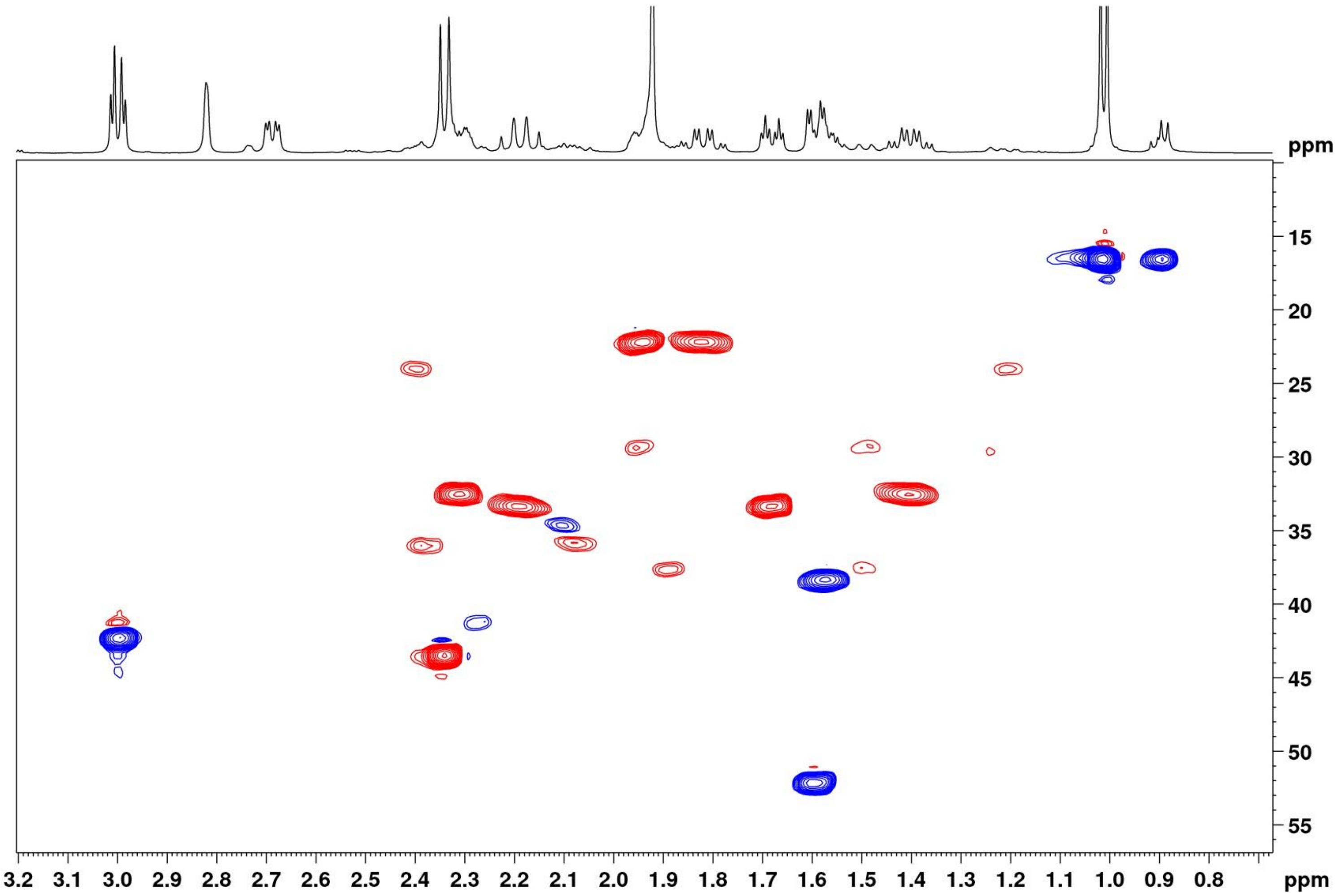

Figure S142. Expansion of part of HSQC NMR spectrum (δ 0.7 – δ 3.2) of **15** in $CDCl_3$.

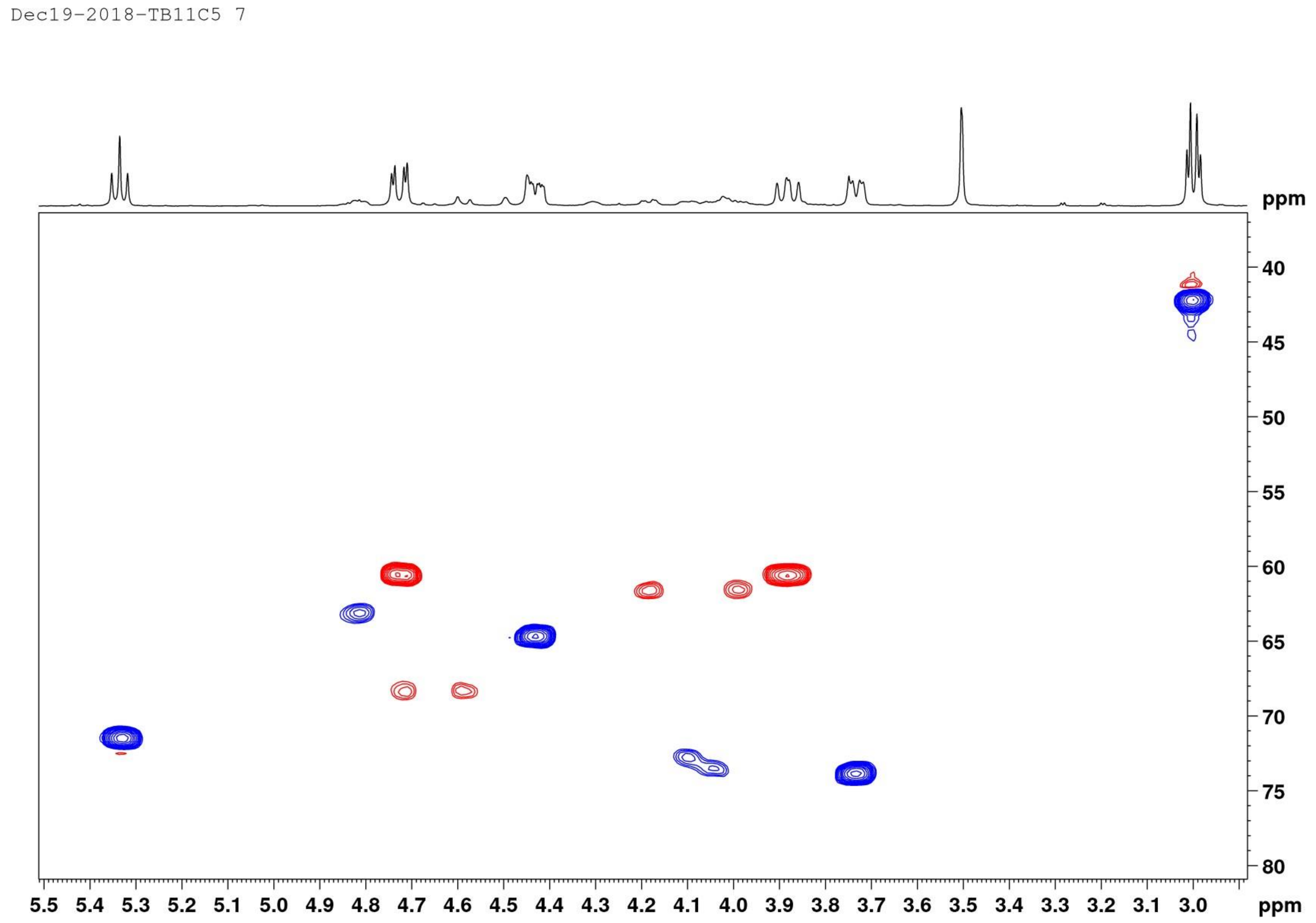

Figure S143. Expansion of part of HSQC NMR spectrum (δ 2.9 – δ 5.5) of **15** in $CDCl_3$.

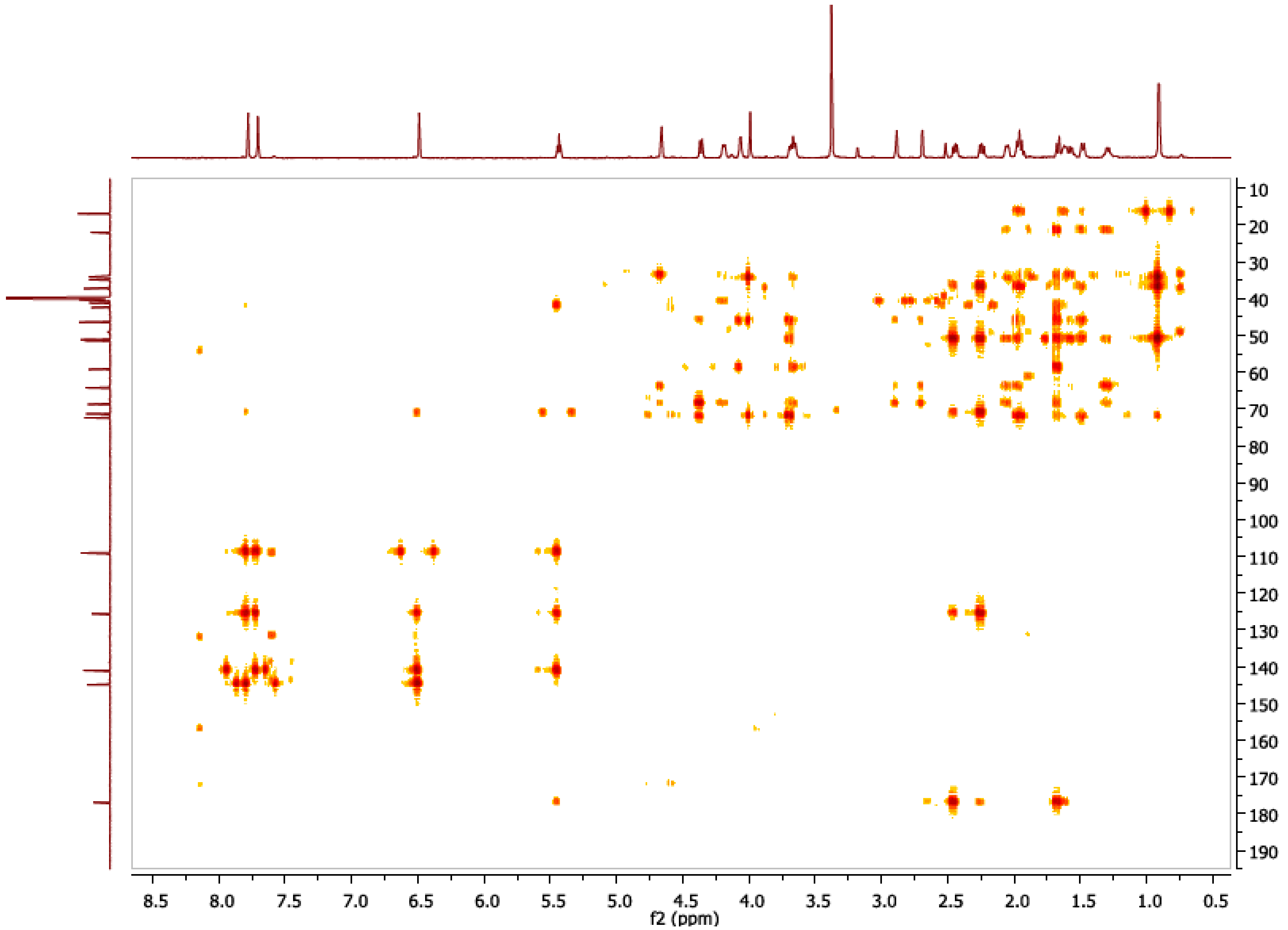

Figure S144. HMBC NMR spectrum of **15** in $(CD_3)_2SO$.

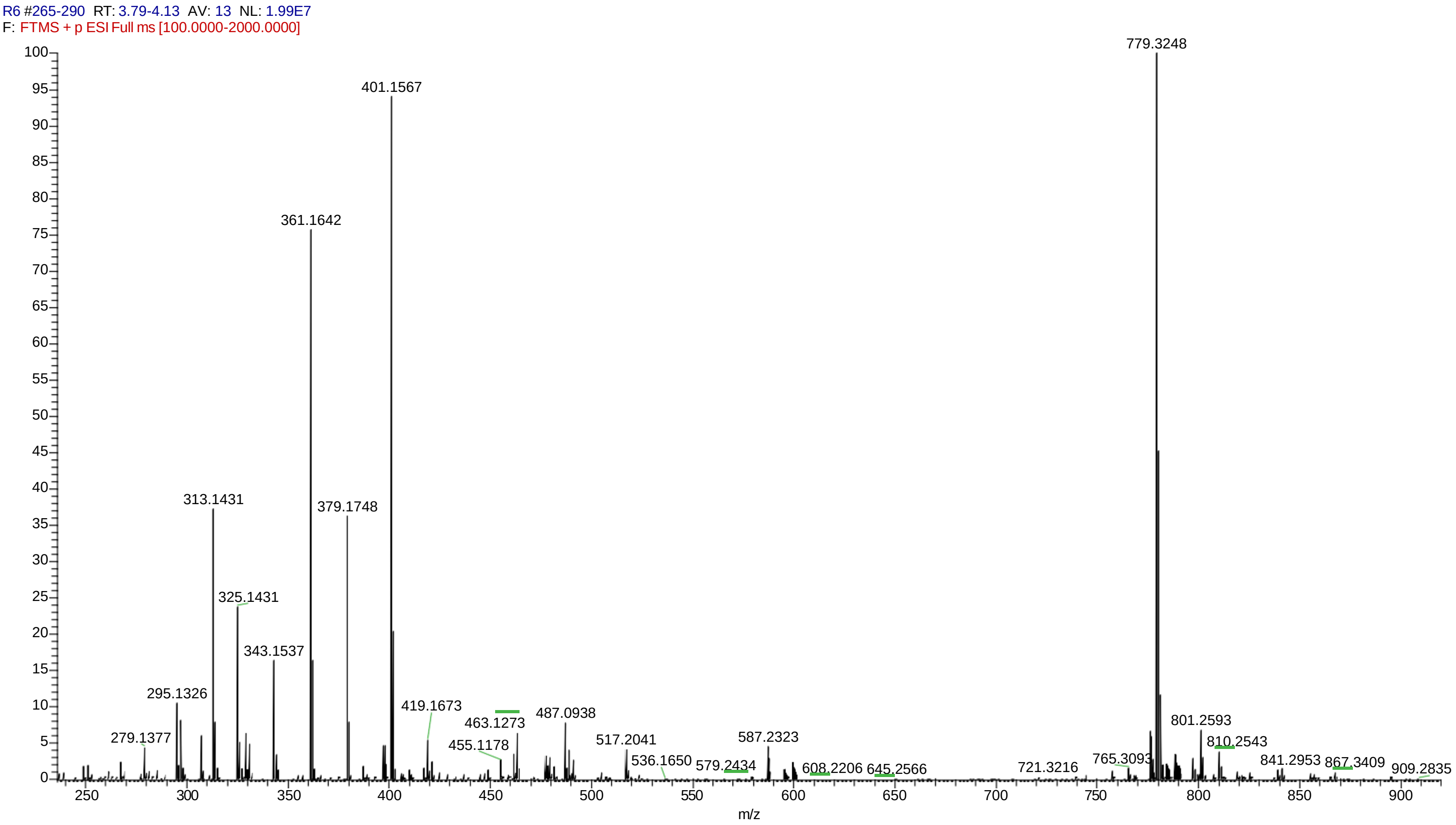

R6 #265-290 RT: 3.79-4.13 AV: 13 NL: 1.99E7
F: FTMS + p ESI Full ms [100.0000-2000.0000]
779.3248
401.1567
361.1642
313.1431
379.1748
325.1431
343.1537
295.1326
419.1673
463.1273
487.0938
455.1178
279.1377
517.2041
587.2323
536.1650
579.2434
608.2206
645.2566
721.3216
765.3093
801.2593
810.2543
841.2953
867.3409
909.2835
m/z

Figure S145. Positive-ion HRESIMS of **15**.

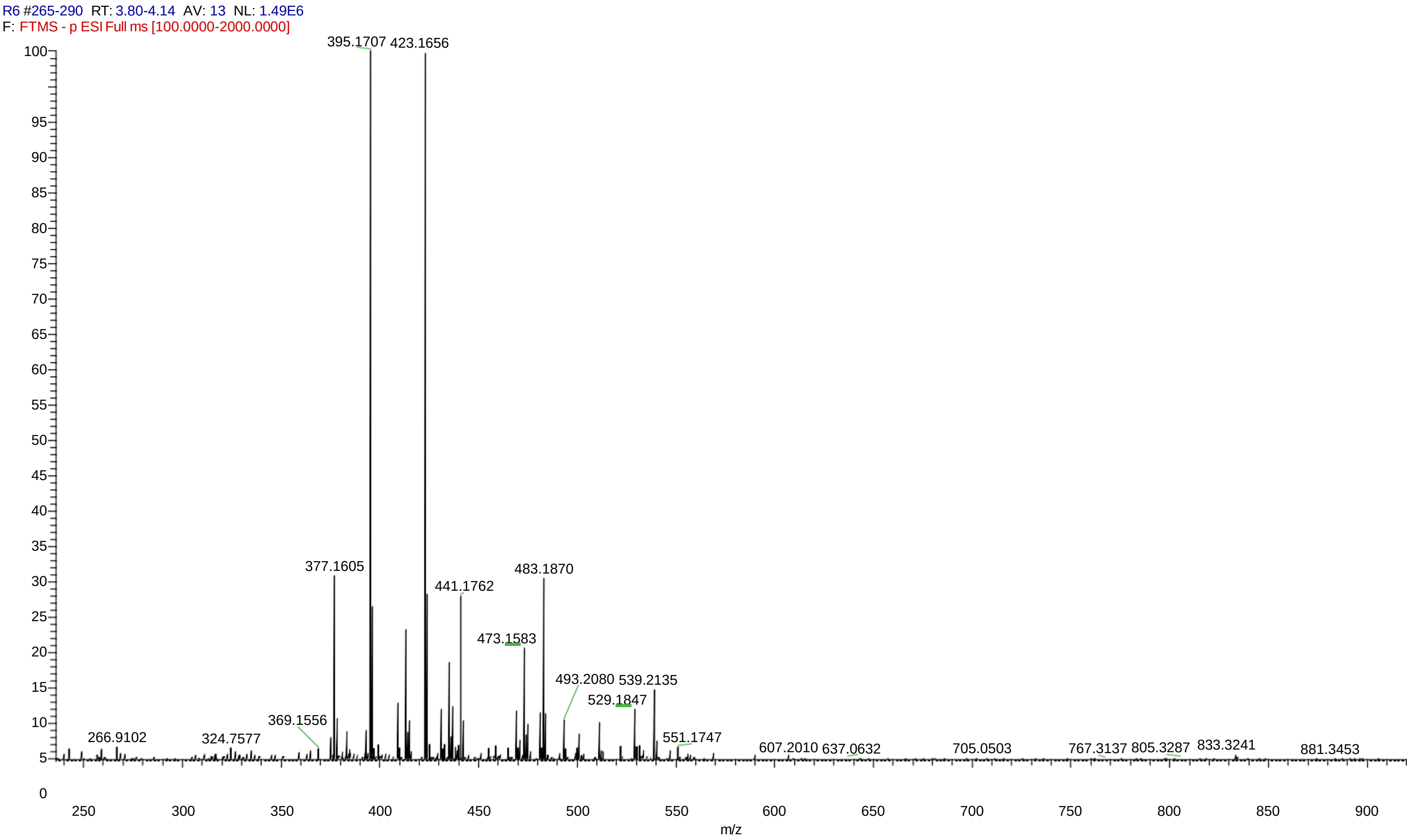
R6 #265-290 RT: 3.80-4.14 AV: 13 NL: 1.49E6
F: FTMS - p ESI Full ms [100.0000-2000.0000]
395.1707
423.1656
377.1605
441.1762
483.1870
473.1583
493.2080
539.2135
529.1847
369.1556
266.9102
324.7577
551.1747
607.2010
637.0632
705.0503
767.3137
805.3287
833.3241
881.3453
m/z

Figure S146. Negative-ion HRESIMS of **15**.

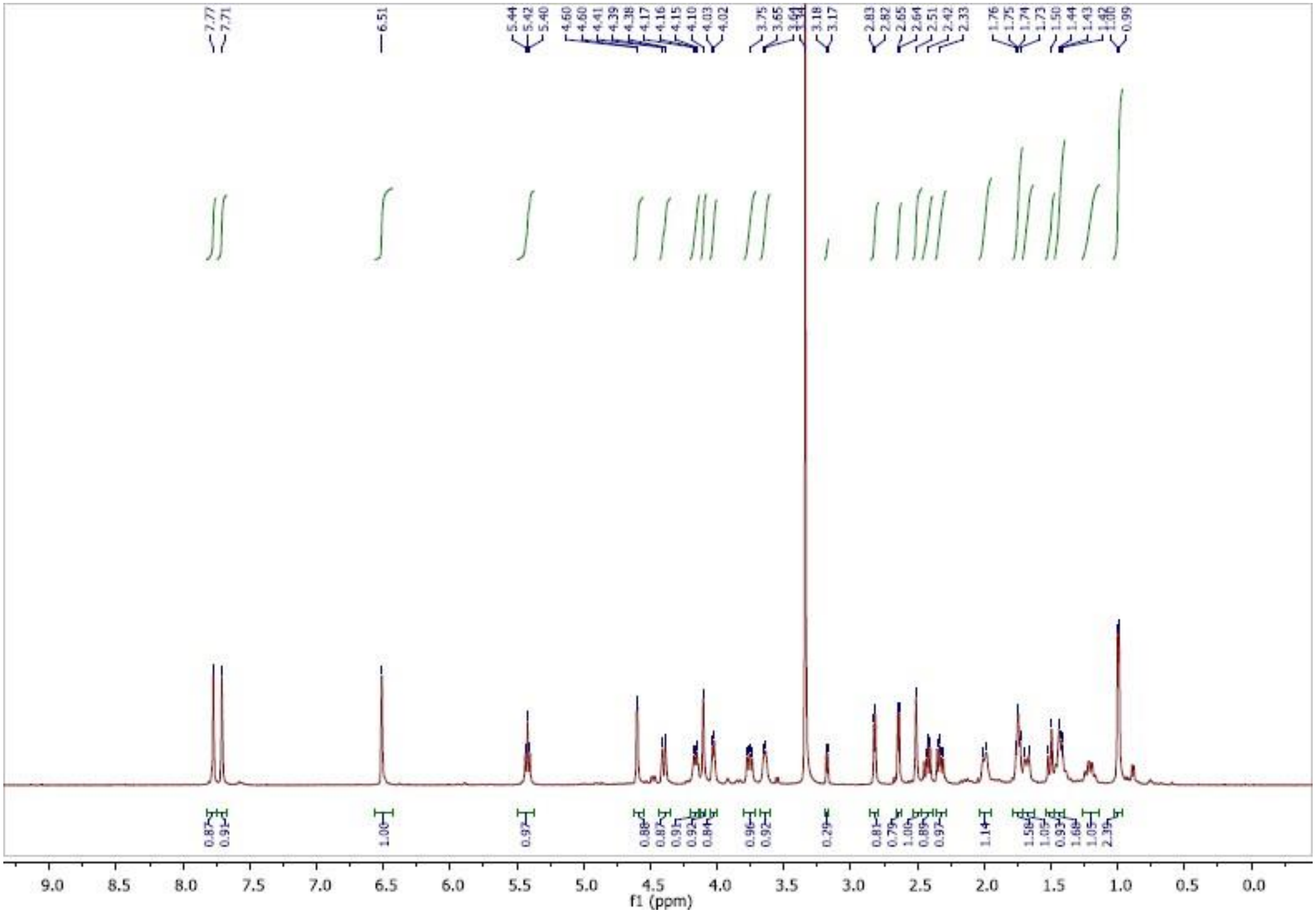

Figure S147. ^{1}H NMR spectrum of **16** in $(CD_3)_2SO$.

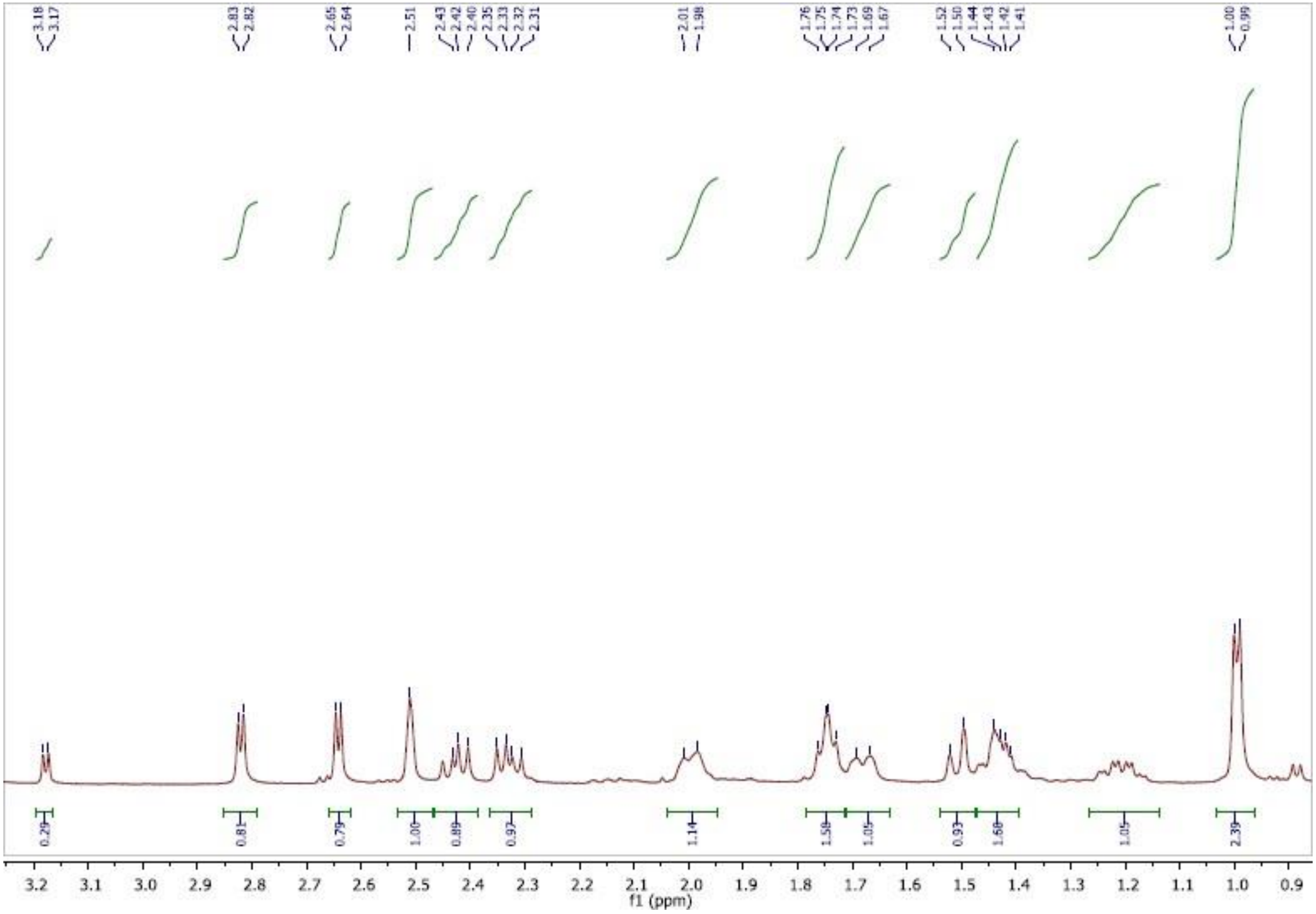

Figure S148. Expansion of part of ^{1}H NMR spectrum (δ 0.9 – δ 3.2) of **16** in $(CD_3)_2SO$.

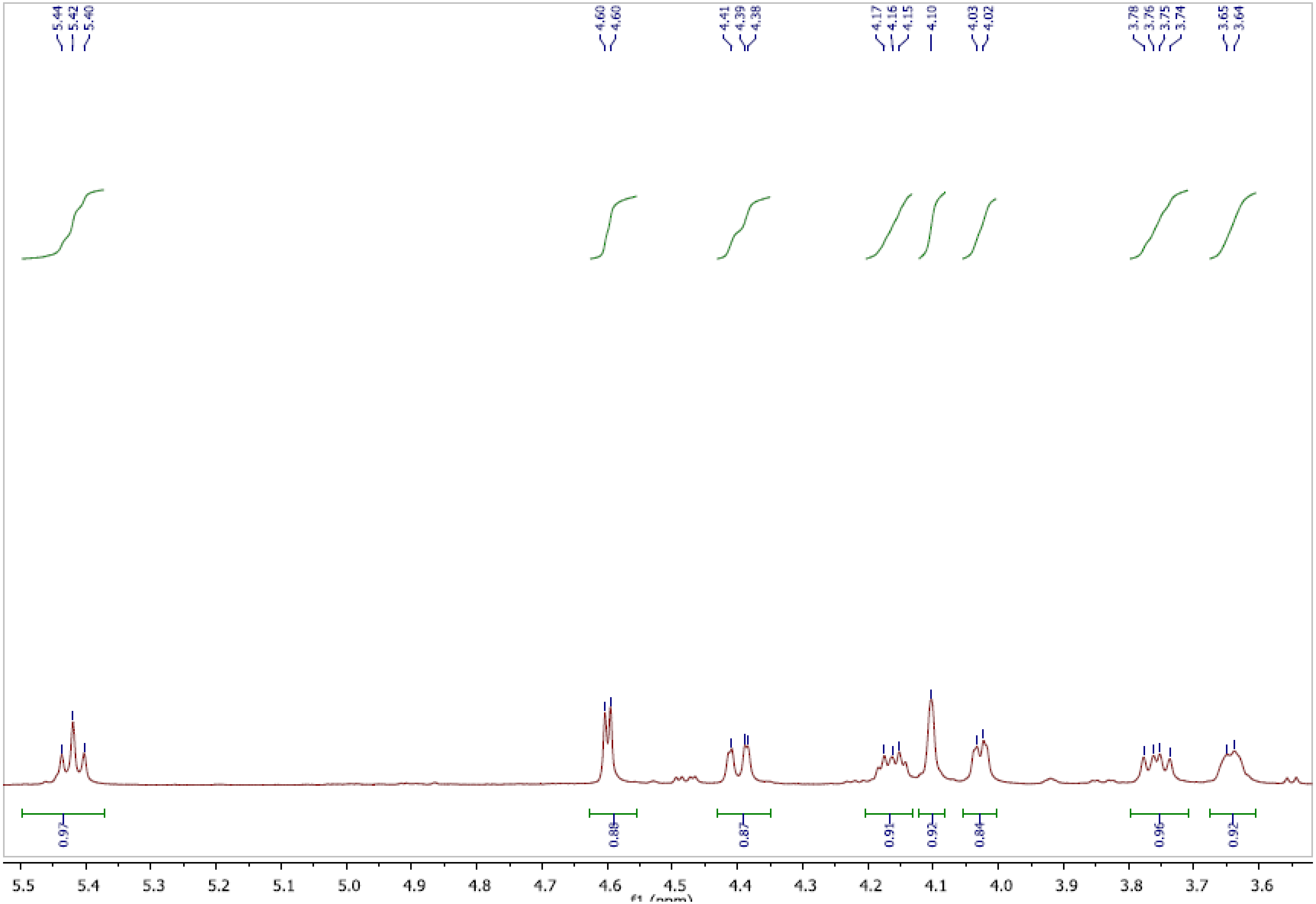

Figure S149. Expansion of part of ^{1}H NMR spectrum (δ 3.6 – δ 5.5) of **16** in $(CD_3)_2SO$.

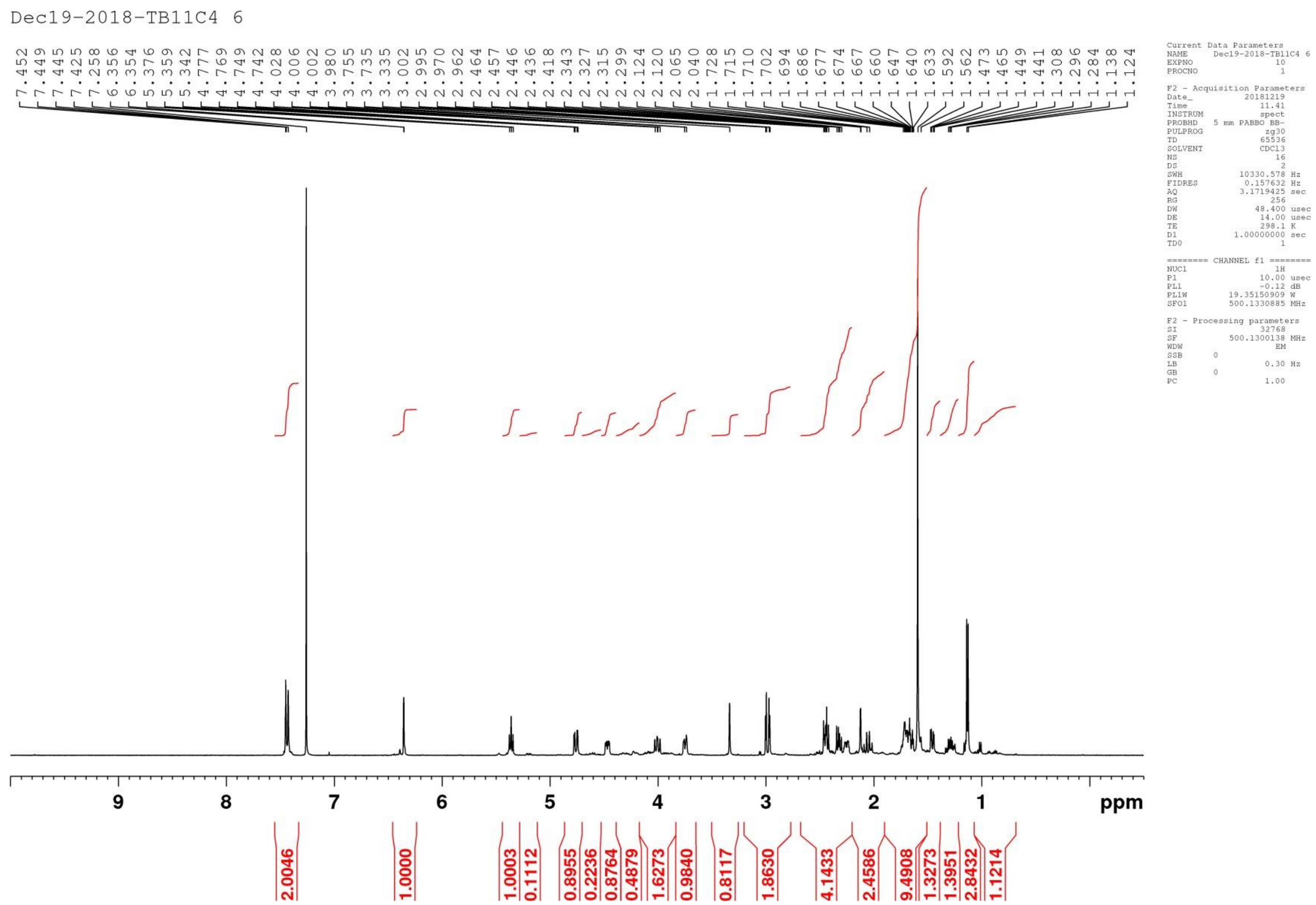

Figure S150. ^{1}H NMR spectrum of **16** in $CDCl_3$.

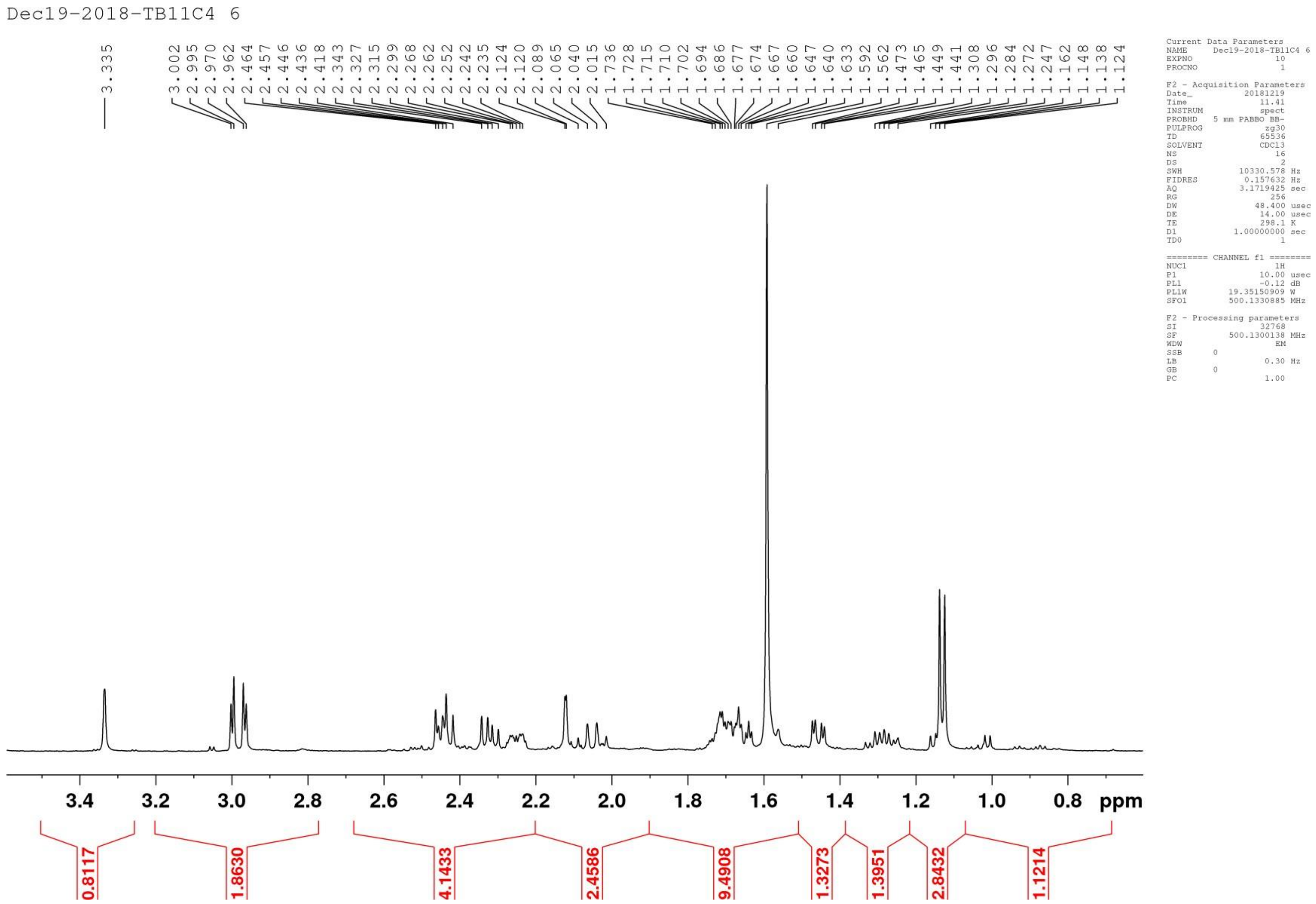

Figure S151. Expansion of part of ^{1}H NMR spectrum (δ 0.7 – δ 3.5) of **16** in $CDCl_3$.

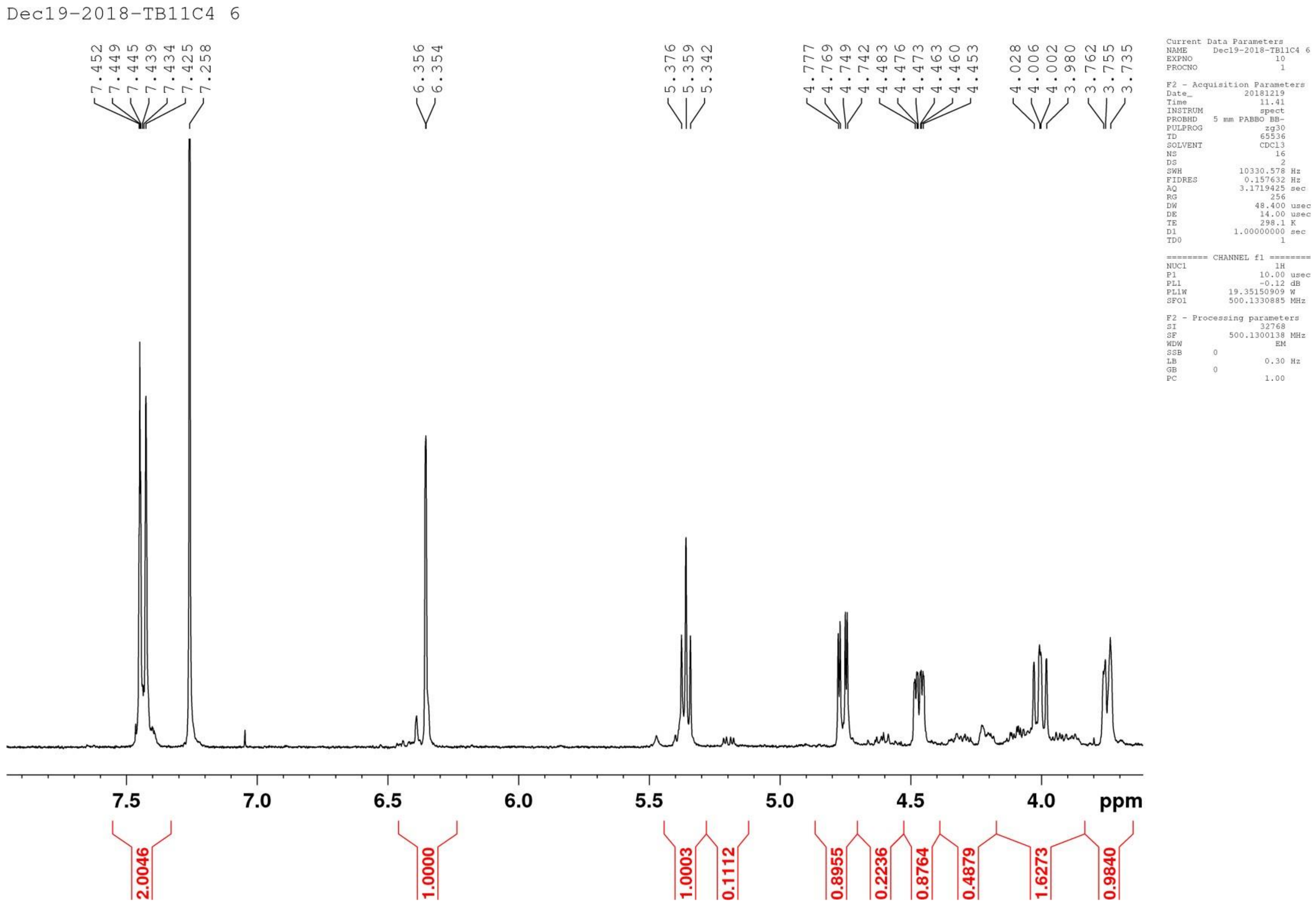

Figure S157. Expansion of part of ^{1}H NMR spectrum (δ 3.7 – δ 7.9) of **16** in $CDCl_3$.

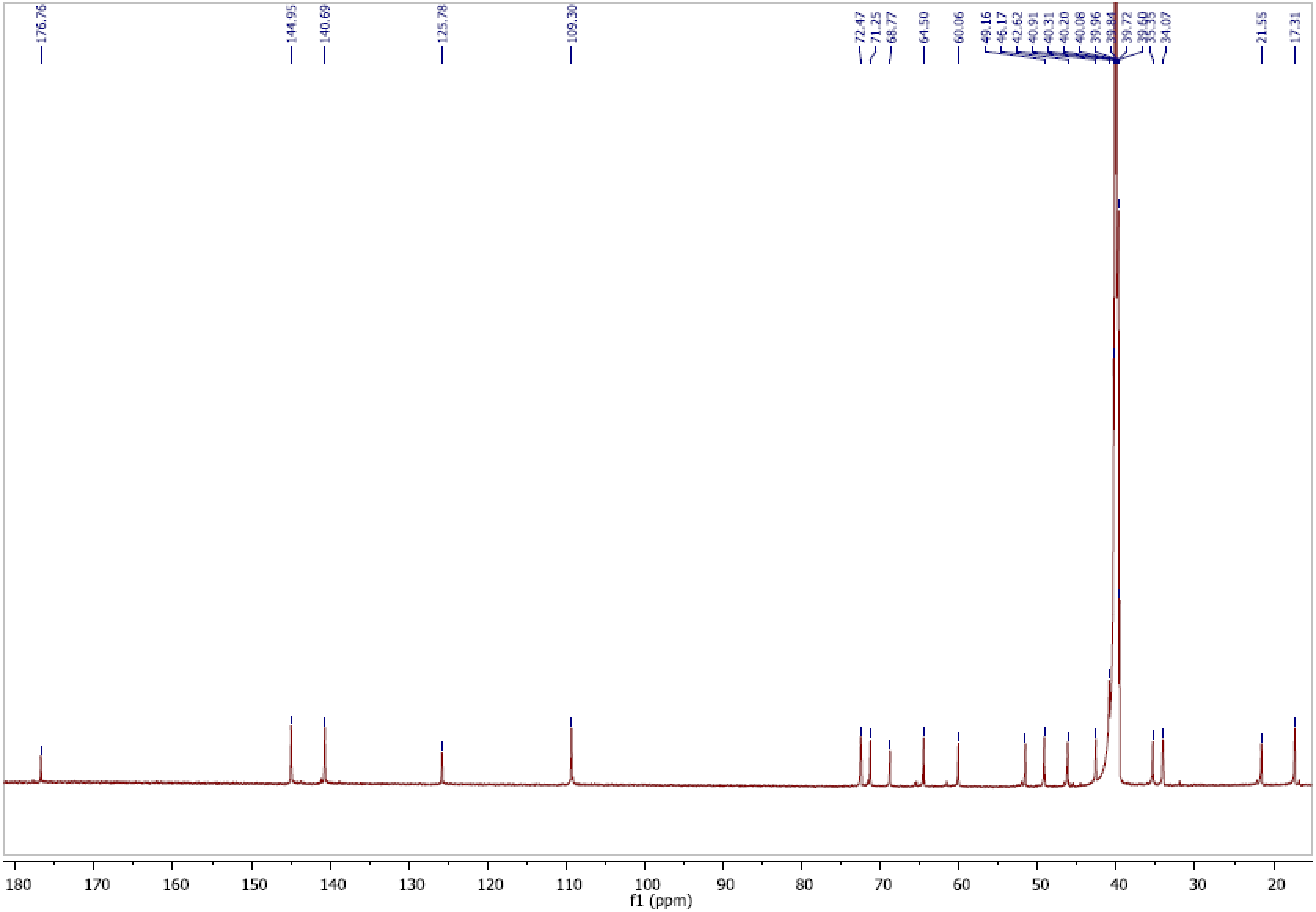

Figure S153. ^{13}C NMR spectrum of **16** in $(CD_3)_2SO$.

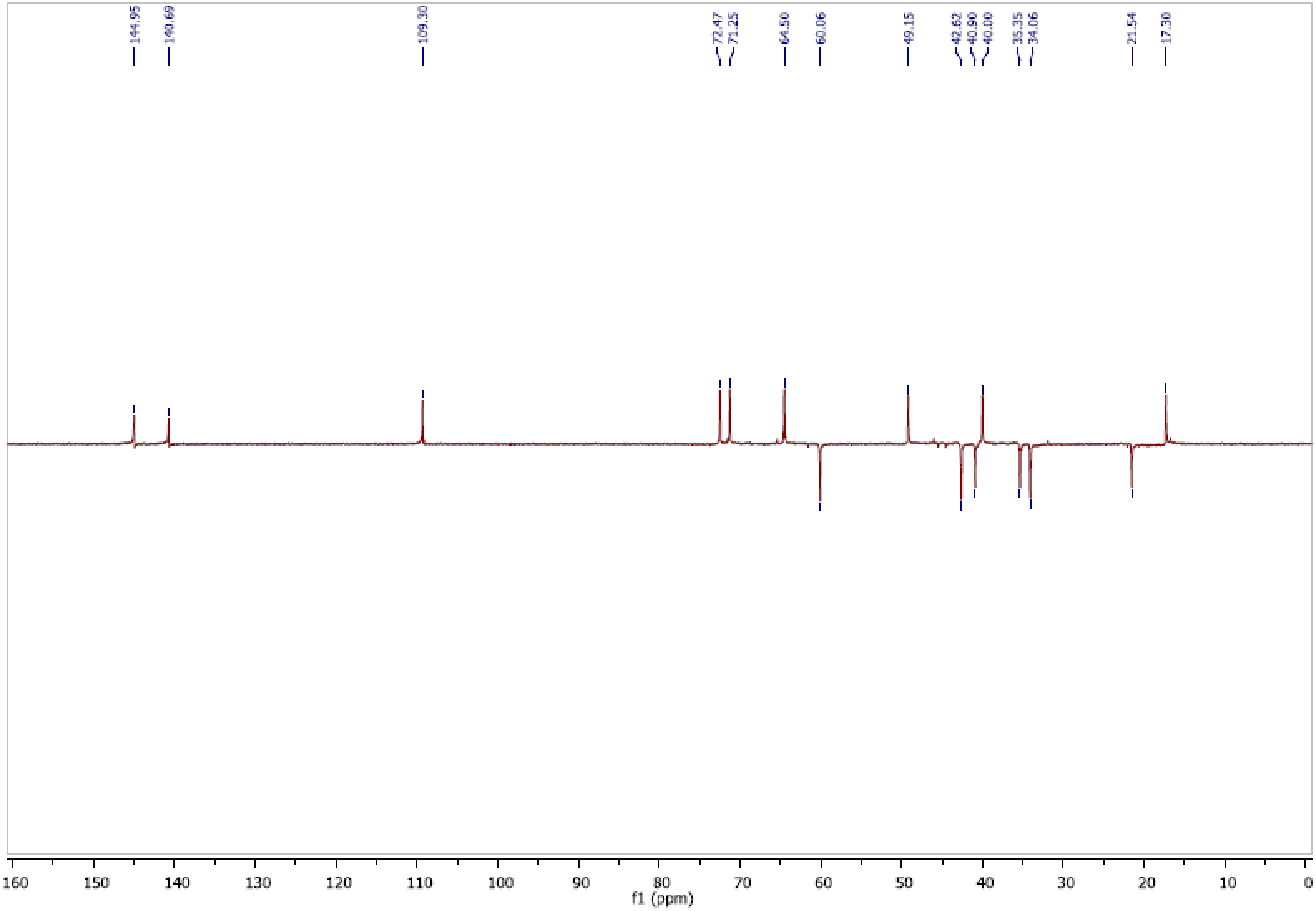

Figure S154. 135DEPT NMR spectrum of **16** in $(CD_3)_2SO$.

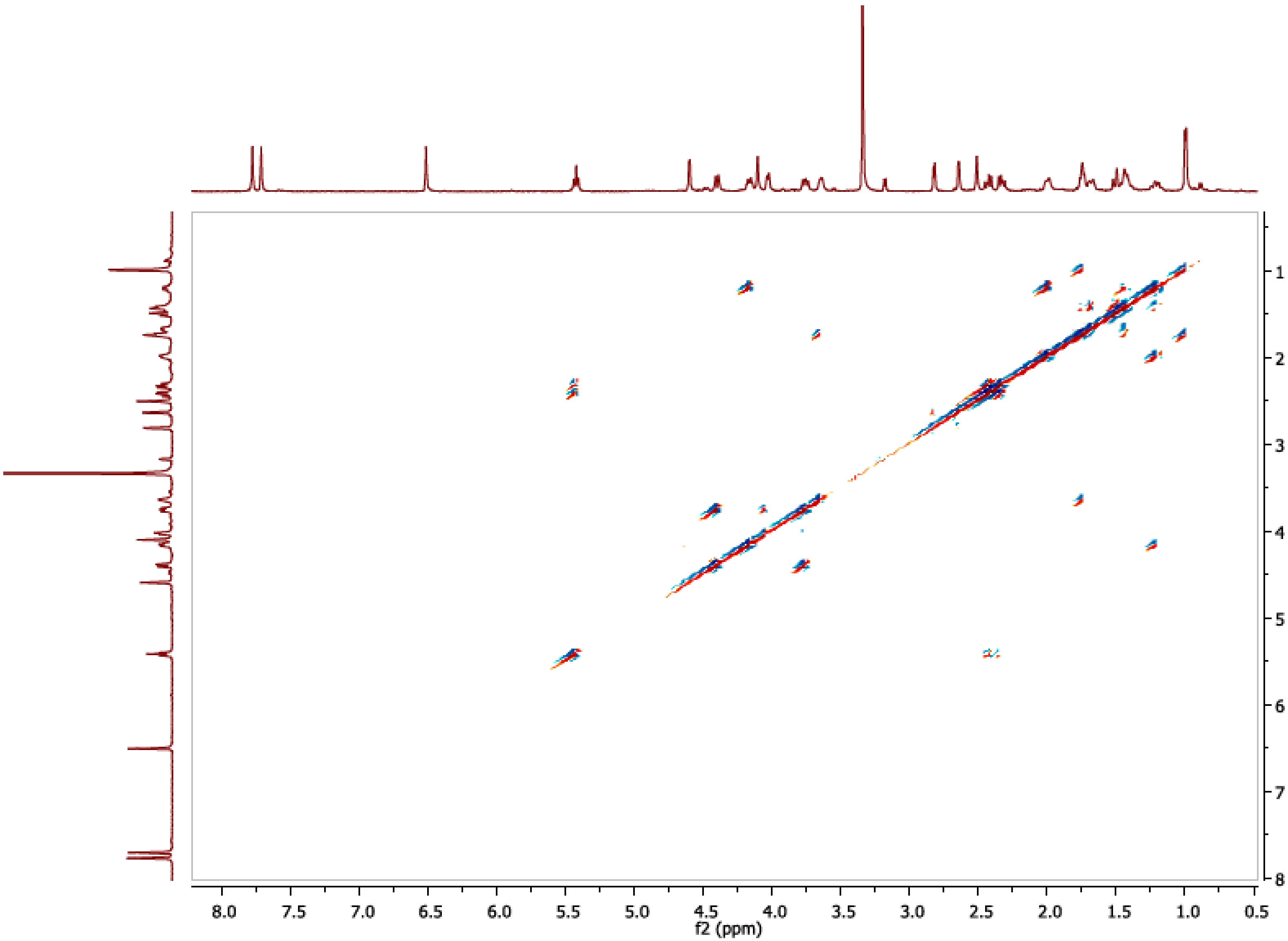

Figure S155. COSY NMR spectrum of **16** in $(CD_3)_2SO$.

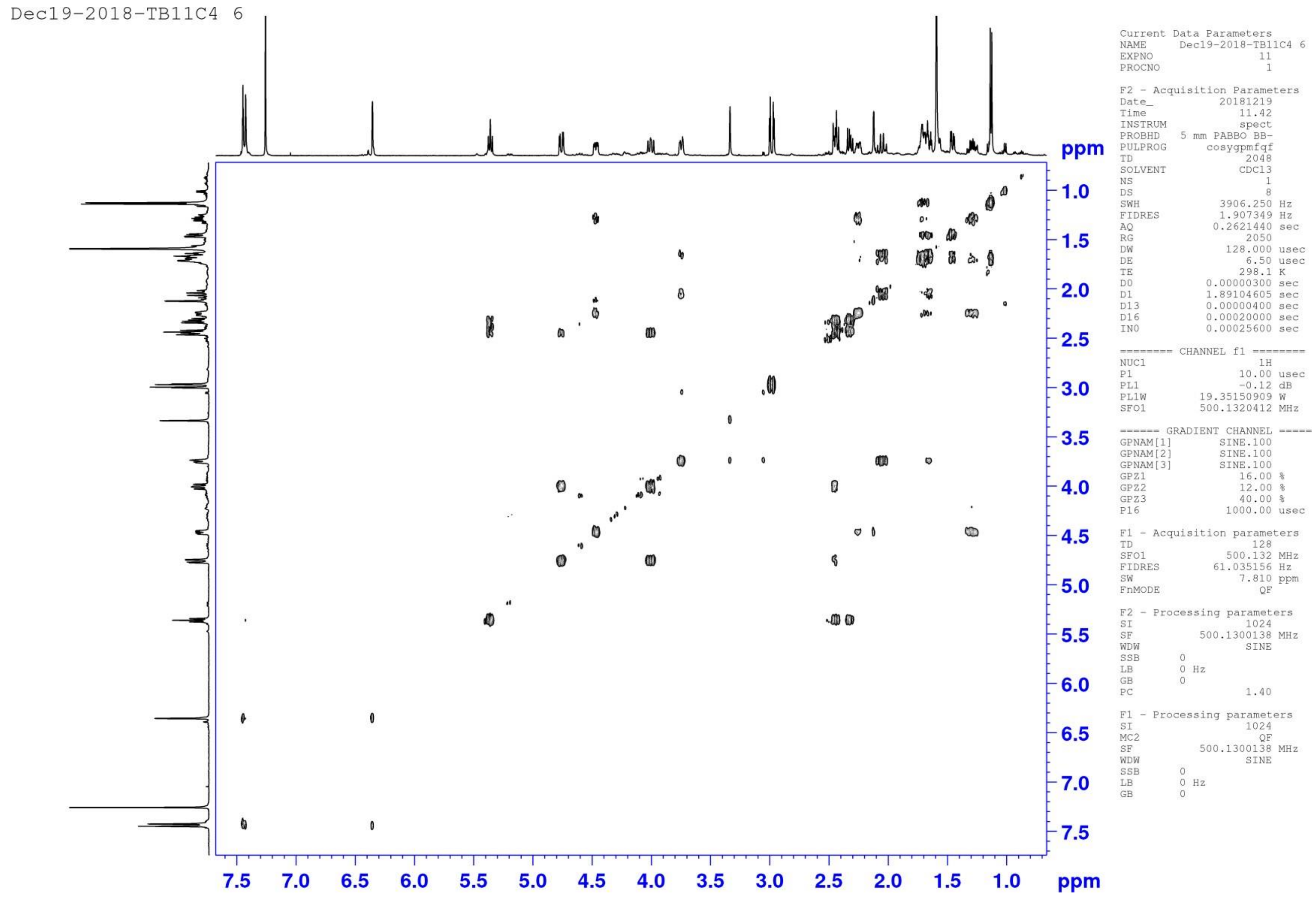

Figure S156. COSY NMR spectrum of **16** in $CDCl_3$.

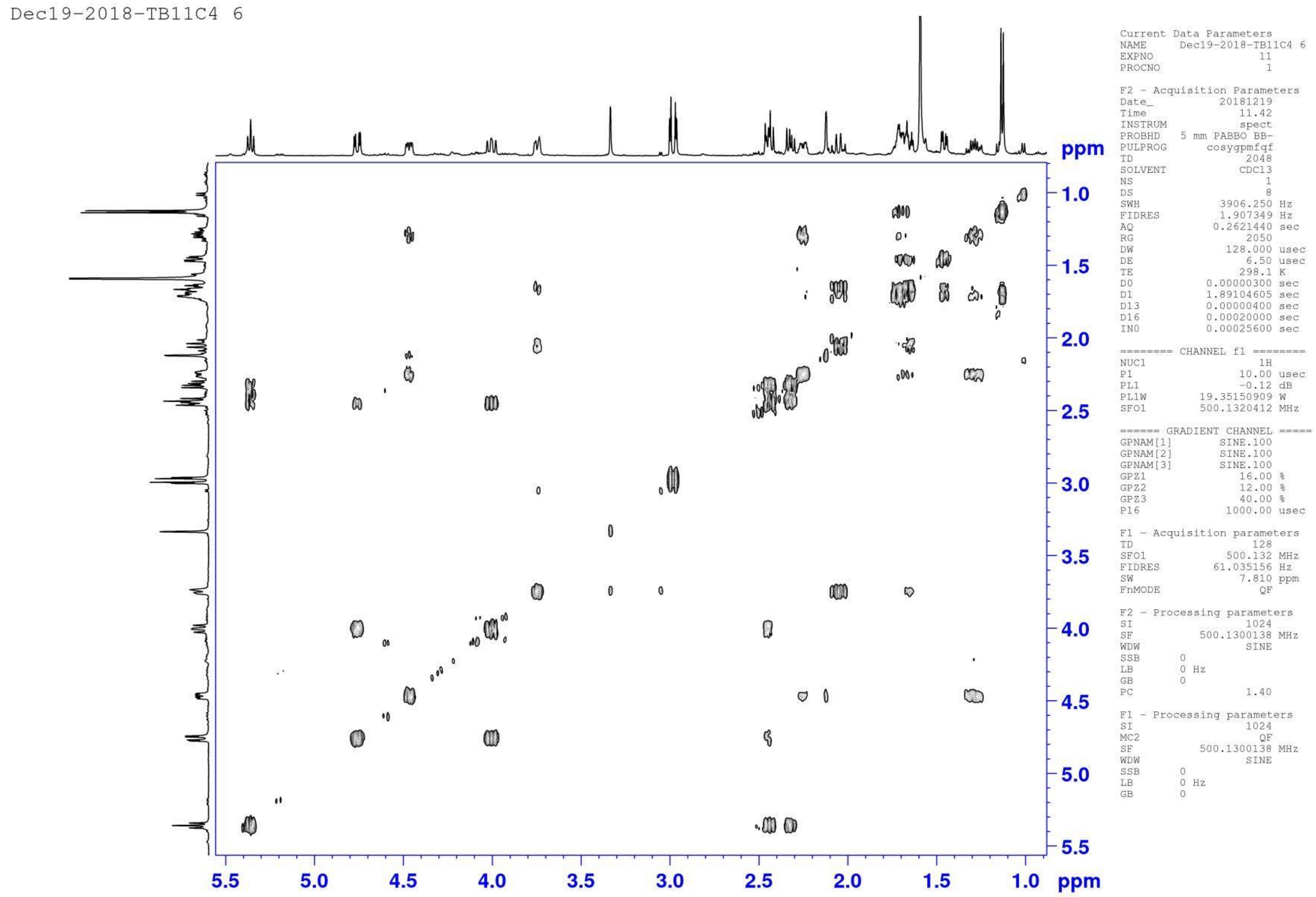

Figure S157. Expansion of part of COSY NMR spectrum (δ 0.9 – δ 5.5) of **16** in $CDCl_3$.

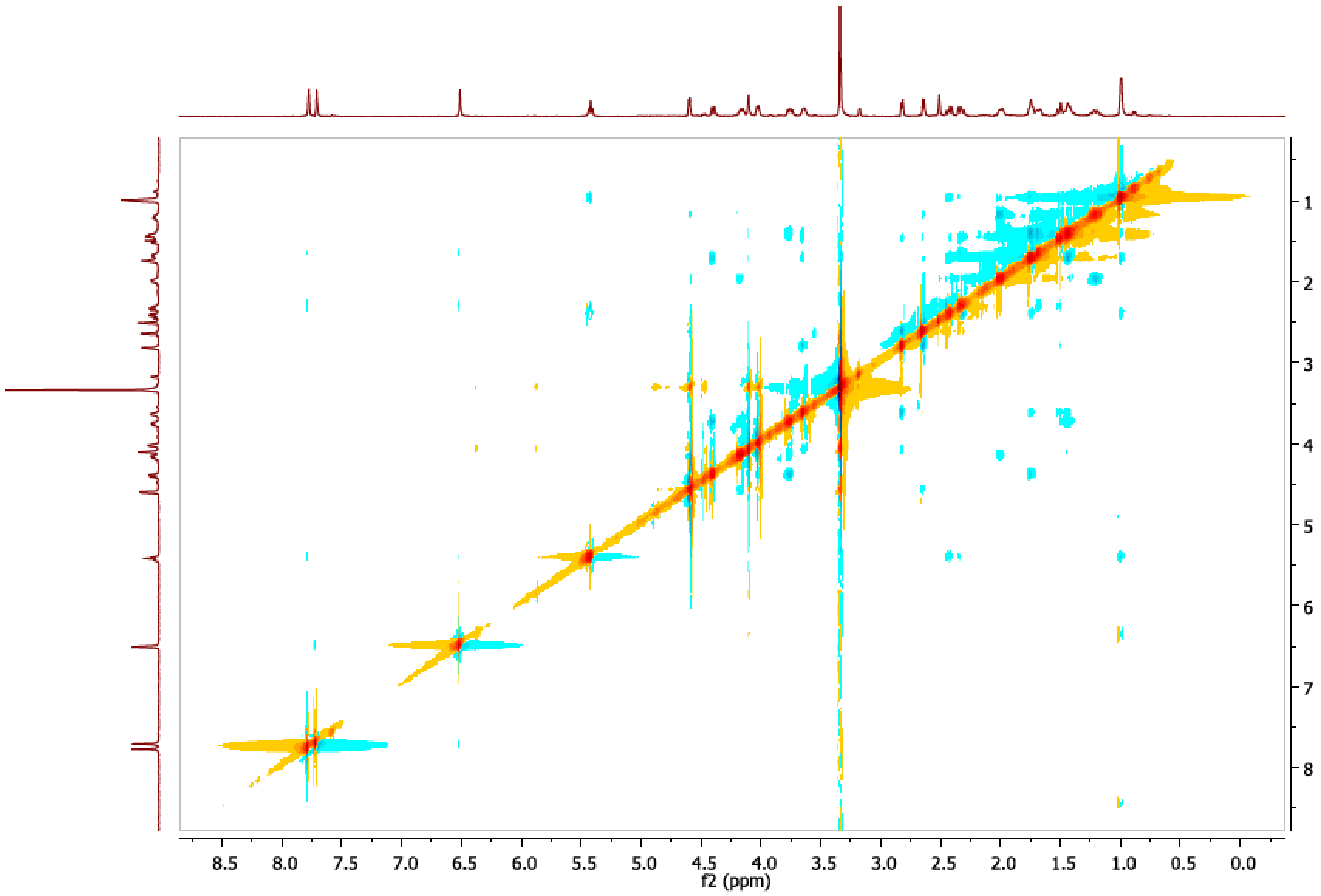

Figure S158. NOESY NMR spectrum of **16** in $(CD_3)_2SO$.

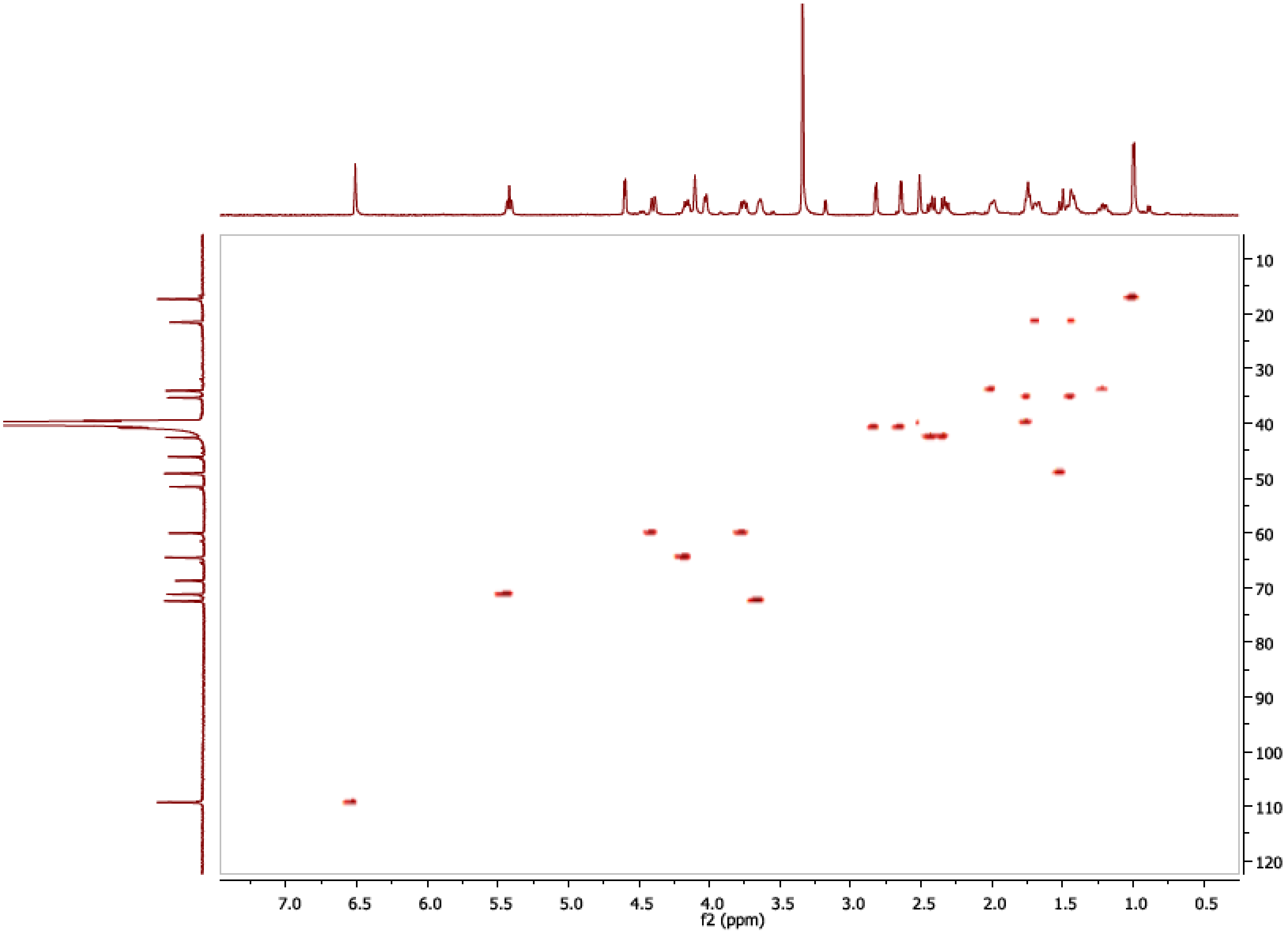

Figure S159. HSQC NMR spectrum of **16** in $(CD_3)_2SO$.

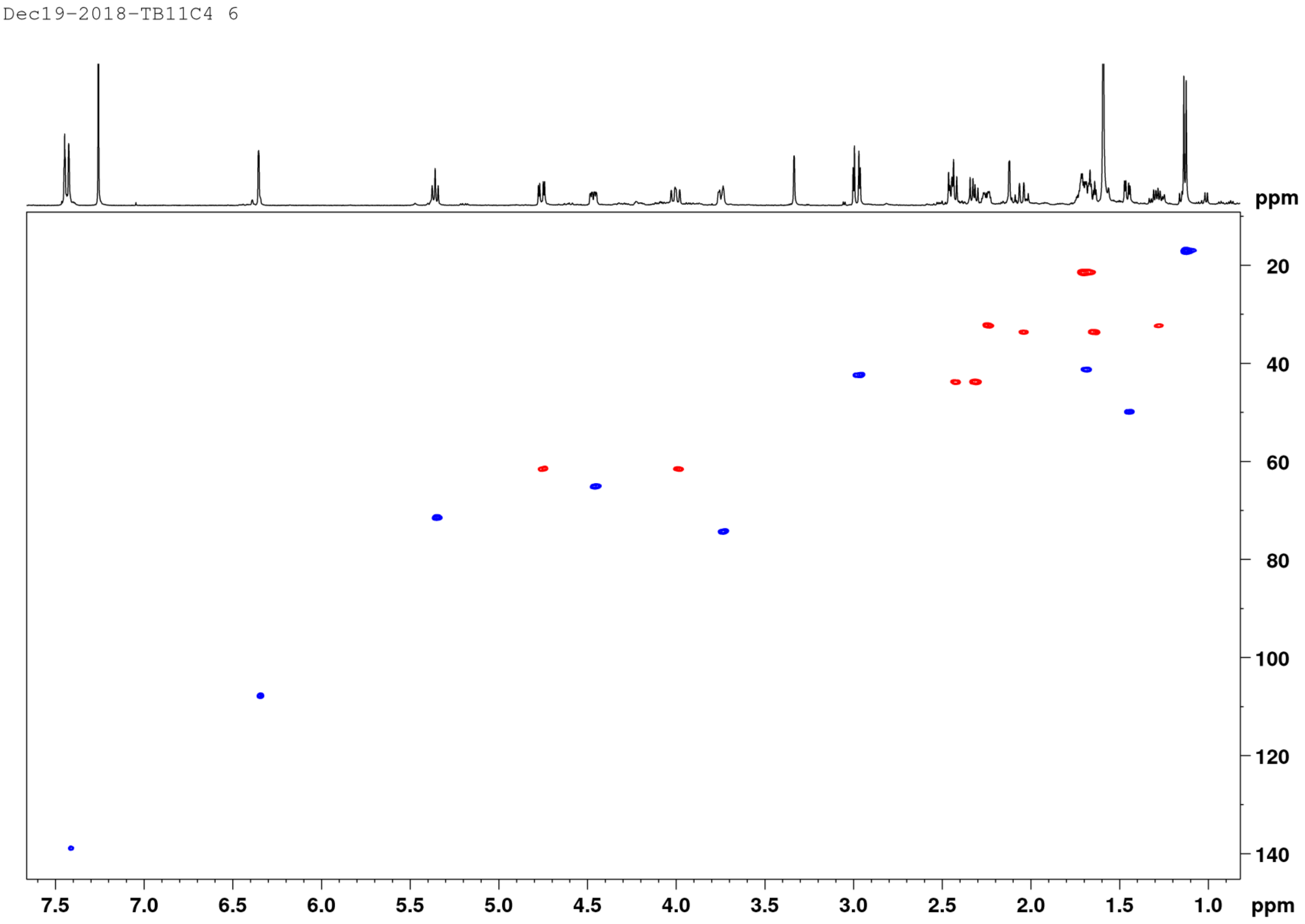

Figure S160. HSQC NMR spectrum of **16** in $CDCl_3$.

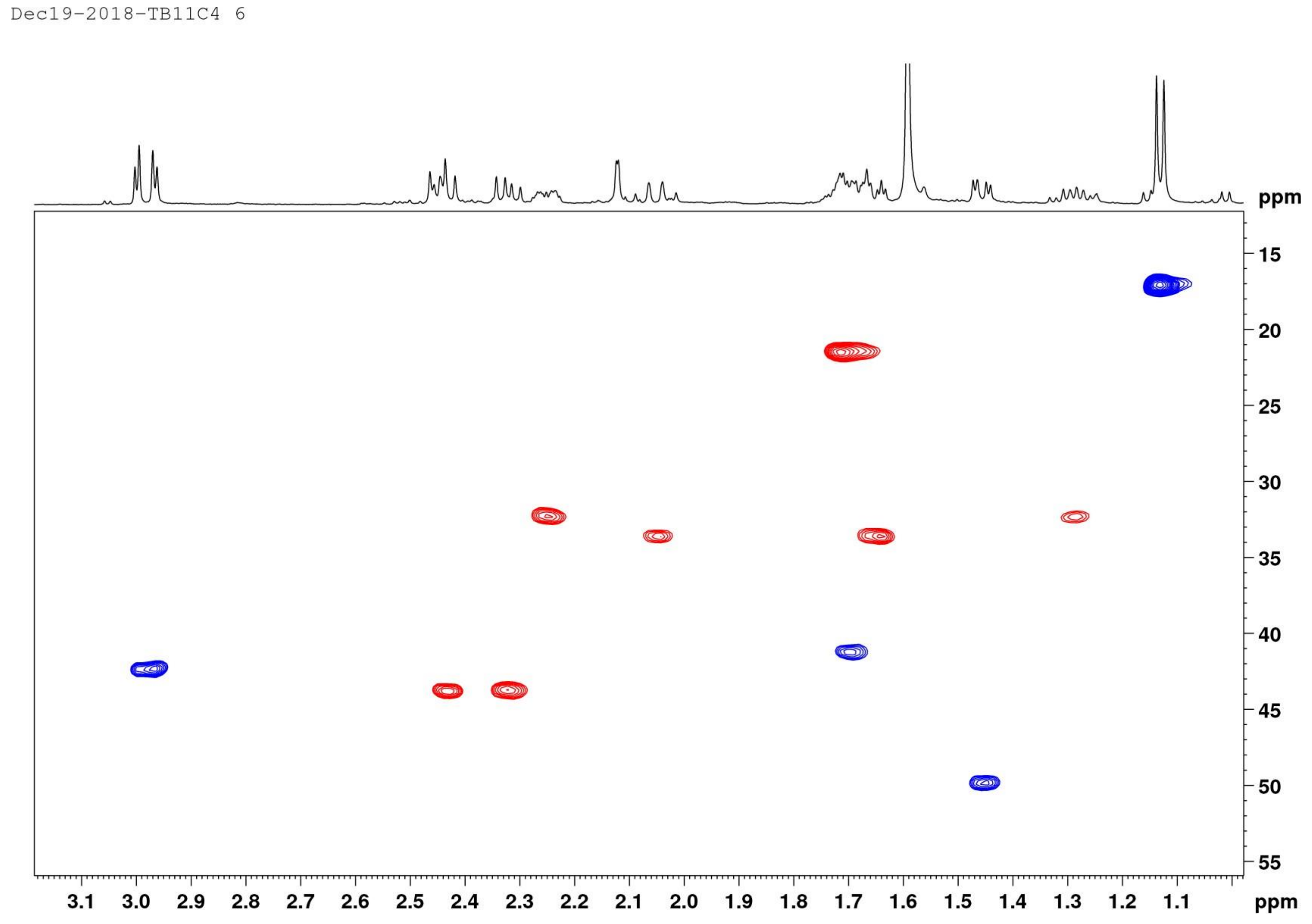

Figure S161. Expansion of part of HSQC NMR spectrum (δ 1.0 – δ 3.1) of **16** in $CDCl_3$.

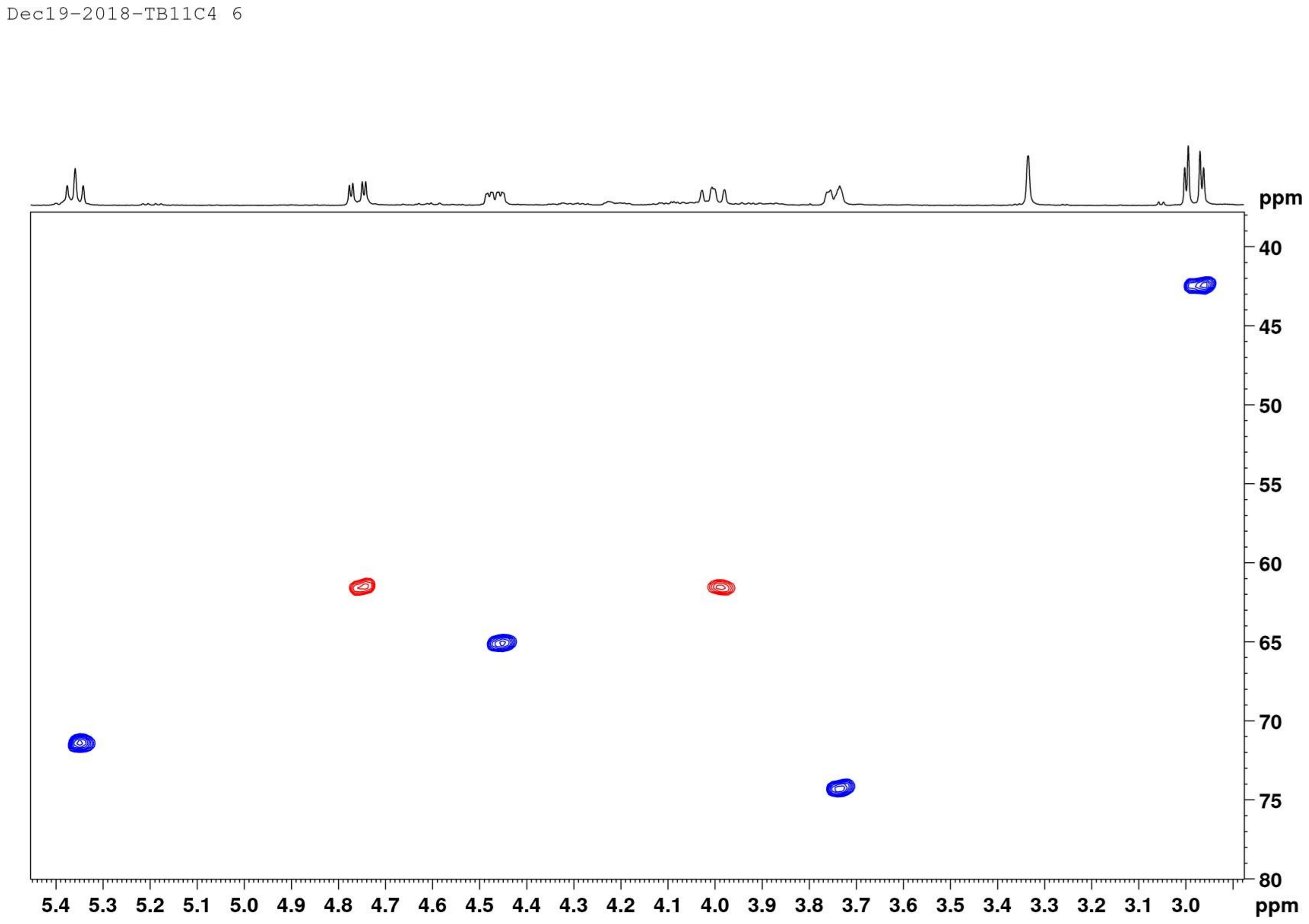

Figure S162. Expansion of part of HSQC NMR spectrum (δ 2.9 – δ 5.4) of **16** in $CDCl_3$.

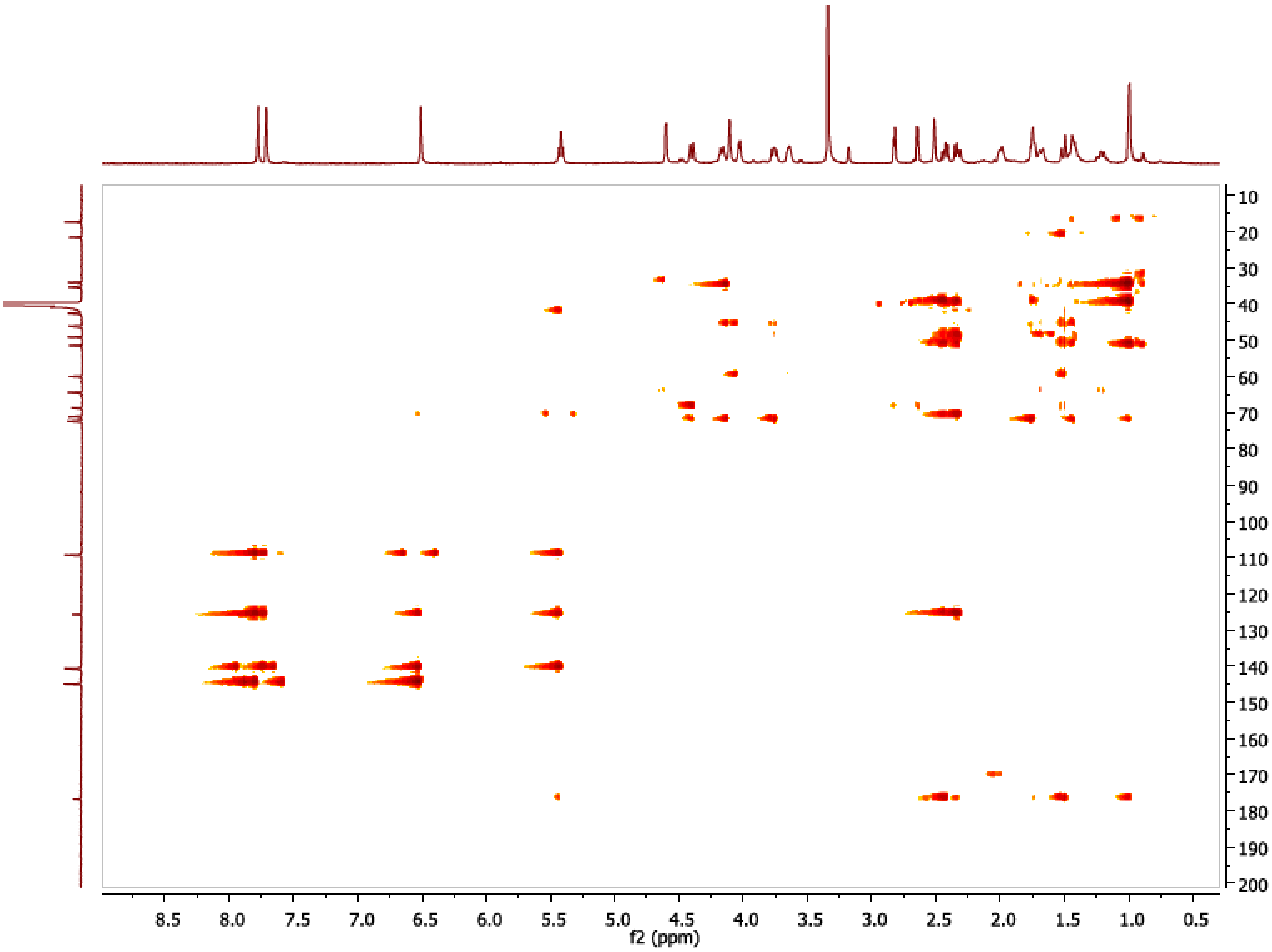

Figure S163. HMBC NMR spectrum of **16** in $(CD_3)_2SO$.

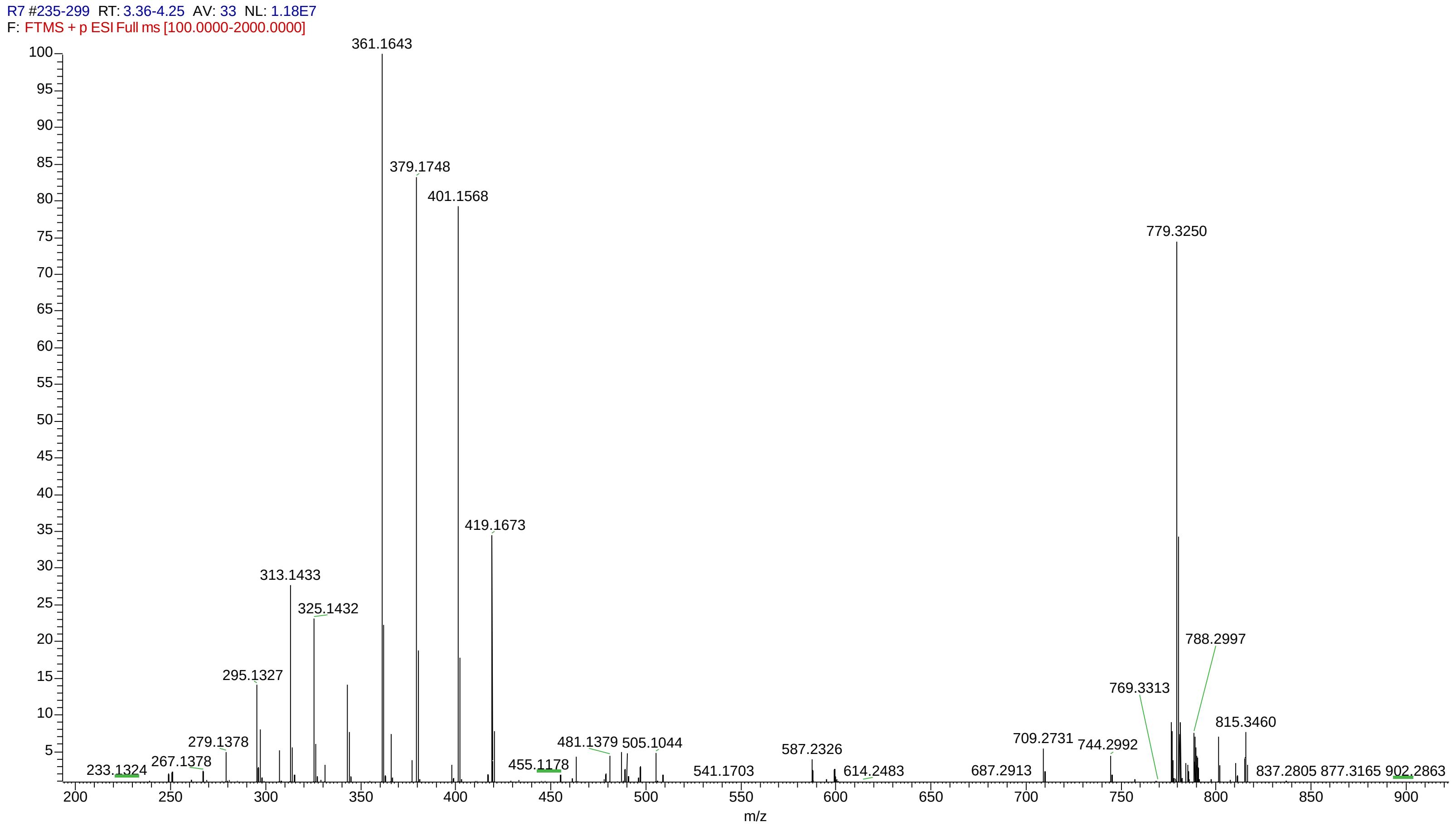

Figure S164. Positive-ion HRESIMS of **16**.

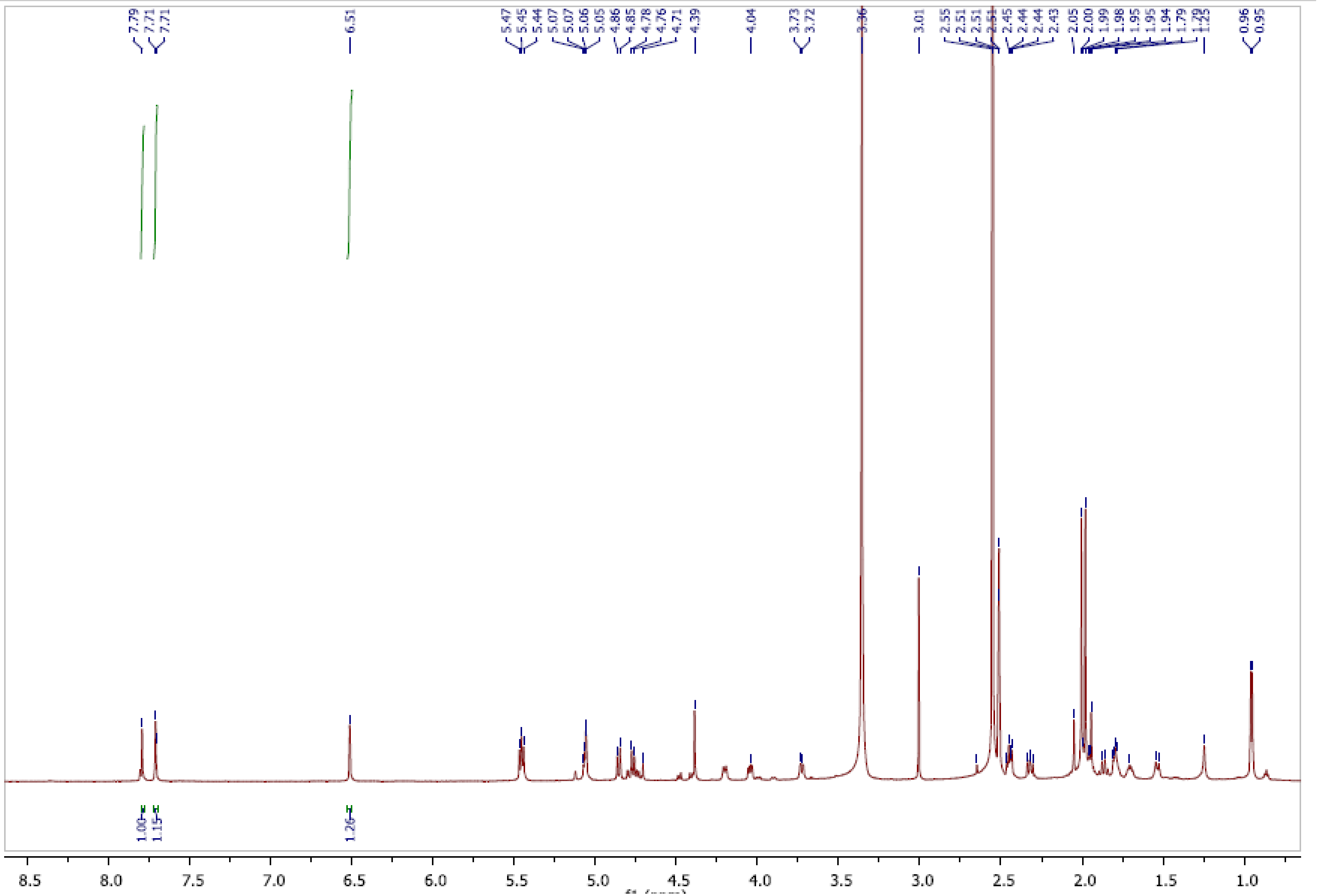

Figure S165. ^{1}H NMR spectrum of **17** in $(CD_3)_2SO$.

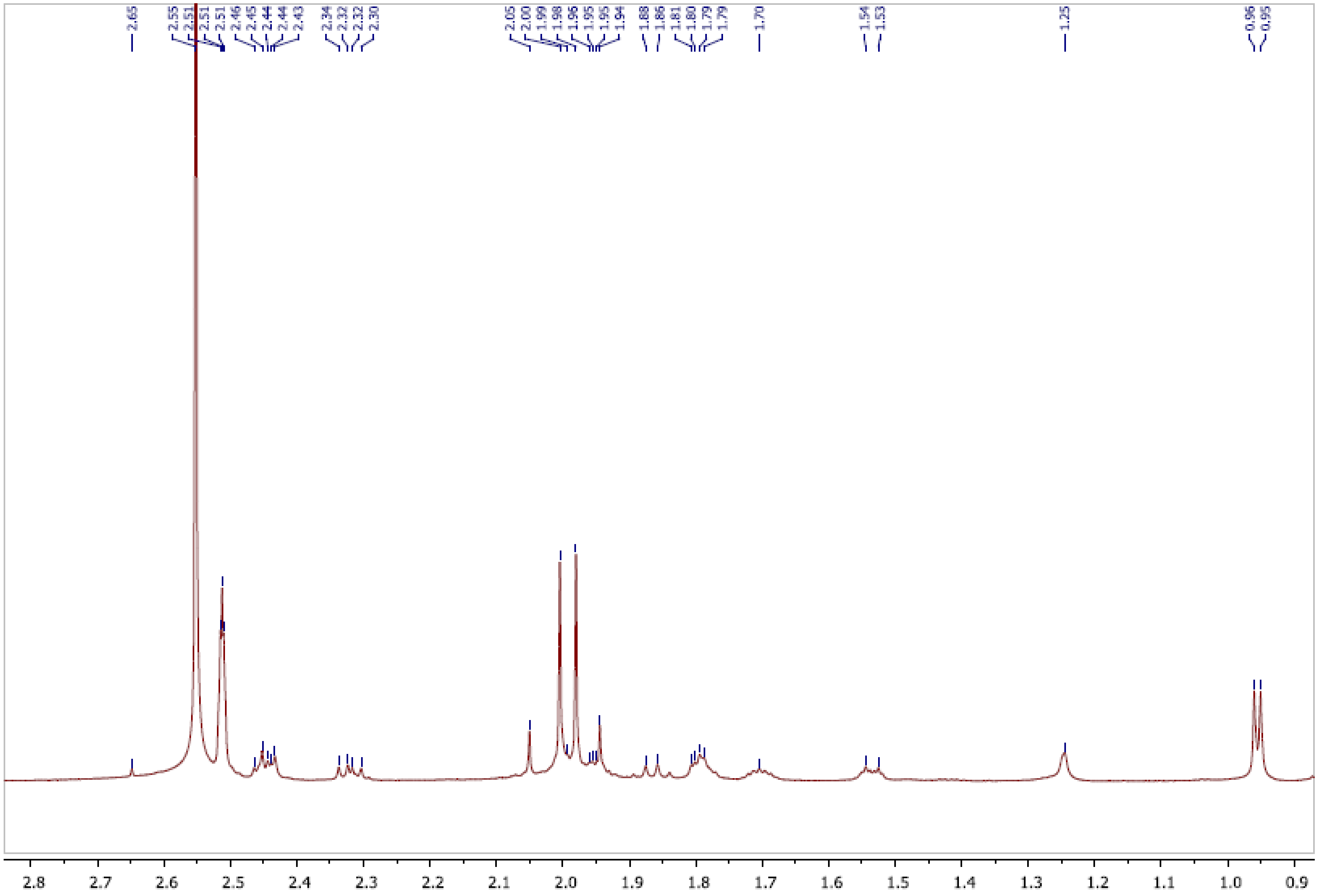

Figure S166. Expansion of part of ^{1}H NMR spectrum (δ 0.9 – δ 2.8) of **17** in $(CD_3)_2SO$.

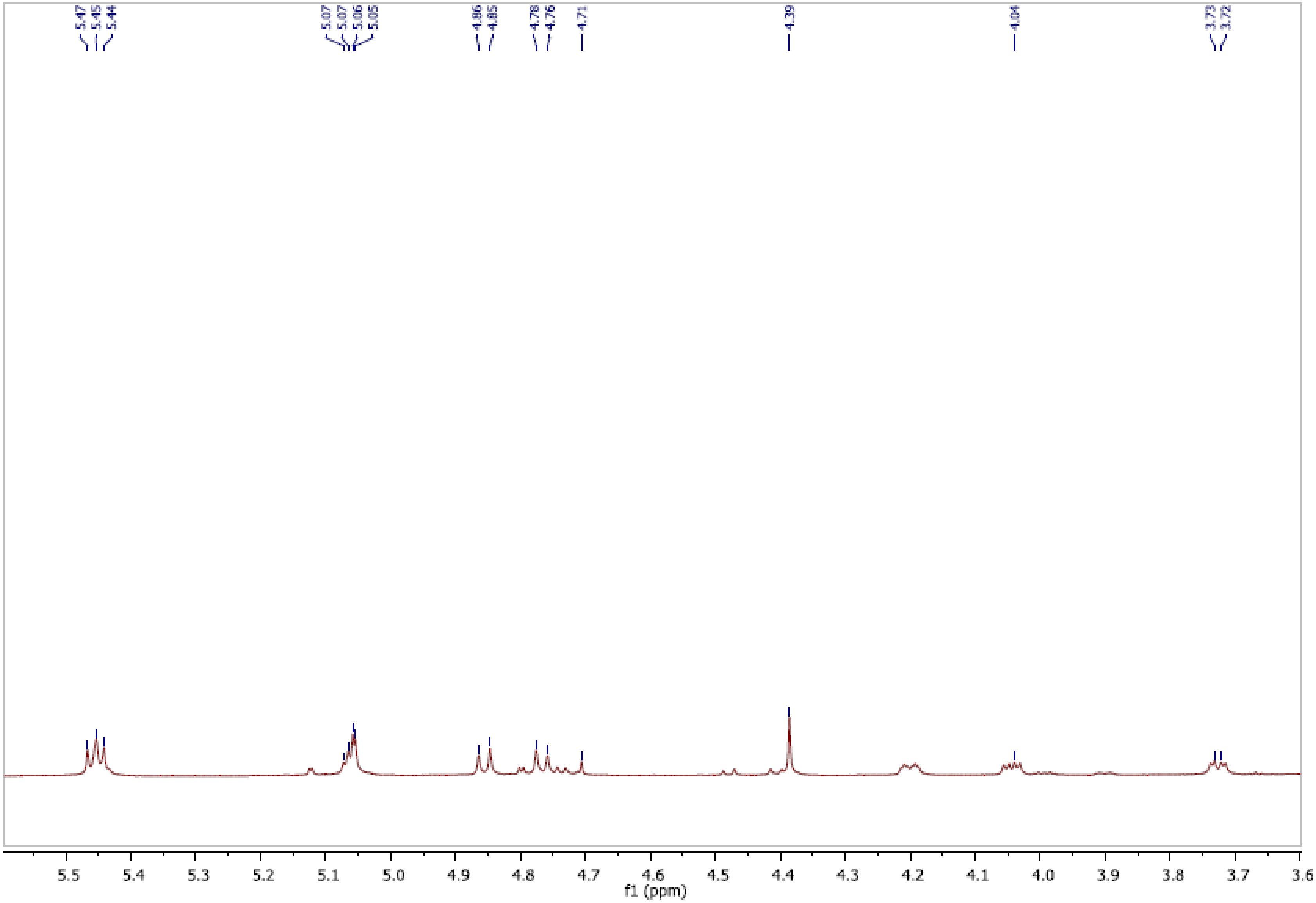

Figure S167. Expansion of part of ^{1}H NMR spectrum (δ 3.6 – δ 5.5) of **17** in $(CD_3)_2SO$.

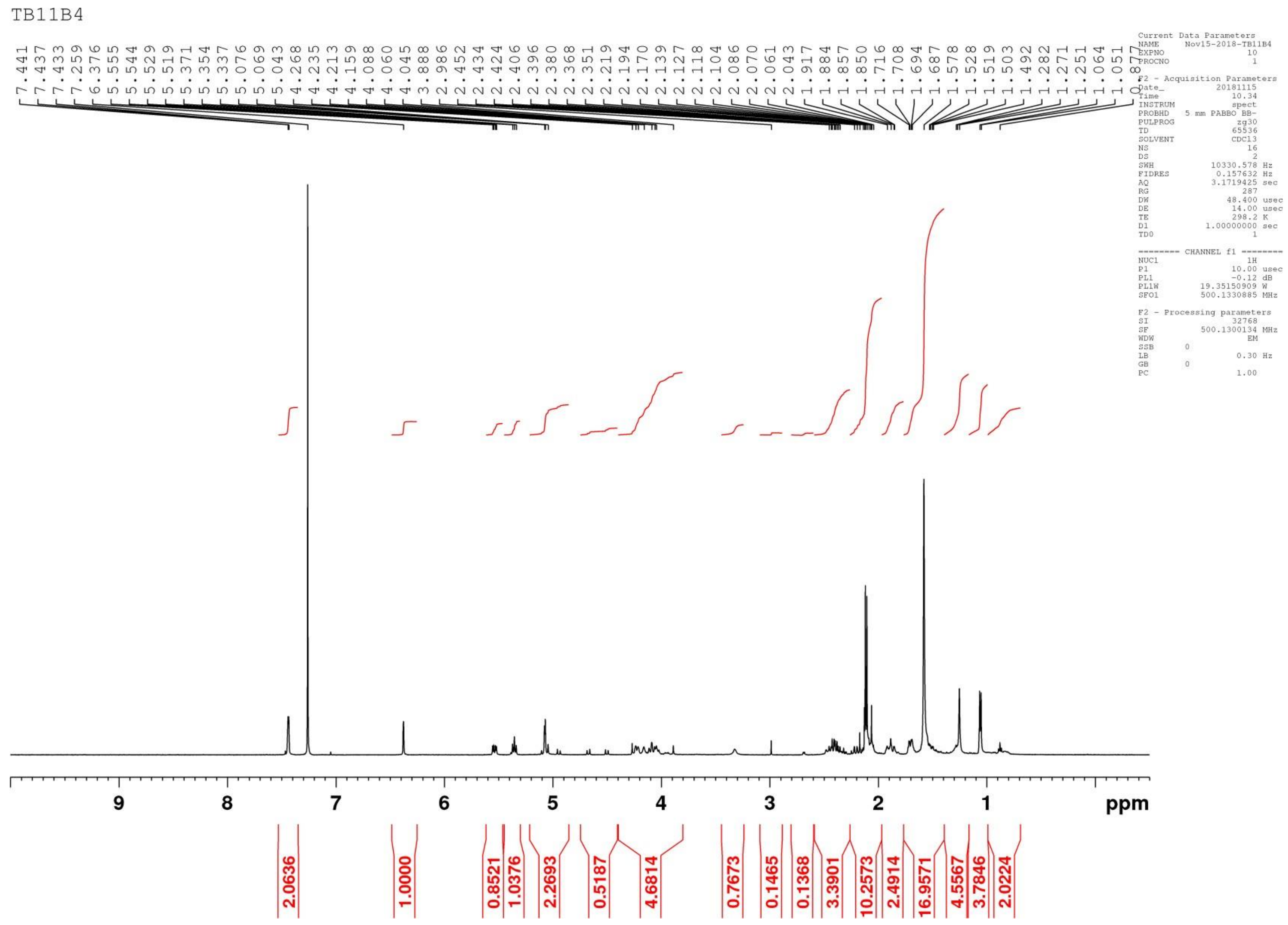

Figure S168. ^{1}H NMR spectrum of **17** in $CDCl_3$.

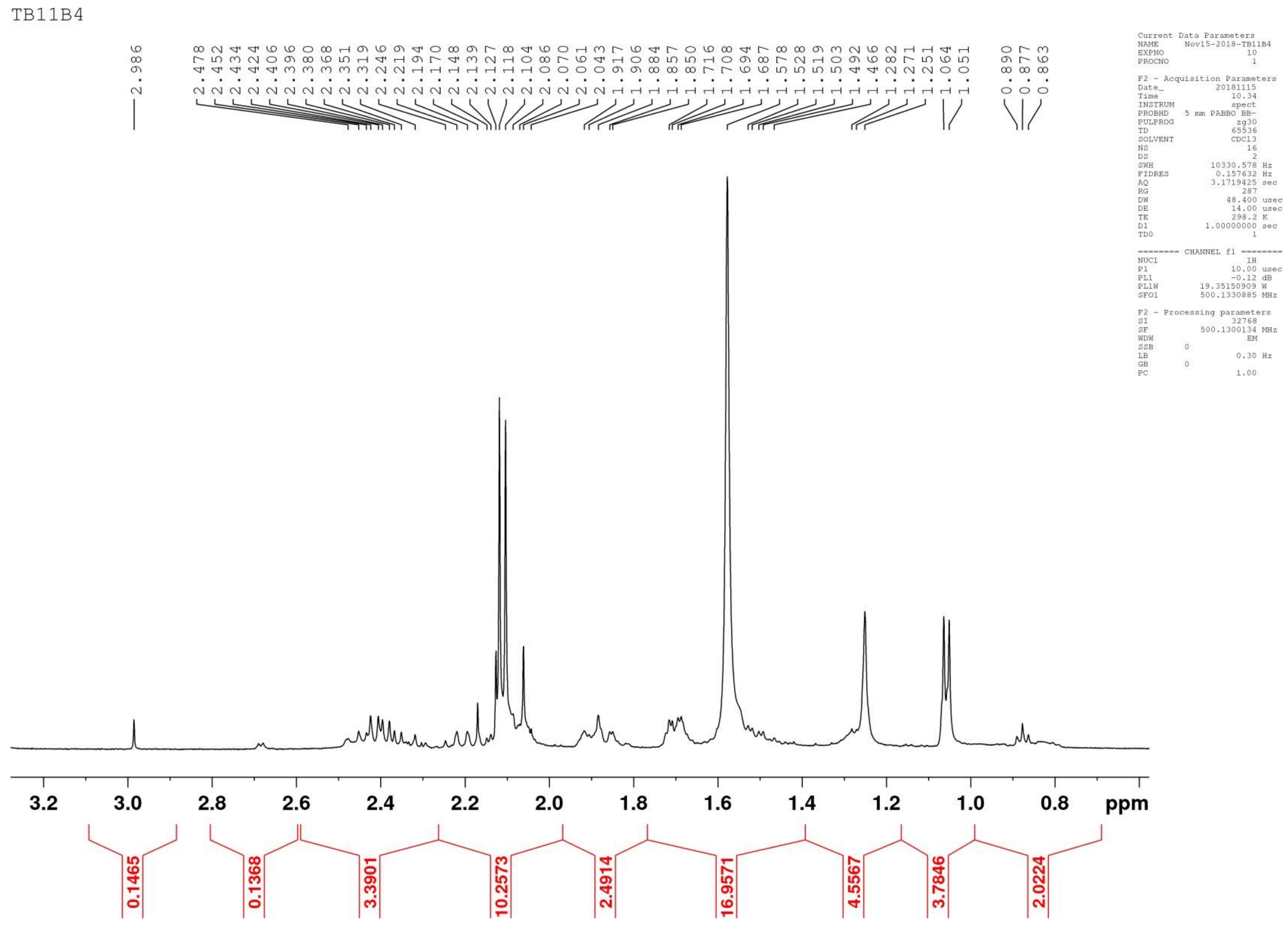

Figure S169. Expansion of part of ^{1}H NMR spectrum (δ 0.7 – δ 3.2) of **17** in $CDCl_3$.

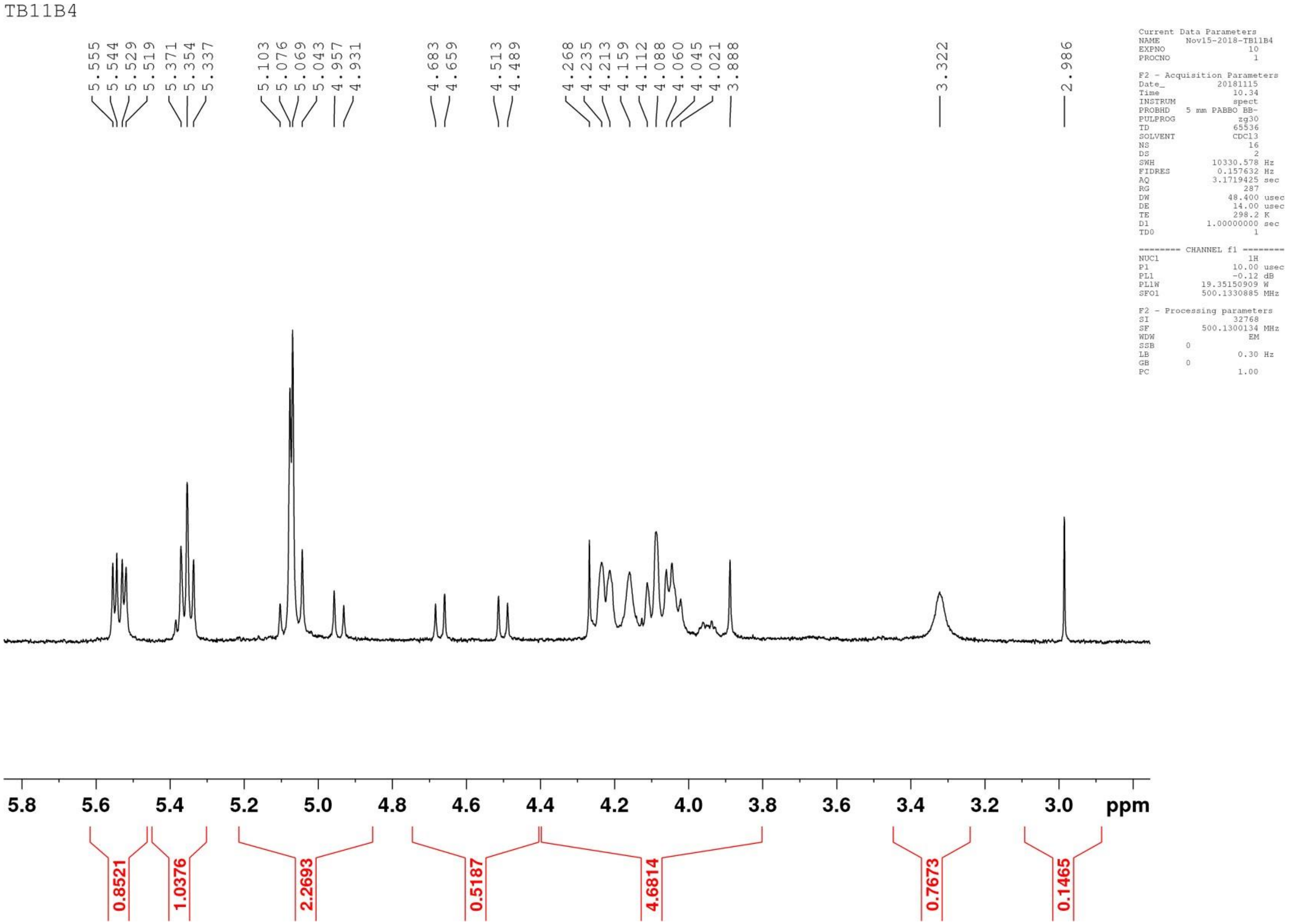

Figure S170. Expansion of part of ^{1}H NMR spectrum (δ 2.8 – δ 5.8) of **17** in $CDCl_3$.

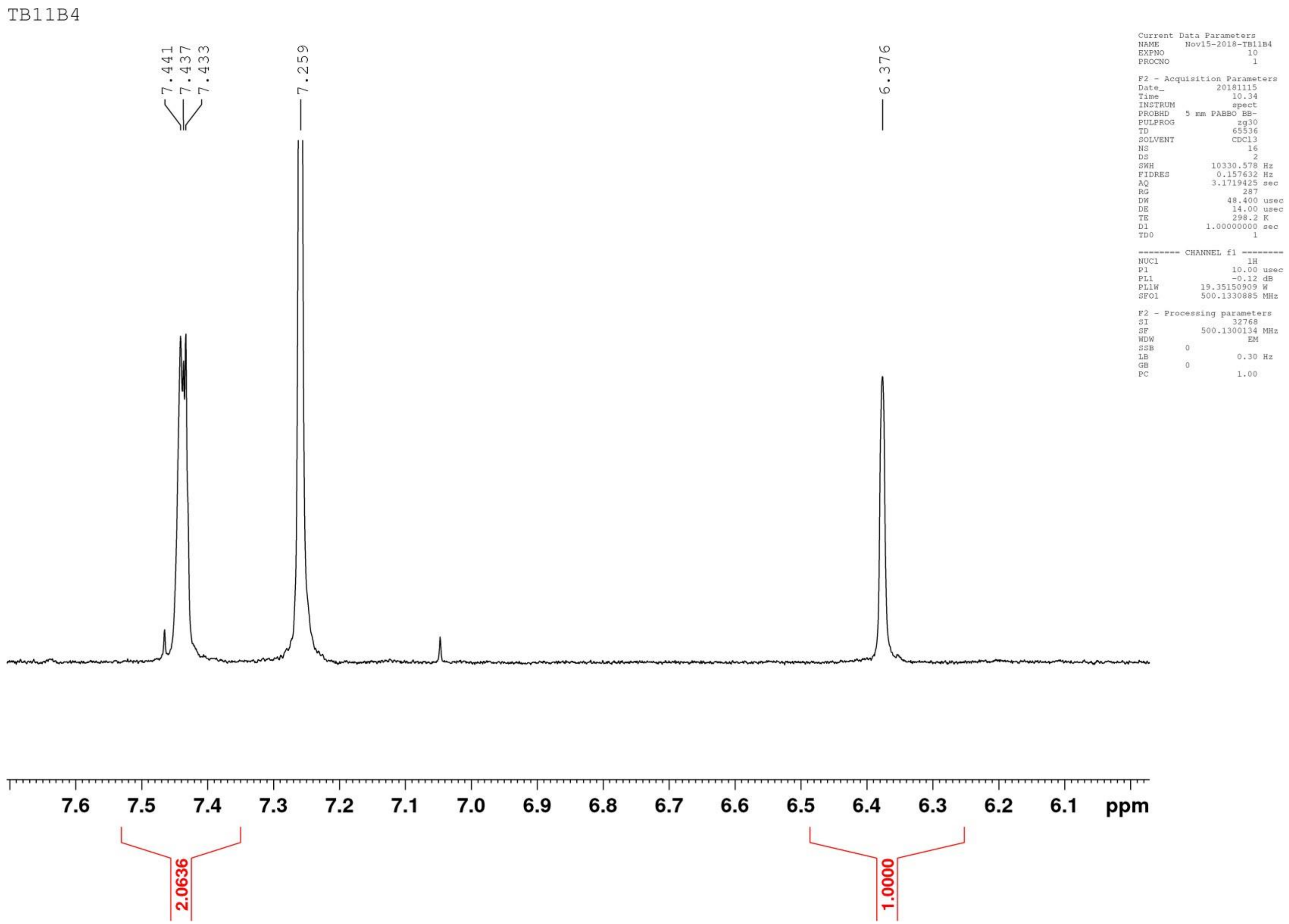

Figure S171. Expansion of part of ^{1}H NMR spectrum (δ 6.0 – δ 7.7) of **17** in $CDCl_3$.

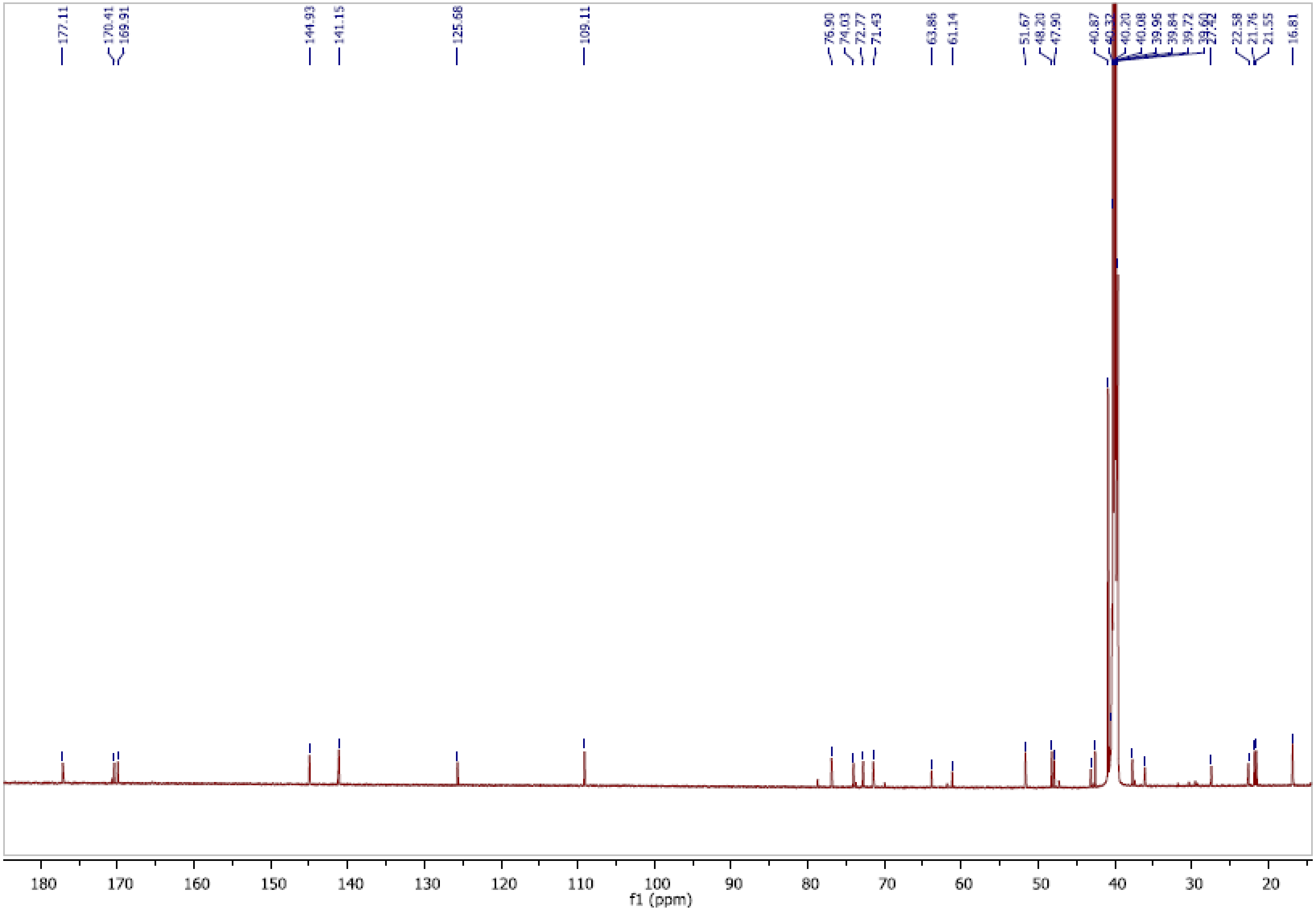

Figure S172. ^{13}C NMR spectrum of **17** in $(CD_3)_2SO$.

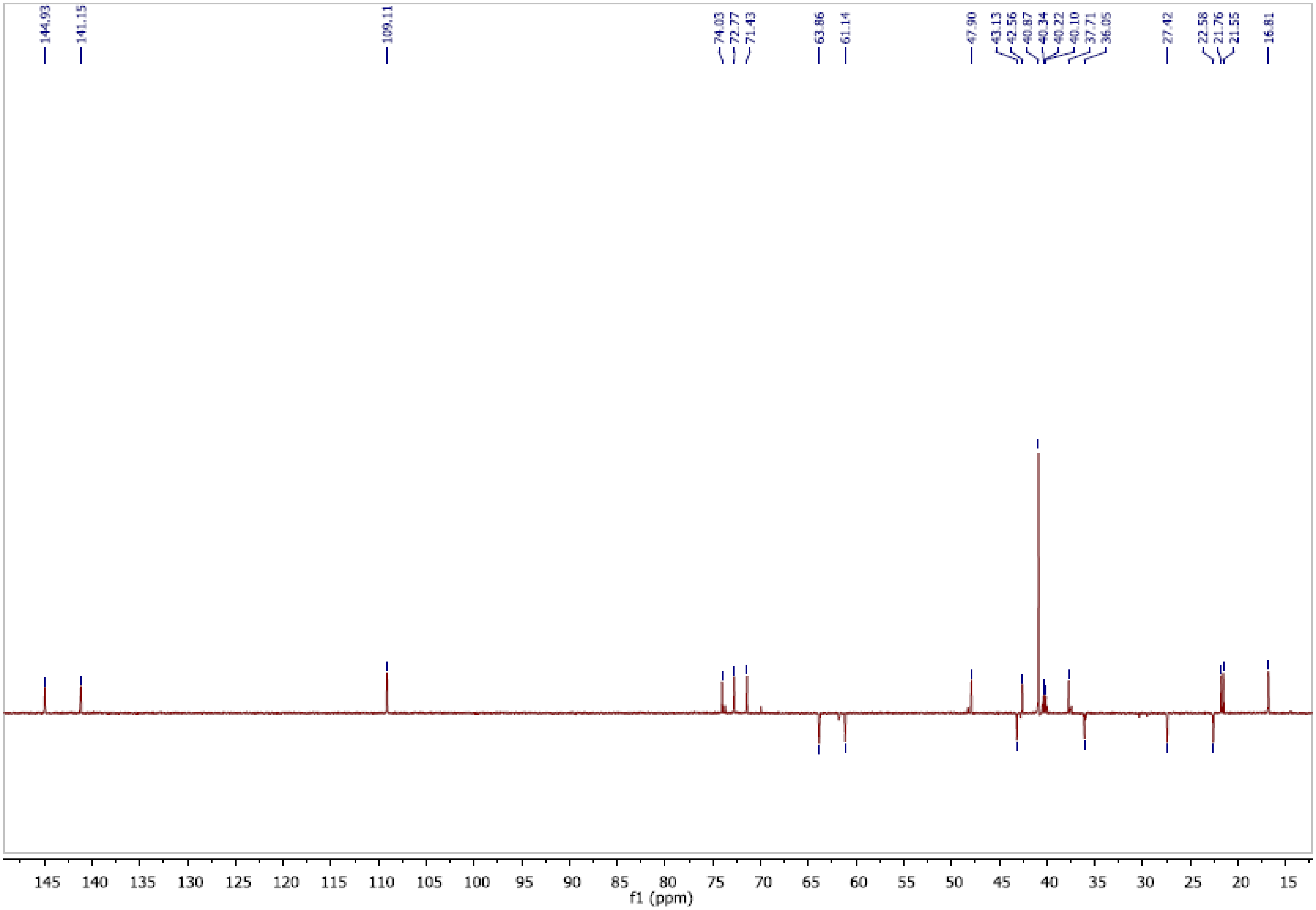

Figure S173. 135DEPT NMR spectrum of **17** in $(CD_3)_2SO$.

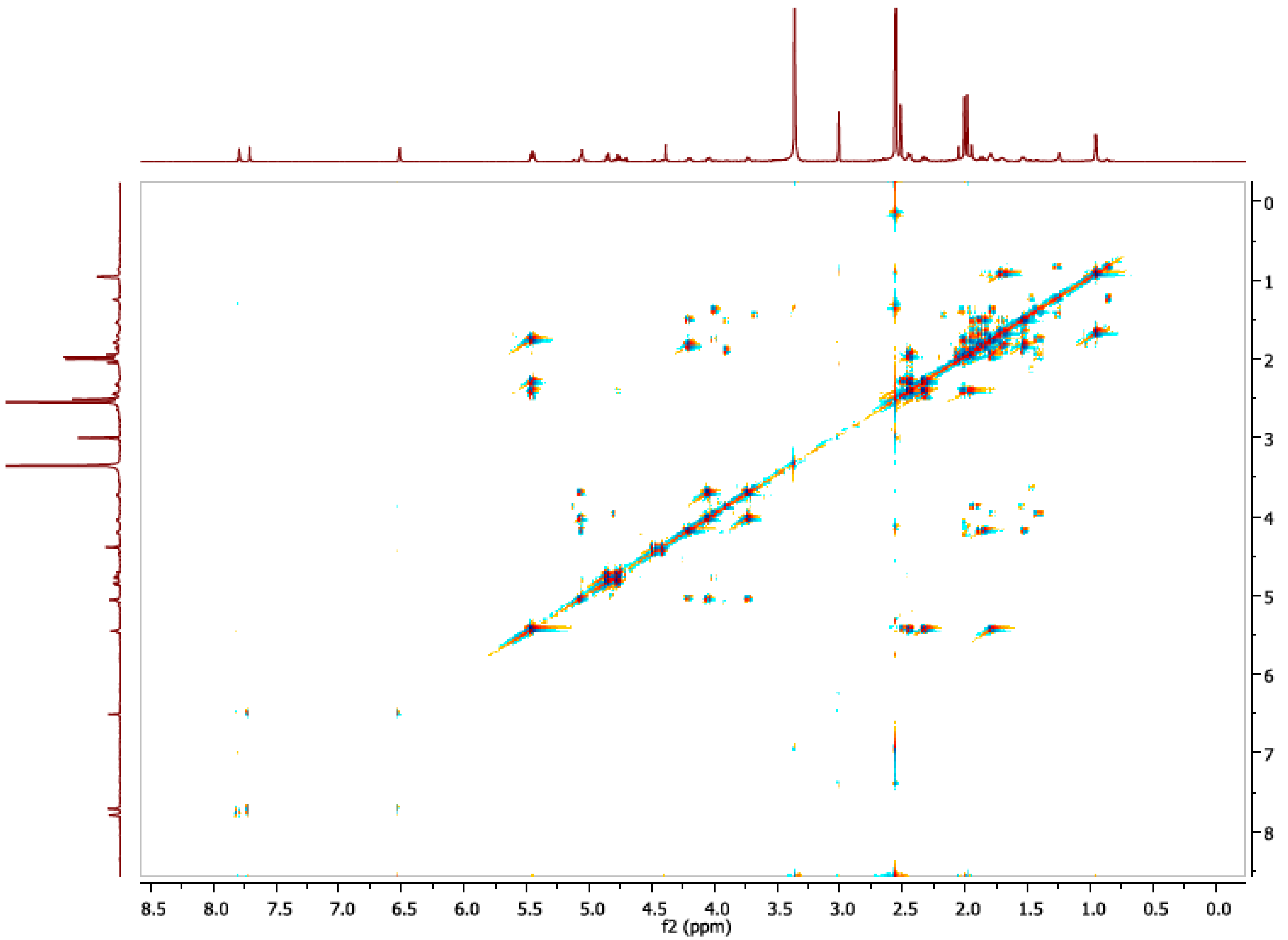

Figure S174. COSY NMR spectrum of **17** in $(CD_3)_2SO$.

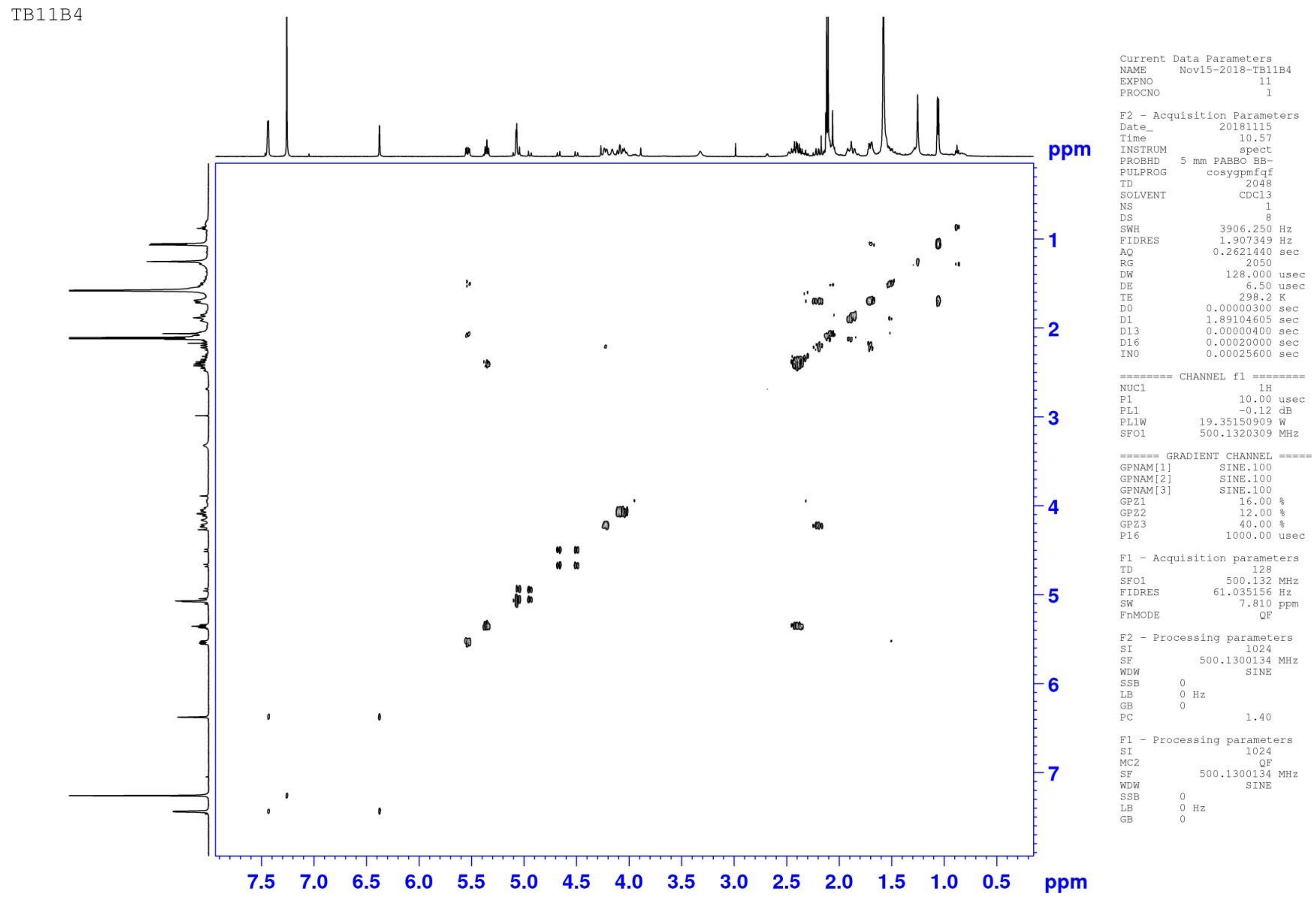

Figure S175. COSY NMR spectrum of **17** in $CDCl_3$.

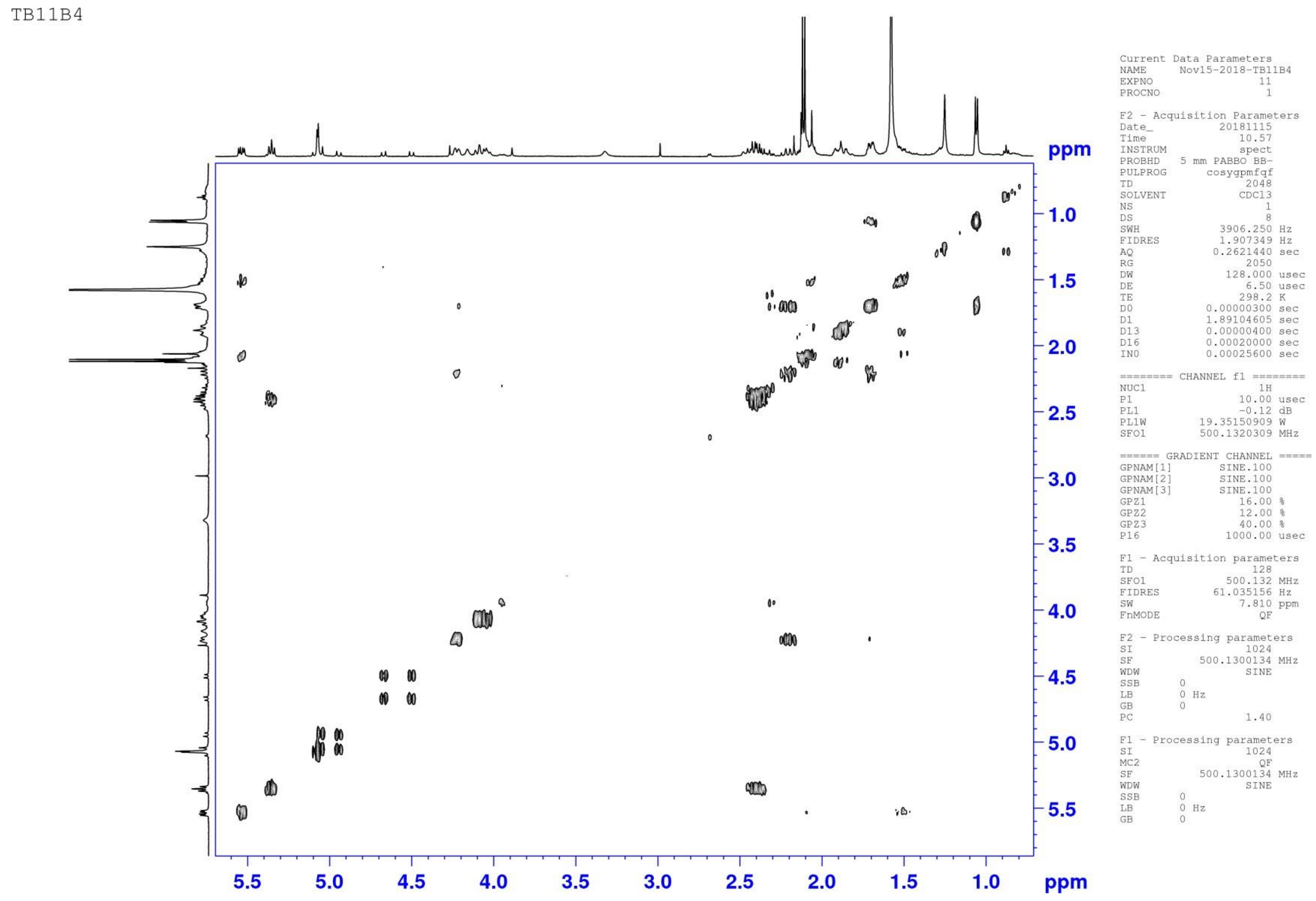

Figure S176. Expansion of part of COSY NMR spectrum (δ 0.5 – δ 5.5) of **17** in $CDCl_3$.

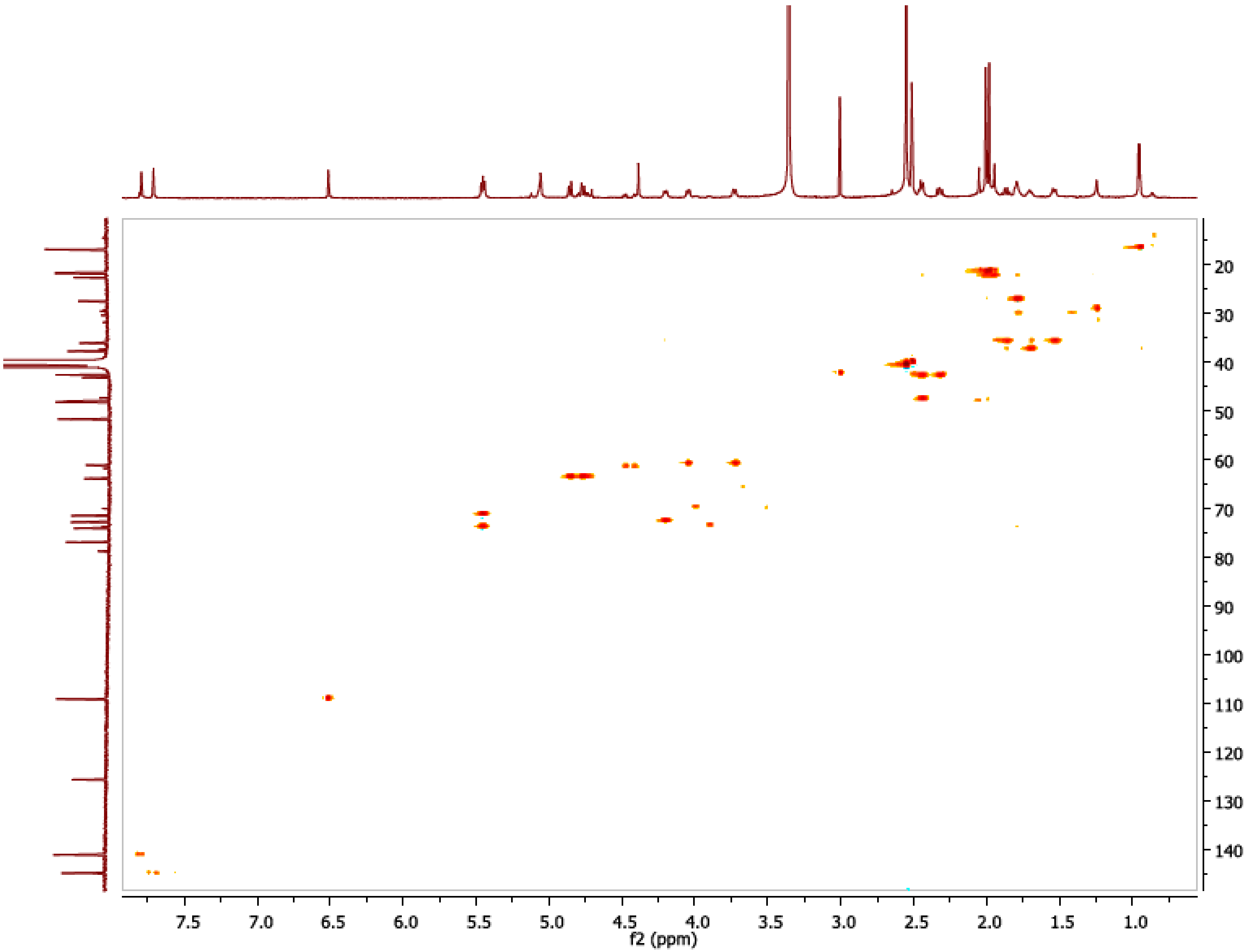

Figure S177. HSQC NMR spectrum of **17** in $(CD_3)_2SO$.

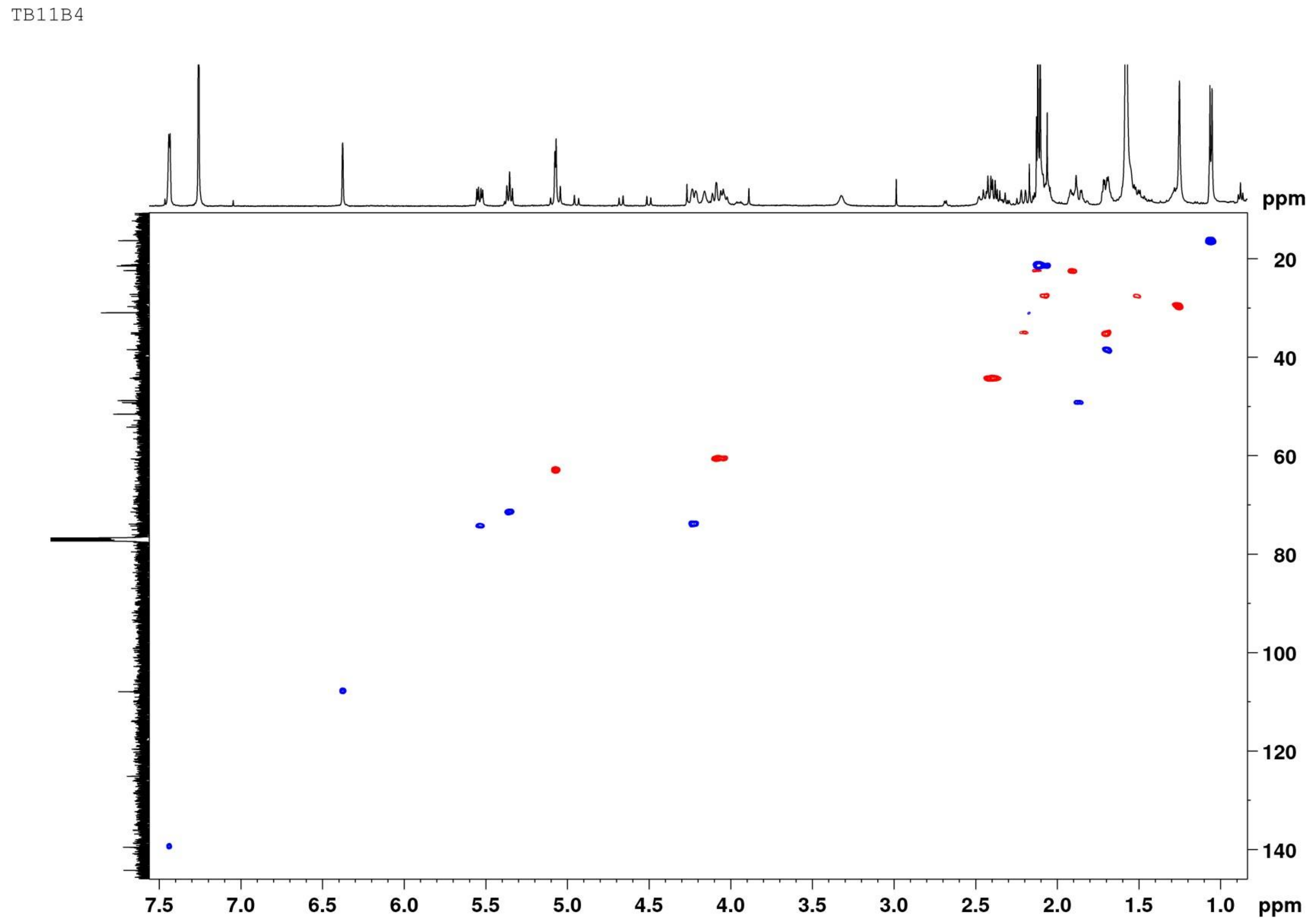

Figure S178. HSQC NMR spectrum of **17** in $CDCl_3$.

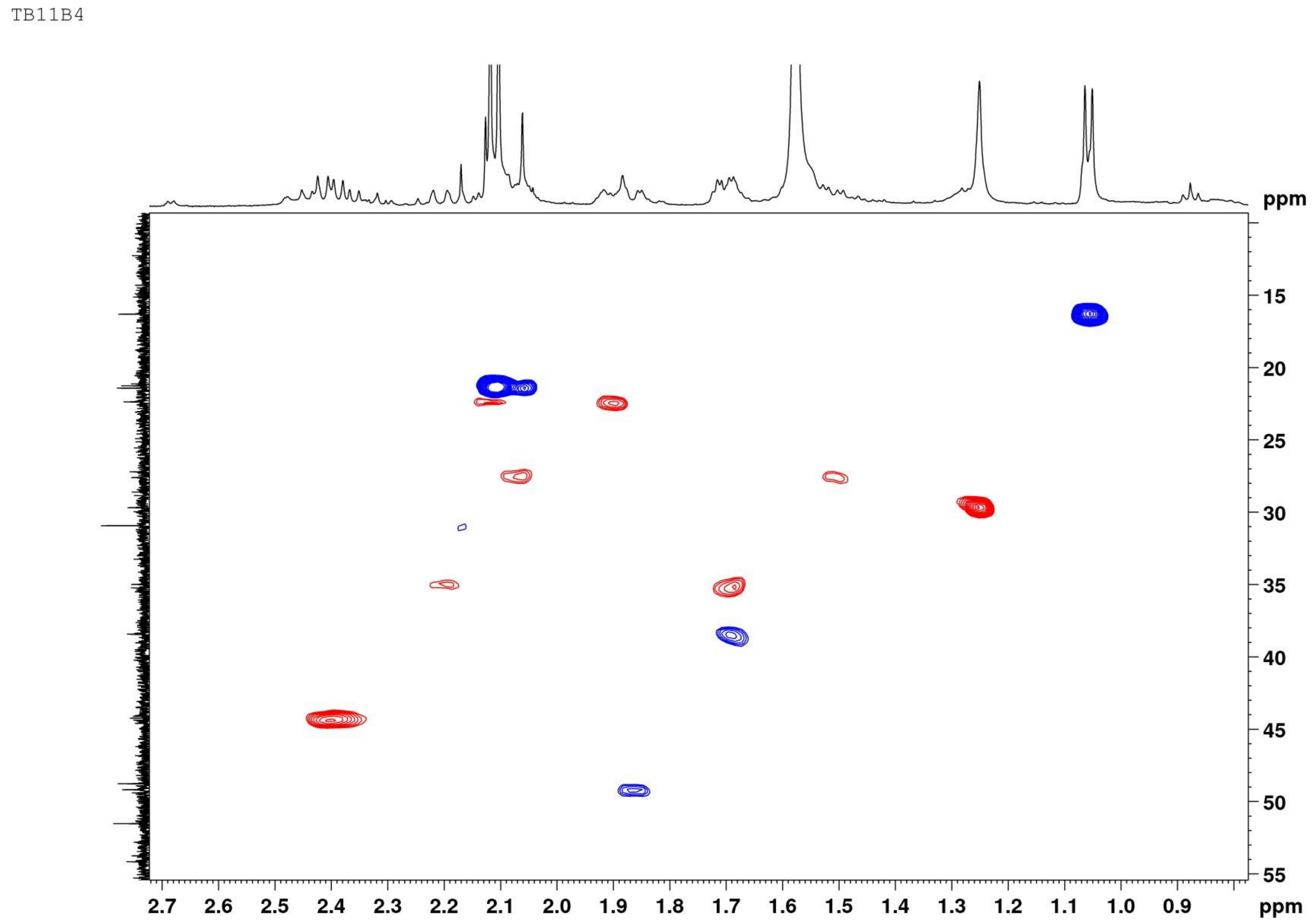

Figure S179. Expansion of part of HSQC NMR spectrum (δ 0.8 – δ 2.7) of **17** in $CDCl_3$.

TB11B4

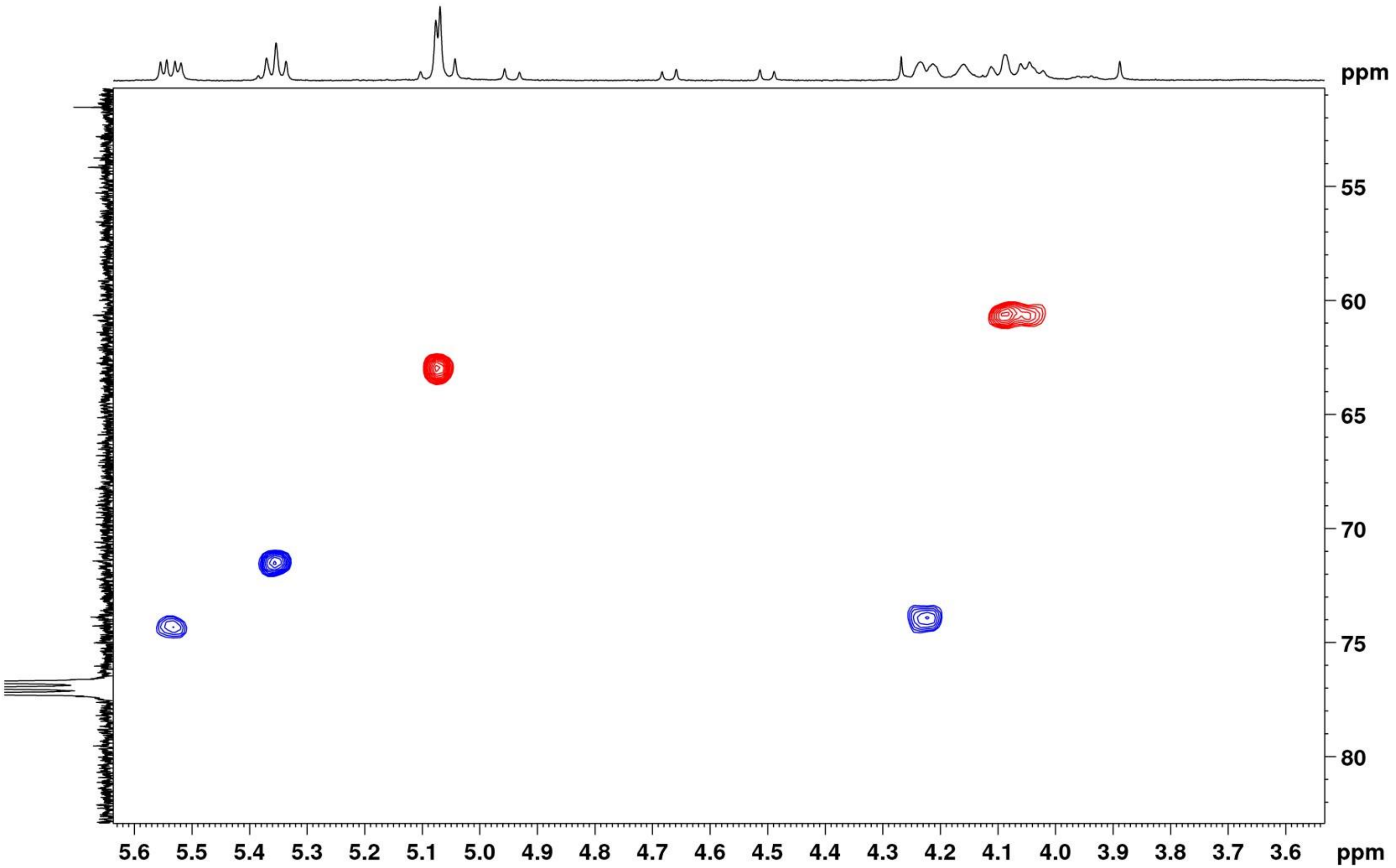

Figure S180. Expansion of part of HSQC NMR spectrum (δ 3.6 – δ 5.6) of **17** in $CDCl_3$.

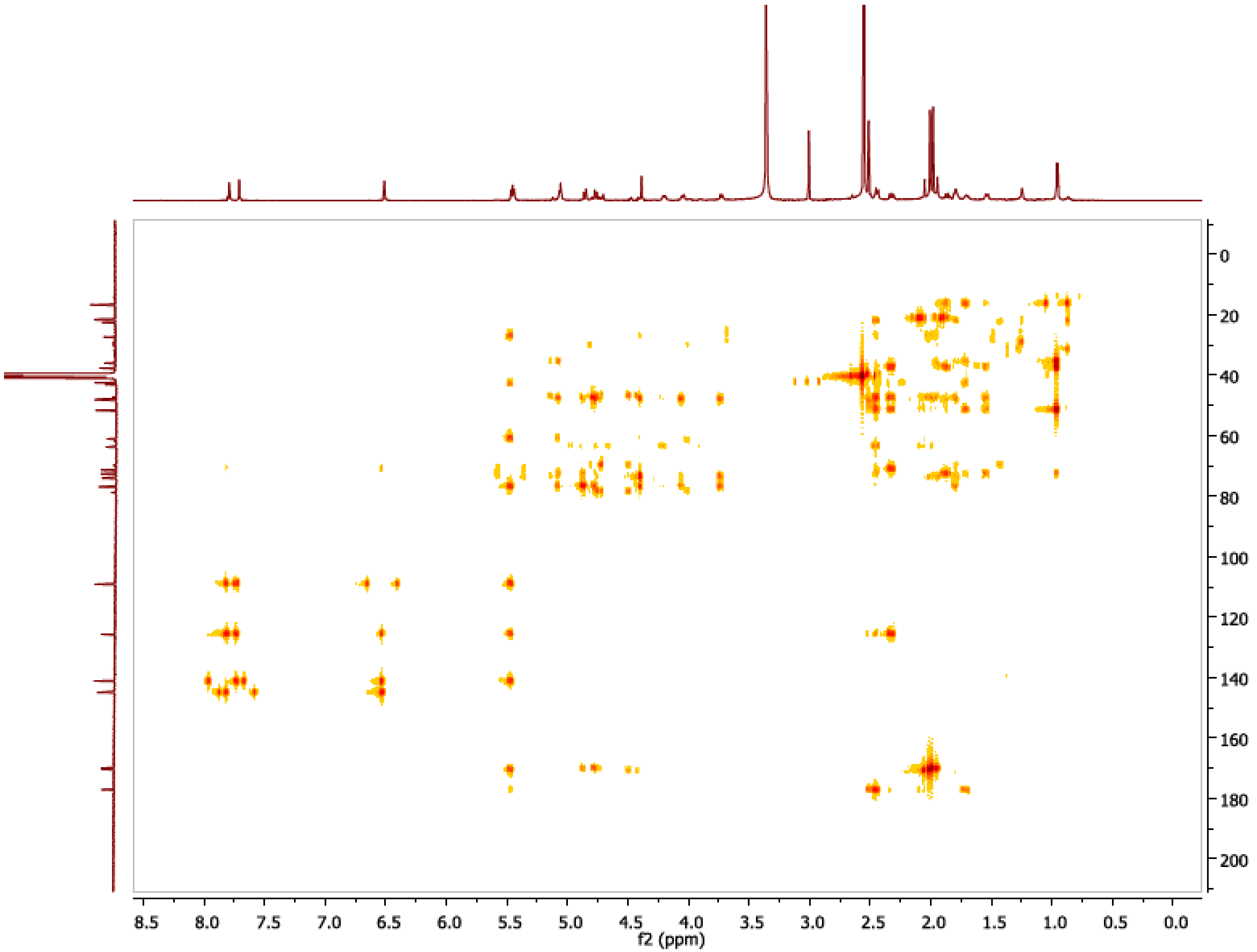

Figure S181. HMBC NMR spectrum of **17** in $(CD_3)_2SO$.

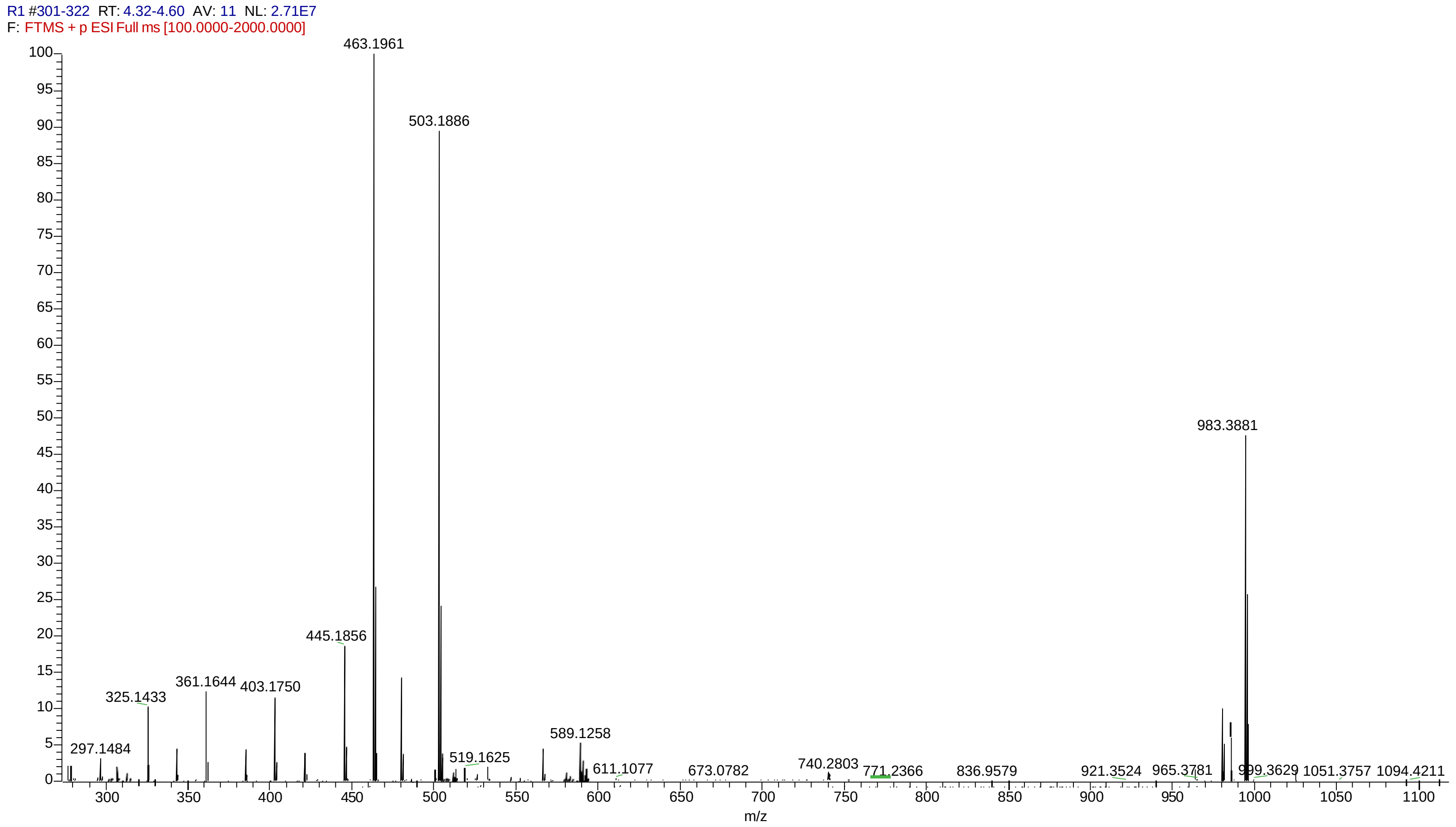
R1 #301-322 RT: 4.32-4.60 AV: 11 NL: 2.71E7
F: FTMS + p ESI Full ms [100.0000-2000.0000]
463.1961
503.1886
983.3881
445.1856
325.1433
361.1644
403.1750
297.1484
589.1258
519.1625
611.1077
673.0782
740.2803
771.2366
836.9579
921.3524
965.3781
999.3629
1051.3757
1094.4211
m/z

Figure S182. Positive-ion HRESIMS of **17**.

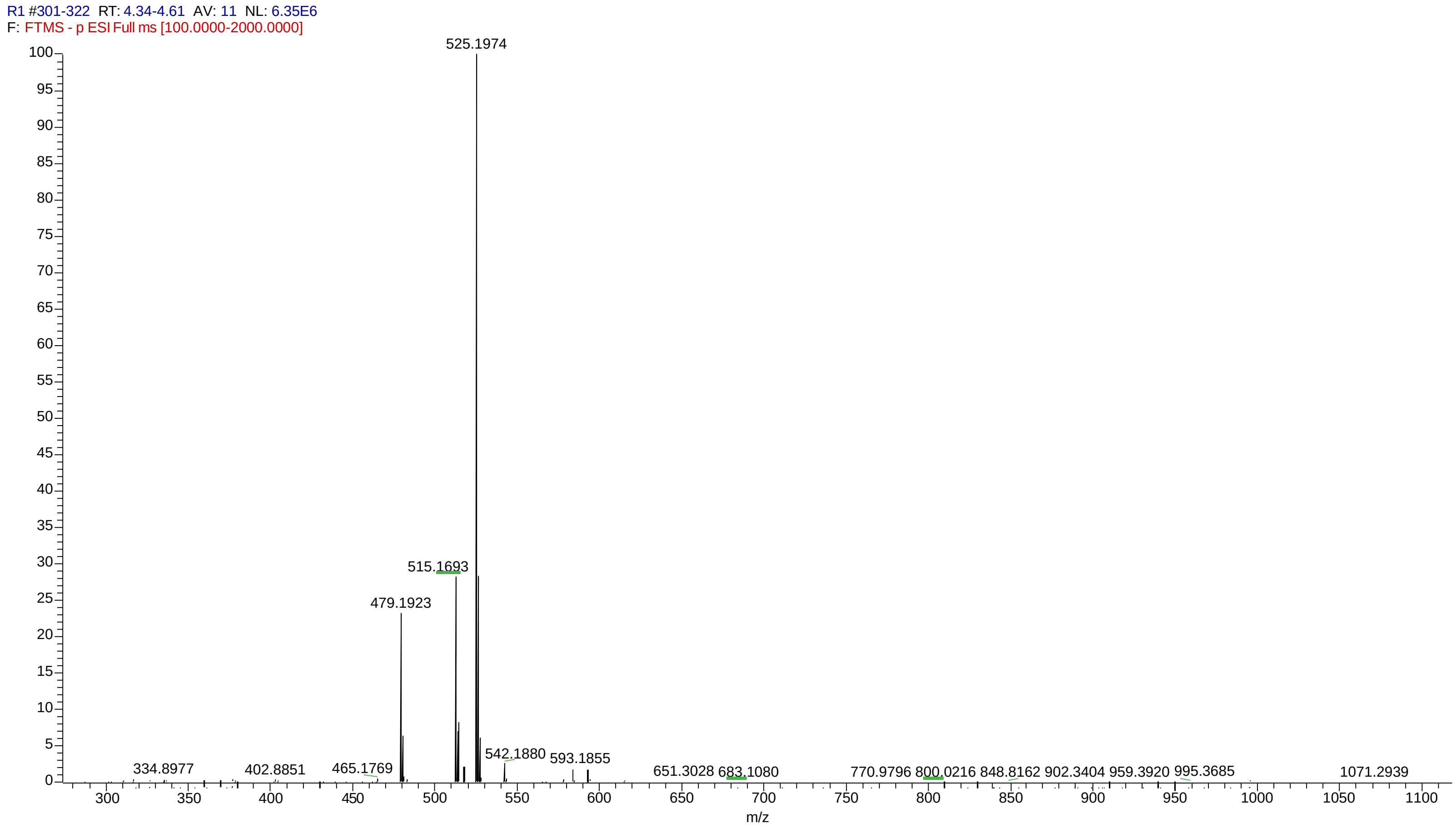

R1 #301-322 RT: 4.34-4.61 AV: 11 NL: 6.35E6
F: FTMS - p ESI Full ms [100.0000-2000.0000]
525.1974
515.1693
479.1923
334.8977
402.8851
465.1769
542.1880
593.1855
651.3028
683.1080
770.9796
800.0216
848.8162
902.3404
959.3920
995.3685
1071.2939
m/z

Figure S183. Negative-ion HRESIMS of **17**.

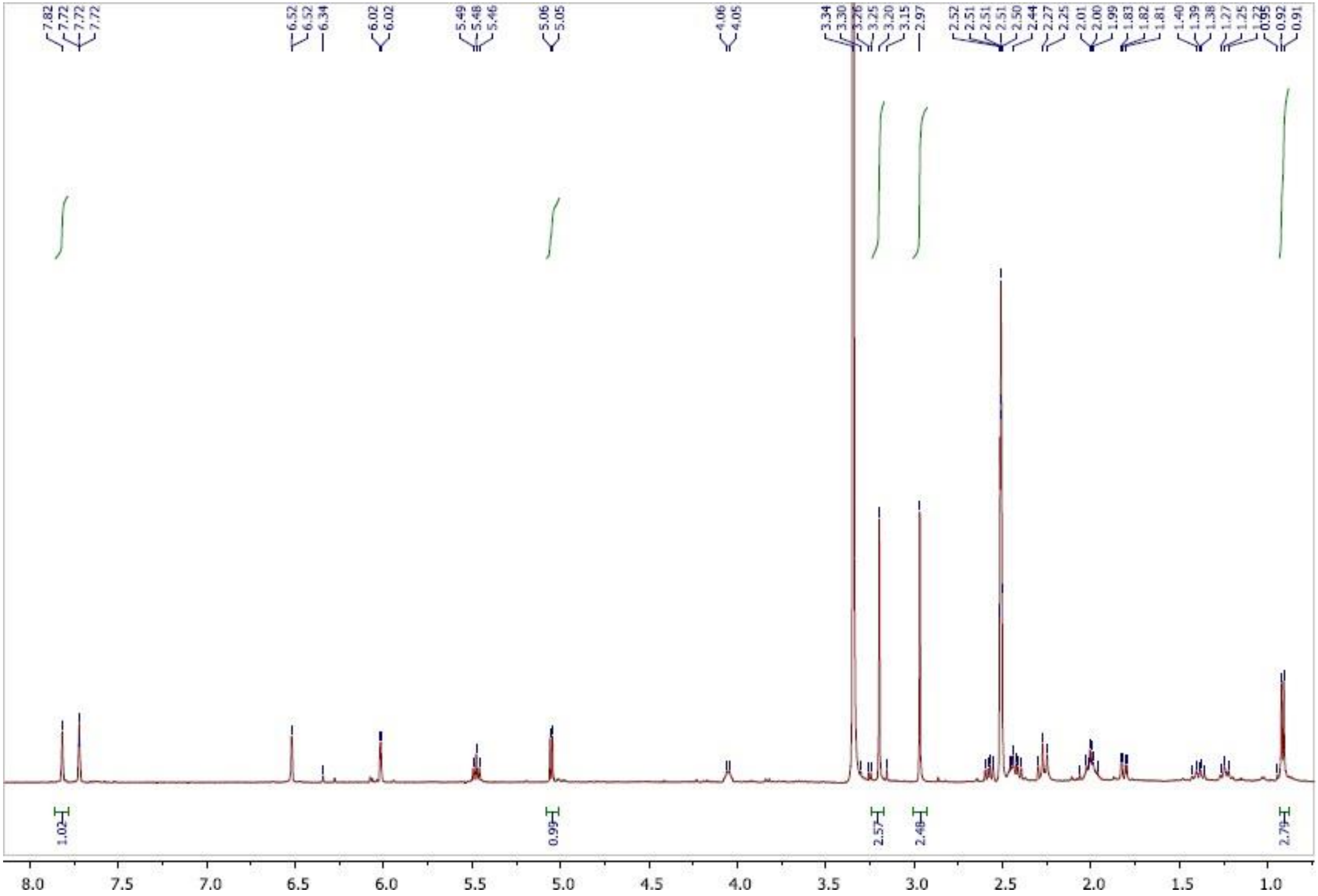

Figure S184. ^{1}H NMR spectrum of **18** in $(CD_3)_2SO$.

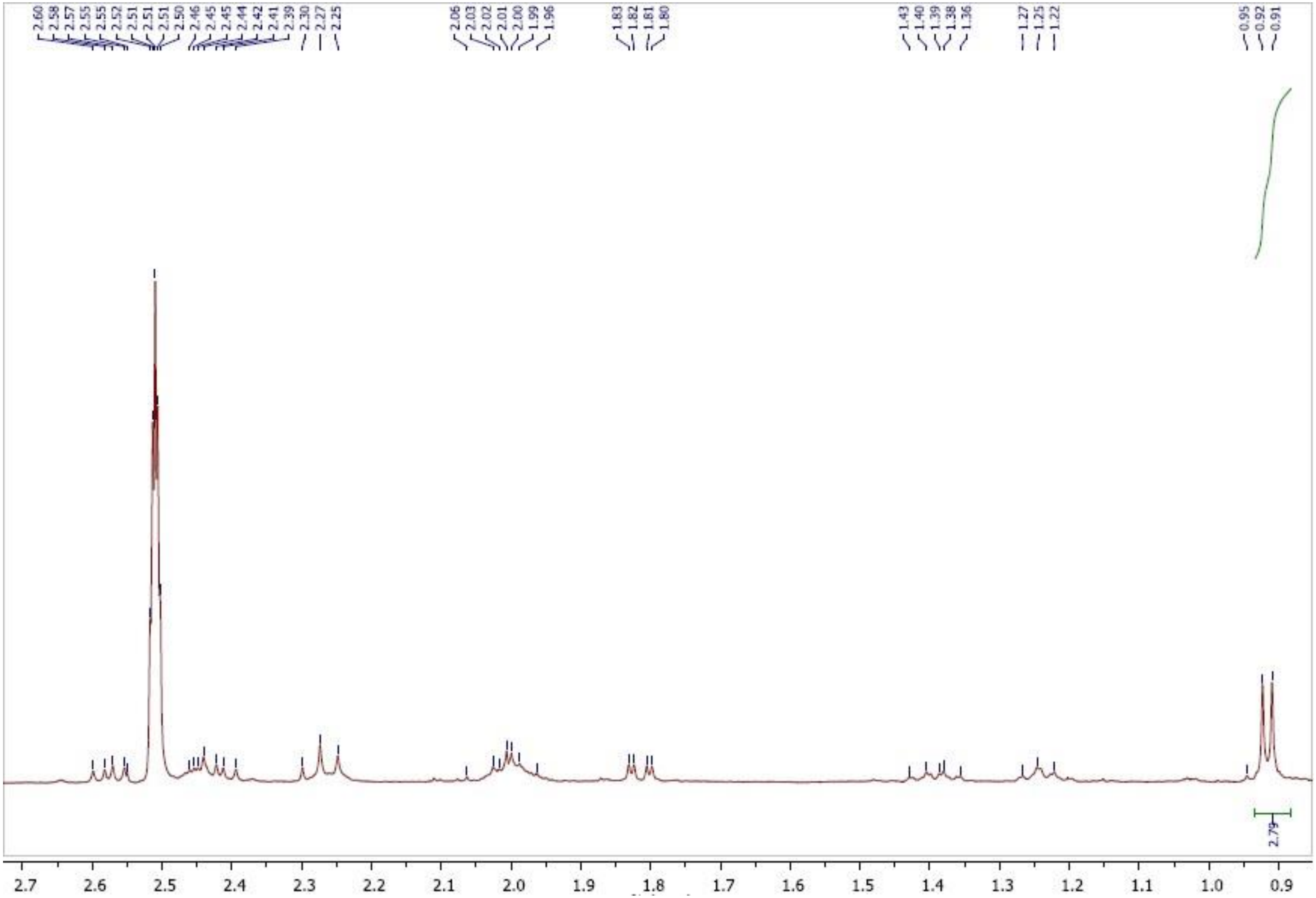

Figure S185. Expansion of part of ^{1}H NMR spectrum (δ 0.9 – δ 2.7) of **18** in $(CD_3)_2SO$.

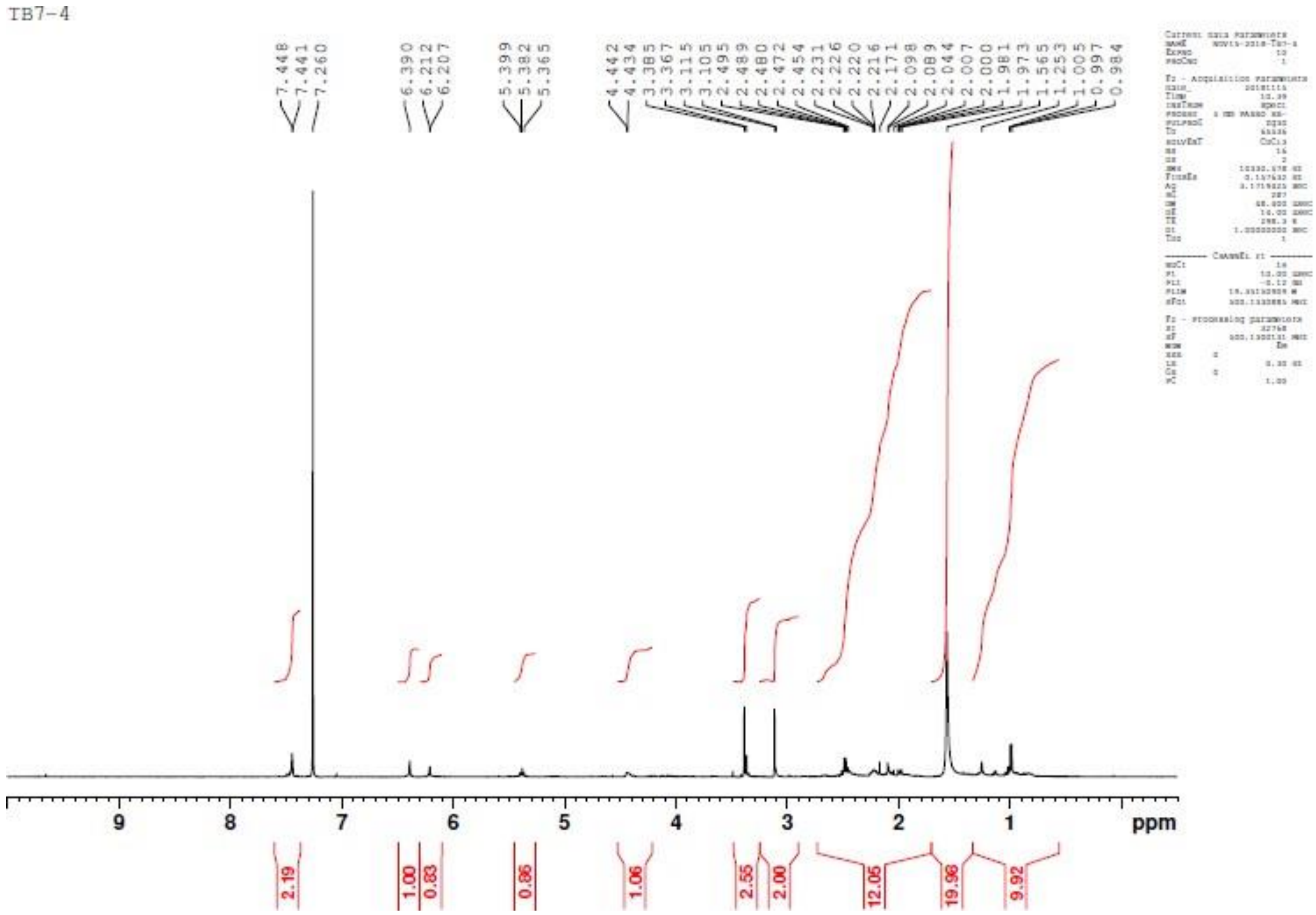

Figure S186. ^{1}H NMR spectrum of **18** in $CDCl_3$.

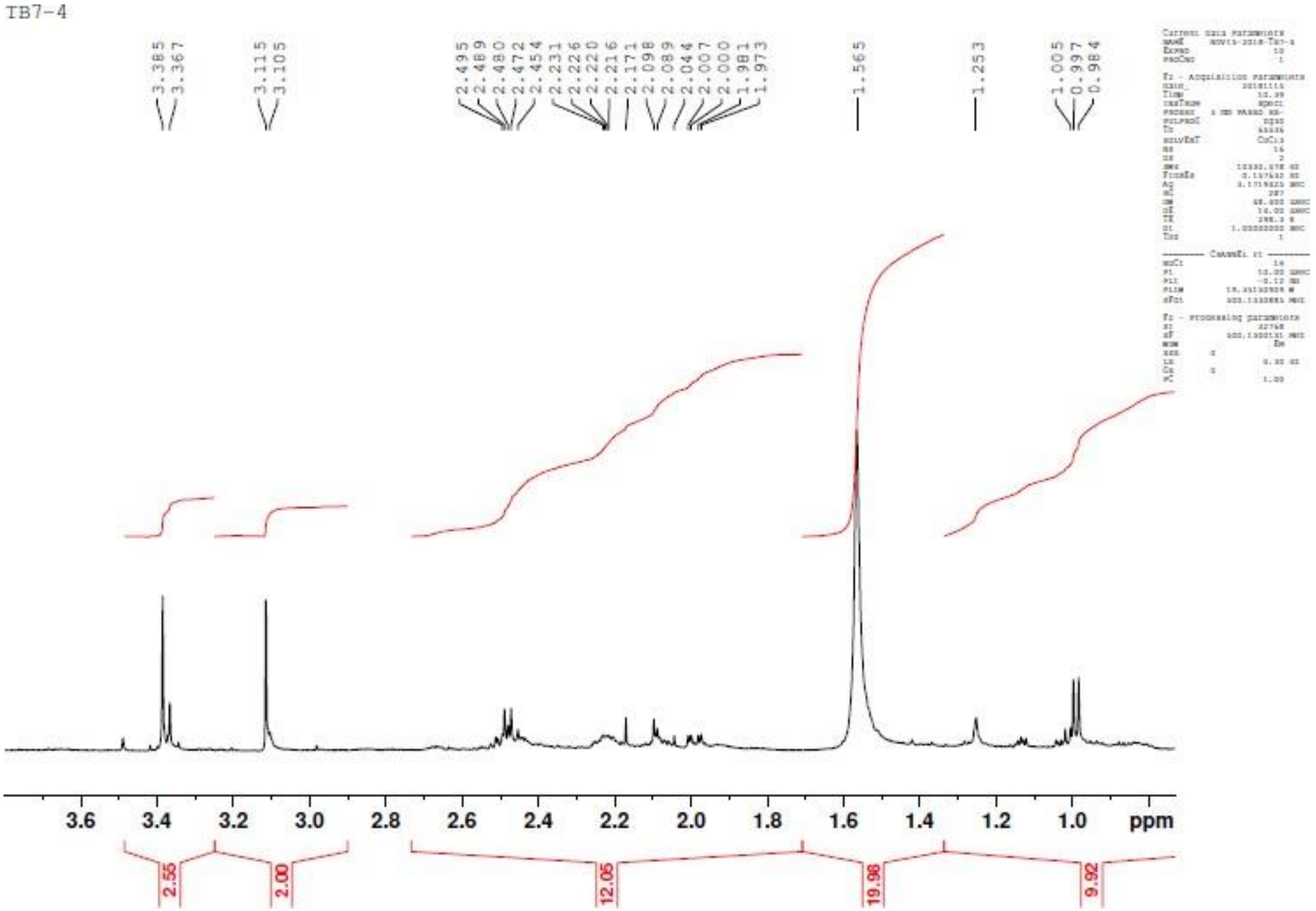

Figure S187. Expansion of part of ^{1}H NMR spectrum (δ 0.8 – δ 3.7) of **18** in $CDCl_3$.

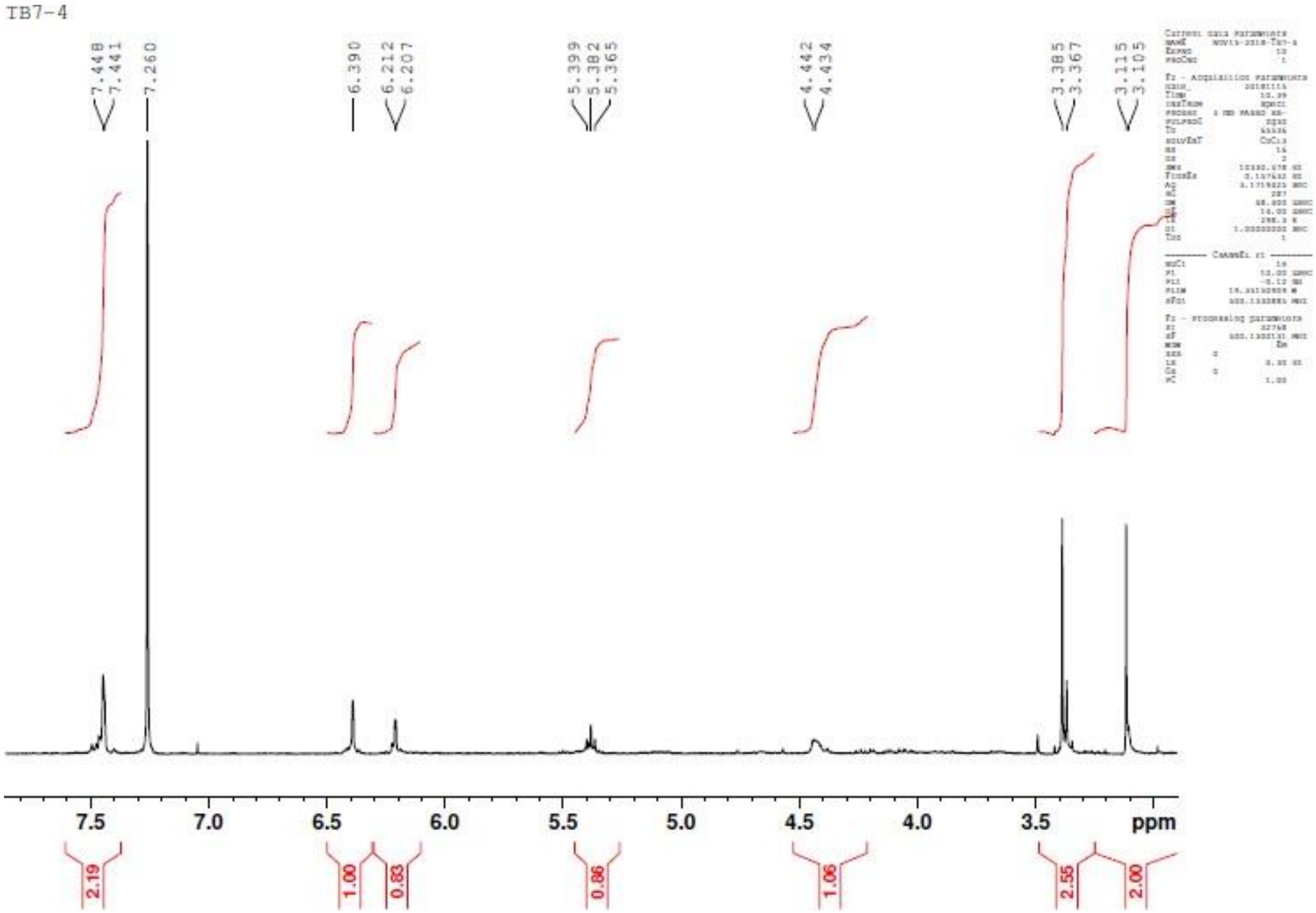

Figure S188. Expansion of part of ^{1}H NMR spectrum (δ 3.0 – δ 7.8) of **18** in $CDCl_3$.

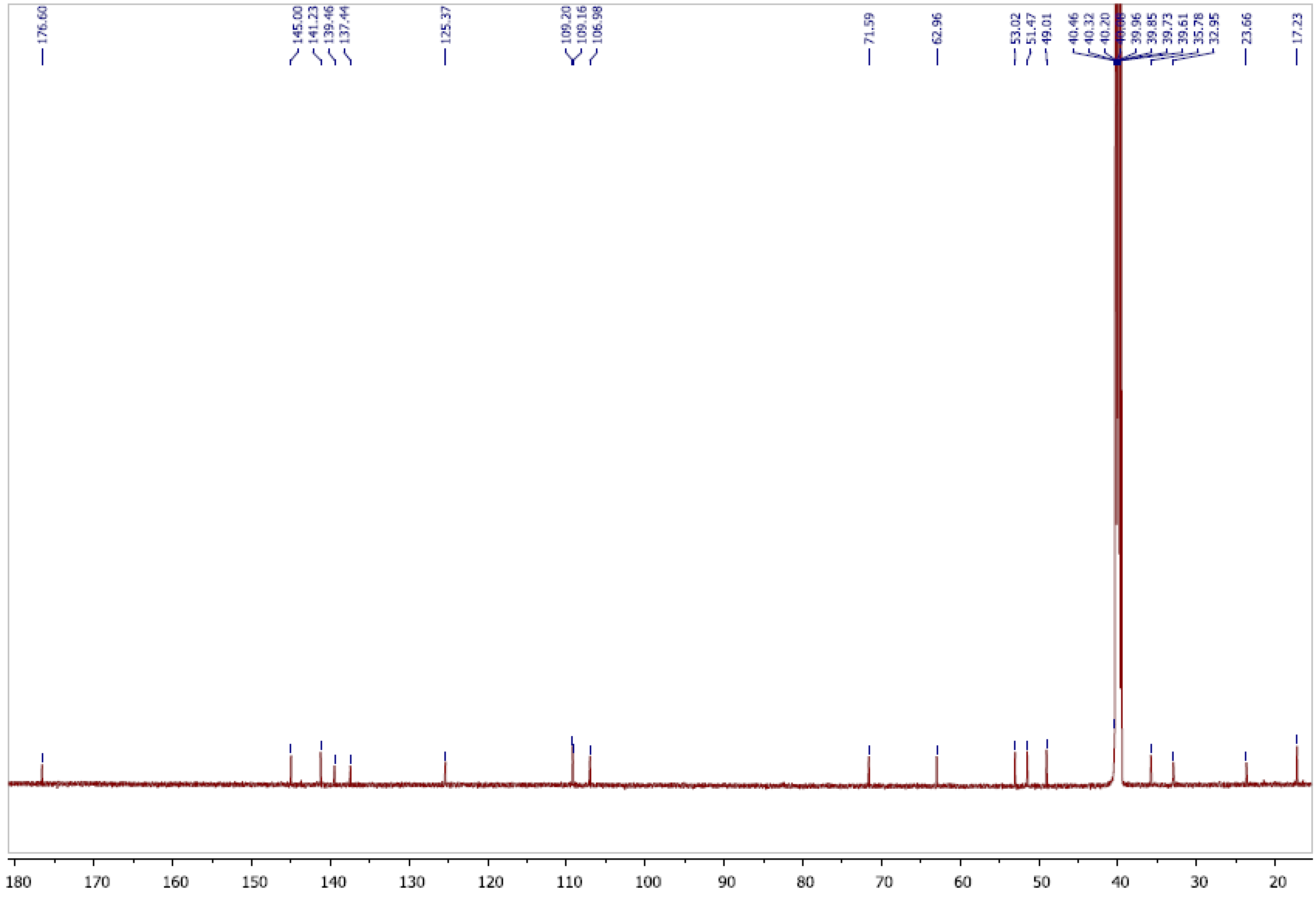

Figure S189. ^{13}C NMR spectrum of **18** in $(CD_3)_2SO$.

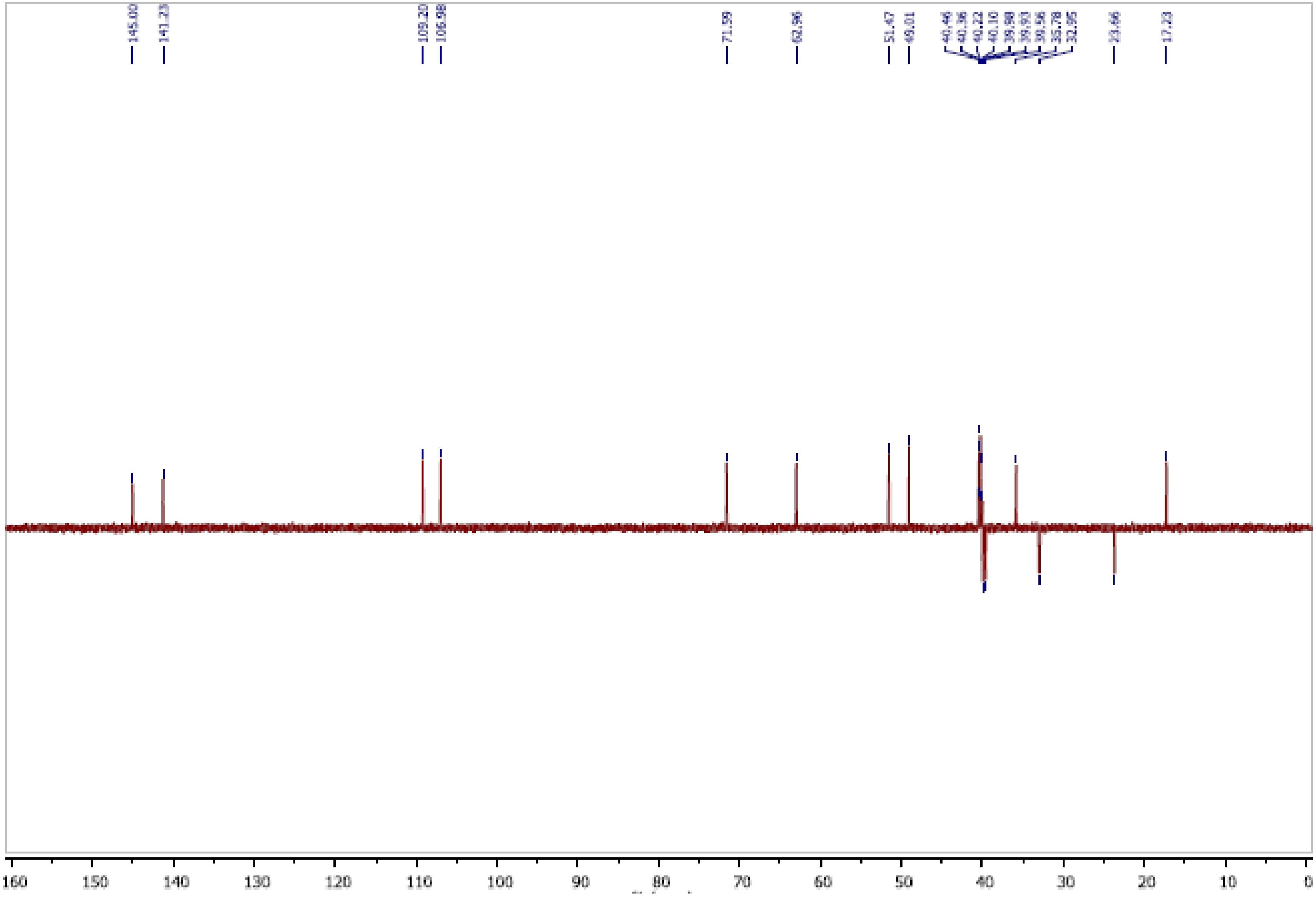

Figure S190. 135DEPT NMR spectrum of **18** in $(CD_3)_2SO$.

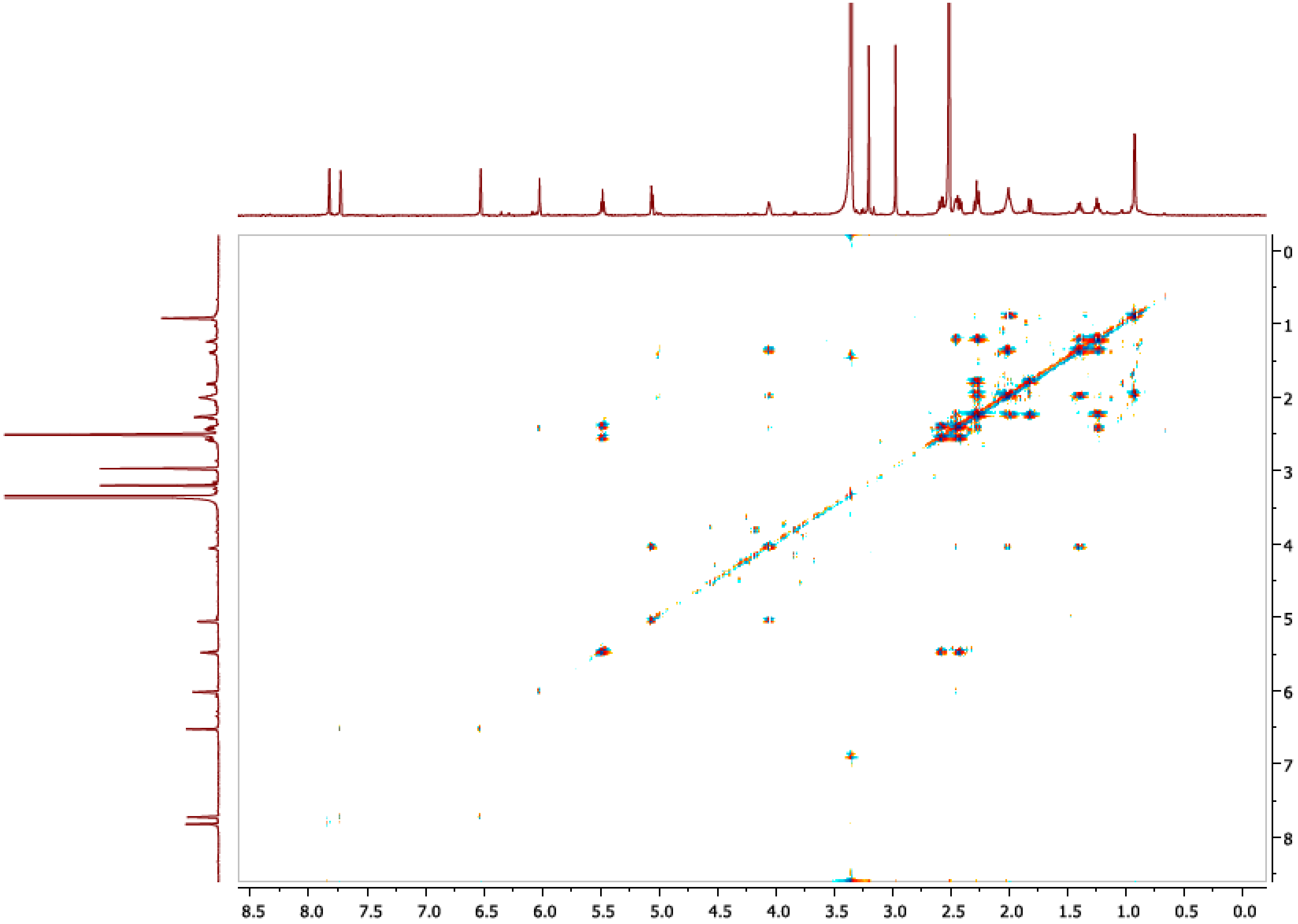

Figure S191. COSY NMR spectrum of **18** in $(CD_3)_2SO$.

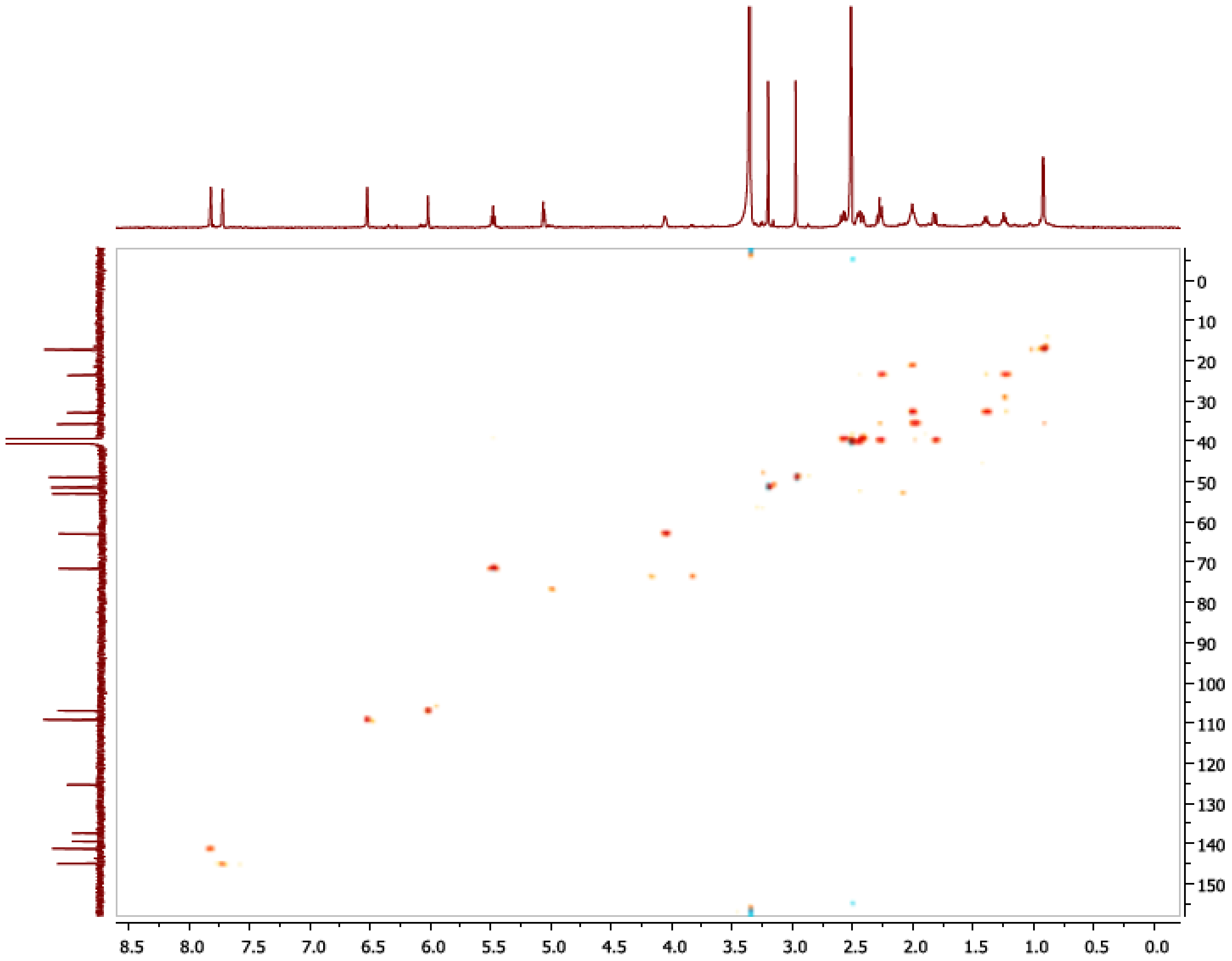

Figure S192. HSQC NMR spectrum of **18** in $(CD_3)_2SO$.

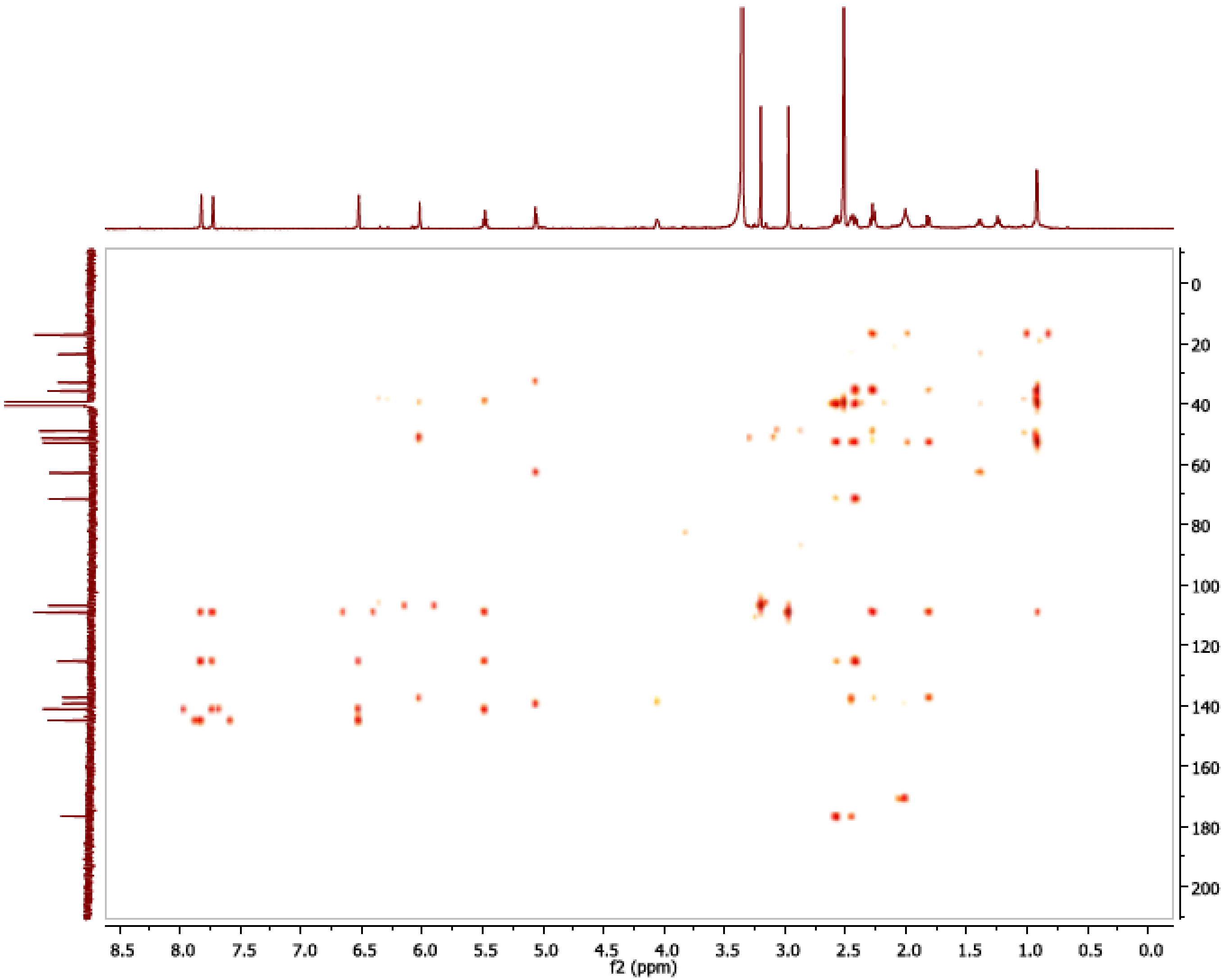

Figure S193. HMBC NMR spectrum of **18** in $(CD_3)_2SO$.

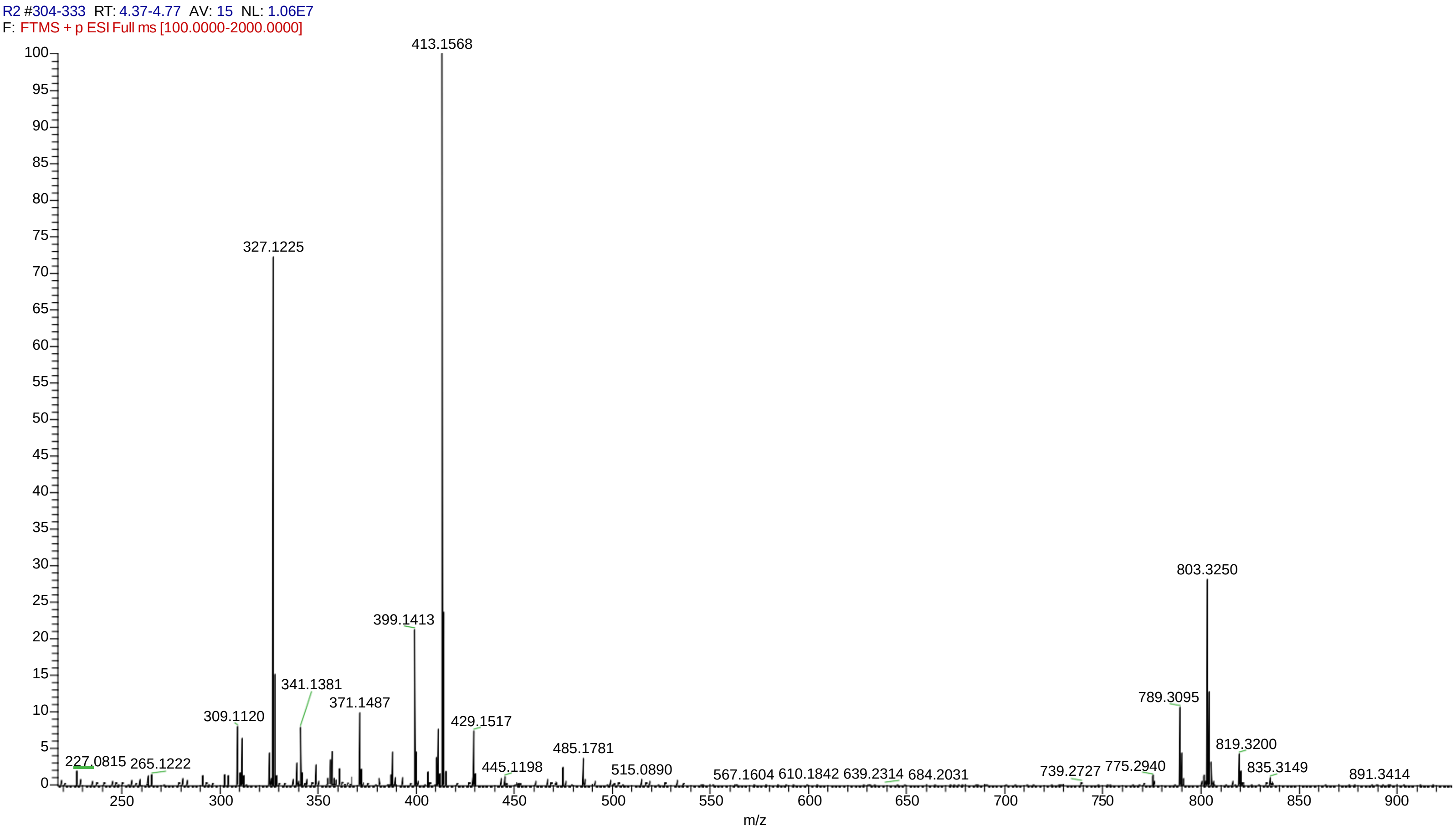

R2 #304-333 RT: 4.37-4.77 AV: 15 NL: 1.06E7
F: FTMS + p ESI Full ms [100.0000-2000.0000]
413.1568
327.1225
399.1413
341.1381
371.1487
309.1120
429.1517
485.1781
445.1198
227.0815
265.1222
515.0890
567.1604
610.1842
639.2314
684.2031
739.2727
775.2940
789.3095
803.3250
819.3200
835.3149
891.3414
m/z

Figure S194. Positive-ion HRESIMS of **18**.

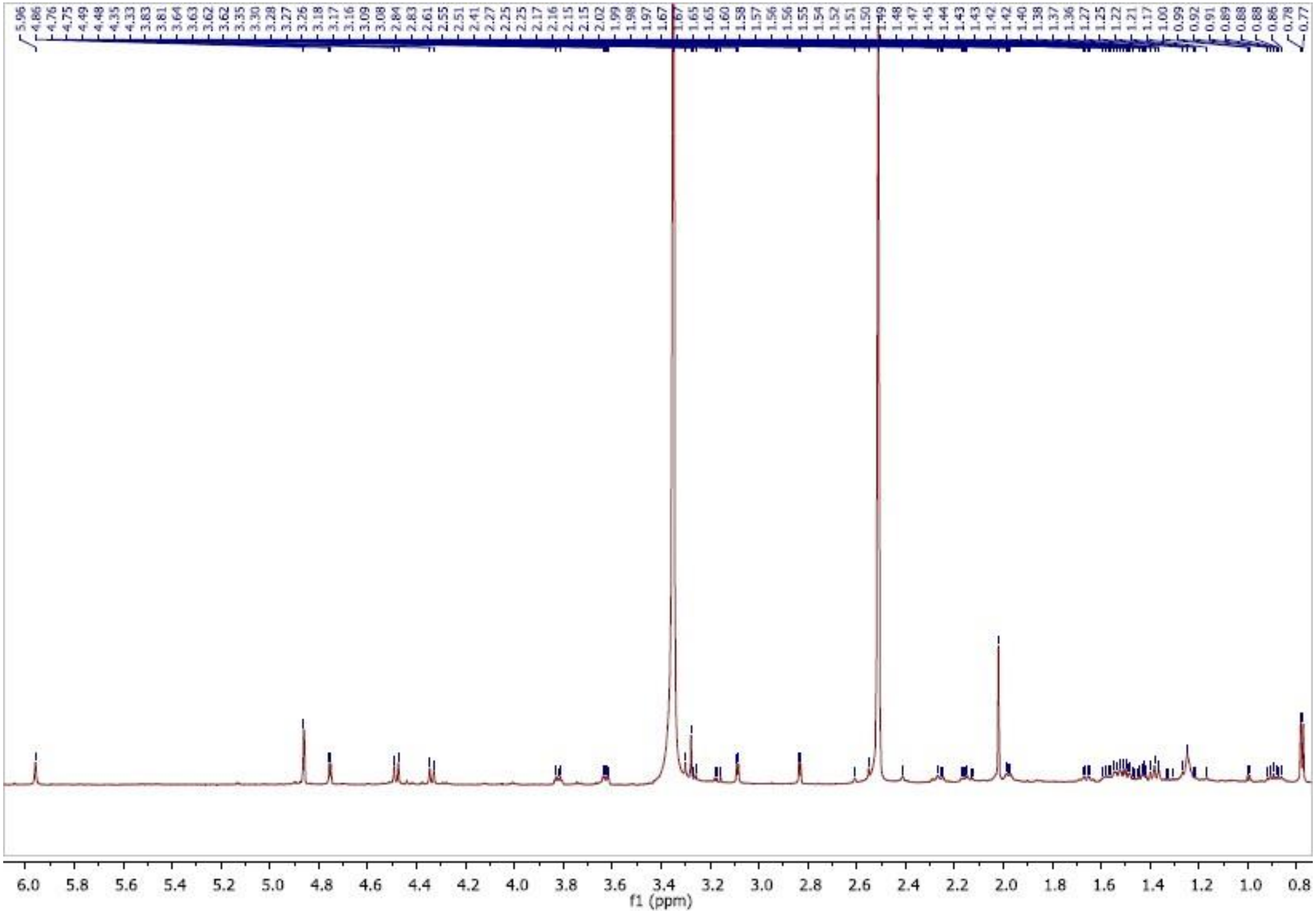

Figure S195. ^1H NMR spectrum of **19** in $(CD_3)_2SO$.

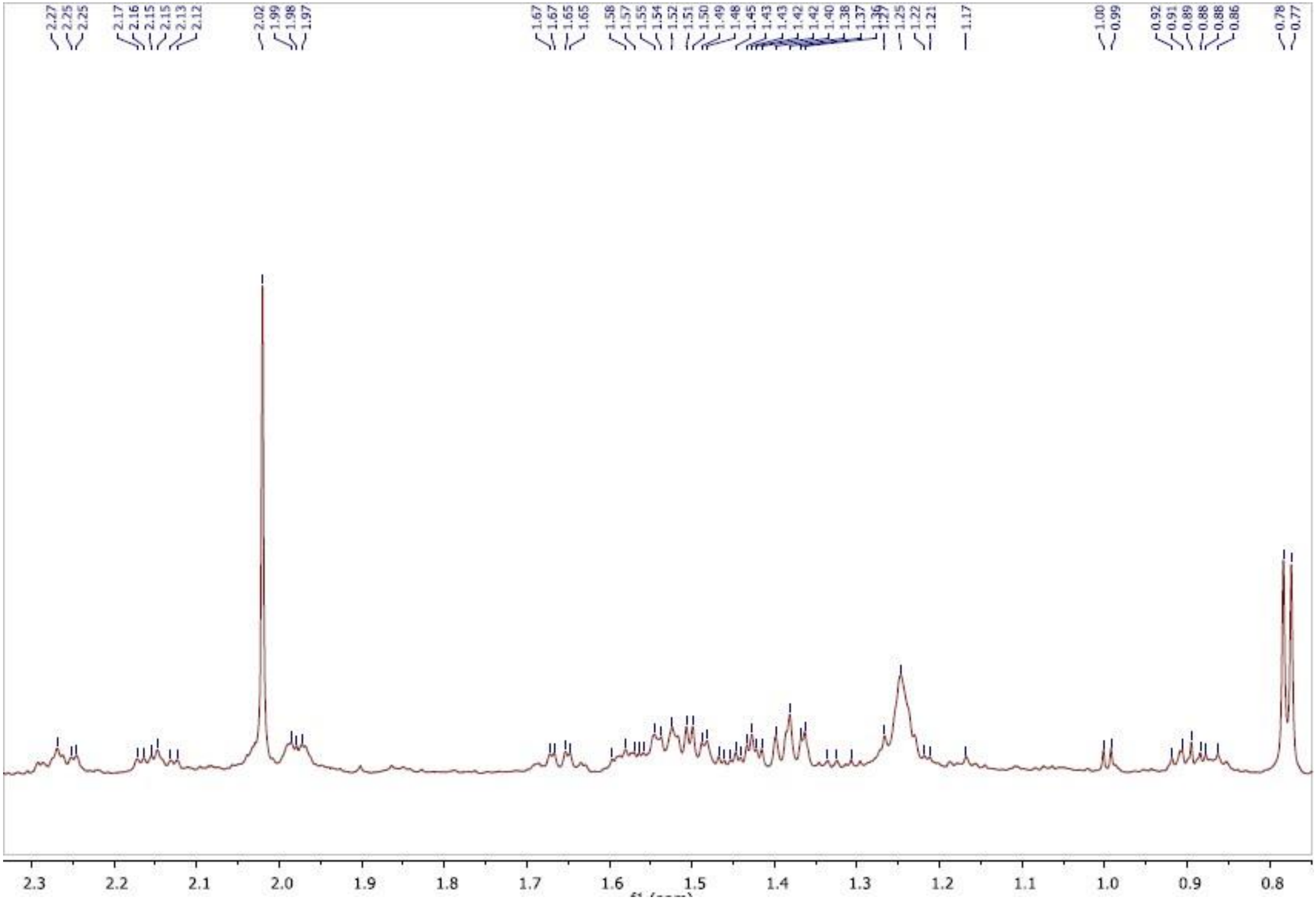

Figure S196. Expansion of part of ^{1}H NMR spectrum (δ 0.75 – δ 2.3) of **19** in $(CD_3)_2SO$.

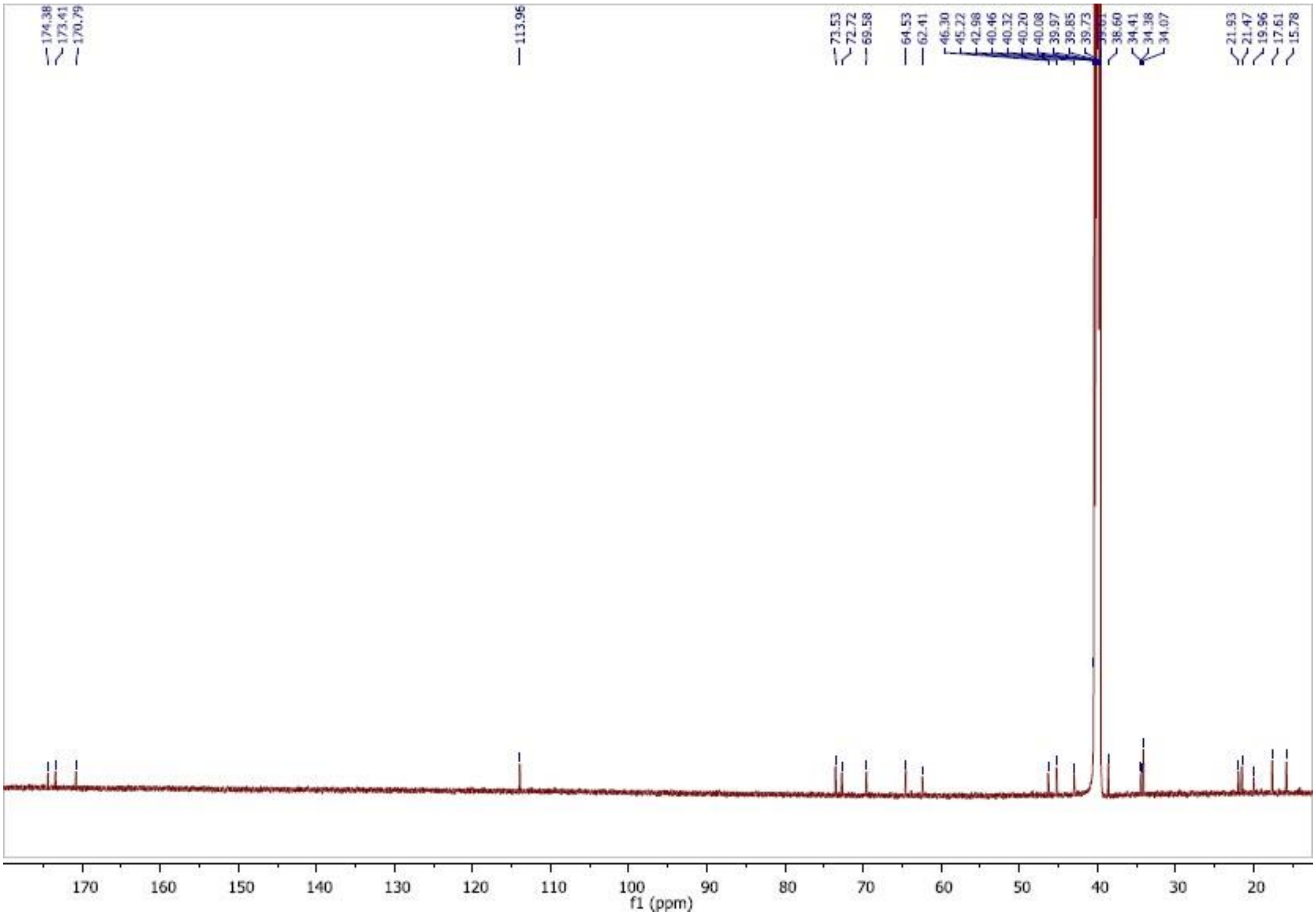

Figure S197. ^{13}C NMR spectrum of **19** in $(CD_3)_2SO$.

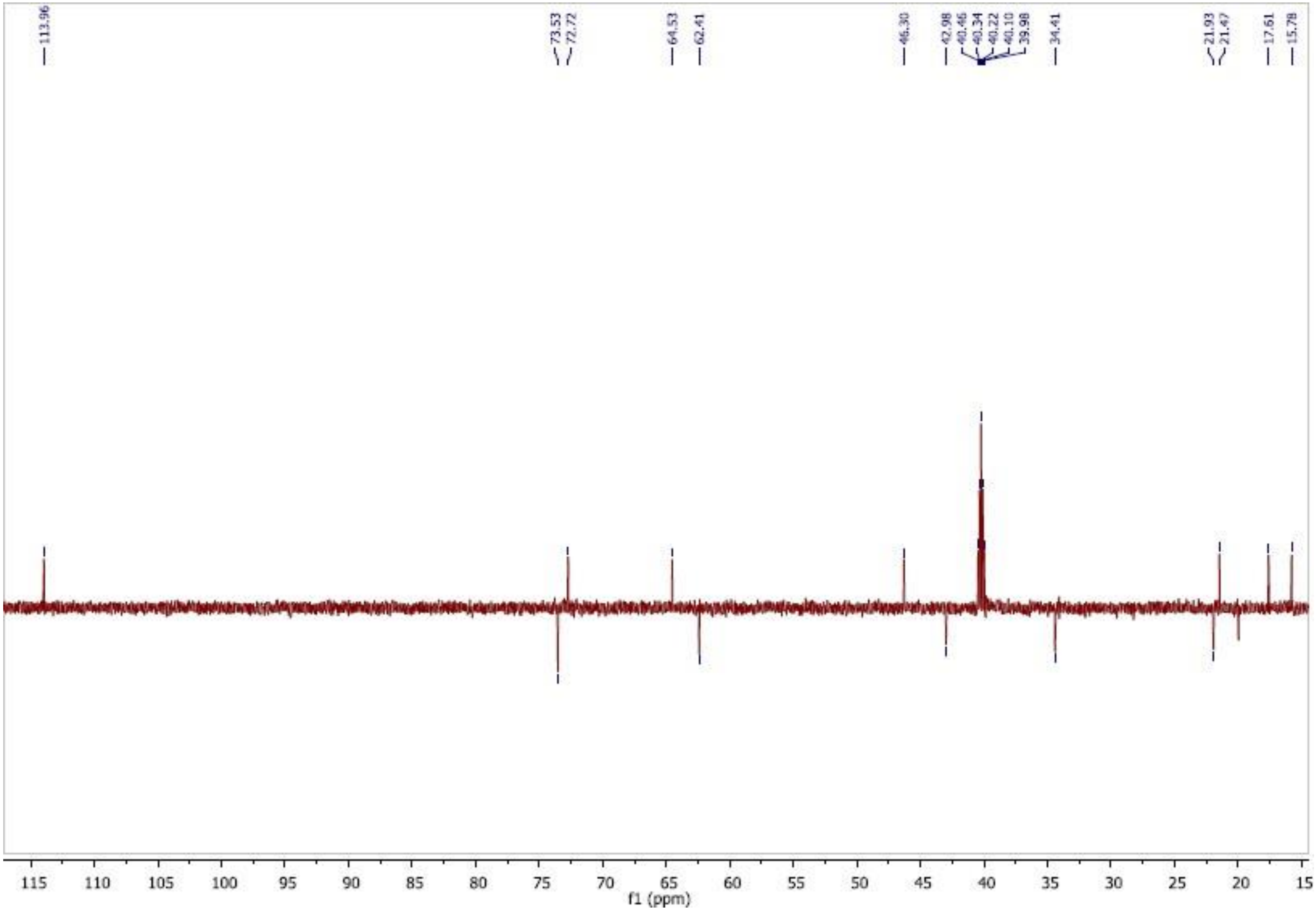

Figure S198. 135DEPT NMR spectrum of **19** in $(CD_3)_2SO$.

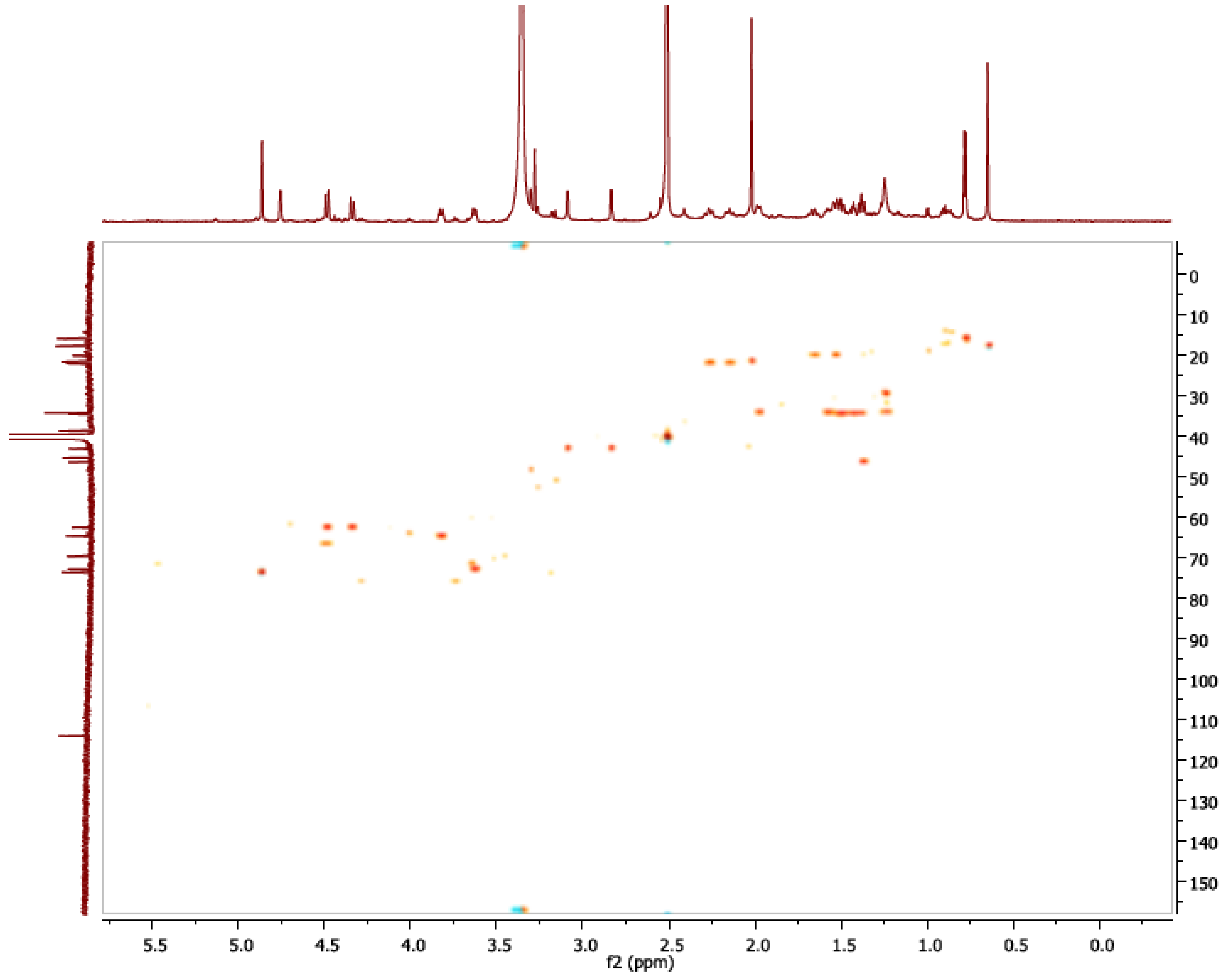

Figure S199. HSQC NMR spectrum of **19** in $(CD_3)_2SO$.

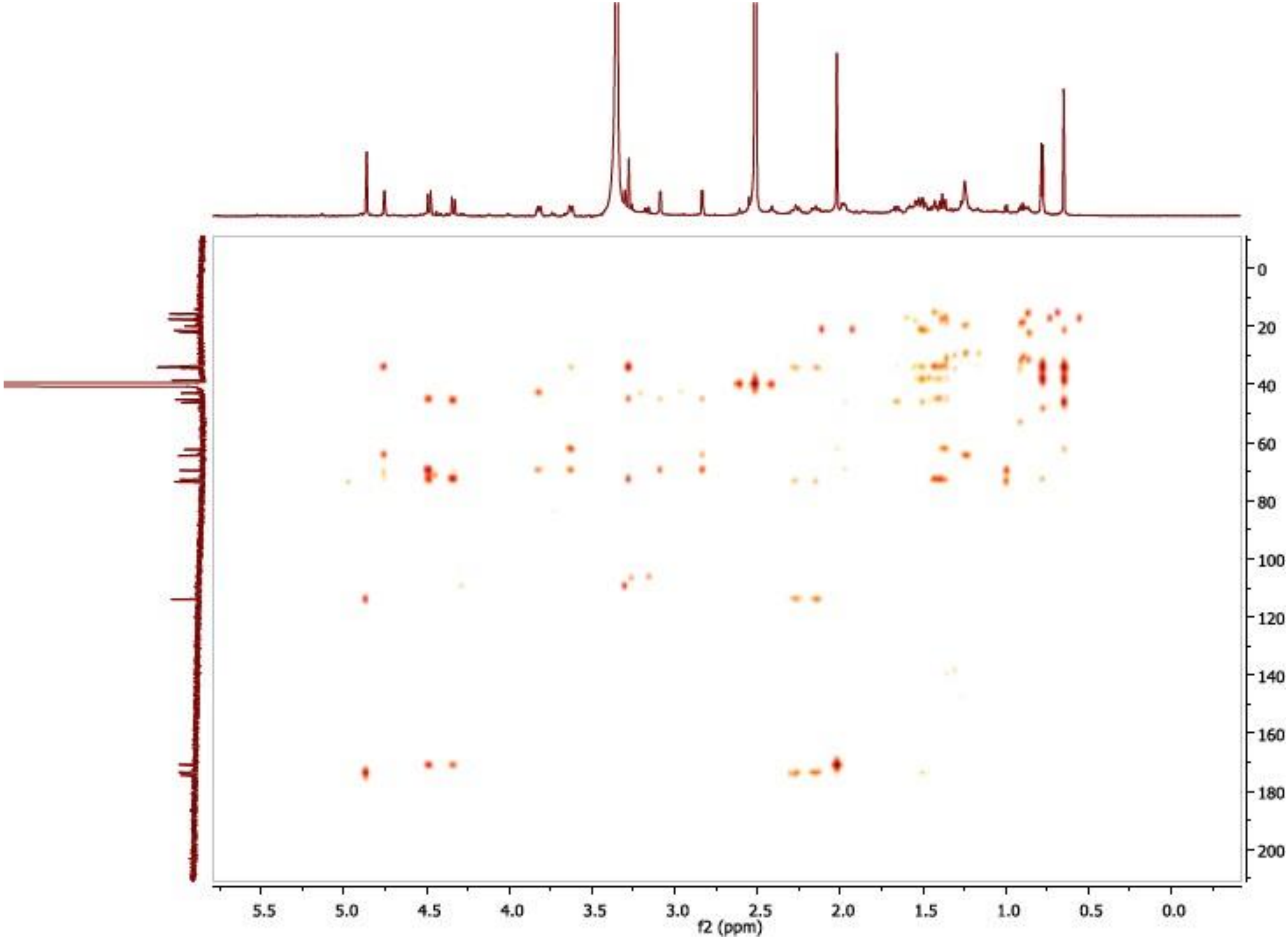

Figure S200. HMBC NMR spectrum of **19** in $(CD_3)_2SO$.

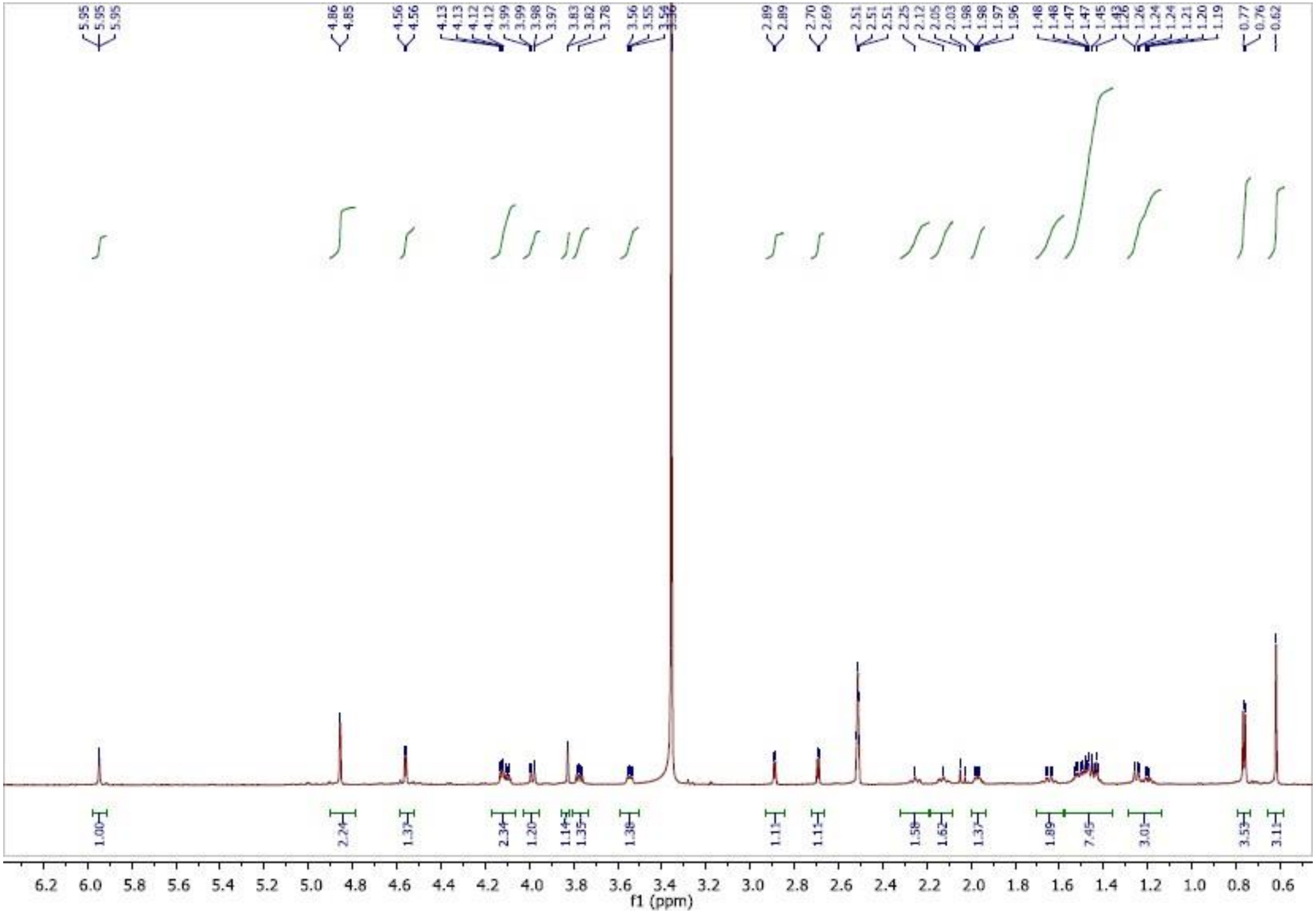

Figure S201. ^1H NMR spectrum of **20** in $(CD_3)_2SO$.

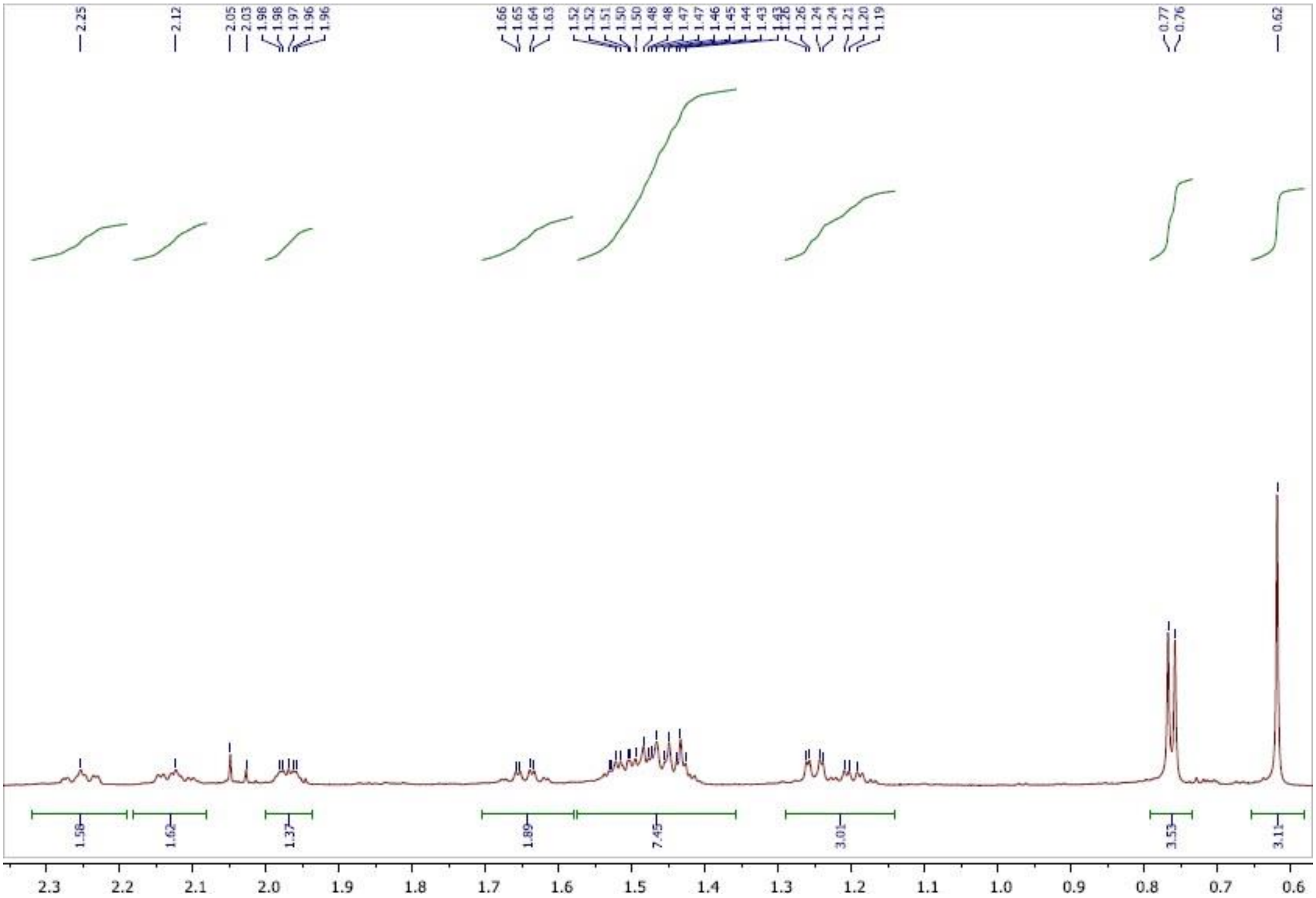

Figure S202. Expansion of part of ^{1}H NMR spectrum (δ 0.6 – δ 2.3) of **20** in $(CD_3)_2SO$.

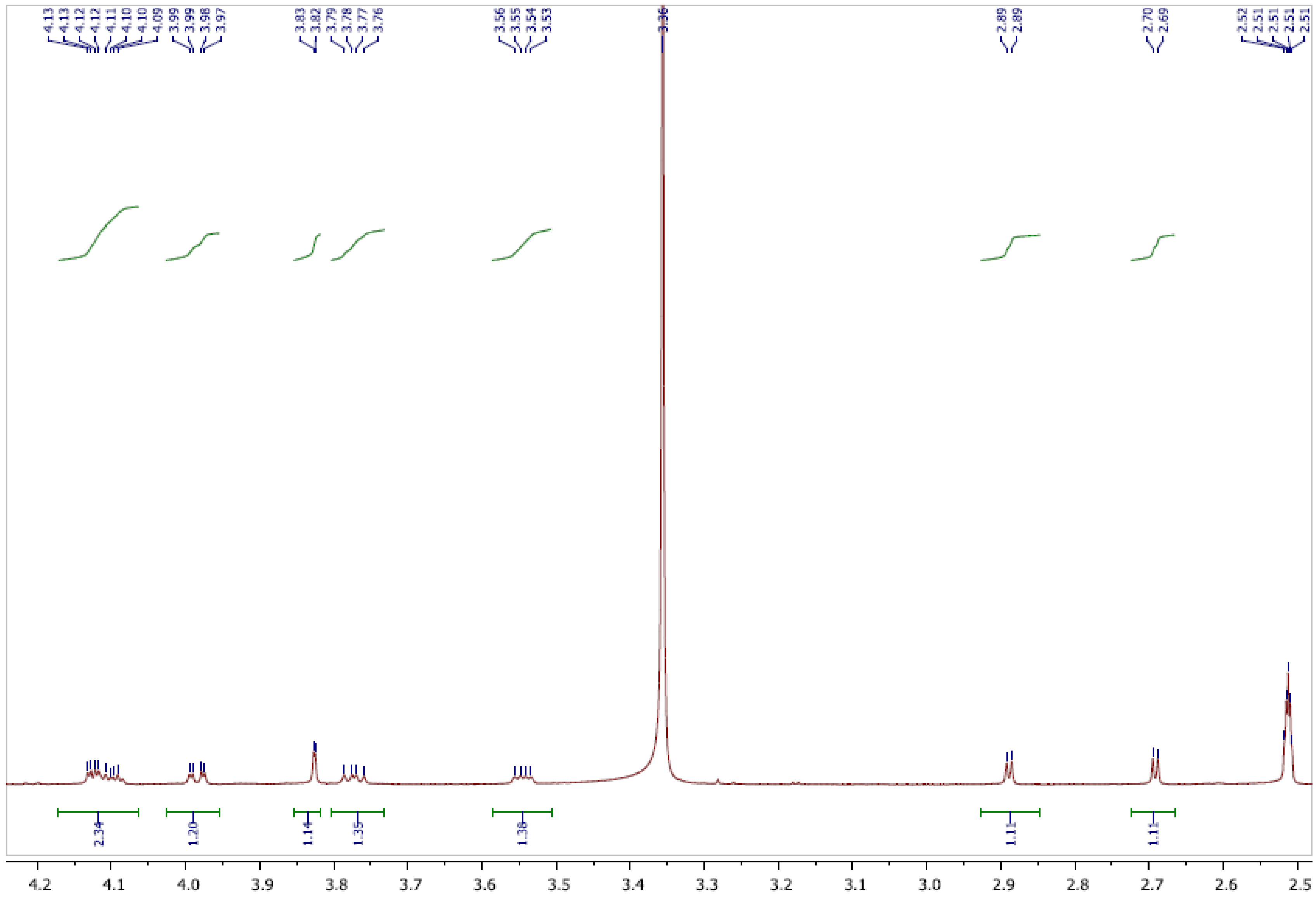

Figure S203. Expansion of part of ^{1}H NMR spectrum (δ 2.5 – δ 4.2) of **20** in $(CD_3)_2SO$.

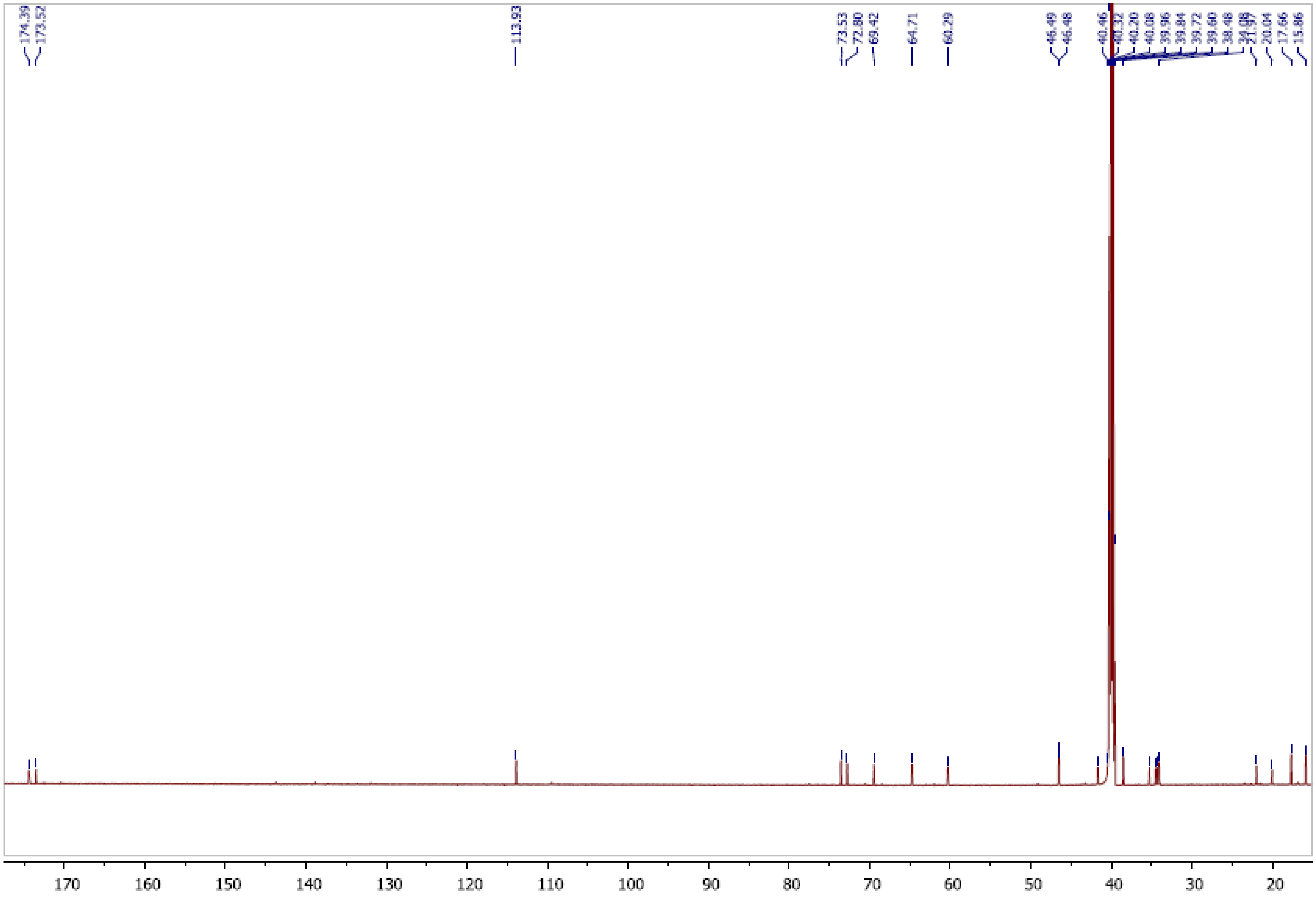

Figure S204. ^{13}C NMR spectrum of **20** in $(CD_3)_2SO$.

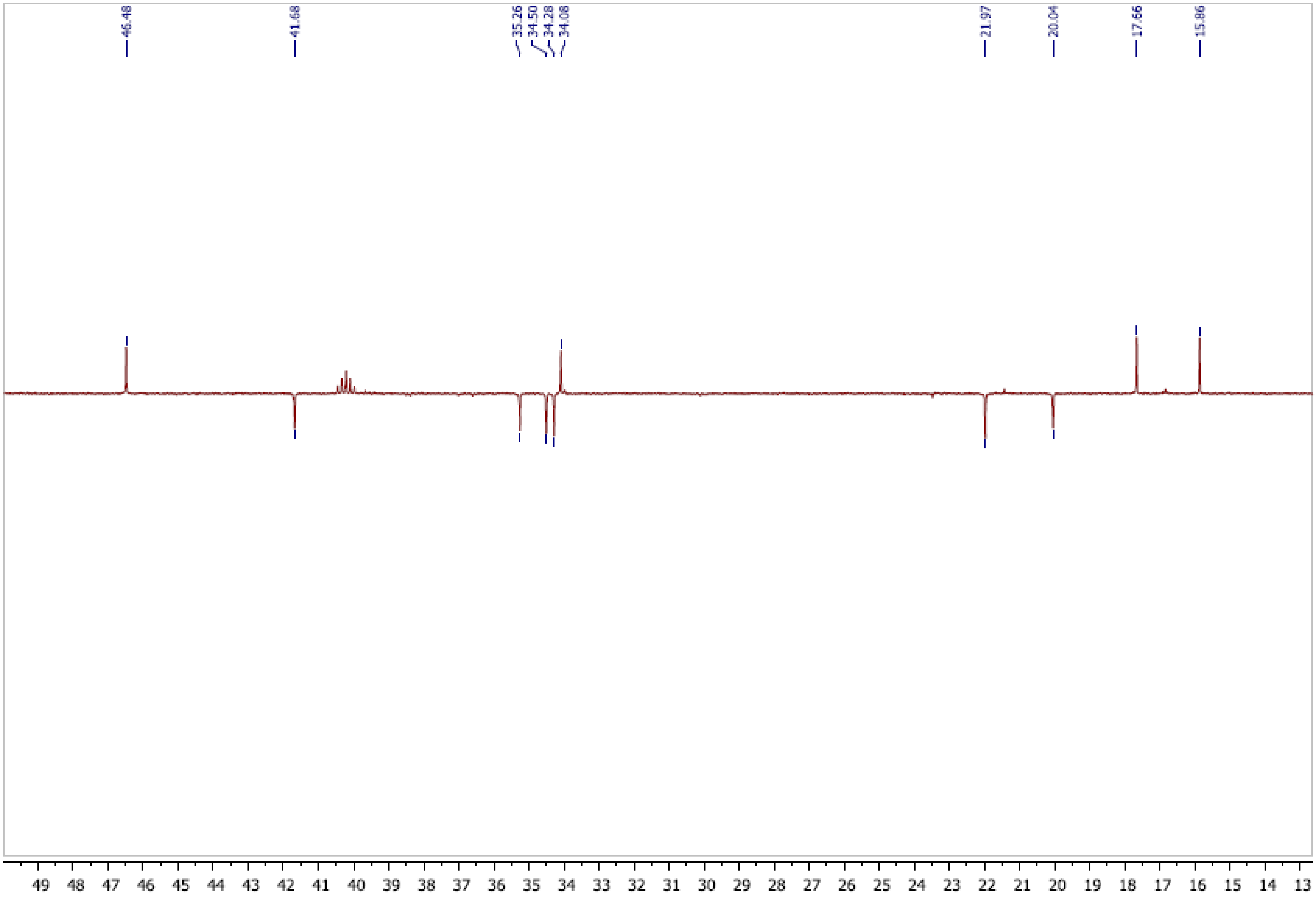

Figure S205. 135DEPT NMR spectrum of **20** in $(CD_3)_2SO$.

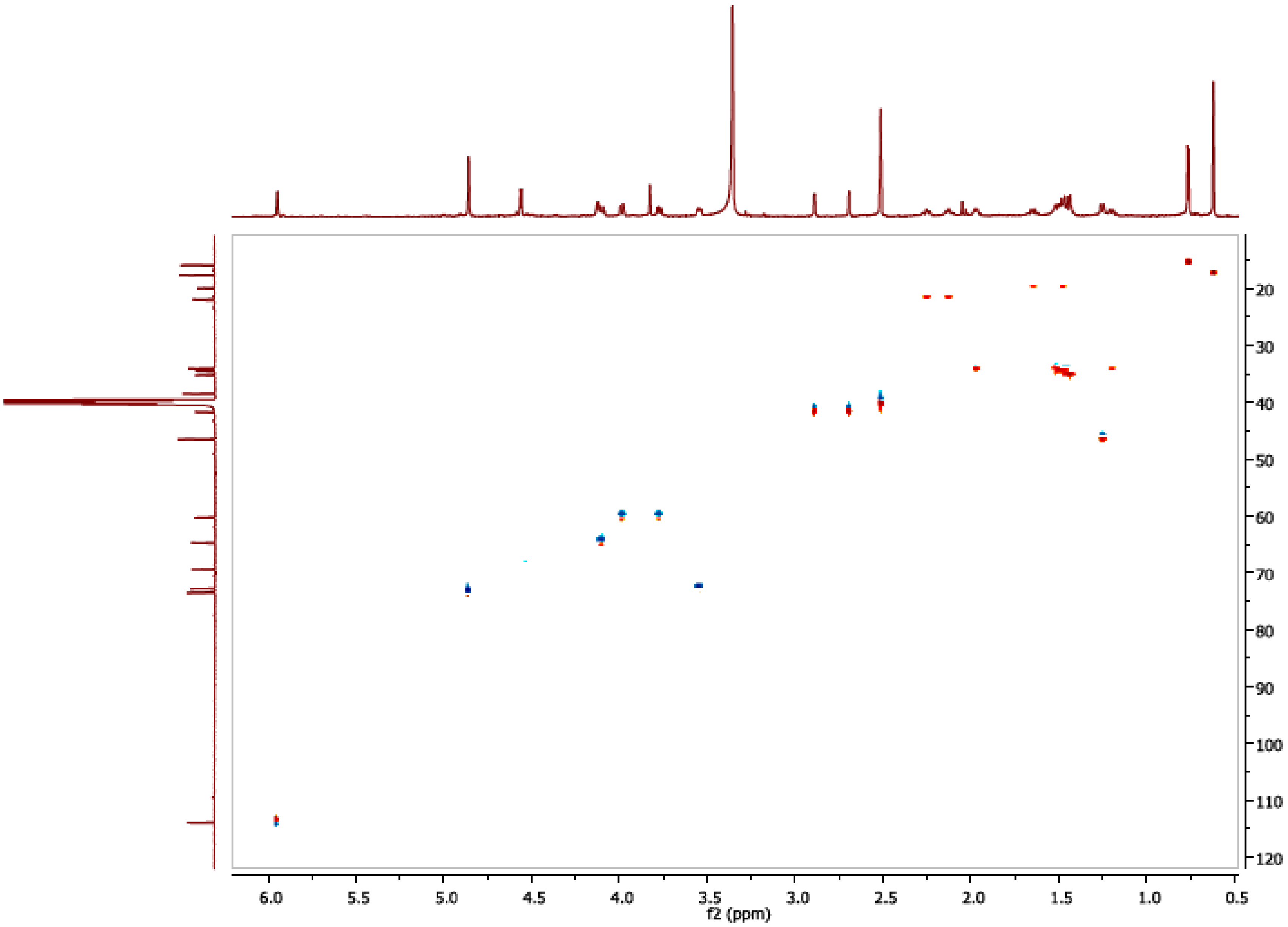

Figure S206. HSQC NMR spectrum of **20** in $(CD_3)_2SO$.

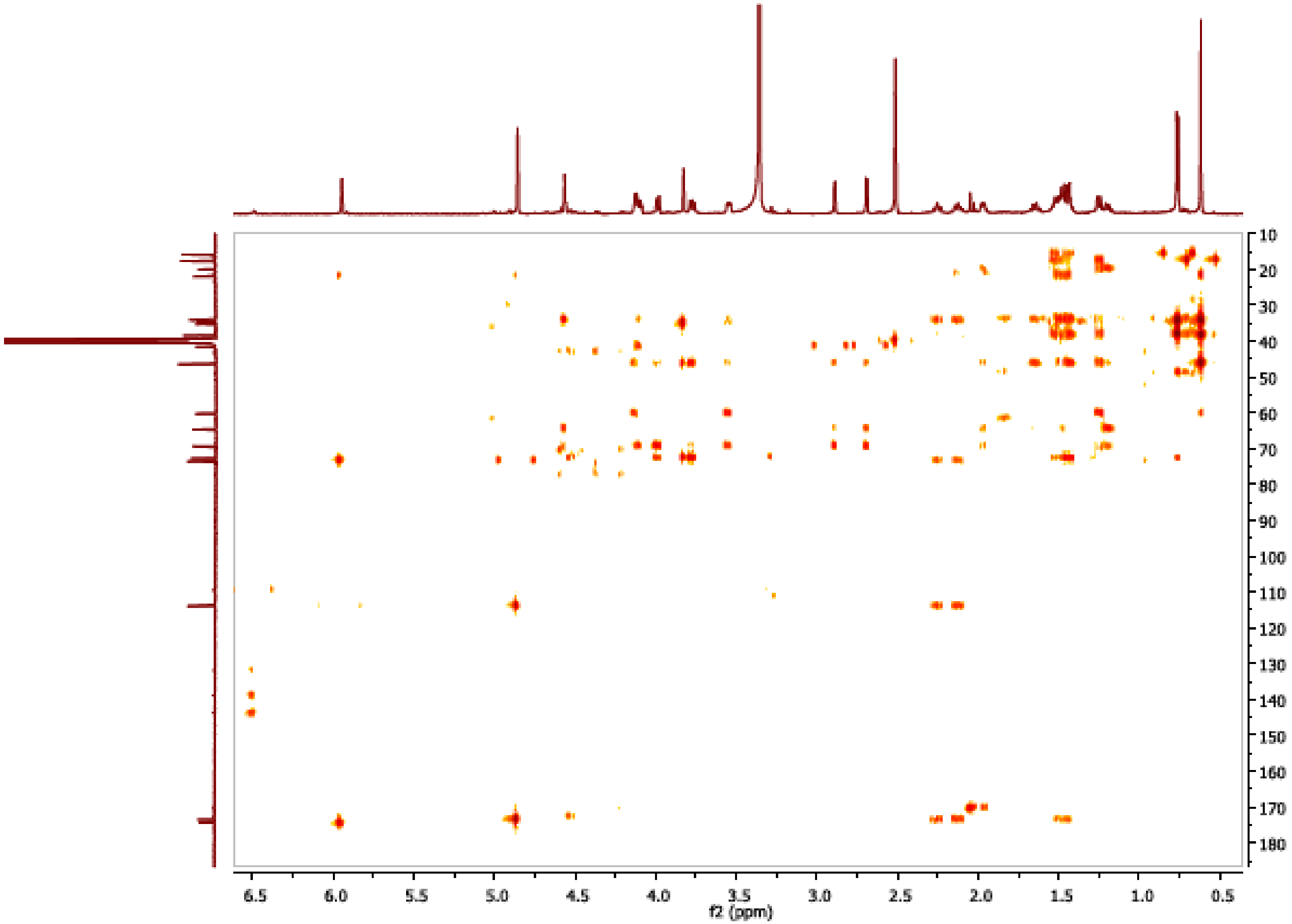

Figure S207. HMBC NMR spectrum of **20** in $(CD_3)_2SO$.

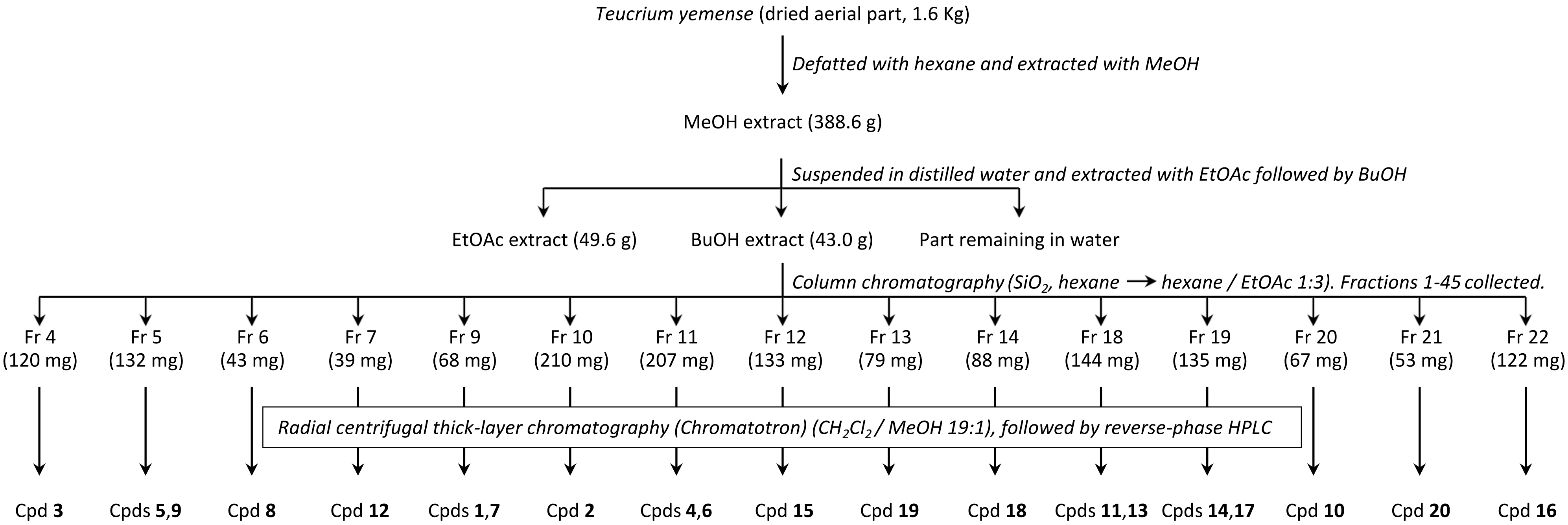

Figure S208. Fractionation tree for isolation of compounds **1**-**20**.

General analytical and chromatographic procedures.

The 1D and 2D NMR experiments (^{1}H, ^{13}C, COSY, NOESY, HSQC and HMBC) were performed on Bruker Avance spectrometers (500 MHz or 400 MHz for ^{1}H and 175 MHz for ^{13}C NMR spectra), using the residual solvent signals as internal standards. Conventional pulse sequences were used for COSY, NOESY, HSQC and HMBC. Coupling constants (*J*) are stated in Hz. Ultra-high accuracy mass analysis was done on a Nano-Flow linear trap quadrupole Fourier Transformation Ion Cyclotron Resonance Mass Spectrometer Ultra (FT-ICR-MS) ("ultra" refers to the high-sensitivity ICR cell) (Triversa Nanomate; Advion Biosciences Limited, Norfolk, UK). Samples were reconstituted in 70% aq. MeOH (100 mL). Samples were vortexed and were centrifuged for 4 min at 13,000 rpm at 0°C. Aliquots (20 mL) of supernatant was then transferred to a clean well on a 128- well plate. The sample (13 mL) was injected by the nano-flow injection system, with an aliquot (5.0 mL) being delivered to the ICR cell. Gas pressure was maintained at 0.5 psi with an applied voltage of 1.5 KV to maintain a consistent current of 60-120 nA. When operating in narrow SIM mode, the resolution was 100,000 and the scan window 30 Da. Each scan was acquired in 60 sec. IR spectra were recorded on a Perkin- Elmer FTIR 600 series spectrometer. Column chromatography was performed using normal-phase silica gel (Merck; 230-400 mm). HPLC was performed on a Shimadzu system (Kyoto, Japan), consisting of two LC-6AD Semi-Preparative Solvent Delivery pumps coupled with Rheodyne manual injector, communications bus module CBM-20A, a multi-wavelength photo-diode array detector (SPD-M20A), FRC-10A fraction collector, all connected to a computer system with Intel Core DUO with Microsoft XP and Shimadzu's LC solution software. It was fitted with two columns, Shim-pack PREP-ODS (H) Kit (A) 250 mm × 4.6 mm i.d., 5 mm particles (B) 250 mm × 20 mm i.d., 5 mm. Analytical HPLC was performed using the column under gradient conditions with a mobile phase consisting of MeCN and water programmed linearly to 100% MeCN over 35 min at the flow rate 1.0 mL min^{-1}. The UV detection wavelengths were 210 and 254 nm. The chromatographic separation HPLC was performed using column and preparative HPLC conditions as for analytical HPLC, except the flow rate was 20 mL min^{-1}.

Assay for enhancement of insulin secretion.

Tolbutamide (TB), collagenase V and bovine serum albumin (BSA) were obtained from Sigma (St. Louis, MO, USA). The mouse ultrasensitive insulin ELISA kit was purchased from Crystal Chem Inc. (IL, USA).

Isolation of murine pancreatic islets. Primary pancreatic islets were isolated from male BALB/c mice (28-36 g) from the ICCBS Animal Research Facility. The animal handling and tissue harvesting were done in accordance with the requirements of the Institutional Animal Care and Use Committee of ICCBS, University of Karachi, Pakistan. Pancreatic islets were isolated by digestion with collagenase V and islet-picking, as described earlier.[50,51] Mice were anaesthetized with sodium thiopental (30 mg $Kg)^{-1}$ and the distended whole pancreas was digested at 37°C in collagenase solution (1.0 mg mL^{-1}) for 15 min. The digested islets were further purified by centrifugation at 1000 rpm for 1 min, followed by filtration using a pre-wetted 70 μm cell strainer. Islets were hand-picked with a siliconized Pasteur pipette under a NIKON SMZ-745 stereomicroscope. The isolation and purification medium used was Hank's Balanced Salt Solution (HBSS) without Ca^{2+}, Mg^{2+} and phenol red.

Assay. Freshly isolated islets (n = 3) of similar diameter (120–160 μm) were pre-incubated for 45 min at 37°C in Krebs-Ringer bicarbonate (KRB) buffer solution containing 0.1% BSA and glucose (3.0 mM) in Eppendorf tubes, and each condition was triplicated. Then islets were washed with KRB buffer before transferred into KRB buffer (300 μL) containing glucose (16.7 mM), supplemented with either test compound (200 μM) or tolbutamide (positive control) (200 μM) and incubated at 37°C for 1 h. After incubation, an aliquot (200 μL) of the mixture was cooled to -40°C until it could be assayed for released insulin. The concentrations of insulin in the collected aliquots were measured using the mouse ultrasensitive insulin ELISA kit according to the high-range protocol. The reading is the concentration of insulin in ng mL^{-1} of medium. After calculation of the absolute amount of insulin in the medium, this amount was divided by three (the number of islets in the tube).

References

50. Siddiqui, B. S. *et al.* Two new compounds from the aerial parts of *Bergenia himalaica* Boriss and their anti-hyperglycemic effect in streptozotocin-nicotinamide induced diabetic rats. *J. Ethnopharmacol*. **152**, 561-567 (2014).

51. Hafizur, R. M., Hameed, A., Shukrana, M., Kabir, N., Chishti, S. Cinnamic acid exerts anti-diabetic activity by improving glucose tolerance *in vivo* and stimulating insulin secretion *in vitro*. *Phytomed. 22*, 297-300 (2015).
